# First *Tetraploa* Genome and Multi‐Omics Analysis Reveal Key Plant‐Microbe‐Soil Interactions for Salt Tolerance and Yield Improvement of Wheat

**DOI:** 10.1111/pbi.70663

**Published:** 2026-04-03

**Authors:** Cheng‐Wei Qiu, Shuo Zhang, Zi‐Feng Gao, Zhong‐Hua Chen, Chulong Zhang, Mohamed Abdelalim Ali, Feibo Wu

**Affiliations:** ^1^ Department of Agronomy, College of Agriculture and Biotechnology Zijingang Campus, Zhejiang University Hangzhou China; ^2^ School of Pharmaceutical Sciences Fuchun Campus, Zhejiang Chinese Medical University Hangzhou China; ^3^ School of Science Western Sydney University Penrith New South Wales Australia; ^4^ School of Agriculture, Food & Wine The University of Adelaide Adelaide South Australia Australia; ^5^ Ministry of Agriculture and Rural Affairs Key Laboratory of Molecular Biology of Crop Pathogens and Insect Pests, Zhejiang Key Laboratory of Biology and Ecological Regulation of Crop Pathogens and Insects of Zhejiang Province Institute of Biotechnology, Zhejiang University Hangzhou China; ^6^ Faculty of Agriculture, Microbiology Department Cairo University Giza Egypt

**Keywords:** genome sequencing, halotolerant endophyte, metabolome, microbiome, transcriptome, *Triticum aestivum*

## Abstract

Salinity is a major threat to global agricultural productivity of staple crops such as wheat. Although microbial‐based solutions hold promise for alleviating salinity stress, practical implementation is hindered by insufficient mechanistic characterization of bioinoculants and their interactions with plants. Here, we assembled the first complete reference genome of a halotolerant strain within the genus *Tetraploa*—the endophytic fungus *Tetraploa* sp. E00680. This novel genomic resource serves as a foundation for exploring previously uncharacterised salt tolerance mechanisms in this potential fungal inoculant. Our research demonstrates that E00680 enhances wheat yield under both controlled and field saline conditions. We found that E00680 systematically modulates the plant‐microbe‐soil interactions by optimizing rhizosphere microbial communities, increasing nutrient bioavailability, and triggering coordinated transcriptional and metabolic reprogramming in wheat. Notably, E00680 expands tryptophan metabolism to synergistically boost auxin biosynthesis in wheat by supplying precursors and activating relevant metabolic pathways. This cross‐kingdom metabolic coupling facilitates better growth and salt tolerance in wheat plants. Our findings offer multi‐omics and rhizosphere‐level insights that can guide the development of microbial inoculants to enhance climate‐resilient and sustainable crop production.

## 1 Introduction

Soil salinization is an increasing threat to global agricultural sustainability and food security. It currently affects 19.5% of irrigated croplands worldwide, with projections indicating that 50% of agricultural land may succumb to salinization by 2050 as its impact is expected to intensify under the changing climate (Dissanayake et al. 2024; Kumar et al. 2024). Wheat ( *Triticum aestivum* ), a staple crop essential for nearly 30% of global human caloric intake, is particularly vulnerable to salinity‐induced yield losses (Wang, Cheng, et al. 2024). These facts underscore the urgent need to improve wheat's salt tolerance as a critical agricultural priority in response to climate change and to ensure food security for a booming global population.

Rhizosphere engineering using plant growth‐promoting microorganisms (PGPMs) has emerged as a promising and ecologically sustainable alternative, particularly given that molecular breeding efforts to enhance salinity tolerance face challenges from polygenic inheritance, complex field conditions, and genotype‐environment interactions (Wang and Song 2022; Zeng et al. 2025). This biological approach offers distinct advantages over genetic engineering methods, including fewer regulatory hurdles, enhanced natural adaptation to fluctuating environmental conditions, and broader social acceptance (Crowther et al. 2024; Feng et al. 2025). Despite this potential, considerable knowledge gaps remain. While recent studies reveal that PGPMs enhance the adaptive plasticity of plants under salinity stress, successful translation is largely limited by experimental constraints (Schmitz et al. 2022; Zheng et al. 2024). One major limitation is that most studies are confined to crops at early vegetative growth stages within controlled greenhouse environments. Consequently, these studies fail to capture long‐term yield outcomes and overlook the intricate, multi‐directional dynamics of the plant‐microbe‐soil continuum in real‐world agricultural settings (Feng et al. 2024; Yang et al. 2024). Furthermore, current research largely relies on a few well‐documented microbial genera, which restricts the discovery of novel, highly efficient salt‐tolerant PGPMs. Hence, it is imperative to identify novel salt‐tolerant PGPM strains and evaluate their efficacy under actual field conditions.

Endophytic PGPMs contribute to plant resilience by inducing metabolic reprogramming in their hosts—a key strategy for combating abiotic stress (Li et al. 2022; Liu, Shi, et al. 2024). However, the specific cross‐kingdom mechanisms orchestrating this process remain a significant black box. Root‐associated microbiota play a crucial role in nutrient acquisition (e.g., phosphate, nitrogen, and iron), modulation of hormone signalling (auxins, ethylene, and abscisic acid), accumulation of osmolytes and antioxidants, and restructuring of soil microbiomes (Hiruma et al. 2016; Arora et al. 2020; Korenblum et al. 2022). While the genetic pathways underlying endophyte‐mediated salt tolerance have been elucidated in model plant species such as *Arabidopsis* (Andrés‐Barrao et al. 2021), poplar (Li et al. 2024), and rice (Wang, Li, et al. 2024), the comprehensive multi‐omics networks governing salinity adaptation in complex crops like wheat remain poorly understood. Additionally, studies on novel stress‐tolerant endophytes frequently prioritize plant physiology and rhizosphere ecology while neglecting the endophytes' intrinsic genomic blueprints.

To bridge these critical gaps, we hypothesize that the unique genome of the halophytic fungus *Tetraploa* sp. E00680 (hereafter E00680), along with specific metabolites and genes induced by this endophytic PGPM, plays pivotal roles in improving wheat salt tolerance via these adaptive mechanisms. Here, we present a novel salinity‐mitigating mechanism mediated by the previously uncharacterized functional traits of the fungal strain E00680, with a particular focus on its cross‐kingdom metabolic regulation within host wheat plants (Figure S1). We first decipher a high‐quality genome assembly for E00680, highlighting the genetic signatures associated with stress adaptation and plant growth promotion. Field and pot trials demonstrate that E00680 significantly enhances wheat biomass and grain yield under saline conditions. By integrating genomic, microbiomic, transcriptomic, and metabolomic analyses, we reveal that fungal‐mediated tryptophan metabolism orchestrates the plant‐microbe‐soil feedback loops during the salinity adaptation of wheat. These findings establish a novel tripartite interaction network involving the fungal endophyte, the host plant, and the soil microbiota within agroecosystems. This work advances the development of microbial bioinoculants aimed at boosting crop productivity in saline soils, which is a critical step toward achieving sustainable agriculture.

## 2 Results

### 2.1 Genome Assembly of the Salt‐Tolerant Endophytic Fungus *Tetraploa* sp. E00680

**FIGURE 1:**
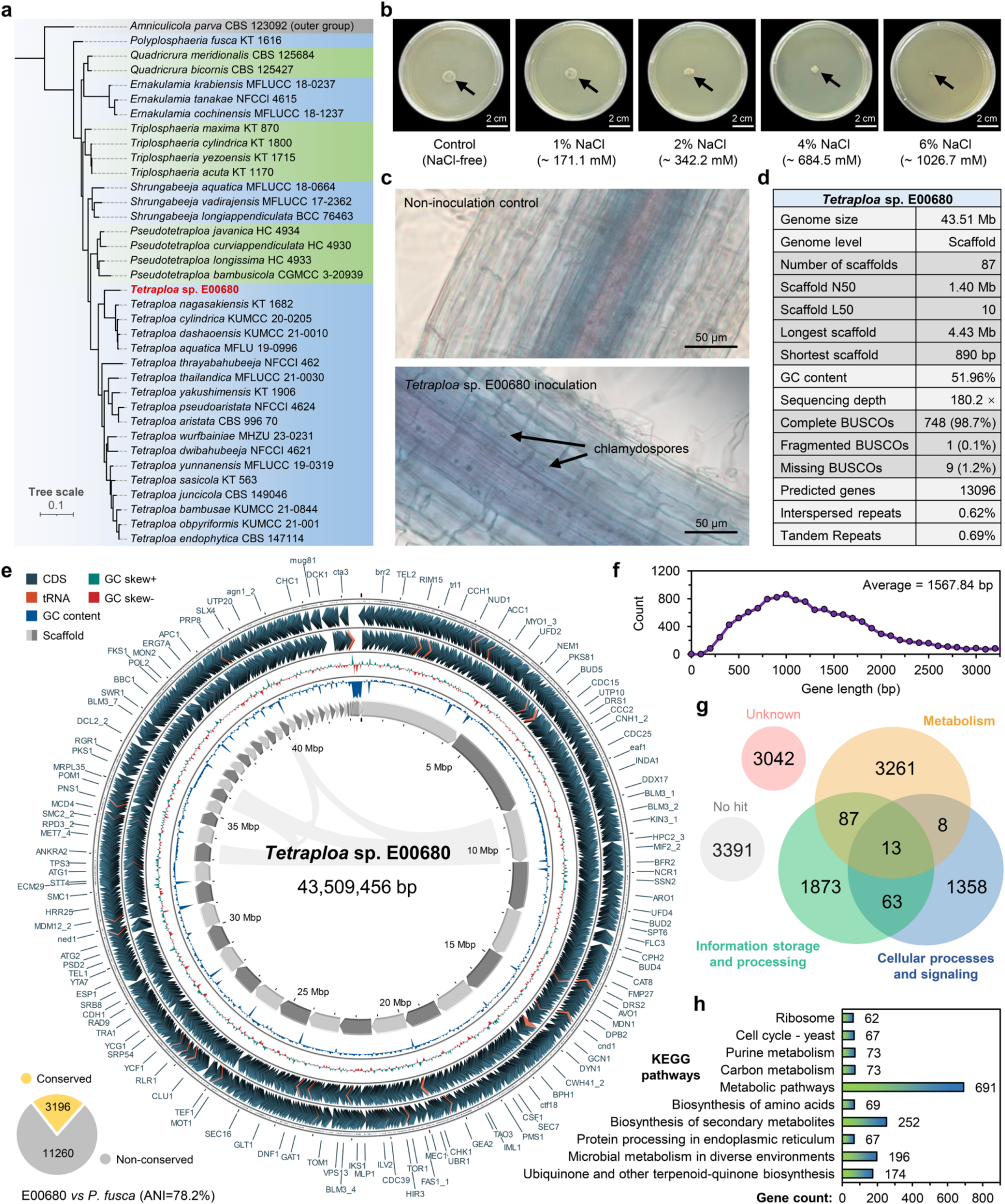
Genomic and phenotypic characterization of a halotolerant fungal strain *Tetraploa* sp. E00680. (a) Maximum‐likelihood phylogenetic tree of *Tetraplosphaeriaceae* based on concatenated sequences of four loci (*LSU*: Large subunit nuclear ribosomal RNA gene; *ITS*: Internal transcribed spacer; *SSU*: Small subunit nuclear ribosomal RNA gene; and *tub2*: β‐tubulin gene). Scale bar indicates substitutions per nucleotide site. (b) Halotolerance assay on potato dextrose agar (PDA) supplemented with NaCl (1%–6% w/v, equivalent to 171.1–1026.7 mM). Arrows indicate viable colonies after 5‐day incubation at 30°C. (c) Intracellular colonization of wheat roots by E00680. Arrows indicate developing chlamydospores within root cells. Images are representative of three independent biological replicates. (d) Genome assembly overview. (e) Circos plot of genome architecture (inner to outer rings): Scaffold lengths; Guanine‐Cytosine (GC) content; GC skew (green: Positive, red: Negative); coding sequences (CDS) and tRNAs on forward/reverse strands. Intra‐genomic syntenic regions and Average Nucleotide Identity (ANI) with *Polyplosphaeria fusca* (GCA_010093805.1) are shown. Gene annotations are available in Table S1. (f) Length distribution of predicted protein‐coding genes. (g) Functional classification by Clusters of Orthologous Groups (COG). (h) Top 10 enriched Kyoto Encyclopedia of Genes and Genomes (KEGG) pathways.

The endophytic fungal strain E00680 was taxonomically identified as *Tetraploa* sp. (Mao et al. 2021; Figure 1a). A NaCl concentration gradient assay revealed its high salt tolerance. Colony growth was not significantly inhibited at 1% (~171.1 mM) NaCl, with growth arrest only occurring at 6% (~1026.7 mM) NaCl (Figure 1b). Trypan blue staining clearly demonstrated the endophytic colonization of *Tetraploa* sp. in wheat roots, showing young chlamydospores within cortical tissues (Figure 1c). Root colonization was further validated via a polymerase chain reaction (PCR) assay utilizing specific primers targeting the E00680 *tub2* gene. Characteristic bands were amplified from DNA extracted from surface‐sterilized roots of E00680‐inoculated plants (Figure S2a,b). Moreover, quantitative PCR (qPCR) analysis established a colonization density of 3597 copies per ng of DNA in the inoculated roots, contrasting with a complete absence of amplification in the uninoculated negative controls (Figure S2c,d). These results strongly support the potential of *Tetraploa* sp. as a PGPM for the mitigation of salt stress in plants.

A hybrid Illumina‐Nanopore sequencing strategy generated a 43.51 Mb reference genome sequence of E00680 (87 scaffolds; GC content 51.96%) with 180.2 × coverage (Figure 1d). The assembly demonstrated high completeness with a BUSCO score of 98.7% using the fungi_odb10 dataset and a low repetitive sequence content of 1.31%. Comparative genomic analysis revealed substantial divergence from *Polyplosphaeria fusca* (average nucleotide identity: 78.2%), indicating evolutionary diversification within *Tetraplosphaeriaceae* (Figure 1e). A total of 13 096 genes were predicted in the genome, with an average gene length of 1568 bp (Figure 1f). Functional annotation categorized all *Tetraploa* sp. genes into three primary Clusters of Orthologous Groups (COG, Figure S3): metabolic processes (3369 genes), information storage/processing (2036 genes), and cellular signalling (1442 genes) (Figure 1g and Table S1). Kyoto Encyclopedia of Genes and Genomes (KEGG) pathway analysis revealed an enrichment in secondary metabolite biosynthesis (map01110) and microbial metabolic pathways (map01120) (Figure 1h and Table S2), which is consistent with the observed salt tolerance and endophytic colonization traits.

Integrated database analyses (PHI, antiSMASH, DFVF, CARD) identified 28 putative host interaction‐related genes, with significant enrichment in plant‐associated functions (42.2% in monocots and 39.5% in eudicots), along with 7 secondary metabolite biosynthetic gene clusters (Tables S3 and S4). While two tubulin genes exhibited sequence similarity to those in vertebrate‐pathogenic fungi (Table S5), the genome lacked critical virulence factors such as hemolysins or antibiotic resistance genes. Tubulin homologues encode highly conserved structural cytoskeletal proteins, which are well recognized as promising targets for antifungal treatments. The absence of inherent toxicity and intact pathogenic pathways suggests that E00680 poses minimal biosafety concerns for humans and the environment.

### 2.2 E00680 Inoculation Enhances Wheat Growth and Physiological Performance Under Salt Stress

**FIGURE 2:**
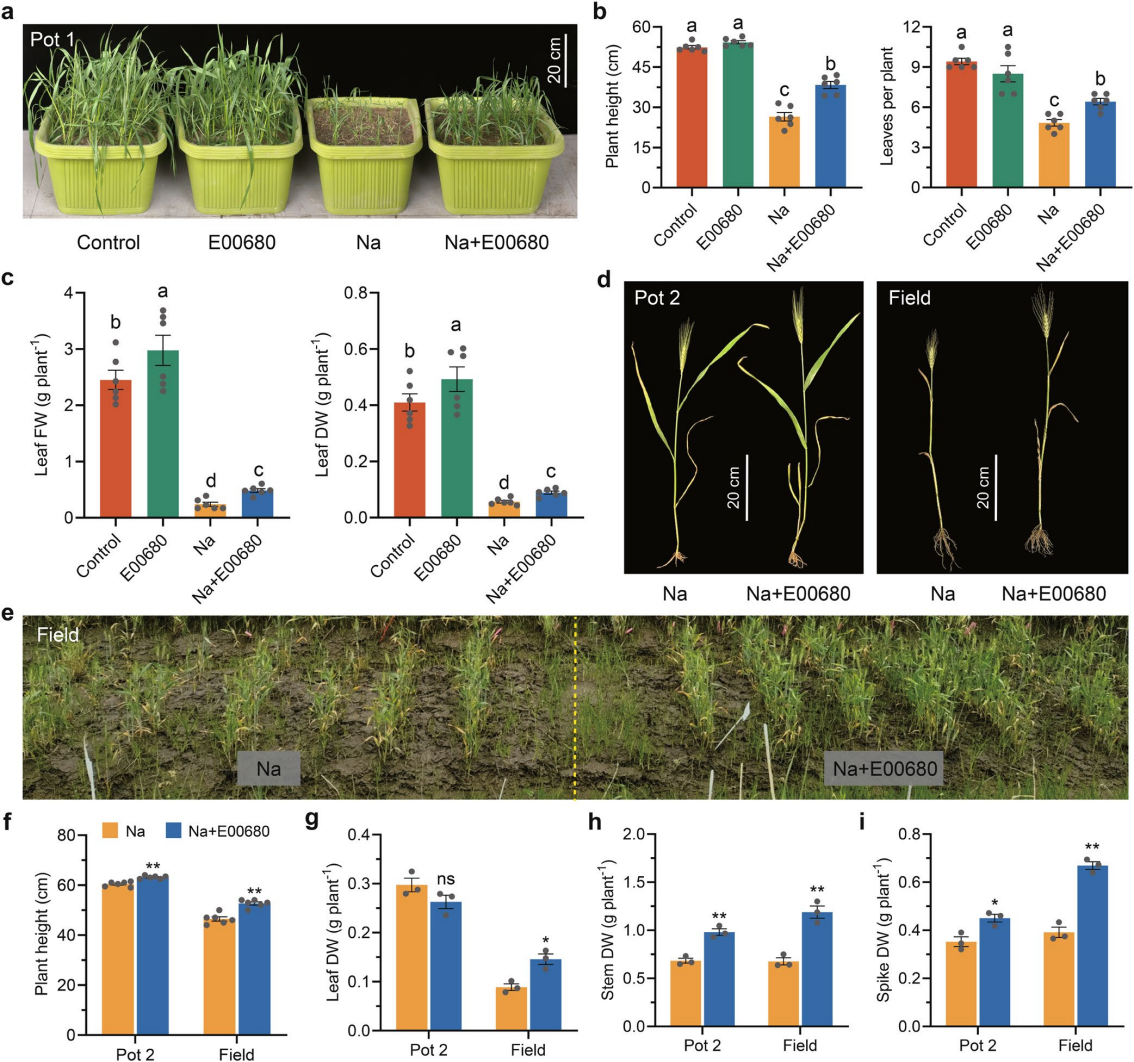
Enhancement of wheat growth by *Tetraploa* sp. E00680 under salt stress. (a–c) Representative plant morphology (a), plant height and leaf count (b), and leaf biomass (c) of wheat plants during the tillering stage in Pot Experiment 1. FW, fresh weight; DW, dry weight; Control, E00680, Na, and Na + E00680 represent wheat plants grown in plain soil, plain soil with E00680 inoculation, plain soil supplemented with 0.3% NaCl (equivalent to 51.3 mM), and plain soil with both 0.3% NaCl and E00680 inoculation, respectively. Data represent mean ± SEM (*n* = 6); lowercase letters indicate statistically significant differences (one‐way ANOVA with Duncan's post hoc test, *p* < 0.05). (d, e) Phenotypic responses of wheat plants in Pot Experiment 2 and the field trial under natural saline‐alkaline soil conditions. (f–i) Plant height (f), leaf DW (g), stem DW (h), and spike DW (i) of wheat plants during the flowering stage in Pot Experiment 2 and the field trial. Na and Na + E00680 represent wheat plants grown in saline‐alkaline soil and saline‐alkaline soil with E00680 inoculation, respectively. Data are shown as mean ± SEM (f, *n* = 6; g–i, *n* = 3); asterisks denote statistical significances between Na + E00680 and Na treatments (two‐tailed Student's *t*‐test: **p < 0.01, *p < 0.05; ns, not significant).

In two independent pot experiments, E00680 inoculation mitigated the growth inhibition caused by 0.3% (~51.3 mM) NaCl and saline‐alkaline stress. At the tillering stage, *Tetraploa* sp.‐inoculated plants exhibited a 100.87% increase in leaf fresh weight and 56.16% higher leaf dry weight relative to salt‐stressed controls (Figure 2a–c). These growth‐promoting effects were consistent at the flowering stage (Figure 2d), with inoculated plants showing 16.32%, 43.15%, and 39.07% greater plant height (cm), stem dry weight (g plant^−1^), and spike dry weight (g plant^−1^), respectively (Figure 2f,h,i). Field trials under natural saline‐alkaline conditions confirmed biomass enhancement (Figure 2d–f), demonstrating 64.01%, 75.58%, and 70.87% increases in leaf, stem, and spike dry weights, respectively, at the flowering stage (Figure 2g–i).

We also found that photosynthetic traits were significantly improved in inoculated plants under salt stress, displaying 28.42% higher SPAD values (relative chlorophyll content measured by a chlorophyll meter developed by the Soil–Plant Analyses Development (SPAD) Section of Minolta Camera; Wu et al. 1998), 35.16% elevated net photosynthetic rate (Pn), 29.73% increased transpiration rate (Tr), and 33.85% enhanced stomatal conductance (Gs) compared to salt‐stressed controls (Figure S4a–d). Oxidative stress analysis revealed that inoculation reduced salt‐induced lipid peroxidation by 68.45%, effectively counteracting the 265.78% malondialdehyde (MDA) accumulation observed in salt‐stressed plants (Figure S4f). This protective effect was correlated with 41.23% greater catalase (CAT) activity (μmol mg^−1^ protein min^−1^) and upregulated antioxidant enzyme profiles (Figure S5).

Element analysis demonstrated improved phosphorus (P) acquisition, with inoculated plants maintaining 24.64% higher leaf P and 23.98% greater root P content under salt stress, compared to the 9.64% P reduction in non‐inoculated salt‐stressed plants (Figure S6). E00680‐mediated ion homeostasis was evidenced by 40.26% lower leaf sodium (Na^+^) accumulation coupled with 52.26% increased leaf potassium (K^+^) content, maintaining favourable leaf Na^+^/K^+^ ratios (Figure S7a–d). Field validation further confirmed these patterns, showing 42.21% reduced leaf Na^+^ and 46.05% elevated leaf K^+^ levels in salt‐treated wheat plants (Figure S7e,f).

### 2.3 E00680 Inoculation Mitigates Salinity‐Induced Yield Loss and Enhances Wheat Productivity

**FIGURE 3:**
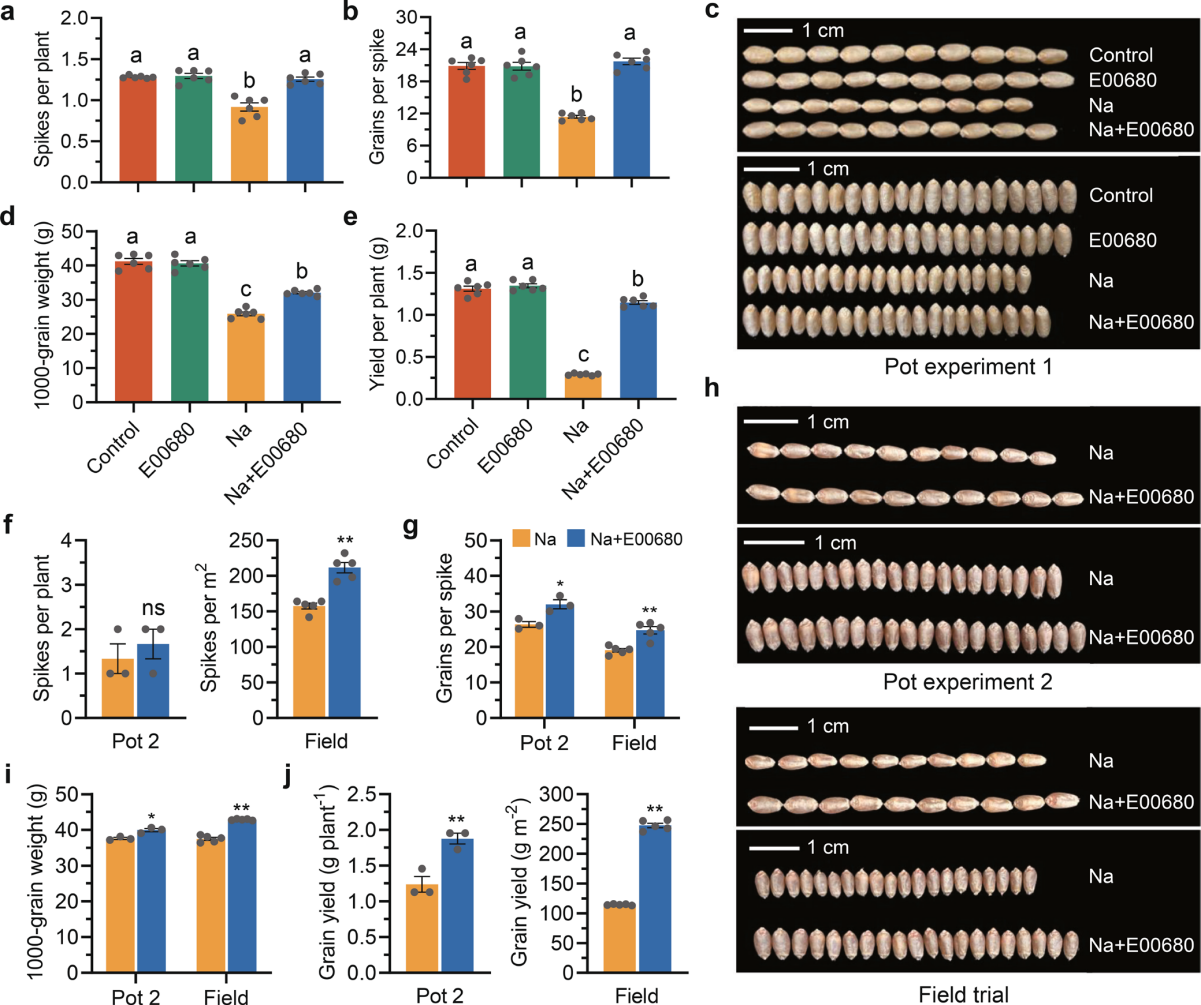
Enhancement of wheat yield by *Tetraploa* sp. E00680 under salt stress. (a–e) Spikes per plant (a), grains per spike (b), grain phenotype (c), 1000‐grain weight (d), and yield per plant (e) of wheat plants in Pot Experiment 1. Control, E00680, Na, and Na + E00680 represent wheat plants grown in plain soil, plain soil with E00680 inoculation, plain soil supplemented with 0.3% NaCl (equivalent to 51.3 mM), and plain soil with both 0.3% NaCl and E00680 inoculation, respectively. Data represent mean ± SEM (*n* = 6); lowercase letters indicate statistically significant differences (one‐way ANOVA with Duncan's post hoc test, *p* < 0.05). (f–j) Spike number (f), grains per spike (g), grain phenotype (h), 1000‐grain weight (i), and grain yield (j) of wheat plants in Pot Experiment 2 and the field trial. Na and Na + E00680 represent wheat plants grown in saline‐alkaline soil and saline‐alkaline soil with E00680 inoculation, respectively. Data represent mean ± SEM (Pot 2, *n* = 3; Field, *n* = 5); asterisks denote significant differences between Na + E00680 and Na treatments (two‐tailed Student's *t*‐test: **p < 0.01, *p < 0.05; ns, not significant).

Grain yield is the ultimate trait for evaluating the efficacy of PGPMs in improving salinity tolerance and agricultural outputs of wheat. In Pot Experiment 1, salt stress caused significant reductions of 21.88% in effective spikes per plant, 41.77% in grains per spike, and 41.13% in 1000‐grain weight compared to non‐stressed plants (Figure 3a,b,d). Grain size was also reduced under salinity stress (Figure 3c), and overall grain yield dropped sharply to 23.29% of the control yield (Figure 3e). However, inoculation with E00680 significantly increased grains per spike, effective spikes per plant, and 1000‐grain weight by 96.83%, 22.72%, and 28.05%, respectively, relative to salt‐stressed plants without E00680 inoculation. These improvements resulted in a significantly higher average yield of 1.1457 g plant^−1^ for inoculated plants, compared to only 0.2901 g plant^−1^ for non‐inoculated plants under salt stress. The substantial increase of 294.92%, calculated as [(1.1457–0.2901)/0.2901 × 100%], can be attributed to the severely reduced growth of non‐inoculated plants under salt stress, which established a low baseline yield and consequently magnified the relative improvement following inoculation.

The benefits of E00680 for wheat yield were even more pronounced in saline‐alkaline field conditions, where inoculated wheat achieved a 2.17‐fold increase in grain yield per square meter compared to the control (Figure 3j). Field trials also showed significant improvements in spikes per square meter and grains per spike by 34.26% and 29.49%, respectively (Figure 3f,g), alongside increases in 1000‐grain weight, grain length, and grain width (Figure 3h,i). Pot Experiment 2 further confirmed the positive effects of E00680 on grain yield under salt stress in wheat (Figure 3f). Taken together, these results highlight the effectiveness of E00680 in enhancing wheat productivity in saline‐alkaline environments by improving key yield‐related traits.

### 2.4 E00680 Inoculation Modifies Soil Properties and Rhizosphere Microbial Composition

Rhizosphere analysis demonstrated that E00680 inoculation increased available nitrogen by 37.22%, phosphorus by 24.80%, and potassium by 57.30% in non‐stressed soil relative to controls (Figure S8a–c). Under salt stress, inoculated soils maintained 47.44%, 18.21%, and 31.30% higher nitrogen, phosphorus, and potassium, respectively, compared to salt‐stressed non‐inoculated controls. The treatment enhanced urease activity by 28.4% and sucrase activity by 22.1%, while alkaline phosphatase increased by 34.26% in control conditions and 39.68% under salt stress (Figure S8d–f), indicating significantly elevated nutrient cycling processes.

**FIGURE 4:**
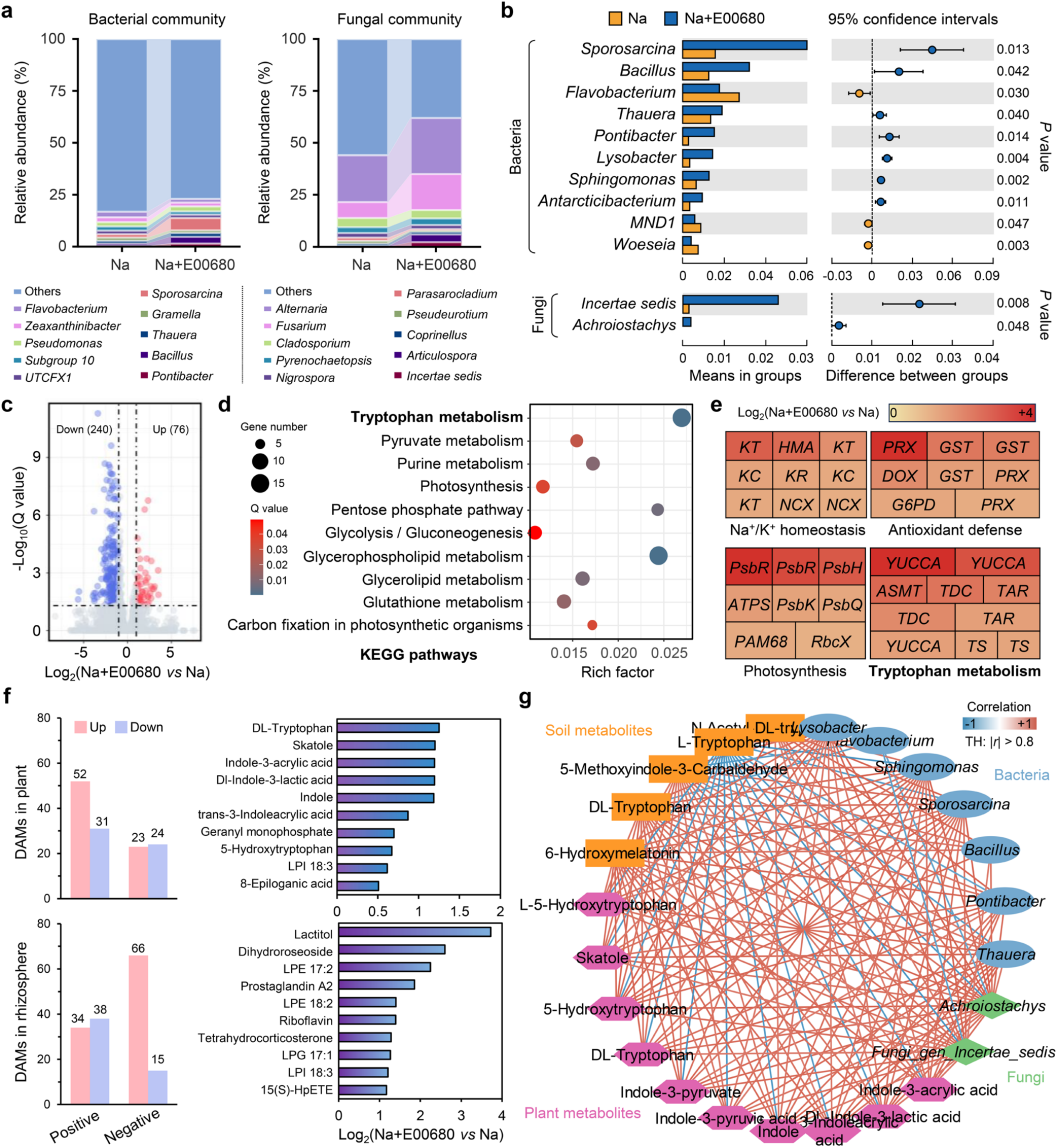
Reprogramming of rhizosphere microbiome, wheat transcriptome, and plant–soil metabolomes by *Tetraploa* sp. E00680 under salt stress. (a) Relative abundance of the top 10 bacterial and fungal genera in the rhizosphere. Na and Na + E00680 represent wheat plants grown in saline‐alkaline soil and saline‐alkaline soil with E00680 inoculation, respectively. (b) Significantly altered microbial taxa in response to E00680 inoculation. Left panels: Mean abundance of selected species; right panels: 95% confidence intervals with *p*‐values (two‐tailed Student's *t*‐test). (c) Differentially expressed genes (DEGs) in wheat leaves. (d) KEGG pathway enrichment analysis of significantly enriched DEGs. (e) Up‐regulated genes associated with salt stress response and core metabolic pathways. Gene identifiers and functional annotations are detailed in Figure S12. (f) Differentially accumulated metabolites (DAMs) in wheat plants and rhizosphere soil. The top 10 up‐regulated metabolites are shown. (g) Correlation network of tryptophan‐related metabolites with rhizosphere microbial genera. Edges represent significant Pearson correlations at a defined threshold (TH) (red: Positive, *r* > 0.8; blue: Negative, *r* < −0.8; *p* < 0.05).

Microbiome profiling revealed E00680‐driven community restructuring, with bacterial communities dominated by *Sporosarcina* (6.0% relative abundance). Inoculation with E00680 increased the proportions of beneficial genera, including *Bacillus*, *Thauera*, and *Sphingomonas*, but reduced the abundance of *Flavobacterium* (Figure 4a,b). Fungal communities exhibited higher abundances of *Fungi_gen_Incertae_sedis* and *Achroiostachys* alongside the dominant *Alternaria* (26.96%) and *Fusarium* (17.23%). It should be noted that *Alternaria* and *Fusarium*, which include many plant‐pathogenic species, are commonly found in soil and were not enriched or recruited through E00680 inoculation. Their abundances showed no significant difference (*p* > 0.05) compared with the non‐inoculated control, whereas only non‐pathogenic taxa were significantly increased upon E00680 treatment (Figure 4b). These compositional shifts in both bacterial and fungal communities were consistent across all samples analysed (Figure S9). Linear discriminant analysis Effect Size (LEfSe) revealed that *Actinobacteriota* and *Bacillaceae* were the key enriched bacterial taxa with *Helotiaceae* representing the key fungal responders (Figure S10), suggesting a strong selective enrichment of salinity‐adapted microbiota in the wheat rhizosphere.

### 2.5 E00680 Triggers Leaf Transcriptomic and Metabolic Reprogramming to Enhance Wheat Salt Tolerance

We then elucidated the molecular mechanisms underlying E00680‐induced salt tolerance in wheat. RNA‐sequencing of field‐grown wheat leaves identified 301 differentially expressed genes (DEGs) between salt‐stressed E00680‐inoculated plants and salt‐stressed controls (Figure 4c). Reverse‐transcription qPCR (RT‐qPCR) validated the reliability of the RNA‐sequencing data (Figure S11). KEGG enrichment analysis revealed that these DEGs were predominantly enriched in pathways associated with photosynthesis and metabolic regulation (Figure 4d). Notably, E00680 inoculation markedly upregulated crucial salt‐responsive genes involved in ion homeostasis (e.g., K^+^ transporters, Na^+^/Ca^2+^ exchangers) and antioxidant defence (e.g., peroxidases, glutathione S‐transferases) (Figure 4e). Comparative analysis further demonstrated distinct expression patterns for metabolism‐related genes across treatments, including 15 and 27 DEGs linked to tryptophan metabolism and lipid metabolism, respectively (Figures 4e and S12).

Moreover, integrated metabolomic profiling of wheat tissues and rhizosphere soil detected 130 plant‐derived and 153 soil‐derived differentially accumulated metabolites (DAMs) (Figures 4f and S13). Among the DAMs, lipid derivatives emerged as the predominant category in both compartments (plants: 22%; soil: 23%), followed by organoheterocyclic compounds (plants: 14%; soil: 11%), as well as phenylpropanoids and polyketides (plants: 8%; soil: 7%). Within these predominant lipid categories, E00680 inoculation significantly upregulated prenol lipids and glycerophospholipids in plants, alongside specific glycerophospholipids in the rhizosphere soil, suggesting potential lipid remodelling in both compartments (Tables S6 and S7). Strikingly, indole‐related metabolites, including *trans*‐3‐indoleacrylic acid, indole, indole‐3‐lactic acid, DL‐tryptophan, and skatole, showed remarkable enrichment in plant tissues, while DL‐tryptophan levels were particularly elevated in rhizosphere soils (Figure 4f, Tables S6 and S7). This metabolic shift exhibited a strong correlation with the DEGs in the tryptophan metabolism pathway identified in transcriptomic profiling. In addition, co‐occurrence network analysis revealed robust interactions between 15 tryptophan‐associated DAMs and 9 microbial taxa, forming 108 correlations (99 positive/9 negative) (Figure 4g), suggesting a strong microbial‐metabolite interplay in modulating tryptophan metabolism under salt stress.

### 2.6 A Proposed Model for E00680‐Mediated Salt Tolerance Through Cross‐Kingdom Tryptophan‐Auxin Coordination

**FIGURE 5:**
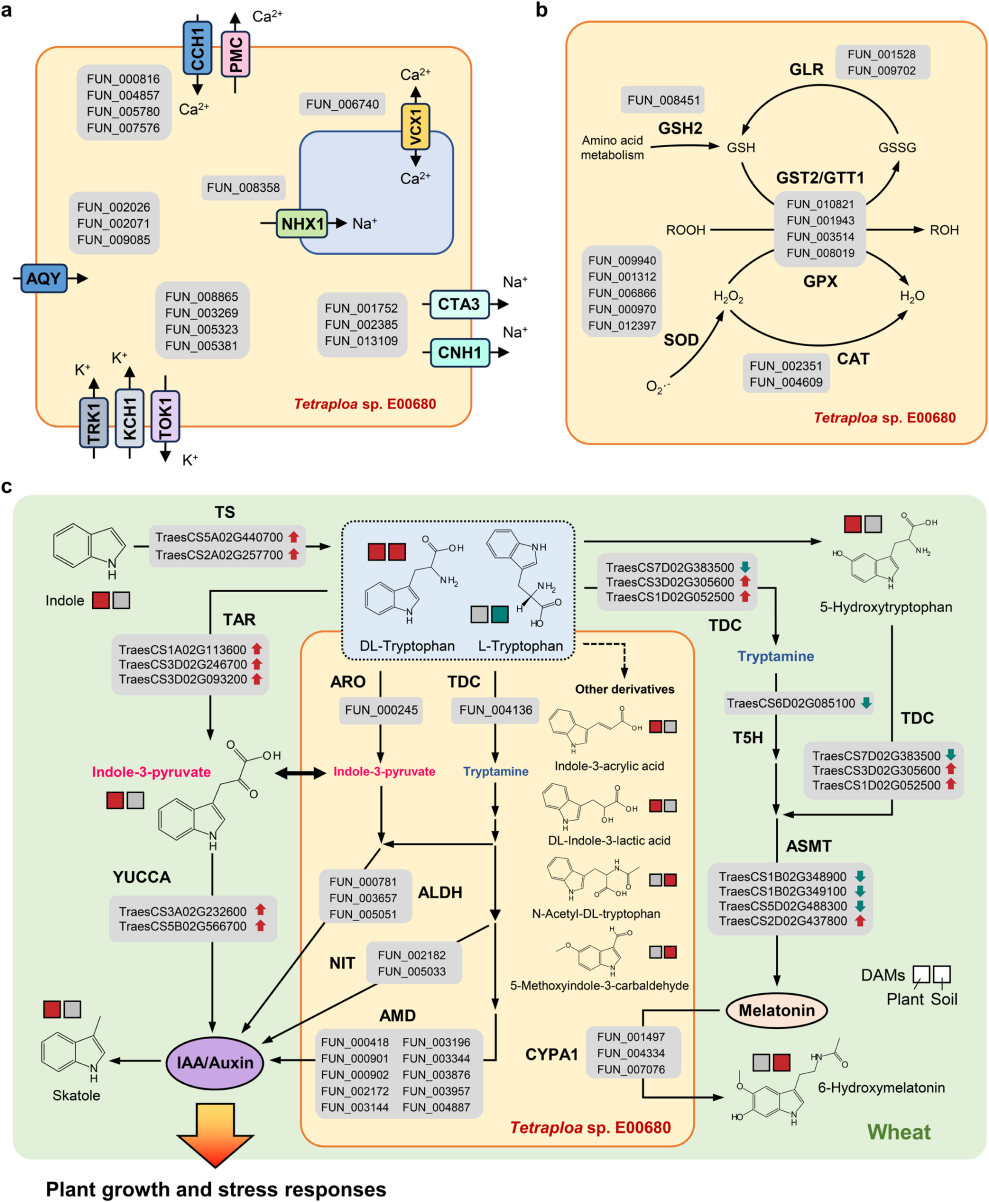
Integrated multi‐omics analysis reveals the mechanism of *Tetraploa* sp. E00680‐mediated salt stress tolerance in wheat. (a, b) Cellular detoxification and salt tolerance mechanisms in E00680, driven by ion (a) and redox (b) homeostasis. Genes encoding transporters and enzymes are highlighted in grey rounded rectangles. Functional annotations: AQY, aquaporin; TRK1, low‐affinity potassium transporter; KCH1, potassium transporter; TOK1, potassium channel; CCH1, calcium channel protein; PMC, plasma membrane calcium ATPase; VCX1, vacuolar calcium ion transporter; CNH1, Na^+^/H^+^ antiporter; NHX1, Na^+^/H^+^ antiporter; CTA3, potassium/sodium efflux transporter; GSH2, glutathione synthetase; GLR, glutathione reductase; GPX, glutathione peroxidase; GST2, glutathione S‐transferase 2; GTT1, bifunctional glutathione transferase/peroxidase. Metabolites: GSH, reduced glutathione; GSSG, oxidized glutathione; ROOH, organic hydroperoxide; ROH, alcohol. (c) Endophytic colonization‐driven cross‐kingdom metabolism enhances salt tolerance in wheat. Differentially accumulated metabolites (DAMs) in wheat (left) and rhizosphere soil (right) are indicated in boxes (red: Up‐regulated; green: Down‐regulated; grey: Unchanged or not detected). Solid lines represent one‐step reactions; dashed lines denote multi‐step processes. Genes from wheat and E00680 encoding pathway enzymes are shown in grey rounded rectangles. Differential gene expression in wheat is marked by arrows (red: Up‐regulated; green: Down‐regulated). Key enzymes: TS, tryptophan synthase; TAR, tryptophan aminotransferase‐related; YUCCA, indole‐3‐pyruvate monooxygenase; ARO, aromatic aminotransferase; ALDH, aldehyde dehydrogenase; NIT, nitrilase; AMD, amidase; T5H, tryptamine 5‐hydroxylase; TDC, tryptophan decarboxylase; ASMT, N‐acetylserotonin methyltransferase; CYPA1, cytochrome P450 family 1 subfamily A1.

Furthermore, the genomic assembly of the halotolerant fungal *Tetraploa* sp. strain E00680 revealed intrinsic salt adaptation mechanisms involving ion homeostasis and oxidative stress mitigation. For instance, the *Tetraploa* sp. genome harbours key salt‐tolerance‐associated genes in the canonical sodium exclusion systems (*CNH1*, *CTA3*, *NHX1*), potassium regulatory components (*TRK1*, *KCH1*, *TOK1*), and calcium signalling modules (*CCH1*, *PMC*, *VCX1*) (Figure 5a and Table S8). Transcriptomic analysis of the strain under salt stress supported these genomic findings, with 779 up‐regulated and 641 down‐regulated genes identified. Notably, the sodium efflux genes *CNH1* and *CTA3*, as well as potassium uptake genes such as *TRK1* and *KCH1*, were up‐regulated, alongside the up‐regulated expression of two aquaporin (*AQY*) genes (Figure S14). Multiple genes associated with antioxidant defence were identified, including those in glutathione metabolic pathway elements (*GSH2*, *GLR*, *GPX*, *GST2*, *GTT1*) and redox enzymes (*SOD*, *CAT*), suggesting the significant potential of E00680 for detoxifying reactive oxygen species (ROS) under saline stress at the genomic level (Figure 5b and Table S8). Correspondingly, the transcriptomic data also revealed the significant up‐regulation of multiple genes associated with oxidative stress responses (Figure S14). Moreover, sequence alignment of 45 identified salt‐tolerance genes against the well‐known halotolerant fungal PGPM *Piriformospora indica* revealed low homology (with over half of the gene identities below 40%), highlighting a distant phylogenetic relationship and suggesting substantially divergent mechanisms of salt adaptation (Table S9).

Complementing these genomic insights, multi‐omics analyses demonstrated that E00680 colonization induces systemic metabolic reprogramming in the wheat host. Transcriptomic profiling of wheat roots revealed salinity‐dependent activation of tryptophan metabolism pathways, with coordinated up‐regulation of key auxin biosynthesis genes including *tryptophan synthase* (*TS*), *tryptophan aminotransferase* (*TAR*), and *indole‐3‐pyruvate monooxygenase* (*YUCCA*) (Figure 5c and Table S5). Notably, the E00680 fungal genome also encodes proteins in the complementary auxin synthesis machinery [aldehyde dehydrogenase (ALDH), nitrilase (NIT), and amidase (AMD)], suggesting a cross‐kingdom metabolic collaboration with the transcriptome‐metabolome networks in wheat (Figure 5c). Targeted metabolomics confirmed this synergy through the coordinated accumulation of tryptophan derivatives and indole compounds in both plant tissues and rhizosphere soil, establishing a novel plant‐fungal metabolic loop for auxin biosynthesis under salt stress (Figure 5c).

**FIGURE 6:**
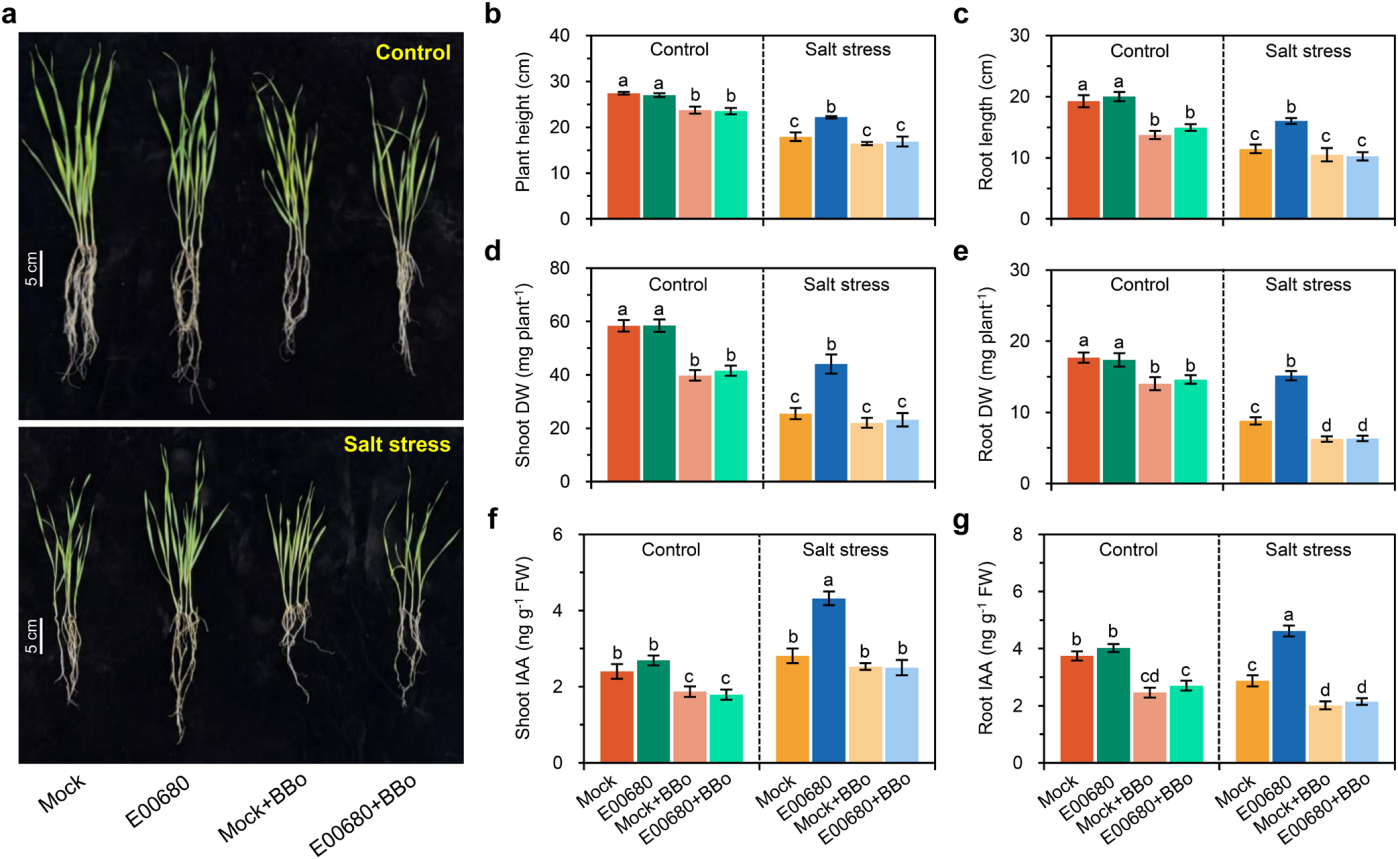
Roles of *Tetraploa* sp. E00680 in auxin production for salt stress tolerance in wheat. (a) Representative plant morphology. Hydroponically grown wheat seedlings were subjected to two primary conditions: Control (BNS) and salt stress (BNS supplemented with 100 mM NaCl). Each condition included four sub‐treatments: Mock (non‐inoculated), E00680 (inoculated with 100 mL microbial inoculum), Mock + BBo (non‐inoculated, supplemented with 3 μM BBo), and E00680 + BBo (inoculated with 100 mL microbial inoculum and 3 μM BBo). BNS, basic nutrient solution; BBo, 4‐biphenylboronic acid (an auxin biosynthesis inhibitor). (b‐g) Plant height (b), root length (c), shoot DW (d), root DW (e), shoot IAA content (f), and root IAA content (g) of wheat seedlings across the different treatments. DW, dry weight; IAA, indole‐3‐acetic acid. Data represent mean ± SEM (*n* = 4); lowercase letters indicate statistically significant differences (one‐way ANOVA with Duncan's post hoc test, *p* < 0.05).

To functionally validate the contribution of this plant‐fungal tryptophan‐auxin pathway to the beneficial phenotypes conferred by E00680, we applied exogenous auxin biosynthesis inhibitors to wheat. Hydroponic experiments were conducted to eliminate potential confounding effects from the soil environment. Under control conditions, no significant phenotypic differences were observed between mock‐ and E00680‐inoculated plants (Figure 6a). The application of 4‐biphenylboronic acid (BBo) significantly reduced indole‐3‐acetic acid (IAA) content, subsequently decreasing plant height, root length, and overall biomass across all treatments (Figure 6b–g). This confirmed the efficacy of BBo in inhibiting auxin biosynthesis and suppressing plant growth. Under salt stress, E00680 inoculation significantly enhanced IAA content in shoots and roots by 53.88% and 60.72%, respectively. It also improved overall plant performance compared to the mock control, leading to increases in plant height, root length, and shoot/root dry weight (Figure 6b–e). However, BBo treatment under salt stress completely abolished these beneficial effects; E00680‐inoculated plants exhibited low IAA content and showed no significant improvement over mock‐inoculated controls across these growth parameters (Figure 6b–g). An independent hydroponic experiment using an alternative inhibitor, 4‐phenoxyphenylboronic acid (PPBo), yielded consistent results (Figure S15). Together, these functional assays demonstrate that the beneficial effects of E00680 on wheat salt tolerance are fundamentally dependent on an intact auxin biosynthesis pathway.

**FIGURE 7:**
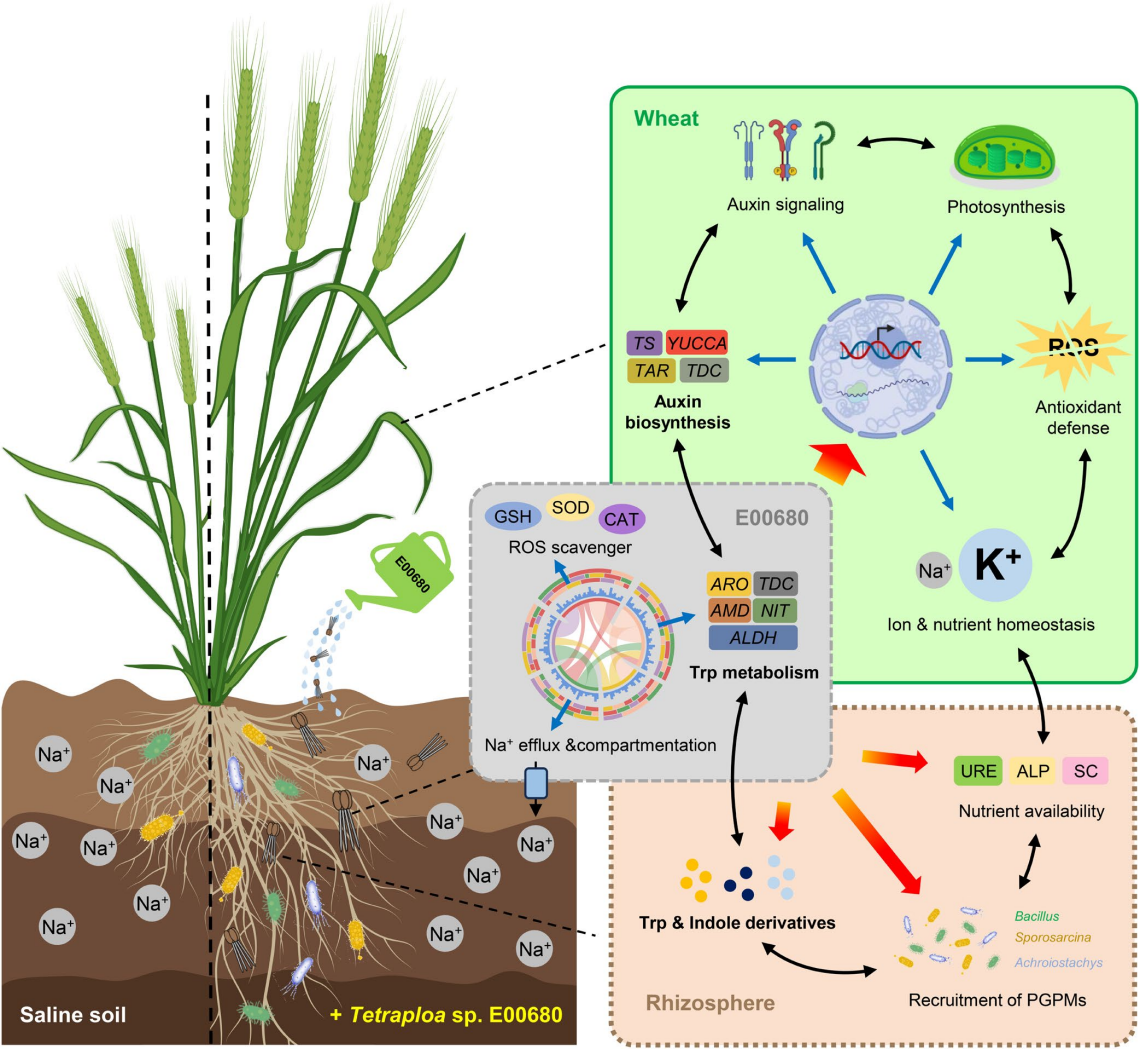
Proposed working model of *Tetraploa* sp. E00680‐mediated salt stress resilience and yield improvement in wheat. Inoculation with the endophytic fungus E00680 enhances plant growth and grain yield under saline stress by improving photosynthetic efficiency, potassium (K^+^) retention, and nitrogen/phosphorus acquisition, while reducing sodium (Na^+^) accumulation and oxidative damage through suppression of reactive oxygen species (ROS). These synergistic effects are driven by multi‐layered plant–microbe–soil interactions, including recruitment of plant growth‐promoting microorganisms (PGPMs) that enhance rhizosphere nutrient availability via urease (URE), alkaline phosphatase (ALP), and sucrase (SC) activities. Concurrently, E00680 induces transcriptional reprogramming in wheat, upregulating genes associated with ion homeostasis, antioxidant defence, photosynthesis, and metabolic pathway modulation. A key regulatory axis involves cross‐kingdom tryptophan (Trp) coordination: Host Trp metabolism through tryptophan synthase (TS), tryptophan aminotransferase‐related protein (TAR), and indole‐3‐pyruvate monooxygenase (YUCCA) drives auxin biosynthesis, while fungal Trp processing via tryptophan decarboxylase (TDC), aldehyde dehydrogenase (ALDH), and amidase (AMD) amplifies auxin production. This coordinated auxin signalling promotes plant growth and systemic salt stress adaptation.

In summary, our integrative analysis supports a dual‐mode salt tolerance mechanism in the *Tetraploa* sp. E00680‐wheat interaction: (1) cross‐compartment auxin signalling via integration of fungal and plant tryptophan metabolic pathways (Figure S16), and (2) systemic stress mitigation through enhanced antioxidant system, ionic Na^+^/K^+^ equilibrium maintenance, photosynthetic optimization, as well as microbial community restructuring and nutrient mobilization (Figure 7). This multilayered adaptation strategy provides a novel framework for understanding microbe‐mediated crop resilience to saline soils for future research endeavours.

## 3 Discussion

Endophytic microorganisms play crucial roles in enhancing plant growth and improving environmental stress resilience, particularly under extreme climatic and environmental conditions (Shu and Huang 2022). Among fungi, previous studies have highlighted the salinity‐ameliorating effects of the desert‐adapted *P. indica* in barley (Baltruschat et al. 2008) and the stress‐mitigating properties of *Fusarium* sp. from the halophytic rice Pokkali (Sampangi‐Ramaiah et al. 2020). Here, we characterized the halotolerant endophytic fungus *Tetraploa* sp. E00680, which was isolated from the salt‐ and heat‐tolerant C4 weed *Eleusine indica* (goosegrass; Mao et al. 2021). As both wheat and goosegrass belong to the grass family Poaceae, their close phylogenetic relationship enables them to share fungal endophytic PGPMs. The E00680 strain exhibits exceptional osmotic adaptability, thriving in media containing up to 6% NaCl, nearly twice the salinity of seawater (6% vs. 3.5%), and maintaining effective root colonization in wheat (Figure 1b,c). Both field and pot experiments demonstrated its significant mitigation of salt‐induced grain yield loss in wheat (Figures 2 and 3). Furthermore, our high‐quality genome assembly of *Tetraploa* sp. E00680 provides the first comprehensive genomic reference for *Tetraploa* species, facilitating comparative genomic and evolutionary analyses within the family Tetraplosphaeriaceae (Figure 1a,d,e). These findings position *Tetraploa* species as promising PGPM candidates in the development of saline‐adaptive biofertilizers for staple crops.

Salt stress poses dual physiological challenges by causing excessive Na^+^ accumulation and ROS overproduction (Van Zelm et al. 2020). Although core cation transporters have been functionally characterized in yeast models (Ariño et al. 2019), their regulatory dynamics in endophyte‐plant systems remain poorly understood. Genomic and transcriptomic analyses of E00680 revealed multiple salt stress‐responsive components, including plasma membrane and tonoplast Na^+^/H^+^ antiporters and K^+^ transporters (Figures 5a and S14), which facilitate the exclusion and vacuolar compartmentalization of Na^+^ (Ramakrishna et al. 2025). Additionally, E00680 inoculation significantly improved the host Na^+^/K^+^ homeostasis under salt stress by promoting K^+^ retention in the roots and limiting Na^+^ translocation to the shoots (Figure S7). This balance is driven by the transcriptomic regulation of multiple wheat K^+^ transporter genes, including *TraesCS2A02G107100*, *TraesCS6D02G275100*, and *TraesCS2B02G123700* (Figures 4e and S12), which is consistent with previous reports that microbial partners modulate the expression of host ion transporters to enhance salinity tolerance (Abdelaziz et al. 2017; Wang, Li, et al. 2024). Furthermore, E00680 inoculation mitigated oxidative damage by stabilizing chlorophyll and optimizing gas exchange to preserve photosynthetic efficiency (Figure S4). It also activated both fungal‐derived ROS‐scavenging pathways and the host plant's endogenous antioxidants (Figures 5b and S5). These coordinated responses reflect common PGPM‐mediated adaptation strategies observed in species such as wheat, tomato, and cabbage (Lastochkina et al. 2017; Feng et al. 2024; Peng et al. 2026).

Tryptophan metabolism serves as a critical nexus in plant stress adaptation, and exogenous tryptophan supplementation has been shown to alleviate salinity stress (Iqbal and Ashraf 2007). As a principal tryptophan derivative and fundamental phytohormone, IAA orchestrates numerous functions in plant development, symbiotic interactions, and stress tolerance (Etesami and Glick 2024). Core IAA biosynthetic components, including the *Tryptophan Aminotransferase of Arabidopsis* (*TAA*)/*YUCCA* gene families, have been implicated in drought and salinity tolerance (Im Kim et al. 2013; Ribba et al. 2020). This metabolic network also extends into microbial domains, with phylogenetically diverse taxa such as *Bacillus*, *Azospirillum*, and *Pseudomonas* species employing conserved enzymatic machinery for tryptophan conversion and IAA production (Patten et al. 2013). Microbial metabolites like indole‐3‐lactic acid and indole‐3‐carboxaldehyde also function in cross‐kingdom signalling (Hilbert et al. 2012; Lu et al. 2024). Our integrated multi‐omics approach reveals that E00680‐mediated salt tolerance relies on the sophisticated, cross‐kingdom regulation of these tryptophan metabolic networks (Figures 4, 5, 6). Angiosperms predominantly utilize the indole‐3‐pyruvic acid (IPyA) pathway for IAA biosynthesis, whereas microbial systems demonstrate metabolic plasticity through parallel routes including the tryptamine (TAM), indole‐3‐acetamide (IAM), and indole‐3‐acetonitrile (IAN) pathways (Cassán et al. 2014; Xu et al. 2023). Notably, E00680 inoculation enhances the plant's endogenous IAA production through precursor supplementation and IPyA pathway activation, which is coupled with microbial coordination of the TAM/IAM/IAN pathways to amplify auxin biosynthesis (Figures 5c and 6). Increased IAA levels enhance root architecture by promoting lateral root initiation and root hair development, thereby expanding the root surface area for better water and nutrient uptake under saline conditions (Figure 6). Additionally, elevated IAA strengthens stress‐responsive signalling, improving osmotic adjustment and ROS scavenging capacity (Rawat et al. 2025). This synergistic metabolic crosstalk generates an auxin‐enriched rhizosphere microenvironment that sustains wheat growth and yield under saline stress.

PGPMs exert multifunctional impacts, encompassing both rhizosphere modification and microbial community restructuring (Philippot et al. 2024; Zhang et al. 2026). In this study, E00680 inoculation significantly elevated alkaline phosphatase, sucrase, and urease activities in the rhizosphere of salt‐stressed wheat (Figures S6–S8). These enzymatic boosts improved nutrient bioavailability—particularly the mineralization and acquisition of organic phosphorus—consistent with known PGPM‐mediated nutrient mobilization mechanisms (Jansson et al. 2023; Liu, Bai, et al. 2024). Furthermore, salt‐induced phospholipid remodelling led to the accumulation of signalling molecules (Table S7), a process previously linked to stress signalling, symbiosis initiation, and cellular homeostasis during prolonged salinity (Okazaki and Saito 2014). Beyond biochemical modifications, E00680 profoundly restructured the rhizobiome (Figure 4a,b), selectively enriching functionally relevant taxa such as halotolerant *Bacillus* (Wu et al. 2024) and phosphate‐mobilizing *Sporosarcina* (Achal et al. 2012). This microbial reprogramming aligns with known PGPM strategies where stress‐adapted consortia enhance plant resilience. For instance, recovery from cadmium stress has been linked to the recruitment of *Gemmatimonas* and *Sphingomonas* (Zhang et al. 2023), and similar microbiome shifts occur in arbuscular mycorrhizal fungal (AMF)‐mediated salinity tolerance (Guo et al. 2024). These results establish E00680 as an effective model microbial inoculant that bolsters wheat salt tolerance by dual‐modulating rhizosphere biochemistry and assembling a beneficial microbiota. Although E00680 did not enrich potential fungal pathogens, future risk assessments remain necessary to evaluate its long‐term ecological impacts on pathogen dynamics under field conditions. Given the emerging strategy of applying rationally designed synthetic communities (SynComs) to improve plant fitness (Xu et al. 2025), future research should explore constructing tailored consortia that combine E00680, AMF, *P. indica* and other beneficial microbes for synergistic stress mitigation.

In conclusion, we have elucidated the genomic architecture of the halophytic fungal strain *Tetraploa* sp. E00680 and its salt adaptation mechanisms in the rhizosphere of wheat. We demonstrated that cross‐kingdom tryptophan metabolism in E00680‐inoculated wheat confers significant growth and yield advantages under saline stress, resulting in up to a 2.17‐fold yield increase in field trials. The host wheat genotype employed in this study, Yangmai 20, is a widely cultivated local variety and serves as a regional trial control, making it an appropriate system for validating the efficacy of E00680. For practical application, the ecologically sustainable E00680 strain can be formulated as a scalable solid or liquid bioinoculant using suitable carriers. Moving forward, these insights support the biotechnological development and field‐scale deployment of E00680‐based strategies, contributing to climate‐resilient and sustainable crop production in salt‐affected soils worldwide.

## 4 Experimental Procedures

### 4.1 Plant Materials and Microbial Inoculum

The wheat cultivar Yangmai 20, a major commercial variety in the study region, was used to evaluate the effects of *Tetraploa* sp. strain E00680 inoculation under saline soil conditions. The holotype and ex‐type culture of E00680 were deposited in the Chinese General Microbiological Culture Collection Center (https://www.cgmcc.net/english/; CGMCC 23278). The fungal strain was cultured in potato dextrose broth (PDB) according to the inoculum preparation protocol described in our previous study (Zhang et al. 2023). The resulting fungal suspension had a concentration of 1.05–2.15 × 10^6^ CFU L^−1^.

### 4.2 Physiological Characterization of E00680

Salt tolerance capacity was evaluated through growth assays on potato dextrose agar (PDA) supplemented with incremental NaCl concentrations: 1% (~171.1 mM), 2% (~342.2 mM), 4% (~684.5 mM), and 6% (~1026.7 mM), with a NaCl‐free control. Triplicate cultures for each concentration were incubated at 30°C for 5 days, with colony growth recorded and analysed.

For assessing endophytic properties, wheat seedlings were harvested, and microbial distribution in the roots was examined using the trypan blue staining method (Michal Johnson et al. 2011), with observations under a Carl Zeiss microscope (Jena, Germany). To further validate root colonization at the molecular level, E00680‐inoculated plants and non‐inoculated control plants were harvested from a hydroponic culture 7 days post‐inoculation. Total DNA was extracted from surface‐sterilized roots and shoots using the cetyltrimethylammonium bromide (CTAB) method. Conventional PCR was performed using wheat‐specific *β‐tubulin* primers alongside E00680 *tub2*‐specific primers (Table S10). For quantitative analysis, a standard curve was generated using serial dilutions (1 × 10^7^ to 10^2^ copies μL^−1^) of a pMD18‐T vector containing the *tub2* gene. The total DNA concentration of the biological samples was standardized to 20 ng μL^−1^. Quantitative PCR was then conducted using a LightCycler 480 system (Roche Diagnostics, Mannheim, Germany) with a FAM‐labelled TaqMan probe, and the exact copy number of E00680 was quantified based on the resulting Ct values against the standard curve.

### 4.3 Whole Genome Sequencing, Assembly, and Annotation

The complete genome of E00680 was sequenced using a hybrid approach combining both short‐read and long‐read sequencing technologies. Short‐read sequencing was conducted on the Illumina NovaSeq 6000 platform (Illumina, San Diego, CA, USA) to generate 2 × 150 bp paired‐end reads, utilizing the NEBNext Ultra DNA Library Prep Kit (New England Biolabs, Ipswich, MA, USA) for library preparation. For long‐read sequencing, high‐molecular‐weight DNA libraries were prepared using the SQK‐LSK109 kit and sequenced on the PromethION platform with R9.4.1 flow cells (Oxford Nanopore Technologies, Oxford, UK). The long‐read assembly was initially performed with Flye (https://github.com/fenderglass/Flye) and subsequently polished using the Illumina short reads. Polishing was carried out by aligning the short reads with minimap2 (https://github.com/lh3/minimap2) and performing error correction with Pilon (https://github.com/broadinstitute/pilon). The quality of the assembly was assessed using BUSCO completeness analysis (https://github.com/metashot/busco). For annotation, the pipeline included repeat element detection with RepeatMasker (https://github.com/Dfam‐consortium/RepeatMasker) and Tandem Repeats Finder (https://github.com/Benson‐Genomics‐Lab/TRF), gene structure prediction using the Funannotate workflow (https://github.com/nextgenusfs/funannotate), and functional characterization through the COG and KEGG databases. Secondary metabolic gene clusters were predicted using antiSMASH (https://fungismash.secondarymetabolites.org/). Virulence genes were identified using BLASTP against databases such as PHI (http://www.phi‐base.org/), DFVF (http://sysbio.unl.edu/DFVF/), and CARD (https://card.mcmaster.ca/), applying a similarity threshold of > 90%. Comparative genomic analyses were performed, including synteny analysis with MCScanX (https://github.com/wyp1125/MCScanX) and genomic similarity assessment using FastANI (https://github.com/ParBLiSS/FastANI) to calculate the average nucleotide identity.

### 4.4 Phylogenetic Analyses

Phylogenetic analyses were conducted in PhyloSuite (http://phylosuite.jushengwu.com/) using a concatenated dataset consisting of the large subunit ribosomal RNA (*LSU*), internal transcribed spacer (*ITS*), small subunit ribosomal RNA (*SSU*), and β‐tubulin (*tub2*) sequences from the *Tetraplosphaeriaceae* family (Table S11). Gene sequences were aligned with MAFFT (using the E‐INS‐i algorithm), trimmed using TrimAl in ‘gappyout’ mode, and then concatenated. Evolutionary relationships were reconstructed using both maximum likelihood (ML) and Bayesian inference (BI). ML analysis was performed using IQ‐TREE, employing an edge‐linked partition model with 10 000 ultrafast bootstrap replicates. BI was implemented in MrBayes using partitioned models over 2 × 10^6^ generations with a 25% burn‐in. Optimal substitution models were selected using ModelFinder based on the Bayesian Information Criterion (BIC). The consensus trees generated by both methods were visualized using the Interactive Tree of Life (iTOL, https://itol.embl.de/) platform. To investigate the homology of the identified salt‐tolerance genes, protein sequence alignments were performed via BLASTP against the well‐known halotolerant fungus *P. indica* (syn. *Serendipita indica*), using the reference genome of *S. indica* DSM 11827.

### 4.5 Pot Experiments

Two pot experiments were conducted in a rainout shelter at Zhejiang University (Hangzhou, China) during the winter seasons of 2022 and 2023. In 2022, surface soil (0–15 cm depth) collected from the campus experimental farm was characterized by a pH of 7.3, total NaCl content of 0.03%, total K of 0.67%, available N of 59.5 mg kg^−1^, and available P of 48.3 mg kg^−1^. In 2023, soil was sourced from a newly reclaimed saline‐alkaline area in Ningbo, China (30.30° N, 121.15° E), with a pH of 8.37, total NaCl content of 0.25% (EC1:5–6.4 mS cm^−1^), total N of 0.085%, available P of 20.8 mg kg^−1^, and available K of 165 mg kg^−1^.

Pot Experiment 1 comprised four treatments: control (plain soil), E00680 (soil inoculated with E00680), Na (soil + 0.3% NaCl), and Na + E00680 (soil + 0.3% NaCl + E00680 inoculation). Pot Experiment 2 featured two treatments: Na (saline‐alkaline soil) and Na + E00680 (saline‐alkaline soil + E00680 inoculation). Both experiments used a randomized block design with four replicates per treatment. Each pot (63 cm × 36 cm × 25 cm) contained 35 kg of air‐dried soil, sown with 70 seeds and later thinned to 16 plants. E00680 inoculation was performed just before sowing by mixing the microbial inoculum with the soil at a final ratio of 1:10 (v/w), resulting in an inoculation intensity of approximately 1.05–2.15 × 10^5^ CFU kg^−1^ soil. A nutrient solution, prepared according to our previous study (Qiu et al. 2019), was applied at 1 L per pot during the tillering stage and 2 L from the jointing stage to maturity.

### 4.6 Field Experiment

The field experiment was conducted on newly reclaimed saline‐alkaline soil in the coastal area of Ningbo, China (30.30° N, 121.15° E) during the winter of 2022. The loamy sand soil had the following properties: a pH of 8.35, total NaCl content of 0.25% (EC1:5–6.4 mS cm^−1^), total *N* of 0.088%, available P of 20.2 mg kg^−1^, and available K of 173 mg kg^−1^. Two treatments were applied: (1) Na, wheat seeds sown in non‐inoculated soil; (2) Na + E00680, wheat seeds sown in soil inoculated with E00680. Inoculation was applied at a rate of 3 L of microbial inoculum (containing 1.05–2.15 × 10^6^ CFU L^−1^) per plot during the tillering stage, a strategy chosen to emulate practical agricultural practices. The experiment used a randomized block design with eight replicates per treatment. Each plot was 2.5 m × 1 m and contained 10 rows spaced 0.25 m apart, with approximately 70 seeds per row. Agronomical practices including irrigation and fertilizer application followed local best management practices.

### 4.7 Determination of Growth, Agronomic, and Yield Traits

Plant samples were collected at the tillering and flowering stages from each treatment. Fresh weights of the different plant parts were recorded. For dry weight determination, samples were subjected to enzyme deactivation at 105°C for 30 min, and then dried at 70°C to a constant weight. At maturity, yield parameters were recorded, including spike number, grains per spike, grain size, 1000‐grain weight, and overall grain yield.

### 4.8 Gas Exchange Measurements

Leaf chlorophyll content was measured using a Minolta SPAD‐502 chlorophyll meter (Minolta Corporation Ltd., Osaka, Japan). Net photosynthetic rate, stomatal conductance, transpiration rate, and intercellular CO_2_ concentration of the second fully expanded leaf were measured on sunny mornings between 9:00 and 11:00 AM using a LI‐6400XT portable photosynthesis system (LI‐COR Inc., Lincoln, NE, USA) as described previously (Wang et al. 2023).

### 4.9 Determination of Lipid Peroxidation and Antioxidant Enzyme Activities

Fresh tissue sample (0.3 g) from the second fully expanded leaf was homogenized in 3 mL of 50 mM phosphate‐buffered saline (PBS, pH 7.8) on ice. The homogenate was centrifuged at 7500× *g* for 15 min at 4°C. The supernatant was used to measure the activities of ascorbate peroxidase (APX, EC 1.11.1.11), peroxidase (POD, EC 1.11.1.7), catalase (CAT, EC 1.11.1.6), and superoxide dismutase (SOD, EC 1.15.1.1), as well as MDA content, as described previously (Qiu et al. 2021).

### 4.10 Plant Element Content

Elemental analysis was performed as described previously (Qiu et al. 2021). Approximately 0.2 g of dry powdered sample was digested in 3 mL of 70% nitric acid using a constant‐temperature metal bath (DTU‐2CN, TAITEC, Saitama, Japan). After dilution, the total Na, K, Ca, and P contents were quantified using an iCAP RQ inductively coupled plasma mass spectrometer (Thermo Scientific, Bremen, Germany).

### 4.11 Soil Mineral Content and Enzyme Activities

Rhizosphere soil samples were collected during the wheat tillering stage. Following the methods described by Potdar et al. (2021), soil available N was determined using the alkaline permanganate method, the available P was extracted with sodium bicarbonate and analysed through the molybdenum blue colorimetric method, and the available K was extracted with ammonium acetate and quantified using flame photometry. The activities of sucrase (EC 3.2.1.26), urease (EC 3.5.1.5), and alkaline phosphatase (EC 3.1.3.1) were evaluated using commercial assay kits (Nanjing Jiancheng Bioengineering Institute, Nanjing, China) following the manufacturer's protocols.

### 4.12 Microbial Community Analysis

Rhizosphere soil samples from the field experiment were collected for microbial community analysis. Total DNA extraction, library preparation for 16S rRNA and fungal internal transcribed spacer (ITS) amplicons, and raw data processing were conducted as described previously (Zhang et al. 2023). Sequencing was performed on an Illumina NovaSeq 6000 platform (Illumina, San Diego, CA, USA). Effective tags were clustered into operational taxonomic units (OTUs) at a 97% similarity threshold using UPARSE. Taxonomic annotation for bacterial OTUs utilized the Silva database (https://www.arb‐silva.de/), while fungal OTUs were annotated using the Unite database (https://unite.ut.ee/). The linear discriminant analysis effect size (LEfSe) algorithm, with a linear discriminant analysis (LDA) score > 4.0 and a *p*‐value < 0.05, was used to identify representative bacterial and fungal taxa.

### 4.13 RNA‐Sequencing and Transcriptome Analysis

Wheat leaf samples were collected during the flowering stage of the field experiment. Fungal samples were collected after 24 h of 1% NaCl treatment (~171.1 mM; a NaCl‐free PDB culture served as the control). Total RNA from the wheat and E00680 samples was extracted using the TRIzol reagent. High‐throughput RNA‐sequencing and data analysis were conducted according to our previous study (Qiu et al. 2023). Raw data were generated by an Illumina HiSeq2500 system (Illumina, San Diego, CA, USA). After quality control, clean reads were aligned to the wheat genome and the E00680 genome using HISAT (https://daehwankimlab.github.io/hisat2/) and Bowtie2 (https://bowtie‐bio.sourceforge.net/bowtie2/). Gene expression levels were calculated with RSEM (https://github.com/deweylab/RSEM). Differentially expressed genes (DEGs) were identified using DESeq2 (https://github.com/thelovelab/DESeq2), which models read count data based on a negative binomial distribution. To rigorously control for false positives, DEGs were defined using an absolute log_2_(Fold Change) ≥ 1 and a Benjamini‐Hochberg false discovery rate (FDR)‐adjusted *Q*‐value ≤ 0.05. To validate RNA sequencing accuracy, 13 DEGs associated with plant salt tolerance were selected for RT‐qPCR analysis. Relative expression levels were quantified using the comparative 2^−ΔΔCt^ method with β‐tubulin as the reference gene, as detailed previously (Qiu et al. 2019). Primer sequences are provided in Table S10.

### 4.14 Plant and Rhizosphere Soil Metabolome Analysis

Leaf and rhizosphere soil samples were collected during the flowering stage of the field experiment. Metabolite extraction and liquid chromatography–tandem mass spectrometry (LC–MS/MS) analysis were conducted according to our previous study (Qiu et al. 2023). Metabolites were annotated using the KEGG, HMDB, and LIPIDMaps databases. Data were processed with the metaX software (https://github.com/wenbostar/metaX), and principal component analysis (PCA) and partial least squares discriminant analysis (PLS‐DA) were performed. Variable importance in projection (VIP) values for PC1 in PLS‐DA were calculated, and *p*‐values were obtained through *t*‐tests. Differential metabolites were identified based on VIP > 1, *p*‐value < 0.05, and fold change (FC) > 1.2 or FC < 0.8333.

### 4.15 Co‐Occurrence Network Analysis of Metabolites and the Microbiome

To investigate the interplay between tryptophan metabolism and the microbial community, a co‐occurrence network was constructed using the relative abundances of specific metabolites and microbes. The analysis included differentially abundant bacterial (mean abundance > 0.01) and fungal genera, alongside key DAMs linked to plant or soil tryptophan metabolism. Network edges were determined using Pearson correlation coefficients, with statistical thresholds for strong correlations set at |*r*| > 0.8 and a significance level of *p* < 0.05 (Wu et al. 2023). To strictly isolate cross‐domain interactions, only significant inter‐category relationships (e.g., plant–microbe, microbe–soil, and plant–soil) were retained in the network; all intra‐category interactions were explicitly excluded (Baker et al. 2024). The final correlation matrix was visualized using the Cytoscape software.

### 4.16 Hydroponic Experiments

Hydroponic experiments were conducted in a growth house at Zhejiang University (Hangzhou, China) to validate the functional role of the auxin biosynthesis pathway. Sterilized wheat seeds were germinated on a moist sand base and incubated at 20°C. Uniform, vigorous, one‐leaf stage (seven‐day‐old) seedlings were transplanted into 1 L plastic containers containing a modified Hoagland's solution as the basic nutrient solution (BNS). The growth house conditions were maintained at 22°C/18°C (day/night), with 50% to 70% relative humidity, and a 14 h/10 h light/dark photoperiod. Three days post‐transplantation, the seedlings were subjected to two main treatments: a control (BNS alone) and salt stress (BNS + 100 mM NaCl). Each treatment group comprised four sub‐treatments: Mock (non‐inoculated), E00680 (inoculated, supplemented with 100 mL of microbial inoculum), Mock + BBo (non‐inoculated, supplemented with 3 μM of the auxin biosynthesis inhibitor BBo [Kakei et al. 2015]), and E00680 + BBo (inoculated, supplemented with 100 mL microbial inoculum and 3 μM of BBo). Seven days post‐treatment, wheat plants from the different groups were harvested to determine growth parameters and endogenous IAA content. To ensure the reliability of the results, an independent hydroponic experiment was repeated under salt stress conditions using an alternative inhibitor PPBo (Kakei et al. 2015), with an identical experimental setup.

### 4.17 Determination of Endogenous IAA Content

Endogenous IAA content was determined following the methodology described by Li et al. (2026). For each sample, 10 shoots or roots were pooled, and four biological replicates were prepared per treatment. Endogenous plant hormones were extracted from the homogenized samples using an acetonitrile solution. Internal standards were incorporated into the extraction buffer to calibrate the subsequent detection results. The endogenous plant hormone IAA was then quantified using a Qsight LX 50 ultra‐high‐performance liquid chromatography system coupled to a Qsight 420 triple quadrupole mass spectrometer (UPLC‐MS/MS; PerkinElmer, Waltham, MA, USA).

### 4.18 Statistical Analysis

The statistical analysis was performed using SPSS software. A two‐sided Student's *t*‐test was utilized to compare two groups, while one‐way analysis of variance (ANOVA) followed by Duncan's post hoc test was used for multiple comparisons.

## Author Contributions

**Cheng‐Wei Qiu:** formal analysis, validation, visualization, writing – original draft, writing – review and editing; **Shuo Zhang:** investigation; **Zi‐Feng Gao:** investigation, formal analysis, data curation; **Chulong Zhang:** resources; **Zhong‐Hua Chen:** formal analysis, writing – review and editing. **Mohamed Abdelalim Ali:** writing – review and editing. **Feibo Wu:** conceptualization, methodology, resources, formal analysis, writing – review and editing, supervision, project administration, funding acquisition.

## Funding

This work was supported by National Natural Science Foundation of China (32161143035), the 111 Project of China (BP0618021), Australian Research Council (FT210100366), Grains Research and Development Corporation (WSU2303‐001RTX).

## Conflicts of Interest

The authors declare no conflicts of interest.

## Supporting information


**Figure S1:** Experimental workflow for investigating *Tetraploa* sp. E00680‐mediated salt stress resilience in wheat. The study integrated functional characterization, genomic profiling, agronomic validation, and multi‐omics mechanistic analysis. Functional assays began with evaluating *Tetraploa* sp. E00680's salt tolerance and endophytic colonization capacity, followed by Illumina and Nanopore sequencing to generate the first complete genome assembly for this species. Two independent pot experiments and a field trial under saline‐alkaline conditions were then conducted to quantify E00680's effects on stress mitigation and grain yield enhancement. Mechanistic insights were derived from integrated multi‐omics analyses, including functional genomics of E00680, rhizosphere microbiome profiling, host transcriptomics, and plant–soil metabolomics, collectively decoding the tripartite plant‐microbe‐soil interactions driving crop resilience.
**Figure S2:** Molecular detection of endophytic colonization by *Tetraploa* sp. E00680 in wheat. (a) Details of biological samples used for PCR analysis. (b) PCR products amplified from the indicated biological samples using wheat‐specific *β‐tubulin* primers and E00680‐specific *tub2* primers, analysed on a 1% agarose gel. (c) Standard curve for absolute quantification of E00680, generated by quantitative PCR (qPCR) using serial dilutions of a pMD18‐T plasmid containing the *tub2* gene. (d) Quantification of E00680‐specific *tub2* copy numbers in DNA samples extracted from surface‐sterilized roots of E00680‐inoculated plants, as determined by qPCR.
**Figure S3:** Functional annotation of *Tetraploa* sp. E00680 protein‐coding genes based on Clusters of Orthologous Groups (COG) classification.
**Figure S4:** Effects of *Tetraploa* sp. E00680 inoculation on physiological traits and malondialdehyde (MDA) content in wheat under salt stress. SPAD value (a), net photosynthetic rate (Pn, b), transpiration rate (Tr, c), stomatal conductance (Gs, d), intercellular CO_2_ concentration (Ci, e), and malondialdehyde content (f) of wheat plants during the tillering stage in Pot Experiment 1. Control, E00680, Na, and Na + E00680 represent wheat plants grown in plain soil, plain soil with E00680 inoculation, plain soil supplemented with 0.3% NaCl, and plain soil with both 0.3% NaCl and E00680 inoculation, respectively. Data represent mean ± SEM (*n* = 6); lowercase letters indicate statistically significant differences (one‐way ANOVA with Duncan's post hoc test, *p* < 0.05).
**Figure S5:** Effects of *Tetraploa* sp. E00680 inoculation on antioxidant enzyme activities in wheat under salt stress. Activities of ascorbate peroxidase (APX, a), peroxidase (POD, b), catalase (CAT, c), and superoxide dismutase (SOD, d) of wheat plants during the tillering stage in Pot Experiment 1. Control, E00680, Na, and Na + E00680 represent wheat plants grown in plain soil, plain soil with E00680 inoculation, plain soil supplemented with 0.3% NaCl, and plain soil with both 0.3% NaCl and E00680 inoculation, respectively. Data represent mean ± SEM (*n* = 3); lowercase letters indicate statistically significant differences (one‐way ANOVA with Duncan's post hoc test, *p* < 0.05).
**Figure S6:** Effects of *Tetraploa* sp. E00680 inoculation on nutrient uptake in wheat under salt stress. Calcium (Ca) and phosphorus (P) content in the leaf (a, b) and root (c, d) of wheat plants during the tillering stage under salt stress in Pot Experiment 1. Control, E00680, Na, and Na + E00680 represent wheat plants grown in plain soil, plain soil with E00680 inoculation, plain soil supplemented with 0.3% NaCl, and plain soil with both 0.3% NaCl and E00680 inoculation, respectively. Data represent mean ± SEM (*n* = 4); lowercase letters indicate statistically significant differences (one‐way ANOVA with Duncan's post hoc test, *p* < 0.05).
**Figure S7:** Effects of *Tetraploa* sp. E00680 inoculation on Na and K distribution in wheat plants under salt stress. (a–d) Na and K content in the leaves and roots of wheat plants during the tillering stage in Pot Experiment 1. Control, E00680, Na, and Na + E00680 represent wheat plants grown in plain soil, plain soil with E00680 inoculation, plain soil supplemented with 0.3% NaCl, and plain soil with both 0.3% NaCl and E00680 inoculation, respectively. Data represent mean ± SEM (*n* = 4); lowercase letters indicate statistically significant differences (one‐way ANOVA with Duncan's post hoc test, *p* < 0.05). (e, f) Na and K content in the leaves and roots of wheat plants during the flowering stage in the field experiment. Na and Na + E00680 represent wheat plants grown in saline‐alkaline soil and saline‐alkaline soil with E00680 inoculation, respectively. Data represent mean ± SEM (*n* = 4); asterisks denote significant differences between Na + E00680 and Na treatments (two‐tailed Student's *t*‐test: **p < 0.01, *p < 0.05; ns, not significant).
**Figure S8:** Effects of *Tetraploa* sp. E00680 inoculation on soil elemental contents and biochemical properties. Available nitrogen (N, a), phosphorus (P, b), potassium (K, c), urease activity (d), alkaline phosphatase activity (e), and sucrase activity (f) in rhizosphere soil of wheat plants during the tillering stage under salt stress in Pot Experiment 1. Control, E00680, Na, and Na + E00680 represent wheat plants grown in plain soil, plain soil with E00680 inoculation, plain soil supplemented with 0.3% NaCl, and plain soil with both 0.3% NaCl and E00680 inoculation, respectively. Data represent mean ± SEM (*n* = 3); lowercase letters indicate statistically significant differences (one‐way ANOVA with Duncan's post hoc test, *p* < 0.05).
**Figure S9:** Principal component analysis (PCA) of bacterial (16S rRNA) and fungal (internal transcribed spacer, ITS) microbiomes. Na and Na + E00680 represent wheat plants grown in saline‐alkaline soil and saline‐alkaline soil with E00680 inoculation, respectively.
**Figure S10:** Differential rhizosphere microbiota profiling through Linear discriminant analysis Effect Size (LEfSe) analysis under salt stress with E00680 inoculation. Phylogenetic signatures showing statistically significant (*p* < 0.05) enrichment of bacterial (a) and fungal (b) taxa in E00680‐treated versus control groups. Biomarker taxa with Linear discriminant analysis (LDA) scores exceeding the threshold (> 4.0) are indicated in red, while olive‐shaded nodes represent microbial features without significant intergroup differences.
**Figure S11:** Experimental validation of transcriptomic data through reverse‐transcription quantitative PCR (RT‐qPCR) analysis. (a) Comparison of expression levels for 13 selected differentially expressed genes (DEGs) between RNA sequencing (RNA‐seq) and RT‐qPCR assays. (b) Correlation analysis of gene expression patterns between RNA‐seq and RT‐qPCR. ** indicates significant Spearman correlation (*p* < 0.01).
**Figure S12:** Functional categorization of salt stress‐responsive genes modulated by E00680 inoculation. Comparative transcriptome heatmaps display differential gene expression patterns between two experimental groups. The log_2_(Na + E00680 vs. Na) ratio was used to assess gene expression changes. Genes were categorised as up‐regulated (log2 ratio > 1, *Q*‐value < 0.05) or down‐regulated (log2 ratio < −1, *Q*‐value < 0.05). Na and Na + E00680 represent wheat plants grown in saline‐alkaline soil and saline‐alkaline soil with E00680 inoculation, respectively.
**Figure S13:** Principal component analysis (PCA) for leaf and soil metabolomes. (a, b) Plant samples in positive and negative ionization modes. (c, d) Soil samples in positive and negative ionization modes. Na and Na + E00680 represent wheat plants grown in saline‐alkaline soil and saline‐alkaline soil with E00680 inoculation, respectively. QC, quality control.
**Figure S14:** Transcriptome analysis of E00680 in response to salt stress. Differentially expressed genes (DEGs) are visualized in a volcano plot. Comparative transcriptome heatmaps display differential gene expression patterns between the two experimental groups. The log_2_(Na vs. control) ratio was used to assess gene expression changes. Genes were categorized as up‐regulated (log2 ratio > 1, *Q*‐value < 0.05) or down‐regulated (log2 ratio < −1, *Q*‐value < 0.05). The control and NaCl treatment groups represent E00680 cultured in an NaCl‐free potato dextrose broth (PDB) medium and treated with 1% NaCl (~171.1 mM) for 24 h, respectively.
**Figure S15:** Effect of an alternative auxin biosynthesis inhibitor on Tetraploa sp. E00680‐mediated salt stress tolerance in wheat. (a) Representative plant morphology. Hydroponically grown wheat seedlings under salt stress (BNS supplemented with 100 mM NaCl) were subjected to four treatments: Mock (non‐inoculated), E00680 (inoculated with 100 mL microbial inoculum), Mock + PPBo (non‐inoculated, supplemented with 3 μM PPBo), and E00680 + PPBo (inoculated with 100 mL microbial inoculum and 3 μM PPBo). BNS, basic nutrient solution; PPBo, 4‐phenoxyphenylboronic acid. (b) Plant growth parameters of wheat seedlings across the different treatments. DW, dry weight. Data represent mean ± SEM (*n* = 4); lowercase letters indicate statistically significant differences (one‐way ANOVA with Duncan's post hoc test, *p* < 0.05).
**Figure S16:** A schematic diagram summarizing multi‐omics interactions. Auxin production mediated by E000680 serves as a central hub connecting genomic, transcriptomic, metabolomic, and microbiome data, conferring salt stress tolerance in wheat. IAA, indole‐3‐acetic acid; Trp, tryptophan.

**Table S1:** COG‐based functional annotation of predicted genes in E00680.
**Table S2:** KEGG‐based functional annotation of predicted genes in E00680.
**Table S3:** Predicted pathogen‐host interaction genes in E00680.
**Table S4:** Predicted secondary metabolite biosynthetic gene clusters in E00680.
**Table S5:** Predicted virulence‐associated genes in E00680.
**Table S6:** Up‐regulated metabolites in wheat plants after E00680 inoculation under salt stress.
**Table S7:** Up‐regulated metabolites in rhizosphere soil after E00680 inoculation under salt stress.
**Table S8:** Key genes related to E00680‐mediated salt stress tolerance.
**Table S9:** BLAST‐based comparison of key genes associated with salt tolerance in E00680 and *Serendipita indica*.
**Table S10:** Primer sequences used for PCR and qPCR validation.
**Table S11:** GenBank accession numbers for phylogenetic analyses.

## Data Availability

The authors confirm that the data supporting the findings of this study are available within the article. Genome sequence data of E00680 have been deposited in the National Microbiology Data Center (https://nmdc.cn/en; NMDC) with the accession number NMDC60214233. Further raw data are available from the corresponding author, upon reasonable request.
