# Supplementary material for: First Tetraploa Genome and Multi‐Omics Analysis Reveal Key Plant‐Microbe‐Soil Interactions for Salt Tolerance and Yield Improvement of Wheat
Source: Plant Biotechnol J. 2026 Apr 3;24(8):4748–65. doi: 10.1111/pbi.70663 (PMC13387892; doi:10.1111/pbi.70663)
Supplement: Supplementary file 1 — Figure S1: Experimental workflow for investigating Tetraploa sp. E00680‐mediated salt stress resilience in wheat. The study integrated functional characterization, genomic profiling, agronomic validation, and multi‐omics mechanistic analysis. Functional assays began with evaluating Tetraploa sp. E00680's salt tolerance and endophytic colonization capacity, followed by Illumina and Nanopore sequencing to generate the first complete genome assembly for this species. Two independent pot experiments and a field trial under saline‐alkaline conditions were then conducted to quantify E00680's effects on stress mitigation and grain yield enhancement. Mechanistic insights were derived from integrated multi‐omics analyses, including functional genomics of E00680, rhizosphere microbiome profiling, host transcriptomics, and plant–soil metabolomics, collectively decoding the tripartite plant‐microbe‐soil interactions driving crop resilience. Figure S2: Molecular detection of endophytic colonization by Tetraploa sp. E00680 in wheat. (a) Details of biological samples used for PCR analysis. (b) PCR products amplified from the indicated biological samples using wheat‐specific β‐tubulin primers and E00680‐specific tub2 primers, analysed on a 1% agarose gel. (c) Standard curve for absolute quantification of E00680, generated by quantitative PCR (qPCR) using serial dilutions of a pMD18‐T plasmid containing the tub2 gene. (d) Quantification of E00680‐specific tub2 copy numbers in DNA samples extracted from surface‐sterilized roots of E00680‐inoculated plants, as determined by qPCR. Figure S3: Functional annotation of Tetraploa sp. E00680 protein‐coding genes based on Clusters of Orthologous Groups (COG) classification. Figure S4: Effects of Tetraploa sp. E00680 inoculation on physiological traits and malondialdehyde (MDA) content in wheat under salt stress. SPAD value (a), net photosynthetic rate (Pn, b), transpiration rate (Tr, c), stomatal conductance (Gs, d), intercellular CO2 concen [file PBI-24-4748-s001.pdf]

Figure S1

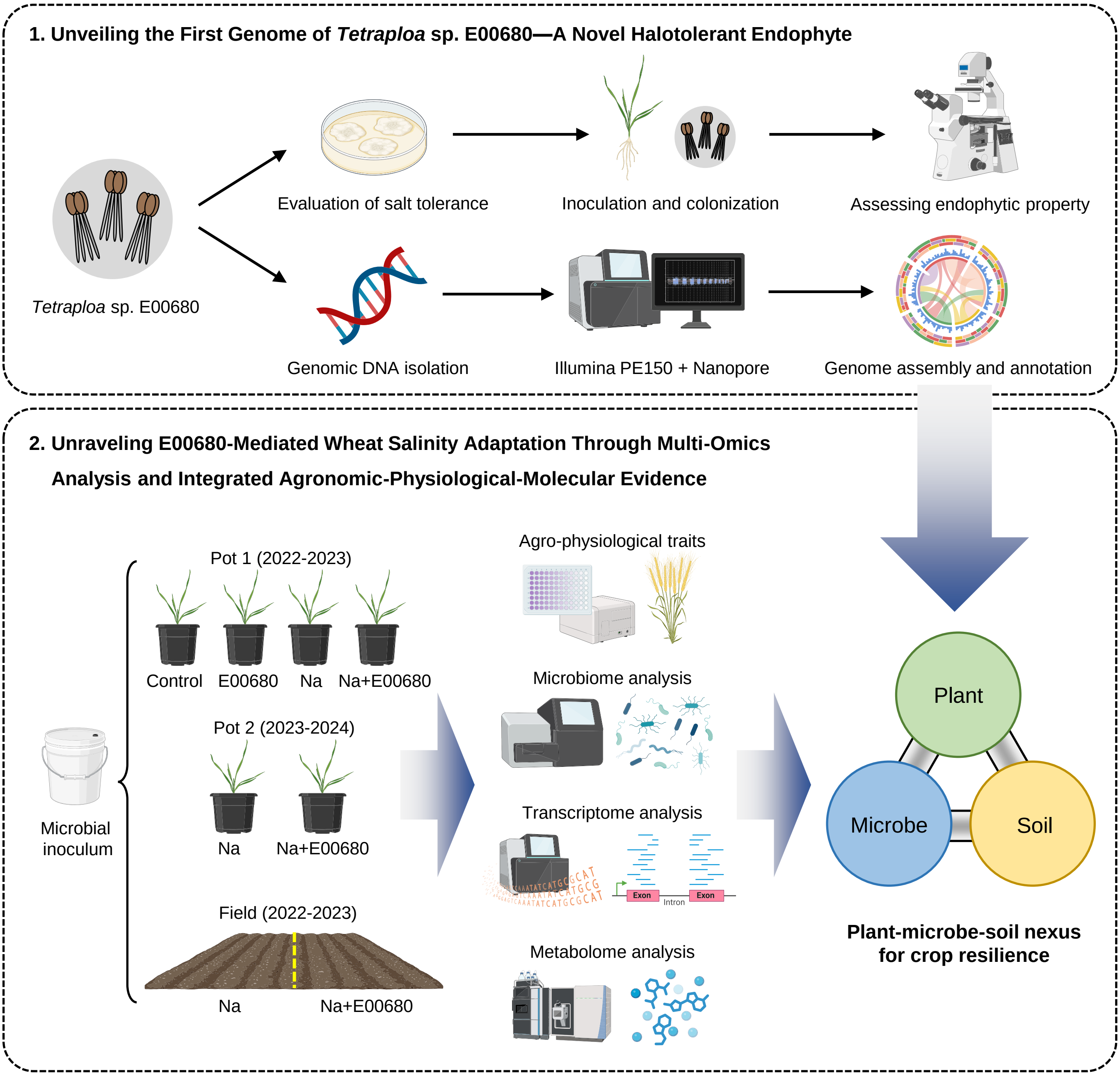

Figure S1 Experimental workflow for investigating *Tetraploa* sp. E00680-mediated salt stress resilience in wheat. The study integrated functional characterization, genomic profiling, agronomic validation, and multi-omics mechanistic analysis. Functional assays began with evaluating *Tetraploa* sp. E00680's salt tolerance and endophytic colonization capacity, followed by Illumina and Nanopore sequencing to generate the first complete genome assembly for this species. Two independent pot experiments and a field trial under saline-alkaline conditions were then conducted to quantify E00680's effects on stress mitigation and grain yield enhancement. Mechanistic insights were derived from integrated multi-omics analyses, including functional genomics of E00680, rhizosphere microbiome profiling, host transcriptomics, and plant-soil metabolomics, collectively decoding the tripartite plant-microbe-soil interactions driving crop resilience.

Figure S2

a

Label	Description
M	2000 bp DNA marker
1	DNA from non-inoculated roots*
2	Genomic DNA of E00680
3	DNA from E00680-inoculated roots*
4	DNA from the shoots of E00680-inoculated plants
5	Genomic DNA of E00680
6	DNA from the shoots of E00680-inoculated plants

* Root samples are thoroughly washed and surface-sterilized.

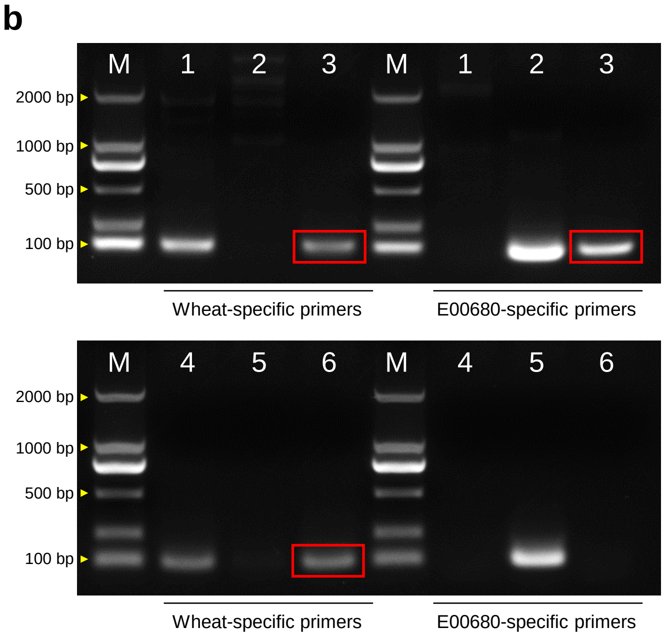

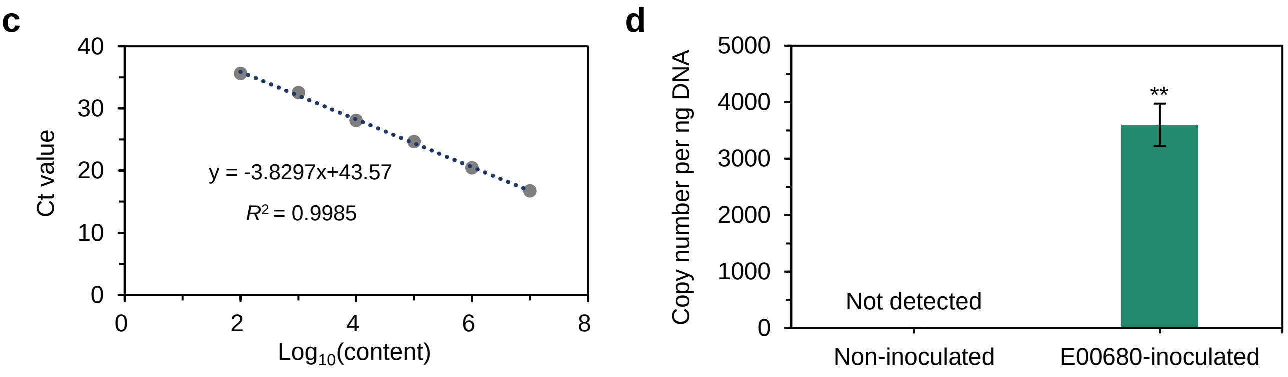

Figure S2 Molecular detection of endophytic colonization by *Tetraploa* sp. E00680 in wheat. (a) Details of biological samples used for PCR analysis. (b) PCR products amplified from the indicated biological samples using wheat-specific *β-tubulin* primers and E00680-specific *tub2* primers, analyzed on a 1% agarose gel. (c) Standard curve for absolute quantification of E00680, generated by quantitative PCR (qPCR) using serial dilutions of a pMD18-T plasmid containing the *tub2* gene. (d) Quantification of E00680-specific *tub2* copy numbers in DNA samples extracted from surface-sterilized roots of E00680-inoculated plants, as determined by qPCR.

Figure S3

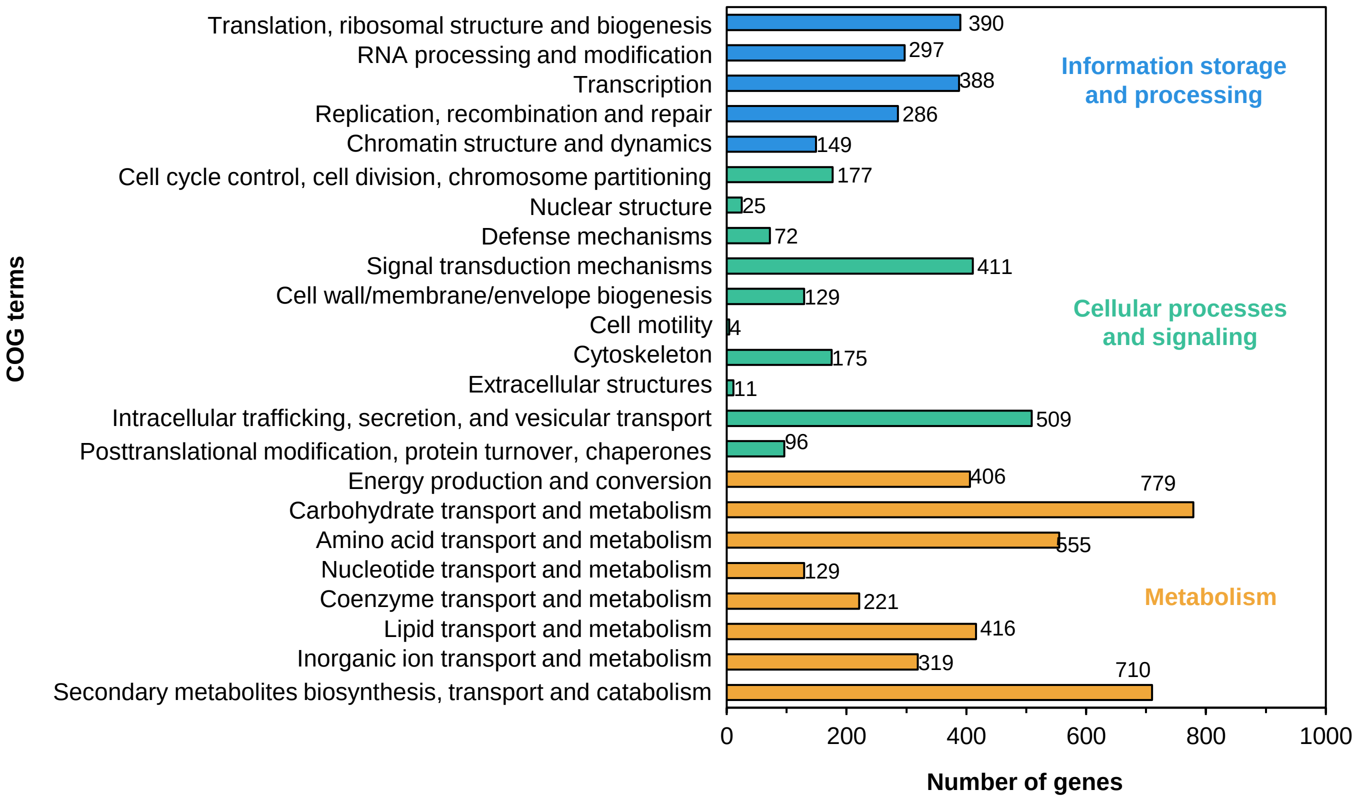

Figure S3 Functional annotation of *Tetraploa* sp. E00680 protein-coding genes based on Clusters of Orthologous Groups (COG) classification.

Figure S4

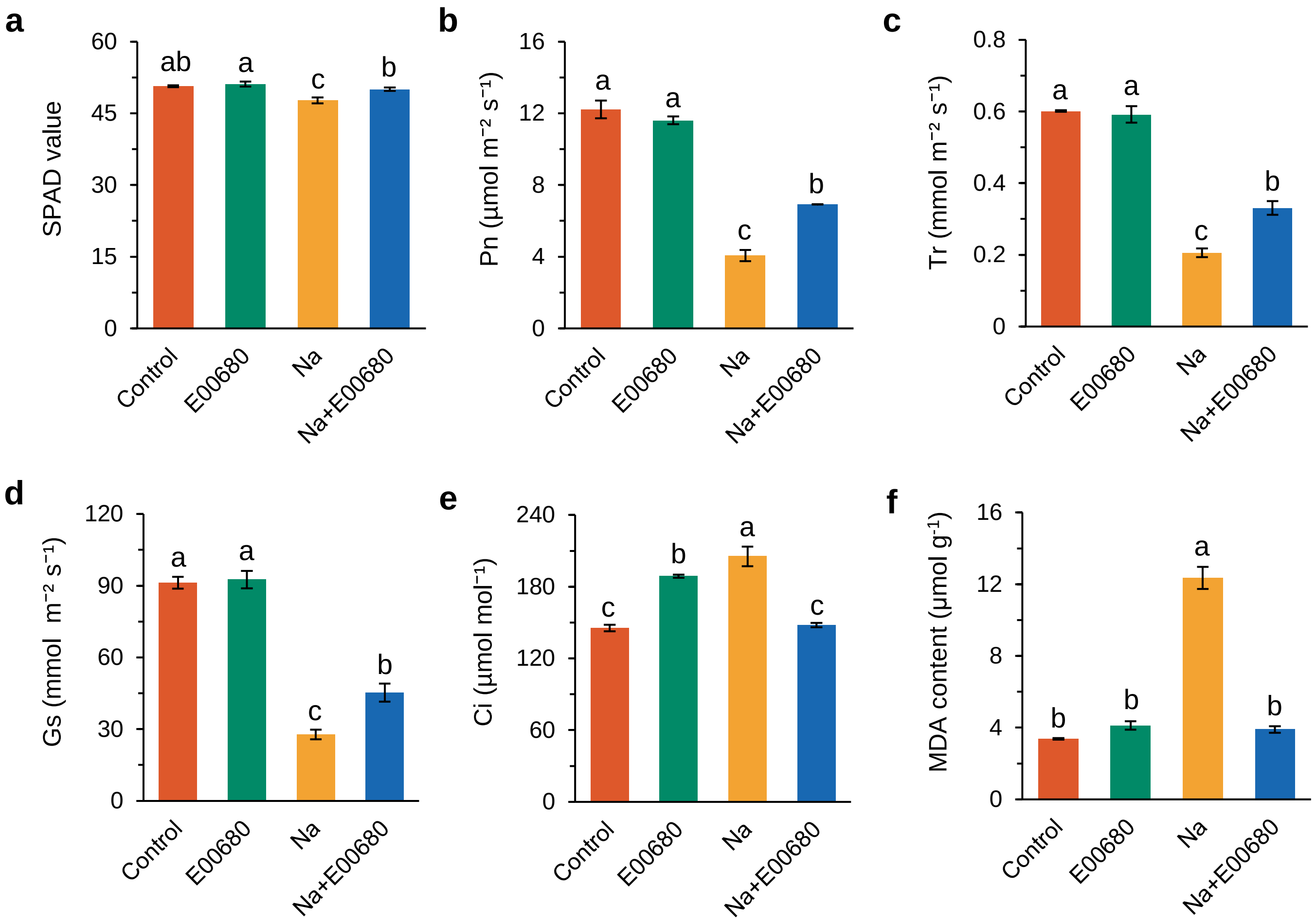

Figure S4 Effects of *Tetraploa* sp. E00680 inoculation on physiological traits and malondialdehyde (MDA) content in wheat under salt stress. SPAD value (a), net photosynthetic rate (Pn, b), transpiration rate (Tr, c), stomatal conductance (Gs, d), intercellular CO_2 concentration (Ci, e), and malondialdehyde content (f) of wheat plants during the tillering stage in Pot Experiment 1. Control, E00680, Na, and Na + E00680 represent wheat plants grown in plain soil, plain soil with E00680 inoculation, plain soil supplemented with 0.3% NaCl, and plain soil with both 0.3% NaCl and E00680 inoculation, respectively. Data represent mean ± s.e.m. (n = 6); lowercase letters indicate statistically significant differences (one-way ANOVA with Duncan's post hoc test, $p < 0.05$).

Figure S5

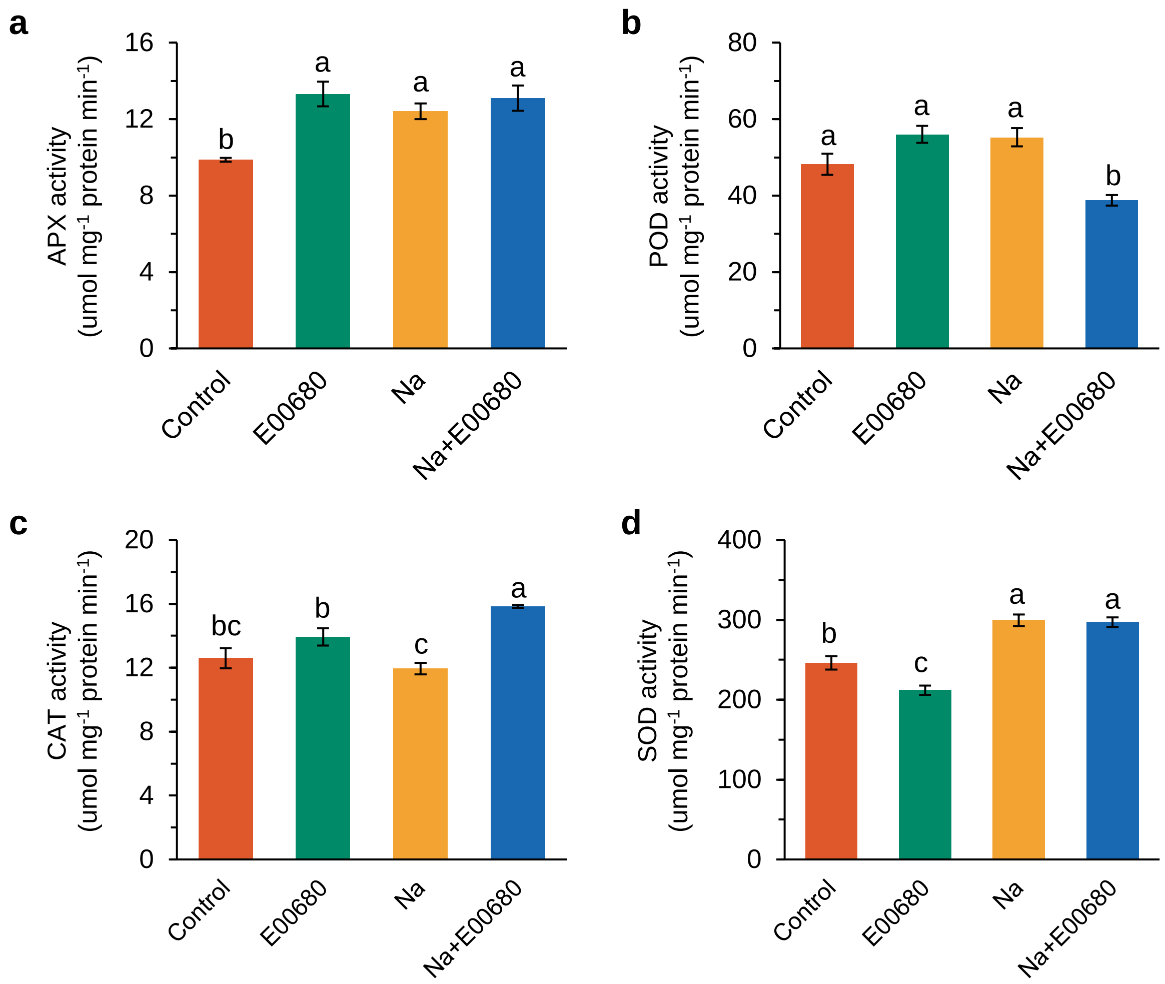

Figure S5 Effects of *Tetraploa* sp. E00680 inoculation on antioxidant enzyme activities in wheat under salt stress. Activities of ascorbate peroxidase (APX, a), peroxidase (POD, b), catalase (CAT, c), and superoxide dismutase (SOD, d) of wheat plants during the tillering stage in Pot Experiment 1. Control, E00680, Na, and Na + E00680 represent wheat plants grown in plain soil, plain soil with E00680 inoculation, plain soil supplemented with 0.3% NaCl, and plain soil with both 0.3% NaCl and E00680 inoculation, respectively. Data represent mean ± s.e.m. (n = 3); lowercase letters indicate statistically significant differences (one-way ANOVA with Duncan's post hoc test, $p < 0.05$).

Figure S6

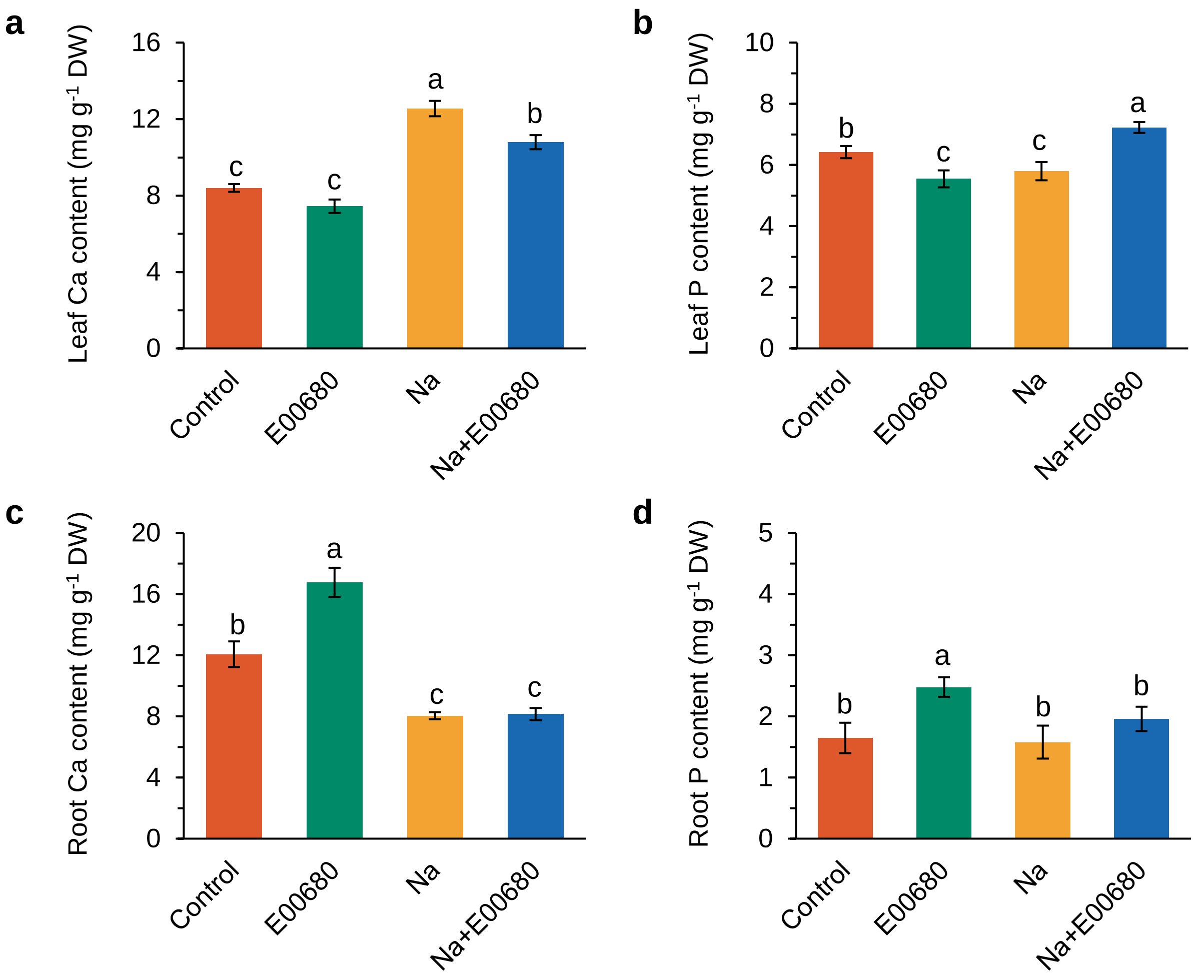

Figure S6 Effects of *Tetraploa* sp. E00680 inoculation on nutrient uptake in wheat under salt stress. Calcium (Ca) and phosphorus (P) content in the leaf (a,b) and root (c,d) of wheat plants during the tillering stage under salt stress in Pot Experiment 1. Control, E00680, Na, and Na + E00680 represent wheat plants grown in plain soil, plain soil with E00680 inoculation, plain soil supplemented with 0.3% NaCl, and plain soil with both 0.3% NaCl and E00680 inoculation, respectively. Data represent mean ± s.e.m. (n = 4); lowercase letters indicate statistically significant differences (one-way ANOVA with Duncan's post hoc test, $p < 0.05$).

Figure S7

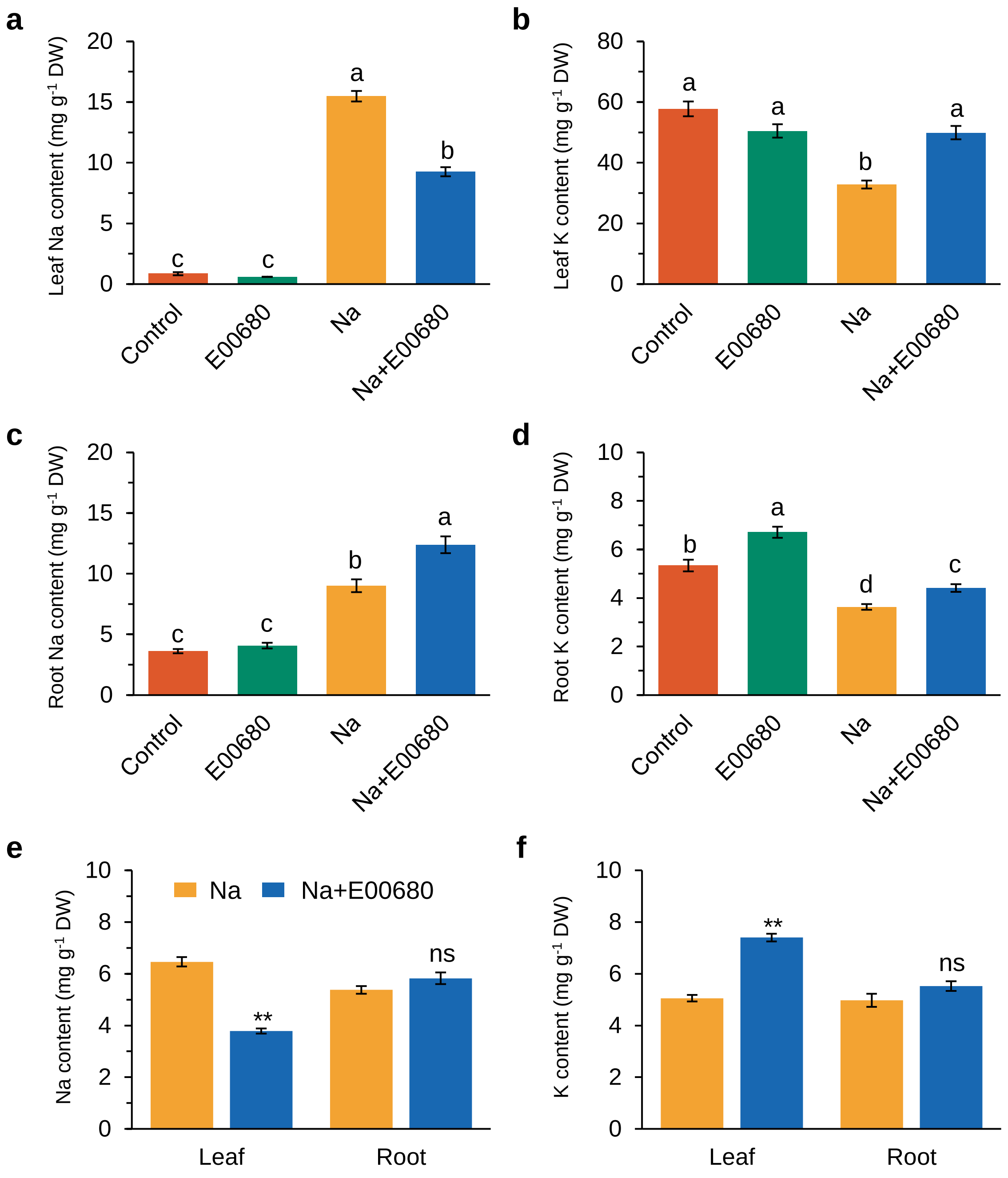

Figure S7 Effects of *Tetraploa* sp. E00680 inoculation on Na and K distribution in wheat plants under salt stress. (a–d) Na and K content in the leaves and roots of wheat plants during the tillering stage in Pot Experiment 1. Control, E00680, Na, and Na + E00680 represent wheat plants grown in plain soil, plain soil with E00680 inoculation, plain soil supplemented with 0.3% NaCl, and plain soil with both 0.3% NaCl and E00680 inoculation, respectively. Data represent mean ± s.e.m. (n = 4); lowercase letters indicate statistically significant differences (one-way ANOVA with Duncan's post hoc test, $p < 0.05$). (e,f) Na and K content in the leaves and roots of wheat plants during the flowering stage in the field experiment. Na and Na + E00680 represent wheat plants grown in saline-alkaline soil and saline-alkaline soil with E00680 inoculation, respectively. Data represent mean ± s.e.m. (n = 4); asterisks denote significant differences between Na + E00680 and Na treatments (two-tailed Student's *t*-test: $^{**}p < 0.01$, $^{*}p < 0.05$; ns, not significant).

Figure S8

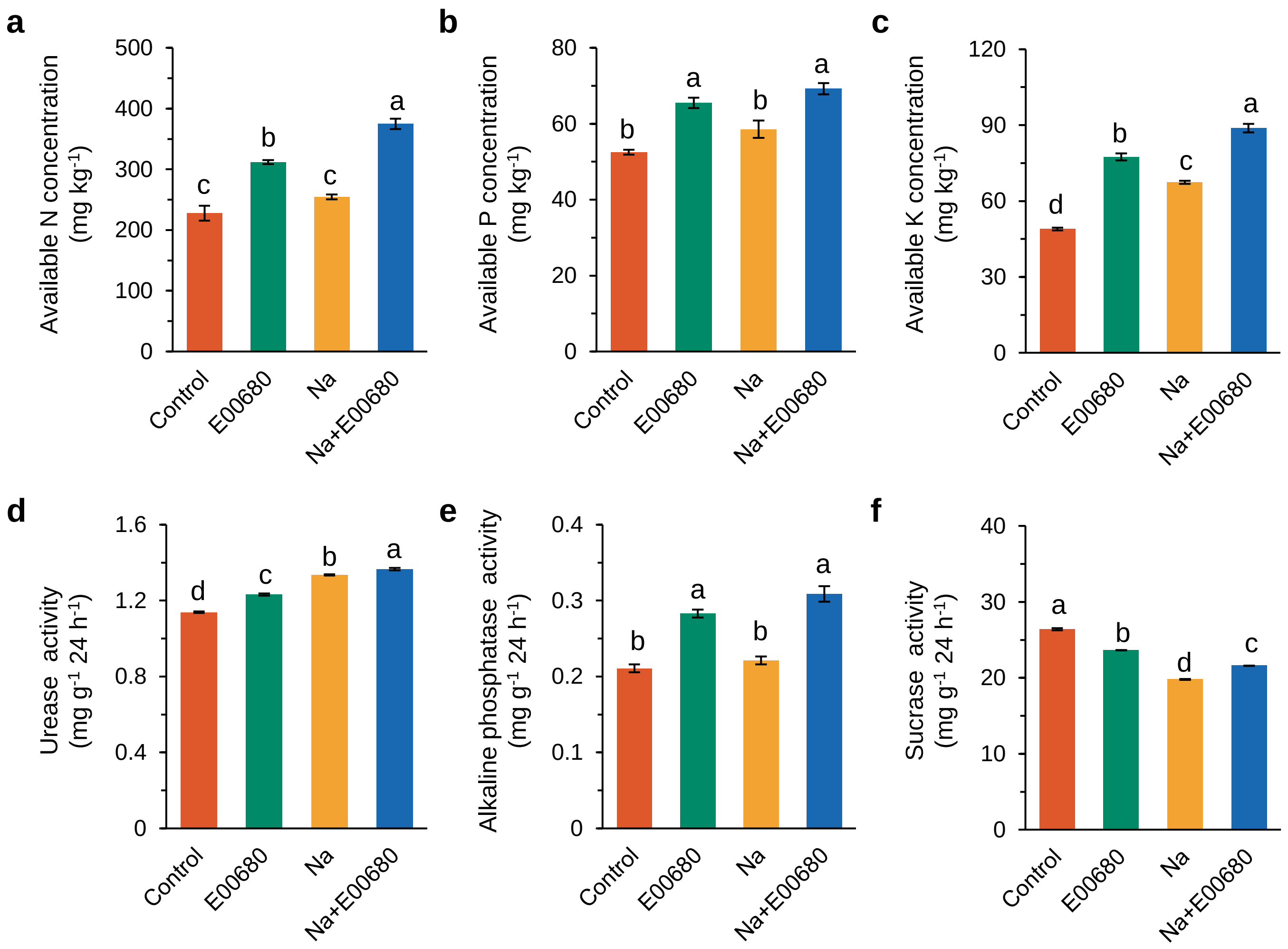

Figure S8 Effects of *Tetraploa* sp. E00680 inoculation on soil elemental contents and biochemical properties. Available nitrogen (N, a), phosphorus (P, b), potassium (K, c), urease activity (d), alkaline phosphatase activity (e), and sucrase activity (f) in rhizosphere soil of wheat plants during the tillering stage under salt stress in Pot Experiment 1. Control, E00680, Na, and Na + E00680 represent wheat plants grown in plain soil, plain soil with E00680 inoculation, plain soil supplemented with 0.3% NaCl, and plain soil with both 0.3% NaCl and E00680 inoculation, respectively. Data represent mean ± s.e.m. (n = 3); lowercase letters indicate statistically significant differences (one-way ANOVA with Duncan's post hoc test, $p < 0.05$).

Figure S9

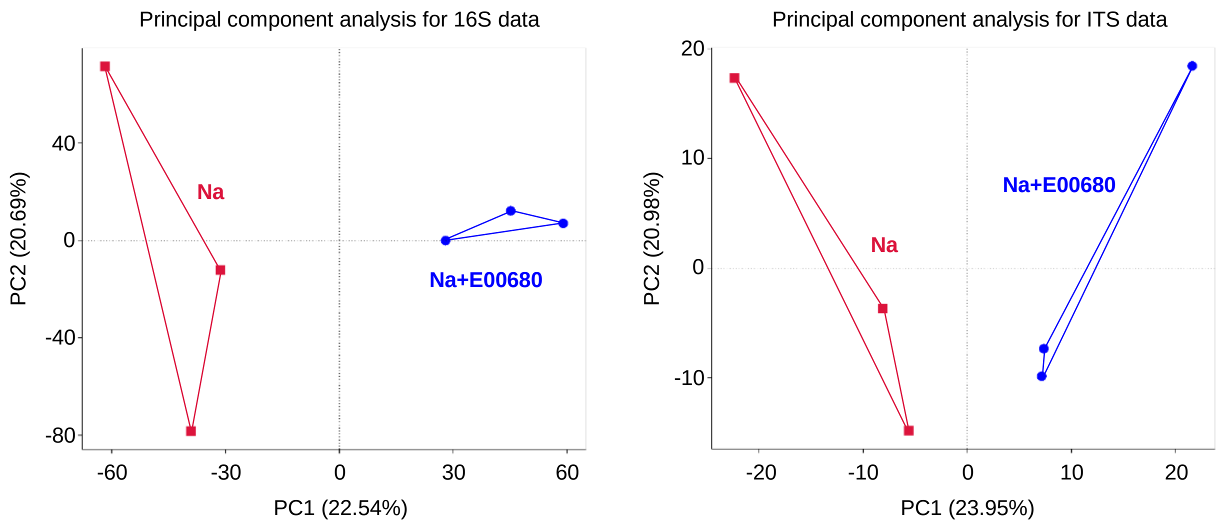

Figure S9 Principal component analysis (PCA) of bacterial (16S rRNA) and fungal (internal transcribed spacer, ITS) microbiomes. Na and Na + E00680 represent wheat plants grown in saline-alkaline soil and saline-alkaline soil with E00680 inoculation, respectively.

Figure S10

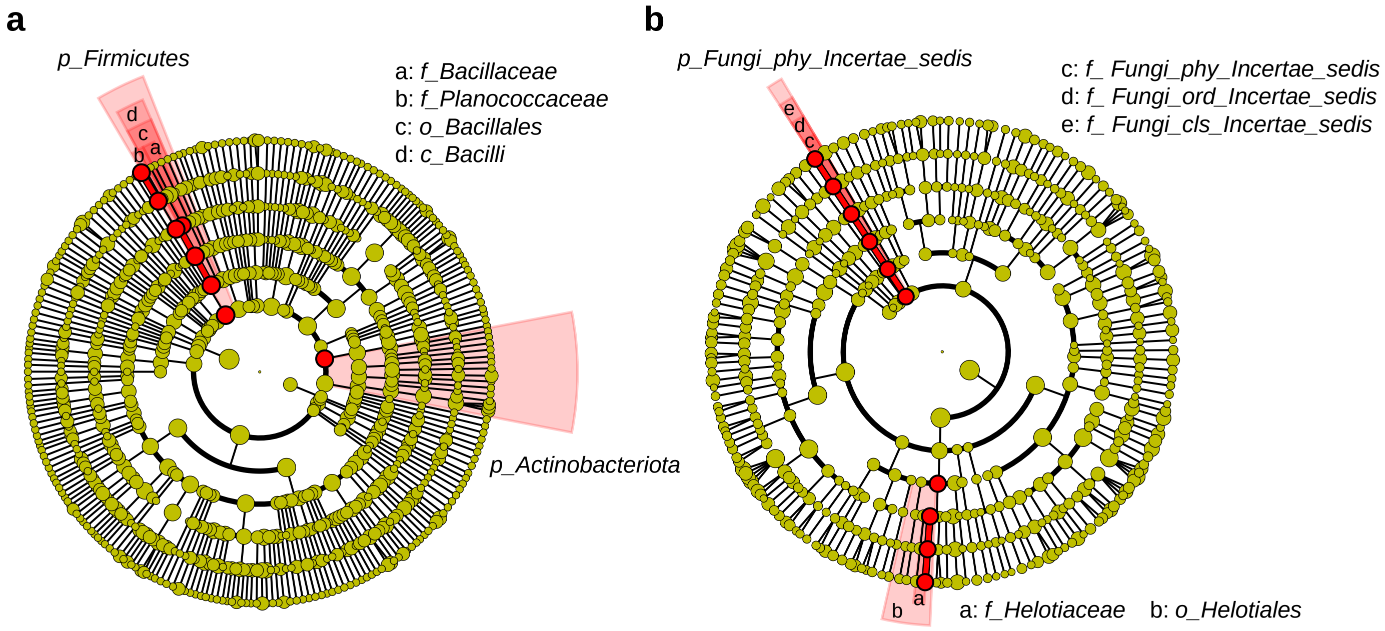

Figure S10 Differential rhizosphere microbiota profiling through Linear discriminant analysis Effect Size (LEfSe) analysis under salt stress with E00680 inoculation. Phylogenetic signatures showing statistically significant (p < 0.05) enrichment of bacterial (a) and fungal (b) taxa in E00680-treated versus control groups. Biomarker taxa with Linear discriminant analysis (LDA) scores exceeding the threshold (> 4.0) are indicated in red, while olive-shaded nodes represent microbial features without significant intergroup differences.

Figure S11

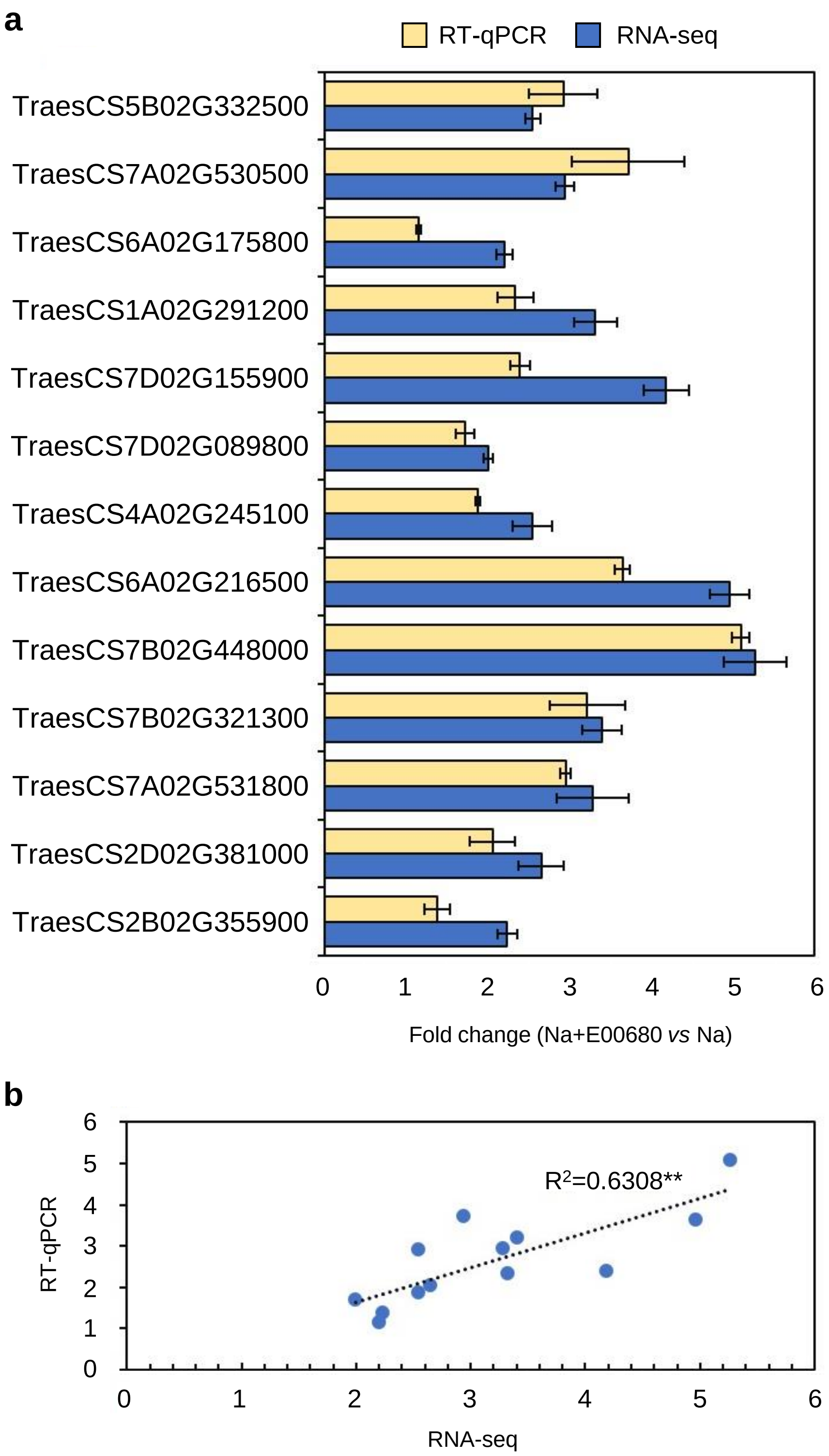

Figure S11 Experimental validation of transcriptomic data through reverse-transcription quantitative PCR (RT-qPCR) analysis. (a) Comparison of expression levels for 13 selected differentially expressed genes (DEGs) between RNA sequencing (RNA-seq) and RT-qPCR assays. (b) Correlation analysis of gene expression patterns between RNA-seq and RT-qPCR. ** indicates significant Spearman correlation ($p < 0.01$).

Figure S12

Na^+/K^+ homeostasis

2.58	TraesCS6D02G275100	Potassium transporter
2.34	TraesCS3D02G228500	Heavy metal transport/detoxification protein
1.64	TraesCS2A02G107100	Potassium transporter
1.25	TraesCS5B02G396400	Ion channel
1.21	TraesCS3D02G288100	SODIUM POTASSIUM ROOT DEFECTIVE
1.13	TraesCS5B02G176500	Ion channel
1.13	TraesCS2B02G123700	Potassium transporter
1.06	TraesCS7A02G183100	Sodium/calcium exchanger protein
1.05	TraesCS7B02G088100	Sodium/calcium exchanger protein
-1.13	TraesCS7B02G295600	Potassium transporter
-1.24	TraesCS7A02G393600	Potassium transporter
-1.35	TraesCS2D02G443700	Sodium/calcium exchanger protein
-1.43	TraesCS7A02G209900	Potassium transporter
-1.67	TraesCS2A02G444600	Sodium/calcium exchanger protein
-1.75	TraesCS7D02G211800	Potassium transporter
-1.76	TraesCS7B02G116700	Potassium transporter

Photosynthesis

3.49	TraesCS6D02G358900	Photosystem II 10 kDa polypeptide PsbR
3.00	TraesCS6B02G412100	Photosystem II 10 kDa polypeptide PsbR
2.69	TraesCS6B02G126500	Photosystem II 10 kDa phosphoprotein
1.39	TraesCS7B02G395800	ATP synthase alpha/beta family
1.26	TraesCS5A02G420800	Photosystem II reaction center protein K
1.17	TraesCS2B02G395700	Oxygen evolving enhancer protein (PsbQ)
1.11	TraesCS1D02G191700	Photosynthesis affected mutant 68
1.00	TraesCS2D02G206500	RbcX protein
-1.10	TraesCS5B02G179800	Phosphoenolpyruvate carboxylase
-1.15	TraesCS5B02G463100	Chlorophyll A-B binding protein
-1.27	TraesCS3B02G188200	Protein of unknown function (DUF2647)
-1.35	TraesCS5D02G186200	Phosphoenolpyruvate carboxylase
-1.40	TraesCS5A02G181800	Phosphoenolpyruvate carboxylase
-1.59	TraesCS7D02G276300	Chlorophyll A-B binding protein
-1.85	TraesCS1D02G375100	Chlorophyll A-B binding protein

Antioxidant defense

3.53	TraesCS3B02G354000	Peroxidase
2.11	TraesCS3B02G471500	Glutathione S-transferase
1.98	TraesCS6B02G429400	Glutathione S-transferase
1.70	TraesCS4A02G090200	Alpha-dioxygenase
1.36	TraesCS4B02G296500	Glutathione S-transferase
1.33	TraesCS2B02G125200	Peroxidase
1.17	TraesCS4B02G178700	Glucose-6-phosphate dehydrogenase
1.07	TraesCS2D02G107900	Peroxidase
-1.10	TraesCS6B02G278100	Glutathione peroxidase
-1.16	TraesCS1B02G331300	Peroxidase
-1.18	TraesCS2A02G144300	Glutathione S-transferase
-1.28	TraesCS1A02G347700	Ferric reductase like
-1.37	TraesCS7B02G275000	Peroxidase
-1.43	TraesCS1D02G096400	Peroxidase
-1.46	TraesCS2A02G437700	Peroxidase
-1.47	TraesCS5B02G021800	Glutathione S-transferase
-1.52	TraesCS6D02G305200	1-aminocyclopropane-1-carboxylate oxidase

Tryptophan metabolism

3.13	TraesCS5B02G566700	indole-3-pyruvate monooxygenase YUCCA1-like
2.50	TraesCS3A02G232600	indole-3-pyruvate monooxygenase YUCCA1-like
2.11	TraesCS2D02G437800	O-methyltransferase domain-containing protein
1.88	TraesCS1D02G052500	tryptophan decarboxylase 1
1.64	TraesCS1A02G113600	tryptophan aminotransferase-related protein 1-like
1.50	TraesCS3D02G305600	tyrosine decarboxylase
1.11	TraesCS3D02G246700	tryptophan aminotransferase-related protein 3
1.11	TraesCS3D02G093200	indole-3-pyruvate monooxygenase YUCCA1-like
1.05	TraesCS5A02G440700	tryptophan synthase alpha chain-like
1.02	TraesCS2A02G257700	Tryptophan synthase beta subunit-like
-1.49	TraesCS7D02G383500	Aromatic-L-amino-acid decarboxylase
-1.78	TraesCS5D02G488300	acetylserotonin O-methyltransferase 1
-2.55	TraesCS6D02G085100	tryptamine 5-hydroxylase
-3.14	TraesCS1B02G349100	acetylserotonin O-methyltransferase 1-like
-3.55	TraesCS1B02G348900	O-methyltransferase domain-containing protein

Lipid metabolism

2.69	TraesCS1D02G413000	protein RADIALIS-like
2.39	TraesCS1B02G388700	phosphomethylethanolamine N-methyltransferase-like
2.09	TraesCS1B02G373100	choline kinase
1.70	TraesCS4A02G090200	Alpha-dioxygenase 2
-1.00	TraesCS2A02G268700	sulfoquinovosyl transferase SQD2-like
-1.00	TraesCS1B02G304500	UTP-glucose-1-phosphate uridylyltransferase
-1.09	TraesCS1B02G133300	Glycerophosphoryl diester phosphodiesterase family
-1.10	TraesCS6B02G278100	Glutathione peroxidase
-1.13	TraesCS1A02G196600	Cytidylyltransferase-like
-1.16	TraesCS1A02G295200	UTP-glucose-1-phosphate uridylyltransferase
-1.16	TraesCS2B02G287500	sulfoquinovosyl transferase SQD2-like
-1.17	TraesCS2D02G267700	sulfoquinovosyl transferase SQD2-like
-1.29	TraesCS3D02G299100	glycerophosphodiester phosphodiesterase GDPD6
-1.38	TraesCS5A02G297000	Lecithin:cholesterol acyltransferase
-1.39	TraesCS5D02G304100	Lecithin:cholesterol acyltransferase
-1.67	TraesCS5B02G296300	Lecithin:cholesterol acyltransferase
-1.78	TraesCS3A02G055500	sulfoquinovosyl transferase SQD2-like
-2.05	TraesCS4A02G212800	Monogalactosyldiacylglycerol (MGDG) synthase
-2.35	TraesCS3A02G287500	Inorganic pyrophosphatase 2
-2.40	TraesCS7A02G263000	Glycerophosphoryl diester phosphodiesterase
-2.47	TraesCS3B02G322100	Inorganic pyrophosphatase 2
-2.52	TraesCS2A02G285900	Glycerophosphoryl diester phosphodiesterase
-2.55	TraesCS7D02G263700	Glycerophosphoryl diester phosphodiesterase
-2.64	TraesCS4D02G099600	Monogalactosyldiacylglycerol (MGDG) synthase
-2.66	TraesCS3B02G322000	Inorganic pyrophosphatase 2
-2.78	TraesCS4D02G093600	Fatty acid hydroxylase
-3.78	TraesCS4B02G015300	Phosphoesterase family

Figure S12 Functional categorization of salt stress-responsive genes modulated by E00680 inoculation. Comparative transcriptome heatmaps display differential gene expression patterns between two experimental groups. The log_2(Na + E00680 *vs* Na) ratio was used to assess gene expression changes. Genes were categorized as up-regulated (log2 ratio > 1, Q-value < 0.05) or down-regulated (log2 ratio < –1, Q-value < 0.05). Na and Na + E00680 represent wheat plants grown in saline-alkaline soil and saline-alkaline soil with E00680 inoculation, respectively.

Figure S13

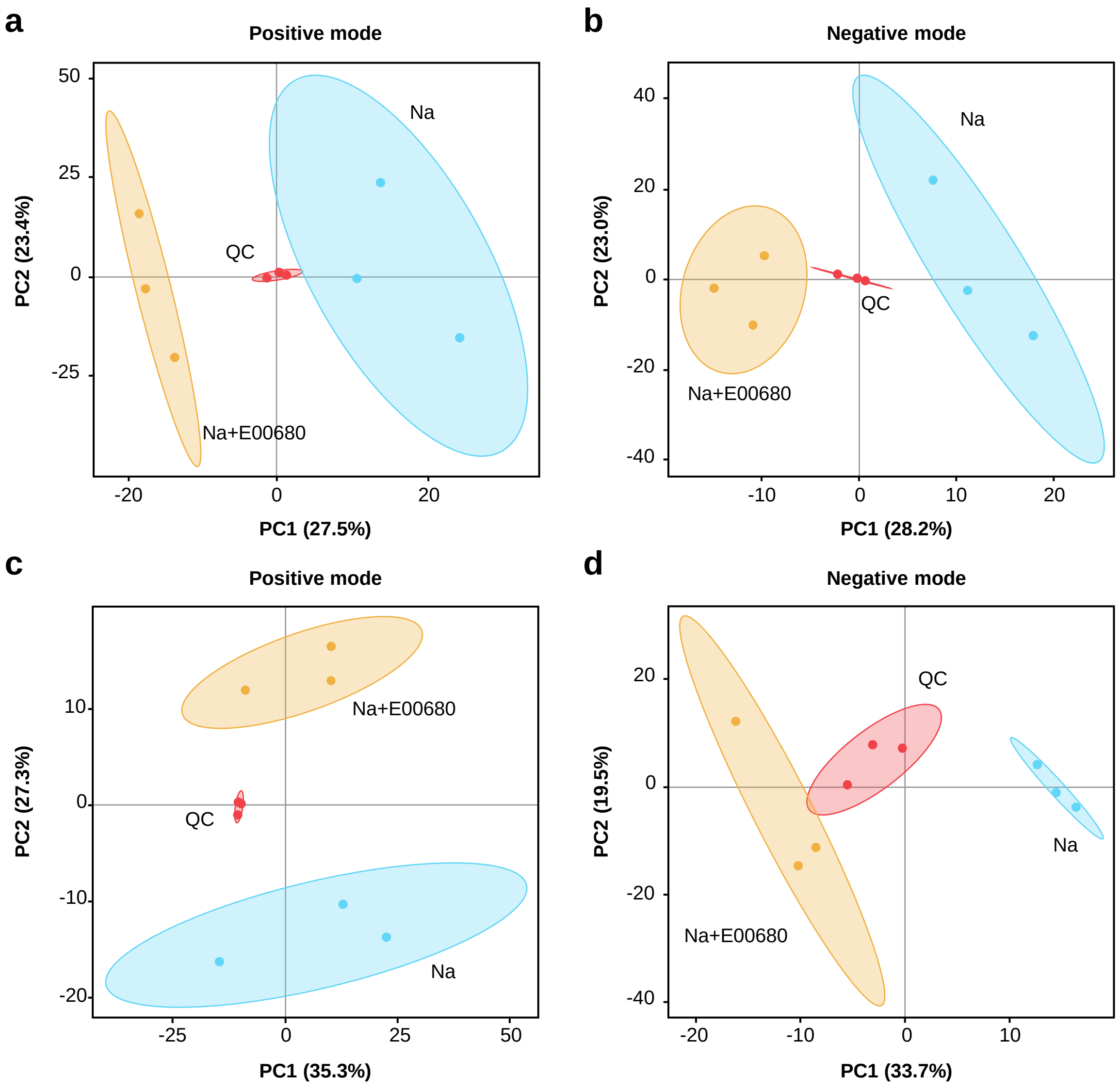

Figure S13 Principal component analysis (PCA) for leaf and soil metabolomes. (a,b) Plant samples in positive and negative ionization modes. (c,d) Soil samples in positive and negative ionization modes. Na and Na + E00680 represent wheat plants grown in saline-alkaline soil and saline-alkaline soil with E00680 inoculation, respectively. QC, quality control.

Figure S14

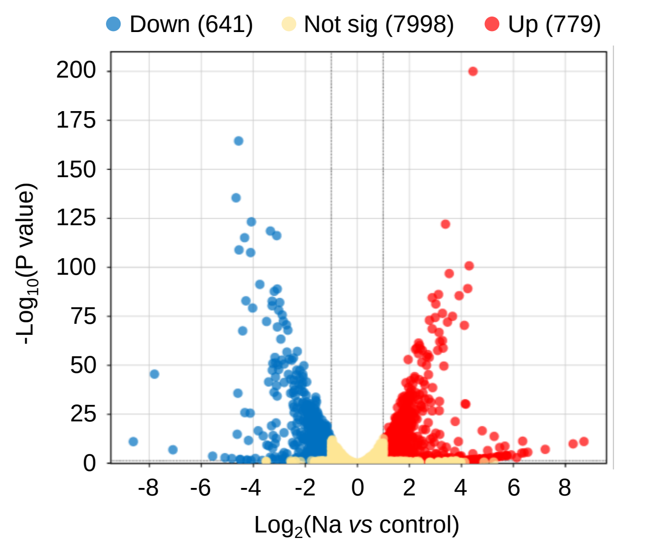

Ion transport and homeostasis

FUN_001752	*CNH1_1*	Na^+/H^+ antiporter
FUN_002303	*CCC2*	Cu^{2+}-transporting P-type ATPase
FUN_002385	*CNH1_2*	Na^+/H^+ antiporter
FUN_003269	*KCH1*	Potassium transporter
FUN_004857	*PMC1_1*	Plasma membrane calcium
FUN_005188	*ZRT1*	High-affinity Zn^{2+} transporter
FUN_005302	*YVC1*	Calcium channel
FUN_005780	*PMC1_2*	plasma membrane calcium
FUN_008865	*TRK1*	Low-affinity potassium transporter
FUN_009842	*ZRT2*	Low-affinity Zn^{2+} transporter
FUN_013109	*cta3*	Potassium/sodium efflux

Transcription regulation and signaling

FUN_000488	*RLM1*	Transcription factor rlm1
FUN_001858	*CRZ1*	DNA-binding transcription factor
FUN_006785	*PKC1_2*	Serine/threonine kinase
FUN_006849	*HAA1*	transcriptional activator haa1
FUN_007064	*YPD1*	Phosphorelay intermediate protein
FUN_008321	*HCM1*	Forkhead transcription factor
FUN_010203	*UME6_1*	DNA-binding transcriptional regulator
FUN_012180	*srk1*	MAPK-activated protein kinase Srk1

Antioxidant defense

FUN_000781	*ALD5*	aldehyde dehydrogenase 5
FUN_000970	*SOD4_1*	Cell surface superoxide dismutase 4
FUN_001787	*lcc1_2*	laccase, multicopper oxidase
FUN_002351	*cat1*	catalase 1
FUN_004609	*CAT1*	catalase A
FUN_006866	*SOD2_1*	Superoxide dismutase
FUN_008019	*GST2_2*	Glutathione S-transferase 2
FUN_009702	*GLR1_2*	Glutathione reductase
FUN_009993	*PRX1*	peroxiredoxin 1
FUN_012339	*AOX1*	Alternative oxidase
FUN_012397	*SOD4_2*	Cell surface superoxide dismutase 4

DNA repair and protein turnover

FUN_002607	*RAD53_2*	Protein kinase protein rad53
FUN_004142	*URM1*	Ubiquitin-related modifier 1
FUN_004627	*msh2*	MSH2 protein
FUN_005151	*atg17*	Autophagy-related protein 17
FUN_006782	*SNQ2_1*	ATP-binding cassette transporter snq2
FUN_007352	*GAD1*	glutamate decarboxylase gad1
FUN_009066	*UGA2_2*	succinate semialdehyde dehydrogenase
FUN_012853	*HSP31_2*	plasma membrane heat shock protein

Osmoregulation and cell integrity

FUN_000247	*CHS3_1*	Chitin synthase, class 3
FUN_001113	*ags1*	Cell wall alpha-1,3-glucan synthase
FUN_002026	*AQY1_1*	Aquaporin-1
FUN_004331	*ERG1*	Squalene epoxidase
FUN_006498	*ATH1*	alpha,alpha-trehalase
FUN_006697	*CHS3_2*	Chitin synthase, class 3
FUN_008368	*ERG3*	c-5 sterol desaturase
FUN_009085	*AQY1_3*	Aquaporin-1
FUN_010256	*TPS3*	Trehalose-6-P synthase/phosphatase
FUN_011293	*CRH1_2*	transglycosylase

Gene ID → Log_2(Na *vs* control) -3 0 +3

Figure S14 Transcriptome analysis of E00680 in response to salt stress. Differentially expressed genes (DEGs) are visualized in a volcano plot. Comparative transcriptome heatmaps display differential gene expression patterns between the two experimental groups. The log_2(Na *vs* control) ratio was used to assess gene expression changes. Genes were categorized as up-regulated (log2 ratio > 1, Q-value < 0.05) or down-regulated (log2 ratio < –1, Q-value < 0.05). The control and NaCl treatment groups represent E00680 cultured in an NaCl-free potato dextrose broth (PDB) medium and treated with 1% NaCl (~171.1 mM) for 24 h, respectively.

Figure S15

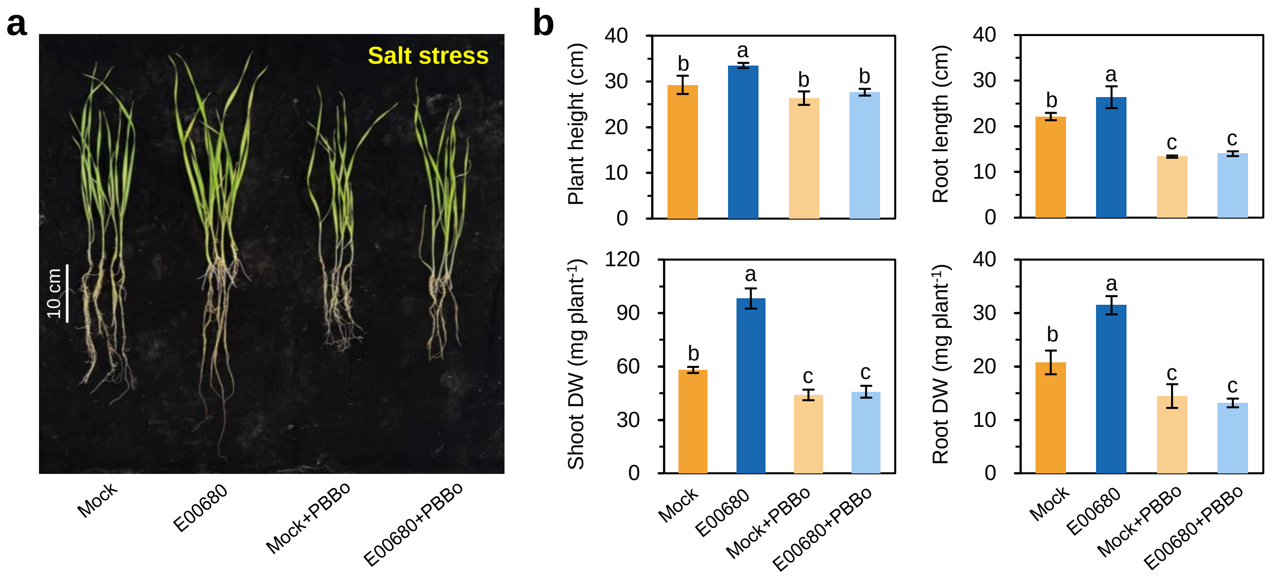

Figure S15 Effect of an alternative auxin biosynthesis inhibitor on Tetraploa sp. E00680-mediated salt stress tolerance in wheat. (a) Representative plant morphology. Hydroponically grown wheat seedlings under salt stress (BNS supplemented with 100 mM NaCl) were subjected to four treatments: Mock (non-inoculated), E00680 (inoculated with 100 mL microbial inoculum), Mock + PPBo (non-inoculated, supplemented with 3 µM PPBo), and E00680 + PPBo (inoculated with 100 mL microbial inoculum and 3 µM PPBo). BNS, basic nutrient solution; PPBo, 4-phenoxyphenylboronic acid. (b) Plant growth parameters of wheat seedlings across the different treatments. DW, dry weight. Data represent mean ± s.e.m. (n = 4); lowercase letters indicate statistically significant differences (one-way ANOVA with Duncan's post hoc test, $p <$ 0.05).

Figure S16

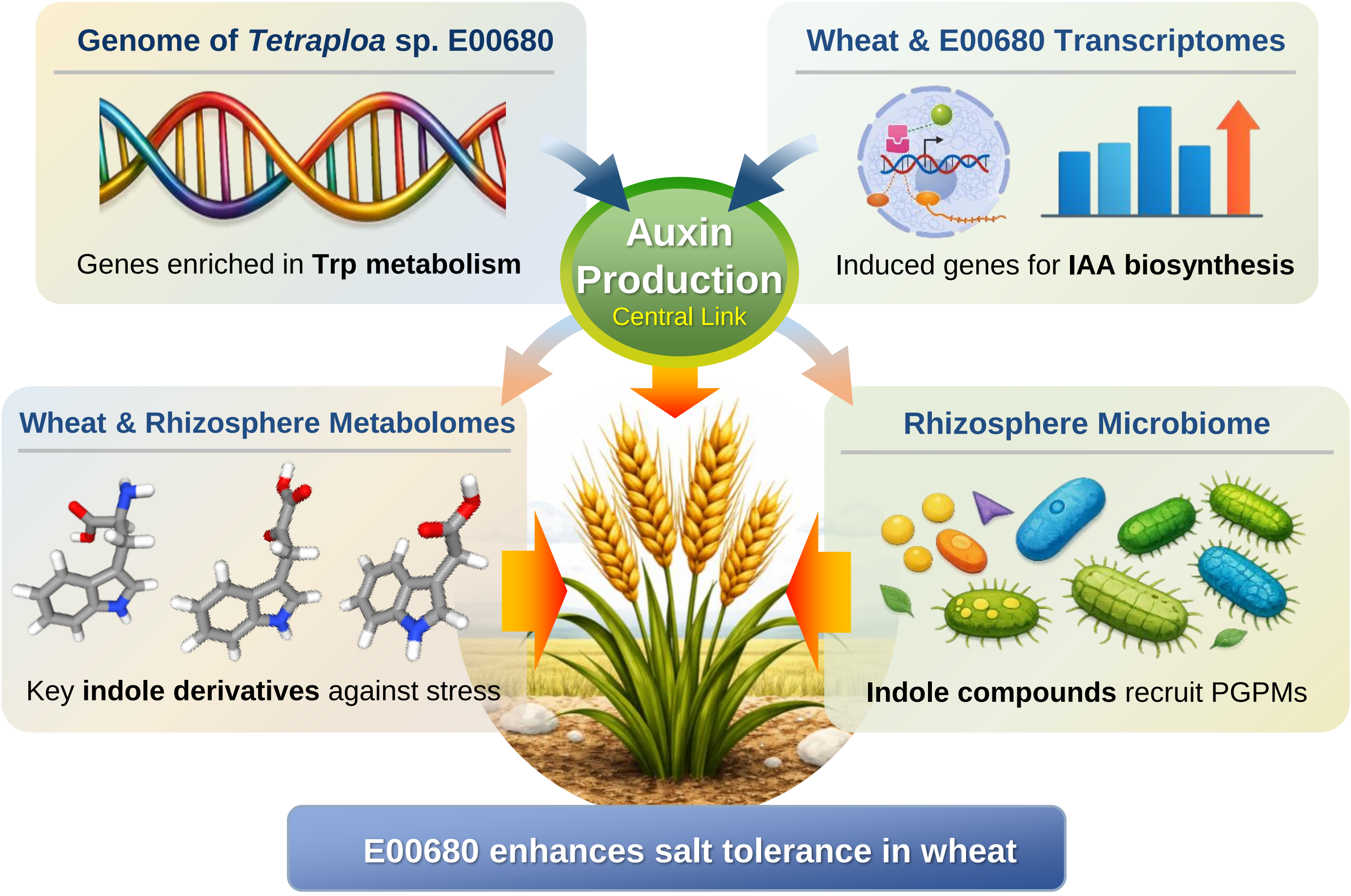

Figure S16 A schematic diagram summarizing multi-omics interactions. Auxin production mediated by E000680 serves as a central hub connecting genomic, transcriptomic, metabolomic, and microbiome data, conferring salt stress tolerance in wheat. Trp, tryptophan; IAA, indole-3-acetic acid.
