# Supplementary material for: First Tetraploa Genome and Multi‐Omics Analysis Reveal Key Plant‐Microbe‐Soil Interactions for Salt Tolerance and Yield Improvement of Wheat
Source: Plant Biotechnol J. 2026 Apr 3;24(8):4748–65. doi: 10.1111/pbi.70663 (PMC13387892; doi:10.1111/pbi.70663)
Supplement: Supplementary file 2 — Table S1: COG‐based functional annotation of predicted genes in E00680. Table S2: KEGG‐based functional annotation of predicted genes in E00680. Table S3: Predicted pathogen‐host interaction genes in E00680. Table S4: Predicted secondary metabolite biosynthetic gene clusters in E00680. Table S5: Predicted virulence‐associated genes in E00680. Table S6: Up‐regulated metabolites in wheat plants after E00680 inoculation under salt stress. Table S7: Up‐regulated metabolites in rhizosphere soil after E00680 inoculation under salt stress. Table S8: Key genes related to E00680‐mediated salt stress tolerance. Table S9: BLAST‐based comparison of key genes associated with salt tolerance in E00680 and Serendipita indica. Table S10: Primer sequences used for PCR and qPCR validation. Table S11: GenBank accession numbers for phylogenetic analyses. [file PBI-24-4748-s002.pdf]

Table S1 COG-based functional annotation of predicted genes in E00680

Gene ID	Scaffold	Start	Stop	Strand	Name	Product
FUN_000002	contig_1	3964	4827	+		hypothetical protein
FUN_000005	contig_1	11738	12896	-		hypothetical protein
FUN_000006	contig_1	13273	14406	-		hypothetical protein
FUN_000007	contig_1	19613	21097	-		hypothetical protein
FUN_000009	contig_1	35229	35753	+		hypothetical protein
FUN_000010	contig_1	36562	38200	-		hypothetical protein
FUN_000011	contig_1	38584	40315	-		hypothetical protein
FUN_000013	contig_1	51371	52644	-		hypothetical protein
FUN_000014	contig_1	53042	54106	+	USB1	poly(U)-specific 3'-to-5' RNA exonuclease
FUN_000016	contig_1	65114	67603	-	NPL4_1	nuclear protein localization protein 4
FUN_000017	contig_1	68002	70044	+	NPL4_2	nuclear protein localization protein 4
FUN_000018	contig_1	71357	72703	-		hypothetical protein
FUN_000020	contig_1	75254	78027	-	HMLALPHA2_1	homeodomain mating type protein alpha2
FUN_000021	contig_1	86155	88658	+	DAL81_1	Fungal specific transcription factor
FUN_000023	contig_1	101652	104598	-	msh1	MutS protein 1
FUN_000024	contig_1	105080	106362	+	HEM12	Uroporphyrinogen decarboxylase in heme biosynthesis
FUN_000025	contig_1	106402	107013	-	FCY1	Cytosine deaminase
FUN_000026	contig_1	107755	109078	+	YDJ1	Type I HSP40 co-chaperone
FUN_000029	contig_1	126943	128638	+		hypothetical protein
FUN_000030	contig_1	129982	131065	+		hypothetical protein
FUN_000031	contig_1	131112	131924	-		hypothetical protein
FUN_000035	contig_1	145734	148194	+		hypothetical protein
FUN_000039	contig_1	157717	158471	+		hypothetical protein
FUN_000040	contig_1	158510	159421	+		hypothetical protein
FUN_000041	contig_1	159720	163812	-		hypothetical protein
FUN_000042	contig_1	164613	165827	+		hypothetical protein
FUN_000043	contig_1	166192	167988	+		hypothetical protein
FUN_000044	contig_1	178147	179404	-		hypothetical protein
FUN_000047	contig_1	189228	189542	-		hypothetical protein
FUN_000048	contig_1	190455	192498	+		hypothetical protein
FUN_000050	contig_1	196585	197538	-		hypothetical protein
FUN_000051	contig_1	201722	203309	+		hypothetical protein
FUN_000052	contig_1	204064	205598	-	ERV46	ER-derived vesicles protein erv46
FUN_000053	contig_1	206109	208649	-		hypothetical protein
FUN_000054	contig_1	210474	211628	+	cdc16	CDC16 protein
FUN_000055	contig_1	212102	212446	+		hypothetical protein
FUN_000056	contig_1	214667	217189	-	KEX2	pheromone processing endoprotease
FUN_000058	contig_1	224416	226251	+		hypothetical protein
FUN_000059	contig_1	228299	229027	+		hypothetical protein
FUN_000060	contig_1	231739	235761	-		hypothetical protein
FUN_000061	contig_1	239727	240869	+		hypothetical protein
FUN_000062	contig_1	241333	242503	+		hypothetical protein
FUN_000063	contig_1	242553	245415	-		hypothetical protein
FUN_000064	contig_1	247012	248266	+		hypothetical protein
FUN_000065	contig_1	249207	250514	+		hypothetical protein
FUN_000066	contig_1	253516	254646	-		hypothetical protein
FUN_000068	contig_1	259110	260135	+		hypothetical protein
FUN_000069	contig_1	260277	262508	-		hypothetical protein
FUN_000070	contig_1	263801	264634	-		hypothetical protein
FUN_000073	contig_1	268634	269821	-		hypothetical protein
FUN_000074	contig_1	318291	320702	-		hypothetical protein
FUN_000075	contig_1	320744	322422	+		hypothetical protein
FUN_000076	contig_1	322626	324159	+		hypothetical protein
FUN_000077	contig_1	325258	326116	+		hypothetical protein
FUN_000078	contig_1	327143	329690	-		hypothetical protein
FUN_000079	contig_1	331023	332562	+		hypothetical protein
FUN_000080	contig_1	333120	334305	+	GAL80	transcription regulator gal80

Gene ID	Scaffold	Start	Stop	Strand	Name	Product
FUN_000081	contig_1	334393	339691	-		hypothetical protein
FUN_000082	contig_1	341548	343011	+		hypothetical protein
FUN_000084	contig_1	344153	346109	+		hypothetical protein
FUN_000085	contig_1	346137	347843	-		hypothetical protein
FUN_000086	contig_1	349301	350439	-		hypothetical protein
FUN_000087	contig_1	351773	352794	+		hypothetical protein
FUN_000091	contig_1	363631	365100	+	CMK2	Calmodulin-dependent protein kinase cmk2
FUN_000092	contig_1	365636	368310	+		hypothetical protein
FUN_000094	contig_1	371240	372683	-		hypothetical protein
FUN_000095	contig_1	374115	375213	-	GRE2_1	methylglyoxal reductase (NADPH-dependent) gre2
FUN_000096	contig_1	376636	377874	+		hypothetical protein
FUN_000097	contig_1	378137	379940	-		hypothetical protein
FUN_000098	contig_1	380951	383936	-		hypothetical protein
FUN_000099	contig_1	384761	386477	+		hypothetical protein
FUN_000100	contig_1	387475	388600	+		hypothetical protein
FUN_000102	contig_1	390952	392559	-		hypothetical protein
FUN_000103	contig_1	393191	394111	-		hypothetical protein
FUN_000104	contig_1	394673	397201	+		hypothetical protein
FUN_000105	contig_1	397512	400508	-		hypothetical protein
FUN_000106	contig_1	400999	402413	+	ERG10	erg10, acetyl-CoA C-acetyltransferase
FUN_000107	contig_1	404709	406382	+		hypothetical protein
FUN_000109	contig_1	425089	425899	-	YTH1	RNA-binding component of cleavage and polyadenylation factor
FUN_000110	contig_1	427498	428720	+		hypothetical protein
FUN_000111	contig_1	429349	432086	+	VPS53	Vacuolar protein sorting-associated protein 53
FUN_000113	contig_1	440749	442676	+		hypothetical protein
FUN_000115	contig_1	445375	446500	+	CAP2	F-actin-capping protein subunit beta
FUN_000116	contig_1	446982	448672	+		hypothetical protein
FUN_000117	contig_1	451108	452812	-		hypothetical protein
FUN_000118	contig_1	455468	456466	-		hypothetical protein
FUN_000119	contig_1	457457	459001	+		hypothetical protein
FUN_000120	contig_1	459143	460236	-		hypothetical protein
FUN_000121	contig_1	460935	464824	-		hypothetical protein
FUN_000124	contig_1	471297	473843	+		hypothetical protein
FUN_000125	contig_1	474586	477802	+		hypothetical protein
FUN_000126	contig_1	478341	480806	-	NOP14	nucleolar complex protein 14
FUN_000128	contig_1	482611	484008	-	TIF1	translation initiation factor eIF4A
FUN_000129	contig_1	484440	486166	+		hypothetical protein
FUN_000130	contig_1	486563	488264	-	SNX4	intercellular trafficking and secretion
FUN_000131	contig_1	488540	489382	-	ADK2	Adenylate kinase 2
FUN_000132	contig_1	490126	491880	+	YLH47	LETM1 domain-containing protein ylh47
FUN_000133	contig_1	492242	493289	+	PCD1	8-oxo-dGTP diphosphatase
FUN_000134	contig_1	493775	494899	-	MEX67	nuclear mRNA export, poly(A)+RNA binding protein
FUN_000135	contig_1	496623	497360	-	TMA22	Translation machinery-associated protein 22
FUN_000136	contig_1	498506	499117	+	GIM4	Cochaperone prefoldin complex subunit
FUN_000137	contig_1	501296	502361	+		hypothetical protein
FUN_000138	contig_1	503099	504426	-	ECM39	Dol-P-Man:Man(7)GlcNAc(2)-PP-Dol alpha-1,6-mannosyltransferase
FUN_000139	contig_1	505629	506518	+	PFA4	Palmitoyltransferase
FUN_000140	contig_1	506673	508302	-		hypothetical protein
FUN_000141	contig_1	509413	510617	-		hypothetical protein
FUN_000142	contig_1	511654	514130	+		hypothetical protein
FUN_000143	contig_1	514802	521431	+	brr2	Pre-mRNA-splicing helicase BRR2
FUN_000144	contig_1	521736	522998	-		hypothetical protein
FUN_000145	contig_1	523762	524725	-		hypothetical protein
FUN_000146	contig_1	526355	527539	+		hypothetical protein
FUN_000147	contig_1	527962	529836	+	ORC3	Origin recognition complex subunit 3
FUN_000148	contig_1	530209	530912	-	BNA1	3-hydroxyanthranilic acid dioxygenase
FUN_000149	contig_1	531165	531980	+	CTR2	copper transppport protein

Gene ID	Scaffold	Start	Stop	Strand	Name	Product
FUN_000150	contig_1	532442	534559	-	OSM2	Osmotic growth protein
FUN_000151	contig_1	535707	537766	+		hypothetical protein
FUN_000152	contig_1	538042	539253	-		hypothetical protein
FUN_000153	contig_1	539762	540905	-	ARC35	Arp complex subunit
FUN_000154	contig_1	541222	544145	+	DIP2	beta transducin
FUN_000155	contig_1	544505	545443	-	tfs1	transcription elongation factor TFIIS
FUN_000156	contig_1	545770	546432	+		hypothetical protein
FUN_000157	contig_1	549234	550436	-		hypothetical protein
FUN_000158	contig_1	554372	557868	-		hypothetical protein
FUN_000161	contig_1	565721	568167	-		hypothetical protein
FUN_000162	contig_1	569468	572231	-		hypothetical protein
FUN_000163	contig_1	574431	575293	+	lcl3	putative endonuclease lcl3
FUN_000164	contig_1	575978	579911	+	CPA2	carbamoyl-phosphate synthase (glutamine-hydrolyzing) cpa2
FUN_000166	contig_1	582063	585443	-		hypothetical protein
FUN_000167	contig_1	585704	587328	+	atg6	Vacuolar protein sorting-associated protein atg6
FUN_000169	contig_1	592960	593866	+	PST2	flavodoxin-like fold protein
FUN_000170	contig_1	594717	596433	-	ZRC1	Zinc resistance conferring protein
FUN_000171	contig_1	597228	598581	-		hypothetical protein
FUN_000172	contig_1	599188	600546	-		hypothetical protein
FUN_000173	contig_1	605378	606341	-		hypothetical protein
FUN_000175	contig_1	609884	611358	-	NdufS2	ndufs2, NADH ubiquinone oxidoreductase 49 kd subunit
FUN_000176	contig_1	611590	612830	+	RPC40	DNA-directed RNA polymerase core subunit rpc40
FUN_000177	contig_1	615555	616705	-	ATP3	atp3 gamma subunit of the F1 sector of mitochondrial F1F0 ATP synthase
FUN_000178	contig_1	617704	620638	+	TOP1	DNA topoisomerase 1
FUN_000179	contig_1	622346	623605	+		hypothetical protein
FUN_000180	contig_1	624298	626903	-		hypothetical protein
FUN_000181	contig_1	627986	629583	-		hypothetical protein
FUN_000182	contig_1	629904	631354	+		hypothetical protein
FUN_000183	contig_1	631764	634015	+	VPS33	Vacuolar protein-sorting-associated protein 33
FUN_000184	contig_1	634650	636824	+		hypothetical protein
FUN_000187	contig_1	641304	642751	-		hypothetical protein
FUN_000188	contig_1	644044	645619	-		hypothetical protein
FUN_000189	contig_1	647009	648170	-		hypothetical protein
FUN_000190	contig_1	648245	649052	-		hypothetical protein
FUN_000191	contig_1	649977	653073	-		hypothetical protein
FUN_000192	contig_1	654146	656301	+	SSB1	Heat shock protein ssb1
FUN_000194	contig_1	661331	661631	-	RPS13	ribosomal 40S subunit protein S13
FUN_000195	contig_1	662356	663661	+	TOS1	target of Sbf
FUN_000196	contig_1	664278	665704	+	PEX13	Peroxisomal membrane protein PAS20
FUN_000197	contig_1	666143	666939	-		hypothetical protein
FUN_000198	contig_1	667992	671794	+	prp10	U2 snRNP component prp10
FUN_000199	contig_1	676116	680156	-		hypothetical protein
FUN_000200	contig_1	680925	682131	-	CKA2	Casein kinase II subunit alpha'
FUN_000201	contig_1	682600	683814	+	RPP1	RNA-binding RNA processing protein rpp1
FUN_000203	contig_1	686792	689589	+	PIC2_1	Cu/Pi carrier
FUN_000204	contig_1	691423	692191	+	MDE1	Methylthioribulose-1-phosphate dehydratase
FUN_000205	contig_1	693088	694392	+		hypothetical protein
FUN_000206	contig_1	695271	696146	+		hypothetical protein
FUN_000208	contig_1	698533	700257	+	FRS2	Phenylalanyl-tRNA synthetase, beta subunit, cytoplasmic
FUN_000209	contig_1	700517	703745	-	NAT10	N-acetyltransferase 10
FUN_000210	contig_1	707174	708731	+		hypothetical protein
FUN_000211	contig_1	710334	715074	+		hypothetical protein
FUN_000212	contig_1	716238	717803	-		hypothetical protein
FUN_000213	contig_1	718563	723376	-	VPS8	Vacuolar protein sorting-associated protein 8
FUN_000214	contig_1	723831	724766	+		hypothetical protein
FUN_000216	contig_1	728889	729567	-		hypothetical protein
FUN_000217	contig_1	733223	735347	+		hypothetical protein

Gene ID	Scaffold	Start	Stop	Strand	Name	Product
FUN_000220	contig_1	746650	749250	-		hypothetical protein
FUN_000223	contig_1	751987	753372	-	dus2	tRNA-dihydrouridine synthase 2
FUN_000224	contig_1	754093	758118	+	MYO1_1	class II myosin
FUN_000225	contig_1	759855	762828	+		hypothetical protein
FUN_000226	contig_1	763338	764014	+		hypothetical protein
FUN_000227	contig_1	764681	770042	-	SIN3_1	Transcriptional regulatory protein sin3
FUN_000228	contig_1	770726	773758	-	ALA1	Alanine--tRNA ligase
FUN_000230	contig_1	775574	776213	-		hypothetical protein
FUN_000231	contig_1	777543	782842	-	SNT2	putative PHD type zinc finger protein with BAH domain-containing protein
FUN_000232	contig_1	787486	791598	+	MPH1	3'-5' DNA helicase
FUN_000233	contig_1	791927	793863	-	ERO1	endoplasmic oxidoreductin-1
FUN_000234	contig_1	794472	795102	+		hypothetical protein
FUN_000235	contig_1	797276	799968	-	MYO1_2	class II myosin
FUN_000236	contig_1	800464	801251	+		hypothetical protein
FUN_000237	contig_1	804528	806367	+	PTR2_1	peptide transporter ptr2
FUN_000238	contig_1	807361	809180	-		hypothetical protein
FUN_000239	contig_1	809550	814141	+	CFT1	mRNA cleavage and polyadenylation factor subunit
FUN_000240	contig_1	814555	815974	+		hypothetical protein
FUN_000242	contig_1	819971	821799	+		hypothetical protein
FUN_000243	contig_1	823832	827776	+		hypothetical protein
FUN_000244	contig_1	830138	830637	+	OST2	oligosaccharyltransferase complex subunit epsilon
FUN_000245	contig_1	830922	832625	-	ARO8_1	Aromatic/aminoadipate aminotransferase 1
FUN_000246	contig_1	833552	835586	-	CHS4	Chitin synthase 4
FUN_000247	contig_1	838036	841852	+	CHS3_1	Chitin synthase, class 3
FUN_000248	contig_1	843059	845094	+		hypothetical protein
FUN_000249	contig_1	846844	849185	-		hypothetical protein
FUN_000250	contig_1	850084	850901	-		hypothetical protein
FUN_000252	contig_1	855326	856432	+	IPP1	Inorganic pyrophosphatase
FUN_000253	contig_1	857336	858723	+	SEC20	Protein transport protein sec20
FUN_000255	contig_1	860729	865283	+	taf2	Transcription initiation factor TFIID subunit 2
FUN_000256	contig_1	865949	866733	-		hypothetical protein
FUN_000257	contig_1	867325	868409	-	ORT1	mitochondrial ornithine carrier protein
FUN_000258	contig_1	869206	869679	+	CGR1_1	rRNA-processing protein cgr1
FUN_000263	contig_1	885940	886418	-		hypothetical protein
FUN_000264	contig_1	887351	889184	-		hypothetical protein
FUN_000266	contig_1	892794	893976	+		hypothetical protein
FUN_000268	contig_1	897863	900019	-		hypothetical protein
FUN_000270	contig_1	907379	908715	+	MNN10	alpha-1,6-mannosyltransferase
FUN_000274	contig_1	915503	916320	-		hypothetical protein
FUN_000275	contig_1	916792	918531	+		hypothetical protein
FUN_000276	contig_1	919215	919961	-	RPS15	ribosomal protein S15
FUN_000277	contig_1	920974	921431	-	RPP2B	60S acidic ribosomal protein P2
FUN_000278	contig_1	921912	923109	+		hypothetical protein
FUN_000279	contig_1	924906	926439	+		hypothetical protein
FUN_000280	contig_1	926931	928190	+		hypothetical protein
FUN_000282	contig_1	931273	933648	-	ATP1	Alpha subunit of the F1 sector of mitochondrial F1F0 ATP synthase
FUN_000283	contig_1	934383	935131	-		hypothetical protein
FUN_000284	contig_1	935709	936369	+	RPB7	DNA-directed RNA polymerase II subunit
FUN_000285	contig_1	937061	939895	+		hypothetical protein
FUN_000286	contig_1	940273	941351	+		hypothetical protein
FUN_000287	contig_1	941539	944190	-	SEC10	Exocyst complex component 5
FUN_000288	contig_1	944768	945926	+		hypothetical protein
FUN_000289	contig_1	946480	947481	-		hypothetical protein
FUN_000290	contig_1	948016	949529	+		hypothetical protein
FUN_000291	contig_1	949812	950567	-		hypothetical protein
FUN_000292	contig_1	950765	951743	+	NSA2	Ribosome bioproteinsis protein

Gene ID	Scaffold	Start	Stop	Strand	Name	Product
FUN_000295	contig_1	956734	957639	-	ATP4	atp4 subunit B of the stator stalk of mitochondrial F1F0 ATP synthase
FUN_000296	contig_1	957847	959521	+	NOC4	Maturation and nuclear export of 40S ribosomal subunits interacting protein
FUN_000297	contig_1	962367	964005	+		hypothetical protein
FUN_000298	contig_1	967864	969918	-		hypothetical protein
FUN_000299	contig_1	973145	974932	-		hypothetical protein
FUN_000300	contig_1	975393	976691	+	PTC7	Protein phosphatase 2C 7
FUN_000301	contig_1	977331	979226	+		hypothetical protein
FUN_000302	contig_1	979712	981547	-		hypothetical protein
FUN_000304	contig_1	983526	985718	+		hypothetical protein
FUN_000305	contig_1	986520	987963	+		hypothetical protein
FUN_000306	contig_1	988965	990295	-		hypothetical protein
FUN_000307	contig_1	996287	997334	-	RPP0	ribosomal protein P0 (A0) (L10E)
FUN_000308	contig_1	997540	999274	+	TRM82	tRNA (guanine-N(7)-)-methyltransferase non-catalytic subunit trm82
FUN_000309	contig_1	1000328	1001360	+	TRR1	thioredoxin-disulfide reductase
FUN_000310	contig_1	1003485	1005145	+		hypothetical protein
FUN_000311	contig_1	1005791	1007082	+	CYS3	cystathionine gamma-lyase cys3
FUN_000312	contig_1	1007470	1010040	-	rad17	RFC checkpoint protein Rad17
FUN_000314	contig_1	1016575	1017432	-		hypothetical protein
FUN_000315	contig_1	1019180	1020632	-		hypothetical protein
FUN_000316	contig_1	1021188	1022592	+		hypothetical protein
FUN_000317	contig_1	1023910	1026122	+		hypothetical protein
FUN_000319	contig_1	1031511	1032915	-	TRM5	tRNA(m(1)G37)methyltransferase
FUN_000320	contig_1	1033287	1038196	+		hypothetical protein
FUN_000321	contig_1	1038742	1040709	+		hypothetical protein
FUN_000322	contig_1	1040977	1044018	-	TEL2	telomere binding protein
FUN_000323	contig_1	1044303	1045764	+	uba3	NEDD8 activating enzyme
FUN_000325	contig_1	1047231	1047419	+		hypothetical protein
FUN_000326	contig_1	1047795	1052105	+		hypothetical protein
FUN_000328	contig_1	1061876	1064029	+		hypothetical protein
FUN_000329	contig_1	1064293	1065061	-	TVP23	Golgi apparatus membrane protein tvp23
FUN_000330	contig_1	1065326	1066337	+		hypothetical protein
FUN_000331	contig_1	1066397	1067982	-		hypothetical protein
FUN_000332	contig_1	1068148	1069501	+	cys12	Cysteine synthase 2
FUN_000333	contig_1	1069959	1074075	+		hypothetical protein
FUN_000334	contig_1	1074429	1075777	-	ENV7	Serine/threonine-protein kinase env7
FUN_000335	contig_1	1076374	1079968	-		hypothetical protein
FUN_000336	contig_1	1080239	1081161	+		hypothetical protein
FUN_000337	contig_1	1081430	1082606	-	PPE1	Protein phosphatase methylesterase 1
FUN_000338	contig_1	1083223	1084885	+		hypothetical protein
FUN_000339	contig_1	1085286	1085978	-		hypothetical protein
FUN_000341	contig_1	1090534	1092174	-	PWP1	rRNA-processing protein
FUN_000342	contig_1	1092872	1095772	+		hypothetical protein
FUN_000343	contig_1	1098215	1100235	+		hypothetical protein
FUN_000344	contig_1	1100675	1102000	-		hypothetical protein
FUN_000346	contig_1	1103382	1104131	+		hypothetical protein
FUN_000347	contig_1	1105326	1105908	+		hypothetical protein
FUN_000348	contig_1	1106554	1107755	+		hypothetical protein
FUN_000349	contig_1	1108992	1109660	-		hypothetical protein
FUN_000351	contig_1	1113511	1113908	+	OAA1	Oxaloacetate tautomerase oaa1, mitochondrial
FUN_000353	contig_1	1115799	1118150	-		hypothetical protein
FUN_000354	contig_1	1119222	1120426	+	TVP38	Tlg2-vesicle protein
FUN_000355	contig_1	1122266	1125736	-	FUN30	DNA-dependent ATPase fun30
FUN_000356	contig_1	1126319	1126753	+	VMA21	vacuolar ATPase assembly integral membrane protein vma21
FUN_000357	contig_1	1127186	1128416	-		hypothetical protein
FUN_000358	contig_1	1130079	1131654	-	ALD6	aldehyde dehydrogenase (NADP(+)) ald6

Gene ID	Scaffold	Start	Stop	Strand	Name	Product
FUN_000359	contig_1	1132594	1134505	-		hypothetical protein
FUN_000360	contig_1	1137004	1138817	-	REG1_1	protein phosphatase regulator
FUN_000361	contig_1	1140884	1142833	+		hypothetical protein
FUN_000363	contig_1	1144613	1145078	+		hypothetical protein
FUN_000364	contig_1	1145493	1146696	+		hypothetical protein
FUN_000365	contig_1	1147642	1149683	+	GPT1	GABA/polyamine transporter
FUN_000366	contig_1	1150043	1151309	+		hypothetical protein
FUN_000367	contig_1	1151689	1153037	-		hypothetical protein
FUN_000368	contig_1	1155303	1156657	-		hypothetical protein
FUN_000370	contig_1	1160008	1160974	+		hypothetical protein
FUN_000371	contig_1	1163101	1163847	-		hypothetical protein
FUN_000372	contig_1	1169234	1170709	-		hypothetical protein
FUN_000373	contig_1	1175066	1176185	-		hypothetical protein
FUN_000374	contig_1	1176469	1178005	-	COQ6	Putative ubiquinone biosynthesis monooxygenase
FUN_000377	contig_1	1183166	1184012	+		hypothetical protein
FUN_000378	contig_1	1184599	1186208	+		hypothetical protein
FUN_000379	contig_1	1188372	1189272	-	GRX3	glutaredoxin
FUN_000380	contig_1	1189578	1190835	+	RPF2	rRNA-binding ribosome biosynthesis protein rpf2
FUN_000381	contig_1	1191005	1192051	-	mtd1	Methylenetetrahydrofolate dehydrogenase [NAD(+)]
FUN_000382	contig_1	1192386	1195780	+		hypothetical protein
FUN_000383	contig_1	1198818	1200683	+		hypothetical protein
FUN_000385	contig_1	1207149	1208132	+		hypothetical protein
FUN_000387	contig_1	1209711	1210988	-	OGG1	8-oxoguanine glycosylase ogg1
FUN_000388	contig_1	1211628	1213351	+		hypothetical protein
FUN_000389	contig_1	1214463	1215631	+		hypothetical protein
FUN_000390	contig_1	1218860	1220516	+		hypothetical protein
FUN_000391	contig_1	1224220	1225068	-		hypothetical protein
FUN_000393	contig_1	1227919	1229756	-		hypothetical protein
FUN_000395	contig_1	1233095	1234815	+		hypothetical protein
FUN_000396	contig_1	1235170	1235805	-		hypothetical protein
FUN_000399	contig_1	1241239	1242197	+		hypothetical protein
FUN_000400	contig_1	1247792	1249225	-		hypothetical protein
FUN_000402	contig_1	1250970	1252402	-		hypothetical protein
FUN_000403	contig_1	1253298	1255145	+		hypothetical protein
FUN_000405	contig_1	1257075	1258387	-		hypothetical protein
FUN_000406	contig_1	1259482	1260641	+		hypothetical protein
FUN_000407	contig_1	1260836	1262275	-	AIM9	Phosphotransferase enzyme
FUN_000409	contig_1	1265533	1266747	-		hypothetical protein
FUN_000410	contig_1	1267747	1268673	+		hypothetical protein
FUN_000411	contig_1	1268812	1269973	-		hypothetical protein
FUN_000412	contig_1	1270827	1271688	+		hypothetical protein
FUN_000413	contig_1	1272068	1273407	-		hypothetical protein
FUN_000414	contig_1	1273904	1278421	+		hypothetical protein
FUN_000415	contig_1	1279437	1282130	+		hypothetical protein
FUN_000416	contig_1	1283016	1284248	-		hypothetical protein
FUN_000417	contig_1	1284305	1284827	-		hypothetical protein
FUN_000418	contig_1	1285353	1287256	-		hypothetical protein
FUN_000419	contig_1	1287689	1289832	+		hypothetical protein
FUN_000420	contig_1	1290192	1291277	+		hypothetical protein
FUN_000421	contig_1	1291860	1293432	+		hypothetical protein
FUN_000422	contig_1	1294664	1295557	+		hypothetical protein
FUN_000423	contig_1	1296238	1297894	+		hypothetical protein
FUN_000424	contig_1	1298256	1299309	+		hypothetical protein
FUN_000425	contig_1	1304867	1306170	-		hypothetical protein
FUN_000427	contig_1	1316831	1319482	-		hypothetical protein
FUN_000428	contig_1	1320709	1321977	+	RPE1	RIBULOSE-phosphate 3-epimerase
FUN_000430	contig_1	1327219	1328999	-		hypothetical protein
FUN_000435	contig_1	1347546	1348403	-		hypothetical protein
FUN_000436	contig_1	1349612	1353398	-	rga1	Rho-type GTPase activating protein Rga1

Gene ID	Scaffold	Start	Stop	Strand	Name	Product
FUN_000437	contig_1	1354460	1355056	+	RPB4	RNA polymerase B
FUN_000438	contig_1	1355753	1357872	-		hypothetical protein
FUN_000439	contig_1	1359002	1360156	-		hypothetical protein
FUN_000441	contig_1	1363501	1364679	-		hypothetical protein
FUN_000442	contig_1	1365078	1366567	-		hypothetical protein
FUN_000443	contig_1	1368495	1370037	+		hypothetical protein
FUN_000444	contig_1	1373431	1377471	-		hypothetical protein
FUN_000445	contig_1	1380224	1382818	+	pso2	DNA cross-link repair protein PSO2/SNM1
FUN_000446	contig_1	1385438	1386844	-		hypothetical protein
FUN_000447	contig_1	1387544	1388828	-		hypothetical protein
FUN_000449	contig_1	1393850	1395114	-		hypothetical protein
FUN_000450	contig_1	1403550	1406267	-		hypothetical protein
FUN_000451	contig_1	1407395	1407888	-	rpc25	DNA-directed RNA polymerase III complex subunit Rpc25
FUN_000452	contig_1	1408479	1410125	+		hypothetical protein
FUN_000454	contig_1	1412937	1413551	+		hypothetical protein
FUN_000456	contig_1	1421819	1423640	-	SNF3_1	Plasma membrane low glucose sensor
FUN_000457	contig_1	1426891	1428308	-	BUD16	Putative pyridoxal kinase
FUN_000458	contig_1	1428874	1429630	-	POL30	proliferating cell nuclear antigen
FUN_000459	contig_1	1434407	1436410	+		hypothetical protein
FUN_000460	contig_1	1457749	1459380	-		hypothetical protein
FUN_000462	contig_1	1464823	1467410	-		hypothetical protein
FUN_000463	contig_1	1467810	1468182	+		hypothetical protein
FUN_000464	contig_1	1468317	1470423	-		hypothetical protein
FUN_000465	contig_1	1470651	1472485	-		hypothetical protein
FUN_000468	contig_1	1478125	1478838	+		hypothetical protein
FUN_000469	contig_1	1479099	1479807	-		hypothetical protein
FUN_000470	contig_1	1480952	1485408	-	AMD1	AMP deaminase
FUN_000471	contig_1	1485677	1487288	+		hypothetical protein
FUN_000472	contig_1	1488028	1490040	+	PEX5	Peroxisomal membrane signal receptor PTS1
FUN_000473	contig_1	1490768	1491604	+		hypothetical protein
FUN_000474	contig_1	1491858	1493058	-		hypothetical protein
FUN_000476	contig_1	1494734	1497198	-	PMT4_1	Dolichyl-phosphate-mannose--protein mannosyltransferase 4
FUN_000477	contig_1	1497727	1498566	-	PMT4_2	Dolichyl-phosphate-mannose--protein mannosyltransferase 4
FUN_000478	contig_1	1499694	1501501	-	CDC55	protein phosphatase 2A regulatory subunit cdc55
FUN_000479	contig_1	1502006	1503344	+	RPL3	60S ribosomal protein L3
FUN_000480	contig_1	1504691	1506349	+	SDH1_1	succinate dehydrogenase flavoprotein subunit
FUN_000481	contig_1	1506885	1507850	-		hypothetical protein
FUN_000482	contig_1	1508734	1512340	-		hypothetical protein
FUN_000484	contig_1	1513582	1515405	-		hypothetical protein
FUN_000485	contig_1	1516367	1518472	-		hypothetical protein
FUN_000487	contig_1	1522978	1526393	+		hypothetical protein
FUN_000488	contig_1	1530396	1532252	+	RLM1	Transcription factor rlm1
FUN_000489	contig_1	1533448	1534699	+		hypothetical protein
FUN_000490	contig_1	1537952	1538899	-		hypothetical protein
FUN_000493	contig_1	1546598	1548833	+		hypothetical protein
FUN_000495	contig_1	1554008	1555810	-	CCT4	T-complex protein 1 subunit delta
FUN_000496	contig_1	1556134	1557613	+	RPT1	26S proteasome regulatory subunit 7
FUN_000497	contig_1	1558081	1560108	-	RRN3	DNA independent RNA polymerase I transcription factor
FUN_000498	contig_1	1560601	1561604	-	CYP3	Peptidyl-prolyl cis-trans isomerase H
FUN_000499	contig_1	1562882	1563786	+		hypothetical protein
FUN_000501	contig_1	1567288	1570205	+		hypothetical protein
FUN_000503	contig_1	1572268	1575443	+		hypothetical protein
FUN_000504	contig_1	1578606	1579703	-		hypothetical protein
FUN_000506	contig_1	1591317	1596059	-		hypothetical protein
FUN_000507	contig_1	1596689	1599369	+	ABP1	actin binding protein
FUN_000508	contig_1	1601941	1602807	+		hypothetical protein

Gene ID	Scaffold	Start	Stop	Strand	Name	Product
FUN_000509	contig_1	1603321	1604355	+		hypothetical protein
FUN_000513	contig_1	1609701	1610960	+		hypothetical protein
FUN_000514	contig_1	1612084	1615258	+	RAD53_1	Protein kinase protein rad53
FUN_000515	contig_1	1615784	1617435	+	YOS9_1	Protein OS-9
FUN_000516	contig_1	1617572	1618801	-		hypothetical protein
FUN_000517	contig_1	1619297	1619958	-		hypothetical protein
FUN_000518	contig_1	1620482	1621192	-	JAC1	molecular chaperone
FUN_000522	contig_1	1630115	1633312	-		hypothetical protein
FUN_000523	contig_1	1635254	1637732	-	STT3	oligosaccharyl transferase stt3 subunit
FUN_000524	contig_1	1640990	1642537	-		hypothetical protein
FUN_000525	contig_1	1644431	1645479	-		hypothetical protein
FUN_000526	contig_1	1645740	1646528	+		hypothetical protein
FUN_000528	contig_1	1652136	1656017	+		hypothetical protein
FUN_000529	contig_1	1658012	1660581	+		hypothetical protein
FUN_000533	contig_1	1666145	1667639	+		hypothetical protein
FUN_000534	contig_1	1668362	1669946	+	SEC9	Protein transport protein S9 plasma membrane t-SNARE
FUN_000535	contig_1	1670372	1673773	-	AMS1	Glycoside hydrolase, 38 vacuolar alpha mannosidase
FUN_000537	contig_1	1676035	1681907	-	RIM15	rim15, signal transduction response regulator
FUN_000538	contig_1	1683806	1684790	-		hypothetical protein
FUN_000539	contig_1	1685676	1687238	-		hypothetical protein
FUN_000542	contig_1	1698162	1700744	-	DNM1	Dynamin-related GTPase protein
FUN_000543	contig_1	1701563	1702417	+		hypothetical protein
FUN_000544	contig_1	1702925	1703173	-	TIM11	F1F0 ATP synthase subunit e, mitochondrial
FUN_000545	contig_1	1703844	1706067	+		hypothetical protein
FUN_000546	contig_1	1708774	1709463	-		hypothetical protein
FUN_000548	contig_1	1712098	1713344	-		hypothetical protein
FUN_000549	contig_1	1715471	1716758	+		hypothetical protein
FUN_000550	contig_1	1718441	1723000	+	VPS10	vacuolar protein sorting/targeting protein PEP1
FUN_000551	contig_1	1727096	1728496	+		hypothetical protein
FUN_000552	contig_1	1729833	1733932	-		hypothetical protein
FUN_000554	contig_1	1737826	1739442	-		hypothetical protein
FUN_000555	contig_1	1742720	1744433	-	NOP12	Nucleolar protein 12
FUN_000556	contig_1	1745372	1747588	+		hypothetical protein
FUN_000557	contig_1	1748690	1750709	+		hypothetical protein
FUN_000558	contig_1	1751460	1751997	+		hypothetical protein
FUN_000559	contig_1	1765862	1766935	-	HAP2	Transcriptional activator
FUN_000561	contig_1	1772619	1773838	+		hypothetical protein
FUN_000562	contig_1	1774172	1775668	+		hypothetical protein
FUN_000563	contig_1	1776446	1780299	+	SLA1	cytoskeletal protein binding protein
FUN_000564	contig_1	1781146	1784263	-		hypothetical protein
FUN_000565	contig_1	1786575	1788914	-		hypothetical protein
FUN_000566	contig_1	1789945	1794273	+	PEX6	peroxisomal assembly protein
FUN_000568	contig_1	1796678	1797718	-		hypothetical protein
FUN_000571	contig_1	1806997	1808013	-		hypothetical protein
FUN_000572	contig_1	1808712	1810275	-		hypothetical protein
FUN_000574	contig_1	1814768	1816425	-		hypothetical protein
FUN_000575	contig_1	1817000	1817966	-		hypothetical protein
FUN_000576	contig_1	1818093	1819978	-		hypothetical protein
FUN_000577	contig_1	1821239	1822277	-		hypothetical protein
FUN_000578	contig_1	1822843	1824442	-		hypothetical protein
FUN_000579	contig_1	1824745	1826475	-		hypothetical protein
FUN_000580	contig_1	1827130	1828554	-		hypothetical protein
FUN_000581	contig_1	1828944	1830219	-		hypothetical protein
FUN_000582	contig_1	1830494	1831882	+		hypothetical protein
FUN_000583	contig_1	1832561	1834227	+		hypothetical protein
FUN_000584	contig_1	1834392	1835387	-		hypothetical protein
FUN_000585	contig_1	1835998	1837022	+		hypothetical protein
FUN_000586	contig_1	1837080	1838127	-		hypothetical protein
FUN_000587	contig_1	1838478	1840297	-		hypothetical protein

Gene ID	Scaffold	Start	Stop	Strand	Name	Product
FUN_000588	contig_1	1840723	1841816	+		hypothetical protein
FUN_000589	contig_1	1842363	1844199	+		hypothetical protein
FUN_000590	contig_1	1844840	1847418	+		hypothetical protein
FUN_000592	contig_1	1850690	1852366	-		hypothetical protein
FUN_000593	contig_1	1853058	1855132	-	NUA3	putative component of NuA3 histone acetyltransferase complex
FUN_000594	contig_1	1855593	1856649	-	TPC1	mitochondrial thiamine pyrophosphate transporter
FUN_000595	contig_1	1856884	1858308	+		hypothetical protein
FUN_000596	contig_1	1859178	1860644	+		hypothetical protein
FUN_000597	contig_1	1860884	1862117	-		hypothetical protein
FUN_000598	contig_1	1862523	1864132	+		hypothetical protein
FUN_000599	contig_1	1866331	1868016	-		hypothetical protein
FUN_000600	contig_1	1868421	1869588	-	ERG26_1	erg26, C-3 sterol dehydrogenase
FUN_000601	contig_1	1870193	1871851	-		hypothetical protein
FUN_000602	contig_1	1872579	1872786	+	TRAPPC4	Trafficking protein particle complex subunit 4
FUN_000603	contig_1	1873231	1874319	-	RPN10	proteasome regulatory particle base subunit rpn10
FUN_000604	contig_1	1874686	1875261	+		hypothetical protein
FUN_000605	contig_1	1876924	1879341	+		hypothetical protein
FUN_000606	contig_1	1880164	1881824	+		hypothetical protein
FUN_000608	contig_1	1885700	1887852	+	IZH1	minc metabolism membrane protein
FUN_000609	contig_1	1891079	1892797	-		hypothetical protein
FUN_000611	contig_1	1899994	1901656	-	NSR1	nuclear localization sequence binding protein
FUN_000612	contig_1	1902570	1904381	+	ALG6	Glucosyltransferase-like protein
FUN_000613	contig_1	1905180	1906502	+	rna1	Ran GAP Rna1
FUN_000615	contig_1	1910308	1913294	+		hypothetical protein
FUN_000616	contig_1	1913958	1915733	+	TUB1_1	alpha-tubulin
FUN_000617	contig_1	1916587	1917859	+		hypothetical protein
FUN_000618	contig_1	1919011	1922154	+	DPS1	aspartate--tRNA ligase dps1
FUN_000619	contig_1	1923289	1924888	+		hypothetical protein
FUN_000621	contig_1	1928003	1930615	+		hypothetical protein
FUN_000623	contig_1	1934275	1938024	-		hypothetical protein
FUN_000624	contig_1	1938659	1939704	-		hypothetical protein
FUN_000625	contig_1	1940146	1941109	+		hypothetical protein
FUN_000626	contig_1	1942413	1943687	+		hypothetical protein
FUN_000627	contig_1	1944314	1946346	+		hypothetical protein
FUN_000629	contig_1	1947581	1949691	-		hypothetical protein
FUN_000630	contig_1	1951316	1955155	-		hypothetical protein
FUN_000631	contig_1	1955939	1957150	-		hypothetical protein
FUN_000634	contig_1	1961407	1964974	-		hypothetical protein
FUN_000635	contig_1	1965900	1967249	-		hypothetical protein
FUN_000636	contig_1	1967761	1969471	+		hypothetical protein
FUN_000637	contig_1	1969812	1970912	-		hypothetical protein
FUN_000638	contig_1	1972020	1973235	+		hypothetical protein
FUN_000639	contig_1	1974005	1977774	+		hypothetical protein
FUN_000640	contig_1	1983703	1985488	-		hypothetical protein
FUN_000643	contig_1	1991323	1993123	-		hypothetical protein
FUN_000644	contig_1	1994333	1995691	+		hypothetical protein
FUN_000645	contig_1	1996246	1997364	-	PRN1	RNA pol II transcription cofactor
FUN_000648	contig_1	2013353	2014303	-		hypothetical protein
FUN_000649	contig_1	2015348	2016751	-		hypothetical protein
FUN_000651	contig_1	2019240	2023415	-		hypothetical protein
FUN_000652	contig_1	2026701	2027702	+		hypothetical protein
FUN_000654	contig_1	2032099	2034580	+		hypothetical protein
FUN_000655	contig_1	2035812	2036225	+		hypothetical protein
FUN_000656	contig_1	2037004	2037892	-		hypothetical protein
FUN_000657	contig_1	2038963	2039859	-		hypothetical protein
FUN_000658	contig_1	2042329	2042870	+		hypothetical protein
FUN_000659	contig_1	2042959	2044729	+		hypothetical protein
FUN_000660	contig_1	2045266	2046362	-		hypothetical protein

Gene ID	Scaffold	Start	Stop	Strand	Name	Product
FUN_000661	contig_1	2047952	2049803	-		hypothetical protein
FUN_000662	contig_1	2050864	2052543	+		hypothetical protein
FUN_000665	contig_1	2058371	2059873	-		hypothetical protein
FUN_000667	contig_1	2081129	2081993	+		hypothetical protein
FUN_000669	contig_1	2086567	2087217	+		hypothetical protein
FUN_000671	contig_1	2091375	2096623	-	dna2	DNA replication endonuclease-helicase Dna2
FUN_000672	contig_1	2097676	2098455	+		hypothetical protein
FUN_000673	contig_1	2098962	2100127	-		hypothetical protein
FUN_000674	contig_1	2100405	2102443	+	SAS10	something about silencing protein 10
FUN_000675	contig_1	2104873	2105481	-		hypothetical protein
FUN_000676	contig_1	2106072	2107739	-	AMT1	ammonium transporter Amt1
FUN_000677	contig_1	2111294	2115429	+	SSK2	Suppressor of Sensor Kinase (SLN1)
FUN_000678	contig_1	2115991	2117955	+	HTS1	Cytoplasmic and mitochondrial histidine tRNA synthetase
FUN_000679	contig_1	2118999	2121519	-	trl1	tRNA ligase
FUN_000680	contig_1	2122493	2124479	-		hypothetical protein
FUN_000681	contig_1	2128184	2128672	-	SFT2	Protein transport protein sft2
FUN_000682	contig_1	2131825	2133552	+		hypothetical protein
FUN_000684	contig_1	2139696	2141069	-	OYE32_1	NADH-dependent flavin oxidoreductase
FUN_000685	contig_1	2142761	2143817	+	CKB2	casein kinase 2 regulatory subunit
FUN_000688	contig_1	2158304	2160913	-		hypothetical protein
FUN_000689	contig_1	2161376	2162888	+		hypothetical protein
FUN_000690	contig_1	2163719	2164894	+		hypothetical protein
FUN_000691	contig_1	2165097	2166123	-		hypothetical protein
FUN_000692	contig_1	2167958	2168842	+		hypothetical protein
FUN_000693	contig_1	2171019	2172310	-		hypothetical protein
FUN_000694	contig_1	2172656	2175574	-		hypothetical protein
FUN_000695	contig_1	2176746	2179626	-		hypothetical protein
FUN_000696	contig_1	2180809	2181966	-	RML2	mitochondrial 54S ribosomal protein rml2
FUN_000697	contig_1	2182869	2184516	+	CLB2	G2/mitotic-specific cyclin
FUN_000698	contig_1	2187087	2188118	-		hypothetical protein
FUN_000699	contig_1	2188665	2190303	+		hypothetical protein
FUN_000700	contig_1	2190517	2191802	-		hypothetical protein
FUN_000701	contig_1	2193002	2196891	+		hypothetical protein
FUN_000702	contig_1	2197286	2198360	+		hypothetical protein
FUN_000704	contig_1	2202303	2204745	-		hypothetical protein
FUN_000705	contig_1	2206053	2208323	-		hypothetical protein
FUN_000706	contig_1	2212366	2213264	-		hypothetical protein
FUN_000707	contig_1	2213553	2213904	-		hypothetical protein
FUN_000708	contig_1	2214140	2215212	+		hypothetical protein
FUN_000709	contig_1	2215403	2217467	-		hypothetical protein
FUN_000710	contig_1	2222383	2223975	+		hypothetical protein
FUN_000711	contig_1	2225933	2227642	-	PRP31	U4/U6-U5 snRNP complex subunit prp31
FUN_000712	contig_1	2228099	2230168	+	CLF1	NineTeen Complex (NTC) component
FUN_000713	contig_1	2232624	2233707	+	GOR1	glyoxylate reductase
FUN_000714	contig_1	2237075	2239190	+		hypothetical protein
FUN_000715	contig_1	2242219	2243427	-		hypothetical protein
FUN_000718	contig_1	2247348	2247856	-		hypothetical protein
FUN_000719	contig_1	2249994	2251096	-	SSN8	RNA polymerase II holoenzyme cyclin-like subunit
FUN_000720	contig_1	2251724	2253951	+		hypothetical protein
FUN_000721	contig_1	2254721	2255296	-	hsp10	mitochondrial heat shock protein Hsp10
FUN_000722	contig_1	2255838	2257661	+		hypothetical protein
FUN_000723	contig_1	2258134	2259966	-		hypothetical protein
FUN_000724	contig_1	2260436	2261268	+		hypothetical protein
FUN_000725	contig_1	2261488	2264148	-		hypothetical protein
FUN_000726	contig_1	2264853	2267802	+		hypothetical protein
FUN_000727	contig_1	2268630	2269483	-		hypothetical protein
FUN_000728	contig_1	2270532	2272703	-		hypothetical protein
FUN_000730	contig_1	2285021	2286211	+		hypothetical protein
FUN_000731	contig_1	2286654	2290106	+	RIC1	WD40 repeat protein

Gene ID	Scaffold	Start	Stop	Strand	Name	Product
FUN_000733	contig_1	2294689	2295051	-		hypothetical protein
FUN_000734	contig_1	2295887	2298286	-		hypothetical protein
FUN_000735	contig_1	2298847	2302126	-	HIR1	HIR complex subunit
FUN_000736	contig_1	2302674	2303639	-		hypothetical protein
FUN_000739	contig_1	2319003	2321315	+	puf6	Pumilio y domain member 6
FUN_000740	contig_1	2321680	2322366	-		hypothetical protein
FUN_000741	contig_1	2323049	2324303	+		hypothetical protein
FUN_000742	contig_1	2324932	2326120	+	RHP51	RecA recombinase Rhp51
FUN_000743	contig_1	2326491	2330229	+		hypothetical protein
FUN_000744	contig_1	2330663	2331937	+		hypothetical protein
FUN_000746	contig_1	2334908	2338345	-		hypothetical protein
FUN_000747	contig_1	2339048	2339899	+		hypothetical protein
FUN_000748	contig_1	2340844	2342996	-		hypothetical protein
FUN_000750	contig_1	2346338	2348293	+	THR4	threonine synthase
FUN_000751	contig_1	2350377	2354181	-	PKC1_1	Serine/threonine kinase
FUN_000754	contig_1	2361957	2362981	-		hypothetical protein
FUN_000755	contig_1	2363446	2364422	+		hypothetical protein
FUN_000756	contig_1	2365049	2367003	-		hypothetical protein
FUN_000757	contig_1	2368816	2372205	-		hypothetical protein
FUN_000758	contig_1	2373375	2374110	-	PSF1	DNA replication protein psf1
FUN_000759	contig_1	2374531	2376050	-		hypothetical protein
FUN_000760	contig_1	2380413	2381983	+		hypothetical protein
FUN_000762	contig_1	2385052	2388290	+		hypothetical protein
FUN_000763	contig_1	2388758	2390519	-	TGL3	triacylglycerol lipase
FUN_000764	contig_1	2390942	2391343	+		hypothetical protein
FUN_000765	contig_1	2391780	2393375	-		hypothetical protein
FUN_000766	contig_1	2395619	2399775	+	SSD1	Translational repressor
FUN_000767	contig_1	2401682	2402765	+		hypothetical protein
FUN_000768	contig_1	2403808	2405145	+		hypothetical protein
FUN_000769	contig_1	2405834	2407112	-		hypothetical protein
FUN_000772	contig_1	2412273	2413108	+		hypothetical protein
FUN_000773	contig_1	2413674	2415602	-	acl2	beta subunit of ATP citrate lyase
FUN_000774	contig_1	2416743	2418971	+	acl1	ATP citrate lyase subunit 1
FUN_000775	contig_1	2420223	2422084	+		hypothetical protein
FUN_000777	contig_1	2424704	2426680	+	FAT1_1	long-chain fatty acid transporter fat1
FUN_000779	contig_1	2429193	2430302	-		hypothetical protein
FUN_000781	contig_1	2435575	2437267	-	ALD5	aldehyde dehydrogenase (NAD(P)(+)) ald5
FUN_000782	contig_1	2438497	2439680	-	YRA1	RNA-binding RNA annealing protein
FUN_000783	contig_1	2440208	2443337	+	VPS11	Vacuolar protein sorting-associated protein 11
FUN_000785	contig_1	2447849	2449338	-		hypothetical protein
FUN_000786	contig_1	2451033	2454940	+	TRM8_1	tRNA (guanine-N(7)-)-methyltransferase (tRNA(m7G46)-methyltransferase)
FUN_000787	contig_1	2455611	2456713	+		hypothetical protein
FUN_000788	contig_1	2456844	2458213	-		hypothetical protein
FUN_000789	contig_1	2459891	2460795	-		hypothetical protein
FUN_000790	contig_1	2461607	2462596	-		hypothetical protein
FUN_000791	contig_1	2463750	2464799	+	SEC13	GTPase-activating protein S13
FUN_000793	contig_1	2468824	2470362	-		hypothetical protein
FUN_000795	contig_1	2477228	2479672	+	GCN20_1	ATP-binding cassette, regulator of translational elongation
FUN_000796	contig_1	2480062	2481910	+	GCN20_2	ATP-binding cassette, regulator of translational elongation
FUN_000797	contig_1	2483767	2485177	-	SAH1	S-adenosyl-L-homocysteine hydrolase
FUN_000798	contig_1	2486515	2487671	+		hypothetical protein
FUN_000799	contig_1	2492220	2494092	+	SFH1	Chromatin structure remodeling complex protein sfh1
FUN_000802	contig_1	2500170	2501341	+	ERV29	ER-derived vesicles protein erv29
FUN_000803	contig_1	2502629	2503694	+		hypothetical protein
FUN_000804	contig_1	2504864	2506692	-		hypothetical protein
FUN_000805	contig_1	2509084	2509712	-		hypothetical protein

Gene ID	Scaffold	Start	Stop	Strand	Name	Product
FUN_000806	contig_1	2512747	2514222	+	PRP46	pre-mRNA-splicing factor prp46
FUN_000807	contig_1	2517363	2520007	-	ORC1	Origin recognition complex, subunit 1
FUN_000808	contig_1	2520742	2521553	-		hypothetical protein
FUN_000809	contig_1	2535939	2538815	-		hypothetical protein
FUN_000812	contig_1	2543434	2544447	+	BGL2	glycoside hydrolase 3 protein
FUN_000813	contig_1	2545068	2546400	+		hypothetical protein
FUN_000814	contig_1	2546777	2548764	-		hypothetical protein
FUN_000815	contig_1	2550246	2552938	-		hypothetical protein
FUN_000816	contig_1	2554150	2560663	+	CCH1	calcium channel protein
FUN_000817	contig_1	2565753	2567177	-		hypothetical protein
FUN_000818	contig_1	2569046	2569767	+		hypothetical protein
FUN_000822	contig_1	2575776	2576894	+		hypothetical protein
FUN_000823	contig_1	2576983	2577972	-		hypothetical protein
FUN_000824	contig_1	2578274	2580377	-		hypothetical protein
FUN_000827	contig_1	2588829	2589776	-		hypothetical protein
FUN_000830	contig_1	2597682	2598990	-	IMP4	snoRNA-binding rRNA-processing protein imp4
FUN_000831	contig_1	2600590	2603001	-	PRP43	DEAH-box ATP-dependent RNA helicase prp43
FUN_000832	contig_1	2603359	2605907	+		hypothetical protein
FUN_000834	contig_1	2616739	2618070	+		hypothetical protein
FUN_000835	contig_1	2619283	2620973	+		hypothetical protein
FUN_000837	contig_1	2626903	2628439	-		hypothetical protein
FUN_000839	contig_1	2631205	2632585	-	VMA5	Vacuolar ATP synthase subunit C
FUN_000840	contig_1	2632812	2634233	+	GCV1	Aminomethyltransferase, mitochondrial
FUN_000841	contig_1	2634852	2635634	+		hypothetical protein
FUN_000842	contig_1	2640885	2641886	+		hypothetical protein
FUN_000843	contig_1	2642048	2643406	-		hypothetical protein
FUN_000845	contig_1	2646757	2647467	-		hypothetical protein
FUN_000846	contig_1	2649050	2649921	+		hypothetical protein
FUN_000847	contig_1	2650190	2652208	-	UBA2_1	E1 ubiquitin-activating protein uba2
FUN_000848	contig_1	2652589	2653839	+		hypothetical protein
FUN_000849	contig_1	2654125	2654890	+		hypothetical protein
FUN_000850	contig_1	2655174	2656763	-		hypothetical protein
FUN_000852	contig_1	2659885	2665289	-		hypothetical protein
FUN_000853	contig_1	2672907	2674243	-	BCD1	Box C/D snoRNA accumulation
FUN_000854	contig_1	2676319	2679212	+		hypothetical protein
FUN_000855	contig_1	2680914	2682095	+		hypothetical protein
FUN_000856	contig_1	2682667	2684982	+		hypothetical protein
FUN_000858	contig_1	2686808	2688323	-		hypothetical protein
FUN_000859	contig_1	2689945	2692562	-		hypothetical protein
FUN_000860	contig_1	2692759	2696363	+		hypothetical protein
FUN_000861	contig_1	2696883	2698706	-	MCH1	Putative monocarboxylate transporter mch1
FUN_000862	contig_1	2699103	2700259	+	RRP42	Exosome complex component rrp42
FUN_000866	contig_1	2709483	2714746	-		hypothetical protein
FUN_000867	contig_1	2721977	2723719	-		hypothetical protein
FUN_000868	contig_1	2724092	2725280	+	LEU5	coenzyme A transporter
FUN_000870	contig_1	2729807	2731343	-	SIT4_1	sporulation-induced protein
FUN_000871	contig_1	2732009	2733894	+		hypothetical protein
FUN_000872	contig_1	2741936	2744387	+	IME2	Serine/threonine protein kinase
FUN_000873	contig_1	2746381	2748296	-	NAR1	Cytosolic Fe-S cluster assembly factor nar1
FUN_000874	contig_1	2748745	2750252	+		hypothetical protein
FUN_000875	contig_1	2750947	2751865	-	NIT2	Carbon-nitrogen hydrolase
FUN_000876	contig_1	2752832	2755593	+		hypothetical protein
FUN_000877	contig_1	2755777	2756597	-	NAS6_1	putative ankyrin-repeat protein
FUN_000879	contig_1	2760385	2762340	+	MDR1	GTPase-activating protein
FUN_000880	contig_1	2763624	2765447	-		hypothetical protein
FUN_000881	contig_1	2767004	2768390	-		hypothetical protein
FUN_000882	contig_1	2769272	2771632	-	ERB1	Ribosome bioproteinsis protein erb1
FUN_000883	contig_1	2771930	2775481	+	PRP5	pre-mRNA processing RNA-helicase
FUN_000884	contig_1	2776268	2777726	-		hypothetical protein

Gene ID	Scaffold	Start	Stop	Strand	Name	Product
FUN_000885	contig_1	2777955	2778788	-		hypothetical protein
FUN_000886	contig_1	2779554	2781436	-		hypothetical protein
FUN_000887	contig_1	2782691	2783831	-	vps26	Vacuolar protein sorting-associated protein 26
FUN_000888	contig_1	2785126	2785873	+		hypothetical protein
FUN_000889	contig_1	2786585	2788248	+		hypothetical protein
FUN_000890	contig_1	2788453	2789253	+		hypothetical protein
FUN_000891	contig_1	2789605	2792275	-	ESC4	regulator of Ty1 Transposition
FUN_000892	contig_1	2793376	2794418	+		hypothetical protein
FUN_000893	contig_1	2803697	2804164	-		hypothetical protein
FUN_000894	contig_1	2811191	2814256	-	DIS3	exosome catalytic subunit dis3
FUN_000895	contig_1	2814700	2816249	+	VMA13	H(+)-transporting V1 sector ATPase subunit H
FUN_000896	contig_1	2820365	2822595	-		hypothetical protein
FUN_000897	contig_1	2827201	2827960	-	sap62	CWF complex protein sap62
FUN_000898	contig_1	2828264	2830082	+		hypothetical protein
FUN_000901	contig_1	2834247	2834853	+		hypothetical protein
FUN_000902	contig_1	2835429	2836009	+		hypothetical protein
FUN_000903	contig_1	2836619	2837735	-		hypothetical protein
FUN_000904	contig_1	2839049	2839936	-		hypothetical protein
FUN_000908	contig_1	2846106	2846949	-		hypothetical protein
FUN_000909	contig_1	2848081	2851307	+		hypothetical protein
FUN_000910	contig_1	2853066	2855177	+	CBK1	Serine/threonine-protein kinase
FUN_000911	contig_1	2857997	2858689	+		hypothetical protein
FUN_000913	contig_1	2863273	2867861	-	CDR1_1	Multidrug resistance protein
FUN_000915	contig_1	2873250	2874070	+		hypothetical protein
FUN_000916	contig_1	2885320	2886537	-		hypothetical protein
FUN_000917	contig_1	2894645	2897257	-		hypothetical protein
FUN_000918	contig_1	2898265	2900340	-		hypothetical protein
FUN_000919	contig_1	2901360	2902541	+		hypothetical protein
FUN_000920	contig_1	2902818	2903415	+		hypothetical protein
FUN_000921	contig_1	2904033	2905044	-		hypothetical protein
FUN_000925	contig_1	2915854	2917097	+		hypothetical protein
FUN_000929	contig_1	2923480	2925544	-	SUR1	zinc-finger protein
FUN_000931	contig_1	2927079	2929736	-		hypothetical protein
FUN_000932	contig_1	2930315	2931647	-		hypothetical protein
FUN_000933	contig_1	2932140	2933398	+		hypothetical protein
FUN_000935	contig_1	2936276	2938667	-		hypothetical protein
FUN_000936	contig_1	2939125	2940078	+	ATP10	Mitochondrial ATPase complex subunit atp10
FUN_000937	contig_1	2940306	2941026	-	GOS1	protein transport protein gos1
FUN_000938	contig_1	2941358	2942048	+	rpl35	60S ribosomal protein L35, L29
FUN_000939	contig_1	2942449	2944148	-	ERG8	phosphomevalonate kinase
FUN_000940	contig_1	2945183	2949064	+		hypothetical protein
FUN_000942	contig_1	2955370	2957062	+	SIR2	NAD-dependent histone deacetylase sir2
FUN_000943	contig_1	2957672	2958868	+		hypothetical protein
FUN_000945	contig_1	2966772	2967493	+	lcc1_1	laccase, multicopper oxidase, benzenediol:oxygen oxidorectuctase
FUN_000947	contig_1	2969509	2971146	-		hypothetical protein
FUN_000948	contig_1	2971756	2974600	-	cdc17	ATP-dependent DNA ligase Cdc17
FUN_000949	contig_1	2975087	2975719	+		hypothetical protein
FUN_000950	contig_1	2976860	2977710	-	RPL10A	60S ribosomal protein L10A
FUN_000951	contig_1	2978375	2979663	-	rcd1	RNA-binding protein, CCR4-NOT complex subunit Rcd1
FUN_000952	contig_1	2980357	2983163	-	KIN2	Serine/threonine-protein kinase
FUN_000953	contig_1	2984560	2986713	+		hypothetical protein
FUN_000954	contig_1	2988809	2989065	+		hypothetical protein
FUN_000955	contig_1	2989802	2990650	+		hypothetical protein
FUN_000957	contig_1	2992306	2993629	-		hypothetical protein
FUN_000958	contig_1	2994072	2995145	-		hypothetical protein
FUN_000959	contig_1	2996103	2996759	-		hypothetical protein
FUN_000960	contig_1	2997921	3000392	-		hypothetical protein
FUN_000962	contig_1	3004376	3005112	+	ARF1	Arf GTPase arf1

Gene ID	Scaffold	Start	Stop	Strand	Name	Product
FUN_000963	contig_1	3005803	3006761	-		hypothetical protein
FUN_000964	contig_1	3007577	3009055	+	LEO1	Paf1 complex component
FUN_000965	contig_1	3009332	3010810	-		hypothetical protein
FUN_000966	contig_1	3011335	3012680	+		hypothetical protein
FUN_000967	contig_1	3013426	3015074	+		hypothetical protein
FUN_000968	contig_1	3015701	3016202	-		hypothetical protein
FUN_000969	contig_1	3017407	3018811	+		hypothetical protein
FUN_000970	contig_1	3019954	3020850	-	SOD4_1	Cell surface superoxide dismutase [Cu-Zn] 4
FUN_000972	contig_1	3026067	3027455	+		hypothetical protein
FUN_000973	contig_1	3032979	3035000	-		hypothetical protein
FUN_000975	contig_1	3039114	3039943	+		hypothetical protein
FUN_000976	contig_1	3040306	3040932	-		hypothetical protein
FUN_000977	contig_1	3042051	3043582	-		hypothetical protein
FUN_000978	contig_1	3045154	3046559	-		hypothetical protein
FUN_000979	contig_1	3047076	3047547	-		hypothetical protein
FUN_000980	contig_1	3049822	3051539	+	TUB2	Tubulin beta chain (Beta tubulin)
FUN_000981	contig_1	3052104	3052988	-	OPI3	Phosphatidyl-N-methylethanolamine N-methyltransferase
FUN_000982	contig_1	3053506	3055524	-		hypothetical protein
FUN_000983	contig_1	3056793	3059752	-	ppk32	Protein kinase domain-containing protein ppk32
FUN_000984	contig_1	3060349	3061752	+	BAT2	branched-chain-amino-acid transaminase bat2
FUN_000985	contig_1	3062178	3063157	+		hypothetical protein
FUN_000986	contig_1	3063782	3065797	-		hypothetical protein
FUN_000988	contig_1	3067892	3070052	-	AGP2	proteinral amino acid permease agp2
FUN_000989	contig_1	3072173	3073514	+	SEN54	tRNA-splicing endonuclease subunit sen54
FUN_000990	contig_1	3073903	3074911	-		hypothetical protein
FUN_000991	contig_1	3075409	3079419	-	KCS1	inositol polyphosphate kinase kcs1
FUN_000993	contig_1	3083006	3088458	+	NUD1	Protein nud1
FUN_000994	contig_1	3088956	3091436	+	SHE4	SWI5-dependent HO expression protein 4
FUN_000995	contig_1	3091804	3092804	-		hypothetical protein
FUN_000996	contig_1	3093466	3095655	+	HSP78	chaperone ATPase hsp78
FUN_000997	contig_1	3095952	3098423	+		hypothetical protein
FUN_000998	contig_1	3105254	3106729	+		hypothetical protein
FUN_001000	contig_1	3115696	3117223	-		hypothetical protein
FUN_001001	contig_1	3122210	3125431	+		hypothetical protein
FUN_001002	contig_1	3125739	3126177	-		hypothetical protein
FUN_001003	contig_1	3126789	3127740	+		hypothetical protein
FUN_001005	contig_1	3130535	3131212	-		hypothetical protein
FUN_001007	contig_1	3134826	3136842	+		hypothetical protein
FUN_001008	contig_1	3137240	3139547	-		hypothetical protein
FUN_001009	contig_1	3139743	3140956	+		hypothetical protein
FUN_001010	contig_1	3142225	3145926	+	GDE1	Glycerophosphocholine phosphodiesterase
FUN_001011	contig_1	3146618	3147097	+		hypothetical protein
FUN_001012	contig_1	3147220	3148027	+		hypothetical protein
FUN_001013	contig_1	3151389	3153346	+		hypothetical protein
FUN_001014	contig_1	3154413	3155237	+		hypothetical protein
FUN_001015	contig_1	3155488	3156521	+		hypothetical protein
FUN_001017	contig_1	3160345	3167778	-		hypothetical protein
FUN_001018	contig_1	3169052	3170665	-		hypothetical protein
FUN_001019	contig_1	3171456	3172829	-		hypothetical protein
FUN_001020	contig_1	3173598	3177105	-		hypothetical protein
FUN_001023	contig_1	3191269	3192894	-	LCB4	sphinganine kinase lcb4
FUN_001024	contig_1	3193345	3196561	-	SEC26	coatomer subunit beta
FUN_001025	contig_1	3197021	3197771	+	MAGO2	Protein mago nashi 2
FUN_001026	contig_1	3198480	3199625	+		hypothetical protein
FUN_001027	contig_1	3200115	3201080	+	TRM8_2	tRNA (guanine-N(7)-)-methyltransferase (tRNA(m7G46)-methyltransferase)
FUN_001028	contig_1	3201280	3202060	-		hypothetical protein
FUN_001029	contig_1	3203003	3204776	+		hypothetical protein
FUN_001030	contig_1	3205115	3206312	-		hypothetical protein

Gene ID	Scaffold	Start	Stop	Strand	Name	Product
FUN_001031	contig_1	3206948	3211221	-		hypothetical protein
FUN_001032	contig_1	3211891	3213772	-		hypothetical protein
FUN_001033	contig_1	3217625	3219295	-	TRM2	tRNA(m5U54)methyltransferase
FUN_001034	contig_1	3219542	3220885	+	MRPS5	28S ribosomal protein S5, mitochondrial
FUN_001035	contig_1	3222401	3224550	-	NRC2	serine/threonine protein kinase, AGC
FUN_001036	contig_1	3225898	3228733	-	MRE11_1	meiotic recombination
FUN_001037	contig_1	3229433	3231826	-	MRE11_2	meiotic recombination
FUN_001038	contig_1	3232088	3232520	+	RSM19	mitochondrial ribosomal small subunit component
FUN_001039	contig_1	3233041	3233493	-		hypothetical protein
FUN_001041	contig_1	3236342	3237876	-	ARP3	Actin-related protein 3
FUN_001042	contig_1	3239059	3240656	+		hypothetical protein
FUN_001043	contig_1	3241402	3242380	-	TFB4	RNA polymerase II transcription factor B subunit 4
FUN_001044	contig_1	3243223	3245344	+		hypothetical protein
FUN_001045	contig_1	3246207	3248604	+		hypothetical protein
FUN_001046	contig_1	3249347	3250453	+	CKB1	casein kinase 2 regulatory subunit
FUN_001048	contig_1	3253088	3253928	-		hypothetical protein
FUN_001049	contig_1	3254886	3255436	-	RPL26A	60S ribosomal protein L26A
FUN_001050	contig_1	3255917	3259451	+		hypothetical protein
FUN_001051	contig_1	3260476	3261664	+	TMA46	Translation machinery-associated protein 46
FUN_001052	contig_1	3262279	3264214	-		hypothetical protein
FUN_001053	contig_1	3265486	3267616	+		hypothetical protein
FUN_001054	contig_1	3268244	3271747	-		hypothetical protein
FUN_001055	contig_1	3272271	3273389	+		hypothetical protein
FUN_001056	contig_1	3273565	3274250	-	MRP20	mitochondrial 54S ribosomal protein YmL41
FUN_001058	contig_1	3277579	3278472	+		hypothetical protein
FUN_001059	contig_1	3278925	3280162	-		hypothetical protein
FUN_001060	contig_1	3284835	3285590	+	GCN4	General control protein
FUN_001061	contig_1	3286714	3288531	-		hypothetical protein
FUN_001062	contig_1	3289103	3289443	+		hypothetical protein
FUN_001063	contig_1	3289930	3290859	-	ssb2	Replication factor A protein 2
FUN_001064	contig_1	3291092	3294090	+	DBP10	ATP-dependent RNA helicase dbp10
FUN_001065	contig_1	3303763	3304984	-	KRR1	ribosomal RNA assembly protein krr1
FUN_001066	contig_1	3305283	3306107	+		hypothetical protein
FUN_001067	contig_1	3306552	3307919	+		hypothetical protein
FUN_001068	contig_1	3311372	3312411	-		hypothetical protein
FUN_001070	contig_1	3316338	3318108	+		hypothetical protein
FUN_001071	contig_1	3318392	3318918	-	GIM5	subunit of tubulin prefoldin
FUN_001072	contig_1	3319448	3320715	-		hypothetical protein
FUN_001073	contig_1	3323713	3324603	+		hypothetical protein
FUN_001075	contig_1	3347061	3348149	-		hypothetical protein
FUN_001076	contig_1	3349362	3350504	-	RAD10	ssDNA endonuclease and repair protein rad10
FUN_001077	contig_1	3351065	3352260	+		hypothetical protein
FUN_001079	contig_1	3356940	3359404	-	DBP4	ATP-dependent RNA helicase dbp4
FUN_001080	contig_1	3359657	3360531	+	spf31	DnaJ sub C member 8
FUN_001081	contig_1	3360917	3361501	-		hypothetical protein
FUN_001082	contig_1	3361939	3363427	-		hypothetical protein
FUN_001084	contig_1	3369991	3371726	-		hypothetical protein
FUN_001085	contig_1	3375493	3375963	+		hypothetical protein
FUN_001087	contig_1	3380073	3381449	-		hypothetical protein
FUN_001088	contig_1	3382273	3382906	+	CYP10	Peptidyl-prolyl cis-trans isomerase cyp10
FUN_001089	contig_1	3383231	3384715	-	ZPR1	nucleolar zinc-finger protein
FUN_001090	contig_1	3384886	3385476	-		hypothetical protein
FUN_001091	contig_1	3385734	3387812	+	DBR1	lariat debranching enzyme
FUN_001092	contig_1	3392457	3394522	-		hypothetical protein
FUN_001093	contig_1	3397040	3398887	-	PUT2	1-pyrroline-5-carboxylate dehydrogenase
FUN_001094	contig_1	3399506	3401242	-	NUG1	nuclear GTP-binding protein nug1
FUN_001095	contig_1	3401824	3402752	+	GPP1	DL-glycerol-3-phosphatase
FUN_001096	contig_1	3404976	3407384	+		hypothetical protein
FUN_001097	contig_1	3410678	3411506	-	MET14	Adenylyl-sulfate kinase

Gene ID	Scaffold	Start	Stop	Strand	Name	Product
FUN_001099	contig_1	3414807	3415622	+	syf2	Pre-mRNA-splicing factor SYF2
FUN_001100	contig_1	3415971	3417014	-	ESF2	RNA-binding ATPase activator esf2
FUN_001101	contig_1	3417347	3418470	+	CWC25	RNA-splicing factor
FUN_001102	contig_1	3418872	3420470	-		hypothetical protein
FUN_001103	contig_1	3420634	3421619	+	PET8	S-adenosylmethionine transporter
FUN_001106	contig_1	3430823	3431946	+		hypothetical protein
FUN_001108	contig_1	3436025	3438364	+	CEF1	Pre-mRNA-splicing factor cef1
FUN_001109	contig_1	3439066	3442668	+		hypothetical protein
FUN_001111	contig_1	3446612	3448113	+		hypothetical protein
FUN_001112	contig_1	3448324	3448905	-		hypothetical protein
FUN_001113	contig_1	3465444	3471004	+	ags1	Cell wall alpha-1,3-glucan synthase ags1
FUN_001114	contig_1	3522469	3524457	-		hypothetical protein
FUN_001115	contig_1	3528061	3530474	-	MDL1	ATP-binding cassette permease mdl1
FUN_001116	contig_1	3531330	3532881	+	CDC37	hsp90 co-chaperone Cdc37
FUN_001117	contig_1	3533309	3534788	-		hypothetical protein
FUN_001118	contig_1	3535205	3536067	+	clc1	Clathrin light chain
FUN_001120	contig_1	3537209	3537655	-		hypothetical protein
FUN_001121	contig_1	3538576	3540881	+		hypothetical protein
FUN_001122	contig_1	3541356	3542101	-		hypothetical protein
FUN_001123	contig_1	3545976	3547717	+		hypothetical protein
FUN_001124	contig_1	3548358	3549577	+		hypothetical protein
FUN_001125	contig_1	3550152	3551754	+	RSB1	phospholipid-translocating ATPase rsb1
FUN_001126	contig_1	3552032	3552370	-	N19M	n19m, NADH-ubiquinone oxidoreductase 9.5 kDa subunit
FUN_001127	contig_1	3552680	3554346	+	WBP1	oligosaccharyl transferase glycoprotein complex, beta subunit
FUN_001128	contig_1	3554721	3556088	-		hypothetical protein
FUN_001130	contig_1	3561509	3562617	-		hypothetical protein
FUN_001131	contig_1	3565212	3566397	+		hypothetical protein
FUN_001133	contig_1	3570172	3572816	-		hypothetical protein
FUN_001134	contig_1	3574569	3575384	-		hypothetical protein
FUN_001135	contig_1	3575981	3577961	+		hypothetical protein
FUN_001136	contig_1	3578813	3580372	+		hypothetical protein
FUN_001137	contig_1	3580602	3582436	-		hypothetical protein
FUN_001140	contig_1	3588627	3589938	-		hypothetical protein
FUN_001141	contig_1	3591424	3592812	-		hypothetical protein
FUN_001142	contig_1	3593758	3595155	+		hypothetical protein
FUN_001143	contig_1	3595756	3597589	+	NDE1_1	NADH:ubiquinone oxidoreductase
FUN_001144	contig_1	3600891	3607942	+	ACC1	acetyl-coenzyme-A carboxylase
FUN_001145	contig_1	3609286	3610587	-		hypothetical protein
FUN_001146	contig_1	3611029	3611837	+		hypothetical protein
FUN_001147	contig_1	3612099	3613155	-	CBR1	NADH-cytochrome b5 reductase
FUN_001150	contig_1	3617545	3619567	-	CDC27	anaphase-promoting complex subunit cdc27
FUN_001151	contig_1	3619929	3620488	+		hypothetical protein
FUN_001152	contig_1	3620695	3622081	-		hypothetical protein
FUN_001153	contig_1	3623570	3625321	-	WHI3	cell cycle RNA binding protein whi3
FUN_001154	contig_1	3634325	3635692	+		hypothetical protein
FUN_001155	contig_1	3635910	3637493	+		hypothetical protein
FUN_001156	contig_1	3638520	3639876	+		hypothetical protein
FUN_001158	contig_1	3641713	3643383	-		hypothetical protein
FUN_001159	contig_1	3644899	3645414	-		hypothetical protein
FUN_001160	contig_1	3646072	3646947	+		hypothetical protein
FUN_001161	contig_1	3647975	3652327	+		hypothetical protein
FUN_001165	contig_1	3661786	3662883	+	SLC1	1-acylglycerol-3-phosphate O-acyltransferase
FUN_001166	contig_1	3663441	3667318	-		hypothetical protein
FUN_001169	contig_1	3672934	3674399	+		hypothetical protein
FUN_001170	contig_1	3674483	3675472	-		hypothetical protein
FUN_001171	contig_1	3677372	3678449	+		hypothetical protein
FUN_001173	contig_1	3682117	3683838	+		hypothetical protein

Gene ID	Scaffold	Start	Stop	Strand	Name	Product
FUN_001174	contig_1	3687204	3691407	+		hypothetical protein
FUN_001177	contig_1	3698259	3698849	+	CBC2	nuclear cap binding complex subunit
FUN_001178	contig_1	3700360	3701425	+		hypothetical protein
FUN_001179	contig_1	3702063	3703660	+		hypothetical protein
FUN_001180	contig_1	3705434	3707028	-		hypothetical protein
FUN_001181	contig_1	3710151	3713260	-		hypothetical protein
FUN_001183	contig_1	3715752	3720837	-		hypothetical protein
FUN_001187	contig_1	3727399	3727803	+		hypothetical protein
FUN_001188	contig_1	3728154	3729214	-		hypothetical protein
FUN_001189	contig_1	3730600	3731779	+		hypothetical protein
FUN_001190	contig_1	3733075	3734099	-		hypothetical protein
FUN_001191	contig_1	3734901	3735553	+		hypothetical protein
FUN_001192	contig_1	3735803	3736902	+		hypothetical protein
FUN_001193	contig_1	3738703	3740078	+		hypothetical protein
FUN_001195	contig_1	3748774	3749656	-	RSR1_1	Ras-related protein rsr1
FUN_001196	contig_1	3751737	3754710	+		hypothetical protein
FUN_001197	contig_1	3755322	3756128	+		hypothetical protein
FUN_001198	contig_1	3756248	3757219	-		hypothetical protein
FUN_001199	contig_1	3758766	3760628	-		hypothetical protein
FUN_001200	contig_1	3761980	3762831	+		hypothetical protein
FUN_001201	contig_1	3764287	3765495	+		hypothetical protein
FUN_001202	contig_1	3766205	3767753	-		hypothetical protein
FUN_001203	contig_1	3768842	3770787	-		hypothetical protein
FUN_001204	contig_1	3772043	3773718	-		hypothetical protein
FUN_001205	contig_1	3774558	3775265	+		hypothetical protein
FUN_001206	contig_1	3775606	3776239	-	HHF1_1	Histone H4
FUN_001207	contig_1	3779125	3780083	+		hypothetical protein
FUN_001209	contig_1	3782237	3783739	+		hypothetical protein
FUN_001210	contig_1	3784659	3785806	-		hypothetical protein
FUN_001212	contig_1	3788600	3790396	-		hypothetical protein
FUN_001214	contig_1	3795581	3797601	-		hypothetical protein
FUN_001216	contig_1	3810359	3810855	-	MRPL31	54S ribosomal protein L31, mitochondrial
FUN_001217	contig_1	3811186	3811718	+	TRS20	TRAPP subunit
FUN_001218	contig_1	3811959	3816976	-		hypothetical protein
FUN_001220	contig_1	3831572	3835769	+		hypothetical protein
FUN_001221	contig_1	3840760	3842928	+		hypothetical protein
FUN_001222	contig_1	3847550	3849700	-		hypothetical protein
FUN_001223	contig_1	3850479	3852874	-	MgSsk1	Two-component response regulator SSK1p
FUN_001224	contig_1	3856313	3857808	-	PKA4	Cytochrome c oxidase subunit 1
FUN_001225	contig_1	3859050	3859836	-	VMA11	v-type proton ATPase 16 kDa proteolipid subunit 2
FUN_001226	contig_1	3860473	3864296	+	ROM2	RHO1 GDP-GTP exchange protein 2
FUN_001229	contig_1	3867833	3869569	-		hypothetical protein
FUN_001230	contig_1	3870086	3870784	+		hypothetical protein
FUN_001231	contig_1	3870942	3872640	-		hypothetical protein
FUN_001232	contig_1	3876734	3878284	-	DIP5	amino acid transporter
FUN_001233	contig_1	3879499	3879966	-		hypothetical protein
FUN_001234	contig_1	3880041	3881634	-		hypothetical protein
FUN_001235	contig_1	3882802	3887875	+	TUS1	Rho guanine nucleotide exchange factor
FUN_001236	contig_1	3888236	3889785	-		hypothetical protein
FUN_001237	contig_1	3890478	3891623	+		hypothetical protein
FUN_001238	contig_1	3893241	3894936	+		hypothetical protein
FUN_001239	contig_1	3896183	3898114	+		hypothetical protein
FUN_001240	contig_1	3907567	3908814	-		hypothetical protein
FUN_001241	contig_1	3909130	3910399	+		hypothetical protein
FUN_001242	contig_1	3910880	3914369	+	GYP2	GTPase activating protein (GAP)
FUN_001243	contig_1	3914686	3916730	+		hypothetical protein
FUN_001245	contig_1	3922116	3923940	+		hypothetical protein
FUN_001247	contig_1	3929382	3930705	+	och1	alpha-1,6-mannosyltransferase Och1
FUN_001248	contig_1	3938123	3939473	+		hypothetical protein

Gene ID	Scaffold	Start	Stop	Strand	Name	Product
FUN_001249	contig_1	3939998	3941239	+		hypothetical protein
FUN_001251	contig_1	3943153	3943803	-		hypothetical protein
FUN_001252	contig_1	3944791	3945846	+		hypothetical protein
FUN_001253	contig_1	3946508	3947071	+		hypothetical protein
FUN_001254	contig_1	3947351	3948544	+		hypothetical protein
FUN_001255	contig_1	3948718	3950358	-		hypothetical protein
FUN_001256	contig_1	3951141	3953189	-		hypothetical protein
FUN_001257	contig_1	3954986	3956790	+		hypothetical protein
FUN_001258	contig_1	3959151	3963275	-		hypothetical protein
FUN_001259	contig_1	3965854	3968789	+		hypothetical protein
FUN_001260	contig_1	3969448	3970568	-		hypothetical protein
FUN_001261	contig_1	3973465	3974477	+		hypothetical protein
FUN_001262	contig_1	3979995	3980948	-		hypothetical protein
FUN_001263	contig_1	3987442	3989102	+		hypothetical protein
FUN_001264	contig_1	3989307	3990332	-		hypothetical protein
FUN_001265	contig_1	3991566	3992958	-		hypothetical protein
FUN_001266	contig_1	3995759	3996616	+		hypothetical protein
FUN_001268	contig_1	4000397	4000780	+		hypothetical protein
FUN_001269	contig_1	4001134	4002905	-		hypothetical protein
FUN_001270	contig_1	4003392	4004622	-		hypothetical protein
FUN_001273	contig_1	4013902	4015083	+		hypothetical protein
FUN_001274	contig_1	4015444	4017114	+		hypothetical protein
FUN_001277	contig_1	4025825	4026145	-		hypothetical protein
FUN_001278	contig_1	4028866	4031058	+		hypothetical protein
FUN_001279	contig_1	4032882	4035019	-	SUB8	serine protease
FUN_001281	contig_1	4036615	4037839	+		hypothetical protein
FUN_001282	contig_1	4038227	4042987	-		hypothetical protein
FUN_001283	contig_1	4044786	4046221	+		hypothetical protein
FUN_001284	contig_1	4046557	4048319	-		hypothetical protein
FUN_001287	contig_1	4059251	4059849	-		hypothetical protein
FUN_001288	contig_1	4059977	4062199	-		hypothetical protein
FUN_001290	contig_1	4065550	4067670	+		hypothetical protein
FUN_001293	contig_1	4070043	4072190	-	RAD30	DNA-directed DNA polymerase eta rad30
FUN_001294	contig_1	4079598	4081120	-		hypothetical protein
FUN_001295	contig_1	4082008	4083410	-		hypothetical protein
FUN_001296	contig_1	4084581	4086887	+		hypothetical protein
FUN_001298	contig_1	4088739	4089812	+		hypothetical protein
FUN_001299	contig_1	4090182	4091354	-		hypothetical protein
FUN_001301	contig_1	4094545	4098310	-		hypothetical protein
FUN_001302	contig_1	4099759	4101510	+	SPT8	Transcription factor spt8
FUN_001303	contig_1	4102558	4103745	+	IPK1	Inositol-pentakisphosphate 2-kinase
FUN_001304	contig_1	4104915	4106726	+	MDM34	ERMES complex subunit
FUN_001307	contig_1	4114584	4121895	-	MYO1_3	class II myosin
FUN_001308	contig_1	4122519	4125424	+		hypothetical protein
FUN_001309	contig_1	4125458	4125941	+		hypothetical protein
FUN_001310	contig_1	4127230	4128227	+		hypothetical protein
FUN_001311	contig_1	4129138	4130330	+	TAL1	Transaldolase
FUN_001312	contig_1	4130892	4131742	+	SOD1	Superoxide dismutase [Cu-Zn]
FUN_001313	contig_1	4132472	4135258	+		hypothetical protein
FUN_001315	contig_1	4158800	4160340	-	ANKRD49	Ankyrin repeat
FUN_001316	contig_1	4166334	4167890	-	SYR1	arginyl-tRNA synthetase
FUN_001317	contig_1	4168264	4170013	-		hypothetical protein
FUN_001319	contig_1	4177616	4178545	-		hypothetical protein
FUN_001320	contig_1	4179825	4182407	-		hypothetical protein
FUN_001322	contig_1	4187044	4190605	+		hypothetical protein
FUN_001324	contig_1	4195111	4197027	-	CTI6	Histone deacetylase complex subunit
FUN_001325	contig_1	4198139	4199800	-		hypothetical protein
FUN_001331	contig_1	4212942	4214706	-	DBP9	ATP-dependent DNA/RNA helicase
FUN_001332	contig_1	4215071	4215971	+		hypothetical protein

Gene ID	Scaffold	Start	Stop	Strand	Name	Product
FUN_001342	contig_1	4300237	4301883	-		hypothetical protein
FUN_001343	contig_1	4302737	4304549	-		hypothetical protein
FUN_001352	contig_1	4323328	4324965	-		hypothetical protein
FUN_001353	contig_1	4325561	4326943	+		hypothetical protein
FUN_001354	contig_1	4328743	4330322	-		hypothetical protein
FUN_001355	contig_1	4332495	4334257	+		hypothetical protein
FUN_001356	contig_1	4334888	4335440	+		hypothetical protein
FUN_001357	contig_1	4336614	4337319	-		hypothetical protein
FUN_001359	contig_1	4341030	4341755	-		hypothetical protein
FUN_001362	contig_1	4352097	4358547	+		hypothetical protein
FUN_001363	contig_1	4359059	4362607	-		hypothetical protein
FUN_001364	contig_1	4365084	4366127	-		hypothetical protein
FUN_001366	contig_1	4369199	4369791	-		hypothetical protein
FUN_001367	contig_1	4370371	4371132	+		hypothetical protein
FUN_001368	contig_1	4371660	4372584	-		hypothetical protein
FUN_001371	contig_1	4381620	4382309	-		hypothetical protein
FUN_001372	contig_1	4382873	4383871	-		hypothetical protein
FUN_001373	contig_1	4385428	4386213	-		hypothetical protein
FUN_001374	contig_1	4386846	4388310	-		hypothetical protein
FUN_001375	contig_1	4388725	4390539	-		hypothetical protein
FUN_001376	contig_1	4390870	4392796	-		hypothetical protein
FUN_001378	contig_1	4398566	4400379	+		hypothetical protein
FUN_001379	contig_1	4401604	4401945	+		hypothetical protein
FUN_001380	contig_1	4402290	4402862	+		hypothetical protein
FUN_001381	contig_1	4403195	4404868	-	RCO1	transcriptional regulatory protein rco1
FUN_001382	contig_1	4405303	4406887	-		hypothetical protein
FUN_001383	contig_1	4407168	4408112	+		hypothetical protein
FUN_001384	contig_1	4408800	4409928	-		hypothetical protein
FUN_001385	contig_1	4411991	4412492	+		hypothetical protein
FUN_001386	contig_1	4413093	4414842	-		hypothetical protein
FUN_001387	contig_1	4415379	4415705	-		hypothetical protein
FUN_001388	contig_1	4416373	4418162	+		hypothetical protein
FUN_001390	contig_2	6779	8965	-		hypothetical protein
FUN_001392	contig_2	13394	15502	-		hypothetical protein
FUN_001393	contig_2	16168	18347	+		hypothetical protein
FUN_001395	contig_2	20436	22159	+		hypothetical protein
FUN_001396	contig_2	22228	23365	-		hypothetical protein
FUN_001397	contig_2	23767	24681	+		hypothetical protein
FUN_001399	contig_2	33683	34225	+		hypothetical protein
FUN_001400	contig_2	34367	36232	-		hypothetical protein
FUN_001401	contig_2	39636	41811	-	UTP25	rRNA-binding ribosome biosynthesis protein utp25
FUN_001402	contig_2	42049	43185	+		hypothetical protein
FUN_001403	contig_2	43820	44600	-		hypothetical protein
FUN_001405	contig_2	50026	50993	-		hypothetical protein
FUN_001406	contig_2	51419	52812	-		hypothetical protein
FUN_001410	contig_2	59677	62739	+	MLH3	DNA mismatch repair protein
FUN_001411	contig_2	64157	64985	+		hypothetical protein
FUN_001412	contig_2	65243	66010	+		hypothetical protein
FUN_001413	contig_2	67983	70461	-		hypothetical protein
FUN_001415	contig_2	73075	73836	-		hypothetical protein
FUN_001416	contig_2	75006	75759	+		hypothetical protein
FUN_001419	contig_2	82171	82581	-		hypothetical protein
FUN_001420	contig_2	83334	86163	-	RNR1	ribonucleotide-diphosphate reductase subunit rnr1
FUN_001421	contig_2	86478	87128	+	mrpl8	54S ribosomal protein L8, mitochondrial
FUN_001422	contig_2	87878	90539	+	SEC23	GTPase-activating protein S23
FUN_001424	contig_2	93998	95186	-	HLJ1	Chaperone protein dnaJ
FUN_001425	contig_2	96380	99800	-	UFD2	Ubiquitin conjugation factor E4
FUN_001426	contig_2	102219	103043	+	RHO1	GTP-binding protein Rho1
FUN_001427	contig_2	104422	106050	-		hypothetical protein

Gene ID	Scaffold	Start	Stop	Strand	Name	Product
FUN_001428	contig_2	106930	109520	-		hypothetical protein
FUN_001429	contig_2	109838	110919	+	FMT1	Methionyl-tRNA formyltransferase
FUN_001431	contig_2	113937	114537	+		hypothetical protein
FUN_001434	contig_2	116547	117321	+		hypothetical protein
FUN_001435	contig_2	117754	118956	-	MVD1	diphosphomevalonate decarboxylase
FUN_001436	contig_2	119348	120244	+	CYT2	Cytochrome c1 heme lyase
FUN_001437	contig_2	120512	121374	-	PEP12	SNAP receptor
FUN_001438	contig_2	121765	123704	+		hypothetical protein
FUN_001442	contig_2	130374	132869	-	CEX1	Nuclear aminoacylation-dependent tRNA export pathway component
FUN_001444	contig_2	137533	138026	-		hypothetical protein
FUN_001445	contig_2	138818	139773	+		hypothetical protein
FUN_001446	contig_2	140773	141513	-	hus5	SUMO conjugating enzyme Hus5
FUN_001447	contig_2	142052	142968	+	PEX19	Peroxisome chaperone and import receptor
FUN_001449	contig_2	147582	148638	+		hypothetical protein
FUN_001452	contig_2	152272	152988	-		hypothetical protein
FUN_001453	contig_2	154551	155749	+		hypothetical protein
FUN_001455	contig_2	158905	159850	-		hypothetical protein
FUN_001456	contig_2	160190	161385	+		hypothetical protein
FUN_001458	contig_2	164240	166386	+	MPP10	U3 snoRNP protein
FUN_001459	contig_2	167024	168473	-		hypothetical protein
FUN_001460	contig_2	169478	171265	+		hypothetical protein
FUN_001468	contig_2	189633	191758	-		hypothetical protein
FUN_001469	contig_2	192241	194108	-	DyP1	dye-decolorizing heme-containing peroxidase
FUN_001470	contig_2	195574	196863	-		hypothetical protein
FUN_001471	contig_2	197704	199200	+		hypothetical protein
FUN_001472	contig_2	199729	201006	+		hypothetical protein
FUN_001474	contig_2	204161	204993	+		hypothetical protein
FUN_001475	contig_2	206214	206924	-		hypothetical protein
FUN_001476	contig_2	209126	211018	-		hypothetical protein
FUN_001477	contig_2	214437	215037	-		hypothetical protein
FUN_001478	contig_2	216236	218236	+		hypothetical protein
FUN_001479	contig_2	220996	222735	-		hypothetical protein
FUN_001480	contig_2	223351	225297	-		hypothetical protein
FUN_001481	contig_2	226173	227359	-		hypothetical protein
FUN_001482	contig_2	229371	230431	+		hypothetical protein
FUN_001483	contig_2	230867	231649	-		hypothetical protein
FUN_001489	contig_2	242359	243521	-		hypothetical protein
FUN_001490	contig_2	245649	246409	-	GET1	GET complex subunit get1
FUN_001493	contig_2	257118	258978	-		hypothetical protein
FUN_001494	contig_2	261196	262809	+		hypothetical protein
FUN_001495	contig_2	263736	266012	+		hypothetical protein
FUN_001496	contig_2	267369	271244	-		hypothetical protein
FUN_001497	contig_2	271982	274843	-		hypothetical protein
FUN_001498	contig_2	276862	279729	+		hypothetical protein
FUN_001500	contig_2	281952	283650	-	MUQ1	choline phosphate cytidylyltransferase
FUN_001502	contig_2	286317	286691	-	ARD1	N-terminal acetyltransferase A complex catalytic subunit ard1
FUN_001503	contig_2	288141	290018	-		hypothetical protein
FUN_001504	contig_2	291155	294556	-		hypothetical protein
FUN_001505	contig_2	294610	295057	-		hypothetical protein
FUN_001506	contig_2	296692	297512	+		hypothetical protein
FUN_001507	contig_2	298560	300746	+		hypothetical protein
FUN_001508	contig_2	301126	301938	+		hypothetical protein
FUN_001509	contig_2	304258	305441	+		hypothetical protein
FUN_001510	contig_2	306173	307675	+		hypothetical protein
FUN_001512	contig_2	319316	320902	+		hypothetical protein
FUN_001513	contig_2	321258	322625	-		hypothetical protein
FUN_001514	contig_2	323366	324514	-		hypothetical protein

Gene ID	Scaffold	Start	Stop	Strand	Name	Product
FUN_001516	contig_2	328795	330237	+		hypothetical protein
FUN_001517	contig_2	331173	332500	-		hypothetical protein
FUN_001518	contig_2	334132	336193	+		hypothetical protein
FUN_001519	contig_2	337910	340326	+		hypothetical protein
FUN_001521	contig_2	344068	345661	-		hypothetical protein
FUN_001522	contig_2	346150	348236	+		hypothetical protein
FUN_001523	contig_2	348900	350782	-	FOL3	folylpolyglutamate synthase
FUN_001525	contig_2	356145	356527	+		hypothetical protein
FUN_001526	contig_2	358541	360577	+		hypothetical protein
FUN_001527	contig_2	361556	364444	+		hypothetical protein
FUN_001528	contig_2	365527	367017	-	GLR1_1	Glutathione reductase
FUN_001529	contig_2	369313	370735	+		hypothetical protein
FUN_001530	contig_2	373836	375245	-		hypothetical protein
FUN_001532	contig_2	380148	382838	+		hypothetical protein
FUN_001533	contig_2	383351	384634	+	CIA1	Cytosolic iron-sulfur protein assembly protein
FUN_001534	contig_2	385348	386809	+	PCT1	choline-phosphate cytidylyltransferase
FUN_001535	contig_2	390004	391833	+	SOK1	Protein SOSEKI 1
FUN_001537	contig_2	393813	395394	+		hypothetical protein
FUN_001538	contig_2	395902	397408	-	ADE13	adenylosuccinase ade13
FUN_001539	contig_2	398105	399145	-		hypothetical protein
FUN_001541	contig_2	407066	408339	-		hypothetical protein
FUN_001542	contig_2	408857	409459	+	QCR7	Cytochrome b-c1 complex subunit 7, mitochondrial
FUN_001543	contig_2	410275	412392	+	MAG1	3-methyladenine DNA glycosylase
FUN_001544	contig_2	415289	416587	+	ecm33_1	cell wall protein Ecm33
FUN_001545	contig_2	421821	423290	+		hypothetical protein
FUN_001546	contig_2	423865	425761	-	HXT5_1	hexose transporter hxt5
FUN_001547	contig_2	426869	428293	-		hypothetical protein
FUN_001548	contig_2	429220	432046	-		hypothetical protein
FUN_001549	contig_2	435818	436527	-		hypothetical protein
FUN_001550	contig_2	438164	440871	-		hypothetical protein
FUN_001551	contig_2	441977	443747	-		hypothetical protein
FUN_001552	contig_2	444281	448026	-	PHO81	phosphate system positive regulatory protein pho81
FUN_001553	contig_2	449222	452868	-	CRM1	Karyopherin transporter
FUN_001554	contig_2	454256	454774	+	YPI1	Type 1 phosphatases regulator ypi1
FUN_001555	contig_2	455268	455823	-	DAD2	DASH complex subunit dad2
FUN_001556	contig_2	456091	458434	+		hypothetical protein
FUN_001559	contig_2	465900	468205	+		hypothetical protein
FUN_001560	contig_2	472733	473308	-		hypothetical protein
FUN_001561	contig_2	474814	476057	-		hypothetical protein
FUN_001562	contig_2	477175	478704	+	CHL4	chromosome loss-related protein
FUN_001564	contig_2	480152	484028	-		hypothetical protein
FUN_001565	contig_2	485014	485815	+	COQ2	Para-hydroxybenzoate--polyprenyltransferase, mitochondrial precursor (PHB:polyprenyltransferase)
FUN_001566	contig_2	488176	489247	-		hypothetical protein
FUN_001567	contig_2	489874	490562	+		hypothetical protein
FUN_001568	contig_2	496117	497767	-		hypothetical protein
FUN_001569	contig_2	498015	498491	-		hypothetical protein
FUN_001570	contig_2	501071	502337	+	YVH1	tyrosine protein phosphatase yvh1
FUN_001571	contig_2	505007	507564	-	ACO2_1	aconitate hydratase
FUN_001572	contig_2	508134	510011	-	ACO2_2	aconitate hydratase
FUN_001573	contig_2	510317	511361	+	ILV6	acetolactate synthase, regulatory subunit
FUN_001574	contig_2	512999	515623	+		hypothetical protein
FUN_001575	contig_2	516291	518621	+	GUT2	mitochondrial glycerol-3-phosphate dehydrogenase
FUN_001576	contig_2	521540	522022	+		hypothetical protein
FUN_001578	contig_2	533365	536317	+	SOG2	RAM signaling network component
FUN_001579	contig_2	537001	539823	-		hypothetical protein
FUN_001580	contig_2	540204	541395	+	ARG3	ornithine carbamoyltransferase
FUN_001581	contig_2	542057	542594	+		hypothetical protein
FUN_001582	contig_2	543284	544600	-		hypothetical protein

Gene ID	Scaffold	Start	Stop	Strand	Name	Product
FUN_001583	contig_2	544993	546801	-		hypothetical protein
FUN_001584	contig_2	547651	548268	+		hypothetical protein
FUN_001585	contig_2	550133	551125	+		hypothetical protein
FUN_001586	contig_2	552216	553460	+		hypothetical protein
FUN_001587	contig_2	553566	556070	-	CWH41_1	Processing alpha glucosidase I
FUN_001588	contig_2	556614	557498	+		hypothetical protein
FUN_001589	contig_2	557930	558892	+		hypothetical protein
FUN_001590	contig_2	560183	561463	+		hypothetical protein
FUN_001591	contig_2	562613	563788	-		hypothetical protein
FUN_001593	contig_2	565729	566565	-		hypothetical protein
FUN_001594	contig_2	567409	568463	+		hypothetical protein
FUN_001595	contig_2	570535	572654	-		hypothetical protein
FUN_001596	contig_2	575267	576234	-		hypothetical protein
FUN_001597	contig_2	576817	577997	+	SCS2	phosphatidylinositol-binding protein scs2
FUN_001598	contig_2	578742	580940	+	ASN1	asparagine synthetase
FUN_001599	contig_2	581394	583358	-	SWC4	swr complex subunit
FUN_001600	contig_2	584140	585900	-	YCK2	Palmitoylated plasma membrane-bound casein kinase
FUN_001601	contig_2	587049	591281	-		hypothetical protein
FUN_001602	contig_2	592228	593518	-		hypothetical protein
FUN_001603	contig_2	593942	594740	+	DRE2	electron carrier
FUN_001604	contig_2	594981	596684	-		hypothetical protein
FUN_001605	contig_2	597454	598321	-		hypothetical protein
FUN_001606	contig_2	598917	599905	-		hypothetical protein
FUN_001607	contig_2	600802	601609	+	ERP1	emp24p/erv25p-related protein
FUN_001609	contig_2	609632	610032	+	GRX5	monothiol glutaredoxin grx5
FUN_001611	contig_2	611951	613072	-	CCP1	heme peroxidase
FUN_001613	contig_2	616349	618049	+	TNA1_1	High-affinity nicotinic acid transporter
FUN_001614	contig_2	618387	619182	-		hypothetical protein
FUN_001615	contig_2	619397	619927	+	rps23	ribosomal protein S23
FUN_001616	contig_2	620446	622305	-		hypothetical protein
FUN_001617	contig_2	622747	624359	+		hypothetical protein
FUN_001618	contig_2	624627	625514	-	ALG14	UDP-N-acetylglucosamine transferase subunit
FUN_001619	contig_2	626049	627468	-	GCD7	GCD complex subunit gcd7
FUN_001620	contig_2	628098	633652	+	NEM1	Nuclear envelope morphology protein 1
FUN_001621	contig_2	634735	636756	+		hypothetical protein
FUN_001622	contig_2	639963	640781	+		hypothetical protein
FUN_001623	contig_2	644685	645533	+	srb5	Mediator of RNA polymerase II transcription subunit 18
FUN_001624	contig_2	646078	647486	+	pnk1	DNA kinase/phosphatase Pnk1
FUN_001625	contig_2	651237	652773	+		hypothetical protein
FUN_001626	contig_2	655531	656805	-		hypothetical protein
FUN_001627	contig_2	660788	661739	+		hypothetical protein
FUN_001628	contig_2	661941	663015	-		hypothetical protein
FUN_001629	contig_2	665511	668420	+		hypothetical protein
FUN_001630	contig_2	669798	670452	+		hypothetical protein
FUN_001631	contig_2	670829	672631	-	NOP15	nucleolar protein
FUN_001632	contig_2	672942	673698	+		hypothetical protein
FUN_001633	contig_2	674735	675458	+		hypothetical protein
FUN_001635	contig_2	679130	679598	+		hypothetical protein
FUN_001636	contig_2	679677	680093	-		hypothetical protein
FUN_001637	contig_2	680610	681009	+		hypothetical protein
FUN_001639	contig_2	685141	685917	-		hypothetical protein
FUN_001640	contig_2	686488	687510	+		hypothetical protein
FUN_001641	contig_2	687900	690324	-		hypothetical protein
FUN_001642	contig_2	692612	694432	-	chk1	Chk1 protein kinase
FUN_001643	contig_2	697089	699122	-	SST2	human AMSH/STAMBP protein ubiquitin specific-protease
FUN_001646	contig_2	709907	711001	-		hypothetical protein
FUN_001647	contig_2	711237	712986	+	ALG11	asparagine-linked glycosylation protein
FUN_001648	contig_2	713578	714266	-		hypothetical protein

Gene ID	Scaffold	Start	Stop	Strand	Name	Product
FUN_001649	contig_2	715319	718731	-		hypothetical protein
FUN_001650	contig_2	721235	722222	-		hypothetical protein
FUN_001651	contig_2	723445	725099	-		hypothetical protein
FUN_001652	contig_2	725417	725904	+	ALTA12	60S acidic ribosomal protein P1
FUN_001653	contig_2	726592	727778	-	HEM2	Aminolevulinate dehydratase
FUN_001658	contig_2	741061	741566	-	NAT2	DUF1279 super
FUN_001659	contig_2	745107	746745	+		hypothetical protein
FUN_001660	contig_2	750550	751164	+		hypothetical protein
FUN_001662	contig_2	754603	757566	-		hypothetical protein
FUN_001664	contig_2	762353	764011	+		hypothetical protein
FUN_001665	contig_2	764608	766873	-	GPI10	glycosylphosphatidylinositol anchor biosynthesis
FUN_001666	contig_2	768951	773168	+		hypothetical protein
FUN_001667	contig_2	776047	776535	+	COA1	cytochrome oxidase assembly protein 1
FUN_001669	contig_2	782035	784022	-	UGA4	GABA-specific high-affinity permease
FUN_001670	contig_2	784956	787113	-		hypothetical protein
FUN_001671	contig_2	787605	788178	-		hypothetical protein
FUN_001672	contig_2	788414	792036	-		hypothetical protein
FUN_001673	contig_2	792366	794722	+		hypothetical protein
FUN_001674	contig_2	797134	797913	+		hypothetical protein
FUN_001676	contig_2	813393	813724	-		hypothetical protein
FUN_001677	contig_2	815248	816649	+	LEU2	3-isopropylmalate dehydrogenase
FUN_001678	contig_2	817899	818836	+		hypothetical protein
FUN_001681	contig_2	822107	826158	+	RSE1	pre-mRNA-splicing factor rse1
FUN_001682	contig_2	827653	830425	+	PAN3	PAB-dependent poly(A)-specific ribonuclease subunit 3
FUN_001683	contig_2	830894	832229	+	ADO1	adenosine kinase
FUN_001684	contig_2	832621	833508	-		hypothetical protein
FUN_001685	contig_2	834073	836846	+		hypothetical protein
FUN_001686	contig_2	837769	839368	-	ucp10	UBX domain-containing protein 10
FUN_001689	contig_2	845945	849358	-		hypothetical protein
FUN_001690	contig_2	853267	854806	-		hypothetical protein
FUN_001691	contig_2	855857	858333	+		hypothetical protein
FUN_001692	contig_2	860500	862567	-		hypothetical protein
FUN_001693	contig_2	863482	864268	-	URE2_1	Transcriptional regulator ure2
FUN_001694	contig_2	865593	867317	-		hypothetical protein
FUN_001696	contig_2	871674	873026	+		hypothetical protein
FUN_001697	contig_2	873249	874112	-		hypothetical protein
FUN_001698	contig_2	875219	877074	+		hypothetical protein
FUN_001699	contig_2	878057	881131	+		hypothetical protein
FUN_001700	contig_2	881894	882963	-		hypothetical protein
FUN_001701	contig_2	883233	884537	-		hypothetical protein
FUN_001703	contig_2	895000	896085	-		hypothetical protein
FUN_001704	contig_2	897486	898937	+		hypothetical protein
FUN_001705	contig_2	902499	903469	-		hypothetical protein
FUN_001707	contig_2	905323	907252	-		hypothetical protein
FUN_001708	contig_2	908979	910376	-		hypothetical protein
FUN_001709	contig_2	914181	916383	-		hypothetical protein
FUN_001710	contig_2	916563	919848	+		hypothetical protein
FUN_001711	contig_2	922810	923898	+		hypothetical protein
FUN_001712	contig_2	924025	924552	+		hypothetical protein
FUN_001713	contig_2	925321	927123	+		hypothetical protein
FUN_001714	contig_2	927341	928592	-		hypothetical protein
FUN_001715	contig_2	930435	932303	+		hypothetical protein
FUN_001716	contig_2	932947	934765	-		hypothetical protein
FUN_001717	contig_2	935559	939292	-	PBP1	poly(A)-binding protein binding protein
FUN_001718	contig_2	940471	944056	+	BUD6	Bud site selection protein 6
FUN_001719	contig_2	945478	946608	-	LST8	TOR complex subunit lst8
FUN_001720	contig_2	947344	948895	+		hypothetical protein
FUN_001721	contig_2	949365	951111	-	PTC2	Protein phosphatase 2C 2
FUN_001722	contig_2	952314	954542	+		hypothetical protein

Gene ID	Scaffold	Start	Stop	Strand	Name	Product
FUN_001723	contig_2	955118	956044	+		hypothetical protein
FUN_001724	contig_2	959108	960353	+	POR1	Mitochondrial porin
FUN_001725	contig_2	961698	963235	+	MAP2	Methionine aminopeptidase 2
FUN_001726	contig_2	963620	965224	+	UBE2D2	Ubiquitin-conjugating enzyme E2 D2
FUN_001727	contig_2	965956	966788	+	vps29	Vacuolar protein sorting-associated protein 29
FUN_001728	contig_2	967431	968552	-		hypothetical protein
FUN_001730	contig_2	981491	983371	+		hypothetical protein
FUN_001731	contig_2	983538	987658	-	ELP1	Putative elongator complex protein 1
FUN_001732	contig_2	988832	992233	+	MTR4	ATP-dependent RNA helicase mtr4
FUN_001733	contig_2	992837	993829	+	STS1	Tethering factor for nuclear proteasome sts1
FUN_001736	contig_2	1002260	1005280	+		hypothetical protein
FUN_001740	contig_2	1012161	1017645	-	PKS81	Non-reducing polyketide synthase PKS8-1
FUN_001741	contig_2	1018656	1019721	+		hypothetical protein
FUN_001742	contig_2	1020563	1020959	-		hypothetical protein
FUN_001743	contig_2	1021505	1021739	+		hypothetical protein
FUN_001744	contig_2	1021783	1022189	+		hypothetical protein
FUN_001745	contig_2	1022381	1024446	-		hypothetical protein
FUN_001747	contig_2	1027977	1029197	+		hypothetical protein
FUN_001748	contig_2	1029752	1032056	+		hypothetical protein
FUN_001750	contig_2	1035500	1036296	-		hypothetical protein
FUN_001751	contig_2	1044865	1047821	-	EFM6	Protein-lysine N-methyltransferase efm6
FUN_001752	contig_2	1048428	1050353	+	CNH1_1	Na+/H+ antiporter
FUN_001753	contig_2	1050515	1052192	-		hypothetical protein
FUN_001754	contig_2	1052978	1055595	-		hypothetical protein
FUN_001755	contig_2	1056176	1057566	-	ARO2	bifunctional chorismate synthase/riboflavin reductase [NAD(P)H] aro2
FUN_001756	contig_2	1057844	1060298	-		hypothetical protein
FUN_001758	contig_2	1065808	1067826	-		hypothetical protein
FUN_001759	contig_2	1068243	1069955	+	PTC5	[Pyruvate dehydrogenase [acetyl-transferring]]-phosphatase 1, mitochondrial
FUN_001761	contig_2	1071025	1072024	+		hypothetical protein
FUN_001764	contig_2	1075979	1077250	+	DES1	sphingolipid delta-4 desaturase
FUN_001765	contig_2	1077487	1078302	-	RSM25	mitochondrial ribosomal small subunit component
FUN_001766	contig_2	1078650	1080404	+		hypothetical protein
FUN_001767	contig_2	1082782	1083349	-		hypothetical protein
FUN_001768	contig_2	1083747	1084658	-		hypothetical protein
FUN_001770	contig_2	1086954	1088535	-		hypothetical protein
FUN_001771	contig_2	1089297	1090323	+		hypothetical protein
FUN_001772	contig_2	1090515	1091739	-		hypothetical protein
FUN_001773	contig_2	1093837	1095093	-		hypothetical protein
FUN_001774	contig_2	1096251	1097774	-		hypothetical protein
FUN_001775	contig_2	1098657	1100111	+		hypothetical protein
FUN_001776	contig_2	1103872	1105155	+		hypothetical protein
FUN_001777	contig_2	1105616	1107422	-	PHO5_1	acid phosphatase pho5
FUN_001778	contig_2	1110616	1112181	+		hypothetical protein
FUN_001782	contig_2	1119797	1121259	-		hypothetical protein
FUN_001785	contig_2	1127703	1128995	+		hypothetical protein
FUN_001786	contig_2	1131402	1132676	+		hypothetical protein
FUN_001787	contig_2	1136311	1138396	+	lcc1_2	laccase, multicopper oxidase, benzenediol:oxygen oxidorectuctase
FUN_001788	contig_2	1139117	1140625	+		hypothetical protein
FUN_001789	contig_2	1143565	1144902	+		hypothetical protein
FUN_001790	contig_2	1146156	1147344	-	MIR1	mitochondrial phosphate carrier protein
FUN_001792	contig_2	1150779	1152684	+		hypothetical protein
FUN_001794	contig_2	1154107	1155909	-		hypothetical protein
FUN_001795	contig_2	1156951	1159871	-		hypothetical protein
FUN_001796	contig_2	1162194	1162958	-		hypothetical protein
FUN_001798	contig_2	1171505	1173285	-		hypothetical protein
FUN_001799	contig_2	1173867	1174541	-	SNU13	RNA binding protein snu13

Gene ID	Scaffold	Start	Stop	Strand	Name	Product
FUN_001800	contig_2	1174754	1177150	-	PUS7	multisubstrate pseudouridine synthase 7
FUN_001801	contig_2	1178403	1180754	-		hypothetical protein
FUN_001802	contig_2	1183550	1184722	-	YEY2_1	Valine--pyruvate aminotransferase
FUN_001803	contig_2	1186032	1188685	+		hypothetical protein
FUN_001804	contig_2	1189597	1190879	+	fsf1	Sideroflexin FSF1
FUN_001805	contig_2	1191643	1194839	+	LHS1	lumenal Hsp70 protein
FUN_001806	contig_2	1195817	1197162	-		hypothetical protein
FUN_001807	contig_2	1198378	1200078	-	ssh4	Protein ssh4
FUN_001808	contig_2	1205133	1208076	+		hypothetical protein
FUN_001809	contig_2	1218105	1219206	+		hypothetical protein
FUN_001816	contig_2	1237265	1237835	-		hypothetical protein
FUN_001817	contig_2	1238553	1239258	+		hypothetical protein
FUN_001818	contig_2	1239478	1240716	-		hypothetical protein
FUN_001819	contig_2	1241580	1242378	+		hypothetical protein
FUN_001821	contig_2	1247420	1248603	+		hypothetical protein
FUN_001825	contig_2	1264310	1266136	-	GCD1	Translation initiation factor eIF-2B subunit gamma
FUN_001826	contig_2	1266429	1267718	+		hypothetical protein
FUN_001827	contig_2	1269615	1271002	+		hypothetical protein
FUN_001829	contig_2	1274697	1275440	-		hypothetical protein
FUN_001830	contig_2	1275842	1277050	-		hypothetical protein
FUN_001831	contig_2	1278052	1279735	+		hypothetical protein
FUN_001832	contig_2	1280041	1280643	-		hypothetical protein
FUN_001833	contig_2	1281198	1283199	+		hypothetical protein
FUN_001835	contig_2	1286626	1287858	+		hypothetical protein
FUN_001836	contig_2	1288300	1290084	+		hypothetical protein
FUN_001837	contig_2	1293820	1294242	+		hypothetical protein
FUN_001838	contig_2	1294657	1295805	+		hypothetical protein
FUN_001840	contig_2	1297483	1298802	-		hypothetical protein
FUN_001841	contig_2	1299729	1300286	+		hypothetical protein
FUN_001842	contig_2	1301398	1302936	+		hypothetical protein
FUN_001843	contig_2	1303561	1305375	+		hypothetical protein
FUN_001844	contig_2	1309961	1312273	+		hypothetical protein
FUN_001845	contig_2	1312866	1315154	-		hypothetical protein
FUN_001846	contig_2	1318019	1318476	-	rec14	Ski complex subunit Rec14
FUN_001847	contig_2	1318960	1319627	+	GUK1	guanylate kinase
FUN_001848	contig_2	1320820	1322071	+	GLE2	RNA export factor gle2
FUN_001849	contig_2	1322567	1324402	-	RAD52	DNA repair protein rad52
FUN_001850	contig_2	1324777	1326772	-	KEX1	Cell death protease
FUN_001851	contig_2	1327860	1328740	-		hypothetical protein
FUN_001852	contig_2	1330230	1330668	+		hypothetical protein
FUN_001853	contig_2	1332869	1333522	-	UPS2	Phospholipid metabolism protein
FUN_001855	contig_2	1336963	1337263	-	ATX1	Cytosolic copper metallochaperone
FUN_001856	contig_2	1337982	1340604	+	RIM20	pH-response regulator protein palA/rim20
FUN_001858	contig_2	1345186	1347411	+	CRZ1	DNA-binding transcription factor
FUN_001859	contig_2	1352014	1353209	+	MCT1	[acyl-carrier-protein] S-malonyltransferase
FUN_001860	contig_2	1353472	1354609	-		hypothetical protein
FUN_001861	contig_2	1355033	1358218	-	NUT1	mediator complex subunit
FUN_001863	contig_2	1361709	1363004	+		hypothetical protein
FUN_001864	contig_2	1363882	1365417	+	GDH3	NADP-dependent glutamate dehydrogenase
FUN_001866	contig_2	1369467	1372385	-		hypothetical protein
FUN_001867	contig_2	1372659	1373330	+		hypothetical protein
FUN_001868	contig_2	1376140	1378695	+		hypothetical protein
FUN_001869	contig_2	1383010	1383747	+		hypothetical protein
FUN_001870	contig_2	1383835	1384506	-		hypothetical protein
FUN_001871	contig_2	1384576	1385241	-		hypothetical protein
FUN_001874	contig_2	1391118	1391964	-	DOT5	thioredoxin peroxidase dot5
FUN_001875	contig_2	1393316	1394914	+		hypothetical protein
FUN_001877	contig_2	1403129	1404906	-	ALG10	glucosyltransferase
FUN_001878	contig_2	1405113	1405978	+	PSF2	DNA replication protein psf2

Gene ID	Scaffold	Start	Stop	Strand	Name	Product
FUN_001879	contig_2	1414035	1417819	+	BUD5	Ras guanine nucleotide exchange factor bud5
FUN_001880	contig_2	1418460	1420917	+		hypothetical protein
FUN_001881	contig_2	1421366	1421984	+	RPL31B	60S ribosomal protein L31B
FUN_001882	contig_2	1422443	1424415	-		hypothetical protein
FUN_001883	contig_2	1426346	1427005	+		hypothetical protein
FUN_001884	contig_2	1429838	1431494	-	SPT14	Phosphatidylinositol N-acetylglucosaminyltransferase GPI3 subunit
FUN_001885	contig_2	1431795	1434150	+	DBP6	ATP-dependent RNA helicase dbp6
FUN_001886	contig_2	1436363	1437872	+		hypothetical protein
FUN_001887	contig_2	1439287	1440012	-	SEC65	signal recognition particle subunit
FUN_001888	contig_2	1442451	1443890	+		hypothetical protein
FUN_001889	contig_2	1446248	1448525	+	LKH1	serine threonine protein kinase CMGC group
FUN_001890	contig_2	1449345	1450552	+		hypothetical protein
FUN_001891	contig_2	1450856	1453750	-		hypothetical protein
FUN_001892	contig_2	1454463	1456312	-		hypothetical protein
FUN_001893	contig_2	1457111	1459230	+	wis1	MAP kinase kinase Wis1
FUN_001894	contig_2	1460085	1462407	+	SEC63	secretory subunit
FUN_001897	contig_2	1468624	1470768	-		hypothetical protein
FUN_001900	contig_2	1477928	1479760	+		hypothetical protein
FUN_001901	contig_2	1480176	1481467	-		hypothetical protein
FUN_001903	contig_2	1487864	1489098	-	URA3_1	orotidine 5'-phosphate decarboxylase
FUN_001905	contig_2	1492285	1493165	+		hypothetical protein
FUN_001906	contig_2	1493409	1494094	-		hypothetical protein
FUN_001907	contig_2	1494162	1495375	-		hypothetical protein
FUN_001908	contig_2	1496435	1497857	+	FTR1	high-affinity iron permease
FUN_001910	contig_2	1500533	1501583	+		hypothetical protein
FUN_001911	contig_2	1504389	1505471	-	SWC5	swr complex subunit
FUN_001912	contig_2	1507607	1508749	-		hypothetical protein
FUN_001913	contig_2	1510785	1511336	+		hypothetical protein
FUN_001915	contig_2	1514256	1515514	+		hypothetical protein
FUN_001916	contig_2	1517491	1519627	+		hypothetical protein
FUN_001917	contig_2	1520978	1523256	+		hypothetical protein
FUN_001918	contig_2	1525575	1526844	+	SCS7	fatty acid alpha-hydroxylase
FUN_001919	contig_2	1527452	1529245	+	rsd1	Phosphatidylinositol-3-phosphatase SAC1
FUN_001921	contig_2	1534594	1536311	-		hypothetical protein
FUN_001923	contig_2	1540197	1540941	-	CNB1	Calcineurin subunit B
FUN_001924	contig_2	1541164	1541843	+		hypothetical protein
FUN_001925	contig_2	1542387	1543094	-		hypothetical protein
FUN_001926	contig_2	1546044	1547370	+		hypothetical protein
FUN_001927	contig_2	1547786	1548880	-		hypothetical protein
FUN_001928	contig_2	1550248	1552950	+	RDH54	helicase
FUN_001929	contig_2	1553137	1554084	-		hypothetical protein
FUN_001930	contig_2	1554865	1559136	-		hypothetical protein
FUN_001931	contig_2	1559965	1560753	-		hypothetical protein
FUN_001932	contig_2	1561365	1562704	+	SUR2	Sphingolipid C4-hydroxylase sur2
FUN_001938	contig_2	1574061	1575839	-		hypothetical protein
FUN_001939	contig_2	1576305	1577523	-		hypothetical protein
FUN_001940	contig_2	1578893	1580586	+		hypothetical protein
FUN_001941	contig_2	1580782	1582853	+		hypothetical protein
FUN_001942	contig_2	1584259	1586793	-		hypothetical protein
FUN_001943	contig_2	1587666	1588931	-	GTT1	bifunctional glutathione transferase/peroxidase
FUN_001944	contig_2	1590375	1591544	-		hypothetical protein
FUN_001945	contig_2	1592839	1594381	+		hypothetical protein
FUN_001948	contig_2	1600161	1601097	-		hypothetical protein
FUN_001949	contig_2	1601765	1605015	-		hypothetical protein
FUN_001950	contig_2	1605461	1606858	-		hypothetical protein
FUN_001952	contig_2	1611285	1612630	-		hypothetical protein
FUN_001953	contig_2	1613795	1615950	+		hypothetical protein
FUN_001954	contig_2	1619997	1622361	+	BNI4	bud neck involved protein

Gene ID	Scaffold	Start	Stop	Strand	Name	Product
FUN_001955	contig_2	1623810	1624414	+	RPS25	40S ribosomal protein S25
FUN_001956	contig_2	1625340	1626350	-		hypothetical protein
FUN_001957	contig_2	1627235	1628604	-	GTR2	GTP-binding protein gtr2
FUN_001959	contig_2	1632780	1633405	+	ARL3	ADP-ribosylation factor protein 3
FUN_001960	contig_2	1633851	1635041	-		hypothetical protein
FUN_001961	contig_2	1635782	1637534	-	DUS1	tRNA dihydrouridine synthase
FUN_001962	contig_2	1639048	1639692	-		hypothetical protein
FUN_001963	contig_2	1641165	1643535	+		hypothetical protein
FUN_001964	contig_2	1644132	1645098	+		hypothetical protein
FUN_001966	contig_2	1648875	1649772	+	RPS18	ribosomal 40S subunit protein S18B
FUN_001968	contig_2	1653688	1654632	-		hypothetical protein
FUN_001969	contig_2	1655333	1657011	+		hypothetical protein
FUN_001970	contig_2	1657573	1659295	+		hypothetical protein
FUN_001971	contig_2	1659745	1661085	+	MSS51	translational activator for mitochondrial COX1
FUN_001972	contig_2	1661594	1661960	-		hypothetical protein
FUN_001973	contig_2	1663561	1664184	-	RPS22	40S ribosomal protein S22
FUN_001976	contig_2	1668055	1669996	-		hypothetical protein
FUN_001977	contig_2	1670993	1673755	+	HSP98	Heat shock protein hsp98
FUN_001978	contig_2	1674277	1675890	-	PTC6	Protein phosphatase 2C 6
FUN_001980	contig_2	1678745	1681811	+	kap95	karyopherin Kap95
FUN_001981	contig_2	1683460	1684409	+		hypothetical protein
FUN_001982	contig_2	1684912	1686229	-	NOP1	Small subunit processome complex component
FUN_001983	contig_2	1687800	1688816	-		hypothetical protein
FUN_001986	contig_2	1695650	1696255	+		hypothetical protein
FUN_001987	contig_2	1697071	1698503	-		hypothetical protein
FUN_001988	contig_2	1700901	1701452	+		hypothetical protein
FUN_001990	contig_2	1705223	1708142	+		hypothetical protein
FUN_001991	contig_2	1708455	1709396	-		hypothetical protein
FUN_001992	contig_2	1710414	1711453	+		hypothetical protein
FUN_001993	contig_2	1714330	1716072	+		hypothetical protein
FUN_001994	contig_2	1716725	1720425	-		hypothetical protein
FUN_001995	contig_2	1721132	1722281	+		hypothetical protein
FUN_001996	contig_2	1722680	1723402	-		hypothetical protein
FUN_001997	contig_2	1723727	1724524	-		hypothetical protein
FUN_001998	contig_2	1725371	1727329	-	TEF4_1	elongation factor EF-1 gamma subunit
FUN_001999	contig_2	1728808	1730325	-	TEF4_2	elongation factor EF-1 gamma subunit
FUN_002000	contig_2	1730561	1731450	+	MRPS9	37S ribosomal protein S9, mitochondrial
FUN_002003	contig_2	1743607	1744005	+		hypothetical protein
FUN_002007	contig_2	1752824	1754363	+		hypothetical protein
FUN_002008	contig_2	1754600	1755577	+		hypothetical protein
FUN_002009	contig_2	1756944	1757519	-		hypothetical protein
FUN_002012	contig_2	1763361	1764261	-		hypothetical protein
FUN_002013	contig_2	1764601	1766175	-		hypothetical protein
FUN_002014	contig_2	1768461	1770242	-		hypothetical protein
FUN_002017	contig_2	1776757	1777596	+		hypothetical protein
FUN_002018	contig_2	1777973	1780007	-		hypothetical protein
FUN_002019	contig_2	1780372	1781183	-		hypothetical protein
FUN_002020	contig_2	1782159	1783929	-		hypothetical protein
FUN_002021	contig_2	1784768	1785951	-		hypothetical protein
FUN_002023	contig_2	1788714	1790178	-		hypothetical protein
FUN_002024	contig_2	1790583	1792368	+		hypothetical protein
FUN_002026	contig_2	1793959	1794597	+	AQY1_1	Aquaporin-1
FUN_002027	contig_2	1794971	1796432	-		hypothetical protein
FUN_002028	contig_2	1798746	1800415	+		hypothetical protein
FUN_002029	contig_2	1800860	1802535	+	TPN1_1	Vitamin B6 transporter
FUN_002031	contig_2	1806543	1808066	-		hypothetical protein
FUN_002032	contig_2	1809159	1811298	-		hypothetical protein
FUN_002034	contig_2	1816448	1821065	-	CDC15	Protein kinase of the Mitotic Exit Network
FUN_002035	contig_2	1821995	1823332	-		hypothetical protein

Gene ID	Scaffold	Start	Stop	Strand	Name	Product
FUN_002036	contig_2	1825005	1826000	-		hypothetical protein
FUN_002037	contig_2	1826618	1828920	+		hypothetical protein
FUN_002038	contig_2	1829609	1830726	+		hypothetical protein
FUN_002039	contig_2	1830846	1832100	-		hypothetical protein
FUN_002040	contig_2	1834069	1835320	+		hypothetical protein
FUN_002042	contig_2	1838864	1840728	-		hypothetical protein
FUN_002045	contig_2	1849665	1852011	-		hypothetical protein
FUN_002047	contig_2	1857513	1858751	-		hypothetical protein
FUN_002048	contig_2	1859914	1861202	-		hypothetical protein
FUN_002050	contig_2	1863939	1865943	+		hypothetical protein
FUN_002051	contig_2	1866296	1866865	-		hypothetical protein
FUN_002052	contig_2	1867164	1868347	+	CDC10	cell division control protein
FUN_002053	contig_2	1869041	1869718	-		hypothetical protein
FUN_002054	contig_2	1870060	1872115	+	RAR1	Putative methionine--tRNA ligase, cytoplasmic protein rar1
FUN_002055	contig_2	1878621	1880404	+		hypothetical protein
FUN_002056	contig_2	1880939	1881967	+		hypothetical protein
FUN_002058	contig_2	1888569	1889630	-		hypothetical protein
FUN_002059	contig_2	1890507	1891935	+		hypothetical protein
FUN_002060	contig_2	1899032	1901020	+		hypothetical protein
FUN_002061	contig_2	1903554	1904310	-		hypothetical protein
FUN_002062	contig_2	1907619	1908947	-		hypothetical protein
FUN_002063	contig_2	1910694	1912676	-		hypothetical protein
FUN_002067	contig_2	1925751	1927257	+		hypothetical protein
FUN_002068	contig_2	1927895	1929916	+		hypothetical protein
FUN_002071	contig_2	1939316	1939890	+	AQY1_2	Aquaporin-1
FUN_002072	contig_2	1940410	1941446	-		hypothetical protein
FUN_002073	contig_2	1942727	1943829	+		hypothetical protein
FUN_002074	contig_2	1943875	1945620	-		hypothetical protein
FUN_002075	contig_2	1946483	1947865	+		hypothetical protein
FUN_002077	contig_2	1953237	1955132	-		hypothetical protein
FUN_002078	contig_2	1957532	1958562	-		hypothetical protein
FUN_002079	contig_2	1963457	1965868	+		hypothetical protein
FUN_002080	contig_2	1966449	1968690	-		hypothetical protein
FUN_002081	contig_2	1969307	1971523	+		hypothetical protein
FUN_002083	contig_2	1976810	1977770	-		hypothetical protein
FUN_002084	contig_2	1978673	1981068	+	MET6	methionine-synthesizing 5-methyltetrahydropteroyltriglutamate--homocysteine methyltransferase
FUN_002085	contig_2	1981972	1983635	-		hypothetical protein
FUN_002087	contig_2	1987265	1989142	+		hypothetical protein
FUN_002088	contig_2	1989422	1990345	+		hypothetical protein
FUN_002089	contig_2	1990738	1994164	+		hypothetical protein
FUN_002090	contig_2	1995147	1996996	+		hypothetical protein
FUN_002091	contig_2	1998415	2000067	-		hypothetical protein
FUN_002092	contig_2	2002102	2003523	+	REG1_2	protein phosphatase regulator
FUN_002093	contig_2	2003561	2005235	-		hypothetical protein
FUN_002096	contig_2	2007686	2008388	+		hypothetical protein
FUN_002098	contig_2	2011326	2012377	-		hypothetical protein
FUN_002100	contig_2	2017068	2018109	+		hypothetical protein
FUN_002101	contig_2	2018328	2018757	+		hypothetical protein
FUN_002102	contig_2	2020497	2022130	+		hypothetical protein
FUN_002105	contig_2	2026292	2028028	-		hypothetical protein
FUN_002107	contig_2	2030879	2032089	-		hypothetical protein
FUN_002108	contig_2	2035938	2036588	-	URA3_2	orotidine 5'-phosphate decarboxylase
FUN_002109	contig_2	2038605	2039870	-		hypothetical protein
FUN_002110	contig_2	2040939	2043967	+		hypothetical protein
FUN_002111	contig_2	2044927	2046648	+	faeB-1_1	Feruloyl esterase
FUN_002114	contig_2	2050441	2050990	+		hypothetical protein

Gene ID	Scaffold	Start	Stop	Strand	Name	Product
FUN_002116	contig_2	2052549	2053736	+		hypothetical protein
FUN_002117	contig_2	2055657	2056478	-		hypothetical protein
FUN_002118	contig_2	2058418	2059630	+		hypothetical protein
FUN_002119	contig_2	2060344	2061368	+		hypothetical protein
FUN_002122	contig_2	2072167	2073429	-		hypothetical protein
FUN_002123	contig_2	2073906	2075304	-		hypothetical protein
FUN_002124	contig_2	2077338	2078689	+		hypothetical protein
FUN_002125	contig_2	2079653	2080597	-		hypothetical protein
FUN_002126	contig_2	2082659	2084844	+		hypothetical protein
FUN_002127	contig_2	2085312	2088610	-		hypothetical protein
FUN_002128	contig_2	2089056	2090208	+		hypothetical protein
FUN_002129	contig_2	2091191	2094878	+		hypothetical protein
FUN_002130	contig_2	2095225	2096224	-		hypothetical protein
FUN_002131	contig_2	2097599	2098767	+		hypothetical protein
FUN_002133	contig_2	2108234	2111425	-		hypothetical protein
FUN_002134	contig_2	2111830	2117232	+	UTP10	snoRNA-binding rRNA-processing protein utp10
FUN_002135	contig_2	2117548	2119639	-	LEU4	2-isopropylmalate synthase (Alpha-isopropylmalate synthase) (Alpha-IPM synthetase)
FUN_002136	contig_2	2120080	2121910	-		hypothetical protein
FUN_002137	contig_2	2122729	2123641	+		hypothetical protein
FUN_002138	contig_2	2123681	2124660	-		hypothetical protein
FUN_002139	contig_2	2125075	2126133	-	AAH1_1	adenine deaminase
FUN_002140	contig_2	2129783	2131397	+		hypothetical protein
FUN_002141	contig_2	2133729	2135811	+		hypothetical protein
FUN_002143	contig_2	2137913	2139538	-	PSD1	phosphatidylserine decarboxylase 1
FUN_002145	contig_2	2142858	2143987	-	TES1	acyl-CoA thioesterase
FUN_002146	contig_2	2144668	2150509	+		hypothetical protein
FUN_002147	contig_2	2151062	2152095	+	RPL2	60S ribosomal protein L2
FUN_002148	contig_2	2152723	2153825	-		hypothetical protein
FUN_002151	contig_2	2165596	2166041	-	lsm7	U6 snRNP-associated protein Lsm7
FUN_002152	contig_2	2167273	2169225	+		hypothetical protein
FUN_002154	contig_2	2174689	2176899	+		hypothetical protein
FUN_002155	contig_2	2179222	2181202	-		hypothetical protein
FUN_002156	contig_2	2184584	2185343	-		hypothetical protein
FUN_002157	contig_2	2188095	2188586	-		hypothetical protein
FUN_002158	contig_2	2189766	2190524	-		hypothetical protein
FUN_002159	contig_2	2194236	2201496	+		hypothetical protein
FUN_002160	contig_2	2205529	2206808	-		hypothetical protein
FUN_002163	contig_2	2217074	2217709	-		hypothetical protein
FUN_002165	contig_2	2220028	2221228	-		hypothetical protein
FUN_002167	contig_2	2223827	2224282	+		hypothetical protein
FUN_002168	contig_2	2224711	2227164	-		hypothetical protein
FUN_002170	contig_2	2229402	2230246	-		hypothetical protein
FUN_002171	contig_2	2231416	2232205	+		hypothetical protein
FUN_002172	contig_2	2233166	2235065	+		hypothetical protein
FUN_002174	contig_2	2241648	2244903	-		hypothetical protein
FUN_002176	contig_2	2250106	2252032	-	DML1	mtDNA inheritance, partitioning of the mitochondrial organelle
FUN_002177	contig_2	2252337	2253419	-		hypothetical protein
FUN_002178	contig_2	2255014	2257758	-		hypothetical protein
FUN_002182	contig_2	2265254	2266650	-		hypothetical protein
FUN_002184	contig_2	2268269	2269512	-		hypothetical protein
FUN_002185	contig_2	2271302	2272479	-		hypothetical protein
FUN_002186	contig_2	2273345	2277461	+		hypothetical protein
FUN_002187	contig_2	2279911	2281178	-		hypothetical protein
FUN_002189	contig_2	2285654	2287299	+		hypothetical protein
FUN_002192	contig_2	2293886	2295589	-		hypothetical protein
FUN_002194	contig_2	2298661	2300577	-		hypothetical protein
FUN_002195	contig_2	2301474	2302573	+		hypothetical protein

Gene ID	Scaffold	Start	Stop	Strand	Name	Product
FUN_002197	contig_2	2306094	2308578	+	SDA1_1	Severe Depolymerization of Actin
FUN_002198	contig_2	2310507	2311949	-		hypothetical protein
FUN_002200	contig_2	2315749	2316694	-		hypothetical protein
FUN_002201	contig_2	2317413	2318569	+		hypothetical protein
FUN_002202	contig_2	2319778	2321680	+	PAP1	polynucleotide adenylyltransferase
FUN_002203	contig_2	2323253	2325169	-		hypothetical protein
FUN_002204	contig_2	2325781	2328788	+	YSH1	endoribonuclease ysh1
FUN_002206	contig_2	2332696	2333501	+		hypothetical protein
FUN_002207	contig_2	2333944	2334141	+		hypothetical protein
FUN_002208	contig_2	2334285	2338919	+		hypothetical protein
FUN_002209	contig_2	2339972	2340820	+		hypothetical protein
FUN_002210	contig_2	2342250	2344649	+		hypothetical protein
FUN_002212	contig_2	2348225	2349283	-	pis1	phosphatidylinositol synthase 1 (CDP-alcohol phosphatidyltransferase1)
FUN_002213	contig_2	2350218	2352679	-	DRS1	nucleolar DEAD-box protein required for synthesis of 60S ribosomal subunit
FUN_002214	contig_2	2353397	2354582	+		hypothetical protein
FUN_002215	contig_2	2357850	2359381	-		hypothetical protein
FUN_002216	contig_2	2360165	2363041	+		hypothetical protein
FUN_002217	contig_2	2366029	2367823	+		hypothetical protein
FUN_002218	contig_2	2368868	2371792	+		hypothetical protein
FUN_002219	contig_2	2372657	2373586	-		hypothetical protein
FUN_002220	contig_2	2376700	2377647	+		hypothetical protein
FUN_002221	contig_2	2378008	2379790	+		hypothetical protein
FUN_002222	contig_2	2380639	2382039	-		hypothetical protein
FUN_002223	contig_2	2389099	2390163	+	ATG27	type II membrane protein
FUN_002224	contig_2	2390695	2392150	-		hypothetical protein
FUN_002225	contig_2	2393139	2394032	+		hypothetical protein
FUN_002228	contig_2	2407040	2408803	-	ALO1	D-arabinono-1,4-lactone oxidase
FUN_002231	contig_2	2414446	2415435	+		hypothetical protein
FUN_002233	contig_2	2418041	2419126	+		hypothetical protein
FUN_002236	contig_2	2422379	2423171	+		hypothetical protein
FUN_002237	contig_2	2423532	2426535	-		hypothetical protein
FUN_002238	contig_2	2427565	2429297	+		hypothetical protein
FUN_002240	contig_2	2431561	2433088	+		hypothetical protein
FUN_002241	contig_2	2433262	2434850	-		hypothetical protein
FUN_002243	contig_2	2435864	2437519	-		hypothetical protein
FUN_002244	contig_2	2438014	2440002	-		hypothetical protein
FUN_002245	contig_2	2440767	2441867	+		hypothetical protein
FUN_002246	contig_2	2442219	2443898	-	RET2	coatomer subunit delta
FUN_002247	contig_2	2444751	2446909	+		hypothetical protein
FUN_002248	contig_2	2449227	2449963	+		hypothetical protein
FUN_002249	contig_2	2450517	2453216	-		hypothetical protein
FUN_002251	contig_2	2458379	2459841	-		hypothetical protein
FUN_002252	contig_2	2461033	2462151	+		hypothetical protein
FUN_002253	contig_2	2467306	2469153	+		hypothetical protein
FUN_002254	contig_2	2469807	2470361	-		hypothetical protein
FUN_002255	contig_2	2472204	2474434	+		hypothetical protein
FUN_002256	contig_2	2476218	2477916	+		hypothetical protein
FUN_002257	contig_2	2479467	2480150	+		hypothetical protein
FUN_002258	contig_2	2480432	2481158	-	ALG3_1	dolichyl-P-Man:Man(5)GlcNAc(2)-PP-dolichol alpha-1,3-mannosyltransferase
FUN_002259	contig_2	2481976	2483721	+		hypothetical protein
FUN_002260	contig_2	2484817	2485941	-		hypothetical protein
FUN_002261	contig_2	2488358	2491445	+		hypothetical protein
FUN_002262	contig_2	2493436	2494892	+	HGT1_1	high affinity glucose transporter
FUN_002264	contig_2	2498002	2499915	+		hypothetical protein
FUN_002265	contig_2	2500016	2500544	-		hypothetical protein
FUN_002266	contig_2	2500744	2502548	-		hypothetical protein

Gene ID	Scaffold	Start	Stop	Strand	Name	Product
FUN_002268	contig_2	2506380	2507959	-		hypothetical protein
FUN_002270	contig_2	2510958	2512268	-		hypothetical protein
FUN_002273	contig_2	2524664	2525476	-		hypothetical protein
FUN_002274	contig_2	2527953	2528788	+		hypothetical protein
FUN_002275	contig_2	2531632	2532376	-		hypothetical protein
FUN_002276	contig_2	2533259	2534695	+	ATR1	multidrug-resistance type transporter aminotriazole resistance
FUN_002277	contig_2	2536878	2538045	-	lad1	L-arabinitol 4-dehydrogenase
FUN_002278	contig_2	2538627	2540058	-		hypothetical protein
FUN_002279	contig_2	2541582	2543350	+	MSK1	mitochondrial lysine-tRNA synthetase
FUN_002280	contig_2	2543834	2545195	+		hypothetical protein
FUN_002281	contig_2	2545998	2546537	-		hypothetical protein
FUN_002282	contig_2	2549932	2551723	+		hypothetical protein
FUN_002283	contig_2	2553341	2554474	-		hypothetical protein
FUN_002284	contig_2	2554651	2555453	-	VPS28	Vacuolar protein-sorting-associated protein 28
FUN_002285	contig_2	2555671	2558045	+	NOC2	Nucleolar Complex 2 protein
FUN_002286	contig_2	2558316	2560811	+	PXA1	ATP-binding cassette long-chain fatty acid transporter pxa1
FUN_002287	contig_2	2563465	2565030	+		hypothetical protein
FUN_002288	contig_2	2565616	2567405	-		hypothetical protein
FUN_002289	contig_2	2568090	2570127	+	NGG1	Transcriptional regulator
FUN_002290	contig_2	2570413	2571902	-		hypothetical protein
FUN_002291	contig_2	2574077	2574784	+		hypothetical protein
FUN_002292	contig_2	2576079	2576497	+		hypothetical protein
FUN_002293	contig_2	2578359	2579785	-		hypothetical protein
FUN_002294	contig_2	2581026	2582209	-		hypothetical protein
FUN_002295	contig_2	2582468	2583357	+		hypothetical protein
FUN_002296	contig_2	2584022	2585261	-		hypothetical protein
FUN_002297	contig_2	2586127	2588843	+	HIS4	trifunctional histidinol dehydrogenase
FUN_002298	contig_2	2589634	2590982	+	XYNF3	Endo-1,4-beta-xylanase F3
FUN_002299	contig_2	2591338	2592472	-		hypothetical protein
FUN_002300	contig_2	2592982	2595185	-		hypothetical protein
FUN_002302	contig_2	2597830	2598699	-		hypothetical protein
FUN_002303	contig_2	2599929	2603414	+	CCC2	Cu(2+)-transporting P-type ATPase
FUN_002304	contig_2	2604761	2605847	+		hypothetical protein
FUN_002305	contig_2	2606434	2607880	+		hypothetical protein
FUN_002307	contig_2	2610212	2612903	-	HRD3	ERAD-associated protein
FUN_002308	contig_2	2613897	2615259	+		hypothetical protein
FUN_002309	contig_2	2615978	2617444	+		hypothetical protein
FUN_002311	contig_2	2620617	2624751	-		hypothetical protein
FUN_002312	contig_2	2625811	2626170	+		hypothetical protein
FUN_002313	contig_2	2626630	2627836	+		hypothetical protein
FUN_002315	contig_2	2630088	2631767	+	MST1	threonyl-tRNA synthetase
FUN_002316	contig_2	2632471	2635017	+		hypothetical protein
FUN_002317	contig_2	2635551	2637072	-	BTS1	geranylgeranyl pyrophosphate synthetase
FUN_002320	contig_2	2644471	2645266	+		hypothetical protein
FUN_002321	contig_2	2647645	2651010	+	BUD14_1	protein phosphatase regulator
FUN_002322	contig_2	2652528	2654291	-		hypothetical protein
FUN_002323	contig_2	2655143	2656951	+		hypothetical protein
FUN_002324	contig_2	2661566	2662988	+		hypothetical protein
FUN_002325	contig_2	2664169	2666138	-		hypothetical protein
FUN_002326	contig_2	2668871	2670444	-		hypothetical protein
FUN_002327	contig_2	2671437	2673529	+		hypothetical protein
FUN_002328	contig_2	2677022	2678226	+		hypothetical protein
FUN_002330	contig_2	2681105	2684693	-		hypothetical protein
FUN_002331	contig_2	2685165	2688343	-		hypothetical protein
FUN_002332	contig_2	2688776	2690187	+		hypothetical protein
FUN_002333	contig_2	2690359	2691299	-		hypothetical protein
FUN_002334	contig_2	2692983	2693853	+		hypothetical protein

Gene ID	Scaffold	Start	Stop	Strand	Name	Product
FUN_002335	contig_2	2695785	2697547	-	CSN12	COP9 signalosome (CSN) subunit
FUN_002336	contig_2	2698198	2698884	+	RPL20B	60S ribosomal protein L20B
FUN_002337	contig_2	2699415	2700452	+		hypothetical protein
FUN_002339	contig_2	2702861	2705182	-		hypothetical protein
FUN_002341	contig_2	2708589	2710039	+		hypothetical protein
FUN_002342	contig_2	2710474	2713003	-		hypothetical protein
FUN_002343	contig_2	2713692	2715048	+		hypothetical protein
FUN_002344	contig_2	2715577	2717167	+	UAP1	UDP-N-acetylglucosamine pyrophosphorylase
FUN_002345	contig_2	2721338	2722151	+	DLH1	carboxymethylenebutenolidase
FUN_002347	contig_2	2726449	2731127	+		hypothetical protein
FUN_002350	contig_2	2734665	2735770	-		hypothetical protein
FUN_002351	contig_2	2736413	2738743	-	cat1	catalase 1
FUN_002352	contig_2	2746579	2747877	+	MDM12_1	Mitochondrial distribution and morphology protein 12
FUN_002353	contig_2	2749786	2753033	+		hypothetical protein
FUN_002354	contig_2	2753706	2757000	-		hypothetical protein
FUN_002355	contig_2	2758164	2760186	+		hypothetical protein
FUN_002356	contig_2	2760899	2761959	+		hypothetical protein
FUN_002357	contig_2	2762662	2764295	-		hypothetical protein
FUN_002359	contig_2	2773792	2775005	+		hypothetical protein
FUN_002363	contig_2	2790758	2791926	-	LAE1	Secondary metabolism regulator lae1
FUN_002364	contig_2	2797123	2798727	-	ARR3	arsenicals resistance
FUN_002365	contig_2	2799196	2799990	-		hypothetical protein
FUN_002366	contig_2	2800741	2802414	-		hypothetical protein
FUN_002369	contig_2	2807830	2810191	-		hypothetical protein
FUN_002373	contig_2	2814798	2817993	-		hypothetical protein
FUN_002374	contig_2	2818209	2820865	-		hypothetical protein
FUN_002375	contig_2	2821682	2825398	+		hypothetical protein
FUN_002376	contig_2	2825757	2826352	-		hypothetical protein
FUN_002377	contig_2	2826717	2828699	+	UBP1	ubiquitin-specific protease ubp1
FUN_002378	contig_2	2829288	2830291	+	GLO2	Cytoplasmic glyoxalase II
FUN_002381	contig_2	2832855	2833422	-		hypothetical protein
FUN_002382	contig_2	2833995	2835776	+		hypothetical protein
FUN_002383	contig_2	2838123	2839135	-		hypothetical protein
FUN_002384	contig_2	2839541	2841461	+		hypothetical protein
FUN_002385	contig_2	2841757	2845098	-	CNH1_2	Na+/H+ antiporter
FUN_002386	contig_2	2847027	2849472	-		hypothetical protein
FUN_002387	contig_2	2851216	2853907	+	DPB11	protein kinase activating protein dpb11
FUN_002388	contig_2	2854038	2855110	-		hypothetical protein
FUN_002389	contig_2	2855564	2857954	+		hypothetical protein
FUN_002390	contig_2	2858844	2860218	-		hypothetical protein
FUN_002391	contig_2	2860950	2862037	+		hypothetical protein
FUN_002392	contig_2	2862520	2864853	+	ASE1	Microtubule bundling protein
FUN_002394	contig_2	2871073	2873229	+	RIM21	pH-response regulator protein palH/rim21
FUN_002395	contig_2	2873800	2876143	+	MVP1	Sorting nexin mvp1
FUN_002396	contig_2	2877997	2880526	+	DCN1_1	Scaffold-type E3 ligase
FUN_002397	contig_2	2881085	2881671	+	DCN1_2	Scaffold-type E3 ligase
FUN_002400	contig_2	2894809	2895429	+		hypothetical protein
FUN_002401	contig_2	2898947	2901161	+		hypothetical protein
FUN_002402	contig_2	2904055	2904404	+		hypothetical protein
FUN_002403	contig_2	2905180	2907865	+		hypothetical protein
FUN_002404	contig_2	2908874	2909842	+		hypothetical protein
FUN_002405	contig_2	2910449	2911760	+		hypothetical protein
FUN_002408	contig_2	2918131	2920381	+		hypothetical protein
FUN_002409	contig_2	2922274	2923293	-	CHT2_1	Chitinase 2
FUN_002410	contig_2	2924799	2926281	-		hypothetical protein
FUN_002411	contig_2	2927027	2928063	-	GP1	fungal class II heme-containing peroxidase
FUN_002412	contig_2	2930904	2932373	+	RIB2	DRAP deaminase
FUN_002413	contig_2	2932566	2933815	-		hypothetical protein
FUN_002414	contig_2	2935942	2937527	-		hypothetical protein

Gene ID	Scaffold	Start	Stop	Strand	Name	Product
FUN_002415	contig_2	2941698	2943558	-		hypothetical protein
FUN_002416	contig_2	2944553	2945658	+		hypothetical protein
FUN_002417	contig_2	2945728	2946181	-		hypothetical protein
FUN_002418	contig_2	2946484	2948425	-		hypothetical protein
FUN_002419	contig_2	2949000	2950648	+		hypothetical protein
FUN_002420	contig_2	2950873	2951673	-		hypothetical protein
FUN_002422	contig_2	2954439	2954823	+		hypothetical protein
FUN_002423	contig_2	2954953	2956039	-		hypothetical protein
FUN_002424	contig_2	2956662	2957266	+		hypothetical protein
FUN_002425	contig_2	2958532	2959596	+		hypothetical protein
FUN_002427	contig_2	2966923	2969142	+	CIPK17	CBL-interacting protein kinase
FUN_002428	contig_2	2970091	2971116	+		hypothetical protein
FUN_002429	contig_2	2971157	2973855	-	APE2_1	Aminopeptidase 2 mitochondrial
FUN_002430	contig_2	2974358	2977706	+		hypothetical protein
FUN_002431	contig_2	2980824	2982019	-		hypothetical protein
FUN_002432	contig_2	2983880	2986114	-		hypothetical protein
FUN_002433	contig_2	2987403	2988871	+		hypothetical protein
FUN_002435	contig_2	2992392	2993654	-	ebp2	rRNA-processing protein EBP2
FUN_002436	contig_2	2993976	2994326	+		hypothetical protein
FUN_002437	contig_2	2994652	2995780	-		hypothetical protein
FUN_002438	contig_2	2996303	2996812	-	TRX3	mitochondrial thioredoxin
FUN_002439	contig_2	2997386	3000524	-		hypothetical protein
FUN_002440	contig_2	3001198	3002921	-		hypothetical protein
FUN_002441	contig_2	3003622	3005022	-	RNR2	Ribonucleotide-diphosphate reductase (RNR), small subunit
FUN_002442	contig_2	3005942	3007880	-		hypothetical protein
FUN_002443	contig_2	3008560	3009616	+		hypothetical protein
FUN_002444	contig_2	3010309	3012348	-		hypothetical protein
FUN_002445	contig_2	3012898	3014900	-	VAC8	Vacuolar protein 8
FUN_002446	contig_2	3016203	3018083	+		hypothetical protein
FUN_002447	contig_2	3020004	3022272	+		hypothetical protein
FUN_002450	contig_2	3025589	3026355	+	trs31	Trafficking protein particle complex subunit 31
FUN_002451	contig_2	3026783	3029061	-		hypothetical protein
FUN_002452	contig_2	3029587	3029929	+		hypothetical protein
FUN_002453	contig_2	3030454	3031957	+		hypothetical protein
FUN_002455	contig_2	3036320	3040364	-		hypothetical protein
FUN_002457	contig_2	3043898	3047619	-		hypothetical protein
FUN_002458	contig_2	3049347	3049987	+		hypothetical protein
FUN_002460	contig_2	3055866	3056611	-	OAC1	Mitochondrial oxaloacetate carrier protein
FUN_002461	contig_2	3057395	3058616	+		hypothetical protein
FUN_002462	contig_2	3058996	3060174	-	LAS1	rRNA-processing protein las1
FUN_002464	contig_2	3070206	3073068	-		hypothetical protein
FUN_002465	contig_2	3074119	3075682	+	SEC61	translocon subunit
FUN_002466	contig_2	3076003	3076791	-		hypothetical protein
FUN_002467	contig_2	3077792	3078556	+		hypothetical protein
FUN_002468	contig_2	3081038	3084295	+		hypothetical protein
FUN_002469	contig_2	3084668	3085567	-	NIT3	Omega-amidase nit3
FUN_002472	contig_2	3094562	3097369	+	URE1	Urease
FUN_002473	contig_2	3098563	3100122	+		hypothetical protein
FUN_002475	contig_2	3103049	3105381	-		hypothetical protein
FUN_002476	contig_2	3106219	3108938	-	MRD1	Multiple RNA-binding domain-containing protein 1
FUN_002477	contig_2	3109223	3109883	+	DYN2	Dynein light chain
FUN_002480	contig_2	3115277	3116707	-		hypothetical protein
FUN_002481	contig_2	3118627	3119331	-	SCD1	Scytalone dehydratase
FUN_002483	contig_2	3123553	3124445	-		hypothetical protein
FUN_002484	contig_2	3125868	3126980	+		hypothetical protein
FUN_002487	contig_2	3137945	3139018	-		hypothetical protein
FUN_002488	contig_2	3139450	3140632	-	MCR1	NADH-cytochrome b5 reductase
FUN_002490	contig_2	3144661	3145571	-		hypothetical protein

Gene ID	Scaffold	Start	Stop	Strand	Name	Product
FUN_002493	contig_2	3155253	3158347	-	ARO80	zinc finger transcriptional activator
FUN_002494	contig_2	3160066	3161814	+		hypothetical protein
FUN_002496	contig_2	3163987	3164847	+		hypothetical protein
FUN_002497	contig_2	3164937	3165762	-		hypothetical protein
FUN_002498	contig_2	3166892	3167784	+		hypothetical protein
FUN_002499	contig_2	3167931	3169848	-	MAS2	Mitochondrial-processing peptidase subunit alpha
FUN_002500	contig_2	3170946	3172397	+		hypothetical protein
FUN_002502	contig_2	3174391	3175732	+		hypothetical protein
FUN_002503	contig_2	3176423	3177751	-		hypothetical protein
FUN_002504	contig_2	3179221	3180102	-		hypothetical protein
FUN_002506	contig_2	3183263	3185246	-		hypothetical protein
FUN_002508	contig_2	3196620	3197864	+		hypothetical protein
FUN_002509	contig_2	3198197	3199516	-		hypothetical protein
FUN_002511	contig_2	3201920	3205984	+		hypothetical protein
FUN_002512	contig_2	3206604	3208190	+		hypothetical protein
FUN_002513	contig_2	3212311	3213559	-	HEM3	porphobilinogen deaminase
FUN_002514	contig_2	3214986	3216999	-	fur4	uracil permease
FUN_002516	contig_2	3221621	3226090	+		hypothetical protein
FUN_002517	contig_2	3226639	3227315	+	rpl22	60S ribosomal protein L22
FUN_002518	contig_2	3227588	3228419	-		hypothetical protein
FUN_002520	contig_2	3232216	3233215	+		hypothetical protein
FUN_002521	contig_2	3234836	3236245	+		hypothetical protein
FUN_002523	contig_2	3239937	3241298	+	LDB19	Endocytosis regulator
FUN_002525	contig_2	3251580	3252757	-		hypothetical protein
FUN_002526	contig_2	3254290	3257842	-	CDC25	cell division cycle-related protein
FUN_002527	contig_2	3261024	3263649	+		hypothetical protein
FUN_002528	contig_2	3264688	3265457	+		hypothetical protein
FUN_002529	contig_2	3270667	3271862	-		hypothetical protein
FUN_002531	contig_2	3276050	3277162	-		hypothetical protein
FUN_002532	contig_2	3277568	3278982	+	PUS4	pseudouridine synthase pus4
FUN_002533	contig_2	3279359	3280888	-		hypothetical protein
FUN_002534	contig_2	3281425	3282091	-	YPT7	Rab GTPase ypt7
FUN_002535	contig_2	3284513	3285551	+		hypothetical protein
FUN_002536	contig_2	3286062	3287795	+		hypothetical protein
FUN_002538	contig_2	3293554	3295539	-		hypothetical protein
FUN_002539	contig_2	3297329	3301518	+		hypothetical protein
FUN_002540	contig_2	3301688	3302785	-		hypothetical protein
FUN_002541	contig_2	3305795	3307519	-		hypothetical protein
FUN_002542	contig_2	3308084	3308856	-	GPI11	Glycosylphosphatidylinositol (GPI) anchor assembly protein
FUN_002543	contig_2	3309450	3310455	+	tsn1	Translin-1
FUN_002545	contig_2	3313810	3315624	-		hypothetical protein
FUN_002546	contig_2	3320206	3322985	-		hypothetical protein
FUN_002547	contig_2	3324191	3325289	-	CHS7_1	Chitin synthase, class 7
FUN_002548	contig_2	3327377	3329052	-		hypothetical protein
FUN_002549	contig_2	3329348	3330528	+		hypothetical protein
FUN_002551	contig_2	3332955	3334523	+		hypothetical protein
FUN_002552	contig_2	3343419	3344496	+		hypothetical protein
FUN_002554	contig_2	3350679	3352833	+		hypothetical protein
FUN_002555	contig_2	3355046	3356746	-		hypothetical protein
FUN_002556	contig_2	3359450	3360642	+		hypothetical protein
FUN_002557	contig_2	3361807	3363156	-		hypothetical protein
FUN_002559	contig_2	3365304	3366827	-		hypothetical protein
FUN_002560	contig_2	3367603	3368934	+		hypothetical protein
FUN_002563	contig_2	3371981	3375429	-	RAD5	DNA helicase rad5
FUN_002564	contig_2	3376053	3377214	+		hypothetical protein
FUN_002565	contig_2	3377231	3378255	-		hypothetical protein
FUN_002567	contig_2	3387379	3388850	-	TUF1	translation elongation factor Tu
FUN_002568	contig_2	3389297	3390761	+	EHD3	3-hydroxyisobutyryl-CoA hydrolase

Gene ID	Scaffold	Start	Stop	Strand	Name	Product
FUN_002569	contig_2	3391090	3393887	+	vps16	Vacuolar protein sorting-associated protein 16
FUN_002570	contig_2	3394128	3396023	-		hypothetical protein
FUN_002571	contig_2	3396411	3399506	+		hypothetical protein
FUN_002572	contig_2	3400104	3401053	+	HSP31_1	plasma membrane heat shock protein
FUN_002576	contig_2	3407301	3408967	-		hypothetical protein
FUN_002577	contig_2	3410877	3411570	+		hypothetical protein
FUN_002578	contig_2	3413871	3416205	-		hypothetical protein
FUN_002580	contig_2	3426957	3428274	+		hypothetical protein
FUN_002582	contig_2	3431157	3432300	-		hypothetical protein
FUN_002584	contig_2	3438574	3439821	+		hypothetical protein
FUN_002585	contig_2	3440931	3442484	-		hypothetical protein
FUN_002586	contig_2	3443342	3444496	-		hypothetical protein
FUN_002587	contig_2	3446746	3448517	+		hypothetical protein
FUN_002588	contig_2	3448865	3450656	+		hypothetical protein
FUN_002589	contig_2	3450814	3452532	-		hypothetical protein
FUN_002594	contig_2	3468624	3470607	-		hypothetical protein
FUN_002595	contig_2	3470990	3473817	-		hypothetical protein
FUN_002598	contig_2	3478623	3479927	+		hypothetical protein
FUN_002599	contig_2	3480551	3481894	+	CAF17	ccr4 associated factor
FUN_002600	contig_2	3481971	3483239	-	BUD14_2	protein phosphatase regulator
FUN_002601	contig_2	3483900	3485546	-	PTM1	Membrane protein ptm1
FUN_002602	contig_2	3486007	3487170	+	CTU2	Cytoplasmic tRNA 2-thiolation protein 2
FUN_002603	contig_2	3488048	3488455	+		hypothetical protein
FUN_002604	contig_2	3488945	3489825	-		hypothetical protein
FUN_002605	contig_2	3490568	3493525	+	DCP2	mRNA-decapping enzyme subunit 2
FUN_002607	contig_2	3495150	3496508	-	RAD53_2	Protein kinase protein rad53
FUN_002608	contig_2	3496779	3497867	-		hypothetical protein
FUN_002611	contig_2	3503574	3504590	+		hypothetical protein
FUN_002613	contig_2	3506232	3507527	-		hypothetical protein
FUN_002614	contig_2	3507883	3510115	-		hypothetical protein
FUN_002615	contig_2	3513721	3514706	+		hypothetical protein
FUN_002617	contig_2	3521825	3523936	-		hypothetical protein
FUN_002619	contig_2	3529251	3530764	+		hypothetical protein
FUN_002620	contig_2	3534866	3538513	+		hypothetical protein
FUN_002621	contig_2	3538535	3539922	-		hypothetical protein
FUN_002622	contig_2	3541001	3542028	+		hypothetical protein
FUN_002623	contig_2	3543375	3545209	-	HXT4	fructose symporter
FUN_002624	contig_2	3550929	3551859	+		hypothetical protein
FUN_002625	contig_2	3552265	3553321	-		hypothetical protein
FUN_002626	contig_2	3554467	3555264	-		hypothetical protein
FUN_002627	contig_2	3555772	3556677	+		hypothetical protein
FUN_002628	contig_2	3556776	3557884	-		hypothetical protein
FUN_002629	contig_2	3558433	3560622	-		hypothetical protein
FUN_002631	contig_2	3563561	3564570	-		hypothetical protein
FUN_002632	contig_2	3565101	3568526	+	FAP1	FKBP12-associated protein
FUN_002633	contig_2	3568911	3570164	-		hypothetical protein
FUN_002635	contig_2	3572003	3573511	-		hypothetical protein
FUN_002636	contig_2	3573755	3574393	+		hypothetical protein
FUN_002637	contig_2	3575387	3576647	+	CHA1	catabolic L-serine/threonine dehydratase
FUN_002638	contig_2	3577295	3579961	-		hypothetical protein
FUN_002639	contig_2	3581102	3584710	-	SET1	histone methyltransferase set1
FUN_002640	contig_2	3586142	3586925	-	RIB4	lumazine synthase
FUN_002641	contig_2	3587404	3589765	-	GFA1	glutamine--fructose-6-phosphate transaminase (isomerizing)
FUN_002642	contig_2	3592581	3594713	-		hypothetical protein
FUN_002643	contig_2	3595285	3599363	+		hypothetical protein
FUN_002644	contig_2	3599530	3601101	-	MAK11	Protein mak11
FUN_002645	contig_2	3601423	3606194	+	eaf1	RNA polymerase II transcription elongation factor SpEAF

Gene ID	Scaffold	Start	Stop	Strand	Name	Product
FUN_002646	contig_2	3606836	3607832	+	RAM2	CAAX geranylgeranyltransferase alpha subunit
FUN_002647	contig_2	3608234	3609348	-		hypothetical protein
FUN_002648	contig_2	3610570	3611709	+	PEX10	peroxisome bioproteinsis factor 10
FUN_002652	contig_2	3620243	3621570	+	ERD1	protein-ER retention protein
FUN_002653	contig_2	3624631	3626674	-		hypothetical protein
FUN_002654	contig_2	3628120	3628892	+		hypothetical protein
FUN_002655	contig_2	3632539	3637035	+		hypothetical protein
FUN_002656	contig_2	3637526	3639081	-	DGA1	diacylglycerol O-acyltransferase 1
FUN_002657	contig_2	3639683	3640886	+		hypothetical protein
FUN_002658	contig_2	3642418	3643220	+		hypothetical protein
FUN_002661	contig_2	3647055	3648127	-		hypothetical protein
FUN_002662	contig_2	3648820	3650921	-		hypothetical protein
FUN_002663	contig_2	3652304	3653554	+		hypothetical protein
FUN_002664	contig_2	3654601	3655853	+		hypothetical protein
FUN_002665	contig_2	3657023	3658116	+		hypothetical protein
FUN_002667	contig_2	3666757	3668671	+		hypothetical protein
FUN_002668	contig_2	3670197	3670818	+		hypothetical protein
FUN_002669	contig_2	3671528	3672666	+		hypothetical protein
FUN_002673	contig_2	3677930	3678792	-	SVP26	erv26 super protein
FUN_002674	contig_2	3679069	3679911	+	BUD23	18S rRNA (guanine1575-N7)-methyltransferase
FUN_002675	contig_2	3680095	3681207	-	IDH1	isocitrate dehydrogenase (NAD(+)) idh1
FUN_002676	contig_2	3682133	3683100	+		hypothetical protein
FUN_002677	contig_2	3684652	3686648	+		hypothetical protein
FUN_002678	contig_2	3687833	3688373	+	RPL38	60S ribosomal protein L38
FUN_002679	contig_2	3688741	3689784	-		hypothetical protein
FUN_002681	contig_2	3692470	3694592	+	MNS1	mannosyl-oligosaccharide alpha-1,2-mannosidase
FUN_002683	contig_2	3697155	3698870	+		hypothetical protein
FUN_002684	contig_2	3699697	3701025	-		hypothetical protein
FUN_002686	contig_2	3704311	3704998	-		hypothetical protein
FUN_002687	contig_2	3706048	3708090	+		hypothetical protein
FUN_002689	contig_2	3710434	3711755	+		hypothetical protein
FUN_002690	contig_2	3711883	3713091	-		hypothetical protein
FUN_002693	contig_2	3715061	3716182	+	atg5	Autophagy protein 5
FUN_002694	contig_2	3716420	3719916	-	MSH3	Mismatch repair protein msh3
FUN_002695	contig_2	3720190	3721548	-		hypothetical protein
FUN_002697	contig_2	3722632	3723390	+		hypothetical protein
FUN_002698	contig_2	3723874	3726284	+		hypothetical protein
FUN_002699	contig_2	3727759	3728694	+		hypothetical protein
FUN_002700	contig_2	3732852	3734929	-		hypothetical protein
FUN_002701	contig_2	3738557	3739217	-	bem46	bem46 protein, variant
FUN_002702	contig_2	3739943	3740592	+	PRE3	Proteasome subunit beta type-1
FUN_002703	contig_2	3741696	3743116	+	PCL7	cyclin-like protein interacting with PHO85
FUN_002704	contig_2	3744167	3745253	+		hypothetical protein
FUN_002707	contig_2	3750160	3751019	+		hypothetical protein
FUN_002709	contig_2	3754005	3756239	+	PRP28	mRNA splicing protein prp28
FUN_002710	contig_2	3757032	3757665	-	PUP3	proteasome core particle subunit beta 3
FUN_002713	contig_2	3761160	3762245	+	PRE10	Putative proteasome subunit alpha type-7
FUN_002714	contig_2	3762517	3765301	+		hypothetical protein
FUN_002717	contig_2	3773873	3774201	+		hypothetical protein
FUN_002718	contig_2	3774296	3775075	+		hypothetical protein
FUN_002720	contig_2	3780518	3782002	+		hypothetical protein
FUN_002721	contig_2	3782429	3784621	-		hypothetical protein
FUN_002722	contig_2	3785266	3787132	-		hypothetical protein
FUN_002723	contig_2	3788199	3789475	+		hypothetical protein
FUN_002724	contig_2	3791778	3793761	+		hypothetical protein
FUN_002725	contig_2	3794204	3795536	+		hypothetical protein
FUN_002727	contig_2	3798571	3800253	-		hypothetical protein
FUN_002728	contig_2	3803047	3804399	+		hypothetical protein
FUN_002730	contig_2	3806794	3807933	-		hypothetical protein

Gene ID	Scaffold	Start	Stop	Strand	Name	Product
FUN_002731	contig_2	3808605	3809635	+		hypothetical protein
FUN_002733	contig_2	3812209	3814362	-		hypothetical protein
FUN_002734	contig_2	3820299	3823055	-		hypothetical protein
FUN_002735	contig_2	3824752	3826286	-		hypothetical protein
FUN_002736	contig_2	3827026	3828823	-		hypothetical protein
FUN_002737	contig_2	3829727	3831381	-		hypothetical protein
FUN_002738	contig_2	3831848	3833956	+		hypothetical protein
FUN_002739	contig_2	3834049	3835943	-		hypothetical protein
FUN_002740	contig_2	3836586	3837764	+		hypothetical protein
FUN_002741	contig_2	3838392	3844017	+		hypothetical protein
FUN_002744	contig_2	3846223	3847404	-		hypothetical protein
FUN_002745	contig_2	3847824	3849485	-		hypothetical protein
FUN_002748	contig_2	3859383	3861260	-		hypothetical protein
FUN_002749	contig_2	3864940	3866735	-		hypothetical protein
FUN_002752	contig_2	3880228	3881401	-	XYL1_1	NAD(P)H-dependent D-xylose reductase (XR)
FUN_002753	contig_2	3884703	3886214	+		hypothetical protein
FUN_002754	contig_2	3887964	3889010	-		hypothetical protein
FUN_002755	contig_2	3889957	3892001	+		hypothetical protein
FUN_002757	contig_2	3894545	3896146	-		hypothetical protein
FUN_002758	contig_2	3898619	3899102	+		hypothetical protein
FUN_002759	contig_2	3899840	3901862	-		hypothetical protein
FUN_002763	contig_2	3906769	3907336	-		hypothetical protein
FUN_002764	contig_2	3908470	3909426	-		hypothetical protein
FUN_002768	contig_2	3917320	3919026	-		hypothetical protein
FUN_002772	contig_2	3928090	3929642	+		hypothetical protein
FUN_002773	contig_2	3929847	3933121	-		hypothetical protein
FUN_002774	contig_2	3933445	3936612	-		hypothetical protein
FUN_002775	contig_2	3937488	3939277	+		hypothetical protein
FUN_002776	contig_2	3942990	3944923	+	INDA1	Amino-acid permease inda1
FUN_002778	contig_2	3946255	3947430	-		hypothetical protein
FUN_002779	contig_3	16870	18179	+		hypothetical protein
FUN_002781	contig_3	39559	40434	-		hypothetical protein
FUN_002782	contig_3	41812	42744	+		hypothetical protein
FUN_002783	contig_3	43885	44526	-		hypothetical protein
FUN_002784	contig_3	51737	53348	-		hypothetical protein
FUN_002786	contig_3	66403	67155	+	PCL1_1	PHO85 cyclin-1
FUN_002787	contig_3	69292	70101	-		hypothetical protein
FUN_002788	contig_3	78415	79125	-		hypothetical protein
FUN_002791	contig_3	100372	101723	-		hypothetical protein
FUN_002792	contig_3	105627	107678	-		hypothetical protein
FUN_002793	contig_3	112053	113042	-		hypothetical protein
FUN_002794	contig_3	113999	114739	+		hypothetical protein
FUN_002795	contig_3	132564	133489	+		hypothetical protein
FUN_002796	contig_3	134630	135399	-	AZF1_1	DNA-binding transcription factor
FUN_002800	contig_3	170308	171705	-		hypothetical protein
FUN_002802	contig_3	180716	182626	-		hypothetical protein
FUN_002803	contig_3	185270	185861	-	DCL2_1	Dicer-like protein 2
FUN_002804	contig_3	194045	195481	+		hypothetical protein
FUN_002807	contig_3	212736	213407	+		hypothetical protein
FUN_002810	contig_3	218743	219660	+		hypothetical protein
FUN_002812	contig_3	223877	225916	+		hypothetical protein
FUN_002813	contig_3	230064	230433	-		hypothetical protein
FUN_002814	contig_3	230927	232572	+		hypothetical protein
FUN_002815	contig_3	234856	235662	+		hypothetical protein
FUN_002816	contig_3	236059	236362	-		hypothetical protein
FUN_002817	contig_3	239057	240014	-		hypothetical protein
FUN_002818	contig_3	241818	242081	+		hypothetical protein
FUN_002820	contig_3	258901	259617	+		hypothetical protein
FUN_002821	contig_3	267815	270475	-		hypothetical protein

Gene ID	Scaffold	Start	Stop	Strand	Name	Product
FUN_002823	contig_3	275434	276615	-		hypothetical protein
FUN_002824	contig_3	279329	280171	+		hypothetical protein
FUN_002825	contig_3	304711	307001	-		hypothetical protein
FUN_002828	contig_3	318100	319338	-		hypothetical protein
FUN_002830	contig_3	353254	353977	-		hypothetical protein
FUN_002831	contig_3	362010	363113	-		hypothetical protein
FUN_002833	contig_3	371844	372215	-		hypothetical protein
FUN_002834	contig_3	372427	373143	+		hypothetical protein
FUN_002836	contig_3	383427	384824	-		hypothetical protein
FUN_002844	contig_3	432505	433677	+		hypothetical protein
FUN_002846	contig_3	439377	439865	+		hypothetical protein
FUN_002847	contig_3	443291	444323	+		hypothetical protein
FUN_002848	contig_3	448839	449336	+		hypothetical protein
FUN_002849	contig_3	454312	455106	+		hypothetical protein
FUN_002851	contig_3	465147	466037	-	DDX17	putative ATP-dependent RNA helicase ddx17
FUN_002853	contig_3	489406	490564	-		hypothetical protein
FUN_002854	contig_3	495904	496435	+		hypothetical protein
FUN_002855	contig_3	501774	502444	-		hypothetical protein
FUN_002856	contig_3	503642	504248	-		hypothetical protein
FUN_002857	contig_3	506053	506798	+		hypothetical protein
FUN_002858	contig_3	509155	509499	-		hypothetical protein
FUN_002860	contig_3	533049	533681	-		hypothetical protein
FUN_002863	contig_3	548060	548773	-		hypothetical protein
FUN_002864	contig_3	560342	564299	+		hypothetical protein
FUN_002865	contig_3	568173	570454	+		hypothetical protein
FUN_002868	contig_3	597291	597722	-		hypothetical protein
FUN_002869	contig_3	599420	600337	+		hypothetical protein
FUN_002870	contig_3	601797	602325	-		hypothetical protein
FUN_002871	contig_3	604239	605370	-		hypothetical protein
FUN_002872	contig_3	607040	610455	+		hypothetical protein
FUN_002875	contig_3	618162	619077	-		hypothetical protein
FUN_002876	contig_3	623554	624033	-		hypothetical protein
FUN_002877	contig_3	627123	629112	-		hypothetical protein
FUN_002878	contig_3	634890	635324	-		hypothetical protein
FUN_002883	contig_3	666292	667752	-		hypothetical protein
FUN_002884	contig_3	669435	670103	-		hypothetical protein
FUN_002885	contig_3	673661	674017	+	ERG4_1	C-24(28) sterol reductase
FUN_002887	contig_3	679123	679965	-		hypothetical protein
FUN_002888	contig_3	691293	692241	+		hypothetical protein
FUN_002889	contig_3	704555	705271	+	BLM3_1	Proteasome activator BLM10
FUN_002890	contig_3	707358	709715	-		hypothetical protein
FUN_002893	contig_3	731044	731298	+		hypothetical protein
FUN_002897	contig_3	787311	788019	-		hypothetical protein
FUN_002898	contig_3	804137	806573	-		hypothetical protein
FUN_002899	contig_3	808288	808761	+		hypothetical protein
FUN_002900	contig_3	809196	811859	+		hypothetical protein
FUN_002904	contig_3	844571	845070	+		hypothetical protein
FUN_002905	contig_3	862110	862655	-	MIF2_1	mitotic fidelity of chromosome transmission-related protein
FUN_002907	contig_3	866729	867478	-	RAD53_3	Protein kinase protein rad53
FUN_002909	contig_3	894512	895438	-		hypothetical protein
FUN_002910	contig_3	896589	897027	+	HPC2_1	HIR complex subunit
FUN_002911	contig_3	897740	900493	-		hypothetical protein
FUN_002912	contig_3	903285	906027	-		hypothetical protein
FUN_002913	contig_3	909648	910037	-		hypothetical protein
FUN_002915	contig_3	918104	920854	-		hypothetical protein
FUN_002916	contig_3	920942	921323	-		hypothetical protein
FUN_002917	contig_3	926972	928755	+		hypothetical protein
FUN_002918	contig_3	930217	931248	-		hypothetical protein

Gene ID	Scaffold	Start	Stop	Strand	Name	Product
FUN_002919	contig_3	931910	932836	+		hypothetical protein
FUN_002921	contig_3	948335	949680	-	PCL1_2	PHO85 cyclin-1
FUN_002922	contig_3	949867	950283	+		hypothetical protein
FUN_002923	contig_3	951533	952167	-		hypothetical protein
FUN_002924	contig_3	965704	966233	+		hypothetical protein
FUN_002925	contig_3	975210	975618	+		hypothetical protein
FUN_002926	contig_3	981535	983480	-		hypothetical protein
FUN_002927	contig_3	991957	994208	-		hypothetical protein
FUN_002930	contig_3	1026697	1032331	-	BLM3_2	Proteasome activator BLM10
FUN_002931	contig_3	1033965	1034748	-		hypothetical protein
FUN_002935	contig_3	1055869	1056849	-		hypothetical protein
FUN_002936	contig_3	1058465	1059334	+		hypothetical protein
FUN_002937	contig_3	1061315	1062125	-		hypothetical protein
FUN_002940	contig_3	1073927	1074563	-		hypothetical protein
FUN_002941	contig_3	1087291	1090375	-		hypothetical protein
FUN_002942	contig_3	1094729	1095372	+		hypothetical protein
FUN_002943	contig_3	1100230	1101821	+		hypothetical protein
FUN_002944	contig_3	1102087	1102941	-	BLM3_3	Proteasome activator BLM10
FUN_002945	contig_3	1108294	1108644	-		hypothetical protein
FUN_002946	contig_3	1112306	1113116	-		hypothetical protein
FUN_002948	contig_3	1115664	1117065	+	MET7_1	Folylpolyglutamate synthetase
FUN_002949	contig_3	1122293	1123157	+		hypothetical protein
FUN_002950	contig_3	1124103	1125245	-		hypothetical protein
FUN_002953	contig_3	1142920	1144564	-		hypothetical protein
FUN_002955	contig_3	1156165	1156857	+		hypothetical protein
FUN_002956	contig_3	1160317	1160933	+		hypothetical protein
FUN_002957	contig_3	1162767	1163414	+		hypothetical protein
FUN_002958	contig_3	1166525	1167065	+		hypothetical protein
FUN_002959	contig_3	1168915	1169643	-		hypothetical protein
FUN_002962	contig_3	1181337	1182618	+	PCL1_3	PHO85 cyclin-1
FUN_002963	contig_3	1187099	1187848	+	RAD53_4	Protein kinase protein rad53
FUN_002964	contig_3	1192409	1194027	-		hypothetical protein
FUN_002966	contig_3	1207422	1207654	+		hypothetical protein
FUN_002969	contig_3	1221918	1222241	-		hypothetical protein
FUN_002972	contig_3	1232754	1233386	+		hypothetical protein
FUN_002973	contig_3	1234098	1235776	+		hypothetical protein
FUN_002974	contig_3	1241847	1242458	+		hypothetical protein
FUN_002979	contig_3	1270315	1271552	-		hypothetical protein
FUN_002981	contig_3	1297286	1298076	-		hypothetical protein
FUN_002982	contig_3	1308797	1309513	-		hypothetical protein
FUN_002983	contig_3	1312507	1313812	+		hypothetical protein
FUN_002985	contig_3	1319942	1320935	+		hypothetical protein
FUN_002986	contig_3	1321140	1321841	-		hypothetical protein
FUN_002987	contig_3	1326386	1326944	+		hypothetical protein
FUN_002988	contig_3	1333558	1334621	-	KIN3_1	G2-specific serine/threonine protein kinase
FUN_002990	contig_3	1340358	1341976	+		hypothetical protein
FUN_002991	contig_3	1357045	1359474	+		hypothetical protein
FUN_002992	contig_3	1360810	1362615	+		hypothetical protein
FUN_002993	contig_3	1363432	1367551	+		hypothetical protein
FUN_002994	contig_3	1372097	1372468	+		hypothetical protein
FUN_002995	contig_3	1378134	1379338	+		hypothetical protein
FUN_002998	contig_3	1411596	1412347	+		hypothetical protein
FUN_002999	contig_3	1412968	1413421	+		hypothetical protein
FUN_003000	contig_3	1414362	1415147	-		hypothetical protein
FUN_003001	contig_3	1416313	1416573	-		hypothetical protein
FUN_003002	contig_3	1416633	1419709	-		hypothetical protein
FUN_003004	contig_3	1428982	1429372	+	SMT3_1	SUMO protein smt3
FUN_003005	contig_3	1449062	1449620	+		hypothetical protein
FUN_003008	contig_3	1463911	1465629	+		hypothetical protein

Gene ID	Scaffold	Start	Stop	Strand	Name	Product
FUN_003009	contig_3	1471484	1473407	-		hypothetical protein
FUN_003010	contig_3	1482255	1483430	+		hypothetical protein
FUN_003011	contig_3	1485299	1487194	+		hypothetical protein
FUN_003013	contig_3	1495311	1497660	+		hypothetical protein
FUN_003014	contig_3	1498623	1500989	-		hypothetical protein
FUN_003015	contig_3	1501582	1503580	-		hypothetical protein
FUN_003018	contig_3	1518148	1518774	+	HPC2_2	HIR complex subunit
FUN_003019	contig_3	1564051	1565769	-		hypothetical protein
FUN_003022	contig_3	1573678	1574100	+		hypothetical protein
FUN_003023	contig_3	1580084	1581279	+		hypothetical protein
FUN_003024	contig_3	1596979	1597521	-		hypothetical protein
FUN_003025	contig_3	1601735	1604695	+		hypothetical protein
FUN_003026	contig_3	1606997	1608302	-		hypothetical protein
FUN_003029	contig_3	1623119	1624011	-		hypothetical protein
FUN_003031	contig_3	1628731	1629016	+		hypothetical protein
FUN_003032	contig_3	1633001	1633387	+		hypothetical protein
FUN_003034	contig_3	1635152	1635871	+		hypothetical protein
FUN_003037	contig_3	1655216	1655774	+		hypothetical protein
FUN_003038	contig_3	1658833	1659623	-		hypothetical protein
FUN_003039	contig_3	1663732	1665261	+	UBA2_2	E1 ubiquitin-activating protein uba2
FUN_003040	contig_3	1667495	1668191	-		hypothetical protein
FUN_003041	contig_3	1672399	1674330	+		hypothetical protein
FUN_003042	contig_3	1674461	1675233	+		hypothetical protein
FUN_003047	contig_3	1694079	1694723	-		hypothetical protein
FUN_003048	contig_3	1728072	1728689	+		hypothetical protein
FUN_003049	contig_3	1729013	1729417	+		hypothetical protein
FUN_003051	contig_3	1756069	1756633	-		hypothetical protein
FUN_003052	contig_3	1757375	1758938	+	HPC2_3	HIR complex subunit
FUN_003053	contig_3	1759600	1760265	+		hypothetical protein
FUN_003054	contig_3	1769679	1770428	+	RAD53_5	Protein kinase protein rad53
FUN_003056	contig_3	1794426	1795672	-		hypothetical protein
FUN_003057	contig_3	1802850	1803017	+		hypothetical protein
FUN_003058	contig_3	1806403	1807614	-		hypothetical protein
FUN_003059	contig_3	1812599	1814217	-		hypothetical protein
FUN_003060	contig_3	1821637	1822080	+		hypothetical protein
FUN_003061	contig_3	1825502	1826844	-	STE23_1	metalloprotease
FUN_003062	contig_3	1828875	1829105	+		hypothetical protein
FUN_003066	contig_3	1842307	1843014	+		hypothetical protein
FUN_003067	contig_3	1849228	1850427	+		hypothetical protein
FUN_003069	contig_3	1857713	1858667	+		hypothetical protein
FUN_003071	contig_3	1869443	1870049	-		hypothetical protein
FUN_003074	contig_3	1890217	1893171	+		hypothetical protein
FUN_003077	contig_3	1907806	1908390	+		hypothetical protein
FUN_003078	contig_3	1912526	1913158	-		hypothetical protein
FUN_003079	contig_3	1915047	1915616	+		hypothetical protein
FUN_003081	contig_3	1921673	1922659	-		hypothetical protein
FUN_003083	contig_3	1936035	1937259	+		hypothetical protein
FUN_003084	contig_3	1937728	1938644	-		hypothetical protein
FUN_003086	contig_3	1941487	1942404	-		hypothetical protein
FUN_003089	contig_3	1975203	1976998	+		hypothetical protein
FUN_003091	contig_3	1988209	1988763	-	MIF2_2	mitotic fidelity of chromosome transmission-related protein
FUN_003093	contig_3	2011717	2012031	-		hypothetical protein
FUN_003094	contig_3	2015162	2015789	+		hypothetical protein
FUN_003095	contig_3	2016066	2016807	+		hypothetical protein
FUN_003096	contig_3	2021225	2022575	+		hypothetical protein
FUN_003099	contig_3	2045433	2046773	-		hypothetical protein
FUN_003100	contig_3	2053749	2055893	+		hypothetical protein
FUN_003101	contig_3	2057244	2057714	+		hypothetical protein

Gene ID	Scaffold	Start	Stop	Strand	Name	Product
FUN_003103	contig_3	2060093	2060662	-		hypothetical protein
FUN_003104	contig_3	2061329	2062393	-		hypothetical protein
FUN_003105	contig_3	2065454	2065690	+		hypothetical protein
FUN_003107	contig_3	2071340	2072329	-		hypothetical protein
FUN_003108	contig_3	2084600	2085821	+		hypothetical protein
FUN_003110	contig_3	2095445	2096720	-		hypothetical protein
FUN_003111	contig_3	2107945	2112017	+		hypothetical protein
FUN_003112	contig_3	2114119	2117800	-		hypothetical protein
FUN_003113	contig_3	2123930	2124958	+		hypothetical protein
FUN_003114	contig_3	2125611	2126251	-		hypothetical protein
FUN_003115	contig_3	2126906	2127329	-	HPC2_4	HIR complex subunit
FUN_003116	contig_3	2130870	2131728	-		hypothetical protein
FUN_003120	contig_3	2163514	2163855	+		hypothetical protein
FUN_003122	contig_3	2173613	2174265	-		hypothetical protein
FUN_003123	contig_3	2181097	2182116	-	PCL1_4	PHO85 cyclin-1
FUN_003124	contig_3	2182318	2183719	-	MET7_2	Folylpolyglutamate synthetase
FUN_003126	contig_3	2189441	2190313	-		hypothetical protein
FUN_003127	contig_3	2200162	2202381	+		hypothetical protein
FUN_003129	contig_3	2220404	2220958	-		hypothetical protein
FUN_003130	contig_3	2221920	2223820	+		hypothetical protein
FUN_003134	contig_4	12618	13559	+		hypothetical protein
FUN_003135	contig_4	14352	15701	+		hypothetical protein
FUN_003136	contig_4	16479	17485	-		hypothetical protein
FUN_003138	contig_4	23424	24026	-		hypothetical protein
FUN_003139	contig_4	25649	27685	+		hypothetical protein
FUN_003140	contig_4	28465	29457	+		hypothetical protein
FUN_003144	contig_4	46260	47718	+		hypothetical protein
FUN_003146	contig_4	51083	53482	-		hypothetical protein
FUN_003147	contig_4	54238	55255	+	MEU1	S-methyl-5-thioadenosine phosphorylase
FUN_003149	contig_4	58903	59482	+		hypothetical protein
FUN_003150	contig_4	62200	63217	+		hypothetical protein
FUN_003151	contig_4	63348	64556	-		hypothetical protein
FUN_003153	contig_4	68177	70478	+		hypothetical protein
FUN_003154	contig_4	73185	75181	-		hypothetical protein
FUN_003155	contig_4	77839	79469	+		hypothetical protein
FUN_003156	contig_4	80517	82227	-		hypothetical protein
FUN_003157	contig_4	82778	83865	-		hypothetical protein
FUN_003159	contig_4	88398	89579	-		hypothetical protein
FUN_003160	contig_4	96859	98546	+		hypothetical protein
FUN_003162	contig_4	100643	102100	-		hypothetical protein
FUN_003163	contig_4	102879	104515	-	BFR2	rRNA-processing protein bfr2
FUN_003164	contig_4	104704	105826	+		hypothetical protein
FUN_003168	contig_4	116487	119560	-		hypothetical protein
FUN_003169	contig_4	120245	121843	-		hypothetical protein
FUN_003171	contig_4	128918	130406	+		hypothetical protein
FUN_003172	contig_4	131990	132953	+		hypothetical protein
FUN_003173	contig_4	133573	135213	-		hypothetical protein
FUN_003174	contig_4	135896	136924	-		hypothetical protein
FUN_003176	contig_4	144022	145068	+		hypothetical protein
FUN_003177	contig_4	145299	146629	+		hypothetical protein
FUN_003178	contig_4	146935	148094	-		hypothetical protein
FUN_003179	contig_4	154976	156291	-		hypothetical protein
FUN_003180	contig_4	161618	162957	+		hypothetical protein
FUN_003182	contig_4	166665	167819	-		hypothetical protein
FUN_003183	contig_4	169734	170712	-		hypothetical protein
FUN_003184	contig_4	173761	175939	-		hypothetical protein
FUN_003185	contig_4	176592	178130	-		hypothetical protein
FUN_003186	contig_4	178981	180846	+		hypothetical protein
FUN_003187	contig_4	183371	184993	-	DBF2_1	serine/threonine-protein kinase dbf2

Gene ID	Scaffold	Start	Stop	Strand	Name	Product
FUN_003188	contig_4	185736	187433	-		hypothetical protein
FUN_003189	contig_4	188116	188847	-		hypothetical protein
FUN_003190	contig_4	189573	192512	-		hypothetical protein
FUN_003191	contig_4	193838	195814	-	GEM1	ERMES complex Ca(2+)-binding regulatory GTPase gem1
FUN_003192	contig_4	196598	198656	+		hypothetical protein
FUN_003193	contig_4	200167	202026	+		hypothetical protein
FUN_003194	contig_4	204697	205986	-	CDC28_1	Cyclin-dependent kinase catalytic subunit
FUN_003195	contig_4	206505	207639	-	CDC28_2	Cyclin-dependent kinase catalytic subunit
FUN_003196	contig_4	208067	209232	+		hypothetical protein
FUN_003197	contig_4	209997	211031	-	saf4_1	Protein saf4
FUN_003198	contig_4	211894	214285	-	SUP35	translation termination factor GTPase eRF3
FUN_003199	contig_4	214831	217238	+	YRB30	Ran-specific GTPase-activating protein 30
FUN_003200	contig_4	217516	218886	-		hypothetical protein
FUN_003201	contig_4	219471	220112	-	TSR2	rRNA accumulation-related protein
FUN_003203	contig_4	222764	224608	+		hypothetical protein
FUN_003204	contig_4	225385	226710	+		hypothetical protein
FUN_003206	contig_4	230724	232223	-		hypothetical protein
FUN_003207	contig_4	233071	233958	+		hypothetical protein
FUN_003208	contig_4	234813	236750	+		hypothetical protein
FUN_003209	contig_4	237858	238332	+		hypothetical protein
FUN_003211	contig_4	243646	245276	+		hypothetical protein
FUN_003212	contig_4	250419	251074	-		hypothetical protein
FUN_003213	contig_4	251842	254104	+		hypothetical protein
FUN_003214	contig_4	258010	258755	-		hypothetical protein
FUN_003216	contig_4	262701	264517	-		hypothetical protein
FUN_003218	contig_4	267867	269258	+		hypothetical protein
FUN_003219	contig_4	272616	274100	+		hypothetical protein
FUN_003221	contig_4	279533	280203	+		hypothetical protein
FUN_003222	contig_4	280704	282746	+		hypothetical protein
FUN_003223	contig_4	283211	284580	-		hypothetical protein
FUN_003224	contig_4	285268	286879	-	CBH1	Exoglucanase 1
FUN_003225	contig_4	315009	316069	+		hypothetical protein
FUN_003226	contig_4	316226	318892	-		hypothetical protein
FUN_003227	contig_4	325874	328161	-		hypothetical protein
FUN_003228	contig_4	329167	332706	+		hypothetical protein
FUN_003229	contig_4	333347	337241	+	NCR1	niemann-Pick type C-related protein 1
FUN_003231	contig_4	357794	359073	+		hypothetical protein
FUN_003242	contig_4	394605	395129	+		hypothetical protein
FUN_003244	contig_4	399584	402265	+		hypothetical protein
FUN_003246	contig_4	404898	406618	+		hypothetical protein
FUN_003259	contig_4	427869	430683	+		hypothetical protein
FUN_003261	contig_4	434123	434659	+		hypothetical protein
FUN_003263	contig_4	436784	438816	-		hypothetical protein
FUN_003264	contig_4	439754	440413	-		hypothetical protein
FUN_003265	contig_4	440933	442618	-	gel4_1	1 3-beta-glucanosyltransferase gel4
FUN_003266	contig_4	444098	446143	-		hypothetical protein
FUN_003268	contig_4	449405	450581	-		hypothetical protein
FUN_003269	contig_4	454080	456120	+	KCH1	Potassium transporter
FUN_003271	contig_4	462425	464669	-	KAR2	ATPase with role in protein import into the ER
FUN_003272	contig_4	465822	467269	-	OYE32_2	NADH-dependent flavin oxidoreductase
FUN_003274	contig_4	470353	472162	-		hypothetical protein
FUN_003276	contig_4	475048	476809	-		hypothetical protein
FUN_003277	contig_4	477509	478639	-	TRM7	tRNA (uridine-2'-O-)-methyltransferase trm7
FUN_003278	contig_4	478939	481194	+	MAK5	ATP-dependent RNA helicase
FUN_003279	contig_4	481280	483072	-	GPI16	Subunit of the glycosylphosphatidylinositol transamidase complex-like protein
FUN_003280	contig_4	483489	484495	+	LUC7	splicing factor

Gene ID	Scaffold	Start	Stop	Strand	Name	Product
FUN_003281	contig_4	489528	490410	+	MIC33	Putative mitochondrial 2-oxoglutarate/malate carrier protein
FUN_003282	contig_4	491522	494025	+		hypothetical protein
FUN_003285	contig_4	503114	505669	+		hypothetical protein
FUN_003286	contig_4	506147	508039	-		hypothetical protein
FUN_003287	contig_4	508506	511570	-	CAP1	F-actin-capping protein subunit alpha
FUN_003290	contig_4	517048	518665	+		hypothetical protein
FUN_003292	contig_4	522045	523231	-		hypothetical protein
FUN_003293	contig_4	524483	528538	-		hypothetical protein
FUN_003294	contig_4	529448	531529	-	HSE1	ESCRT-0 subunit protein hse1
FUN_003295	contig_4	532648	535706	-	STE20	signal transducing kinase of the PAK
FUN_003296	contig_4	539771	544381	-		hypothetical protein
FUN_003297	contig_4	545853	547592	+		hypothetical protein
FUN_003298	contig_4	548493	549287	-		hypothetical protein
FUN_003299	contig_4	550094	552424	-	ENP2	Small ribosomal subunit bioproteinsis
FUN_003300	contig_4	552971	557939	+	SSN2	mediator of RNA polymerase II transcription subunit 13
FUN_003302	contig_4	559096	560424	-	SWF1	palmitoyltransferase swf1
FUN_003303	contig_4	560718	563325	+		hypothetical protein
FUN_003304	contig_4	565577	567090	-		hypothetical protein
FUN_003305	contig_4	568041	569754	+		hypothetical protein
FUN_003308	contig_4	574628	575158	-		hypothetical protein
FUN_003309	contig_4	575555	577105	+		hypothetical protein
FUN_003311	contig_4	579519	580949	-		hypothetical protein
FUN_003312	contig_4	583911	585620	-		hypothetical protein
FUN_003314	contig_4	588648	590560	+		hypothetical protein
FUN_003316	contig_4	592676	594430	+		hypothetical protein
FUN_003318	contig_4	596433	596936	+		hypothetical protein
FUN_003319	contig_4	597496	598046	+		hypothetical protein
FUN_003320	contig_4	599293	599919	+		hypothetical protein
FUN_003322	contig_4	601967	602248	-		hypothetical protein
FUN_003323	contig_4	602607	603847	+		hypothetical protein
FUN_003326	contig_4	609539	611113	+		hypothetical protein
FUN_003327	contig_4	612071	613812	+		hypothetical protein
FUN_003328	contig_4	614366	615728	+		hypothetical protein
FUN_003330	contig_4	623689	626421	-		hypothetical protein
FUN_003331	contig_4	628269	629646	+		hypothetical protein
FUN_003333	contig_4	642765	644685	-	HXT5_2	hexose transporter hxt5
FUN_003335	contig_4	648079	648691	+		hypothetical protein
FUN_003336	contig_4	651723	652701	-		hypothetical protein
FUN_003337	contig_4	653299	656108	+	CCT5	T-complex protein 1 subunit epsilon
FUN_003338	contig_4	662063	664038	-		hypothetical protein
FUN_003339	contig_4	665738	667542	-		hypothetical protein
FUN_003340	contig_4	673037	674134	+		hypothetical protein
FUN_003341	contig_4	683860	685036	-		hypothetical protein
FUN_003342	contig_4	685228	687198	-		hypothetical protein
FUN_003343	contig_4	687737	688677	+		hypothetical protein
FUN_003344	contig_4	689211	691092	-		hypothetical protein
FUN_003346	contig_4	697419	699149	-		hypothetical protein
FUN_003348	contig_4	701675	703107	-		hypothetical protein
FUN_003350	contig_4	705885	708265	+		hypothetical protein
FUN_003351	contig_4	708647	710629	+		hypothetical protein
FUN_003352	contig_4	713507	714618	+		hypothetical protein
FUN_003353	contig_4	714975	717581	+		hypothetical protein
FUN_003354	contig_4	717674	718638	-		hypothetical protein
FUN_003357	contig_4	722357	723711	+		hypothetical protein
FUN_003358	contig_4	724003	725289	-		hypothetical protein
FUN_003359	contig_4	726002	727426	-		hypothetical protein
FUN_003360	contig_4	731305	732392	-		hypothetical protein
FUN_003361	contig_4	735412	737317	+		hypothetical protein

Gene ID	Scaffold	Start	Stop	Strand	Name	Product
FUN_003363	contig_4	739925	741145	+		hypothetical protein
FUN_003367	contig_4	747863	749714	-		hypothetical protein
FUN_003369	contig_4	756615	757904	-		hypothetical protein
FUN_003370	contig_4	759242	761323	+		hypothetical protein
FUN_003372	contig_4	763297	764941	-	VPS27	Vacuolar protein-sorting-associated protein 27
FUN_003373	contig_4	765479	768823	+	DOA4	ubiquitin-specific protease doa4
FUN_003374	contig_4	769504	770963	+	ARG1	argininosuccinate synthetase
FUN_003376	contig_4	779957	781908	-		hypothetical protein
FUN_003377	contig_4	782721	784472	+		hypothetical protein
FUN_003378	contig_4	784593	785491	-		hypothetical protein
FUN_003379	contig_4	786026	787148	-		hypothetical protein
FUN_003380	contig_4	789310	790918	-		hypothetical protein
FUN_003382	contig_4	794681	797649	-		hypothetical protein
FUN_003384	contig_4	801914	803845	-	gla1	glycoside hydrolase 15 protein
FUN_003385	contig_4	806139	807229	+	RFWD3	RING finger and WD repeat domain-containing protein 3
FUN_003388	contig_4	814703	823516	+		hypothetical protein
FUN_003390	contig_4	830286	831301	-		hypothetical protein
FUN_003391	contig_4	832107	833717	+		hypothetical protein
FUN_003392	contig_4	834924	835325	+		hypothetical protein
FUN_003394	contig_4	838137	839341	+		hypothetical protein
FUN_003395	contig_4	840038	841444	+		hypothetical protein
FUN_003396	contig_4	842931	843833	-	PTR2_2	peptide transporter ptr2
FUN_003397	contig_4	844482	845473	-		hypothetical protein
FUN_003398	contig_4	850788	853702	+		hypothetical protein
FUN_003400	contig_4	856085	857968	+		hypothetical protein
FUN_003401	contig_4	858241	858874	-		hypothetical protein
FUN_003402	contig_4	859320	860600	-		hypothetical protein
FUN_003403	contig_4	861701	862396	+		hypothetical protein
FUN_003404	contig_4	866793	867512	+		hypothetical protein
FUN_003405	contig_4	867683	870022	-		hypothetical protein
FUN_003406	contig_4	871578	873680	-		hypothetical protein
FUN_003408	contig_4	882488	883884	+		hypothetical protein
FUN_003409	contig_4	888487	890064	+		hypothetical protein
FUN_003410	contig_4	890507	891547	-	RPS0	structural constituent of ribosome
FUN_003411	contig_4	891860	892844	+		hypothetical protein
FUN_003412	contig_4	893342	896341	-		hypothetical protein
FUN_003413	contig_4	897312	898331	+	AAH1_2	adenine deaminase
FUN_003414	contig_4	899194	900273	-		hypothetical protein
FUN_003416	contig_4	903083	903494	+		hypothetical protein
FUN_003419	contig_4	910853	911564	+		hypothetical protein
FUN_003420	contig_4	911847	913885	-	NOP58	Nucleolar protein 58
FUN_003421	contig_4	914239	915942	+		hypothetical protein
FUN_003423	contig_4	927209	928372	+		hypothetical protein
FUN_003424	contig_4	928869	930780	+		hypothetical protein
FUN_003425	contig_4	933488	934353	+		hypothetical protein
FUN_003426	contig_4	934702	936049	-		hypothetical protein
FUN_003428	contig_4	938616	939361	-		hypothetical protein
FUN_003430	contig_4	942384	947297	+	ARO1	3-dehydroquinate dehydratase (3-dehydroquinase)
FUN_003432	contig_4	949846	950842	+	PMU1_1	putative phosphoglycerate mutase pmu1
FUN_003433	contig_4	951763	953294	+	IDP1	Isocitrate dehydrogenase [NADP], mitochondrial precursor (Oxalosuccinate decarboxylase)
FUN_003435	contig_4	956135	956773	+	MBF1	multiprotein-bridging factor 1
FUN_003437	contig_4	962132	965096	-	ADE3	tetrahydrofolate synthase
FUN_003439	contig_4	967857	968961	+	TIF34	translation initiation factor eIF3 subunit
FUN_003440	contig_4	969257	971635	-	NTH1_1	alpha,alpha-trehalase nth1
FUN_003442	contig_4	976360	977419	+		hypothetical protein
FUN_003445	contig_4	991675	995007	-		hypothetical protein
FUN_003447	contig_4	1007045	1016769	-		hypothetical protein
FUN_003448	contig_4	1018774	1020560	+		hypothetical protein

Gene ID	Scaffold	Start	Stop	Strand	Name	Product
FUN_003449	contig_4	1020736	1022289	-		hypothetical protein
FUN_003450	contig_4	1023444	1024892	-		hypothetical protein
FUN_003452	contig_4	1027036	1029088	-		hypothetical protein
FUN_003453	contig_4	1029652	1031679	-	GUF1	Translation factor guf1 mitochondrial
FUN_003454	contig_4	1032021	1032997	+	MAD2	Mitotic spindle checkpoint component mad2
FUN_003455	contig_4	1034151	1036895	+		hypothetical protein
FUN_003456	contig_4	1037851	1042308	-	RPO31	DNA-directed RNA polymerase III subunit C1 (rpo31)
FUN_003457	contig_4	1042862	1044913	+		hypothetical protein
FUN_003459	contig_4	1046856	1047904	-		hypothetical protein
FUN_003460	contig_4	1049236	1050964	-		hypothetical protein
FUN_003461	contig_4	1051699	1054137	+		hypothetical protein
FUN_003462	contig_4	1054412	1056724	-		hypothetical protein
FUN_003463	contig_4	1057123	1057816	+		hypothetical protein
FUN_003465	contig_4	1059374	1060268	-		hypothetical protein
FUN_003466	contig_4	1060992	1061774	+		hypothetical protein
FUN_003467	contig_4	1062053	1063995	-		hypothetical protein
FUN_003468	contig_4	1066684	1068809	-		hypothetical protein
FUN_003469	contig_4	1071228	1071711	+	MSRB5	Peptide methionine sulfoxide reductase B5
FUN_003473	contig_4	1078017	1079570	-		hypothetical protein
FUN_003474	contig_4	1084146	1084537	+		hypothetical protein
FUN_003475	contig_4	1085842	1086904	-		hypothetical protein
FUN_003479	contig_4	1100198	1103406	+		hypothetical protein
FUN_003482	contig_4	1106506	1108633	-		hypothetical protein
FUN_003483	contig_4	1109054	1110895	+		hypothetical protein
FUN_003484	contig_4	1111837	1112873	-		hypothetical protein
FUN_003485	contig_4	1114266	1115217	-		hypothetical protein
FUN_003486	contig_4	1116142	1118518	+	MAM3	cell agglutination protein Mam3
FUN_003487	contig_4	1119272	1120882	+		hypothetical protein
FUN_003488	contig_4	1121497	1122915	+		hypothetical protein
FUN_003489	contig_4	1129655	1131650	+	ERT1	Transcriptional regulator of nonfermentable carbon utilization
FUN_003490	contig_4	1132276	1133141	-	FMN1	riboflavin kinase
FUN_003491	contig_4	1133499	1134496	+		hypothetical protein
FUN_003492	contig_4	1134917	1138704	+		hypothetical protein
FUN_003493	contig_4	1139183	1141002	-		hypothetical protein
FUN_003494	contig_4	1141242	1142153	+	PRE9	Proteasome subunit alpha type-3
FUN_003496	contig_4	1145179	1147769	-		hypothetical protein
FUN_003497	contig_4	1149955	1152639	-	BYE1	Transcription factor bye1
FUN_003499	contig_4	1154603	1156513	+	AGE2	ARF GAP with effector function(s)
FUN_003500	contig_4	1156853	1157229	-		hypothetical protein
FUN_003501	contig_4	1157795	1159957	-		hypothetical protein
FUN_003505	contig_4	1169050	1172286	-	MET10	sulfite reductase [NADPH] flavoprotein component
FUN_003506	contig_4	1174105	1175525	+		hypothetical protein
FUN_003507	contig_4	1177461	1178201	-	FMP52	Protein fmp52, mitochondrial
FUN_003508	contig_4	1179065	1180930	-		hypothetical protein
FUN_003509	contig_4	1181570	1182974	-	GLO1	Lactoylglutathione lyase
FUN_003511	contig_4	1184988	1186646	-		hypothetical protein
FUN_003513	contig_4	1188563	1189834	-		hypothetical protein
FUN_003514	contig_4	1191553	1192620	+	GST2_1	Glutathione S-transferase 2
FUN_003515	contig_4	1192820	1194332	-		hypothetical protein
FUN_003516	contig_4	1195672	1196073	+		hypothetical protein
FUN_003518	contig_4	1209277	1210687	+		hypothetical protein
FUN_003520	contig_4	1228030	1229883	-		hypothetical protein
FUN_003521	contig_4	1230440	1233225	+		hypothetical protein
FUN_003522	contig_4	1233322	1234870	-		hypothetical protein
FUN_003523	contig_4	1237859	1239584	-		hypothetical protein
FUN_003524	contig_4	1241114	1243053	-		hypothetical protein
FUN_003525	contig_4	1243772	1245996	-		hypothetical protein
FUN_003526	contig_4	1247326	1248868	-	COX15	Cytochrome c oxidase assembly protein cox15

Gene ID	Scaffold	Start	Stop	Strand	Name	Product
FUN_003527	contig_4	1249332	1252289	+		hypothetical protein
FUN_003532	contig_4	1276738	1277787	+		hypothetical protein
FUN_003533	contig_4	1278897	1280512	+		hypothetical protein
FUN_003534	contig_4	1280925	1281935	+		hypothetical protein
FUN_003535	contig_4	1282561	1284612	+		hypothetical protein
FUN_003536	contig_4	1284776	1286358	-		hypothetical protein
FUN_003537	contig_4	1286877	1287731	-	MED7	Mediator of RNA polymerase II transcription subunit 7
FUN_003538	contig_4	1288317	1289401	+	ACTTS2	Trans-enoyl reductase actts2
FUN_003540	contig_4	1292787	1294438	-		hypothetical protein
FUN_003541	contig_4	1295185	1301017	-	UFD4	Ubiquitin fusion degradation protein 4
FUN_003542	contig_4	1302669	1303544	+	NMA1	Nicotinamide/nicotinic acid mononucleotide adenylyltransferase 1
FUN_003543	contig_4	1304496	1307032	+		hypothetical protein
FUN_003546	contig_4	1310714	1311794	+		hypothetical protein
FUN_003547	contig_4	1314056	1315759	+	GIT2	glycerophosphoinositol permease
FUN_003549	contig_4	1321338	1322229	-	PEX11	Peroxisomal membrane protein PMP27
FUN_003550	contig_4	1322649	1324040	-	ABD1	mRNA cap guanine-N7 methyltransferase
FUN_003551	contig_4	1324401	1325956	+	RPN7	proteasome regulatory particle subunit
FUN_003552	contig_4	1326776	1328339	+	ALT1	alanine transaminase
FUN_003553	contig_4	1328977	1330396	+		hypothetical protein
FUN_003554	contig_4	1331938	1332817	+		hypothetical protein
FUN_003558	contig_4	1341596	1344500	+		hypothetical protein
FUN_003560	contig_4	1346263	1346698	+		hypothetical protein
FUN_003561	contig_4	1350090	1352327	+	PHM7_1	phosphate metabolism protein 7
FUN_003562	contig_4	1354243	1356027	-	MPS1	Serine/threonine kinase mps1
FUN_003563	contig_4	1356994	1359169	-		hypothetical protein
FUN_003570	contig_4	1376600	1377964	+		hypothetical protein
FUN_003571	contig_4	1378109	1378811	-		hypothetical protein
FUN_003572	contig_4	1379776	1380660	+		hypothetical protein
FUN_003573	contig_4	1381270	1381943	+		hypothetical protein
FUN_003575	contig_4	1387519	1388985	-		hypothetical protein
FUN_003576	contig_4	1390877	1392200	-		hypothetical protein
FUN_003577	contig_4	1395976	1397549	+		hypothetical protein
FUN_003578	contig_4	1399291	1402130	-		hypothetical protein
FUN_003579	contig_4	1402598	1403782	+		hypothetical protein
FUN_003580	contig_4	1404481	1406383	-		hypothetical protein
FUN_003581	contig_4	1407079	1408936	-		hypothetical protein
FUN_003582	contig_4	1409359	1410184	+		hypothetical protein
FUN_003584	contig_4	1413469	1414575	-	RDH1_1	short-chain alcohol dehydrogenase
FUN_003586	contig_4	1417479	1419542	+		hypothetical protein
FUN_003590	contig_4	1427607	1428853	+	RRD1	Serine/threonine-protein phosphatase 2A activator 1
FUN_003591	contig_4	1430113	1430880	+		hypothetical protein
FUN_003592	contig_4	1431635	1434617	+		hypothetical protein
FUN_003593	contig_4	1434820	1438322	-		hypothetical protein
FUN_003594	contig_4	1438863	1440710	-		hypothetical protein
FUN_003595	contig_4	1441042	1442144	+	TFB3	TFIIH/NER complex subunit
FUN_003598	contig_4	1449215	1451029	+		hypothetical protein
FUN_003600	contig_4	1453225	1454958	-		hypothetical protein
FUN_003601	contig_4	1455856	1456772	+		hypothetical protein
FUN_003602	contig_4	1456824	1457774	-	OTU2	OTU protein
FUN_003604	contig_4	1459649	1461353	-		hypothetical protein
FUN_003606	contig_4	1465525	1466912	-		hypothetical protein
FUN_003607	contig_4	1468851	1470389	-		hypothetical protein
FUN_003608	contig_4	1470701	1472469	-		hypothetical protein
FUN_003610	contig_4	1477140	1478096	-		hypothetical protein
FUN_003611	contig_4	1483142	1485688	-		hypothetical protein
FUN_003612	contig_4	1487380	1492908	-	SPO14	Phospholipase D1
FUN_003615	contig_4	1500037	1501086	+		hypothetical protein
FUN_003616	contig_4	1503110	1504695	+		hypothetical protein

Gene ID	Scaffold	Start	Stop	Strand	Name	Product
FUN_003618	contig_4	1512144	1513514	-		hypothetical protein
FUN_003619	contig_4	1515493	1517397	+	CRP1	Cruciform DNA binding protein
FUN_003622	contig_4	1522548	1523795	+	VMA6	H(+)-transporting V0 sector ATPase subunit d
FUN_003623	contig_4	1524939	1525709	+		hypothetical protein
FUN_003624	contig_4	1525978	1527753	-	DEG1	pseudouridine synthase deg1
FUN_003625	contig_4	1528479	1532070	+		hypothetical protein
FUN_003626	contig_4	1532848	1534297	+	PRS5	ribose-phosphate pyrophosphokinase
FUN_003627	contig_4	1535079	1536366	-		hypothetical protein
FUN_003628	contig_4	1539480	1540724	-		hypothetical protein
FUN_003629	contig_4	1541111	1542915	-	ICL1	isocitrate lyase 1
FUN_003630	contig_4	1544098	1545957	-		hypothetical protein
FUN_003631	contig_4	1546659	1548709	-		hypothetical protein
FUN_003634	contig_4	1554799	1558872	+	BUD2	GTPase activating factor
FUN_003635	contig_4	1560710	1562207	-	CPR6	peptidyl-prolyl cis-trans isomerase cpr6
FUN_003636	contig_4	1562892	1564182	-		hypothetical protein
FUN_003637	contig_4	1568427	1570460	-	NOT5	proteinral negative regulator of transcription subunit 5
FUN_003638	contig_4	1570957	1571723	+	DTD1	D-tyrosyl-tRNA(Tyr) deacylase
FUN_003639	contig_4	1573378	1574167	-	SPT10_1	Protein spt10
FUN_003640	contig_4	1574528	1575975	-		hypothetical protein
FUN_003641	contig_4	1576523	1577393	-		hypothetical protein
FUN_003642	contig_4	1578994	1580283	+		hypothetical protein
FUN_003643	contig_4	1580878	1582658	-		hypothetical protein
FUN_003644	contig_4	1582998	1584088	+	SCL1	Proteasome subunit YC7alpha/Y8 (protease yscE subunit 7)
FUN_003647	contig_4	1600625	1601758	+		hypothetical protein
FUN_003648	contig_4	1601819	1603422	-		hypothetical protein
FUN_003649	contig_4	1605086	1605538	-		hypothetical protein
FUN_003650	contig_4	1605848	1606869	-		hypothetical protein
FUN_003651	contig_4	1607556	1608301	+		hypothetical protein
FUN_003652	contig_4	1608693	1609578	+		hypothetical protein
FUN_003653	contig_4	1609823	1611457	-		hypothetical protein
FUN_003654	contig_4	1612160	1613241	+		hypothetical protein
FUN_003657	contig_4	1615958	1617590	+	HFD1	Hexadecenal dehydrogenase
FUN_003659	contig_4	1619851	1621378	-		hypothetical protein
FUN_003660	contig_4	1622083	1623486	+		hypothetical protein
FUN_003661	contig_4	1623886	1625049	-		hypothetical protein
FUN_003662	contig_4	1625385	1627005	+		hypothetical protein
FUN_003663	contig_4	1627306	1628190	-		hypothetical protein
FUN_003664	contig_4	1630794	1637265	-		hypothetical protein
FUN_003665	contig_4	1638996	1645272	+		hypothetical protein
FUN_003668	contig_4	1650937	1652742	-		hypothetical protein
FUN_003669	contig_4	1653182	1653781	+		hypothetical protein
FUN_003670	contig_4	1657318	1659267	-		hypothetical protein
FUN_003671	contig_4	1661003	1663277	+		hypothetical protein
FUN_003672	contig_4	1664887	1668111	-		hypothetical protein
FUN_003673	contig_4	1668196	1669726	+		hypothetical protein
FUN_003676	contig_4	1678315	1680398	-		hypothetical protein
FUN_003677	contig_4	1683207	1684255	+		hypothetical protein
FUN_003680	contig_4	1689304	1690629	+		hypothetical protein
FUN_003681	contig_4	1695309	1696755	-		hypothetical protein
FUN_003682	contig_4	1699227	1700496	+		hypothetical protein
FUN_003683	contig_4	1701640	1703943	+		hypothetical protein
FUN_003684	contig_4	1707960	1708987	+		hypothetical protein
FUN_003686	contig_4	1711380	1713265	-		hypothetical protein
FUN_003689	contig_4	1717528	1719098	-		hypothetical protein
FUN_003690	contig_4	1722399	1724768	+		hypothetical protein
FUN_003691	contig_4	1725013	1726157	+		hypothetical protein
FUN_003693	contig_4	1728750	1729752	-		hypothetical protein
FUN_003695	contig_4	1738033	1741002	-		hypothetical protein

Gene ID	Scaffold	Start	Stop	Strand	Name	Product
FUN_003697	contig_4	1742714	1743604	-		hypothetical protein
FUN_003698	contig_4	1744452	1745699	+		hypothetical protein
FUN_003699	contig_4	1746208	1747362	+		hypothetical protein
FUN_003701	contig_4	1750733	1751960	-		hypothetical protein
FUN_003703	contig_4	1755330	1757128	+	CCT3	T-complex protein 1 subunit gamma
FUN_003705	contig_4	1760414	1761998	-		hypothetical protein
FUN_003706	contig_4	1764198	1765708	-	STE7	MAP kinase kinase (MEK)
FUN_003707	contig_4	1766825	1771259	+	SPT6	Transcription elongation factor spt6
FUN_003708	contig_4	1771925	1773120	+		hypothetical protein
FUN_003709	contig_4	1774113	1776607	+		hypothetical protein
FUN_003711	contig_4	1782271	1783087	-		hypothetical protein
FUN_003712	contig_4	1784518	1785830	-	ARP1	Centractin
FUN_003713	contig_4	1786212	1788344	-		hypothetical protein
FUN_003714	contig_4	1789503	1791100	+		hypothetical protein
FUN_003717	contig_4	1799289	1801046	-		hypothetical protein
FUN_003718	contig_4	1807700	1808589	-		hypothetical protein
FUN_003719	contig_4	1808856	1812919	+		hypothetical protein
FUN_003720	contig_4	1813673	1814464	-	SNF7	ESCRT-III subunit protein snf7
FUN_003721	contig_4	1814871	1815281	+		hypothetical protein
FUN_003722	contig_4	1816296	1817595	+		hypothetical protein
FUN_003723	contig_4	1817776	1820583	+		hypothetical protein
FUN_003726	contig_4	1826693	1828422	-		hypothetical protein
FUN_003727	contig_4	1829696	1831009	+		hypothetical protein
FUN_003731	contig_4	1839755	1840567	-		hypothetical protein
FUN_003732	contig_4	1841018	1842549	+	WRS1	tryptophan--tRNA ligase
FUN_003733	contig_4	1843490	1845382	+		hypothetical protein
FUN_003734	contig_4	1845721	1846635	-		hypothetical protein
FUN_003735	contig_4	1847612	1850407	+	SPC98	Microtubule-nucleating Tub4p (gamma-tubulin) complex component
FUN_003736	contig_4	1851058	1852629	-		hypothetical protein
FUN_003737	contig_4	1853649	1856821	-		hypothetical protein
FUN_003738	contig_4	1857731	1858204	-	stp1	Low molecular weight phosphotyrosine protein phosphatase
FUN_003740	contig_4	1860080	1861893	+		hypothetical protein
FUN_003741	contig_4	1863002	1865820	+	SNF1	Protein kinase
FUN_003742	contig_4	1866156	1867170	-	ABZ2	Aminodeoxychorismate lyase
FUN_003743	contig_4	1867968	1870048	+	TOM70	TOM (translocase of outer membrane) complex component
FUN_003745	contig_4	1873061	1874778	-		hypothetical protein
FUN_003746	contig_4	1876006	1877122	+		hypothetical protein
FUN_003747	contig_4	1879650	1880952	-		hypothetical protein
FUN_003748	contig_4	1883573	1884336	+		hypothetical protein
FUN_003750	contig_4	1891320	1899244	-		hypothetical protein
FUN_003751	contig_4	1900476	1902379	-		hypothetical protein
FUN_003752	contig_4	1906472	1907989	+		hypothetical protein
FUN_003754	contig_4	1915980	1917728	-	SKO1	Transcription factor
FUN_003757	contig_4	1925817	1927676	+		hypothetical protein
FUN_003759	contig_4	1929760	1931829	+		hypothetical protein
FUN_003762	contig_4	1935174	1937680	-	HAS1	ATP-dependent RNA helicase
FUN_003763	contig_4	1938326	1939496	+		hypothetical protein
FUN_003765	contig_4	1948481	1949965	+		hypothetical protein
FUN_003766	contig_4	1950530	1975285	-		hypothetical protein
FUN_003767	contig_4	1979586	1984699	+		hypothetical protein
FUN_003769	contig_4	1994286	1997021	-		hypothetical protein
FUN_003770	contig_4	1997527	1999807	+	NAS6_2	putative ankyrin-repeat protein
FUN_003771	contig_4	2003779	2004360	-	TIM17	translocase of the inner membrane
FUN_003772	contig_4	2005562	2008759	+		hypothetical protein
FUN_003773	contig_4	2009312	2010442	+		hypothetical protein
FUN_003774	contig_4	2011272	2012480	+		hypothetical protein

Gene ID	Scaffold	Start	Stop	Strand	Name	Product
FUN_003775	contig_4	2013897	2017928	+		hypothetical protein
FUN_003776	contig_4	2018954	2023673	-		hypothetical protein
FUN_003777	contig_4	2025990	2028300	-	FLC3	Putative flavin carrier protein 3
FUN_003779	contig_4	2032750	2038557	+		hypothetical protein
FUN_003782	contig_4	2044733	2045647	-		hypothetical protein
FUN_003783	contig_4	2046704	2048064	+		hypothetical protein
FUN_003788	contig_4	2074164	2074754	-		hypothetical protein
FUN_003790	contig_4	2078271	2080623	-		hypothetical protein
FUN_003793	contig_4	2091979	2100105	-		hypothetical protein
FUN_003794	contig_4	2101855	2102712	-		hypothetical protein
FUN_003795	contig_5	5520	5802	+		hypothetical protein
FUN_003796	contig_5	5872	6714	+		hypothetical protein
FUN_003798	contig_5	10908	12492	-		hypothetical protein
FUN_003799	contig_5	14661	16714	+		hypothetical protein
FUN_003800	contig_5	18073	19601	+		hypothetical protein
FUN_003802	contig_5	24242	25987	-		hypothetical protein
FUN_003803	contig_5	26578	28226	-		hypothetical protein
FUN_003804	contig_5	29115	31248	-		hypothetical protein
FUN_003806	contig_5	35107	35496	-		hypothetical protein
FUN_003807	contig_5	37031	39027	+		hypothetical protein
FUN_003808	contig_5	39331	42675	+		hypothetical protein
FUN_003809	contig_5	44071	44400	-	NTH1_2	alpha,alpha-trehalase nth1
FUN_003813	contig_5	52714	53743	-		hypothetical protein
FUN_003815	contig_5	56181	59251	-		hypothetical protein
FUN_003816	contig_5	59900	61616	-		hypothetical protein
FUN_003817	contig_5	62261	63959	+		hypothetical protein
FUN_003818	contig_5	64512	66485	+		hypothetical protein
FUN_003819	contig_5	68263	69862	+		hypothetical protein
FUN_003821	contig_5	72480	73144	-		hypothetical protein
FUN_003823	contig_5	78310	79027	-		hypothetical protein
FUN_003825	contig_5	84203	85192	+		hypothetical protein
FUN_003830	contig_5	95332	97036	-	SDH1_2	succinate dehydrogenase flavoprotein subunit
FUN_003831	contig_5	98072	99118	-		hypothetical protein
FUN_003832	contig_5	103237	104807	+		hypothetical protein
FUN_003833	contig_5	106938	107545	-		hypothetical protein
FUN_003834	contig_5	108443	110411	+	ATE1	Arginyl-tRNA--protein transferase 1
FUN_003835	contig_5	110715	111735	+	TSC13	Very-long-chain enoyl-CoA reductase
FUN_003836	contig_5	113067	116540	+		hypothetical protein
FUN_003840	contig_5	136857	138985	-		hypothetical protein
FUN_003841	contig_5	146623	148169	-		hypothetical protein
FUN_003842	contig_5	149818	150561	+		hypothetical protein
FUN_003843	contig_5	151170	152381	-		hypothetical protein
FUN_003844	contig_5	154168	155675	-		hypothetical protein
FUN_003845	contig_5	155887	157791	+		hypothetical protein
FUN_003846	contig_5	158090	159628	+	LAG1	sphingosine N-acyltransferase lag1
FUN_003847	contig_5	161750	163540	+	LIP1	sphingosine N-acyltransferase subunit lip1
FUN_003848	contig_5	163851	166062	-	GSH1	glutamate--cysteine ligase
FUN_003849	contig_5	168884	169939	-		hypothetical protein
FUN_003850	contig_5	180170	183321	+	CPH2	Clr6 histone deacetylase associated PHD protein-2 Cph2
FUN_003851	contig_5	183974	184872	+		hypothetical protein
FUN_003853	contig_5	192623	193615	+	BUD32	serine/threonine-protein kinase bud32
FUN_003855	contig_5	198709	200550	+		hypothetical protein
FUN_003856	contig_5	201071	202880	-		hypothetical protein
FUN_003857	contig_5	208565	210467	-		hypothetical protein
FUN_003858	contig_5	211406	212470	+		hypothetical protein
FUN_003859	contig_5	212544	213214	-		hypothetical protein
FUN_003860	contig_5	214108	217311	-		hypothetical protein
FUN_003861	contig_5	218545	220483	+		hypothetical protein
FUN_003862	contig_5	223806	224630	+		hypothetical protein

Gene ID	Scaffold	Start	Stop	Strand	Name	Product
FUN_003863	contig_5	227041	228836	+		hypothetical protein
FUN_003864	contig_5	242749	244406	-		hypothetical protein
FUN_003865	contig_5	248740	250266	-		hypothetical protein
FUN_003867	contig_5	258442	259034	+	OMA1	metalloendopeptidase
FUN_003868	contig_5	264443	268040	-		hypothetical protein
FUN_003870	contig_5	280362	281963	+		hypothetical protein
FUN_003871	contig_5	282838	284407	-	ZRT3	Zinc transporter
FUN_003872	contig_5	285133	287051	-	ATG20	Sorting nexin, cytoplasm-to-vacuole targeting pathway/endosomal sorting
FUN_003874	contig_5	290654	295786	-		hypothetical protein
FUN_003876	contig_5	298562	300725	-		hypothetical protein
FUN_003877	contig_5	303238	305178	-	VPS17	Vacuolar protein sorting-associated protein 17
FUN_003878	contig_5	305928	308365	+	MEF2	Ribosome-releasing factor 2, mitochondrial
FUN_003879	contig_5	308920	310626	-		hypothetical protein
FUN_003880	contig_5	311226	313332	+	PFS2	pre-mRNA cleavage and polyadenylation factor (CPF) complex subunit
FUN_003881	contig_5	315603	316924	-	TIF5	eukaryotic translation initiation factor 5
FUN_003882	contig_5	318127	319842	+	THI6	thiamine biosynthetic bifunctional enzyme
FUN_003883	contig_5	320461	322716	-		hypothetical protein
FUN_003884	contig_5	324389	325910	+		hypothetical protein
FUN_003885	contig_5	326279	327582	-		hypothetical protein
FUN_003887	contig_5	335904	338761	+	PSY2	Platinum sensitivity protein
FUN_003888	contig_5	340517	341303	+	CEL12A	glycoside hydrolase 12
FUN_003889	contig_5	341960	342703	+		hypothetical protein
FUN_003890	contig_5	343528	344088	+		hypothetical protein
FUN_003892	contig_5	348220	352240	-		hypothetical protein
FUN_003893	contig_5	353601	354458	+		hypothetical protein
FUN_003894	contig_5	355070	356341	-		hypothetical protein
FUN_003895	contig_5	357027	359568	+		hypothetical protein
FUN_003896	contig_5	359998	361169	+		hypothetical protein
FUN_003897	contig_5	363231	364343	+		hypothetical protein
FUN_003899	contig_5	370114	371501	+		hypothetical protein
FUN_003900	contig_5	372912	374300	-		hypothetical protein
FUN_003901	contig_5	375143	376900	+		hypothetical protein
FUN_003902	contig_5	378048	379382	+		hypothetical protein
FUN_003903	contig_5	381001	383237	+		hypothetical protein
FUN_003905	contig_5	388720	390182	-		hypothetical protein
FUN_003906	contig_5	390760	392172	+		hypothetical protein
FUN_003907	contig_5	392936	393937	+		hypothetical protein
FUN_003908	contig_5	394019	395510	-	ATF1_1	Alcohol acetyltransferase
FUN_003909	contig_5	397428	398838	+		hypothetical protein
FUN_003911	contig_5	404994	407135	-	set9	histone lysine methyltransferase Set9
FUN_003912	contig_5	407685	408192	+	mrpl38	54S ribosomal protein L38, mitochondrial
FUN_003913	contig_5	408782	409280	-		hypothetical protein
FUN_003914	contig_5	409821	410574	+	pin1	peptidyl-prolyl cis-trans isomerase Pin1
FUN_003916	contig_5	416041	420691	-	BUD4	Bud site selection protein bud4
FUN_003917	contig_5	421701	423371	+	MRH4	RNA helicase
FUN_003918	contig_5	425561	428002	+	QOR1	quinone oxidoreductase
FUN_003919	contig_5	429199	430576	+	xyl1	D-xylose reductase
FUN_003921	contig_5	442130	443007	+		hypothetical protein
FUN_003924	contig_5	450978	452789	-		hypothetical protein
FUN_003926	contig_5	456268	463604	-		hypothetical protein
FUN_003927	contig_5	465419	467500	-	srp72	Signal recognition particle subunit SRP72
FUN_003930	contig_5	473580	475090	-	PDA1	alpha subunit of pyruvate dehydrogenase
FUN_003931	contig_5	475610	477431	-		hypothetical protein
FUN_003932	contig_5	478603	479452	-	PIC2_2	Cu/Pi carrier
FUN_003934	contig_5	482962	485201	-		hypothetical protein
FUN_003935	contig_5	486341	487099	-		hypothetical protein
FUN_003936	contig_5	488844	490501	+	cys2	Serine O-succinyltransferase

Gene ID	Scaffold	Start	Stop	Strand	Name	Product
FUN_003937	contig_5	491061	492823	+		hypothetical protein
FUN_003938	contig_5	493759	495881	-	KU70	ATP-dependent DNA helicase II subunit 1
FUN_003939	contig_5	496191	497517	+	HEM15	ferrochelatase hem15
FUN_003941	contig_5	500871	502177	-		hypothetical protein
FUN_003942	contig_5	502557	504509	+	PUS1	tRNA pseudouridine synthase 1
FUN_003943	contig_5	508112	510049	-		hypothetical protein
FUN_003944	contig_5	510531	511372	-	OYE32_3	NADH-dependent flavin oxidoreductase
FUN_003945	contig_5	512819	514513	+		hypothetical protein
FUN_003946	contig_5	515358	517243	-		hypothetical protein
FUN_003947	contig_5	524007	526421	+		hypothetical protein
FUN_003948	contig_5	527504	528079	+		hypothetical protein
FUN_003949	contig_5	528325	529596	+	ALG5	dolichyl-phosphate beta-glucosyltransferase
FUN_003953	contig_5	537553	541294	-		hypothetical protein
FUN_003954	contig_5	543145	544999	+		hypothetical protein
FUN_003955	contig_5	545726	546883	+		hypothetical protein
FUN_003956	contig_5	547957	548817	+		hypothetical protein
FUN_003957	contig_5	550770	552856	+		hypothetical protein
FUN_003958	contig_5	553565	554639	-		hypothetical protein
FUN_003959	contig_5	555201	556901	+		hypothetical protein
FUN_003960	contig_5	557368	558912	+		hypothetical protein
FUN_003961	contig_5	559852	561311	-		hypothetical protein
FUN_003962	contig_5	568154	571078	-	LIG4	DNA ligase (ATP)
FUN_003963	contig_5	571414	573546	+	IRS4	Increased rDNA silencing protein
FUN_003964	contig_5	573762	574796	-		hypothetical protein
FUN_003965	contig_5	575276	575974	+		hypothetical protein
FUN_003969	contig_5	594642	596305	-		hypothetical protein
FUN_003970	contig_5	596568	598057	-	tit1	tRNA dimethylallyltransferase, mitochondrial
FUN_003971	contig_5	598280	599188	+	CAF16	CCR4-NOT regulatory complex component
FUN_003972	contig_5	600868	601386	-		hypothetical protein
FUN_003973	contig_5	603405	604499	-		hypothetical protein
FUN_003975	contig_5	606004	606626	-	tom22	mitochondrial import receptor subunit Tom22
FUN_003976	contig_5	607294	608458	-	TOM40	translocase of outer mitochondrial membrane
FUN_003977	contig_5	609124	610877	+		hypothetical protein
FUN_003978	contig_5	613768	614672	+		hypothetical protein
FUN_003980	contig_5	618060	619223	-		hypothetical protein
FUN_003981	contig_5	619611	621072	+		hypothetical protein
FUN_003982	contig_5	623107	624264	+		hypothetical protein
FUN_003984	contig_5	626213	634824	-		hypothetical protein
FUN_003985	contig_5	638415	640267	+		hypothetical protein
FUN_003986	contig_5	642534	644897	-		hypothetical protein
FUN_003988	contig_5	648098	648954	+		hypothetical protein
FUN_003990	contig_5	650559	652052	+		hypothetical protein
FUN_003992	contig_5	653760	654641	+		hypothetical protein
FUN_003993	contig_5	655205	656304	+		hypothetical protein
FUN_003994	contig_5	656590	658624	-		hypothetical protein
FUN_003996	contig_5	660553	662429	+		hypothetical protein
FUN_003998	contig_5	664096	665169	+		hypothetical protein
FUN_004002	contig_5	673114	674647	-		hypothetical protein
FUN_004003	contig_5	676344	677426	-		hypothetical protein
FUN_004004	contig_5	680092	681708	+		hypothetical protein
FUN_004005	contig_5	681748	682885	-		hypothetical protein
FUN_004006	contig_5	683378	684844	+		hypothetical protein
FUN_004007	contig_5	685041	686465	-		hypothetical protein
FUN_004008	contig_5	691752	693487	-		hypothetical protein
FUN_004009	contig_5	693951	694721	-		hypothetical protein
FUN_004010	contig_5	694915	697482	-		hypothetical protein
FUN_004011	contig_5	697832	699707	+		hypothetical protein
FUN_004012	contig_5	700147	701713	+		hypothetical protein
FUN_004013	contig_5	703927	705779	+		hypothetical protein

Gene ID	Scaffold	Start	Stop	Strand	Name	Product
FUN_004014	contig_5	706869	707426	+		hypothetical protein
FUN_004015	contig_5	707641	708973	-		hypothetical protein
FUN_004016	contig_5	710921	712432	-		hypothetical protein
FUN_004017	contig_5	713446	715090	+		hypothetical protein
FUN_004018	contig_5	716570	718301	-		hypothetical protein
FUN_004019	contig_5	720099	721793	-		hypothetical protein
FUN_004020	contig_5	722381	723994	-		hypothetical protein
FUN_004021	contig_5	725361	726413	+		hypothetical protein
FUN_004022	contig_5	726684	728111	+		hypothetical protein
FUN_004025	contig_5	734736	736515	-		hypothetical protein
FUN_004026	contig_5	737316	742434	-		hypothetical protein
FUN_004027	contig_5	743474	745738	+	CDR1_2	Multidrug resistance protein
FUN_004028	contig_5	746612	748677	+	CDR1_3	Multidrug resistance protein
FUN_004029	contig_5	753521	755137	+		hypothetical protein
FUN_004030	contig_5	756980	757996	-		hypothetical protein
FUN_004032	contig_5	761909	762613	-		hypothetical protein
FUN_004033	contig_5	763491	764779	-	SNF4	AMP-activated serine/threonine-protein kinase regulatory subunit
FUN_004034	contig_5	765150	766893	-	MgPP2CL-1	mgpp2cl-1, protein phosphatase 2C-like protein 1
FUN_004035	contig_5	768447	769699	-	DID4	ESCRT-III subunit protein did4
FUN_004036	contig_5	769799	772562	+	FZO1_1	mitofusin
FUN_004037	contig_5	772910	775392	+	FZO1_2	mitofusin
FUN_004038	contig_5	775878	777383	+		hypothetical protein
FUN_004039	contig_5	777901	779542	-		hypothetical protein
FUN_004040	contig_5	780482	781064	-	cyn1	Cyanate hydratase
FUN_004041	contig_5	781821	785192	+		hypothetical protein
FUN_004042	contig_5	785532	785915	+		hypothetical protein
FUN_004043	contig_5	787776	789766	-	CDC14	cell division control protein 14
FUN_004044	contig_5	790601	792627	+		hypothetical protein
FUN_004045	contig_5	794324	797125	+	CAT8	DNA-binding transcription factor cat8
FUN_004046	contig_5	799801	800850	-	KTI12	kti12, chromatin associated
FUN_004048	contig_5	803866	805095	-		hypothetical protein
FUN_004049	contig_5	807381	808504	-		hypothetical protein
FUN_004053	contig_5	813799	815739	+	dak1	dihydroxyacetone kinase Dak1
FUN_004054	contig_5	815932	816469	-	SMD2	mRNA splicing protein
FUN_004055	contig_5	817242	821163	+		hypothetical protein
FUN_004057	contig_5	823838	825507	+	PUB1	E3 ubiquitin-protein ligase pub1
FUN_004058	contig_5	829961	831091	-		hypothetical protein
FUN_004059	contig_5	832227	833033	+		hypothetical protein
FUN_004060	contig_5	833482	834542	-		hypothetical protein
FUN_004061	contig_5	835345	837798	-	EDC3	enhancer of mRNA decapping
FUN_004062	contig_5	838340	840752	-		hypothetical protein
FUN_004063	contig_5	841823	843800	-		hypothetical protein
FUN_004064	contig_5	846592	848768	+	CLB4	B-type cyclin
FUN_004065	contig_5	850327	852342	+		hypothetical protein
FUN_004067	contig_5	854270	855100	+	tfg3	transcription factor TFIIF complex subunit Tfg3
FUN_004068	contig_5	855903	857903	+		hypothetical protein
FUN_004069	contig_5	858327	859135	+		hypothetical protein
FUN_004073	contig_5	865435	866602	+	MNN9	Golgi mannosyltransferase complex subunit
FUN_004074	contig_5	867057	867913	-		hypothetical protein
FUN_004075	contig_5	868737	870320	-	DBP5	RNA helicase required for poly(A+) mRNA export
FUN_004076	contig_5	870934	873388	+	bzz1_1	Protein BZZ1
FUN_004077	contig_5	874011	875480	+		hypothetical protein
FUN_004078	contig_5	876910	878334	+	bzz1_2	Protein BZZ1
FUN_004079	contig_5	879891	880520	-		hypothetical protein
FUN_004080	contig_5	882921	883968	+	EFM7	Protein N-terminal and lysine N-methyltransferase efm7
FUN_004083	contig_5	887866	889286	+		hypothetical protein
FUN_004084	contig_5	889492	890409	-	sbp1	Ran GTPase binding protein Sbp1
FUN_004085	contig_5	890936	893350	-	omh4_1	O-glycoside alpha-1,2-mannosyltransferase 4

Gene ID	Scaffold	Start	Stop	Strand	Name	Product
FUN_004086	contig_5	894035	895534	-	omh4_2	O-glycoside alpha-1,2-mannosyltransferase 4
FUN_004087	contig_5	896496	896996	+		hypothetical protein
FUN_004090	contig_5	911900	915316	-		hypothetical protein
FUN_004092	contig_5	922869	924206	+		hypothetical protein
FUN_004093	contig_5	927011	929416	-		hypothetical protein
FUN_004094	contig_5	932641	933427	-		hypothetical protein
FUN_004095	contig_5	933821	935390	-	hob1	BAR adaptor protein Hob1
FUN_004096	contig_5	935928	937702	+	NFS1	cysteine desulfurase
FUN_004097	contig_5	938204	940149	+	STP22	Suppressor protein stp22 of temperature-sensitive alpha-factor receptor and arginine permease
FUN_004098	contig_5	940709	941763	-		hypothetical protein
FUN_004099	contig_5	942568	943816	-		hypothetical protein
FUN_004101	contig_5	945732	946932	+	STE2	pheromone alpha factor receptor
FUN_004102	contig_5	948072	948762	-		hypothetical protein
FUN_004103	contig_5	949783	951441	-	SSN3	cyclin-dependent protein kinase
FUN_004104	contig_5	952032	953649	+	RIB1_1	GTP cyclohydrolase II
FUN_004105	contig_5	954030	955711	+	RIB1_2	GTP cyclohydrolase II
FUN_004106	contig_5	957392	963065	-		hypothetical protein
FUN_004107	contig_5	963491	965855	+	TRP3_1	anthranilate synthase / indole-3-glycerol phosphate synthase
FUN_004108	contig_5	966404	968229	+		hypothetical protein
FUN_004109	contig_5	968744	970659	-	YOX1	Homeobox protein yox1
FUN_004110	contig_5	973416	974209	+		hypothetical protein
FUN_004111	contig_5	975150	976970	+		hypothetical protein
FUN_004112	contig_5	977560	978895	-		hypothetical protein
FUN_004116	contig_5	984572	986968	-		hypothetical protein
FUN_004117	contig_5	988168	989349	-		hypothetical protein
FUN_004118	contig_5	990380	991950	+	TNA1_2	High-affinity nicotinic acid transporter
FUN_004120	contig_5	994249	995632	+		hypothetical protein
FUN_004121	contig_5	995857	997530	+	cyp8	cyclophilin peptidyl-prolyl cis-trans isomerase Cyp8
FUN_004122	contig_5	997961	998656	-		hypothetical protein
FUN_004123	contig_5	1000022	1001318	-		hypothetical protein
FUN_004125	contig_5	1004722	1006462	-	ATG22_1	Autophagy protein 22
FUN_004126	contig_5	1007203	1008532	+	MRPL10	YmL10
FUN_004127	contig_5	1015002	1016916	+		hypothetical protein
FUN_004128	contig_5	1019870	1021414	-		hypothetical protein
FUN_004130	contig_5	1024020	1025237	-		hypothetical protein
FUN_004131	contig_5	1026018	1026358	+		hypothetical protein
FUN_004132	contig_5	1026787	1028614	-	NDE1_2	NADH:ubiquinone oxidoreductase
FUN_004134	contig_5	1030243	1031981	-	ENP1	snoRNA-binding rRNA-processing protein
FUN_004135	contig_5	1032174	1033943	+	ASH2	transcription factor, contains a PHD finger motif
FUN_004136	contig_5	1034397	1036032	+		hypothetical protein
FUN_004137	contig_5	1037710	1041396	+		hypothetical protein
FUN_004138	contig_5	1041470	1043362	-		hypothetical protein
FUN_004141	contig_5	1061476	1063923	-	ELP2	Elongator subunit elp2
FUN_004142	contig_5	1064094	1064525	+	URM1	Ubiquitin-related modifier 1
FUN_004143	contig_5	1065479	1067311	+		hypothetical protein
FUN_004145	contig_5	1070325	1072415	+		hypothetical protein
FUN_004147	contig_5	1073684	1076162	-		hypothetical protein
FUN_004148	contig_5	1077478	1078616	-		hypothetical protein
FUN_004149	contig_5	1079268	1082650	+	POL3	DNA-directed DNA polymerase delta
FUN_004150	contig_5	1084074	1086551	+		hypothetical protein
FUN_004151	contig_5	1086693	1087166	-		hypothetical protein
FUN_004153	contig_5	1087801	1088943	-		hypothetical protein
FUN_004154	contig_5	1089852	1090727	-		hypothetical protein
FUN_004155	contig_5	1091710	1092844	-		hypothetical protein
FUN_004157	contig_5	1097177	1098505	+		hypothetical protein
FUN_004158	contig_5	1099508	1108268	-	FMP27	Protein SABRE
FUN_004159	contig_5	1111408	1113395	+		hypothetical protein

Gene ID	Scaffold	Start	Stop	Strand	Name	Product
FUN_004160	contig_5	1113443	1114906	-		hypothetical protein
FUN_004161	contig_5	1116441	1118063	-		hypothetical protein
FUN_004162	contig_5	1118578	1119462	-		hypothetical protein
FUN_004163	contig_5	1122636	1124291	-		hypothetical protein
FUN_004164	contig_5	1125227	1126865	-		hypothetical protein
FUN_004165	contig_5	1127595	1128461	+		hypothetical protein
FUN_004166	contig_5	1128564	1129921	-	YEY2_2	Valine--pyruvate aminotransferase
FUN_004167	contig_5	1130228	1134348	+		hypothetical protein
FUN_004168	contig_5	1135311	1137146	+		hypothetical protein
FUN_004171	contig_5	1140585	1141706	+		hypothetical protein
FUN_004173	contig_5	1153625	1154809	+		hypothetical protein
FUN_004174	contig_5	1155037	1155375	+		hypothetical protein
FUN_004175	contig_5	1156452	1158814	+		hypothetical protein
FUN_004177	contig_5	1167029	1167828	-		hypothetical protein
FUN_004178	contig_5	1168263	1170143	-		hypothetical protein
FUN_004179	contig_5	1170557	1171683	-		hypothetical protein
FUN_004182	contig_5	1177025	1177746	+		hypothetical protein
FUN_004184	contig_5	1180619	1181653	-		hypothetical protein
FUN_004185	contig_5	1182485	1184085	+		hypothetical protein
FUN_004186	contig_5	1186405	1186770	+		hypothetical protein
FUN_004187	contig_5	1188128	1189146	-		hypothetical protein
FUN_004188	contig_5	1190278	1197474	-		hypothetical protein
FUN_004189	contig_5	1200581	1202444	-		hypothetical protein
FUN_004190	contig_5	1203589	1205098	+		hypothetical protein
FUN_004192	contig_5	1210424	1211450	+		hypothetical protein
FUN_004193	contig_5	1211987	1212709	+		hypothetical protein
FUN_004194	contig_5	1213527	1215317	+		hypothetical protein
FUN_004195	contig_5	1215517	1216683	-		hypothetical protein
FUN_004196	contig_5	1220598	1223558	+	PHM7_2	phosphate metabolism protein 7
FUN_004197	contig_5	1226797	1227687	-		hypothetical protein
FUN_004198	contig_5	1228013	1229351	-		hypothetical protein
FUN_004199	contig_5	1229905	1231740	+		hypothetical protein
FUN_004201	contig_5	1233444	1235267	-		hypothetical protein
FUN_004202	contig_5	1235769	1237018	-		hypothetical protein
FUN_004203	contig_5	1240107	1240829	-		hypothetical protein
FUN_004204	contig_5	1241426	1242638	-	TAZ1	Lyso-phosphatidylcholine acyltransferase
FUN_004205	contig_5	1242903	1244450	-		hypothetical protein
FUN_004206	contig_5	1245800	1246567	+	RRM1	Ribonucleoside-diphosphate reductase large subunit
FUN_004207	contig_5	1247212	1248099	+		hypothetical protein
FUN_004208	contig_5	1248462	1249945	-		hypothetical protein
FUN_004209	contig_5	1250388	1251711	+		hypothetical protein
FUN_004210	contig_5	1255991	1257043	-	mtg1	Mitochondrial GTPase 1
FUN_004211	contig_5	1257952	1258982	+		hypothetical protein
FUN_004213	contig_5	1262089	1263593	-	GCS1	ADP-ribosylation factor GTPase-activating protein gcs1
FUN_004214	contig_5	1264370	1265935	-		hypothetical protein
FUN_004215	contig_5	1268414	1269573	-	SPS19	peroxisomal 2 4-dienoyl-CoA reductase sps19
FUN_004217	contig_5	1271451	1274183	-	SFC1	Mitochondrial succinate-fumarate transporter
FUN_004218	contig_5	1275170	1276954	-	ECM14	Putative metallocarboxypeptidase ecm14
FUN_004219	contig_5	1277533	1281950	+		hypothetical protein
FUN_004220	contig_5	1284367	1286018	-		hypothetical protein
FUN_004221	contig_5	1286831	1287703	+		hypothetical protein
FUN_004222	contig_5	1289475	1291970	-		hypothetical protein
FUN_004223	contig_5	1297313	1300122	+	ALG8	glycosyl transferase
FUN_004224	contig_5	1300696	1302221	+	sgf73	SAGA complex subunit Sgf73
FUN_004225	contig_5	1302820	1303920	-		hypothetical protein
FUN_004226	contig_5	1304353	1305740	+		hypothetical protein
FUN_004229	contig_5	1310970	1312697	+		hypothetical protein
FUN_004230	contig_5	1312896	1314116	-		hypothetical protein
FUN_004231	contig_5	1314799	1315784	+		hypothetical protein

Gene ID	Scaffold	Start	Stop	Strand	Name	Product
FUN_004234	contig_5	1320605	1323897	-		hypothetical protein
FUN_004235	contig_5	1324889	1327007	-		hypothetical protein
FUN_004237	contig_5	1331610	1332140	-	cdc42	GTPase Cdc42
FUN_004238	contig_5	1333742	1335128	+		hypothetical protein
FUN_004239	contig_5	1336218	1338579	+		hypothetical protein
FUN_004240	contig_5	1339683	1341190	+	CPA1	Multifunctional pyrimidine synthesis protein CAD
FUN_004243	contig_5	1348566	1352691	+		hypothetical protein
FUN_004244	contig_5	1353292	1355078	-		hypothetical protein
FUN_004245	contig_5	1361419	1362349	+		hypothetical protein
FUN_004246	contig_5	1364820	1365783	+		hypothetical protein
FUN_004247	contig_5	1365913	1367936	-		hypothetical protein
FUN_004249	contig_5	1370923	1372023	+		hypothetical protein
FUN_004251	contig_5	1376001	1376918	-	CWC24	RNA-splicing factor
FUN_004252	contig_5	1378005	1383218	+		hypothetical protein
FUN_004253	contig_5	1384456	1385226	+	ISY1	NineTeen Complex (NTC) component
FUN_004254	contig_5	1385584	1386784	-		hypothetical protein
FUN_004255	contig_5	1387468	1389242	+	CCT8	T-complex protein 1 subunit theta
FUN_004257	contig_5	1391213	1395310	+	DRS2	aminophospholipid translocase
FUN_004258	contig_5	1396210	1397196	+		hypothetical protein
FUN_004259	contig_5	1402170	1403423	+		hypothetical protein
FUN_004260	contig_5	1404438	1406574	+		hypothetical protein
FUN_004263	contig_5	1415567	1417101	-		hypothetical protein
FUN_004264	contig_5	1418183	1419563	-		hypothetical protein
FUN_004265	contig_5	1422568	1423904	-		hypothetical protein
FUN_004266	contig_5	1424933	1425987	-		hypothetical protein
FUN_004267	contig_5	1426312	1426686	+	ini1	Pre-mRNA-splicing factor ini1
FUN_004268	contig_5	1431758	1432918	+	ATG3	E2-like enzyme
FUN_004269	contig_5	1434880	1437096	+		hypothetical protein
FUN_004270	contig_5	1438703	1445281	-		hypothetical protein
FUN_004271	contig_5	1446083	1447000	-	CMD1	Calmodulin
FUN_004272	contig_5	1447504	1451035	+	tlg2	t-SNARE affecting a late Golgi compartment protein 2
FUN_004274	contig_5	1453255	1454760	-		hypothetical protein
FUN_004275	contig_5	1455157	1456134	+	NUC1	nuclease
FUN_004276	contig_5	1457617	1458348	+		hypothetical protein
FUN_004277	contig_5	1460113	1461597	-		hypothetical protein
FUN_004278	contig_5	1463595	1464581	-		hypothetical protein
FUN_004279	contig_5	1465561	1467600	-		hypothetical protein
FUN_004280	contig_5	1467771	1468255	+	NIP7	ribosome biosynthesis protein nip7
FUN_004283	contig_5	1471353	1472993	-	FDC1	Ferulic acid decarboxylase 1
FUN_004284	contig_5	1473409	1474209	+	PAD1	phenylacrylic acid decarboxylase
FUN_004286	contig_5	1475822	1476538	-	CWC15	complexed with cef1p
FUN_004287	contig_5	1476748	1477726	+	RRP45	3'-5'-exoribonuclease
FUN_004288	contig_5	1478533	1479431	+		hypothetical protein
FUN_004289	contig_5	1479994	1482833	+		hypothetical protein
FUN_004290	contig_5	1483237	1484839	+	RMD1	sporulation protein rmd1
FUN_004291	contig_5	1485358	1488557	+	PIK1	Phosphatidylinositol 4-kinase pik1alpha (PI4-kinase)(PtdIns-4-kinase)
FUN_004292	contig_5	1489711	1490964	+		hypothetical protein
FUN_004294	contig_5	1496595	1498241	+		hypothetical protein
FUN_004295	contig_5	1499223	1500659	+		hypothetical protein
FUN_004296	contig_5	1501575	1503120	+		hypothetical protein
FUN_004297	contig_5	1503799	1505142	+		hypothetical protein
FUN_004298	contig_5	1505578	1507512	-		hypothetical protein
FUN_004300	contig_5	1511467	1512601	-	TWF1	Twinfilin-1
FUN_004302	contig_5	1518921	1519911	-		hypothetical protein
FUN_004303	contig_5	1521574	1523802	+	CRN1	Coronin-like protein crn1
FUN_004305	contig_5	1532502	1534297	+		hypothetical protein
FUN_004306	contig_5	1534629	1536177	-		hypothetical protein
FUN_004307	contig_5	1536953	1538384	-		hypothetical protein

Gene ID	Scaffold	Start	Stop	Strand	Name	Product
FUN_004308	contig_5	1539025	1541827	+	VPS34	Phosphatidylinositol (PI) 3-kinase
FUN_004309	contig_5	1542021	1544453	-		hypothetical protein
FUN_004310	contig_5	1544974	1546529	+		hypothetical protein
FUN_004314	contig_5	1553408	1554133	+		hypothetical protein
FUN_004315	contig_5	1555048	1555789	-	RSR1_2	Ras-related protein rsr1
FUN_004316	contig_5	1556431	1557721	+		hypothetical protein
FUN_004318	contig_5	1561022	1561945	+		hypothetical protein
FUN_004319	contig_5	1562053	1565330	-		hypothetical protein
FUN_004320	contig_5	1565803	1568527	-		hypothetical protein
FUN_004322	contig_5	1574219	1575450	-	sds22	protein phosphatase regulatory subunit Sds22
FUN_004323	contig_5	1577891	1579864	+		hypothetical protein
FUN_004324	contig_5	1580875	1582064	+	vps66	Lysophosphatidic acid:oleoyl-CoA acyltransferase 1
FUN_004325	contig_5	1582862	1585893	+	KAR3	kinesin-like nuclear fusion protein
FUN_004326	contig_5	1587401	1589599	-	URA7	CTP synthase ura7
FUN_004327	contig_5	1590386	1591844	-		hypothetical protein
FUN_004328	contig_5	1594540	1595100	+		hypothetical protein
FUN_004329	contig_5	1599400	1600242	+		hypothetical protein
FUN_004331	contig_5	1603302	1604906	-	ERG1	Squalene epoxidase
FUN_004333	contig_5	1609796	1610750	+	YSA1	ADP-ribose diphosphatase
FUN_004334	contig_5	1611513	1613053	+		hypothetical protein
FUN_004336	contig_5	1614362	1615552	-		hypothetical protein
FUN_004337	contig_5	1615789	1618361	+	AVO1	Component of a membrane-bound complex containing the Tor2p kinase
FUN_004338	contig_5	1619003	1623750	-		hypothetical protein
FUN_004339	contig_5	1624583	1626380	-		hypothetical protein
FUN_004340	contig_5	1630083	1632095	+		hypothetical protein
FUN_004341	contig_5	1640059	1641288	-		hypothetical protein
FUN_004343	contig_5	1643842	1644701	+		hypothetical protein
FUN_004345	contig_5	1650059	1651434	+		hypothetical protein
FUN_004346	contig_5	1652196	1653810	+		hypothetical protein
FUN_004347	contig_5	1654434	1655627	-		hypothetical protein
FUN_004348	contig_5	1656963	1659186	+		hypothetical protein
FUN_004350	contig_5	1663674	1664788	-		hypothetical protein
FUN_004353	contig_5	1672759	1673670	+		hypothetical protein
FUN_004354	contig_5	1676545	1677411	+	PTR2_3	peptide transporter ptr2
FUN_004360	contig_5	1686748	1688099	-		hypothetical protein
FUN_004361	contig_5	1688525	1689562	+		hypothetical protein
FUN_004362	contig_5	1689756	1690722	-		hypothetical protein
FUN_004364	contig_5	1692294	1697057	+		hypothetical protein
FUN_004365	contig_5	1697490	1700727	+		hypothetical protein
FUN_004366	contig_5	1701773	1703775	+		hypothetical protein
FUN_004367	contig_5	1704102	1704987	-	CIR1	Putative electron transfer flavoprotein subunit
FUN_004368	contig_5	1705286	1707261	-	TAH18	NAPDH-dependent diflavin reductase
FUN_004369	contig_5	1708092	1709342	+		hypothetical protein
FUN_004370	contig_5	1710759	1711918	+		hypothetical protein
FUN_004371	contig_5	1712848	1713902	-		hypothetical protein
FUN_004372	contig_5	1714219	1715949	+	HAT1	histone acetyltransferase 1
FUN_004373	contig_5	1716328	1717059	+	ade5	Bifunctional purine biosynthetic protein ADE5,7
FUN_004375	contig_5	1719434	1720857	-		hypothetical protein
FUN_004376	contig_5	1721211	1722784	-	CEM1	Mitochondrial beta-keto-acyl synthase
FUN_004377	contig_5	1724618	1726286	+		hypothetical protein
FUN_004379	contig_5	1728634	1730736	-		hypothetical protein
FUN_004380	contig_5	1731164	1732467	-		hypothetical protein
FUN_004381	contig_5	1733509	1735738	-		hypothetical protein
FUN_004382	contig_5	1748588	1751811	-		hypothetical protein
FUN_004383	contig_5	1752248	1756751	+	PDS5	Sister chromatid cohesion protein pds5
FUN_004384	contig_5	1757940	1759130	+	cbp3	Serine carboxypeptidase 3
FUN_004385	contig_5	1759513	1760835	-	CEG1	Dcp1p-Dcp2p decapping enzyme complex alpha subunit
FUN_004387	contig_5	1764546	1768033	-		hypothetical protein

Gene ID	Scaffold	Start	Stop	Strand	Name	Product
FUN_004388	contig_5	1770045	1772075	+		hypothetical protein
FUN_004389	contig_5	1773080	1773616	-		hypothetical protein
FUN_004390	contig_5	1774495	1775301	+		hypothetical protein
FUN_004391	contig_5	1780157	1782627	+		hypothetical protein
FUN_004392	contig_5	1783244	1786392	-		hypothetical protein
FUN_004394	contig_5	1791202	1791968	+	STE14	farnesyl cysteine-carboxyl methyltransferase
FUN_004396	contig_5	1795951	1797343	+		hypothetical protein
FUN_004398	contig_5	1801625	1803205	-		hypothetical protein
FUN_004399	contig_5	1803887	1805689	+	ILV3	dihydroxy-acid dehydratase ilv3
FUN_004401	contig_5	1814702	1816752	+		hypothetical protein
FUN_004402	contig_5	1817123	1818800	-		hypothetical protein
FUN_004403	contig_5	1819944	1820948	+		hypothetical protein
FUN_004404	contig_5	1821159	1822324	-		hypothetical protein
FUN_004405	contig_5	1822824	1823857	+		hypothetical protein
FUN_004406	contig_5	1830516	1831998	-		hypothetical protein
FUN_004407	contig_5	1832229	1832684	-		hypothetical protein
FUN_004410	contig_5	1838964	1839495	+		hypothetical protein
FUN_004412	contig_5	1842678	1844144	+		hypothetical protein
FUN_004417	contig_5	1857777	1858889	-		hypothetical protein
FUN_004418	contig_5	1859588	1860815	-		hypothetical protein
FUN_004419	contig_5	1861306	1865816	-		hypothetical protein
FUN_004420	contig_5	1866289	1867675	+		hypothetical protein
FUN_004422	contig_5	1870840	1871763	+		hypothetical protein
FUN_004424	contig_5	1873707	1875245	+	NSRP1	Nuclear speckle splicing regulatory protein 1
FUN_004425	contig_5	1875669	1878161	+		hypothetical protein
FUN_004426	contig_5	1883491	1885853	-		hypothetical protein
FUN_004427	contig_5	1886614	1887988	-		hypothetical protein
FUN_004428	contig_5	1889340	1889582	+		hypothetical protein
FUN_004430	contig_5	1891092	1892098	-	PRO3	delta 1-pyrroline-5-carboxylate reductase
FUN_004431	contig_5	1892406	1893146	+		hypothetical protein
FUN_004432	contig_5	1893345	1893992	-	MNP1	54S ribosomal protein L12, mitochondrial
FUN_004433	contig_5	1894264	1908965	+	MDN1	AAA ATPase midasin
FUN_004439	contig_5	1927030	1928253	+		hypothetical protein
FUN_004441	contig_5	1958989	1960205	+		hypothetical protein
FUN_004443	contig_5	1965836	1966857	+		hypothetical protein
FUN_004444	contig_5	1968558	1970483	-		hypothetical protein
FUN_004445	contig_5	1971882	1973378	-	TPK2_1	cAMP-dependent protein kinase catalytic subunit
FUN_004446	contig_5	1973923	1975168	-		hypothetical protein
FUN_004447	contig_5	1975479	1977010	+		hypothetical protein
FUN_004448	contig_5	1977887	1979722	+		hypothetical protein
FUN_004450	contig_5	1984049	1985415	+	GEF2_1	chloride channel
FUN_004452	contig_5	1986873	1989789	+	GEF2_2	chloride channel
FUN_004453	contig_5	1993781	1994919	+		hypothetical protein
FUN_004454	contig_5	1997732	2001870	+		hypothetical protein
FUN_004456	contig_5	2004405	2007006	+		hypothetical protein
FUN_004457	contig_5	2009636	2010754	-		hypothetical protein
FUN_004458	contig_5	2012060	2012993	+		hypothetical protein
FUN_004459	contig_5	2013707	2015203	-		hypothetical protein
FUN_004460	contig_5	2016425	2017777	+		hypothetical protein
FUN_004462	contig_5	2020759	2021700	+		hypothetical protein
FUN_004463	contig_6	2244	3530	+		hypothetical protein
FUN_004465	contig_6	8829	9904	+		hypothetical protein
FUN_004466	contig_6	15985	18459	+		hypothetical protein
FUN_004467	contig_6	26444	27644	+		hypothetical protein
FUN_004471	contig_6	56735	60176	+	ENA2_1	P-type ATPase
FUN_004472	contig_6	63492	64026	+		hypothetical protein
FUN_004473	contig_6	65340	67925	+		hypothetical protein
FUN_004475	contig_6	79515	80429	-		hypothetical protein
FUN_004476	contig_6	80856	82102	+		hypothetical protein

Gene ID	Scaffold	Start	Stop	Strand	Name	Product
FUN_004477	contig_6	82255	82696	-		hypothetical protein
FUN_004478	contig_6	82974	86089	+	CDC54	MCM DNA helicase complex subunit
FUN_004479	contig_6	97578	98549	+		hypothetical protein
FUN_004481	contig_6	101360	103030	+	LYP1	lysine permease
FUN_004483	contig_6	109652	110650	-		hypothetical protein
FUN_004485	contig_6	123494	125084	-		hypothetical protein
FUN_004486	contig_6	152486	154372	-		hypothetical protein
FUN_004487	contig_6	161304	165120	-		hypothetical protein
FUN_004488	contig_6	166507	173765	-		hypothetical protein
FUN_004489	contig_6	174117	176507	-	DPB2	DNA-directed DNA polymerase epsilon, subunit B
FUN_004490	contig_6	176780	178171	+		hypothetical protein
FUN_004491	contig_6	179025	180075	+		hypothetical protein
FUN_004493	contig_6	183280	184285	-		hypothetical protein
FUN_004494	contig_6	184863	185878	-		hypothetical protein
FUN_004495	contig_6	186165	187405	+		hypothetical protein
FUN_004496	contig_6	187650	190106	+		hypothetical protein
FUN_004500	contig_6	202313	204223	-		hypothetical protein
FUN_004501	contig_6	205323	205869	-		hypothetical protein
FUN_004503	contig_6	208711	210437	+		hypothetical protein
FUN_004504	contig_6	211095	212098	-		hypothetical protein
FUN_004505	contig_6	212554	214347	+		hypothetical protein
FUN_004506	contig_6	214860	216717	+	HEM14	oxygen-dependent protoporphyrinogen oxidase
FUN_004508	contig_6	225626	227085	-		hypothetical protein
FUN_004509	contig_6	227863	228937	-		hypothetical protein
FUN_004511	contig_6	231540	233532	-		hypothetical protein
FUN_004512	contig_6	236494	238443	+		hypothetical protein
FUN_004513	contig_6	239920	241223	+		hypothetical protein
FUN_004515	contig_6	245002	245933	+		hypothetical protein
FUN_004516	contig_6	246008	247170	-		hypothetical protein
FUN_004517	contig_6	247543	248129	-	RPL44	40s ribosomal protein L44e
FUN_004518	contig_6	249062	249358	+		hypothetical protein
FUN_004519	contig_6	249613	251760	-	SRC1	inner nuclear membrane protein enriched at telomere/subtelomere region
FUN_004526	contig_6	284592	285047	-		hypothetical protein
FUN_004527	contig_6	285350	288626	+		hypothetical protein
FUN_004528	contig_6	289182	296202	-		hypothetical protein
FUN_004529	contig_6	296861	298182	+	RPN6	26S proteasome regulatory subunit rpn6
FUN_004530	contig_6	300380	301467	+	GRP2_1	Glycine-rich RNA-binding protein 2, mitochondrial
FUN_004531	contig_6	305106	306677	+		hypothetical protein
FUN_004532	contig_6	306772	312142	-		hypothetical protein
FUN_004533	contig_6	312432	314031	+	prp3	U4/U5/U6 small nuclear ribonucleoprotein prp3
FUN_004536	contig_6	328940	330527	-		hypothetical protein
FUN_004537	contig_6	331313	333182	-	NAG1_1	Glucosamine-6-phosphate isomerase (Glucosamine-6-phosphate deaminase) (GNPDA) (GlcN6P deaminase)
FUN_004538	contig_6	333975	335510	-	CRH1_1	transglycosylase
FUN_004539	contig_6	336385	337932	+	PRO2	glutamate-5-semialdehyde dehydrogenase
FUN_004540	contig_6	338662	340447	-		hypothetical protein
FUN_004541	contig_6	341581	343002	+		hypothetical protein
FUN_004542	contig_6	343644	344485	+		hypothetical protein
FUN_004543	contig_6	345240	346934	+		hypothetical protein
FUN_004544	contig_6	348234	349297	+		hypothetical protein
FUN_004545	contig_6	350324	352503	+	ANKRD52	Ankyrin repeat domain-containing protein 52
FUN_004546	contig_6	353120	354235	-		hypothetical protein
FUN_004550	contig_6	361934	363142	+		hypothetical protein
FUN_004552	contig_6	366415	368377	-		hypothetical protein
FUN_004553	contig_6	368905	370248	-	HYM1_1	Hym1p
FUN_004554	contig_6	370788	372445	-	HYM1_2	Hym1p
FUN_004555	contig_6	372821	375741	+	SYP1	Suppressor of profilin deletion
FUN_004556	contig_6	377440	383319	+		hypothetical protein

Gene ID	Scaffold	Start	Stop	Strand	Name	Product
FUN_004557	contig_6	384220	385788	+		hypothetical protein
FUN_004559	contig_6	389516	391399	-		hypothetical protein
FUN_004560	contig_6	391757	396544	+		hypothetical protein
FUN_004562	contig_6	402028	404901	-		hypothetical protein
FUN_004563	contig_6	405120	407139	+	UTP18	U3 snoRNP protein
FUN_004564	contig_6	407445	408868	-		hypothetical protein
FUN_004566	contig_6	410713	412840	-	fim1	fimbrin
FUN_004567	contig_6	414933	416999	-		hypothetical protein
FUN_004568	contig_6	426986	432666	-		hypothetical protein
FUN_004569	contig_6	434480	436750	-	ATG7	Autophagy protein 7
FUN_004571	contig_6	441318	442859	-	HST2	Sir2 histone deacetylase Hst2
FUN_004572	contig_6	444325	445391	+		hypothetical protein
FUN_004573	contig_6	446311	447437	+		hypothetical protein
FUN_004574	contig_6	447808	449523	+	MPE1	Protein mpe1
FUN_004575	contig_6	451098	455202	+		hypothetical protein
FUN_004576	contig_6	455723	458561	-		hypothetical protein
FUN_004577	contig_6	458955	461017	+	GYP1	GTPase-activating protein
FUN_004578	contig_6	461425	462494	+		hypothetical protein
FUN_004579	contig_6	469090	472362	-	SWE1	mitosis inhibitor protein kinase swe1
FUN_004580	contig_6	474705	476005	-		hypothetical protein
FUN_004583	contig_6	483979	485368	-		hypothetical protein
FUN_004585	contig_6	488618	490131	+	hxk1	hexokinase
FUN_004586	contig_6	496115	497552	-	SCJ1	DnaJ-related protein scj1
FUN_004587	contig_6	498009	498794	+		hypothetical protein
FUN_004588	contig_6	499183	500539	-	CDC7	Cell division control protein 7
FUN_004589	contig_6	500860	504771	+	cnd1	condensin complex non-SMC subunit Cnd1
FUN_004590	contig_6	508705	510079	-		hypothetical protein
FUN_004591	contig_6	512965	514102	+		hypothetical protein
FUN_004592	contig_6	517049	517692	-		hypothetical protein
FUN_004593	contig_6	517951	518334	+	LRO1_1	phospholipid:diacylglycerol acyltransferase
FUN_004594	contig_6	518464	519995	+	LRO1_2	phospholipid:diacylglycerol acyltransferase
FUN_004596	contig_6	524315	527811	-		hypothetical protein
FUN_004597	contig_6	528111	528952	-	RET3	Golgi-to-ER vesicle coat component
FUN_004599	contig_6	537910	538227	+		hypothetical protein
FUN_004600	contig_6	538683	541240	+	IST1	Vacuolar protein sorting-associated protein ist1
FUN_004601	contig_6	544883	548059	+	VAC7	Vacuolar inheritance and morphology protein
FUN_004602	contig_6	554394	555373	+		hypothetical protein
FUN_004603	contig_6	556051	557281	-		hypothetical protein
FUN_004606	contig_6	565871	567597	+	INV1	Invertase
FUN_004608	contig_6	569556	572606	+		hypothetical protein
FUN_004609	contig_6	577295	578906	-	CAT1	catalase A
FUN_004610	contig_6	579600	581405	-		hypothetical protein
FUN_004612	contig_6	587453	588808	+		hypothetical protein
FUN_004615	contig_6	593645	594485	+		hypothetical protein
FUN_004616	contig_6	595161	596315	+		hypothetical protein
FUN_004617	contig_6	597157	597805	+		hypothetical protein
FUN_004619	contig_6	601166	603589	-		hypothetical protein
FUN_004620	contig_6	605987	607623	-		hypothetical protein
FUN_004621	contig_6	608140	610546	-	COG4	Golgi transport complex subunit 4
FUN_004622	contig_6	611292	613346	+		hypothetical protein
FUN_004623	contig_6	615192	616303	-		hypothetical protein
FUN_004624	contig_6	616746	617486	-	sof1	Protein sof1
FUN_004625	contig_6	618382	619068	+	RPL27B	60S ribosomal protein L27B
FUN_004626	contig_6	620391	621066	-		hypothetical protein
FUN_004627	contig_6	623288	626153	-	msh2	MSH2 protein
FUN_004628	contig_6	627253	628627	+		hypothetical protein
FUN_004629	contig_6	629770	630687	-		hypothetical protein
FUN_004630	contig_6	631199	632276	-		hypothetical protein
FUN_004632	contig_6	634803	637695	+	prp1	U4/U6 x U5 tri-snRNP complex subunit Prp1

Gene ID	Scaffold	Start	Stop	Strand	Name	Product
FUN_004633	contig_6	638394	639751	+		hypothetical protein
FUN_004634	contig_6	640269	641819	-		hypothetical protein
FUN_004635	contig_6	644444	646279	+		hypothetical protein
FUN_004636	contig_6	646838	648045	+		hypothetical protein
FUN_004637	contig_6	648219	649095	-		hypothetical protein
FUN_004638	contig_6	651030	652114	+		hypothetical protein
FUN_004640	contig_6	655866	656517	-	ISU1	iron-binding protein
FUN_004641	contig_6	656929	659672	-	ubp14	ubiquitin C-terminal hydrolase Ubp14
FUN_004642	contig_6	659983	663178	-	POL5	DNA-directed DNA polymerase
FUN_004643	contig_6	665987	667869	+	MIH1	m-phase inducer phosphatase
FUN_004644	contig_6	669032	669832	+	SLD5	GINS complex subunit
FUN_004645	contig_6	670066	671300	-		hypothetical protein
FUN_004646	contig_6	671538	673634	+	RFA1	Replication factor A protein 1
FUN_004647	contig_6	674141	675241	+		hypothetical protein
FUN_004649	contig_6	678726	681509	+	ACF2_1	endo-1,3-beta glucanase
FUN_004650	contig_6	683972	685435	-		hypothetical protein
FUN_004651	contig_6	687870	689546	+	AUR1	Phosphatidylinositol:ceramide phosphoinositol transferase (IPC synthase)
FUN_004653	contig_6	693105	693681	+		hypothetical protein
FUN_004654	contig_6	694460	696198	-	GBP2	g-strand binding protein
FUN_004655	contig_6	698893	700621	+	NAG4	Synaptic vesicle transporter SVOP
FUN_004656	contig_6	701910	704387	+		hypothetical protein
FUN_004657	contig_6	704877	706182	-		hypothetical protein
FUN_004659	contig_6	732977	734050	-		hypothetical protein
FUN_004660	contig_6	734675	737050	-		hypothetical protein
FUN_004661	contig_6	738428	739223	+		hypothetical protein
FUN_004663	contig_6	744040	747866	-		hypothetical protein
FUN_004664	contig_6	751194	752898	-		hypothetical protein
FUN_004665	contig_6	756379	759240	+		hypothetical protein
FUN_004666	contig_6	760549	762662	-		hypothetical protein
FUN_004667	contig_6	763366	764686	-	URA4	dihydroorotase
FUN_004668	contig_6	765048	768648	-		hypothetical protein
FUN_004669	contig_6	769577	770850	-		hypothetical protein
FUN_004671	contig_6	773574	775054	-		hypothetical protein
FUN_004672	contig_6	775580	776718	-		hypothetical protein
FUN_004673	contig_6	777532	779131	+		hypothetical protein
FUN_004674	contig_6	784377	785100	-		hypothetical protein
FUN_004675	contig_6	786094	787184	-	OST3	oligosaccharyl transferase subunit ost3/OST6
FUN_004677	contig_6	792144	793339	+		hypothetical protein
FUN_004678	contig_6	794402	795535	-		hypothetical protein
FUN_004679	contig_6	797341	798311	+	MRM2	2' O-ribose methyltransferase
FUN_004681	contig_6	801536	804173	-		hypothetical protein
FUN_004682	contig_6	809285	810614	+		hypothetical protein
FUN_004683	contig_6	811957	814523	+	APL2	beta-adaptin
FUN_004684	contig_6	814913	817430	+	vid27	Vacuolar import and degradation protein 27
FUN_004685	contig_6	820176	820371	+	TPO5_1	polyamine transporter tpo5
FUN_004687	contig_6	825860	829236	+		hypothetical protein
FUN_004688	contig_6	831593	832797	+		hypothetical protein
FUN_004690	contig_6	835853	837694	+		hypothetical protein
FUN_004691	contig_6	839226	841026	-	TPS1	Trehalose-6-P synthase/phosphatase complex synthase subunit
FUN_004692	contig_6	843812	845150	+		hypothetical protein
FUN_004693	contig_6	846446	848424	-		hypothetical protein
FUN_004695	contig_6	853465	855211	+	RTG2	retrograde regulation protein 2
FUN_004696	contig_6	856636	859960	+	GDH2	NAD-dependent glutamate dehydrogenase
FUN_004698	contig_6	862289	863238	-		hypothetical protein
FUN_004699	contig_6	863723	865090	+	SAM2	methionine adenosyltransferase sam2
FUN_004701	contig_6	868295	870635	+		hypothetical protein
FUN_004702	contig_6	870754	872343	-	RFT1	Oligosaccharide translocation protein rft1

Gene ID	Scaffold	Start	Stop	Strand	Name	Product
FUN_004703	contig_6	874482	876769	+		hypothetical protein
FUN_004704	contig_6	877580	885764	-	GCN1	translational activator of GCN4
FUN_004705	contig_6	886116	890604	+		hypothetical protein
FUN_004707	contig_6	892835	893503	-		hypothetical protein
FUN_004708	contig_6	893962	894923	-		hypothetical protein
FUN_004709	contig_6	895757	896174	-		hypothetical protein
FUN_004710	contig_6	897449	899644	+	SKN7	kinase-regulated stress-responsive transcription factor skn7
FUN_004711	contig_6	900483	902813	+	GCD6	translation initiation factor eIF-2B epsilon subunit, GEF
FUN_004712	contig_6	903455	905286	+		hypothetical protein
FUN_004713	contig_6	906001	906289	-		hypothetical protein
FUN_004714	contig_6	909968	911474	+		hypothetical protein
FUN_004715	contig_6	912082	912940	-		hypothetical protein
FUN_004716	contig_6	913668	915500	-	GND1	phosphogluconate dehydrogenase (decarboxylating) gnd1
FUN_004717	contig_6	916462	917466	+		hypothetical protein
FUN_004718	contig_6	918360	920590	+		hypothetical protein
FUN_004719	contig_6	923191	924222	+		hypothetical protein
FUN_004722	contig_6	929407	930664	+		hypothetical protein
FUN_004724	contig_6	936458	937350	-		hypothetical protein
FUN_004726	contig_6	940535	944398	+		hypothetical protein
FUN_004728	contig_6	946993	948329	-	TYS1	Tyrosine--tRNA ligase cytoplasmic
FUN_004729	contig_6	948793	950803	-		hypothetical protein
FUN_004730	contig_6	954073	957726	+		hypothetical protein
FUN_004731	contig_6	962838	967471	-	KRE5	killer toxin resistant protein
FUN_004732	contig_6	968157	969700	-	OST1	dolichyl-diphosphooligosaccharide--protein glycosyltransferase subunit 1
FUN_004733	contig_6	969915	971304	+		hypothetical protein
FUN_004734	contig_6	971395	972633	+		hypothetical protein
FUN_004736	contig_6	977383	977948	-		hypothetical protein
FUN_004737	contig_6	979316	981905	-	VTC4	vacuolar transporter chaperone
FUN_004738	contig_6	982728	983831	+		hypothetical protein
FUN_004739	contig_6	984442	986963	-		hypothetical protein
FUN_004741	contig_6	992025	993150	-		hypothetical protein
FUN_004742	contig_6	994292	996202	+		hypothetical protein
FUN_004743	contig_6	997097	999104	+		hypothetical protein
FUN_004744	contig_6	999731	1000958	-		hypothetical protein
FUN_004745	contig_6	1002989	1004318	-		hypothetical protein
FUN_004747	contig_6	1010711	1011820	+		hypothetical protein
FUN_004748	contig_6	1012537	1013866	-	FEN1	Elongation of fatty acids protein 2
FUN_004749	contig_6	1014475	1015905	+		hypothetical protein
FUN_004750	contig_6	1016691	1017527	+	UBC1	Ubiquitin-conjugating enzyme E2 1
FUN_004751	contig_6	1021130	1024325	+	TPS2	threalose-6-phosphate phosphatase
FUN_004752	contig_6	1026847	1029223	+		hypothetical protein
FUN_004754	contig_6	1031340	1031773	-	RPL28	60S ribosomal protein L28
FUN_004755	contig_6	1032355	1035381	-	SFB3	COPII coat Sec23p-Sfb3p heterodimer component
FUN_004756	contig_6	1036335	1037126	-	aps2	AP-2 complex subunit sigma
FUN_004757	contig_6	1037595	1038271	-		hypothetical protein
FUN_004758	contig_6	1042209	1043806	-	SGA1	Glucoamylase, intracellular sporulation-specific
FUN_004760	contig_6	1048230	1048805	+		hypothetical protein
FUN_004761	contig_6	1048835	1049917	-		hypothetical protein
FUN_004762	contig_6	1050447	1055021	+		hypothetical protein
FUN_004763	contig_6	1056715	1058546	-		hypothetical protein
FUN_004764	contig_6	1059156	1061057	+	cct1	chaperonin-containing T-complex alpha subunit Cct1
FUN_004765	contig_6	1062147	1063328	+		hypothetical protein
FUN_004768	contig_6	1078186	1079277	+		hypothetical protein
FUN_004769	contig_6	1079839	1081234	-	ATG18	autophagy protein
FUN_004770	contig_6	1081571	1084917	+	PPM2	tRNA methyltransferase ppm2
FUN_004771	contig_6	1086717	1087649	-		hypothetical protein
FUN_004772	contig_6	1088198	1088895	+		hypothetical protein

Gene ID	Scaffold	Start	Stop	Strand	Name	Product
FUN_004773	contig_6	1090495	1092481	+	PPS1	tyrosine/serine/threonine protein phosphatase pps1
FUN_004774	contig_6	1093869	1095423	+		hypothetical protein
FUN_004775	contig_6	1096021	1096760	+	YUH1	ubiquitinyl hydrolase 1
FUN_004776	contig_6	1097979	1099284	+	GPR1	G protein-coupled receptor gpr1
FUN_004777	contig_6	1099668	1101803	-		hypothetical protein
FUN_004778	contig_6	1103437	1104693	+	GAL10	UDP-glucose-4-epimerase
FUN_004779	contig_6	1105276	1106646	+	ARC1	G4 quadruplex nucleic acid binding protein
FUN_004781	contig_6	1109835	1110886	+	LIA1	deoxyhypusine hydroxylase
FUN_004782	contig_6	1111067	1111528	-	RPA12	DNA-directed RNA polymerase I core subunit rpa12
FUN_004783	contig_6	1112001	1117684	+	SCC2	Sister chromatid cohesion protein 2
FUN_004784	contig_6	1118322	1121737	+	FIG4	phosphatidylinositol-3,5-bisphosphate 5-phosphatase
FUN_004785	contig_6	1121821	1123667	-		hypothetical protein
FUN_004787	contig_6	1129101	1130305	+		hypothetical protein
FUN_004789	contig_6	1131746	1132545	-		hypothetical protein
FUN_004790	contig_6	1132925	1134847	-		hypothetical protein
FUN_004791	contig_6	1136551	1137370	+	SPC25	kinetochore-associated Ndc80 complex subunit spc25
FUN_004792	contig_6	1137502	1139725	-		hypothetical protein
FUN_004793	contig_6	1140070	1141964	-		hypothetical protein
FUN_004795	contig_6	1145768	1148947	+		hypothetical protein
FUN_004797	contig_6	1152113	1152918	-		hypothetical protein
FUN_004801	contig_6	1167131	1169686	-		hypothetical protein
FUN_004802	contig_6	1171028	1171626	+		hypothetical protein
FUN_004806	contig_6	1178342	1179666	+		hypothetical protein
FUN_004809	contig_6	1182540	1195661	-	DYN1	dynein heavy chain
FUN_004810	contig_6	1196241	1198966	-	GEF1	glycerol ethanol, ferric requiring protein
FUN_004811	contig_6	1199509	1201038	-		hypothetical protein
FUN_004812	contig_6	1201489	1202232	+		hypothetical protein
FUN_004813	contig_6	1202400	1203117	-	POA1	ADP-ribose 1''-phosphate phosphatase
FUN_004814	contig_6	1203594	1204594	+	RIP1	Cytochrome b-c1 complex subunit Rieske, mitochondrial
FUN_004815	contig_6	1205071	1206047	-	BCP1	Mss4p nuclear export
FUN_004816	contig_6	1206269	1207952	+	GUA1	GMP synthase (glutamine-hydrolyzing)
FUN_004818	contig_6	1210968	1211759	+		hypothetical protein
FUN_004819	contig_6	1212646	1213564	+		hypothetical protein
FUN_004821	contig_6	1214803	1216250	-		hypothetical protein
FUN_004824	contig_6	1223618	1226009	+		hypothetical protein
FUN_004825	contig_6	1226396	1227099	-	EMP24	p24 complex component
FUN_004826	contig_6	1227594	1228827	+		hypothetical protein
FUN_004827	contig_6	1229258	1231078	-		hypothetical protein
FUN_004832	contig_6	1243802	1245389	-	MUP3	low-affinity methionine permease
FUN_004833	contig_6	1248917	1251537	+		hypothetical protein
FUN_004834	contig_6	1252080	1254281	-		hypothetical protein
FUN_004835	contig_6	1255021	1257537	-	dpp4	Dipeptidyl peptidase 4
FUN_004836	contig_6	1259108	1261770	-		hypothetical protein
FUN_004837	contig_6	1266289	1268286	+		hypothetical protein
FUN_004838	contig_6	1268745	1269453	+		hypothetical protein
FUN_004839	contig_6	1271351	1272486	-	ERG6_1	Delta(24)-sterol C-methyltransferase
FUN_004840	contig_6	1272911	1274280	-		hypothetical protein
FUN_004842	contig_6	1284255	1285008	+		hypothetical protein
FUN_004844	contig_6	1286469	1287404	-		hypothetical protein
FUN_004845	contig_6	1287798	1289435	+		hypothetical protein
FUN_004846	contig_6	1289630	1290844	-		hypothetical protein
FUN_004848	contig_6	1292740	1293360	-		hypothetical protein
FUN_004849	contig_6	1296117	1297674	-	LAP2_1	Leucyl aminopeptidase yscIV
FUN_004851	contig_6	1301173	1301976	-		hypothetical protein
FUN_004852	contig_6	1303462	1304114	+		hypothetical protein
FUN_004853	contig_6	1305688	1307122	+		hypothetical protein
FUN_004856	contig_6	1312726	1314319	-		hypothetical protein
FUN_004857	contig_6	1315025	1318898	-	PMC1_1	plasma membrane calcium
FUN_004860	contig_6	1324217	1325533	+		hypothetical protein

Gene ID	Scaffold	Start	Stop	Strand	Name	Product
FUN_004861	contig_6	1328412	1331316	-		hypothetical protein
FUN_004863	contig_6	1334066	1336554	-		hypothetical protein
FUN_004864	contig_6	1339784	1340834	+		hypothetical protein
FUN_004865	contig_6	1344097	1345885	+		hypothetical protein
FUN_004867	contig_6	1348738	1351939	+	UBA1	E1 ubiquitin-activating protein
FUN_004868	contig_6	1352260	1353351	+	NBP35	cytosolic Fe-S cluster assembly factor nbp35
FUN_004872	contig_6	1361363	1362604	-		hypothetical protein
FUN_004873	contig_6	1362770	1363652	+	TM1	ToMV susceptible protein tm-1(GCR26)
FUN_004874	contig_6	1365812	1366640	+		hypothetical protein
FUN_004875	contig_6	1367242	1368096	+		hypothetical protein
FUN_004876	contig_6	1369732	1370832	+	ERG26_2	erg26, C-3 sterol dehydrogenase
FUN_004877	contig_6	1379540	1380099	+		hypothetical protein
FUN_004878	contig_6	1381636	1382530	-		hypothetical protein
FUN_004879	contig_6	1383475	1384930	+		hypothetical protein
FUN_004880	contig_6	1385166	1386493	-		hypothetical protein
FUN_004881	contig_6	1386997	1388552	+		hypothetical protein
FUN_004882	contig_6	1388938	1391335	-		hypothetical protein
FUN_004883	contig_6	1391576	1392837	-		hypothetical protein
FUN_004887	contig_6	1401599	1403284	-	AMD2	Acetamidase
FUN_004888	contig_6	1403760	1405266	+		hypothetical protein
FUN_004890	contig_6	1408425	1410449	-		hypothetical protein
FUN_004891	contig_6	1412376	1413577	+		hypothetical protein
FUN_004892	contig_6	1413906	1415057	+		hypothetical protein
FUN_004894	contig_6	1417400	1417946	-		hypothetical protein
FUN_004895	contig_6	1418829	1420463	+		hypothetical protein
FUN_004896	contig_6	1421445	1423324	-	HMLALPHA2_2	homeodomain mating type protein alpha2
FUN_004897	contig_6	1425999	1426669	+		hypothetical protein
FUN_004900	contig_6	1430712	1433284	+		hypothetical protein
FUN_004901	contig_6	1434310	1436771	-		hypothetical protein
FUN_004902	contig_6	1437782	1440335	+		hypothetical protein
FUN_004906	contig_6	1448844	1450901	+		hypothetical protein
FUN_004907	contig_6	1451414	1453232	+		hypothetical protein
FUN_004909	contig_6	1455923	1458643	+		hypothetical protein
FUN_004910	contig_6	1459565	1461241	+		hypothetical protein
FUN_004912	contig_6	1464310	1464832	+		hypothetical protein
FUN_004913	contig_6	1466285	1468425	+	dpp5_1	Dipeptidyl-peptidase 5
FUN_004914	contig_6	1469969	1471976	+		hypothetical protein
FUN_004916	contig_6	1479588	1481671	-		hypothetical protein
FUN_004917	contig_6	1482062	1483291	-		hypothetical protein
FUN_004919	contig_6	1491117	1492319	-	pan6	pantoate-beta-alanine ligase
FUN_004920	contig_6	1492639	1495089	+	DBP7	ATP-dependent RNA helicase dbp7
FUN_004922	contig_6	1497074	1498649	+		hypothetical protein
FUN_004923	contig_6	1501786	1502374	+		hypothetical protein
FUN_004924	contig_6	1506104	1508521	-	NOP4	RNA recognition motif-containing protein
FUN_004926	contig_6	1511326	1515602	+		hypothetical protein
FUN_004927	contig_6	1516805	1518582	+		hypothetical protein
FUN_004928	contig_6	1519814	1520377	+	RPL9B	60S ribosomal protein L9B
FUN_004929	contig_6	1520947	1522140	+		hypothetical protein
FUN_004932	contig_6	1527393	1529531	+		hypothetical protein
FUN_004933	contig_6	1530853	1531790	+	FAE1	Feruloyl esterase B
FUN_004934	contig_6	1537061	1538679	-		hypothetical protein
FUN_004935	contig_6	1539572	1541278	-		hypothetical protein
FUN_004936	contig_6	1542026	1543524	+	CUE5	ubiquitin-binding protein cue5
FUN_004938	contig_6	1545632	1546660	-		hypothetical protein
FUN_004939	contig_6	1547651	1548725	+		hypothetical protein
FUN_004940	contig_6	1549004	1550460	-		hypothetical protein
FUN_004943	contig_6	1561644	1563019	+		hypothetical protein
FUN_004945	contig_6	1566961	1567847	+		hypothetical protein
FUN_004948	contig_6	1572661	1575039	-		hypothetical protein

Gene ID	Scaffold	Start	Stop	Strand	Name	Product
FUN_004949	contig_6	1576835	1577179	+		hypothetical protein
FUN_004950	contig_6	1577647	1578384	-		hypothetical protein
FUN_004951	contig_6	1578811	1579563	-	VPS20	Vacuolar protein sorting-associated protein 20
FUN_004952	contig_6	1579799	1582143	+	RIX7	Ribosome bioproteinsis ATPase rix7
FUN_004959	contig_6	1593921	1595883	-		hypothetical protein
FUN_004960	contig_6	1596480	1597253	+		hypothetical protein
FUN_004962	contig_6	1599255	1600532	+		hypothetical protein
FUN_004963	contig_6	1600948	1602447	-		hypothetical protein
FUN_004964	contig_6	1604496	1605930	+		hypothetical protein
FUN_004965	contig_6	1606412	1607294	+		hypothetical protein
FUN_004966	contig_6	1607674	1608669	-		hypothetical protein
FUN_004967	contig_6	1609201	1609388	+		hypothetical protein
FUN_004968	contig_6	1610242	1611816	-		hypothetical protein
FUN_004969	contig_6	1616118	1616873	+		hypothetical protein
FUN_004970	contig_6	1617011	1618206	+		hypothetical protein
FUN_004971	contig_6	1618568	1619925	-		hypothetical protein
FUN_004972	contig_6	1620466	1621874	+		hypothetical protein
FUN_004973	contig_6	1622061	1623932	-		hypothetical protein
FUN_004974	contig_6	1624634	1626967	-		hypothetical protein
FUN_004975	contig_6	1628425	1630477	+		hypothetical protein
FUN_004977	contig_6	1633129	1634006	-		hypothetical protein
FUN_004978	contig_6	1634504	1635839	-		hypothetical protein
FUN_004979	contig_6	1638570	1639517	-		hypothetical protein
FUN_004980	contig_6	1642278	1644487	+		hypothetical protein
FUN_004981	contig_6	1645372	1647350	+		hypothetical protein
FUN_004983	contig_6	1651914	1653649	-		hypothetical protein
FUN_004984	contig_6	1654266	1656027	-		hypothetical protein
FUN_004985	contig_6	1656378	1657424	-	LYS4_1	mitochondrial Homoaconitase
FUN_004986	contig_6	1658058	1658780	+		hypothetical protein
FUN_004987	contig_6	1659283	1661869	+	CWH41_2	Processing alpha glucosidase I
FUN_004988	contig_6	1662224	1663188	-		hypothetical protein
FUN_004990	contig_6	1664534	1665913	+		hypothetical protein
FUN_004993	contig_6	1672750	1674135	-		hypothetical protein
FUN_004994	contig_6	1674504	1675293	+		hypothetical protein
FUN_004995	contig_6	1676203	1678073	-		hypothetical protein
FUN_004998	contig_6	1688250	1689633	-		hypothetical protein
FUN_004999	contig_6	1691837	1693018	+	hus1	Checkpoint protein hus1
FUN_005000	contig_6	1702126	1704381	-	MSS1	mitochondrial splicing system protein
FUN_005001	contig_6	1704847	1706142	+	mak16	Protein MAK16
FUN_005004	contig_6	1710285	1712096	+		hypothetical protein
FUN_005005	contig_6	1714290	1715383	+	CDKN2B	Cyclin-dependent kinase
FUN_005006	contig_6	1722403	1724316	+		hypothetical protein
FUN_005007	contig_6	1727063	1728078	-		hypothetical protein
FUN_005008	contig_6	1732304	1734595	+		hypothetical protein
FUN_005009	contig_6	1735253	1736840	+		hypothetical protein
FUN_005010	contig_6	1736911	1737792	-		hypothetical protein
FUN_005011	contig_6	1738101	1739496	-		hypothetical protein
FUN_005012	contig_6	1740943	1741544	+		hypothetical protein
FUN_005013	contig_6	1741842	1743021	-		hypothetical protein
FUN_005014	contig_6	1743575	1745516	+		hypothetical protein
FUN_005015	contig_6	1746138	1747206	+		hypothetical protein
FUN_005016	contig_6	1747691	1748410	+		hypothetical protein
FUN_005019	contig_6	1757191	1758061	-		hypothetical protein
FUN_005020	contig_6	1758486	1759337	+		hypothetical protein
FUN_005021	contig_6	1759481	1760228	-		hypothetical protein
FUN_005023	contig_6	1765979	1767693	-		hypothetical protein
FUN_005024	contig_6	1770265	1771441	+		hypothetical protein
FUN_005026	contig_6	1777035	1778675	-		hypothetical protein
FUN_005027	contig_6	1779137	1780605	+		hypothetical protein

Gene ID	Scaffold	Start	Stop	Strand	Name	Product
FUN_005028	contig_6	1780704	1781906	-		hypothetical protein
FUN_005029	contig_6	1782271	1783811	+		hypothetical protein
FUN_005031	contig_6	1785852	1787655	-	HOM3	Aspartokinase
FUN_005032	contig_6	1787773	1789132	-	MET2	L-homoserine-O-acetyltransferase
FUN_005033	contig_6	1790334	1791547	+	NIT1	Nitrilase
FUN_005035	contig_6	1795169	1796773	+	MET15_1	Homocysteine/cysteine synthase
FUN_005037	contig_6	1799564	1802207	+		hypothetical protein
FUN_005038	contig_6	1802670	1804058	+		hypothetical protein
FUN_005039	contig_6	1804674	1805513	-		hypothetical protein
FUN_005040	contig_6	1807369	1810649	-		hypothetical protein
FUN_005045	contig_6	1827223	1828728	+	FDH1_1	formate dehydrogenase (NAD+)
FUN_005047	contig_6	1830251	1835610	+		hypothetical protein
FUN_005050	contig_6	1841809	1843612	+		hypothetical protein
FUN_005051	contig_6	1845683	1847222	-		hypothetical protein
FUN_005054	contig_6	1851656	1853092	-		hypothetical protein
FUN_005055	contig_6	1853587	1854455	+		hypothetical protein
FUN_005057	contig_6	1857158	1858819	-		hypothetical protein
FUN_005058	contig_6	1860198	1862273	-		hypothetical protein
FUN_005059	contig_6	1862855	1864215	-		hypothetical protein
FUN_005060	contig_6	1865906	1867819	-		hypothetical protein
FUN_005061	contig_6	1868504	1869972	+		hypothetical protein
FUN_005062	contig_6	1870489	1873419	-	PLK5	Inactive serine/threonine-protein kinase plk5
FUN_005063	contig_6	1874576	1876237	+		hypothetical protein
FUN_005064	contig_7	4252	5442	-		hypothetical protein
FUN_005065	contig_7	5995	6629	-		hypothetical protein
FUN_005066	contig_7	8632	10484	+		hypothetical protein
FUN_005067	contig_7	10644	11718	-		hypothetical protein
FUN_005068	contig_7	13545	14636	+		hypothetical protein
FUN_005069	contig_7	16239	17427	+		hypothetical protein
FUN_005071	contig_7	23114	24157	-		hypothetical protein
FUN_005072	contig_7	25512	27490	+		hypothetical protein
FUN_005073	contig_7	27653	28494	-	cwf29	RNA-binding protein Cwf29
FUN_005074	contig_7	28789	30497	+		hypothetical protein
FUN_005075	contig_7	32658	33779	-		hypothetical protein
FUN_005076	contig_7	39762	41166	-	CDC11	Cell division control protein 11
FUN_005077	contig_7	42294	43573	+	HSV2	Phosphatidylinositol 3,5-bisphosphate-binding protein
FUN_005078	contig_7	44240	45103	-		hypothetical protein
FUN_005079	contig_7	45455	46041	+	MRPS16	37S ribosomal protein S16, mitochondrial
FUN_005080	contig_7	46332	48016	-		hypothetical protein
FUN_005081	contig_7	48437	48975	+		hypothetical protein
FUN_005082	contig_7	51419	52003	+	SHO1	Transmembrane osmosensor
FUN_005083	contig_7	53236	54161	+		hypothetical protein
FUN_005084	contig_7	55078	56698	+	lag1	Sphingosine N-acyltransferase lag1
FUN_005085	contig_7	57063	59004	+	MCCC2	Methylcrotonoyl-CoA carboxylase beta chain, mitochondrial
FUN_005086	contig_7	59427	60840	-		hypothetical protein
FUN_005088	contig_7	64136	65518	-		hypothetical protein
FUN_005089	contig_7	65763	66353	-		hypothetical protein
FUN_005090	contig_7	66778	67807	-		hypothetical protein
FUN_005091	contig_7	68322	69542	+		hypothetical protein
FUN_005092	contig_7	69999	71741	+		hypothetical protein
FUN_005093	contig_7	72238	74526	-		hypothetical protein
FUN_005094	contig_7	75319	77459	+		hypothetical protein
FUN_005095	contig_7	77741	79216	+	SQT1	60S ribosomal subunit assembly or modification protein
FUN_005096	contig_7	86602	87236	+		hypothetical protein
FUN_005099	contig_7	91689	93545	+		hypothetical protein
FUN_005101	contig_7	95938	99724	+	SMC3	Structural maintenance of chromosomes protein 3
FUN_005102	contig_7	100559	101863	-		hypothetical protein
FUN_005104	contig_7	108393	109493	+		hypothetical protein

Gene ID	Scaffold	Start	Stop	Strand	Name	Product
FUN_005106	contig_7	110560	111699	-		hypothetical protein
FUN_005108	contig_7	113881	114530	-		hypothetical protein
FUN_005109	contig_7	115084	116323	-		hypothetical protein
FUN_005110	contig_7	116752	117758	-		hypothetical protein
FUN_005111	contig_7	118376	120028	+		hypothetical protein
FUN_005112	contig_7	120431	121730	+		hypothetical protein
FUN_005119	contig_7	144525	145316	+		hypothetical protein
FUN_005123	contig_7	149683	150344	-		hypothetical protein
FUN_005124	contig_7	150388	150972	-		hypothetical protein
FUN_005125	contig_7	152241	155015	+		hypothetical protein
FUN_005128	contig_7	165793	166951	+		hypothetical protein
FUN_005135	contig_7	180277	188439	-	BPH1	Beige protein-like 1
FUN_005136	contig_7	189253	191118	+	PRB1	proteinase B
FUN_005137	contig_7	192030	194351	-		hypothetical protein
FUN_005138	contig_7	194993	196648	+		hypothetical protein
FUN_005141	contig_7	201410	201908	-		hypothetical protein
FUN_005142	contig_7	203654	206155	-	lub1	WD repeat protein Lub1
FUN_005143	contig_7	208245	209599	+		hypothetical protein
FUN_005144	contig_7	213505	215207	+		hypothetical protein
FUN_005145	contig_7	215769	216658	+		hypothetical protein
FUN_005147	contig_7	220274	222094	-		hypothetical protein
FUN_005148	contig_7	223073	225229	-		hypothetical protein
FUN_005150	contig_7	229627	231180	-	rio1	Serine/threonine-protein kinase rio1
FUN_005151	contig_7	231642	233175	-	atg17	Autophagy-related protein 17
FUN_005152	contig_7	234164	236390	+		hypothetical protein
FUN_005153	contig_7	237924	239164	-		hypothetical protein
FUN_005154	contig_7	239820	241769	+		hypothetical protein
FUN_005155	contig_7	251081	252058	+	PFA5	palmitoyltransferase pfa5
FUN_005156	contig_7	266908	267847	-	YAF9	NuA4 histone H4 acetyltransferase complex and the SWR1 complex subunit
FUN_005157	contig_7	268158	269253	+		hypothetical protein
FUN_005158	contig_7	270998	273099	-	TGL1	cholesterol esterase
FUN_005159	contig_7	273570	274063	+		hypothetical protein
FUN_005161	contig_7	277570	279365	+	ACTT5	Acyl-CoA synthetase actt5
FUN_005168	contig_7	301670	304786	-		hypothetical protein
FUN_005170	contig_7	306635	308214	-		hypothetical protein
FUN_005171	contig_7	310271	311083	-		hypothetical protein
FUN_005172	contig_7	311521	315284	+		hypothetical protein
FUN_005173	contig_7	325244	330027	-		hypothetical protein
FUN_005174	contig_7	330519	331314	+		hypothetical protein
FUN_005175	contig_7	331807	332852	+		hypothetical protein
FUN_005176	contig_7	336517	337065	+		hypothetical protein
FUN_005177	contig_7	337846	338792	+	HBN1	Putative nitroreductase
FUN_005178	contig_7	339375	340316	-		hypothetical protein
FUN_005179	contig_7	341626	343911	+		hypothetical protein
FUN_005180	contig_7	353200	353731	+		hypothetical protein
FUN_005181	contig_7	358832	360119	+	mns1B	Mannosyl-oligosaccharide alpha-1,2-mannosidase 1B
FUN_005183	contig_7	364070	364714	-		hypothetical protein
FUN_005184	contig_7	366554	367200	+		hypothetical protein
FUN_005186	contig_7	367732	368694	-		hypothetical protein
FUN_005187	contig_7	369150	370094	-	PRA1	Prenylated Rab acceptor protein 1
FUN_005188	contig_7	371349	372529	+	ZRT1	high-affinity Zn(2+) transporter zrt1
FUN_005190	contig_7	378183	379359	-		hypothetical protein
FUN_005191	contig_7	380308	381788	-		hypothetical protein
FUN_005192	contig_7	382566	384083	+		hypothetical protein
FUN_005193	contig_7	385550	387078	-		hypothetical protein
FUN_005195	contig_7	391966	393017	+		hypothetical protein
FUN_005196	contig_7	393166	394578	-		hypothetical protein
FUN_005197	contig_7	396323	397822	+		hypothetical protein

Gene ID	Scaffold	Start	Stop	Strand	Name	Product
FUN_005198	contig_7	398270	399385	+		hypothetical protein
FUN_005199	contig_7	400860	404156	+		hypothetical protein
FUN_005201	contig_7	408426	409400	+		hypothetical protein
FUN_005204	contig_7	420536	421507	+		hypothetical protein
FUN_005205	contig_7	421632	422792	-		hypothetical protein
FUN_005207	contig_7	424923	426284	+		hypothetical protein
FUN_005209	contig_7	433398	435361	+		hypothetical protein
FUN_005211	contig_7	440566	444236	-		hypothetical protein
FUN_005212	contig_7	445310	447017	+		hypothetical protein
FUN_005213	contig_7	447611	451123	+		hypothetical protein
FUN_005214	contig_7	460368	460958	-	ats1	Peroxygenase 1
FUN_005215	contig_7	461098	462315	-		hypothetical protein
FUN_005216	contig_7	462791	465348	+	SWI6	transcriptional regulator swi6
FUN_005217	contig_7	465739	467526	-		hypothetical protein
FUN_005218	contig_7	468113	469929	+		hypothetical protein
FUN_005220	contig_7	477714	479738	+		hypothetical protein
FUN_005221	contig_7	480596	482283	+		hypothetical protein
FUN_005222	contig_7	482890	484282	+	SPO7	Nem1-Spo7 phosphatase regulatory subunit
FUN_005223	contig_7	490164	491353	-		hypothetical protein
FUN_005224	contig_7	491822	492415	+		hypothetical protein
FUN_005227	contig_7	496705	497840	+		hypothetical protein
FUN_005229	contig_7	499751	500571	-		hypothetical protein
FUN_005230	contig_7	504696	505870	+		hypothetical protein
FUN_005232	contig_7	507114	508473	-		hypothetical protein
FUN_005233	contig_7	510047	511620	+		hypothetical protein
FUN_005234	contig_7	512160	513883	+		hypothetical protein
FUN_005237	contig_7	522317	524005	-		hypothetical protein
FUN_005238	contig_7	526696	528365	-		hypothetical protein
FUN_005239	contig_7	528713	531283	-	PLA2G4F	Cytosolic phospholipase A2 zeta
FUN_005241	contig_7	535048	537086	+	EXO70	exocyst complex component exo70
FUN_005242	contig_7	538020	539687	+	CDC3	Cell division control protein 3
FUN_005243	contig_7	540660	541544	+	POM33	Transmembrane nucleoporin
FUN_005245	contig_7	546174	549085	+	GPH1	Non-essential glycogen phosphorylase
FUN_005246	contig_7	549407	550483	+		hypothetical protein
FUN_005247	contig_7	551238	553376	+		hypothetical protein
FUN_005249	contig_7	556831	558433	+		hypothetical protein
FUN_005250	contig_7	563091	565337	-		hypothetical protein
FUN_005251	contig_7	565542	571086	-	ctf18	Chromosome transmission fidelity protein 18
FUN_005252	contig_7	576674	577571	+		hypothetical protein
FUN_005253	contig_7	579480	580502	-		hypothetical protein
FUN_005254	contig_7	581510	586563	+		hypothetical protein
FUN_005258	contig_7	592104	593162	+		hypothetical protein
FUN_005265	contig_7	606425	608143	+		hypothetical protein
FUN_005267	contig_7	610309	611226	-		hypothetical protein
FUN_005268	contig_7	612464	615293	-	IRC5	Putative ATPase
FUN_005269	contig_7	615605	616138	-		hypothetical protein
FUN_005270	contig_7	617059	618454	+		hypothetical protein
FUN_005271	contig_7	620233	621787	+		hypothetical protein
FUN_005272	contig_7	621959	623643	-		hypothetical protein
FUN_005276	contig_7	632279	633520	+		hypothetical protein
FUN_005280	contig_7	640434	641708	-		hypothetical protein
FUN_005281	contig_7	642105	642943	-	MET8	Bifunctional dehydrogenase and ferrochelatase
FUN_005282	contig_7	643269	645397	+	PEP7	carboxypeptidase Y-deficient
FUN_005284	contig_7	650426	653669	-	SEC5	Exocyst complex component S5
FUN_005286	contig_7	657333	658283	-		hypothetical protein
FUN_005287	contig_7	659159	660212	+		hypothetical protein
FUN_005289	contig_7	664294	666037	+		hypothetical protein
FUN_005290	contig_7	666216	668335	-		hypothetical protein
FUN_005291	contig_7	668982	669865	-		hypothetical protein

Gene ID	Scaffold	Start	Stop	Strand	Name	Product
FUN_005292	contig_7	670368	671879	+		hypothetical protein
FUN_005293	contig_7	674393	676484	-		hypothetical protein
FUN_005296	contig_7	682161	684177	+	GLN4	Glutaminyl-tRNA synthetase
FUN_005297	contig_7	685357	687485	+		hypothetical protein
FUN_005298	contig_7	687724	691648	+	SPF1	putative cation-transporting ATPase 1
FUN_005300	contig_7	694148	695405	+		hypothetical protein
FUN_005301	contig_7	697583	699721	-		hypothetical protein
FUN_005302	contig_7	704774	706660	-	YVC1	Calcium channel yvc1
FUN_005303	contig_7	707324	708313	-		hypothetical protein
FUN_005317	contig_7	747199	749024	-		hypothetical protein
FUN_005318	contig_7	751229	752325	+		hypothetical protein
FUN_005320	contig_7	758574	759975	+		hypothetical protein
FUN_005321	contig_7	762179	763923	-		hypothetical protein
FUN_005322	contig_7	765778	766839	+		hypothetical protein
FUN_005323	contig_7	768151	770641	-	TOK1_1	Potassium channel
FUN_005327	contig_7	780566	782412	+		hypothetical protein
FUN_005329	contig_7	786665	788508	+		hypothetical protein
FUN_005330	contig_7	788740	789403	-		hypothetical protein
FUN_005335	contig_7	805632	807054	+		hypothetical protein
FUN_005337	contig_7	809284	811339	-		hypothetical protein
FUN_005338	contig_7	811963	812988	+	SET5	SET domain-containing protein 5
FUN_005340	contig_7	814271	816298	-		hypothetical protein
FUN_005341	contig_7	818403	819763	-		hypothetical protein
FUN_005342	contig_7	821123	822442	-	LYS1	Saccharopine dehydrogenase
FUN_005343	contig_7	823031	825626	+		hypothetical protein
FUN_005344	contig_7	827063	828635	+	JEN1_1	Carboxylic acid transporter
FUN_005345	contig_7	829208	830020	+		hypothetical protein
FUN_005347	contig_7	832630	833445	+		hypothetical protein
FUN_005349	contig_7	835602	837132	-		hypothetical protein
FUN_005350	contig_7	840199	841153	-		hypothetical protein
FUN_005355	contig_7	848527	849756	-	mrpl3	54S ribosomal protein L3 mitochondrial
FUN_005356	contig_7	850411	851570	-		hypothetical protein
FUN_005357	contig_7	852199	853887	+	SRV2	suppressor of rasval19
FUN_005358	contig_7	855346	856983	-		hypothetical protein
FUN_005361	contig_7	860992	862740	+		hypothetical protein
FUN_005362	contig_7	866417	867268	-		hypothetical protein
FUN_005363	contig_7	867507	870334	+		hypothetical protein
FUN_005364	contig_7	870660	871841	+		hypothetical protein
FUN_005365	contig_7	873341	876836	+	BST1	GPI inositol deacylase
FUN_005366	contig_7	877636	878507	+	BOS1	Protein transport protein bos1
FUN_005369	contig_7	884983	887031	+		hypothetical protein
FUN_005370	contig_7	888029	891696	+		hypothetical protein
FUN_005371	contig_7	891867	893925	-	irc3	Putative ATP-dependent helicase IRC3
FUN_005372	contig_7	894141	896156	+		hypothetical protein
FUN_005373	contig_7	896689	898137	+		hypothetical protein
FUN_005375	contig_7	901566	902843	-		hypothetical protein
FUN_005376	contig_7	903356	909334	-	SEC7	guanine nucleotide exchange protein for ADP-robosylation factor
FUN_005377	contig_7	909968	911632	+	LAT1	pyruvate dehydrogenase complex dihydrolipoamide acetyltransferase component (E2)
FUN_005378	contig_7	911957	914126	-		hypothetical protein
FUN_005379	contig_7	915273	915719	-		hypothetical protein
FUN_005380	contig_7	916220	917239	+	MIA40	Oxidoreductase
FUN_005381	contig_7	917474	919833	-	TOK1_2	Potassium channel
FUN_005382	contig_7	920416	921571	-	ETF1	Electron transfer flavoprotein alpha-subunit
FUN_005383	contig_7	922315	924592	+	ssr2	SWI/SNF and RSC complex subunit Ssr2
FUN_005384	contig_7	928194	933220	+	SMY2	kinesin-like protein
FUN_005385	contig_7	933768	934407	-	spc24	putative kinetochore protein spc24
FUN_005386	contig_7	934793	935301	+	FIS1	Mitochondrial fission 1 protein

Gene ID	Scaffold	Start	Stop	Strand	Name	Product
FUN_005387	contig_7	936192	937990	+		hypothetical protein
FUN_005388	contig_7	939864	944976	+		hypothetical protein
FUN_005390	contig_7	948900	952187	-	FUN12	eukaryotic translation initiation factor 5B
FUN_005391	contig_7	954050	959617	-		hypothetical protein
FUN_005392	contig_7	961473	966876	+		hypothetical protein
FUN_005393	contig_7	967342	968605	+	END3	endocytosis defective-related protein
FUN_005394	contig_7	972610	975354	+	CHS7_2	Chitin synthase, class 7
FUN_005395	contig_7	980716	983030	-	KRE6_1	beta-glucan synthesis-associated protein
FUN_005396	contig_7	984310	985119	-	THI80	thiamine pyrophosphokinase
FUN_005398	contig_7	1000576	1001822	+		hypothetical protein
FUN_005399	contig_7	1003818	1007436	+	NMD2	mRNA decay protein
FUN_005400	contig_7	1009009	1009890	+		hypothetical protein
FUN_005401	contig_7	1010344	1011873	-		hypothetical protein
FUN_005402	contig_7	1012242	1014126	-		hypothetical protein
FUN_005403	contig_7	1015835	1017797	+	CAT2	Carnitine O-acetyltransferase mitochondrial
FUN_005404	contig_7	1018486	1019816	-	MDH1	Malate dehydrogenase, cytoplasmic
FUN_005408	contig_7	1030015	1030921	-		hypothetical protein
FUN_005410	contig_7	1035029	1036460	+	ARH1	NADPH-adrenodoxin reductase
FUN_005411	contig_7	1038325	1041576	+	TEF3	translational elongation factor EF-1 alpha
FUN_005412	contig_7	1042559	1044044	+		hypothetical protein
FUN_005413	contig_7	1044960	1045316	-		hypothetical protein
FUN_005414	contig_7	1045687	1049326	+	BMS1	Glycoside hydrolase 2 (Mannanase, beta-galactosidase)
FUN_005415	contig_7	1049565	1051517	-	ACH1	acetyl-CoA hydrolase
FUN_005416	contig_7	1052075	1053049	+		hypothetical protein
FUN_005417	contig_7	1053429	1054668	-	CIC1	proteasome-interacting protein cic1
FUN_005418	contig_7	1056017	1057765	+	TOS4	target of SBF
FUN_005422	contig_7	1067854	1069692	-		hypothetical protein
FUN_005423	contig_7	1070333	1071063	-	ANB1	translation initiation factor eIF5A
FUN_005424	contig_7	1071401	1073504	+	VPS52	Vacuolar protein sorting-associated protein 52
FUN_005425	contig_7	1074370	1084171	-	CSF1	Macrophage colony-stimulating factor 1 receptor
FUN_005427	contig_7	1097782	1099720	+		hypothetical protein
FUN_005428	contig_7	1105600	1107237	-	SDH1_3	succinate dehydrogenase flavoprotein subunit
FUN_005430	contig_7	1111699	1112789	-		hypothetical protein
FUN_005431	contig_7	1113335	1114646	+		hypothetical protein
FUN_005432	contig_7	1116823	1119384	+	CDC53	ubiquitin ligase (cullin) of SCF
FUN_005434	contig_7	1122180	1123211	-		hypothetical protein
FUN_005435	contig_7	1123975	1125522	-		hypothetical protein
FUN_005436	contig_7	1126947	1129128	-	CTK2	RNA polymerase II C-terminal domain kinase beta subunit
FUN_005437	contig_7	1129620	1130893	+		hypothetical protein
FUN_005438	contig_7	1130979	1132165	-		hypothetical protein
FUN_005439	contig_7	1134349	1135037	+		hypothetical protein
FUN_005440	contig_7	1137462	1138790	+		hypothetical protein
FUN_005442	contig_7	1145399	1146756	-		hypothetical protein
FUN_005443	contig_7	1147658	1148953	+		hypothetical protein
FUN_005445	contig_7	1150311	1153085	-		hypothetical protein
FUN_005446	contig_7	1153514	1154355	-		hypothetical protein
FUN_005448	contig_7	1155702	1156676	-	JHD1_1	JmjC domain-containing histone demethylation protein 1
FUN_005449	contig_7	1160532	1161681	-		hypothetical protein
FUN_005450	contig_7	1162120	1162898	+		hypothetical protein
FUN_005452	contig_7	1165054	1166007	+		hypothetical protein
FUN_005453	contig_7	1166773	1168505	+		hypothetical protein
FUN_005454	contig_7	1169427	1170521	+	THI4	thiamine metabolism-related protein
FUN_005456	contig_7	1173958	1174621	-		hypothetical protein
FUN_005457	contig_7	1175455	1178632	-		hypothetical protein
FUN_005459	contig_7	1183099	1184053	+	SDT1	Putative suppressor of disruption of TFIIS
FUN_005460	contig_7	1184522	1185460	+		hypothetical protein
FUN_005462	contig_7	1193647	1196125	+		hypothetical protein
FUN_005463	contig_7	1196502	1200956	-	KK1G	ABC-type transporter kk1G

Gene ID	Scaffold	Start	Stop	Strand	Name	Product
FUN_005465	contig_7	1211074	1215070	+	RGA2_1	Rho-type gtpase-activating protein
FUN_005466	contig_7	1216067	1217548	-	IDH2	NAD-dependent isocitrate dehydrogenase
FUN_005467	contig_7	1226671	1227861	-	scn1	Cut9-interacting protein scn1
FUN_005468	contig_7	1228837	1229680	+		hypothetical protein
FUN_005470	contig_7	1238208	1238731	-	GPA2	Guanine nucleotide-binding protein alpha-2 subunit
FUN_005471	contig_7	1241262	1242662	+	HOG1	MAPK protein hog1
FUN_005472	contig_7	1243409	1245519	+		hypothetical protein
FUN_005474	contig_7	1250119	1251318	-		hypothetical protein
FUN_005475	contig_7	1251922	1254409	+	SSE1	adenyl-nucleotide exchange factor sse1
FUN_005476	contig_7	1255363	1258414	+		hypothetical protein
FUN_005478	contig_7	1261275	1262205	+		hypothetical protein
FUN_005479	contig_7	1262638	1263716	-		hypothetical protein
FUN_005480	contig_7	1264454	1266065	-		hypothetical protein
FUN_005481	contig_7	1268987	1272734	+	STU1	suppressor of tub2 mutation
FUN_005482	contig_7	1273681	1274118	+		hypothetical protein
FUN_005485	contig_7	1277087	1277974	-		hypothetical protein
FUN_005486	contig_7	1279125	1281727	+		hypothetical protein
FUN_005487	contig_7	1285685	1287294	+		hypothetical protein
FUN_005488	contig_7	1287930	1289927	-		hypothetical protein
FUN_005489	contig_7	1294296	1294715	+		hypothetical protein
FUN_005490	contig_7	1295648	1297360	+	faeB-1_2	Feruloyl esterase
FUN_005491	contig_7	1297934	1299707	+	ERG5	RNA polymerase C-22 sterol desaturase
FUN_005492	contig_7	1300926	1303687	+		hypothetical protein
FUN_005493	contig_7	1304809	1306985	-		hypothetical protein
FUN_005494	contig_7	1307632	1310289	-	EFT2	translation elongation factor 2
FUN_005496	contig_7	1313476	1315053	-		hypothetical protein
FUN_005497	contig_7	1315353	1316566	+		hypothetical protein
FUN_005499	contig_7	1320069	1325957	+	PMS1	ATP-binding mismatch repair protein
FUN_005500	contig_7	1326288	1329120	-	MCM3	MCM DNA helicase complex subunit
FUN_005501	contig_7	1329851	1331165	-		hypothetical protein
FUN_005502	contig_7	1332223	1333153	-		hypothetical protein
FUN_005503	contig_7	1334324	1337085	+		hypothetical protein
FUN_005504	contig_7	1338613	1340659	-	MTC6	Maintenance of telomere capping protein 6
FUN_005505	contig_7	1343855	1344804	-		hypothetical protein
FUN_005506	contig_7	1358893	1364027	-		hypothetical protein
FUN_005507	contig_7	1367250	1368515	+	SEC62	Translocation protein S62
FUN_005509	contig_7	1373336	1376083	+		hypothetical protein
FUN_005510	contig_7	1377899	1378679	+		hypothetical protein
FUN_005511	contig_7	1379893	1381043	+		hypothetical protein
FUN_005512	contig_7	1382945	1383799	+		hypothetical protein
FUN_005515	contig_7	1389786	1391951	-		hypothetical protein
FUN_005516	contig_7	1392266	1393784	+	CDS1	phosphatidate cytidylyltransferase
FUN_005517	contig_7	1394419	1396255	+		hypothetical protein
FUN_005518	contig_7	1396347	1398410	+	TEP1	Telomerase protein component 1
FUN_005519	contig_7	1399631	1403060	+		hypothetical protein
FUN_005520	contig_7	1405346	1407606	+		hypothetical protein
FUN_005522	contig_7	1411254	1412016	+		hypothetical protein
FUN_005523	contig_7	1412192	1413256	-		hypothetical protein
FUN_005525	contig_7	1417672	1420388	-		hypothetical protein
FUN_005526	contig_7	1421262	1422157	-		hypothetical protein
FUN_005527	contig_7	1422659	1425072	-		hypothetical protein
FUN_005528	contig_7	1426222	1430770	+		hypothetical protein
FUN_005530	contig_7	1435020	1435461	+		hypothetical protein
FUN_005531	contig_7	1436944	1437392	+		hypothetical protein
FUN_005532	contig_7	1438254	1439678	-		hypothetical protein
FUN_005533	contig_7	1441411	1443350	+		hypothetical protein
FUN_005534	contig_7	1444093	1446103	-		hypothetical protein
FUN_005535	contig_7	1446509	1447486	-	RDH1_2	short-chain alcohol dehydrogenase
FUN_005536	contig_7	1447977	1448889	+		hypothetical protein

Gene ID	Scaffold	Start	Stop	Strand	Name	Product
FUN_005537	contig_7	1451539	1452582	-		hypothetical protein
FUN_005538	contig_7	1453205	1453620	-		hypothetical protein
FUN_005539	contig_7	1455855	1457032	-		hypothetical protein
FUN_005544	contig_7	1474227	1478983	+		hypothetical protein
FUN_005546	contig_7	1484817	1487172	+		hypothetical protein
FUN_005547	contig_7	1487727	1489361	-		hypothetical protein
FUN_005548	contig_7	1489937	1492331	+		hypothetical protein
FUN_005549	contig_7	1492528	1493347	-		hypothetical protein
FUN_005550	contig_7	1494124	1495397	+		hypothetical protein
FUN_005551	contig_7	1495720	1498077	+	lsb6	Phosphatidylinositol 4-kinase LSB6
FUN_005552	contig_7	1498848	1502360	+		hypothetical protein
FUN_005553	contig_7	1506099	1507769	+		hypothetical protein
FUN_005554	contig_7	1509230	1511050	-		hypothetical protein
FUN_005555	contig_7	1513044	1514264	+		hypothetical protein
FUN_005557	contig_7	1516441	1519089	+		hypothetical protein
FUN_005562	contig_7	1526911	1530122	-		hypothetical protein
FUN_005563	contig_7	1530889	1532853	+	SRP68	signal recognition particle subunit srp68
FUN_005564	contig_7	1534393	1535415	+	GZF3	GATA zinc finger protein 3
FUN_005565	contig_7	1536096	1539689	-		hypothetical protein
FUN_005566	contig_7	1540787	1541308	+	atp9	ATP synthetase subunit 9
FUN_005568	contig_7	1545269	1547980	-		hypothetical protein
FUN_005569	contig_7	1554080	1554976	-		hypothetical protein
FUN_005570	contig_7	1555387	1557157	+	PRS1	ribose-phosphate pyrophosphokinase 1
FUN_005571	contig_7	1559959	1560981	-		hypothetical protein
FUN_005572	contig_7	1561681	1563386	+		hypothetical protein
FUN_005573	contig_7	1564057	1565338	-		hypothetical protein
FUN_005579	contig_7	1584065	1584958	+	saf4_2	Protein saf4
FUN_005580	contig_7	1586585	1587237	-		hypothetical protein
FUN_005581	contig_7	1588895	1589790	+		hypothetical protein
FUN_005582	contig_7	1590205	1590670	+		hypothetical protein
FUN_005585	contig_7	1594307	1595616	-	GYP8	GTPase-activating protein gyp8
FUN_005586	contig_7	1596698	1601073	+		hypothetical protein
FUN_005588	contig_7	1603154	1604182	-		hypothetical protein
FUN_005589	contig_7	1604737	1605290	+	srb7	Mediator of RNA polymerase II transcription subunit 21
FUN_005591	contig_7	1607420	1608522	-		hypothetical protein
FUN_005592	contig_7	1609642	1611235	+	TMS1	Membrane protein tms1
FUN_005597	contig_7	1621765	1623427	+		hypothetical protein
FUN_005598	contig_7	1624822	1626182	-		hypothetical protein
FUN_005599	contig_7	1626723	1627757	+		hypothetical protein
FUN_005605	contig_8	4462	5825	-		hypothetical protein
FUN_005606	contig_8	9248	11128	-		hypothetical protein
FUN_005607	contig_8	14717	15750	+		hypothetical protein
FUN_005608	contig_8	18460	19760	-		hypothetical protein
FUN_005609	contig_8	24788	27482	+		hypothetical protein
FUN_005610	contig_8	34991	36084	+		hypothetical protein
FUN_005611	contig_8	39140	39722	-		hypothetical protein
FUN_005612	contig_8	40155	41491	+		hypothetical protein
FUN_005617	contig_8	58070	59193	+	PPH3	phosphoprotein phosphatase PP4 catalytic subunit
FUN_005618	contig_8	59370	60484	-	REX4	3'-5' exonuclease
FUN_005619	contig_8	60769	62503	+	ESA1	Histone acetyltransferase
FUN_005621	contig_8	65536	68579	+		hypothetical protein
FUN_005622	contig_8	69578	71212	+		hypothetical protein
FUN_005623	contig_8	71648	76297	-		hypothetical protein
FUN_005624	contig_8	76652	77545	+	ARO7	chorismate mutase aro7
FUN_005625	contig_8	77916	79315	+	ALG3_2	dolichyl-P-Man:Man(5)GlcNAc(2)-PP-dolichol alpha-1,3-mannosyltransferase
FUN_005626	contig_8	79471	79785	-		hypothetical protein
FUN_005627	contig_8	80653	82345	+		hypothetical protein
FUN_005628	contig_8	83475	84971	+		hypothetical protein

Gene ID	Scaffold	Start	Stop	Strand	Name	Product
FUN_005630	contig_8	90830	92598	+		hypothetical protein
FUN_005631	contig_8	93000	97773	-		hypothetical protein
FUN_005632	contig_8	102716	104168	-		hypothetical protein
FUN_005633	contig_8	105363	105787	+		hypothetical protein
FUN_005634	contig_8	107495	109648	-	ESF1	pre-rRNA-processing protein esf1
FUN_005636	contig_8	114816	115991	-		hypothetical protein
FUN_005637	contig_8	118271	122726	+		hypothetical protein
FUN_005638	contig_8	124769	125767	-		hypothetical protein
FUN_005639	contig_8	127435	128329	-		hypothetical protein
FUN_005643	contig_8	138189	139025	+		hypothetical protein
FUN_005644	contig_8	147689	153511	+	IML1	vacuolar membrane-associated protein iml1
FUN_005645	contig_8	154307	157987	-		hypothetical protein
FUN_005646	contig_8	162246	164417	-		hypothetical protein
FUN_005647	contig_8	164814	166676	-		hypothetical protein
FUN_005648	contig_8	171121	175900	-	VPS15	Serine/threonine-protein kinase
FUN_005649	contig_8	176212	177686	+	ALG2	Alpha-1,3-mannosyltransferase-like protein
FUN_005650	contig_8	178969	179533	+		hypothetical protein
FUN_005651	contig_8	183409	184215	-	ras1	RAS1 protein
FUN_005652	contig_8	185707	186722	-		hypothetical protein
FUN_005653	contig_8	187143	188055	+		hypothetical protein
FUN_005654	contig_8	189425	191105	+		hypothetical protein
FUN_005655	contig_8	192144	194080	+		hypothetical protein
FUN_005658	contig_8	198196	199513	+	LEA1	U2 snRNP complex subunit
FUN_005659	contig_8	199875	201405	-	FIP1	Cleavage polyadenylation factor subunit fip1
FUN_005660	contig_8	201572	203168	+	NUP49	Nucleoporin nup49/NSP49 (Nuclear pore protein nup49/NSP49)
FUN_005661	contig_8	205884	206991	-	ECM4	S-glutathionyl-(chloro)hydroquinone reductase
FUN_005662	contig_8	207748	210856	-	RPN1	proteasome regulatory particle base subunit
FUN_005663	contig_8	211361	212879	+	rio2	Serine/threonine-protein kinase rio2
FUN_005664	contig_8	213738	215641	-		hypothetical protein
FUN_005665	contig_8	219093	221233	-		hypothetical protein
FUN_005666	contig_8	222683	230654	+	TAO3	Cell morphoproteinsis protein PAG1
FUN_005667	contig_8	231537	235012	+		hypothetical protein
FUN_005668	contig_8	239548	243351	+		hypothetical protein
FUN_005669	contig_8	243721	245291	-		hypothetical protein
FUN_005670	contig_8	245798	247637	-	NMD3	ribosome-binding protein
FUN_005671	contig_8	247927	249195	+	ATP25	ATPase synthesis protein 25 mitochondrial
FUN_005674	contig_8	257684	259603	+		hypothetical protein
FUN_005677	contig_8	271661	275219	+		hypothetical protein
FUN_005678	contig_8	275546	277021	-		hypothetical protein
FUN_005679	contig_8	277772	279641	-		hypothetical protein
FUN_005682	contig_8	289703	292362	-		hypothetical protein
FUN_005683	contig_8	293067	293701	+		hypothetical protein
FUN_005684	contig_8	294461	296248	+	LYS21	homocitrate synthase lys21
FUN_005685	contig_8	296896	297845	+	IDI1	isopentenyl-diphosphate delta-isomerase idi1
FUN_005686	contig_8	299600	300478	+		hypothetical protein
FUN_005688	contig_8	303441	305005	+	NUF2	kinetochore-associated Ndc80 complex subunit nuf2
FUN_005689	contig_8	305995	308323	+	CHT2_2	Chitinase 2
FUN_005690	contig_8	308624	309859	+	CHT2_3	Chitinase 2
FUN_005691	contig_8	311555	312223	+	MRPS12	37S ribosomal protein S12, mitochondrial
FUN_005693	contig_8	320116	320591	-	TRX1	thioredoxin trx1
FUN_005695	contig_8	329816	330409	-	SFU1	Suppressor of ferric uptake 1
FUN_005696	contig_8	334459	335634	-	GCN3	translation initiation factor eIF-2B subunit alpha
FUN_005697	contig_8	336352	342989	-		hypothetical protein
FUN_005698	contig_8	343809	345761	-		hypothetical protein
FUN_005699	contig_8	346722	347716	-		hypothetical protein
FUN_005700	contig_8	347979	349391	-	PEX12	ubiquitin-protein ligase peroxin 12
FUN_005701	contig_8	351448	353017	-		hypothetical protein
FUN_005704	contig_8	360355	362246	-		hypothetical protein

Gene ID	Scaffold	Start	Stop	Strand	Name	Product
FUN_005705	contig_8	363834	364489	+		hypothetical protein
FUN_005706	contig_8	365082	366223	+		hypothetical protein
FUN_005707	contig_8	367304	368086	+		hypothetical protein
FUN_005710	contig_8	373514	374363	+		hypothetical protein
FUN_005714	contig_8	380832	382092	+		hypothetical protein
FUN_005716	contig_8	387701	390559	-		hypothetical protein
FUN_005718	contig_8	394829	397927	+	CHO2	phosphatidylethanolamine N-methyltransferase
FUN_005719	contig_8	399201	402035	+		hypothetical protein
FUN_005720	contig_8	403389	404151	-		hypothetical protein
FUN_005727	contig_8	424372	426006	+		hypothetical protein
FUN_005729	contig_8	429046	433344	+	RPO41	DNA-directed RNA polymerase
FUN_005730	contig_8	436281	437519	-		hypothetical protein
FUN_005731	contig_8	438160	439866	+		hypothetical protein
FUN_005732	contig_8	440288	441401	+		hypothetical protein
FUN_005734	contig_8	452400	456238	-		hypothetical protein
FUN_005735	contig_8	456973	458901	+		hypothetical protein
FUN_005736	contig_8	459414	461594	-		hypothetical protein
FUN_005737	contig_8	461950	463568	+		hypothetical protein
FUN_005738	contig_8	463678	465385	-		hypothetical protein
FUN_005739	contig_8	466118	467312	+		hypothetical protein
FUN_005740	contig_8	470180	471448	-		hypothetical protein
FUN_005742	contig_8	478982	480021	-		hypothetical protein
FUN_005743	contig_8	481626	483044	+		hypothetical protein
FUN_005744	contig_8	485976	487583	+		hypothetical protein
FUN_005745	contig_8	487983	489474	-		hypothetical protein
FUN_005746	contig_8	496571	497455	+		hypothetical protein
FUN_005747	contig_8	497939	499781	-		hypothetical protein
FUN_005748	contig_8	500729	502516	-		hypothetical protein
FUN_005749	contig_8	504634	506919	-		hypothetical protein
FUN_005750	contig_8	507859	508601	+	BIO2_1	biotin synthase
FUN_005751	contig_8	508758	510038	-	BIO2_2	biotin synthase
FUN_005752	contig_8	510482	513047	+		hypothetical protein
FUN_005753	contig_8	513174	514073	-	SEN34	tRNA-splicing endonuclease subunit
FUN_005754	contig_8	514724	515908	-	ELP4	Elongator subunit elp4
FUN_005756	contig_8	519303	519645	+	RPB8	DNA-directed RNA polymerases I, II, and III subunit RPABC3
FUN_005757	contig_8	519895	521030	-		hypothetical protein
FUN_005758	contig_8	521331	522845	+	TRM12	S-adenosylmethionine-dependent methyltransferase
FUN_005760	contig_8	526436	529670	-	NUP84	Nucleoporin nup84
FUN_005761	contig_8	531230	531540	+	SMT3_2	SUMO protein smt3
FUN_005763	contig_8	533934	536212	+		hypothetical protein
FUN_005764	contig_8	536865	538273	-		hypothetical protein
FUN_005765	contig_8	539505	540316	+	RDI1	rho GDP dissociation inhibitor
FUN_005767	contig_8	541771	544325	+	APL6	AP-3 complex subunit beta
FUN_005769	contig_8	548730	549800	+	PPG1	Putative serine/threonine protein phosphatase
FUN_005770	contig_8	550170	551508	-	URK1	Uridine kinase
FUN_005771	contig_8	552092	553222	+		hypothetical protein
FUN_005772	contig_8	553625	556693	-	MCM6	MCM DNA helicase complex subunit mcm6
FUN_005773	contig_8	557335	558572	-	PPH1	Serine/threonine-protein phosphatase PP2A catalytic subunit
FUN_005774	contig_8	559036	560941	+		hypothetical protein
FUN_005775	contig_8	561479	562923	-		hypothetical protein
FUN_005777	contig_8	567158	569468	-	YND1	Golgi apyrase
FUN_005778	contig_8	570162	571014	-		hypothetical protein
FUN_005779	contig_8	572023	573362	+		hypothetical protein
FUN_005780	contig_8	578203	582471	+	PMC1_2	plasma membrane calcium
FUN_005781	contig_8	583297	583723	+	DAD1	Dolichyl-diphosphooligosaccharide-protein glycosyltransferase subunit dad1
FUN_005782	contig_8	584397	585365	-	rho4	RHO4 protein

Gene ID	Scaffold	Start	Stop	Strand	Name	Product
FUN_005783	contig_8	585789	587671	+	LCB3	Long-chain base-1-phosphate phosphatase
FUN_005784	contig_8	587925	588600	+		hypothetical protein
FUN_005785	contig_8	588883	591342	-		hypothetical protein
FUN_005786	contig_8	592559	594364	-		hypothetical protein
FUN_005787	contig_8	595454	596435	+	asa1	Astra associated protein 1 Asa1
FUN_005788	contig_8	596564	598656	-		hypothetical protein
FUN_005789	contig_8	599473	601955	-	YPO7	Fe-S oxidoreductase
FUN_005790	contig_8	602382	603384	-	FAD1	3'-phosphoadenosine 5'-phosphosulfate sulfotransferase
FUN_005791	contig_8	603973	608796	+	MET5	Sulfite reductase [NADPH] subunit beta
FUN_005792	contig_8	611394	613178	+		hypothetical protein
FUN_005793	contig_8	614493	618402	+		hypothetical protein
FUN_005794	contig_8	619164	621996	+		hypothetical protein
FUN_005796	contig_8	623955	625533	-	RAD18	E3 ubiquitin-protein ligase rad18
FUN_005797	contig_8	625936	627978	-		hypothetical protein
FUN_005798	contig_8	628803	629756	+	RKI1	ribose-5-phosphate isomerase rki1
FUN_005800	contig_8	631451	632897	-		hypothetical protein
FUN_005802	contig_8	637292	639680	-		hypothetical protein
FUN_005803	contig_8	640196	642839	+	CCA1	CCA tRNA nucleotidyltransferase, mitochondrial
FUN_005804	contig_8	642907	644118	-	DAO1	D-amino acid oxidase
FUN_005805	contig_8	644816	645888	+		hypothetical protein
FUN_005806	contig_8	646546	647099	-	ERG2	C-8 sterol isomerase
FUN_005807	contig_8	648740	650541	+		hypothetical protein
FUN_005812	contig_8	661848	662471	-	ATP17	ATP synthase f chain, mitochondrial precursor
FUN_005813	contig_8	662724	663501	+	NdufS4	ndufs4 NADH dehydrogenase Fe-S protein subunit
FUN_005814	contig_8	663983	664806	-		hypothetical protein
FUN_005817	contig_8	671717	672509	+		hypothetical protein
FUN_005818	contig_8	675378	676957	-		hypothetical protein
FUN_005819	contig_8	677198	680842	+		hypothetical protein
FUN_005821	contig_8	683366	686093	-	sec1	syntaxin binding protein 1
FUN_005822	contig_8	686481	688961	+	SUV3	RNA helicase
FUN_005823	contig_8	689591	690605	-		hypothetical protein
FUN_005824	contig_8	691239	692208	+		hypothetical protein
FUN_005825	contig_8	692658	694949	-		hypothetical protein
FUN_005826	contig_8	695978	696945	-		hypothetical protein
FUN_005827	contig_8	698956	700256	-		hypothetical protein
FUN_005828	contig_8	701762	704419	-	NPR1	Nitrogen permease reactivator protein
FUN_005829	contig_8	705491	706842	+	SVF1	Putative cell survival pathways protein
FUN_005830	contig_8	710117	712407	+	mlh1	DNA mismatch repair protein Mlh1
FUN_005831	contig_8	714901	718141	-		hypothetical protein
FUN_005832	contig_8	720382	721330	+		hypothetical protein
FUN_005833	contig_8	721359	722620	-	TAP42	Type 2A phosphatase-associated protein 42
FUN_005834	contig_8	723348	728172	+	dcl1	Dicer-like protein 1
FUN_005835	contig_8	728372	730230	-		hypothetical protein
FUN_005836	contig_8	730866	731744	+		hypothetical protein
FUN_005837	contig_8	732194	733188	-	VMA8	H(+)-transporting V1 sector ATPase subunit D
FUN_005838	contig_8	733704	736223	+	SYF1	pre-mRNA-splicing factor syf1
FUN_005840	contig_8	741715	743401	-	RHOBTB1	Rho-related BTB domain-containing protein 1
FUN_005843	contig_8	748286	748934	-	toa2	Transcription initiation factor IIA subunit 2
FUN_005844	contig_8	749236	750037	+	vma16	V-type proton ATPase subunit c''
FUN_005845	contig_8	750384	752660	+		hypothetical protein
FUN_005848	contig_8	757582	761092	-		hypothetical protein
FUN_005849	contig_8	761387	762971	+		hypothetical protein
FUN_005850	contig_8	763504	764770	+		hypothetical protein
FUN_005851	contig_8	765023	765676	+		hypothetical protein
FUN_005852	contig_8	766328	767800	+		hypothetical protein
FUN_005853	contig_8	768672	769469	+		hypothetical protein
FUN_005854	contig_8	769663	771401	-		hypothetical protein
FUN_005855	contig_8	774813	775988	-	ZTA1	NADPH:quinone reductase
FUN_005856	contig_8	776790	778547	-	GAS4	Glycolipid anchored surface protein 4 precursor

Gene ID	Scaffold	Start	Stop	Strand	Name	Product
FUN_005857	contig_8	779258	784272	-	GEA2	GDP/GTP exchange factor for ARF
FUN_005858	contig_8	785142	786289	-	ANP1	Mannan polymerase II complex anp1 subunit
FUN_005859	contig_8	788777	789838	+	MTR3	3'-5'-exoribonuclease
FUN_005861	contig_8	792883	795040	-	EXO84	exocyst complex component exo84
FUN_005862	contig_8	795365	795988	+	TOM20	mitochondrial import receptor subunit tom20
FUN_005863	contig_8	796449	797232	-	ryh1	GTPase Ryh1
FUN_005864	contig_8	797559	798944	+		hypothetical protein
FUN_005865	contig_8	799325	800808	+	CLP1	Cleavage polyadenylation factor subunit clp1
FUN_005866	contig_8	800979	802045	-		hypothetical protein
FUN_005867	contig_8	802527	804517	+		hypothetical protein
FUN_005868	contig_8	804937	805640	-		hypothetical protein
FUN_005870	contig_8	808651	809403	-	PDX3	pyridoxamine-phosphate oxidase
FUN_005872	contig_8	810738	811427	+		hypothetical protein
FUN_005873	contig_8	812039	813619	+		hypothetical protein
FUN_005874	contig_8	813856	814545	-		hypothetical protein
FUN_005875	contig_8	815039	816139	-		hypothetical protein
FUN_005876	contig_8	816638	818122	+		hypothetical protein
FUN_005877	contig_8	818281	819601	-		hypothetical protein
FUN_005879	contig_8	829072	830757	-		hypothetical protein
FUN_005881	contig_8	835398	836218	+		hypothetical protein
FUN_005883	contig_8	839740	840978	-		hypothetical protein
FUN_005884	contig_8	842741	844088	-	KRE2	alpha 1,2-mannosyltransferase 2.4.1
FUN_005885	contig_8	844778	847389	+	PAT1	DNA topoisomerase 2-associated protein pat1
FUN_005886	contig_8	849077	850399	+		hypothetical protein
FUN_005887	contig_8	852295	855975	-	PRP22	DEAH-box ATP-dependent RNA helicase prp22
FUN_005888	contig_8	857799	858960	-	ADH1	Alcohol dehydrogenase
FUN_005890	contig_8	862238	863927	-		hypothetical protein
FUN_005891	contig_8	864560	865496	+		hypothetical protein
FUN_005892	contig_8	865740	866573	-		hypothetical protein
FUN_005894	contig_8	869460	870430	-		hypothetical protein
FUN_005895	contig_8	870959	872226	+	SHE9	sensitivity to high expression protein she9
FUN_005896	contig_8	884424	887158	-		hypothetical protein
FUN_005897	contig_8	891608	893110	+		hypothetical protein
FUN_005900	contig_8	897844	899305	-	CEL1_1	Esterase/lipase/thioesterase
FUN_005901	contig_8	903525	905194	-		hypothetical protein
FUN_005902	contig_8	905623	906904	-		hypothetical protein
FUN_005903	contig_8	907081	908355	+	BUB3	mitotic spindle checkpoint protein Bub3
FUN_005905	contig_8	909895	910515	-	POP5	RNA-binding protein pop5
FUN_005906	contig_8	911272	911729	+		hypothetical protein
FUN_005907	contig_8	912506	918134	-	URA2	Carbamoyl-phosphate synthase
FUN_005908	contig_8	919431	922861	+		hypothetical protein
FUN_005909	contig_8	924272	925618	+		hypothetical protein
FUN_005910	contig_8	927124	930381	-		hypothetical protein
FUN_005911	contig_8	932194	932733	+		hypothetical protein
FUN_005912	contig_8	932946	933361	+		hypothetical protein
FUN_005913	contig_8	933737	934553	-	RPL6_1	60S ribosomal protein L6
FUN_005914	contig_8	934778	936410	+		hypothetical protein
FUN_005915	contig_8	938215	939899	+		hypothetical protein
FUN_005916	contig_8	944197	944932	+	XYL1_2	NAD(P)H-dependent D-xylose reductase (XR)
FUN_005917	contig_8	947267	948322	+		hypothetical protein
FUN_005918	contig_8	950223	952004	+	MEP1	low affinity high capacity ammonium permease
FUN_005920	contig_8	957991	959035	-		hypothetical protein
FUN_005921	contig_8	960209	962427	-		hypothetical protein
FUN_005922	contig_8	967415	968197	-		hypothetical protein
FUN_005923	contig_8	968879	970938	+	VPS62	Vacuolar protein sorting-associated protein 62
FUN_005925	contig_8	974195	976009	-		hypothetical protein
FUN_005926	contig_8	976823	979147	+		hypothetical protein
FUN_005927	contig_8	980994	981758	-		hypothetical protein
FUN_005929	contig_8	987391	989692	-		hypothetical protein

Gene ID	Scaffold	Start	Stop	Strand	Name	Product
FUN_005930	contig_8	989957	991653	-		hypothetical protein
FUN_005931	contig_8	992424	993367	-		hypothetical protein
FUN_005932	contig_8	994114	995333	-		hypothetical protein
FUN_005933	contig_8	995696	996391	+		hypothetical protein
FUN_005934	contig_8	996781	997627	+		hypothetical protein
FUN_005935	contig_8	997719	1005347	-		hypothetical protein
FUN_005936	contig_8	1005859	1008282	+	RAD3	TFIIH/NER complex ATP-dependent 5'-3' DNA helicase subunit
FUN_005937	contig_8	1008746	1009120	-		hypothetical protein
FUN_005938	contig_8	1010410	1011961	-	FPR3	peptidylprolyl isomerase fpr3
FUN_005939	contig_8	1012625	1016386	+	REV1	deoxycytidyl transferase
FUN_005940	contig_8	1016707	1017378	-		hypothetical protein
FUN_005941	contig_8	1018283	1020590	-		hypothetical protein
FUN_005942	contig_8	1024392	1026228	+		hypothetical protein
FUN_005943	contig_8	1026674	1028134	-		hypothetical protein
FUN_005944	contig_8	1030299	1037004	-		hypothetical protein
FUN_005945	contig_8	1038442	1040865	-		hypothetical protein
FUN_005946	contig_8	1041207	1043087	+		hypothetical protein
FUN_005947	contig_8	1043765	1044714	+		hypothetical protein
FUN_005948	contig_8	1045329	1046683	+	45748	aspartic proteinase precursor
FUN_005949	contig_8	1047496	1052084	+	ATG26	Sterol 3-beta-glucosyltransferase
FUN_005950	contig_8	1053038	1055572	+	fcp1	CTD phosphatase Fcp1
FUN_005952	contig_8	1059285	1061535	+		hypothetical protein
FUN_005953	contig_8	1061926	1062639	-	MRPL19	mitochondrial 54S ribosomal protein YmL19
FUN_005954	contig_8	1062999	1064135	-	RPN12_1	regulatory particle non-ATPase
FUN_005955	contig_8	1064514	1065989	-	RPN12_2	regulatory particle non-ATPase
FUN_005956	contig_8	1066378	1068169	-	LPD1	dihydrolipoamide dehydrogenase precursor
FUN_005957	contig_8	1068438	1070983	-		hypothetical protein
FUN_005958	contig_8	1071162	1071630	+		hypothetical protein
FUN_005959	contig_8	1071914	1072615	-		hypothetical protein
FUN_005960	contig_8	1073231	1074841	+		hypothetical protein
FUN_005961	contig_8	1077184	1080941	-		hypothetical protein
FUN_005962	contig_8	1081772	1083452	-		hypothetical protein
FUN_005963	contig_8	1083920	1084828	-		hypothetical protein
FUN_005964	contig_8	1085382	1087809	-	SNF5	SWI/SNF chromatin-remodeling complex subunit
FUN_005965	contig_8	1089050	1090390	-	VPS74	Vacuolar protein sorting-associated protein 74
FUN_005966	contig_8	1090887	1091561	-	ACP2	mitochondrial acyl carrier protein
FUN_005967	contig_8	1093213	1094894	-		hypothetical protein
FUN_005969	contig_8	1098536	1099996	+		hypothetical protein
FUN_005970	contig_8	1101553	1106779	+	LTE1	Guanine nucleotide exchange factor lte1
FUN_005971	contig_8	1108949	1111087	+		hypothetical protein
FUN_005972	contig_8	1111602	1114163	+		hypothetical protein
FUN_005973	contig_8	1114958	1116885	-		hypothetical protein
FUN_005974	contig_8	1117243	1118947	-		hypothetical protein
FUN_005976	contig_8	1122770	1126989	-		hypothetical protein
FUN_005977	contig_8	1127818	1129534	-		hypothetical protein
FUN_005978	contig_8	1130029	1133884	-	BUB1	protein kinase
FUN_005979	contig_8	1134566	1135828	-		hypothetical protein
FUN_005980	contig_8	1136535	1137570	+	PHB2	Prohibitin-2, subunit of the prohibitin complex (Phb1p-Phb2p)
FUN_005981	contig_8	1137827	1139485	-	PRI1	p48 polypeptide of DNA primase
FUN_005982	contig_8	1139583	1140774	+		hypothetical protein
FUN_005983	contig_8	1142799	1144441	+	DAL1	Allantoinase
FUN_005984	contig_8	1146856	1148223	+	ACE2	Metallothionein expression activator
FUN_005986	contig_8	1152481	1154165	+		hypothetical protein
FUN_005987	contig_8	1156327	1163037	-		hypothetical protein
FUN_005989	contig_8	1164781	1167890	+	SEC18	transport between ER and Golgi ATPase protein
FUN_005990	contig_8	1169889	1170427	+		hypothetical protein
FUN_005991	contig_8	1173560	1175371	+		hypothetical protein

Gene ID	Scaffold	Start	Stop	Strand	Name	Product
FUN_005992	contig_8	1175463	1176862	+		hypothetical protein
FUN_005993	contig_8	1177568	1178289	+	RPL19B	60S ribosomal protein L19B
FUN_005994	contig_8	1178923	1179975	-	SPT3	Transcription initiation protein spt3
FUN_005995	contig_8	1180841	1181818	+	DPH5	diphthine synthase
FUN_005998	contig_8	1186062	1186727	-		hypothetical protein
FUN_005999	contig_8	1187139	1189877	-		hypothetical protein
FUN_006000	contig_8	1192950	1193994	+		hypothetical protein
FUN_006001	contig_8	1194590	1197770	+	SEC24	COPII subunit
FUN_006002	contig_8	1201237	1201783	-	USV1	Up in starvation
FUN_006003	contig_8	1204310	1206641	-		hypothetical protein
FUN_006004	contig_8	1211069	1211710	-	RPL30	60S ribosomal protein L30
FUN_006005	contig_8	1212175	1214952	-	ATG9	autophagy protein atg9
FUN_006006	contig_8	1215374	1217519	+	COG6	Golgi transport complex subunit 6
FUN_006007	contig_8	1218336	1219592	+		hypothetical protein
FUN_006008	contig_8	1220378	1221382	+		hypothetical protein
FUN_006009	contig_8	1222193	1224047	-		hypothetical protein
FUN_006010	contig_8	1227571	1230169	+		hypothetical protein
FUN_006011	contig_8	1230879	1233880	-		hypothetical protein
FUN_006012	contig_8	1234152	1235700	-	ALG1	mannosyltransferase
FUN_006013	contig_8	1236147	1239015	+	MSC2	Putative zinc transporter msc2
FUN_006014	contig_8	1243074	1243652	-		hypothetical protein
FUN_006015	contig_8	1245314	1245829	+		hypothetical protein
FUN_006016	contig_8	1248175	1248521	+		hypothetical protein
FUN_006018	contig_8	1251095	1252534	-		hypothetical protein
FUN_006019	contig_8	1253167	1254050	-		hypothetical protein
FUN_006020	contig_8	1254286	1255983	+	RPN3	26S proteasome non-ATPase regulatory subunit
FUN_006021	contig_8	1256350	1257660	-		hypothetical protein
FUN_006022	contig_8	1258249	1260151	-	MCD1	sister chromatid cohesion protein 1
FUN_006023	contig_8	1261054	1264351	+	PMR1	High affinity Ca2+/Mn2+ P-type ATPase-like protein
FUN_006025	contig_8	1267867	1269894	+		hypothetical protein
FUN_006026	contig_8	1272716	1274333	-	CBS2	Cytochrome B translational activator protein cbs2
FUN_006028	contig_8	1278607	1279867	-	CNS1	HSP70/90 co-chaperone
FUN_006029	contig_8	1282958	1286990	+		hypothetical protein
FUN_006031	contig_8	1290059	1291216	-	PPM1	carboxy methyl transferase for protein phosphatase 2A
FUN_006032	contig_8	1291444	1292253	+	RPB5	DNA-directed RNA polymerases II 24 kDa polypeptide (RNA polymerase II subunit 5)
FUN_006033	contig_8	1292612	1293541	-		hypothetical protein
FUN_006034	contig_8	1294066	1295790	+		hypothetical protein
FUN_006035	contig_8	1295951	1298089	-	MAD1	coiled-coil domain-containing protein mad1
FUN_006036	contig_8	1304085	1306295	+		hypothetical protein
FUN_006037	contig_8	1310001	1311086	-		hypothetical protein
FUN_006038	contig_8	1311440	1313371	+	DBP3	RNA-dependent ATPase
FUN_006039	contig_8	1318118	1319382	+	tmk1	mitogen activated protein kinase
FUN_006040	contig_8	1323483	1323918	+		hypothetical protein
FUN_006041	contig_8	1326950	1328937	-		hypothetical protein
FUN_006042	contig_8	1330024	1337190	-	CHK1	Chk1 protein kinase
FUN_006043	contig_8	1337942	1340445	+		hypothetical protein
FUN_006044	contig_8	1341167	1342304	+	DAL2	Allantoicase
FUN_006045	contig_8	1342704	1343022	+		hypothetical protein
FUN_006047	contig_8	1345654	1346502	-		hypothetical protein
FUN_006048	contig_8	1347315	1350291	-		hypothetical protein
FUN_006049	contig_8	1351636	1352703	+		hypothetical protein
FUN_006050	contig_8	1353259	1354927	-		hypothetical protein
FUN_006051	contig_8	1355494	1358733	+		hypothetical protein
FUN_006052	contig_8	1358846	1359725	-		hypothetical protein
FUN_006053	contig_8	1360069	1361352	+	COQ1	coq1 putative hexaprenyl diphosphate synthase
FUN_006054	contig_8	1361648	1362916	-	SWD3	WD domain protein
FUN_006055	contig_8	1363094	1364481	+	ISC1	phospholipase C type enzyme
FUN_006056	contig_8	1365381	1369362	+	RAD54	DNA-dependent ATPase protein rad54

Gene ID	Scaffold	Start	Stop	Strand	Name	Product
FUN_006057	contig_8	1369763	1370578	-		hypothetical protein
FUN_006059	contig_8	1376135	1377895	-		hypothetical protein
FUN_006060	contig_8	1378345	1380095	+		hypothetical protein
FUN_006061	contig_8	1380586	1382381	-		hypothetical protein
FUN_006062	contig_8	1382736	1385537	+	MCM2	MCM DNA helicase complex subunit
FUN_006063	contig_8	1391295	1392644	+	PTH2	Gluconate transport-inducing protein
FUN_006064	contig_8	1393835	1395613	+	DAL5	Allantoate permease
FUN_006065	contig_8	1396724	1397405	-		hypothetical protein
FUN_006066	contig_8	1398989	1400245	-		hypothetical protein
FUN_006067	contig_8	1400807	1404484	+	ACR2	DNA-directed RNA polymerase I subunit RPA2
FUN_006068	contig_8	1404703	1405381	-		hypothetical protein
FUN_006069	contig_8	1405948	1407551	-	BFR1	multicopy suppressor of BFA (Brefeldin A)
FUN_006070	contig_8	1407825	1412272	+		hypothetical protein
FUN_006071	contig_8	1412535	1413206	-		hypothetical protein
FUN_006072	contig_8	1415746	1418127	-	gap1	RasGAP protein
FUN_006074	contig_8	1422406	1424433	-		hypothetical protein
FUN_006075	contig_8	1425198	1426426	-	CAR1	Arginase, catabolizes arginine to ornithine and urea
FUN_006076	contig_8	1427378	1427642	-		hypothetical protein
FUN_006077	contig_8	1429944	1434873	-		hypothetical protein
FUN_006078	contig_8	1435837	1436499	-	COQ9	Ubiquinone biosynthesis protein coq9, mitochondrial
FUN_006079	contig_8	1437251	1438015	+	cwf16_1	Pre-mRNA-splicing factor cwf16
FUN_006081	contig_8	1439678	1446285	-	UBR1	E3 ubiquitin-protein ligase ubr1
FUN_006082	contig_8	1447837	1448757	+		hypothetical protein
FUN_006084	contig_8	1450878	1454390	+		hypothetical protein
FUN_006085	contig_8	1455065	1459540	-	JHD1_2	JmjC domain-containing histone demethylation protein 1
FUN_006086	contig_8	1460159	1460673	+		hypothetical protein
FUN_006087	contig_8	1463405	1465009	+		hypothetical protein
FUN_006088	contig_8	1465041	1466123	-	PRE2	Proteasome subunit beta type-5
FUN_006089	contig_8	1467669	1469335	-		hypothetical protein
FUN_006090	contig_8	1469624	1471242	-	HHT1	histone H3.1
FUN_006091	contig_8	1471637	1472078	+	HHF1_2	Histone H4
FUN_006092	contig_8	1472654	1473990	+		hypothetical protein
FUN_006093	contig_8	1474342	1475089	-		hypothetical protein
FUN_006094	contig_8	1475989	1476803	-	ras2	RAS2 protein
FUN_006095	contig_8	1478087	1481065	+		hypothetical protein
FUN_006096	contig_9	827	8750	-		hypothetical protein
FUN_006097	contig_9	11381	12525	-		hypothetical protein
FUN_006099	contig_9	22345	25363	-		hypothetical protein
FUN_006100	contig_9	28339	29048	+	TAD2	tRNA(adenine34) deaminase
FUN_006101	contig_9	30197	32383	-		hypothetical protein
FUN_006104	contig_9	34973	35749	+		hypothetical protein
FUN_006105	contig_9	36152	37504	-		hypothetical protein
FUN_006106	contig_9	37938	39328	+		hypothetical protein
FUN_006107	contig_9	42002	42687	+	PFY1	profilin, required for normal timing of actin polymerization in response to thermal stress
FUN_006108	contig_9	43135	45168	-		hypothetical protein
FUN_006109	contig_9	45644	46702	+		hypothetical protein
FUN_006110	contig_9	48785	50098	-		hypothetical protein
FUN_006111	contig_9	52187	53555	-		hypothetical protein
FUN_006112	contig_9	54769	58066	-	NAN1	NET1-associated nuclear protein 1
FUN_006113	contig_9	59444	59699	+		hypothetical protein
FUN_006114	contig_9	61317	63146	-		hypothetical protein
FUN_006115	contig_9	64165	65020	-	CCS1	copper chaperone
FUN_006116	contig_9	65368	65939	+	MRPL23	54S ribosomal protein L23, mitochondrial
FUN_006117	contig_9	66530	67482	-		hypothetical protein
FUN_006118	contig_9	68382	72408	+		hypothetical protein
FUN_006119	contig_9	73661	75484	+		hypothetical protein
FUN_006120	contig_9	80091	81616	+		hypothetical protein
FUN_006121	contig_9	82295	83392	+	GRP2_2	Glycine-rich RNA-binding protein 2, mitochondrial

Gene ID	Scaffold	Start	Stop	Strand	Name	Product
FUN_006122	contig_9	84311	84657	+	NTF2	Nuclear transport factor 2
FUN_006123	contig_9	85234	86530	+		hypothetical protein
FUN_006124	contig_9	86691	88553	-		hypothetical protein
FUN_006125	contig_9	89219	90439	-	LET1	26S protease regulatory subunit 8
FUN_006126	contig_9	91305	94884	+		hypothetical protein
FUN_006127	contig_9	96159	97856	+		hypothetical protein
FUN_006128	contig_9	102068	103091	-		hypothetical protein
FUN_006130	contig_9	105507	106791	-		hypothetical protein
FUN_006131	contig_9	108189	110502	-		hypothetical protein
FUN_006132	contig_9	111199	114642	-	SEC4	GTP-binding protein
FUN_006133	contig_9	115190	117356	-		hypothetical protein
FUN_006134	contig_9	117923	119701	-		hypothetical protein
FUN_006135	contig_9	124595	125925	-		hypothetical protein
FUN_006136	contig_9	126683	130281	-	NIC96	nuclear pore complex subunit
FUN_006137	contig_9	131323	132314	-		hypothetical protein
FUN_006138	contig_9	132657	133655	-		hypothetical protein
FUN_006139	contig_9	134693	137596	-	SCH9	Serine/threonine-protein kinase
FUN_006140	contig_9	138988	139523	+		hypothetical protein
FUN_006141	contig_9	139982	140746	+		hypothetical protein
FUN_006142	contig_9	141321	142478	+		hypothetical protein
FUN_006144	contig_9	150411	151802	+	ERG13	3-hydroxy-3-methylglutaryl coenzyme A synthase
FUN_006145	contig_9	152362	152767	-	COX16	Cytochrome oxidase assembly
FUN_006146	contig_9	153210	161059	+	MEC1	serine/threonine-protein kinase M1
FUN_006148	contig_9	163694	165313	-		hypothetical protein
FUN_006149	contig_9	167100	169163	-		hypothetical protein
FUN_006150	contig_9	170212	171879	+	HER2	Trimeric GatFAB AmidoTransferase(AdT) complex subunit
FUN_006151	contig_9	173535	175322	-		hypothetical protein
FUN_006152	contig_9	176025	176474	+		hypothetical protein
FUN_006153	contig_9	177075	179363	+		hypothetical protein
FUN_006154	contig_9	185091	186445	+		hypothetical protein
FUN_006155	contig_9	187056	188612	+		hypothetical protein
FUN_006156	contig_9	188951	190398	+	ARP6	Actin-related protein 6
FUN_006157	contig_9	190596	192171	+		hypothetical protein
FUN_006158	contig_9	197492	198739	-		hypothetical protein
FUN_006160	contig_9	201902	202906	+	CWC26	Pre-mRNA-splicing factor cwc26
FUN_006163	contig_9	210984	213294	-	AFG2	AAA+-type ATPase
FUN_006165	contig_9	220688	221436	-		hypothetical protein
FUN_006166	contig_9	222644	223957	-		hypothetical protein
FUN_006167	contig_9	224663	225460	+	dot2	ESCRT II complex subunit Dot2
FUN_006168	contig_9	226509	227894	+		hypothetical protein
FUN_006169	contig_9	228335	231011	-	NIP1	Translation initiation factor 3 subunit c
FUN_006170	contig_9	232031	235029	-	YAK1	dual specificity protein kinase yak1
FUN_006171	contig_9	236410	238108	-	SIDA2	L-ornithine N(5)-monooxygenase
FUN_006172	contig_9	240310	256301	+		hypothetical protein
FUN_006173	contig_9	256856	258912	-		hypothetical protein
FUN_006175	contig_9	264719	266514	+		hypothetical protein
FUN_006177	contig_9	281051	282308	-		hypothetical protein
FUN_006178	contig_9	282787	284128	-		hypothetical protein
FUN_006179	contig_9	284769	286230	-	ecm33_2	cell wall protein Ecm33
FUN_006180	contig_9	286869	288963	-		hypothetical protein
FUN_006183	contig_9	294912	295546	+		hypothetical protein
FUN_006184	contig_9	296321	297269	+		hypothetical protein
FUN_006185	contig_9	298350	299852	+		hypothetical protein
FUN_006188	contig_9	317354	317889	+		hypothetical protein
FUN_006190	contig_9	321985	323129	-		hypothetical protein
FUN_006191	contig_9	323728	325605	-		hypothetical protein
FUN_006192	contig_9	326347	327306	+	CTP1	CtIP-related endonuclease
FUN_006193	contig_9	328252	328467	+		hypothetical protein

Gene ID	Scaffold	Start	Stop	Strand	Name	Product
FUN_006194	contig_9	328552	330014	+		hypothetical protein
FUN_006195	contig_9	330796	332112	-		hypothetical protein
FUN_006196	contig_9	332580	339027	-	FAS1_1	beta subunit of fatty acid synthetase
FUN_006197	contig_9	340163	345842	+	FAS1_2	beta subunit of fatty acid synthetase
FUN_006198	contig_9	347618	348969	+		hypothetical protein
FUN_006203	contig_9	362585	363983	-		hypothetical protein
FUN_006204	contig_9	364298	366521	-	ARP8	Actin-like protein arp8
FUN_006205	contig_9	368120	369515	-	DAL81_2	Fungal specific transcription factor
FUN_006206	contig_9	370812	372637	-		hypothetical protein
FUN_006207	contig_9	373295	375537	+		hypothetical protein
FUN_006208	contig_9	376317	379560	-		hypothetical protein
FUN_006209	contig_9	379858	381374	-	BCS1	Complex III assembly protein translocase and chaperone
FUN_006210	contig_9	381837	383436	+		hypothetical protein
FUN_006211	contig_9	384037	384495	+		hypothetical protein
FUN_006212	contig_9	384818	386025	+		hypothetical protein
FUN_006213	contig_9	386129	386533	-		hypothetical protein
FUN_006214	contig_9	386983	388511	+		hypothetical protein
FUN_006217	contig_9	394728	395566	-		hypothetical protein
FUN_006218	contig_9	395924	396701	+		hypothetical protein
FUN_006221	contig_9	408083	409987	-		hypothetical protein
FUN_006222	contig_9	412349	418011	-		hypothetical protein
FUN_006223	contig_9	421592	422891	-		hypothetical protein
FUN_006224	contig_9	423995	425051	+		hypothetical protein
FUN_006225	contig_9	425318	426497	-		hypothetical protein
FUN_006226	contig_9	427082	428985	-		hypothetical protein
FUN_006227	contig_9	429693	431455	-		hypothetical protein
FUN_006230	contig_9	437797	439610	-	LSC2	succinate--CoA ligase beta chain
FUN_006231	contig_9	439835	440636	+		hypothetical protein
FUN_006232	contig_9	440960	442886	-		hypothetical protein
FUN_006233	contig_9	443281	445035	+		hypothetical protein
FUN_006235	contig_9	448354	449872	-	NPL6	chromatin structure-remodeling complex subunit RSC7
FUN_006237	contig_9	452477	453052	+		hypothetical protein
FUN_006238	contig_9	457375	458447	+	HAC1	transcription factor that binds to CRE motif
FUN_006239	contig_9	461175	465417	-	VIP1	inositol hexakisphosphate and diphosphoinositol-pentakisphosphate kinase
FUN_006240	contig_9	466169	469427	+	UTP21	rRNA-processing protein utp21
FUN_006243	contig_9	482567	484330	-		hypothetical protein
FUN_006244	contig_9	484966	487283	+		hypothetical protein
FUN_006245	contig_9	487826	489163	-	HMT1	Nuclear SAM-dependent mono-and asymmetric methyltransferase
FUN_006247	contig_9	492823	493653	-	RIB5	Riboflavin synthase alpha chain
FUN_006248	contig_9	493961	495518	+		hypothetical protein
FUN_006249	contig_9	495797	497507	+		hypothetical protein
FUN_006250	contig_9	498456	501140	-	BRF1	transcription factor TFIIIB subunit brf1
FUN_006252	contig_9	505685	506915	+		hypothetical protein
FUN_006253	contig_9	507385	508463	+		hypothetical protein
FUN_006254	contig_9	509058	510521	+		hypothetical protein
FUN_006255	contig_9	510762	512196	-		hypothetical protein
FUN_006258	contig_9	521046	521925	+		hypothetical protein
FUN_006261	contig_9	526658	527567	-		hypothetical protein
FUN_006264	contig_9	534236	535339	-		hypothetical protein
FUN_006266	contig_9	540802	543771	-	vps35	retromer complex subunit Vps35
FUN_006268	contig_9	545466	547247	+		hypothetical protein
FUN_006269	contig_9	547645	548912	-		hypothetical protein
FUN_006270	contig_9	551349	556745	-		hypothetical protein
FUN_006271	contig_9	561749	562776	+		hypothetical protein
FUN_006272	contig_9	563417	565179	-		hypothetical protein
FUN_006273	contig_9	566228	567163	+	QA3	Quinate dehydrogenase
FUN_006274	contig_9	568152	568476	-		hypothetical protein

Gene ID	Scaffold	Start	Stop	Strand	Name	Product
FUN_006276	contig_9	571999	574347	-		hypothetical protein
FUN_006278	contig_9	579312	580389	+	CYC3	holocytochrome c synthase
FUN_006279	contig_9	580985	582428	-		hypothetical protein
FUN_006280	contig_9	583328	585900	-		hypothetical protein
FUN_006281	contig_9	586501	588149	+	GDT1	GCR1-dependent translation factor 1
FUN_006282	contig_9	588555	589733	-		hypothetical protein
FUN_006283	contig_9	590266	591996	+		hypothetical protein
FUN_006284	contig_9	592212	593687	+		hypothetical protein
FUN_006285	contig_9	595862	597567	+	PFK1	6-phosphofructokinase, alpha subunit
FUN_006286	contig_9	597859	599097	+	ETR1	mitochondrial 2-enoyl thioester reductase
FUN_006287	contig_9	599532	601021	-		hypothetical protein
FUN_006288	contig_9	601564	603546	-	SRB4	RNA polymerase II mediator complex subunit
FUN_006289	contig_9	604361	605682	+	ISN1	IMP 5'-nucleotidase
FUN_006291	contig_9	607654	609968	+	NDC80	kinetochore-associated Ndc80 complex subunit ndc80
FUN_006292	contig_9	610517	612304	-		hypothetical protein
FUN_006294	contig_9	615664	617733	+		hypothetical protein
FUN_006295	contig_9	621315	623373	+		hypothetical protein
FUN_006296	contig_9	623462	624307	-		hypothetical protein
FUN_006297	contig_9	625652	626494	-	RPC31	DNA-directed RNA polymerase III subunit C31
FUN_006299	contig_9	632389	633512	+		hypothetical protein
FUN_006301	contig_9	638029	638957	+	ERG25	C-4 sterol methyl oxidase
FUN_006302	contig_9	644059	644888	+		hypothetical protein
FUN_006303	contig_9	647403	648525	-		hypothetical protein
FUN_006304	contig_9	649886	657169	-	TOR1	phosphatidylinositol kinase-related protein kinase tor1
FUN_006306	contig_9	660149	662015	+		hypothetical protein
FUN_006308	contig_9	664223	668617	-		hypothetical protein
FUN_006309	contig_9	679912	682130	-		hypothetical protein
FUN_006310	contig_9	682591	683420	+	CSM3	chromosome segregation in meiosis-related protein
FUN_006311	contig_9	683891	687991	+	SKI2	Antiviral helicase ski2
FUN_006312	contig_9	689011	692399	+	NMD5	Nonsense-mediated mRNA decay protein 5
FUN_006313	contig_9	692998	693977	-		hypothetical protein
FUN_006314	contig_9	694375	695210	+	RER1	retention in endoplasmic reticulum protein 1
FUN_006315	contig_9	695744	696956	-		hypothetical protein
FUN_006317	contig_9	698266	699746	+	RPT5	26S proteasome regulatory subunit 6A
FUN_006318	contig_9	701329	703282	+		hypothetical protein
FUN_006319	contig_9	704704	709283	+		hypothetical protein
FUN_006320	contig_9	709591	711093	-	TFB2	RNA polymerase II transcription factor B 52 kDa subunit
FUN_006321	contig_9	711784	712124	+		hypothetical protein
FUN_006323	contig_9	715150	718803	-	TOF1	Topoisomerase 1-associated factor 1
FUN_006324	contig_9	719125	720180	+		hypothetical protein
FUN_006328	contig_9	726196	726597	+		hypothetical protein
FUN_006329	contig_9	727042	727984	-		hypothetical protein
FUN_006332	contig_9	731608	733120	+	zrg17	cation diffusion zinc membrane transporter Zrg17
FUN_006333	contig_9	733709	735862	+		hypothetical protein
FUN_006336	contig_9	738778	740083	+		hypothetical protein
FUN_006338	contig_9	742665	743897	+		hypothetical protein
FUN_006341	contig_9	748394	749035	-		hypothetical protein
FUN_006342	contig_9	750366	753078	+		hypothetical protein
FUN_006343	contig_9	753460	754203	-	ARL1	Arf GTPase arl1
FUN_006345	contig_9	757660	758235	-		hypothetical protein
FUN_006347	contig_9	762932	764229	+		hypothetical protein
FUN_006350	contig_9	768661	770616	-		hypothetical protein
FUN_006351	contig_9	771543	773044	-		hypothetical protein
FUN_006352	contig_9	773598	774215	-	rex2	Phosphatidylinositol 3,4,5-trisphosphate-dependent Rac exchanger 2 protein
FUN_006353	contig_9	774482	779046	+	POL1	DNA-directed DNA polymerase alpha catalytic subunit pol1
FUN_006354	contig_9	779247	780745	-	BNA5	Kynureninase (L-kynurenine hydrolase)
FUN_006355	contig_9	781004	781627	+	ISA2	[4Fe-4S] proteins maturation

Gene ID	Scaffold	Start	Stop	Strand	Name	Product
FUN_006356	contig_9	782585	783781	+		hypothetical protein
FUN_006359	contig_9	789035	792568	-		hypothetical protein
FUN_006361	contig_9	796762	802799	-	HIR3	Histone transcription regulator 3
FUN_006362	contig_9	803191	805559	+	BUD7	bud site selection protein
FUN_006363	contig_9	805999	806624	-	RPS16	40S ribosomal protein S16
FUN_006364	contig_9	807195	808513	+	ATS1	alpha tubulin suppressor
FUN_006365	contig_9	808875	809856	-		hypothetical protein
FUN_006367	contig_9	814296	814745	+	DBP2_1	ATP-dependent RNA helicase dbp2
FUN_006368	contig_9	815465	818063	+	DBP2_2	ATP-dependent RNA helicase dbp2
FUN_006369	contig_9	826998	828513	+		hypothetical protein
FUN_006370	contig_9	831896	833854	+		hypothetical protein
FUN_006371	contig_9	834829	836524	+		hypothetical protein
FUN_006372	contig_9	838674	840562	+		hypothetical protein
FUN_006373	contig_9	843905	844847	+		hypothetical protein
FUN_006374	contig_9	845051	846055	-	rtf2	Replication termination factor 2
FUN_006375	contig_9	846611	850107	-		hypothetical protein
FUN_006376	contig_9	851912	852396	-	ATP14	ATP synthase F0 subcomplex subunit H atp14
FUN_006377	contig_9	852994	854091	+	SUA7_1	transcription initiation factor IIB
FUN_006381	contig_9	861855	863109	-	GLD2	Glycerol 2-dehydrogenase (NADP(+))
FUN_006382	contig_9	864273	865772	+		hypothetical protein
FUN_006383	contig_9	867823	870855	+	DEF1	RNAPII degradation factor
FUN_006385	contig_9	874350	876331	-	PRP4	U4/U6 small nuclear ribonucleoprotein prp4
FUN_006386	contig_9	882044	883871	+		hypothetical protein
FUN_006387	contig_9	887610	889831	-		hypothetical protein
FUN_006388	contig_9	891458	892658	+	HGH1	Protein hgh1
FUN_006389	contig_9	892756	894395	-		hypothetical protein
FUN_006390	contig_9	895586	897725	-	TFG1	transcription factor IIF subunit tfg1
FUN_006391	contig_9	898372	900600	+		hypothetical protein
FUN_006392	contig_9	900977	902671	-	HGT1_2	high affinity glucose transporter
FUN_006393	contig_9	906879	907721	-	UBC6	Ubiquitin-conjugating enzyme E2 6
FUN_006394	contig_9	908071	909755	+		hypothetical protein
FUN_006396	contig_9	916819	917258	+	DAP1	Dihydrodipicolinate synthase
FUN_006398	contig_9	920702	921813	+		hypothetical protein
FUN_006399	contig_9	921885	922811	-		hypothetical protein
FUN_006402	contig_9	929595	932561	-		hypothetical protein
FUN_006403	contig_9	937080	938987	-	PCM1	Phosphoacetylglucosamine Mutase
FUN_006404	contig_9	940176	940725	+		hypothetical protein
FUN_006405	contig_9	941268	941921	-	RHO2	Rho GTPase
FUN_006406	contig_9	943327	947080	+	NST1	Stress response protein nst1
FUN_006407	contig_9	947914	952662	+		hypothetical protein
FUN_006408	contig_9	952867	953815	-		hypothetical protein
FUN_006409	contig_9	954792	956576	+		hypothetical protein
FUN_006410	contig_9	957402	959633	+	BPL1	biotin holocarboxylase synthetase
FUN_006411	contig_9	959940	961559	-	kes1	Oxysterol-binding protein 4
FUN_006412	contig_9	962083	964119	-		hypothetical protein
FUN_006413	contig_9	964736	966659	-	PGM2	Phosphoglucomutase-2
FUN_006414	contig_9	967694	969079	+		hypothetical protein
FUN_006416	contig_9	970664	971950	+		hypothetical protein
FUN_006417	contig_9	975144	976205	-		hypothetical protein
FUN_006418	contig_9	976842	978011	+	HOM6	Homoserine dehydrogenase
FUN_006419	contig_9	978113	979306	-		hypothetical protein
FUN_006421	contig_9	982423	983395	+		hypothetical protein
FUN_006423	contig_9	989432	993567	-	ATG11	oligomeric, coiled-coil, peripheral membrane protein
FUN_006424	contig_9	993769	994607	+		hypothetical protein
FUN_006425	contig_9	996728	997211	+	NHP6	Non-histone chromosomal protein 6
FUN_006426	contig_9	998545	999135	-	SEC11	Signal peptidase complex catalytic subunit
FUN_006428	contig_9	1004583	1005946	+		hypothetical protein
FUN_006430	contig_9	1008693	1010801	+		hypothetical protein
FUN_006432	contig_9	1021378	1024041	-		hypothetical protein

Gene ID	Scaffold	Start	Stop	Strand	Name	Product
FUN_006433	contig_9	1025641	1026213	-		hypothetical protein
FUN_006434	contig_9	1027845	1028687	+		hypothetical protein
FUN_006435	contig_9	1035108	1036736	+		hypothetical protein
FUN_006436	contig_9	1037266	1038278	+		hypothetical protein
FUN_006437	contig_9	1038466	1039431	-		hypothetical protein
FUN_006438	contig_9	1039807	1040423	+		hypothetical protein
FUN_006439	contig_9	1042185	1044798	+		hypothetical protein
FUN_006441	contig_9	1049342	1053079	-	rhp26	DNA repair protein rhp26
FUN_006442	contig_9	1054710	1056442	+		hypothetical protein
FUN_006443	contig_9	1056662	1057268	-		hypothetical protein
FUN_006445	contig_9	1062202	1063469	-	PEX14	peroxisomal membrane protein pex14
FUN_006448	contig_9	1065538	1067188	-		hypothetical protein
FUN_006449	contig_9	1067748	1069003	-		hypothetical protein
FUN_006450	contig_9	1070135	1071434	+		hypothetical protein
FUN_006451	contig_9	1071629	1073064	-	zuo1	Zuotin
FUN_006452	contig_9	1073334	1077026	+	IMH1	Golgin imh1
FUN_006453	contig_9	1078220	1080364	+	SKY1	serine/threonine protein kinase, CMGC
FUN_006454	contig_9	1081699	1084833	+		hypothetical protein
FUN_006455	contig_9	1085707	1087473	-		hypothetical protein
FUN_006456	contig_9	1088507	1090083	-	ATF1_2	Alcohol acetyltransferase
FUN_006458	contig_9	1092410	1093971	+		hypothetical protein
FUN_006459	contig_9	1095653	1103082	+		hypothetical protein
FUN_006460	contig_9	1103566	1106490	-		hypothetical protein
FUN_006461	contig_9	1108029	1109277	-		hypothetical protein
FUN_006462	contig_9	1110774	1112171	+		hypothetical protein
FUN_006463	contig_9	1113291	1115945	+	SIP5	SNF1-interacting protein
FUN_006465	contig_9	1124828	1126121	+	AIM6_1	Altered inheritance of mitochondria protein 6
FUN_006466	contig_9	1126470	1127322	+		hypothetical protein
FUN_006468	contig_9	1128698	1130601	-		hypothetical protein
FUN_006469	contig_9	1133829	1135570	+		hypothetical protein
FUN_006471	contig_9	1138312	1138854	+		hypothetical protein
FUN_006472	contig_9	1139750	1142008	+		hypothetical protein
FUN_006474	contig_9	1144607	1147597	+		hypothetical protein
FUN_006476	contig_9	1150372	1151421	-		hypothetical protein
FUN_006478	contig_9	1154067	1154669	+		hypothetical protein
FUN_006479	contig_9	1156099	1157997	+		hypothetical protein
FUN_006480	contig_9	1163931	1165312	-	SKS1	Serine/threonine protein kinase
FUN_006481	contig_9	1172949	1174397	+		hypothetical protein
FUN_006483	contig_9	1176131	1179278	-	HOF1	formin-binding protein
FUN_006484	contig_9	1180238	1187246	+	CDC39	CCR4-NOT core subunit cdc39
FUN_006486	contig_9	1198129	1199882	-		hypothetical protein
FUN_006488	contig_9	1203873	1205095	+		hypothetical protein
FUN_006489	contig_9	1205412	1206579	+		hypothetical protein
FUN_006491	contig_9	1208176	1209972	-		hypothetical protein
FUN_006492	contig_9	1210331	1211257	+		hypothetical protein
FUN_006493	contig_9	1212037	1213165	+	FBA1	Fructose-bisphosphate aldolase 1
FUN_006495	contig_9	1216016	1217514	-		hypothetical protein
FUN_006496	contig_9	1218109	1220304	-	ARP9	Actin-like protein arp9
FUN_006497	contig_9	1220625	1225222	+	NTE1	phosphatidylcholine and lysophosphatidylcholine phospholipase
FUN_006498	contig_9	1225559	1228639	-	ATH1	alpha,alpha-trehalase ath1
FUN_006499	contig_9	1229761	1232725	-	GCV2	glycine decarboxylase subunit P
FUN_006501	contig_9	1237061	1238994	+		hypothetical protein
FUN_006502	contig_9	1239903	1240993	+		hypothetical protein
FUN_006503	contig_9	1241785	1243029	+		hypothetical protein
FUN_006504	contig_9	1243262	1246096	-		hypothetical protein
FUN_006505	contig_9	1246580	1247638	-		hypothetical protein
FUN_006508	contig_9	1250293	1251868	-		hypothetical protein
FUN_006509	contig_9	1253064	1255286	-	TRM1	RNA methyltransferase tRNA(m5U54)methyltransferase

Gene ID	Scaffold	Start	Stop	Strand	Name	Product
FUN_006512	contig_9	1266671	1268287	-		hypothetical protein
FUN_006513	contig_9	1268787	1269788	-		hypothetical protein
FUN_006516	contig_9	1273032	1274599	+		hypothetical protein
FUN_006517	contig_9	1276130	1278342	+		hypothetical protein
FUN_006520	contig_9	1282535	1284853	+		hypothetical protein
FUN_006521	contig_9	1285650	1286876	+		hypothetical protein
FUN_006522	contig_9	1288907	1290672	-		hypothetical protein
FUN_006523	contig_9	1291396	1292828	-		hypothetical protein
FUN_006524	contig_9	1293206	1293955	-		hypothetical protein
FUN_006525	contig_9	1294072	1294437	-		hypothetical protein
FUN_006526	contig_9	1295435	1296958	+		hypothetical protein
FUN_006529	contig_9	1300802	1302267	-		hypothetical protein
FUN_006530	contig_9	1303756	1306992	-		hypothetical protein
FUN_006532	contig_9	1309779	1310651	+	rpl13	60S ribosomal protein L13
FUN_006533	contig_9	1311158	1312538	-		hypothetical protein
FUN_006535	contig_9	1314517	1315747	-		hypothetical protein
FUN_006536	contig_9	1316534	1317679	-	SLT11	Pre-mRNA-splicing factor slt11
FUN_006537	contig_9	1318110	1319406	+	ERG6_2	Delta(24)-sterol C-methyltransferase
FUN_006538	contig_9	1320104	1321393	+	PDB1	pyruvate dehydrogenase E1, beta subunit
FUN_006540	contig_9	1326127	1327401	+	CSH1_1	CSG1/SUR1-like protein
FUN_006541	contig_9	1328132	1330003	+		hypothetical protein
FUN_006542	contig_9	1330876	1332632	-	RRP3	ribosomal RNA processing protein
FUN_006543	contig_9	1332907	1334307	+	SSF1	rRNA-binding ribosome biosynthesis protein
FUN_006545	contig_9	1335950	1336929	+		hypothetical protein
FUN_006547	contig_9	1341693	1343174	-		hypothetical protein
FUN_006548	contig_9	1343968	1344989	-		hypothetical protein
FUN_006549	contig_9	1348599	1349536	+		hypothetical protein
FUN_006550	contig_9	1349947	1351990	+		hypothetical protein
FUN_006551	contig_9	1352778	1354151	+		hypothetical protein
FUN_006552	contig_9	1354447	1355958	-		hypothetical protein
FUN_006553	contig_9	1357039	1358741	+		hypothetical protein
FUN_006554	contig_9	1358983	1360916	-		hypothetical protein
FUN_006555	contig_9	1363989	1364818	+	tbp1	TATA-binding protein (TBP)
FUN_006556	contig_9	1365383	1366727	-		hypothetical protein
FUN_006557	contig_9	1367190	1368269	+		hypothetical protein
FUN_006559	contig_9	1372704	1374345	+		hypothetical protein
FUN_006561	contig_9	1379058	1385994	-	ILV2	Acetolactate synthase, mitochondrial
FUN_006562	contig_9	1386303	1387768	+		hypothetical protein
FUN_006563	contig_9	1389202	1389915	-		hypothetical protein
FUN_006565	contig_9	1394049	1395927	-		hypothetical protein
FUN_006567	contig_9	1397690	1399262	-		hypothetical protein
FUN_006568	contig_9	1400053	1400865	-	DPM1	dolichol-P-mannose synthesis
FUN_006569	contig_9	1401022	1402000	+		hypothetical protein
FUN_006570	contig_9	1402339	1405503	-		hypothetical protein
FUN_006572	contig_9	1409178	1410837	-	GAL1	galactokinase
FUN_006573	contig_9	1411339	1412075	-		hypothetical protein
FUN_006575	contig_9	1415069	1416586	-		hypothetical protein
FUN_006576	contig_9	1416980	1419345	-		hypothetical protein
FUN_006577	contig_9	1419718	1421019	+		hypothetical protein
FUN_006579	contig_9	1422705	1423663	+		hypothetical protein
FUN_006581	contig_9	1424687	1426581	-		hypothetical protein
FUN_006582	contig_9	1427183	1428943	+		hypothetical protein
FUN_006583	contig_9	1429281	1430631	-		hypothetical protein
FUN_006586	contig_9	1437328	1439002	-		hypothetical protein
FUN_006588	contig_9	1442560	1443717	+	GRE2_2	methylglyoxal reductase (NADPH-dependent) gre2
FUN_006590	contig_9	1449127	1450008	+		hypothetical protein
FUN_006593	contig_9	1454432	1455265	+		hypothetical protein
FUN_006597	contig_9	1464156	1465064	+		hypothetical protein
FUN_006598	contig_9	1466117	1467750	-		hypothetical protein

Gene ID	Scaffold	Start	Stop	Strand	Name	Product
FUN_006601	contig_9	1475142	1475336	-		hypothetical protein
FUN_006602	contig_9	1475601	1480632	-	UGA2_1	succinate semialdehyde dehydrogenase NADP+ linked
FUN_006603	contig_9	1481560	1482837	+		hypothetical protein
FUN_006604	contig_9	1483401	1485479	+		hypothetical protein
FUN_006605	contig_9	1487196	1488565	-		hypothetical protein
FUN_006606	contig_9	1489793	1490902	+		hypothetical protein
FUN_006607	contig_9	1491334	1491940	-		hypothetical protein
FUN_006608	contig_9	1492712	1493660	+		hypothetical protein
FUN_006609	contig_9	1494261	1495826	-		hypothetical protein
FUN_006610	contig_9	1499244	1506771	+		hypothetical protein
FUN_006611	contig_10	1576	2042	+		hypothetical protein
FUN_006613	contig_10	4302	6398	+		hypothetical protein
FUN_006615	contig_10	9548	11040	-		hypothetical protein
FUN_006616	contig_10	13986	15582	+		hypothetical protein
FUN_006617	contig_10	21351	24672	-		hypothetical protein
FUN_006618	contig_10	25754	27547	-		hypothetical protein
FUN_006619	contig_10	30770	31875	+		hypothetical protein
FUN_006620	contig_10	32400	33881	-		hypothetical protein
FUN_006622	contig_10	36349	39120	-		hypothetical protein
FUN_006623	contig_10	40648	43683	+		hypothetical protein
FUN_006624	contig_10	43912	45785	-		hypothetical protein
FUN_006625	contig_10	50033	51272	+	GLN1	glutamate--ammonia ligase
FUN_006626	contig_10	52095	54158	-		hypothetical protein
FUN_006628	contig_10	55854	56300	+		hypothetical protein
FUN_006629	contig_10	56686	57000	-		hypothetical protein
FUN_006631	contig_10	62915	64114	-		hypothetical protein
FUN_006632	contig_10	67527	71386	+		hypothetical protein
FUN_006633	contig_10	72643	74634	+		hypothetical protein
FUN_006634	contig_10	75307	78963	+	ISW2	chromatin remodeling complex Adenosinetriphosphatase
FUN_006635	contig_10	79770	81557	+		hypothetical protein
FUN_006637	contig_10	83501	84403	+		hypothetical protein
FUN_006638	contig_10	84563	85601	-		hypothetical protein
FUN_006639	contig_10	87152	88285	-	GET3	Golgi to ER traffic-related protein
FUN_006641	contig_10	91072	91866	+		hypothetical protein
FUN_006642	contig_10	92359	93237	+	PIN3	protein that induces appearance of [PIN+] prion when overproduced
FUN_006643	contig_10	93635	94879	+		hypothetical protein
FUN_006646	contig_10	101296	102616	-		hypothetical protein
FUN_006647	contig_10	103240	104349	+		hypothetical protein
FUN_006648	contig_10	105833	108786	+		hypothetical protein
FUN_006649	contig_10	110328	112251	+		hypothetical protein
FUN_006650	contig_10	112377	113595	-		hypothetical protein
FUN_006651	contig_10	114664	116594	-		hypothetical protein
FUN_006653	contig_10	121352	122215	-		hypothetical protein
FUN_006654	contig_10	122685	125706	-	USO1	Vesicle-mediated ER to Golgi transport protein
FUN_006655	contig_10	126192	129270	+	RAT1	5'-3' exoribonuclease 2
FUN_006656	contig_10	130493	132474	+		hypothetical protein
FUN_006658	contig_10	134084	135447	-		hypothetical protein
FUN_006659	contig_10	137134	138492	+		hypothetical protein
FUN_006660	contig_10	139705	140383	+		hypothetical protein
FUN_006662	contig_10	146621	147190	+		hypothetical protein
FUN_006663	contig_10	147982	149192	+		hypothetical protein
FUN_006666	contig_10	156413	158384	-		hypothetical protein
FUN_006667	contig_10	159785	161152	-	MRF1	Peptide chain release factor 1, mitochondrial
FUN_006668	contig_10	161630	163540	+		hypothetical protein
FUN_006669	contig_10	164900	166486	+		hypothetical protein
FUN_006670	contig_10	171285	172910	-		hypothetical protein
FUN_006671	contig_10	173290	174766	+		hypothetical protein
FUN_006672	contig_10	175710	178883	+		hypothetical protein

Gene ID	Scaffold	Start	Stop	Strand	Name	Product
FUN_006673	contig_10	179614	181182	+		hypothetical protein
FUN_006676	contig_10	189014	190256	-	cpc2	cross-pathway control WD-repeat protein cpc2
FUN_006677	contig_10	190817	192010	+		hypothetical protein
FUN_006678	contig_10	192303	192995	-	YHC1	U1 small nuclear ribonucleoprotein C
FUN_006679	contig_10	193245	195310	-	SCP1	calponin
FUN_006680	contig_10	195652	196522	+		hypothetical protein
FUN_006681	contig_10	197408	198744	+		hypothetical protein
FUN_006682	contig_10	199445	201582	+		hypothetical protein
FUN_006684	contig_10	204034	204973	+		hypothetical protein
FUN_006687	contig_10	210476	211592	+		hypothetical protein
FUN_006688	contig_10	211677	213332	-	ICP55	aminopeptidase
FUN_006689	contig_10	213592	214599	+		hypothetical protein
FUN_006690	contig_10	215003	216685	-		hypothetical protein
FUN_006691	contig_10	218364	220115	-	YAP1	DNA-binding transcription factor yap1
FUN_006692	contig_10	221595	222558	+		hypothetical protein
FUN_006693	contig_10	223156	226838	+	PAN2	poly(A)-specific ribonuclease
FUN_006694	contig_10	228138	229520	+		hypothetical protein
FUN_006695	contig_10	230187	231654	+		hypothetical protein
FUN_006696	contig_10	231757	232458	-		hypothetical protein
FUN_006697	contig_10	233098	236041	-	CHS3_2	Chitin synthase, class 3
FUN_006699	contig_10	238898	240016	-		hypothetical protein
FUN_006701	contig_10	241823	243210	-	ILV5	Bifunctional acetohydroxyacid reductoisomerase
FUN_006702	contig_10	243666	244340	-	UBC12	NEDD8-conjugating protein ubc12
FUN_006703	contig_10	244869	246077	+	RIB7	2,5-diamino-6-(ribosylamino)-4(3H)-pyrimidinone 5'-phosphate reductase
FUN_006704	contig_10	247420	248163	-	ERV25	vesicle coat component
FUN_006705	contig_10	248672	249703	-		hypothetical protein
FUN_006706	contig_10	254026	258049	-	RAV1	regulator of (H+)-ATPase in vacuolar membrane
FUN_006707	contig_10	258592	260314	-	rmt3	Ribosomal protein arginine N-methyltransferase rmt3
FUN_006710	contig_10	264541	265366	+		hypothetical protein
FUN_006711	contig_10	266094	268137	-		hypothetical protein
FUN_006715	contig_10	281660	283830	-		hypothetical protein
FUN_006716	contig_10	284916	288203	-		hypothetical protein
FUN_006717	contig_10	289338	289677	+	SLY1_1	Vesicle trafficking between the ER and Golgi
FUN_006718	contig_10	289999	292034	+	SLY1_2	Vesicle trafficking between the ER and Golgi
FUN_006719	contig_10	294130	296698	-		hypothetical protein
FUN_006720	contig_10	297481	299604	-		hypothetical protein
FUN_006721	contig_10	300731	301857	-		hypothetical protein
FUN_006722	contig_10	306944	308671	-		hypothetical protein
FUN_006723	contig_10	310801	313731	+		hypothetical protein
FUN_006725	contig_10	318428	323393	-		hypothetical protein
FUN_006726	contig_10	323961	325327	+		hypothetical protein
FUN_006727	contig_10	325910	328708	+	VPH1	H(+)-transporting V0 sector ATPase subunit a
FUN_006728	contig_10	329744	331688	+		hypothetical protein
FUN_006729	contig_10	333911	335001	+		hypothetical protein
FUN_006730	contig_10	336036	337115	+		hypothetical protein
FUN_006732	contig_10	339828	344778	-	HFM1	ATP-dependent DNA helicase MER3
FUN_006733	contig_10	345253	346738	-	gms1	UDP-galactose transporter Gms1
FUN_006734	contig_10	348031	354705	-	MLP1	Protein mlp1
FUN_006735	contig_10	355545	356285	+		hypothetical protein
FUN_006736	contig_10	356670	357554	+	COQ3	Hexaprenyldihydroxybenzoate methyltransferase, mitochondrial
FUN_006737	contig_10	357857	358376	-	MRPL27	60S ribosomal protein L27, mitochondrial
FUN_006738	contig_10	358593	361535	+		hypothetical protein
FUN_006739	contig_10	361938	364381	+	ACF2_2	endo-1,3-beta glucanase
FUN_006740	contig_10	365913	367357	-	VCX1	Vacuolar calcium ion transporter
FUN_006743	contig_10	373158	374779	+		hypothetical protein
FUN_006744	contig_10	374892	376260	-		hypothetical protein
FUN_006746	contig_10	381927	382661	-		hypothetical protein

Gene ID	Scaffold	Start	Stop	Strand	Name	Product
FUN_006747	contig_10	383493	384169	+		hypothetical protein
FUN_006748	contig_10	385536	387208	-		hypothetical protein
FUN_006750	contig_10	390765	392183	-		hypothetical protein
FUN_006751	contig_10	392517	393337	-		hypothetical protein
FUN_006753	contig_10	396889	398011	+		hypothetical protein
FUN_006755	contig_10	402108	403079	+		hypothetical protein
FUN_006756	contig_10	404013	405906	+		hypothetical protein
FUN_006759	contig_10	424423	425158	-		hypothetical protein
FUN_006761	contig_10	438253	439000	+		hypothetical protein
FUN_006762	contig_10	439297	442240	+		hypothetical protein
FUN_006763	contig_10	444099	445467	+		hypothetical protein
FUN_006764	contig_10	447041	447986	-		hypothetical protein
FUN_006765	contig_10	448798	450159	-		hypothetical protein
FUN_006766	contig_10	450797	452211	-	HIS5	histidinol-phosphate transaminase
FUN_006767	contig_10	454318	455952	-	MTG2	GTPase of the mitochondrial inner membrane that associates with the large ribosomal subunit
FUN_006768	contig_10	456257	457343	+		hypothetical protein
FUN_006771	contig_10	462440	465606	-	KLP1	Kinesin heavy chain
FUN_006772	contig_10	466381	467024	-	UBC2	Ubiquitin-conjugating enzyme E2 2
FUN_006773	contig_10	467736	469640	-		hypothetical protein
FUN_006774	contig_10	473509	475566	-		hypothetical protein
FUN_006776	contig_10	477752	478979	-		hypothetical protein
FUN_006777	contig_10	479461	480393	-	RPL7_1	60S ribosomal protein L7
FUN_006778	contig_10	480975	483613	+	NCL1	tRNA (cytosine-5-)-methyltransferase ncl1
FUN_006779	contig_10	483961	484968	-	COQ5	2-hexaprenyl-6-methoxy-1,4-benzoquinone methyltransferase
FUN_006780	contig_10	485191	486734	+		hypothetical protein
FUN_006782	contig_10	489246	494236	+	SNQ2_1	ATP-binding cassette transporter snq2
FUN_006783	contig_10	495641	497849	+	PCK1	Protein kinase C-like 1
FUN_006784	contig_10	498640	501577	-		hypothetical protein
FUN_006785	contig_10	503148	505130	-	PKC1_2	Serine/threonine kinase
FUN_006786	contig_10	510794	511585	-	ubc8	ubiquitin-conjugating enzyme E2 H
FUN_006787	contig_10	512794	513880	+	ECM31	cell wall bioproteinsis and architecture protein
FUN_006788	contig_10	514259	514595	-	ATG12	Ubiquitin-like protein
FUN_006789	contig_10	515718	517582	-	DBF4	Cdc7p-Dbf4p kinase complex regulatory subunit
FUN_006791	contig_10	520609	521652	+		hypothetical protein
FUN_006792	contig_10	523672	525777	+		hypothetical protein
FUN_006794	contig_10	530602	532255	-		hypothetical protein
FUN_006795	contig_10	533054	538623	+		hypothetical protein
FUN_006796	contig_10	539223	540343	+		hypothetical protein
FUN_006797	contig_10	540780	542943	+	IKS1	putative serine/threonine-protein kinase iks1
FUN_006798	contig_10	543710	547068	+		hypothetical protein
FUN_006799	contig_10	548285	549694	+		hypothetical protein
FUN_006800	contig_10	550196	551463	-		hypothetical protein
FUN_006801	contig_10	551950	553428	-		hypothetical protein
FUN_006802	contig_10	553944	555008	+	MET22	3'(2'),5'-bisphosphate nucleotidase
FUN_006803	contig_10	555432	557332	-		hypothetical protein
FUN_006804	contig_10	558338	558907	+	MRPL6	54S ribosomal protein L6 mitochondrial
FUN_006805	contig_10	561442	562644	-		hypothetical protein
FUN_006806	contig_10	563251	563940	+	ORM1	sphingolipid homeostasis protein orm1
FUN_006807	contig_10	564474	565943	+		hypothetical protein
FUN_006808	contig_10	566045	566957	-	RSM24	37S ribosomal protein S24, mitochondrial
FUN_006809	contig_10	567428	568227	+	ALG13	N-acetylglucosaminyldiphosphodolichol N-acetylglucosaminyltransferase catalytic subunit alg13
FUN_006810	contig_10	569418	571059	+		hypothetical protein
FUN_006812	contig_10	574111	575495	+	EXG2_1	glucan exo-1,3-beta-glucosidase
FUN_006813	contig_10	579490	580918	+		hypothetical protein
FUN_006814	contig_10	585267	586681	+		hypothetical protein
FUN_006815	contig_10	590986	592135	+		hypothetical protein

Gene ID	Scaffold	Start	Stop	Strand	Name	Product
FUN_006816	contig_10	592460	594188	+		hypothetical protein
FUN_006817	contig_10	594360	595721	-		hypothetical protein
FUN_006818	contig_10	597307	601041	+		hypothetical protein
FUN_006819	contig_10	601709	603482	-		hypothetical protein
FUN_006820	contig_10	605095	607056	-	RGA2_2	Rho-type gtpase-activating protein
FUN_006822	contig_10	609912	610733	-		hypothetical protein
FUN_006823	contig_10	612156	615839	+		hypothetical protein
FUN_006824	contig_10	617185	617929	-		hypothetical protein
FUN_006825	contig_10	623299	625956	+		hypothetical protein
FUN_006827	contig_10	627622	628515	+	rpl15	60S ribosomal protein L15
FUN_006828	contig_10	629422	631920	+		hypothetical protein
FUN_006831	contig_10	641141	646279	+		hypothetical protein
FUN_006833	contig_10	651270	654188	+		hypothetical protein
FUN_006834	contig_10	656035	657008	+		hypothetical protein
FUN_006835	contig_10	657095	658943	-		hypothetical protein
FUN_006836	contig_10	660156	661028	+		hypothetical protein
FUN_006837	contig_10	661707	662940	-		hypothetical protein
FUN_006838	contig_10	663553	664904	-		hypothetical protein
FUN_006839	contig_10	666115	667674	+	DIA4	Serine--tRNA ligase, mitochondrial
FUN_006841	contig_10	670908	672305	+		hypothetical protein
FUN_006842	contig_10	673299	675356	+		hypothetical protein
FUN_006843	contig_10	677003	679871	-	ATG13	autophagy protein 13
FUN_006844	contig_10	682662	687533	+		hypothetical protein
FUN_006845	contig_10	687891	688865	-		hypothetical protein
FUN_006846	contig_10	689824	690726	+		hypothetical protein
FUN_006847	contig_10	692518	692905	+		hypothetical protein
FUN_006848	contig_10	693012	693872	+		hypothetical protein
FUN_006849	contig_10	697155	698714	-	HAA1	transcriptional activator haa1
FUN_006850	contig_10	700989	703585	+		hypothetical protein
FUN_006852	contig_10	706120	709478	+	RSC9	Chromatin structure-remodeling complex protein rsc9
FUN_006853	contig_10	710348	712474	-		hypothetical protein
FUN_006855	contig_10	716867	719573	-		hypothetical protein
FUN_006856	contig_10	720276	721306	+		hypothetical protein
FUN_006857	contig_10	721442	721903	-		hypothetical protein
FUN_006858	contig_10	722493	725897	+	SAS3	Histone acetyltransferase
FUN_006859	contig_10	728062	731549	+		hypothetical protein
FUN_006860	contig_10	741493	743869	-	SCT1	Glycerol-3-phosphate/dihydroxyacetone phosphate acyltransferase
FUN_006861	contig_10	744781	746567	-		hypothetical protein
FUN_006862	contig_10	747478	748186	-		hypothetical protein
FUN_006863	contig_10	749149	750854	+		hypothetical protein
FUN_006864	contig_10	751011	753621	-	CDC45	DNA replication initiation factor cdc45
FUN_006865	contig_10	754027	754754	-	FPS1	glycerol channel
FUN_006866	contig_10	758298	758951	-	SOD2_1	Superoxide dismutase [Mn], mitochondrial
FUN_006867	contig_10	761858	763377	+		hypothetical protein
FUN_006868	contig_10	763824	764993	-	GPI8	glycosylphosphatidylinositol anchor biosynthesis
FUN_006869	contig_10	765357	767616	+	OPT8	OPT super
FUN_006873	contig_10	778319	780229	-		hypothetical protein
FUN_006875	contig_10	785082	786809	+		hypothetical protein
FUN_006876	contig_10	787163	788031	-	QCR6	Cytochrome b-c1 complex subunit 6, mitochondrial
FUN_006877	contig_10	788425	789299	-		hypothetical protein
FUN_006878	contig_10	789729	793645	-	YME1	i-AAA protease yme1
FUN_006879	contig_10	798179	800618	+	PKH1	serine/threonine protein kinase
FUN_006880	contig_10	801901	803427	-	ssr3	SWI/SNF and RSC complex subunit Ssr3
FUN_006881	contig_10	803990	805719	+		hypothetical protein
FUN_006882	contig_10	806244	808515	+		hypothetical protein
FUN_006883	contig_10	812062	813426	+		hypothetical protein
FUN_006885	contig_10	814530	818315	-		hypothetical protein
FUN_006886	contig_10	818522	821406	+		hypothetical protein

Gene ID	Scaffold	Start	Stop	Strand	Name	Product
FUN_006887	contig_10	821922	825678	-		hypothetical protein
FUN_006889	contig_10	828327	829793	-		hypothetical protein
FUN_006890	contig_10	830352	831707	+	TAM41	Mitochondrial translocator assembly and maintenance protein 41
FUN_006891	contig_10	832176	833795	+		hypothetical protein
FUN_006892	contig_10	835148	836347	+		hypothetical protein
FUN_006893	contig_10	836605	837252	-		hypothetical protein
FUN_006894	contig_10	838009	839033	-	ALI1	Putative NADH-ubiquinone oxidoreductase 30.4 kDa subunit, mitochondrial
FUN_006895	contig_10	839449	842054	+	NOT4	transcriptional repressor proteinral negative regulator of transcription subunit 4
FUN_006897	contig_10	845837	847713	-		hypothetical protein
FUN_006898	contig_10	848491	849618	-		hypothetical protein
FUN_006899	contig_10	850489	852384	-		hypothetical protein
FUN_006900	contig_10	854615	858489	+		hypothetical protein
FUN_006901	contig_10	858993	860716	-	RTF1	RNA polymerase-associated protein rtf1
FUN_006902	contig_10	861074	861829	+	COQ7	ubiquinone biosynthesis monooxygenase Coq7
FUN_006904	contig_10	869523	871297	+		hypothetical protein
FUN_006905	contig_10	871939	874239	-		hypothetical protein
FUN_006907	contig_10	877761	878924	-		hypothetical protein
FUN_006908	contig_10	879471	880482	-		hypothetical protein
FUN_006909	contig_10	881031	882226	-	RER2	cis-prenyltransferase
FUN_006911	contig_10	884440	885235	-	PSF3	DNA replication protein
FUN_006912	contig_10	886233	892242	+	BLM3_4	Proteasome activator BLM10
FUN_006913	contig_10	892611	894069	-		hypothetical protein
FUN_006914	contig_10	894447	895383	-	rpl8	60S ribosomal protein L8
FUN_006915	contig_10	895787	897261	+	GPI18	ER membrane glycoprotein subunit of the GPI transamidase complex-like protein
FUN_006916	contig_10	898618	899681	-		hypothetical protein
FUN_006917	contig_10	900374	901663	-		hypothetical protein
FUN_006918	contig_10	902289	904097	-		hypothetical protein
FUN_006919	contig_10	904773	906408	+		hypothetical protein
FUN_006920	contig_10	906771	908569	-		hypothetical protein
FUN_006921	contig_10	913166	915964	-		hypothetical protein
FUN_006922	contig_10	916417	918564	-		hypothetical protein
FUN_006923	contig_10	919563	922795	-	CDC4	SCF ubiquitin ligase complex subunit cdc4
FUN_006924	contig_10	924675	927170	+		hypothetical protein
FUN_006927	contig_10	935061	936076	-		hypothetical protein
FUN_006930	contig_10	943298	945028	+		hypothetical protein
FUN_006931	contig_10	947154	949787	-		hypothetical protein
FUN_006932	contig_10	950717	957220	-		hypothetical protein
FUN_006933	contig_10	958555	959342	+	CDC31	Calcium-binding component of the spindle pole body (SPB) half-bridge
FUN_006934	contig_10	959598	961222	-	NDH51	NADH dehydrogenase [ubiquinone] flavoprotein 1, mitochondrial
FUN_006936	contig_10	966981	970551	-		hypothetical protein
FUN_006937	contig_10	971408	972341	+	RRF1	ribosome-recycling factor
FUN_006938	contig_10	972463	973615	-		hypothetical protein
FUN_006939	contig_10	974917	976402	-	LIS1	Lissencephaly-1
FUN_006940	contig_10	977116	979830	+		hypothetical protein
FUN_006942	contig_10	985776	987842	+	lcc1_3	laccase, multicopper oxidase, benzenediol:oxygen oxidorectuctase
FUN_006943	contig_10	989493	990348	-	RIB3	3,4-dihydroxy 2-butanone 4-phosphate synthase
FUN_006944	contig_10	991553	993479	-		hypothetical protein
FUN_006947	contig_10	1003800	1004987	+	FBP1	Fructose-1,6-bisphosphatase
FUN_006949	contig_10	1014839	1016043	+		hypothetical protein
FUN_006950	contig_10	1017206	1019134	+		hypothetical protein
FUN_006952	contig_10	1024135	1025217	-	PCL1_5	PHO85 cyclin-1
FUN_006953	contig_10	1030605	1040227	-	VPS13	Vacuolar protein sorting-associated protein 13

Gene ID	Scaffold	Start	Stop	Strand	Name	Product
FUN_006954	contig_10	1040953	1041513	+		hypothetical protein
FUN_006955	contig_10	1042229	1045304	-	SSA2	Hsp70 chaperone
FUN_006956	contig_10	1046599	1047539	+		hypothetical protein
FUN_006957	contig_10	1048294	1050066	+	GUT1	Glycerol kinase
FUN_006958	contig_10	1056933	1058307	+	ERG27	3-keto-steroid reductase
FUN_006960	contig_10	1062541	1063547	+		hypothetical protein
FUN_006961	contig_10	1064923	1066695	+		hypothetical protein
FUN_006962	contig_10	1066966	1070951	-		hypothetical protein
FUN_006963	contig_10	1071538	1074250	+		hypothetical protein
FUN_006964	contig_10	1075143	1076164	+		hypothetical protein
FUN_006965	contig_10	1076772	1078250	+	POT1	3-ketoacyl-CoA thiolase with broad chain length specificity
FUN_006966	contig_10	1078916	1080096	-		hypothetical protein
FUN_006971	contig_10	1090107	1091527	-		hypothetical protein
FUN_006972	contig_10	1092547	1094116	+		hypothetical protein
FUN_006973	contig_10	1094364	1095737	-		hypothetical protein
FUN_006975	contig_10	1097136	1097888	-		hypothetical protein
FUN_006976	contig_10	1098356	1100619	+	DUR4	urea permease
FUN_006977	contig_10	1103656	1104921	+		hypothetical protein
FUN_006979	contig_10	1109680	1110831	+		hypothetical protein
FUN_006981	contig_10	1115293	1117906	+		hypothetical protein
FUN_006982	contig_10	1120749	1121184	+		hypothetical protein
FUN_006984	contig_10	1133306	1134369	+		hypothetical protein
FUN_006986	contig_10	1137169	1139025	-		hypothetical protein
FUN_006987	contig_10	1140177	1141616	-		hypothetical protein
FUN_006988	contig_10	1146559	1147824	-		hypothetical protein
FUN_006989	contig_10	1151321	1153165	+		hypothetical protein
FUN_006991	contig_10	1156576	1159288	+		hypothetical protein
FUN_006993	contig_10	1170053	1171431	+		hypothetical protein
FUN_006994	contig_10	1175252	1177323	-		hypothetical protein
FUN_006995	contig_10	1178222	1180115	+		hypothetical protein
FUN_006996	contig_10	1180382	1181234	-		hypothetical protein
FUN_006997	contig_10	1181787	1183810	-	SMF3	NRAMP-like transporter smf-3
FUN_006998	contig_10	1184831	1188040	-	CDC24	Guanine nucleotide exchange factor for Cdc42p
FUN_006999	contig_10	1190385	1193095	+		hypothetical protein
FUN_007000	contig_10	1195266	1200054	+	CDR1_4	Multidrug resistance protein
FUN_007001	contig_10	1201438	1203773	-		hypothetical protein
FUN_007004	contig_10	1212894	1214143	+	CSH1_2	CSG1/SUR1-like protein
FUN_007005	contig_10	1216253	1218788	-		hypothetical protein
FUN_007006	contig_10	1222069	1223667	-		hypothetical protein
FUN_007007	contig_10	1224260	1224808	+		hypothetical protein
FUN_007008	contig_10	1225198	1226673	+	RAM1	CAAX farnesyltransferase (FTase) subunit beta
FUN_007009	contig_10	1226785	1227519	-		hypothetical protein
FUN_007011	contig_10	1236613	1237593	+	MPG1_1	mannose-1-phosphate guanyltransferase
FUN_007012	contig_10	1239121	1240417	+	MPG1_2	mannose-1-phosphate guanyltransferase
FUN_007013	contig_10	1242733	1244233	-		hypothetical protein
FUN_007015	contig_10	1255211	1256577	+		hypothetical protein
FUN_007016	contig_10	1257261	1259216	-	SPB4	ATP-dependent rRNA helicase spb4
FUN_007017	contig_10	1259954	1261151	+	DGK1	Diacylglycerol kinase
FUN_007018	contig_10	1262404	1264483	-		hypothetical protein
FUN_007019	contig_10	1266980	1267595	+	ubc15	Ubiquitin-conjugating enzyme E2 15
FUN_007021	contig_10	1284345	1285985	-	LAP3	bleomycin hydrolase
FUN_007023	contig_10	1288731	1290866	+		hypothetical protein
FUN_007024	contig_10	1291885	1292944	+		hypothetical protein
FUN_007025	contig_10	1293638	1295969	+		hypothetical protein
FUN_007026	contig_10	1297416	1299235	+		hypothetical protein
FUN_007027	contig_10	1303541	1305211	+		hypothetical protein
FUN_007028	contig_10	1305898	1306607	-		hypothetical protein
FUN_007029	contig_10	1307300	1307914	-	SNU23	U4/U6.U5 snRNP associated protein

Gene ID	Scaffold	Start	Stop	Strand	Name	Product
FUN_007032	contig_10	1313116	1316522	+		hypothetical protein
FUN_007033	contig_10	1318353	1318856	-		hypothetical protein
FUN_007034	contig_10	1320359	1321758	+		hypothetical protein
FUN_007036	contig_10	1323448	1326198	+		hypothetical protein
FUN_007038	contig_10	1328683	1330026	+		hypothetical protein
FUN_007039	contig_10	1330591	1335925	+	TOP2	DNA topoisomerase 2
FUN_007040	contig_10	1336899	1338616	-		hypothetical protein
FUN_007042	contig_10	1341296	1342497	+		hypothetical protein
FUN_007043	contig_10	1352878	1353386	-	RPL6_2	60S ribosomal protein L6
FUN_007048	contig_10	1361193	1362692	+		hypothetical protein
FUN_007049	contig_10	1371338	1373374	-	TFB1	RNA polymerase II transcription factor B subunit 1
FUN_007050	contig_10	1374410	1377675	+	KGD1	2-oxoglutarate dehydrogenase E1 component
FUN_007052	contig_10	1380328	1381455	-		hypothetical protein
FUN_007053	contig_10	1382001	1383372	+		hypothetical protein
FUN_007054	contig_10	1383553	1384845	-		hypothetical protein
FUN_007055	contig_10	1386219	1388911	-		hypothetical protein
FUN_007056	contig_11	2488	5410	+		hypothetical protein
FUN_007058	contig_11	10148	11787	-		hypothetical protein
FUN_007059	contig_11	13626	15479	+		hypothetical protein
FUN_007060	contig_11	15648	17344	-		hypothetical protein
FUN_007063	contig_11	22058	22939	-		hypothetical protein
FUN_007064	contig_11	23807	24275	-	YPD1	Phosphorelay intermediate protein
FUN_007065	contig_11	26747	28379	-	AAT1	aspartate transaminase aat1
FUN_007066	contig_11	28698	30974	+	GEP3	Mitochondrial ribosome small subunit bioproteinsis protein
FUN_007067	contig_11	31404	32492	-	MNN11	putative alpha-1,6-mannosyltransferase mnn11
FUN_007068	contig_11	33392	34707	+		hypothetical protein
FUN_007069	contig_11	35339	36645	+		hypothetical protein
FUN_007070	contig_11	37355	38161	+		hypothetical protein
FUN_007073	contig_11	45181	45552	+		hypothetical protein
FUN_007074	contig_11	46652	58343	+	TOM1	E3 ubiquitin-protein ligase tom1
FUN_007076	contig_11	61961	65534	-		hypothetical protein
FUN_007077	contig_11	66654	67720	-		hypothetical protein
FUN_007078	contig_11	71980	72754	-		hypothetical protein
FUN_007079	contig_11	73271	74262	+	AYR1_1	NADPH-dependent 1-acyl dihydroxyacetone phosphate reductase
FUN_007080	contig_11	77105	79234	-		hypothetical protein
FUN_007081	contig_11	80715	81692	+		hypothetical protein
FUN_007082	contig_11	81940	82779	+		hypothetical protein
FUN_007084	contig_11	84743	85879	+		hypothetical protein
FUN_007086	contig_11	92907	95216	+		hypothetical protein
FUN_007087	contig_11	95279	96946	-		hypothetical protein
FUN_007089	contig_11	99676	101763	-		hypothetical protein
FUN_007090	contig_11	101944	102705	+	NRK1	ribosylnicotinamide kinase
FUN_007091	contig_11	102981	104281	-	EAF3	Esa1p-associated factor
FUN_007092	contig_11	104711	105162	+		hypothetical protein
FUN_007093	contig_11	106022	107405	-		hypothetical protein
FUN_007095	contig_11	113196	117827	-	dcl2	Dicer-like protein 2
FUN_007096	contig_11	118436	121764	+	VAM6	Vacuolar morphoproteinsis protein 6
FUN_007097	contig_11	122534	125026	+		hypothetical protein
FUN_007098	contig_11	125390	127535	-		hypothetical protein
FUN_007099	contig_11	129164	129844	+	YFH1	Mitochondrial matrix iron chaperone
FUN_007100	contig_11	130205	130506	-	DPH3	Diphthamide biosynthesis protein 3
FUN_007101	contig_11	130702	133049	+	SEC6	SNARE-binding exocyst subunit S6
FUN_007103	contig_11	135857	136878	-		hypothetical protein
FUN_007105	contig_11	143979	144416	-		hypothetical protein
FUN_007106	contig_11	144761	145257	+	MHF1	MHF histone-fold complex component
FUN_007108	contig_11	147967	149276	-		hypothetical protein
FUN_007109	contig_11	150509	151699	+		hypothetical protein

Gene ID	Scaffold	Start	Stop	Strand	Name	Product
FUN_007110	contig_11	151994	152461	+		hypothetical protein
FUN_007111	contig_11	153207	154072	+		hypothetical protein
FUN_007112	contig_11	158679	160289	-	MGS1	DNA-dependent ATPase mgs1
FUN_007113	contig_11	160617	162157	+	BNA3	arylformamidase
FUN_007114	contig_11	170792	173689	+	SSN6	glucose repression mediator protein
FUN_007116	contig_11	177411	179558	+	KRE6_2	beta-glucan synthesis-associated protein
FUN_007117	contig_11	180996	182123	+		hypothetical protein
FUN_007118	contig_11	182362	186342	-	SEC31	protein transport protein S31
FUN_007119	contig_11	186642	187145	+		hypothetical protein
FUN_007120	contig_11	187520	188058	-		hypothetical protein
FUN_007121	contig_11	188361	188875	+	COX12	Cytochrome c oxidase subunit 6B
FUN_007122	contig_11	189652	192765	-		hypothetical protein
FUN_007125	contig_11	203771	206531	+		hypothetical protein
FUN_007126	contig_11	207108	208918	+	DPL1	Dihydrosphingosine phosphate lyase
FUN_007127	contig_11	209093	209870	-	mrpl16	39S ribosomal protein L16, mitochondrial
FUN_007128	contig_11	210150	213699	+	rfc1_1	DNA replication factor C complex subunit Rfc1
FUN_007132	contig_11	223773	224060	-	MRPL33	39S ribosomal protein L33, mitochondrial
FUN_007134	contig_11	229020	229811	+	mge1	GrpE, mitochondrial
FUN_007135	contig_11	230395	231476	-	rpb3	RNA polymerase II subunit 3
FUN_007136	contig_11	233290	234135	+		hypothetical protein
FUN_007137	contig_11	235014	235856	+	RPS6	40S ribosomal protein S6
FUN_007138	contig_11	237873	239105	-	PRS4	ribose phosphate diphosphokinase subunit prs4
FUN_007139	contig_11	240353	242162	-		hypothetical protein
FUN_007140	contig_11	247199	248665	-	MSF1	phenylalanyl-tRNA synthetase alpha subunit, mitochondrial
FUN_007141	contig_11	249144	250168	+	DIC1	Mitochondrial dicarboxylate transporter
FUN_007142	contig_11	250424	251251	-		hypothetical protein
FUN_007143	contig_11	254282	254879	+	BET4	Rab geranylgeranyltransferase
FUN_007144	contig_11	255417	261929	+		hypothetical protein
FUN_007147	contig_11	268359	271835	-		hypothetical protein
FUN_007148	contig_11	273127	276358	+		hypothetical protein
FUN_007149	contig_11	277472	280911	+		hypothetical protein
FUN_007150	contig_11	282472	282821	+	MOB1	Mitotic exit network component
FUN_007151	contig_11	283988	285157	-	EGL2	Endoglucanase EG-II
FUN_007152	contig_11	286459	288900	-		hypothetical protein
FUN_007153	contig_11	289496	291908	-	ACS2	acetyl-coenzyme A synthetase 2
FUN_007154	contig_11	293507	294301	-		hypothetical protein
FUN_007155	contig_11	295449	296232	+		hypothetical protein
FUN_007160	contig_11	301852	303172	+		hypothetical protein
FUN_007162	contig_11	304623	306464	+	MRS6	Rab proteins geranylgeranyltransferase component A
FUN_007163	contig_11	321082	321843	-		hypothetical protein
FUN_007164	contig_11	322492	322861	+	rps30a	40S ribosomal protein S30
FUN_007165	contig_11	323637	324248	+		hypothetical protein
FUN_007166	contig_11	325036	329636	+		hypothetical protein
FUN_007167	contig_11	330250	335589	+		hypothetical protein
FUN_007168	contig_11	338471	340882	+		hypothetical protein
FUN_007169	contig_11	341374	342978	-		hypothetical protein
FUN_007170	contig_11	343172	343711	+	cyp1	Peptidyl-prolyl cis-trans isomerase-like 1
FUN_007171	contig_11	343872	345200	-		hypothetical protein
FUN_007173	contig_11	347699	348388	-		hypothetical protein
FUN_007174	contig_11	349699	350384	-		hypothetical protein
FUN_007178	contig_11	357510	358243	+		hypothetical protein
FUN_007179	contig_11	358587	360142	+	ARG8	acetylornithine aminotransferase
FUN_007180	contig_11	360898	364731	+		hypothetical protein
FUN_007181	contig_11	365982	367073	+	RFG1_1	slightly ste11-like protein
FUN_007182	contig_11	367552	369693	+	RFG1_2	slightly ste11-like protein
FUN_007183	contig_11	370182	372599	+		hypothetical protein
FUN_007185	contig_11	378535	379248	+		hypothetical protein
FUN_007186	contig_11	381564	383262	-		hypothetical protein

Gene ID	Scaffold	Start	Stop	Strand	Name	Product
FUN_007187	contig_11	390042	391119	+		hypothetical protein
FUN_007190	contig_11	396637	400225	+	LYS2	large subunit of alpha-aminoadipate reductase
FUN_007191	contig_11	400438	403726	-		hypothetical protein
FUN_007192	contig_11	403926	404992	-		hypothetical protein
FUN_007193	contig_11	405845	406719	+		hypothetical protein
FUN_007197	contig_11	419962	421001	-		hypothetical protein
FUN_007198	contig_11	423132	424420	+		hypothetical protein
FUN_007199	contig_11	424986	425628	+		hypothetical protein
FUN_007201	contig_11	426974	427996	-		hypothetical protein
FUN_007202	contig_11	428927	430503	+		hypothetical protein
FUN_007203	contig_11	430721	432605	-	MSC7	Meiotic Sister-Chromatid recombination aldehyde dehydrogenase
FUN_007204	contig_11	432908	434423	-		hypothetical protein
FUN_007205	contig_11	435318	436499	-		hypothetical protein
FUN_007207	contig_11	440791	441641	+		hypothetical protein
FUN_007208	contig_11	442736	444208	+		hypothetical protein
FUN_007210	contig_11	448608	450386	+		hypothetical protein
FUN_007211	contig_11	450886	451915	-		hypothetical protein
FUN_007214	contig_11	459293	460630	+		hypothetical protein
FUN_007215	contig_11	461328	462542	+		hypothetical protein
FUN_007216	contig_11	462670	463159	-		hypothetical protein
FUN_007217	contig_11	464027	464851	-	VMA4	V-ATPase V1 sector subunit E
FUN_007218	contig_11	465437	467292	+		hypothetical protein
FUN_007219	contig_11	467528	469638	+		hypothetical protein
FUN_007220	contig_11	469825	471109	+		hypothetical protein
FUN_007221	contig_11	472198	472848	+		hypothetical protein
FUN_007222	contig_11	472919	473812	-		hypothetical protein
FUN_007223	contig_11	478132	480626	-		hypothetical protein
FUN_007224	contig_11	483691	485250	-		hypothetical protein
FUN_007225	contig_11	486751	487399	-		hypothetical protein
FUN_007226	contig_11	488417	489520	+		hypothetical protein
FUN_007227	contig_11	489638	492328	-		hypothetical protein
FUN_007228	contig_11	492895	493982	-		hypothetical protein
FUN_007229	contig_11	494750	496271	+		hypothetical protein
FUN_007230	contig_11	496517	497304	+		hypothetical protein
FUN_007232	contig_11	509217	510884	+		hypothetical protein
FUN_007233	contig_11	512788	514672	+		hypothetical protein
FUN_007234	contig_11	515096	516213	+		hypothetical protein
FUN_007237	contig_11	522280	523620	-		hypothetical protein
FUN_007238	contig_11	529279	531761	-		hypothetical protein
FUN_007239	contig_11	534453	534991	+		hypothetical protein
FUN_007240	contig_11	535028	535879	-		hypothetical protein
FUN_007241	contig_11	541606	543214	-		hypothetical protein
FUN_007242	contig_11	544713	546401	+		hypothetical protein
FUN_007243	contig_11	546712	548575	-		hypothetical protein
FUN_007245	contig_11	550505	552689	-	FRP1_1	ferric-chelate reductase Frp1
FUN_007246	contig_11	553626	554259	+		hypothetical protein
FUN_007248	contig_11	556385	557226	+	EGD1	Nascent polypeptide-associated complex subunit beta
FUN_007249	contig_11	558299	559594	+		hypothetical protein
FUN_007250	contig_11	559790	561033	-	FMO1_1	monooxygenase
FUN_007251	contig_11	562104	563700	+		hypothetical protein
FUN_007252	contig_11	564074	565126	+		hypothetical protein
FUN_007253	contig_11	565602	566633	+		hypothetical protein
FUN_007254	contig_11	567110	569434	-	KIN3_2	G2-specific serine/threonine protein kinase
FUN_007255	contig_11	569730	571726	+		hypothetical protein
FUN_007256	contig_11	572153	573124	+		hypothetical protein
FUN_007257	contig_11	574503	577123	+		hypothetical protein
FUN_007258	contig_11	580955	581808	+		hypothetical protein

Gene ID	Scaffold	Start	Stop	Strand	Name	Product
FUN_007259	contig_11	582968	584758	+	SUB2	Suppressor of the cold-sensitive snRNP bioproteinsis mutant brr1-1
FUN_007260	contig_11	590726	593281	+		hypothetical protein
FUN_007261	contig_11	594872	596533	+	GLK1	glucokinase
FUN_007263	contig_11	601115	603136	+		hypothetical protein
FUN_007264	contig_11	603863	604806	+	SHB17	Sedoheptulose 1,7-bisphosphatase
FUN_007266	contig_11	606672	607241	-		hypothetical protein
FUN_007267	contig_11	608003	608645	-		hypothetical protein
FUN_007268	contig_11	609520	610287	-		hypothetical protein
FUN_007269	contig_11	610559	611250	+		hypothetical protein
FUN_007270	contig_11	612096	614364	+		hypothetical protein
FUN_007273	contig_11	622280	624295	-		hypothetical protein
FUN_007274	contig_11	624571	625700	+	hrf1	Protein transport protein yif1
FUN_007275	contig_11	626849	628959	-	PCL5	PHO85 cyclin-5
FUN_007277	contig_11	636471	637651	+		hypothetical protein
FUN_007279	contig_11	646191	647611	-	MRS2	magnesium ion transporter
FUN_007280	contig_11	649331	652061	-	ARG6	Protein arg-6, mitochondrial
FUN_007281	contig_11	652956	653611	-	SNC2	Vesicle membrane receptor protein (v-SNARE)
FUN_007282	contig_11	654839	656299	+		hypothetical protein
FUN_007283	contig_11	657182	658554	+		hypothetical protein
FUN_007284	contig_11	659697	661649	+	ADE4	amidophosphoribosyltransferase
FUN_007285	contig_11	662273	664669	+		hypothetical protein
FUN_007288	contig_11	675207	677750	-	RGP1	Golgi membrane exchange factor (Ric1p-Rgp1p) subunit
FUN_007291	contig_11	683410	685944	-		hypothetical protein
FUN_007293	contig_11	690173	692177	+		hypothetical protein
FUN_007296	contig_11	696646	698872	-		hypothetical protein
FUN_007298	contig_11	701565	703206	-	PDH1	ATP-binding cassette transporter CGR1
FUN_007299	contig_11	704266	704965	-	ARC19	Arp complex subunit
FUN_007300	contig_11	705266	707146	+	UBP6	deubiquitinating enzyme
FUN_007301	contig_11	707602	710103	+	rec8	R8 protein
FUN_007302	contig_11	717441	720421	+	GAT1	Sodium- and chloride-dependent GABA transporter 1
FUN_007303	contig_11	721553	722509	+		hypothetical protein
FUN_007304	contig_11	723586	725568	-		hypothetical protein
FUN_007306	contig_11	729774	730975	+		hypothetical protein
FUN_007307	contig_11	736218	737998	+		hypothetical protein
FUN_007309	contig_11	740725	742944	+		hypothetical protein
FUN_007310	contig_11	749840	751417	-		hypothetical protein
FUN_007311	contig_11	753338	755999	+		hypothetical protein
FUN_007312	contig_11	756848	758702	+		hypothetical protein
FUN_007313	contig_11	760041	764268	+		hypothetical protein
FUN_007314	contig_11	764680	765582	-		hypothetical protein
FUN_007315	contig_11	766014	767537	+		hypothetical protein
FUN_007316	contig_11	770149	772486	-	SIN3_2	Transcriptional regulatory protein sin3
FUN_007317	contig_11	773017	774903	-		hypothetical protein
FUN_007318	contig_11	775968	777264	+	SMC2_1	Structural maintenance of chromosomes protein 2
FUN_007320	contig_11	779672	780917	-	RMT2_1	Arginine N-methyltransferase 2
FUN_007321	contig_11	781196	781846	-		hypothetical protein
FUN_007322	contig_11	782921	783998	+	rfc1_2	DNA replication factor C complex subunit Rfc1
FUN_007323	contig_11	784598	785280	-		hypothetical protein
FUN_007324	contig_11	786358	787353	-		hypothetical protein
FUN_007326	contig_11	791648	792633	+		hypothetical protein
FUN_007330	contig_11	800727	801977	+		hypothetical protein
FUN_007331	contig_11	802016	804013	-		hypothetical protein
FUN_007332	contig_11	805259	808012	+		hypothetical protein
FUN_007333	contig_11	812899	814880	-		hypothetical protein
FUN_007334	contig_11	819519	822956	-	NEW1	[NU+] prion formation protein 1
FUN_007335	contig_11	823528	826612	-		hypothetical protein
FUN_007336	contig_11	827104	828745	-	CIT3	citrate synthase
FUN_007337	contig_11	829385	831373	+	ICL2	mitochondrial 2-methylisocitrate lyase

Gene ID	Scaffold	Start	Stop	Strand	Name	Product
FUN_007338	contig_11	835042	836211	+		hypothetical protein
FUN_007339	contig_11	837578	841137	-	SMC5	Structural maintenance of chromosomes protein 5
FUN_007340	contig_11	841409	842958	-	FUM1	fumarase fum1
FUN_007341	contig_11	844387	845819	+	SAN1	Ubiquitin-protein ligase
FUN_007342	contig_11	846563	848627	+	DBF2_2	serine/threonine-protein kinase dbf2
FUN_007345	contig_11	862103	862807	-		hypothetical protein
FUN_007346	contig_11	870572	872411	-		hypothetical protein
FUN_007348	contig_11	874018	874965	+		hypothetical protein
FUN_007349	contig_11	875470	875869	-		hypothetical protein
FUN_007350	contig_11	878085	879180	+		hypothetical protein
FUN_007351	contig_11	881038	882479	-		hypothetical protein
FUN_007352	contig_11	883794	885820	+	GAD1	glutamate decarboxylase gad1
FUN_007353	contig_11	887048	890271	+		hypothetical protein
FUN_007355	contig_11	893922	894612	-		hypothetical protein
FUN_007357	contig_11	897617	898313	-		hypothetical protein
FUN_007358	contig_11	898810	899362	+		hypothetical protein
FUN_007359	contig_11	899446	899952	-		hypothetical protein
FUN_007360	contig_11	900820	904301	+		hypothetical protein
FUN_007361	contig_11	904790	905934	-		hypothetical protein
FUN_007362	contig_11	906803	908072	-		hypothetical protein
FUN_007363	contig_11	909459	910784	+		hypothetical protein
FUN_007364	contig_11	911637	915532	+		hypothetical protein
FUN_007365	contig_11	915977	916639	+		hypothetical protein
FUN_007366	contig_11	917686	918656	+		hypothetical protein
FUN_007369	contig_11	923980	924812	+	RPL24	60S ribosomal protein L24
FUN_007373	contig_11	929283	929825	-		hypothetical protein
FUN_007374	contig_11	935236	935938	-		hypothetical protein
FUN_007376	contig_11	941443	945630	-	GIN4	serine/threonine-protein kinase gin4
FUN_007378	contig_11	961697	962226	+		hypothetical protein
FUN_007380	contig_11	965918	968429	+		hypothetical protein
FUN_007381	contig_11	972009	973464	+	ARG4	argininosuccinate lyase
FUN_007382	contig_11	974049	975227	+		hypothetical protein
FUN_007383	contig_11	977633	978242	-		hypothetical protein
FUN_007384	contig_11	978664	983283	+		hypothetical protein
FUN_007385	contig_11	986715	988478	-	HNM1	choline transporter
FUN_007386	contig_11	990971	991812	-	MCM1	transcription factor of the MADS box
FUN_007387	contig_11	993704	994642	+		hypothetical protein
FUN_007389	contig_11	1002595	1007299	+	DNF1	phospholipid transporting ATPase
FUN_007391	contig_11	1018727	1020559	-	TRM44_1	tRNA(Ser) Um(44) 2'-O-methyltransferase
FUN_007392	contig_11	1020892	1021595	-	TRM44_2	tRNA(Ser) Um(44) 2'-O-methyltransferase
FUN_007394	contig_11	1025111	1026045	+		hypothetical protein
FUN_007395	contig_11	1026254	1027651	-		hypothetical protein
FUN_007397	contig_11	1033071	1036065	+	STE11	ATP binding
FUN_007398	contig_11	1037085	1038336	+		hypothetical protein
FUN_007399	contig_11	1039434	1040589	+	RFC5	Replication factor C (RF-C) subunit
FUN_007401	contig_11	1041652	1042488	-		hypothetical protein
FUN_007402	contig_11	1042778	1043456	+		hypothetical protein
FUN_007403	contig_11	1045767	1047847	+		hypothetical protein
FUN_007404	contig_11	1048608	1050687	-	RAD7_1	UV-damaged DNA-binding protein rad7
FUN_007405	contig_11	1051355	1052481	-	RAD7_2	UV-damaged DNA-binding protein rad7
FUN_007406	contig_11	1053547	1056008	+	TSR1	ribosome bioproteinsis protein tsr1
FUN_007407	contig_11	1056456	1058196	-	utp7	putative U3 small nucleolar RNA-associated protein 7
FUN_007408	contig_11	1058861	1059911	-		hypothetical protein
FUN_007409	contig_11	1062540	1063598	-		hypothetical protein
FUN_007410	contig_11	1063970	1064964	-		hypothetical protein
FUN_007411	contig_11	1065267	1068858	+		hypothetical protein
FUN_007412	contig_11	1074478	1075686	+		hypothetical protein
FUN_007415	contig_11	1082856	1083495	+	TIF11	Translation initiation factor 1A
FUN_007416	contig_11	1083794	1086661	-	rad16	DNA repair protein RAD16

Gene ID	Scaffold	Start	Stop	Strand	Name	Product
FUN_007417	contig_11	1088161	1088715	-	cbp4	Assembly factor cbp4
FUN_007418	contig_11	1089847	1093179	+		hypothetical protein
FUN_007419	contig_11	1094517	1095573	+		hypothetical protein
FUN_007421	contig_11	1097337	1099208	+		hypothetical protein
FUN_007422	contig_11	1099854	1100333	+		hypothetical protein
FUN_007423	contig_11	1100802	1102604	-		hypothetical protein
FUN_007426	contig_11	1115281	1116427	-		hypothetical protein
FUN_007427	contig_11	1117607	1118771	-		hypothetical protein
FUN_007429	contig_11	1123973	1126212	-		hypothetical protein
FUN_007431	contig_11	1130754	1131540	-		hypothetical protein
FUN_007432	contig_11	1133213	1135503	-		hypothetical protein
FUN_007433	contig_11	1138081	1138703	+		hypothetical protein
FUN_007434	contig_11	1140082	1141600	+	DPH1	Diphthamide biosynthesis protein 1
FUN_007436	contig_11	1146124	1146832	+	RPS8A	ribosomal protein S8A
FUN_007438	contig_11	1149547	1150382	+	ATP5	ATP synthase F0 subcomplex subunit OSCP atp5
FUN_007439	contig_11	1150527	1151033	-	BTN1_1	battenin CLN3 protein
FUN_007440	contig_11	1151082	1152093	-	BTN1_2	battenin CLN3 protein
FUN_007441	contig_11	1153997	1156606	+		hypothetical protein
FUN_007442	contig_11	1158118	1159587	+	FBP26	Fructose-2,6-bisphosphatase
FUN_007444	contig_11	1164744	1167292	-		hypothetical protein
FUN_007445	contig_11	1168347	1169765	-	PDK2	[Pyruvate dehydrogenase (acetyl-transferring)] kinase isozyme 2
FUN_007446	contig_11	1170819	1171519	-	CYC1	iso-1-cytochrome c
FUN_007447	contig_11	1172324	1173371	+		hypothetical protein
FUN_007448	contig_11	1173748	1176988	-		hypothetical protein
FUN_007449	contig_11	1177429	1178205	+	RRG9	Required for respiratory growth protein 9 mitochondrial
FUN_007450	contig_11	1179113	1180603	+		hypothetical protein
FUN_007452	contig_11	1183232	1184185	-		hypothetical protein
FUN_007453	contig_11	1185074	1185856	-	ISA1	Iron-sulfur assembly protein 1
FUN_007454	contig_11	1186254	1187808	-		hypothetical protein
FUN_007455	contig_11	1188936	1190474	-	ERC1	ethionine resistance protein
FUN_007457	contig_11	1192687	1194022	-		hypothetical protein
FUN_007458	contig_11	1194602	1198515	+		hypothetical protein
FUN_007459	contig_11	1209306	1211891	+		hypothetical protein
FUN_007460	contig_11	1214749	1217110	+		hypothetical protein
FUN_007461	contig_11	1220305	1222538	+		hypothetical protein
FUN_007463	contig_11	1226191	1227839	-		hypothetical protein
FUN_007464	contig_11	1230088	1233841	+		hypothetical protein
FUN_007465	contig_11	1235616	1236425	+	vti1	t-SNARE VTI1
FUN_007466	contig_11	1239360	1240086	-		hypothetical protein
FUN_007467	contig_11	1242720	1244030	+		hypothetical protein
FUN_007468	contig_11	1245132	1246836	-		hypothetical protein
FUN_007469	contig_11	1247119	1248289	+		hypothetical protein
FUN_007470	contig_11	1253494	1255929	+		hypothetical protein
FUN_007471	contig_11	1257316	1258898	-		hypothetical protein
FUN_007472	contig_11	1260590	1262419	-		hypothetical protein
FUN_007473	contig_11	1262967	1263818	-		hypothetical protein
FUN_007474	contig_11	1265224	1266244	+		hypothetical protein
FUN_007475	contig_11	1267579	1270762	+		hypothetical protein
FUN_007476	contig_11	1270863	1273177	-		hypothetical protein
FUN_007478	contig_11	1276031	1277275	+		hypothetical protein
FUN_007479	contig_11	1279698	1280288	-		hypothetical protein
FUN_007480	contig_11	1280710	1282038	+	SEC17	vesicular-fusion protein S17
FUN_007481	contig_11	1282505	1284821	-		hypothetical protein
FUN_007482	contig_11	1285558	1287447	+		hypothetical protein
FUN_007483	contig_11	1288383	1289167	-	ATP7	ATP synthase d subunit
FUN_007484	contig_11	1289481	1290225	-	EGD2	GAL4 enhancer protein
FUN_007485	contig_11	1290701	1291265	-		hypothetical protein
FUN_007486	contig_11	1292194	1293426	-		hypothetical protein

Gene ID	Scaffold	Start	Stop	Strand	Name	Product
FUN_007488	contig_11	1295831	1296702	-	SAR1	COPII coat GTPase
FUN_007491	contig_11	1306578	1307363	+		hypothetical protein
FUN_007494	contig_11	1311359	1313299	-		hypothetical protein
FUN_007497	contig_11	1320040	1321813	-		hypothetical protein
FUN_007499	contig_11	1323766	1324753	+		hypothetical protein
FUN_007502	contig_11	1332497	1334214	+		hypothetical protein
FUN_007503	contig_11	1336866	1344384	+		hypothetical protein
FUN_007504	contig_11	1346866	1347477	+		hypothetical protein
FUN_007506	contig_11	1355087	1355935	-		hypothetical protein
FUN_007512	contig_12	31034	32511	+		hypothetical protein
FUN_007513	contig_12	33243	34886	-		hypothetical protein
FUN_007514	contig_12	35746	37779	+		hypothetical protein
FUN_007515	contig_12	39556	40499	+		hypothetical protein
FUN_007516	contig_12	41409	43281	-		hypothetical protein
FUN_007517	contig_12	44124	46035	-		hypothetical protein
FUN_007518	contig_12	46476	48190	+		hypothetical protein
FUN_007519	contig_12	49135	53674	+		hypothetical protein
FUN_007520	contig_12	54111	55349	+		hypothetical protein
FUN_007522	contig_12	70311	71642	-		hypothetical protein
FUN_007523	contig_12	72844	74841	+		hypothetical protein
FUN_007524	contig_12	75081	76793	-		hypothetical protein
FUN_007525	contig_12	78603	79604	-		hypothetical protein
FUN_007527	contig_12	83425	86053	-	GYP7_1	GTPase activating protein
FUN_007528	contig_12	86519	87433	-	GYP7_2	GTPase activating protein
FUN_007529	contig_12	87830	90917	-	CSE1	importin-alpha export receptor
FUN_007530	contig_12	91570	96405	+	TSC2	Tuberous sclerosis 2-like protein
FUN_007531	contig_12	97135	98279	+		hypothetical protein
FUN_007532	contig_12	98910	99752	+		hypothetical protein
FUN_007534	contig_12	101454	102387	-		hypothetical protein
FUN_007535	contig_12	104530	108420	-	ECM16	putative ATP-dependent RNA helicase DHR1
FUN_007536	contig_12	108762	109259	+		hypothetical protein
FUN_007537	contig_12	109482	110146	-	FES1	hsp70 nucleotide exchange factor fes1
FUN_007538	contig_12	111169	112729	+	ACT1_1	actin
FUN_007539	contig_12	113501	114210	+		hypothetical protein
FUN_007540	contig_12	117717	119488	-		hypothetical protein
FUN_007542	contig_12	123764	125008	+		hypothetical protein
FUN_007543	contig_12	125957	127092	-		hypothetical protein
FUN_007544	contig_12	127588	129516	+		hypothetical protein
FUN_007545	contig_12	129898	132970	+		hypothetical protein
FUN_007546	contig_12	133851	136881	+		hypothetical protein
FUN_007548	contig_12	138986	139542	-		hypothetical protein
FUN_007549	contig_12	141396	142322	+	AIM32	Altered inheritance of mitochondria protein 32
FUN_007550	contig_12	142437	145480	-		hypothetical protein
FUN_007551	contig_12	145804	147729	+		hypothetical protein
FUN_007554	contig_12	158761	160043	-	SER1	Phosphoserine transaminase
FUN_007555	contig_12	160466	163029	+	LYS4_2	mitochondrial Homoaconitase
FUN_007557	contig_12	165708	168150	-		hypothetical protein
FUN_007558	contig_12	168711	170569	-		hypothetical protein
FUN_007560	contig_12	174359	176273	-		hypothetical protein
FUN_007561	contig_12	176732	178606	-		hypothetical protein
FUN_007563	contig_12	183680	184929	-		hypothetical protein
FUN_007564	contig_12	185472	189574	+		hypothetical protein
FUN_007565	contig_12	189954	191441	-		hypothetical protein
FUN_007566	contig_12	192845	194732	+		hypothetical protein
FUN_007567	contig_12	200993	202215	+		hypothetical protein
FUN_007568	contig_12	202289	203171	-	ECI1	dodecenoyl-CoA isomerase
FUN_007569	contig_12	204206	205101	-		hypothetical protein
FUN_007570	contig_12	205390	211781	+		hypothetical protein
FUN_007571	contig_12	211888	213521	-		hypothetical protein

Gene ID	Scaffold	Start	Stop	Strand	Name	Product
FUN_007572	contig_12	213833	216135	+		hypothetical protein
FUN_007573	contig_12	216411	217430	+		hypothetical protein
FUN_007574	contig_12	218271	222263	-		hypothetical protein
FUN_007575	contig_12	222578	224597	+		hypothetical protein
FUN_007576	contig_12	225581	229360	+	PMC1_3	plasma membrane calcium
FUN_007577	contig_12	229788	232613	+	alp4	gamma tubulin complex Spc97/GCP2 subunit Alp4
FUN_007579	contig_12	236088	237125	+		hypothetical protein
FUN_007581	contig_12	239774	240764	+	PUP2	proteasome component pup2
FUN_007582	contig_12	241096	241786	-		hypothetical protein
FUN_007583	contig_12	243353	244991	-		hypothetical protein
FUN_007584	contig_12	246962	248689	-		hypothetical protein
FUN_007585	contig_12	253821	255545	+		hypothetical protein
FUN_007586	contig_12	256855	257808	+		hypothetical protein
FUN_007588	contig_12	267170	270201	+	PMA1	plasma membrane H+-ATPase
FUN_007589	contig_12	270972	271961	+		hypothetical protein
FUN_007590	contig_12	272608	274419	+		hypothetical protein
FUN_007591	contig_12	276091	276948	+		hypothetical protein
FUN_007592	contig_12	278327	279325	+		hypothetical protein
FUN_007593	contig_12	279942	283558	+	rpc2	DNA-directed RNA polymerase III complex subunit Rpc2
FUN_007595	contig_12	285208	286012	-		hypothetical protein
FUN_007596	contig_12	286403	288113	+	TUB1_2	alpha-tubulin
FUN_007597	contig_12	288694	291401	-	mcl1	DNA polymerase alpha accessory factor Mcl1
FUN_007598	contig_12	292160	292596	+	chz1	Histone H2A.Z-specific chaperone CHZ1
FUN_007599	contig_12	296164	302803	+	GLT1	glutamate synthase [NADH]
FUN_007600	contig_12	303419	306023	+	YME2	mitochondrial escape protein 2
FUN_007601	contig_12	306367	307074	-	BET1	protein transport protein bet1
FUN_007602	contig_12	307794	308818	-	ADK1	Adenylate kinase
FUN_007603	contig_12	309905	312067	-	PCF11	mRNA 3' end processing factor
FUN_007604	contig_12	312932	314378	+	IWS1	Transcription factor iws1
FUN_007605	contig_12	315430	318208	-		hypothetical protein
FUN_007606	contig_12	319566	320924	-	NUP57	Nucleoporin nup57
FUN_007607	contig_12	321351	321973	+	NdufA8	ndufa8, NADH-ubiquinone oxidoreductase complex I 19kd subunit
FUN_007609	contig_12	323680	324813	+		hypothetical protein
FUN_007610	contig_12	326232	327022	-	cox5	Cytochrome c oxidase subunit 5B, mitochondrial
FUN_007611	contig_12	328450	329746	+		hypothetical protein
FUN_007612	contig_12	330958	333705	+	HDA1	Histone deacetylase hda1
FUN_007613	contig_12	337867	339100	+	PHO80_1	Pho80p cyclin
FUN_007615	contig_12	343602	346955	+		hypothetical protein
FUN_007616	contig_12	348619	350608	-	ATP2	atp2, beta subunit of the F1 sector of mitochondrial F1F0 ATP synthase
FUN_007617	contig_12	351204	353558	-	GLC3	alpha-1,4-glucan branching enzyme
FUN_007618	contig_12	354173	356155	+	MCX1	ATP-binding protein
FUN_007619	contig_12	358761	360500	-	SPT23	SPT3 Dosage dependent suppressor of Ty-induced promoter mutations-like protein
FUN_007621	contig_12	371063	374267	-	GPI13	mannose-ethanolamine phosphotransferase gpi13
FUN_007623	contig_12	379208	382097	+	PUF3	mRNA binding protein puf3
FUN_007624	contig_12	383197	387478	-		hypothetical protein
FUN_007626	contig_12	393762	394699	+	CBF1	basic helix-loop-helix protein
FUN_007627	contig_12	395414	398669	+	FAR11	Factor arrest protein 11
FUN_007628	contig_12	400509	402324	+		hypothetical protein
FUN_007629	contig_12	402964	405324	-		hypothetical protein
FUN_007630	contig_12	407903	410382	-	VTC2_1	Phosphate metabolism transcription protein
FUN_007631	contig_12	410836	412827	-	ADE17	bifunctional phosphoribosylaminoimidazolecarboxamide formyltransferase/IMP cyclohydrolase
FUN_007632	contig_12	413278	414727	+		hypothetical protein
FUN_007633	contig_12	415761	416804	+	RCF2	Replication factor C, subunit RFC4
FUN_007634	contig_12	417228	419724	-		hypothetical protein

Gene ID	Scaffold	Start	Stop	Strand	Name	Product
FUN_007635	contig_12	420302	421128	+		hypothetical protein
FUN_007636	contig_12	421279	423546	-		hypothetical protein
FUN_007637	contig_12	424381	426340	-		hypothetical protein
FUN_007638	contig_12	428537	430084	+	FAH12	Oleate hydroxylase fah12
FUN_007639	contig_12	431105	433552	-	NdufS1	ndufs1 NADH-ubiquinone oxidoreductase subunit
FUN_007640	contig_12	434646	436313	-		hypothetical protein
FUN_007641	contig_12	438432	440109	+		hypothetical protein
FUN_007642	contig_12	440840	443650	+		hypothetical protein
FUN_007643	contig_12	444718	447537	+	SEY1	Dynamin-like GTPase that mediates homotypic ER fusion
FUN_007645	contig_12	449616	450419	-	YPT31	Rab GTPase ypt31
FUN_007646	contig_12	450822	454054	+		hypothetical protein
FUN_007647	contig_12	454809	456751	+		hypothetical protein
FUN_007648	contig_12	458568	463181	+		hypothetical protein
FUN_007649	contig_12	465400	466307	+	PET9	ADP/ATP carrier protein
FUN_007650	contig_12	466808	467491	-		hypothetical protein
FUN_007651	contig_12	468579	469739	+		hypothetical protein
FUN_007653	contig_12	479866	483823	+		hypothetical protein
FUN_007654	contig_12	484416	485632	-	MRL1	Cation-independent mannose-6-phosphate receptor CI-MPR
FUN_007655	contig_12	486036	487617	+	ERG24	erg24, C-14 sterol reductase
FUN_007656	contig_12	488516	490433	-		hypothetical protein
FUN_007658	contig_12	492922	493977	-		hypothetical protein
FUN_007659	contig_12	494731	496099	+		hypothetical protein
FUN_007660	contig_12	496905	497545	+		hypothetical protein
FUN_007661	contig_12	497963	499099	-		hypothetical protein
FUN_007662	contig_12	500802	502746	+		hypothetical protein
FUN_007663	contig_12	508806	509159	+		hypothetical protein
FUN_007664	contig_12	510644	512293	-	SIK3	Serine/threonine-protein kinase sik3
FUN_007665	contig_12	512805	514491	-		hypothetical protein
FUN_007666	contig_12	514608	516408	-		hypothetical protein
FUN_007667	contig_12	517045	518168	+		hypothetical protein
FUN_007669	contig_12	520541	522061	-		hypothetical protein
FUN_007670	contig_12	522392	523718	-		hypothetical protein
FUN_007671	contig_12	524123	526046	+		hypothetical protein
FUN_007672	contig_12	526598	529434	+		hypothetical protein
FUN_007673	contig_12	529610	531527	-		hypothetical protein
FUN_007674	contig_12	531701	533358	+		hypothetical protein
FUN_007675	contig_12	534597	536277	+		hypothetical protein
FUN_007680	contig_12	547961	548626	-		hypothetical protein
FUN_007682	contig_12	551195	553451	+		hypothetical protein
FUN_007685	contig_12	560635	561921	+		hypothetical protein
FUN_007686	contig_12	562541	563140	+		hypothetical protein
FUN_007691	contig_12	574620	575698	-		hypothetical protein
FUN_007694	contig_12	582810	584795	-	kri1	Kinetochore protein Spc24
FUN_007695	contig_12	585100	589319	+	SIP3	SNF1-interacting protein
FUN_007703	contig_12	606735	609679	-		hypothetical protein
FUN_007706	contig_12	614720	615459	-		hypothetical protein
FUN_007707	contig_12	616166	617456	+		hypothetical protein
FUN_007708	contig_12	618032	619276	+	RPN8	proteasome regulatory particle subunit
FUN_007710	contig_12	623426	624595	+		hypothetical protein
FUN_007711	contig_12	625815	626979	+	LEM3	alkylphosphocholine resistance protein lem3
FUN_007712	contig_12	627536	628438	+		hypothetical protein
FUN_007713	contig_12	630988	631460	+		hypothetical protein
FUN_007714	contig_12	632724	633599	+	RPS7A	ribosomal protein S7A
FUN_007715	contig_12	634194	635207	+		hypothetical protein
FUN_007716	contig_12	636220	637926	+		hypothetical protein
FUN_007717	contig_12	640580	641785	-		hypothetical protein
FUN_007718	contig_12	642136	643152	-	WWM1	WW domain-containing protein wwm1

Gene ID	Scaffold	Start	Stop	Strand	Name	Product
FUN_007719	contig_12	643692	648194	-		hypothetical protein
FUN_007720	contig_12	649170	649568	-		hypothetical protein
FUN_007721	contig_12	650593	651556	+		hypothetical protein
FUN_007722	contig_12	652022	652901	-	ERD2	endoplasmic reticulum retention protein
FUN_007723	contig_12	653286	655532	+		hypothetical protein
FUN_007726	contig_12	665182	667090	+		hypothetical protein
FUN_007727	contig_12	672539	674879	+	MIF2_3	mitotic fidelity of chromosome transmission-related protein
FUN_007728	contig_12	679967	681308	-		hypothetical protein
FUN_007729	contig_12	685240	686409	+		hypothetical protein
FUN_007730	contig_12	690845	693805	-		hypothetical protein
FUN_007732	contig_12	696331	700464	-		hypothetical protein
FUN_007733	contig_12	703096	704880	-		hypothetical protein
FUN_007735	contig_12	709478	710387	-		hypothetical protein
FUN_007736	contig_12	711255	712464	-		hypothetical protein
FUN_007737	contig_12	713202	714122	-		hypothetical protein
FUN_007738	contig_12	715693	718893	+	HGT3	Ribulose bisphosphate carboxylase large chain
FUN_007739	contig_12	719660	722146	+	PMT2	Dolichyl-phosphate-mannose--protein mannosyltransferase 2
FUN_007740	contig_12	729613	731538	+	TPK2_2	cAMP-dependent protein kinase catalytic subunit
FUN_007741	contig_12	733447	734710	-	MED6	Mediator of RNA polymerase II transcription subunit 6
FUN_007742	contig_12	734946	735839	+		hypothetical protein
FUN_007743	contig_12	735897	737695	-		hypothetical protein
FUN_007744	contig_12	738271	739097	-		hypothetical protein
FUN_007745	contig_12	739842	740712	-	SEC22	SNAP receptor
FUN_007746	contig_12	741438	741995	+		hypothetical protein
FUN_007747	contig_12	742088	744853	-	ABZ1	para-aminobenzoate synthase, (PABA)
FUN_007748	contig_12	746254	747257	-		hypothetical protein
FUN_007749	contig_12	748150	750920	-	YHM1	high copy suppressor of abf2
FUN_007750	contig_12	751455	752386	-		hypothetical protein
FUN_007751	contig_12	753605	754769	-	RFC4	replication factor C subunit 4
FUN_007752	contig_12	756338	757900	+		hypothetical protein
FUN_007753	contig_12	758521	759659	-		hypothetical protein
FUN_007754	contig_12	760018	764933	-		hypothetical protein
FUN_007755	contig_12	766090	767126	+		hypothetical protein
FUN_007756	contig_12	770804	772716	+	VEL1	velum formation-related protein
FUN_007757	contig_12	775730	776968	+	MET15_2	Homocysteine/cysteine synthase
FUN_007758	contig_12	778257	780567	+	FAA2	medium-chain fatty acid-CoA ligase faa2
FUN_007759	contig_12	781100	783143	+		hypothetical protein
FUN_007760	contig_12	783895	784839	-	SPT4	transcription elongation factor spt4
FUN_007761	contig_12	785260	786792	+	PPT1	Palmitoyl-protein thioesterase 1
FUN_007763	contig_12	789003	790275	-		hypothetical protein
FUN_007764	contig_12	791051	793687	-		hypothetical protein
FUN_007765	contig_12	794448	795631	+		hypothetical protein
FUN_007767	contig_12	800190	801687	+		hypothetical protein
FUN_007769	contig_12	805174	807471	+		hypothetical protein
FUN_007770	contig_12	811353	812388	-		hypothetical protein
FUN_007771	contig_12	816131	819133	+	SET2	histone methyltransferase set2
FUN_007773	contig_12	824105	825238	+	UGA1	4-aminobutyrate transaminase
FUN_007776	contig_12	831244	832749	+	RVB2	RuvB-like protein 2
FUN_007777	contig_12	834510	835144	+	MNN4_1	mannosyltransferase
FUN_007778	contig_12	835700	836728	+		hypothetical protein
FUN_007782	contig_12	847436	849112	-		hypothetical protein
FUN_007783	contig_12	849933	851198	-	YHM2	Mitochondrial DNA replication protein yhm2
FUN_007784	contig_12	852277	853948	-	CIT1	citrate (Si)-synthase
FUN_007785	contig_12	855756	857968	+		hypothetical protein
FUN_007786	contig_12	858719	859441	-		hypothetical protein
FUN_007787	contig_12	859927	861937	+		hypothetical protein
FUN_007788	contig_12	863306	865114	+		hypothetical protein

Gene ID	Scaffold	Start	Stop	Strand	Name	Product
FUN_007789	contig_12	865331	868086	-	VTC2_2	Phosphate metabolism transcription protein
FUN_007791	contig_12	871783	872292	-		hypothetical protein
FUN_007792	contig_12	872636	873214	+		hypothetical protein
FUN_007795	contig_12	882452	883366	-		hypothetical protein
FUN_007796	contig_12	887037	889455	-		hypothetical protein
FUN_007798	contig_12	891785	894673	-		hypothetical protein
FUN_007800	contig_12	906304	907258	-		hypothetical protein
FUN_007801	contig_12	908252	909359	-	RRP36	rRNA bioproteinsis protein rrp36
FUN_007802	contig_12	909825	910889	+		hypothetical protein
FUN_007804	contig_12	912601	917982	-	SEC16	vesicle coat component
FUN_007805	contig_12	918886	922877	+	NEO1	Putative aminophospholipid-translocase
FUN_007806	contig_12	923090	924297	-		hypothetical protein
FUN_007807	contig_12	925747	926278	-		hypothetical protein
FUN_007809	contig_12	931117	932316	-		hypothetical protein
FUN_007810	contig_12	937119	938339	+		hypothetical protein
FUN_007811	contig_12	938435	940139	-		hypothetical protein
FUN_007812	contig_12	943636	944474	-		hypothetical protein
FUN_007813	contig_12	945901	948004	+		hypothetical protein
FUN_007815	contig_12	955113	959742	+		hypothetical protein
FUN_007817	contig_12	963157	963948	+		hypothetical protein
FUN_007818	contig_12	964952	967041	+		hypothetical protein
FUN_007819	contig_12	968328	969460	+		hypothetical protein
FUN_007821	contig_12	972098	977281	-		hypothetical protein
FUN_007822	contig_12	977806	982434	+		hypothetical protein
FUN_007823	contig_12	983470	985009	+		hypothetical protein
FUN_007824	contig_12	985269	986494	-	HEM13	Coproporphyrinogen-III oxidase
FUN_007825	contig_12	987559	988021	+	ATG8	ubiquitin-like protein atg8
FUN_007826	contig_12	992684	993990	-	RCE1	CAAX prenyl protease
FUN_007827	contig_12	995009	997309	+		hypothetical protein
FUN_007828	contig_12	998419	999381	+	TIF6	Eukaryotic translation initiation factor 6
FUN_007829	contig_12	1000090	1001454	+		hypothetical protein
FUN_007831	contig_12	1012206	1013677	+	RAD23	UV excision repair protein rad23
FUN_007832	contig_12	1015152	1018165	+		hypothetical protein
FUN_007834	contig_12	1019340	1021494	+		hypothetical protein
FUN_007835	contig_12	1023629	1026813	+		hypothetical protein
FUN_007836	contig_12	1027519	1028691	+		hypothetical protein
FUN_007837	contig_12	1029283	1030367	+		hypothetical protein
FUN_007839	contig_12	1033967	1037047	-	YAT1	carnitine O-acetyltransferase yat1
FUN_007840	contig_12	1038402	1039857	+	rhp57	DNA repair protein rhp57
FUN_007842	contig_12	1041698	1044099	-		hypothetical protein
FUN_007843	contig_12	1044389	1046083	+		hypothetical protein
FUN_007844	contig_12	1046244	1048667	-		hypothetical protein
FUN_007845	contig_12	1048840	1050009	-		hypothetical protein
FUN_007846	contig_12	1050399	1050950	+	CWC21	RNA-splicing factor
FUN_007847	contig_12	1051812	1052671	-		hypothetical protein
FUN_007848	contig_12	1053334	1055455	-		hypothetical protein
FUN_007850	contig_12	1069437	1070896	-		hypothetical protein
FUN_007852	contig_12	1072980	1074541	-		hypothetical protein
FUN_007854	contig_12	1076000	1076560	+		hypothetical protein
FUN_007855	contig_12	1081149	1084280	+		hypothetical protein
FUN_007856	contig_12	1084681	1086138	-		hypothetical protein
FUN_007857	contig_12	1095792	1096598	-		hypothetical protein
FUN_007858	contig_12	1097503	1098299	+		hypothetical protein
FUN_007859	contig_12	1100038	1100784	+		hypothetical protein
FUN_007860	contig_12	1101297	1103281	-		hypothetical protein
FUN_007861	contig_12	1103886	1106240	-		hypothetical protein
FUN_007862	contig_12	1106529	1108167	-		hypothetical protein
FUN_007863	contig_12	1108788	1109660	+		hypothetical protein
FUN_007864	contig_12	1109870	1110988	-		hypothetical protein

Gene ID	Scaffold	Start	Stop	Strand	Name	Product
FUN_007865	contig_12	1111477	1113298	+		hypothetical protein
FUN_007867	contig_12	1115719	1117151	-		hypothetical protein
FUN_007870	contig_12	1122370	1124004	-		hypothetical protein
FUN_007871	contig_12	1125378	1126741	+		hypothetical protein
FUN_007872	contig_12	1130072	1132084	+		hypothetical protein
FUN_007873	contig_12	1132193	1132906	-		hypothetical protein
FUN_007874	contig_12	1133573	1134787	+		hypothetical protein
FUN_007877	contig_12	1143674	1144828	+		hypothetical protein
FUN_007878	contig_12	1145278	1146859	-		hypothetical protein
FUN_007879	contig_12	1147004	1147699	-		hypothetical protein
FUN_007885	contig_12	1165725	1167148	-		hypothetical protein
FUN_007886	contig_12	1169589	1171802	+	PFK26	6-phosphofructo-2-kinase
FUN_007889	contig_12	1187205	1188040	-		hypothetical protein
FUN_007890	contig_12	1188509	1189538	-		hypothetical protein
FUN_007891	contig_12	1191528	1192306	-		hypothetical protein
FUN_007893	contig_12	1195191	1197020	+		hypothetical protein
FUN_007896	contig_12	1206987	1209509	-		hypothetical protein
FUN_007897	contig_12	1221856	1225513	+	EFR3	plasma membrane localization protein
FUN_007898	contig_12	1226350	1228511	-		hypothetical protein
FUN_007899	contig_12	1229285	1231640	+		hypothetical protein
FUN_007900	contig_12	1234194	1237971	-		hypothetical protein
FUN_007901	contig_12	1238483	1241308	+		hypothetical protein
FUN_007905	contig_12	1245855	1246970	+	ORC5	Origin recognition complex subunit 5
FUN_007906	contig_12	1247576	1248059	-		hypothetical protein
FUN_007907	contig_12	1248600	1250654	+		hypothetical protein
FUN_007908	contig_12	1251320	1252846	+	ecm33_3	cell wall protein Ecm33
FUN_007911	contig_12	1257476	1258114	-		hypothetical protein
FUN_007913	contig_12	1260868	1262901	-		hypothetical protein
FUN_007914	contig_12	1264532	1265854	+		hypothetical protein
FUN_007916	contig_12	1276744	1279487	-		hypothetical protein
FUN_007917	contig_12	1280116	1281635	+		hypothetical protein
FUN_007918	contig_12	1281715	1282317	-	LOC1	60S ribosomal subunit assembly/export protein
FUN_007919	contig_12	1282550	1283178	+	VPS68	Vacuolar protein sorting-associated protein 68
FUN_007920	contig_12	1284509	1285365	+	PRE5	Proteasome subunit alpha type-6
FUN_007921	contig_12	1286538	1289224	+	MAK10	N-alpha-acetyltransferase, non-catalitic subunit
FUN_007922	contig_12	1290502	1292034	-	QCR2	ubiquinol-cytochrome c reductase core subunit 1
FUN_007923	contig_12	1292504	1294555	+	RPC82	RNA polymerase III subunit C82
FUN_007924	contig_12	1297169	1298521	-		hypothetical protein
FUN_007925	contig_12	1300517	1301275	+		hypothetical protein
FUN_007926	contig_12	1302012	1304328	+		hypothetical protein
FUN_007928	contig_12	1313393	1315466	+		hypothetical protein
FUN_007929	contig_12	1315927	1316172	-		hypothetical protein
FUN_007930	contig_12	1321172	1322364	-		hypothetical protein
FUN_007931	contig_12	1323028	1323702	+		hypothetical protein
FUN_007932	contig_12	1327580	1328678	-		hypothetical protein
FUN_007933	contig_12	1329872	1330958	-		hypothetical protein
FUN_007934	contig_12	1334113	1335579	+		hypothetical protein
FUN_007935	contig_12	1339356	1346198	+		hypothetical protein
FUN_007937	contig_12	1354526	1357563	+		hypothetical protein
FUN_007941	contig_13	15661	19789	+		hypothetical protein
FUN_007943	contig_13	23274	24897	-		hypothetical protein
FUN_007945	contig_13	26897	27906	+		hypothetical protein
FUN_007946	contig_13	28233	30580	-		hypothetical protein
FUN_007947	contig_13	34557	35091	+		hypothetical protein
FUN_007949	contig_13	44101	46013	+		hypothetical protein
FUN_007951	contig_13	71451	72809	-		hypothetical protein
FUN_007952	contig_13	73472	74292	+	HETE1	Vegetative incompatibility protein HET-E-1
FUN_007953	contig_13	80550	83798	+		hypothetical protein
FUN_007955	contig_13	86731	91179	+		hypothetical protein

Gene ID	Scaffold	Start	Stop	Strand	Name	Product
FUN_007956	contig_13	98610	103109	+		hypothetical protein
FUN_007958	contig_13	106868	109173	+		hypothetical protein
FUN_007959	contig_13	110249	111232	+		hypothetical protein
FUN_007960	contig_13	111326	113489	-		hypothetical protein
FUN_007961	contig_13	115386	116881	+		hypothetical protein
FUN_007962	contig_13	117136	118379	-	ELO2	Fatty acyl-CoA elongase/Polyunsaturated fatty acid specific elongation enzyme
FUN_007963	contig_13	118790	120440	+		hypothetical protein
FUN_007965	contig_13	125622	127821	+		hypothetical protein
FUN_007967	contig_13	128733	129394	+	EFM4	Protein-lysine N-methyltransferase efm4
FUN_007968	contig_13	129514	130569	-		hypothetical protein
FUN_007971	contig_13	137674	140676	-		hypothetical protein
FUN_007972	contig_13	141962	143173	-		hypothetical protein
FUN_007975	contig_13	153879	155073	-		hypothetical protein
FUN_007976	contig_13	159057	160594	+		hypothetical protein
FUN_007977	contig_13	161090	162811	+		hypothetical protein
FUN_007978	contig_13	164504	165861	+		hypothetical protein
FUN_007979	contig_13	169956	170494	+		hypothetical protein
FUN_007980	contig_13	181944	183802	-		hypothetical protein
FUN_007981	contig_13	186062	187118	+		hypothetical protein
FUN_007983	contig_13	192726	196532	-		hypothetical protein
FUN_007987	contig_13	211783	213095	+		hypothetical protein
FUN_007988	contig_13	213549	215483	-		hypothetical protein
FUN_007990	contig_13	225235	227778	+	GLG2	glycogenin glucosyltransferase
FUN_007991	contig_13	228550	230162	+		hypothetical protein
FUN_007992	contig_13	232971	234058	+		hypothetical protein
FUN_007993	contig_13	235110	236352	+		hypothetical protein
FUN_007994	contig_13	239791	240404	+		hypothetical protein
FUN_007995	contig_13	240970	244565	+		hypothetical protein
FUN_007996	contig_13	255222	255963	-		hypothetical protein
FUN_007997	contig_13	259202	260759	+		hypothetical protein
FUN_008000	contig_13	265666	266313	-		hypothetical protein
FUN_008001	contig_13	268643	269550	-		hypothetical protein
FUN_008002	contig_13	270249	272379	-		hypothetical protein
FUN_008003	contig_13	273058	274861	-	ARE2	Sterol O-acyltransferase 2 (Sterol-ester synthase 2)
FUN_008004	contig_13	275476	278202	+	APL4	clathrin associated protein complex large subunit
FUN_008005	contig_13	279600	281282	+		hypothetical protein
FUN_008006	contig_13	281382	282297	-		hypothetical protein
FUN_008007	contig_13	282647	285572	+		hypothetical protein
FUN_008009	contig_13	288052	288650	-	RPS12_1	40S ribosomal protein S12
FUN_008010	contig_13	289521	291407	-	RPS12_2	40S ribosomal protein S12
FUN_008011	contig_13	292897	293991	-		hypothetical protein
FUN_008012	contig_13	295026	296447	-	URH1	Uridine nucleosidase 1
FUN_008013	contig_13	296996	298799	+		hypothetical protein
FUN_008014	contig_13	300460	303712	-	AKL1	Ark- serine/threonine protein kinase
FUN_008015	contig_13	306440	308477	+	GRR1	SCF ubiquitin ligase complex subunit
FUN_008017	contig_13	312468	314114	+		hypothetical protein
FUN_008018	contig_13	315576	317497	-	BBP1	Branchpoint-bridging protein
FUN_008019	contig_13	318590	319443	+	GST2_2	Glutathione S-transferase 2
FUN_008020	contig_13	322816	325607	+		hypothetical protein
FUN_008021	contig_13	326729	327733	-		hypothetical protein
FUN_008022	contig_13	328587	330183	+		hypothetical protein
FUN_008023	contig_13	330507	332829	-		hypothetical protein
FUN_008024	contig_13	332847	334629	-		hypothetical protein
FUN_008025	contig_13	336641	337856	+		hypothetical protein
FUN_008026	contig_13	339696	341504	+		hypothetical protein
FUN_008027	contig_13	344481	347705	-	CPP1	tyrosine/serine/threonine protein phosphatase
FUN_008028	contig_13	350523	351561	+	RPL4B	60S ribosomal protein L4B
FUN_008029	contig_13	351827	352290	-		hypothetical protein

Gene ID	Scaffold	Start	Stop	Strand	Name	Product
FUN_008030	contig_13	352575	353643	+		hypothetical protein
FUN_008031	contig_13	354232	355893	-	PRP45	mRNA splicing protein
FUN_008032	contig_13	357068	361927	+		hypothetical protein
FUN_008033	contig_13	361955	363129	-		hypothetical protein
FUN_008034	contig_13	363815	365568	+		hypothetical protein
FUN_008036	contig_13	372391	378437	-	MOT1	TATA-binding protein-associated factor mot1
FUN_008037	contig_13	382031	382987	-	TPT1	tRNA 2'-phosphotransferase
FUN_008038	contig_13	385463	387216	-	GIN1	Gypsy retrotransposon integrase-like protein 1
FUN_008039	contig_13	391295	392181	+	RPL16A	60S ribosomal protein L16A
FUN_008040	contig_13	392846	394697	+		hypothetical protein
FUN_008041	contig_13	399223	401568	+		hypothetical protein
FUN_008042	contig_13	402618	405121	+	FKH2	transcription factor
FUN_008043	contig_13	406054	406876	-		hypothetical protein
FUN_008046	contig_13	412800	413838	+		hypothetical protein
FUN_008047	contig_13	414489	416643	+		hypothetical protein
FUN_008050	contig_13	422698	424766	+		hypothetical protein
FUN_008055	contig_13	434619	435963	+		hypothetical protein
FUN_008056	contig_13	436350	437381	-	RPS3	40S ribosomal protein S3
FUN_008057	contig_13	437902	440440	-	YMR1	phosphatidylinositol-3-phosphatase ymr1
FUN_008058	contig_13	443695	445759	+		hypothetical protein
FUN_008059	contig_13	446423	446982	+		hypothetical protein
FUN_008063	contig_13	454839	457121	+	EXG3	Glucan 1,3-beta-glucosidase 3
FUN_008065	contig_13	459971	460737	-		hypothetical protein
FUN_008066	contig_13	461671	464548	+		hypothetical protein
FUN_008068	contig_13	471998	473868	+		hypothetical protein
FUN_008069	contig_13	474348	475457	+		hypothetical protein
FUN_008070	contig_13	475655	476803	-		hypothetical protein
FUN_008072	contig_13	484291	486616	+		hypothetical protein
FUN_008073	contig_13	492399	495369	-		hypothetical protein
FUN_008076	contig_13	503707	505863	-		hypothetical protein
FUN_008077	contig_13	507065	508813	-		hypothetical protein
FUN_008079	contig_13	522360	523514	-		hypothetical protein
FUN_008080	contig_13	523828	526066	+	TOP3	DNA topoisomerase
FUN_008081	contig_13	527156	528319	+	STB3	DNA-binding proteins Bright/BRCAA1/RBP1 and proteins containing BRIGHT domain
FUN_008082	contig_13	531026	533436	-		hypothetical protein
FUN_008083	contig_13	534167	534593	-	soh1	Mediator of RNA polymerase II transcription subunit 31
FUN_008084	contig_13	535672	537458	+		hypothetical protein
FUN_008086	contig_13	541507	549281	+	TEF1	translation elongation factor EF-1 alpha
FUN_008087	contig_13	549694	551234	-		hypothetical protein
FUN_008089	contig_13	553193	553468	+		hypothetical protein
FUN_008090	contig_13	553874	554527	-		hypothetical protein
FUN_008091	contig_13	555178	556744	+	FUN31_1	serine/threonine protein kinase
FUN_008092	contig_13	557974	561608	+	FUN31_2	serine/threonine protein kinase
FUN_008093	contig_13	563195	565462	+		hypothetical protein
FUN_008094	contig_13	566126	566649	-	RPC11	RNA polymerase III C11 subunit
FUN_008095	contig_13	567466	568506	+	RDH1_3	short-chain alcohol dehydrogenase
FUN_008096	contig_13	569339	571125	-		hypothetical protein
FUN_008097	contig_13	572330	574344	+		hypothetical protein
FUN_008098	contig_13	576500	578062	+		hypothetical protein
FUN_008101	contig_13	584528	585815	-		hypothetical protein
FUN_008102	contig_13	586800	588062	-		hypothetical protein
FUN_008103	contig_13	590876	593462	+		hypothetical protein
FUN_008105	contig_13	598972	599571	+		hypothetical protein
FUN_008106	contig_13	599643	601879	-		hypothetical protein
FUN_008107	contig_13	602524	604838	+	YKU80	ATP-dependent DNA helicase yku80
FUN_008108	contig_13	605345	605713	+	PIN4_1	Peptidyl-prolyl cis-trans isomerase pin4
FUN_008109	contig_13	606268	609675	-		hypothetical protein
FUN_008110	contig_13	610664	611550	+		hypothetical protein

Gene ID	Scaffold	Start	Stop	Strand	Name	Product
FUN_008112	contig_13	616325	619807	+	ILS1	isoleucine--tRNA ligase
FUN_008113	contig_13	620601	624157	+		hypothetical protein
FUN_008116	contig_13	629178	631723	-		hypothetical protein
FUN_008117	contig_13	633563	634792	+		hypothetical protein
FUN_008120	contig_13	640209	642114	+		hypothetical protein
FUN_008121	contig_13	642334	643356	-		hypothetical protein
FUN_008122	contig_13	644501	645646	-		hypothetical protein
FUN_008124	contig_13	647637	648705	+		hypothetical protein
FUN_008125	contig_13	648943	649239	-		hypothetical protein
FUN_008126	contig_13	649706	650482	+		hypothetical protein
FUN_008128	contig_13	658404	659723	+	OYE32_4	NADH-dependent flavin oxidoreductase
FUN_008129	contig_13	660507	662620	+		hypothetical protein
FUN_008130	contig_13	665601	667599	-		hypothetical protein
FUN_008133	contig_13	673098	674093	+	SUI3	translation initiation factor eIF-2 beta subunit
FUN_008134	contig_13	674802	678125	+		hypothetical protein
FUN_008135	contig_13	678830	680266	+		hypothetical protein
FUN_008137	contig_13	681847	683070	+		hypothetical protein
FUN_008138	contig_13	683440	684651	-		hypothetical protein
FUN_008139	contig_13	685500	688741	-		hypothetical protein
FUN_008140	contig_13	689688	690950	+	NOP13	Nucleolar protein 13
FUN_008141	contig_13	691632	692747	+		hypothetical protein
FUN_008143	contig_13	701137	701942	+		hypothetical protein
FUN_008144	contig_13	702135	703352	-		hypothetical protein
FUN_008146	contig_13	712802	713789	+		hypothetical protein
FUN_008148	contig_13	717499	718898	-		hypothetical protein
FUN_008149	contig_13	722265	723790	+		hypothetical protein
FUN_008151	contig_13	727870	730421	-		hypothetical protein
FUN_008153	contig_13	732862	734058	+		hypothetical protein
FUN_008154	contig_13	734334	735267	-		hypothetical protein
FUN_008155	contig_13	736078	737086	-		hypothetical protein
FUN_008156	contig_13	737378	739149	+		hypothetical protein
FUN_008157	contig_13	742650	744101	+		hypothetical protein
FUN_008159	contig_13	751345	753373	-	FRS1	phenylalanine--tRNA ligase subunit beta
FUN_008160	contig_13	754124	755685	-	MKK1	Protein kinase C signaling pathway involved MAPKK protein
FUN_008161	contig_13	760167	760835	+		hypothetical protein
FUN_008162	contig_13	761096	762481	-		hypothetical protein
FUN_008163	contig_13	763266	765445	+	TPD3	protein phosphatase 2A structural subunit
FUN_008164	contig_13	765904	766261	-	TTR1	Glutaredoxin
FUN_008165	contig_13	766920	768950	+		hypothetical protein
FUN_008166	contig_13	769688	770518	+		hypothetical protein
FUN_008167	contig_13	810573	812120	-		hypothetical protein
FUN_008168	contig_13	814829	816529	+		hypothetical protein
FUN_008171	contig_13	824792	826960	+		hypothetical protein
FUN_008172	contig_13	828088	829911	-		hypothetical protein
FUN_008173	contig_13	831075	833063	+	DBP8_1	Putative RNA helicase
FUN_008174	contig_13	833249	834869	+	DBP8_2	Putative RNA helicase
FUN_008177	contig_13	841005	841548	-	NUT2	RNA polymerase II mediator complex subunit
FUN_008178	contig_13	841778	843178	+		hypothetical protein
FUN_008179	contig_13	850748	852353	-		hypothetical protein
FUN_008180	contig_13	854315	855807	-	RBG1	GTP-binding protein rbg1
FUN_008181	contig_13	856384	857074	+		hypothetical protein
FUN_008182	contig_13	857499	860644	+		hypothetical protein
FUN_008185	contig_13	865009	866637	-		hypothetical protein
FUN_008186	contig_13	867634	868568	-		hypothetical protein
FUN_008187	contig_13	871023	871710	-		hypothetical protein
FUN_008189	contig_13	878959	879819	+		hypothetical protein
FUN_008190	contig_13	880774	882532	-		hypothetical protein
FUN_008191	contig_13	883389	885494	-		hypothetical protein

Gene ID	Scaffold	Start	Stop	Strand	Name	Product
FUN_008192	contig_13	885894	887763	-	LEU3	Regulatory protein leu3
FUN_008193	contig_13	888625	889694	+		hypothetical protein
FUN_008196	contig_13	894875	895833	+		hypothetical protein
FUN_008197	contig_13	896844	897622	-		hypothetical protein
FUN_008198	contig_13	897902	898838	+		hypothetical protein
FUN_008199	contig_13	900714	903952	-		hypothetical protein
FUN_008201	contig_13	907376	909475	+		hypothetical protein
FUN_008202	contig_13	909609	910246	-		hypothetical protein
FUN_008203	contig_13	910513	911451	-		hypothetical protein
FUN_008204	contig_13	913606	914450	+		hypothetical protein
FUN_008205	contig_13	914638	918416	-		hypothetical protein
FUN_008206	contig_13	918904	920769	+		hypothetical protein
FUN_008207	contig_13	921245	922780	-		hypothetical protein
FUN_008208	contig_13	923346	924317	+		hypothetical protein
FUN_008209	contig_13	924831	926501	+		hypothetical protein
FUN_008210	contig_13	930330	932445	+		hypothetical protein
FUN_008211	contig_13	932945	933739	+		hypothetical protein
FUN_008212	contig_13	933900	934557	-		hypothetical protein
FUN_008213	contig_13	935185	936277	-		hypothetical protein
FUN_008215	contig_13	938610	940767	-		hypothetical protein
FUN_008216	contig_13	941530	943420	+		hypothetical protein
FUN_008217	contig_13	946162	947845	-		hypothetical protein
FUN_008218	contig_13	948757	950594	+		hypothetical protein
FUN_008221	contig_13	956034	957240	-		hypothetical protein
FUN_008222	contig_13	957945	958977	-		hypothetical protein
FUN_008224	contig_13	962522	964900	+		hypothetical protein
FUN_008225	contig_13	965304	967438	-		hypothetical protein
FUN_008226	contig_13	968264	968729	-	QCR8	Cytochrome b-c1 complex subunit 8, mitochondrial
FUN_008227	contig_13	969413	970029	+		hypothetical protein
FUN_008228	contig_13	971480	974196	+	AFG3	AAA ATPase afg3
FUN_008229	contig_13	975596	976442	+		hypothetical protein
FUN_008231	contig_13	982374	983291	+		hypothetical protein
FUN_008232	contig_13	983916	984353	+		hypothetical protein
FUN_008233	contig_13	984722	986068	-		hypothetical protein
FUN_008234	contig_13	989742	991243	+		hypothetical protein
FUN_008236	contig_13	993373	995175	-		hypothetical protein
FUN_008237	contig_13	996202	999180	+		hypothetical protein
FUN_008238	contig_13	1000534	1001640	+		hypothetical protein
FUN_008239	contig_13	1002662	1004128	+		hypothetical protein
FUN_008240	contig_13	1008235	1009260	-		hypothetical protein
FUN_008242	contig_13	1013378	1014727	-	AIM18	Altered inheritance of mitochondria protein 18 mitochondrial
FUN_008243	contig_13	1015036	1015927	+		hypothetical protein
FUN_008244	contig_13	1016296	1018548	+	SPP1	COMPASS (complex proteins associated with Set1p) component
FUN_008245	contig_13	1021650	1022713	+		hypothetical protein
FUN_008247	contig_13	1026432	1028841	-		hypothetical protein
FUN_008249	contig_13	1034391	1035967	-		hypothetical protein
FUN_008250	contig_13	1036654	1038073	+		hypothetical protein
FUN_008251	contig_13	1038408	1039838	-		hypothetical protein
FUN_008252	contig_13	1042104	1043055	-	BIM1	microtubule integrity protein mal3
FUN_008253	contig_13	1043474	1044838	-		hypothetical protein
FUN_008254	contig_13	1048149	1050620	-		hypothetical protein
FUN_008256	contig_13	1055544	1057651	-		hypothetical protein
FUN_008258	contig_13	1062113	1062528	+	MOH1	protein yippee-like moh1
FUN_008259	contig_13	1063043	1064283	+		hypothetical protein
FUN_008260	contig_13	1065199	1066316	+	HEK2	RNA binding protein, heterogenous nuclear RNP-K like protein
FUN_008263	contig_13	1071373	1072681	+	HUT1	UDP-galactose transporter

Gene ID	Scaffold	Start	Stop	Strand	Name	Product
FUN_008264	contig_13	1073404	1076498	+		hypothetical protein
FUN_008265	contig_13	1077324	1078125	+		hypothetical protein
FUN_008271	contig_13	1088075	1090162	-		hypothetical protein
FUN_008272	contig_13	1091064	1092613	+		hypothetical protein
FUN_008275	contig_13	1098133	1100595	+	VPS21	Vacuolar protein sorting-associated protein 21
FUN_008276	contig_13	1101053	1101994	-		hypothetical protein
FUN_008283	contig_13	1127990	1130044	-		hypothetical protein
FUN_008284	contig_13	1131199	1132614	+		hypothetical protein
FUN_008285	contig_13	1143905	1144863	+		hypothetical protein
FUN_008286	contig_13	1157308	1159002	+		hypothetical protein
FUN_008287	contig_13	1166652	1167767	-		hypothetical protein
FUN_008288	contig_13	1168595	1172571	-	CLU1	Intracellular distribution of mitochondria
FUN_008292	contig_13	1180054	1180482	+		hypothetical protein
FUN_008294	contig_13	1184236	1185372	+		hypothetical protein
FUN_008296	contig_13	1190314	1199058	+		hypothetical protein
FUN_008298	contig_13	1207439	1209658	-		hypothetical protein
FUN_008299	contig_13	1210192	1211315	+		hypothetical protein
FUN_008300	contig_13	1212136	1212862	-		hypothetical protein
FUN_008301	contig_13	1214333	1215309	-		hypothetical protein
FUN_008302	contig_13	1215946	1218048	-		hypothetical protein
FUN_008304	contig_13	1220901	1221614	+		hypothetical protein
FUN_008308	contig_13	1229745	1230721	+		hypothetical protein
FUN_008309	contig_13	1232977	1235098	-		hypothetical protein
FUN_008311	contig_13	1240162	1241431	+		hypothetical protein
FUN_008312	contig_13	1244438	1245097	-		hypothetical protein
FUN_008313	contig_13	1266556	1267847	+		hypothetical protein
FUN_008314	contig_13	1270121	1271170	+		hypothetical protein
FUN_008316	contig_13	1276382	1277576	+		hypothetical protein
FUN_008317	contig_13	1277911	1278975	-		hypothetical protein
FUN_008318	contig_14	1804	2580	+		hypothetical protein
FUN_008319	contig_14	2602	2892	+		hypothetical protein
FUN_008321	contig_14	7861	10080	+	HCM1	Forkhead transcription factor
FUN_008322	contig_14	11283	12064	-	HIS3	imidazoleglycerol-phosphate dehydratase
FUN_008323	contig_14	13081	13715	-	NdufA6	ndufa6 NADH-ubiquinone oxidoreductase subunit
FUN_008324	contig_14	13952	14615	+	BUD31	Component of the SF3b subcomplex of the U2 snRNP
FUN_008327	contig_14	28518	29949	+	ECM42	Arginine biosynthesis bifunctional protein ArgJ, mitochondrial
FUN_008328	contig_14	30383	32260	-		hypothetical protein
FUN_008329	contig_14	32676	34040	+	APM4	clathrin associated protein complex medium subunit
FUN_008330	contig_14	34349	38047	-	CDC60	cytosolic leucyl tRNA synthetase
FUN_008331	contig_14	39677	40912	+		hypothetical protein
FUN_008332	contig_14	41287	43581	-		hypothetical protein
FUN_008334	contig_14	49682	51406	-	ORC2	Origin recognition complex subunit 2
FUN_008335	contig_14	52568	54258	+		hypothetical protein
FUN_008336	contig_14	55110	55898	+		hypothetical protein
FUN_008337	contig_14	61472	62686	-		hypothetical protein
FUN_008338	contig_14	63931	66350	-		hypothetical protein
FUN_008339	contig_14	67791	69368	-		hypothetical protein
FUN_008341	contig_14	70416	71529	-		hypothetical protein
FUN_008343	contig_14	82420	82912	+		hypothetical protein
FUN_008344	contig_14	83246	85005	-	ELP3	Elongator subunit
FUN_008345	contig_14	85564	86861	+	LSC1	ligase of succinyl-coa
FUN_008346	contig_14	87210	88525	-		hypothetical protein
FUN_008347	contig_14	88959	90357	-	FYV10	GID complex subunit containing RING finger motif
FUN_008348	contig_14	90866	92822	-	ARB1	ABC transporter ATP-binding protein arb1
FUN_008349	contig_14	93516	94555	-		hypothetical protein
FUN_008350	contig_14	95918	99144	-	RAD16	DNA repair protein rad16
FUN_008351	contig_14	101052	104861	+		hypothetical protein
FUN_008352	contig_14	110682	111650	-		hypothetical protein

Gene ID	Scaffold	Start	Stop	Strand	Name	Product
FUN_008353	contig_14	112000	113141	-	mrpl7	54S ribosomal protein L7, mitochondrial
FUN_008354	contig_14	113658	116414	+		hypothetical protein
FUN_008355	contig_14	118886	119712	+		hypothetical protein
FUN_008356	contig_14	130153	131372	+		hypothetical protein
FUN_008357	contig_14	131749	133719	-		hypothetical protein
FUN_008358	contig_14	134585	136797	+	NHX1	monovalent cation:H+ antiporter, CPA1 (nhx1)
FUN_008359	contig_14	139352	140707	+	CEL6C	1,4-beta-D-glucan cellobiohydrolase cel6c
FUN_008362	contig_14	145576	146248	-		hypothetical protein
FUN_008363	contig_14	146874	148782	+	pli1	E3 SUMO-protein ligase pli1
FUN_008365	contig_14	154336	156769	-		hypothetical protein
FUN_008366	contig_14	158355	160578	+	TUP1	proteinral transcription repressor
FUN_008367	contig_14	170673	172255	+	AZF1_2	DNA-binding transcription factor
FUN_008368	contig_14	178466	179617	+	ERG3	c-5 sterol desaturase
FUN_008369	contig_14	181308	183450	-		hypothetical protein
FUN_008370	contig_14	186612	187991	+		hypothetical protein
FUN_008373	contig_14	193519	194403	+		hypothetical protein
FUN_008374	contig_14	194856	196157	-		hypothetical protein
FUN_008375	contig_14	196986	198168	-	HAP5	Transcriptional activator hap5
FUN_008376	contig_14	199284	200279	+	fcf2	dTDP-fucopyranose mutase
FUN_008378	contig_14	205212	205669	+		hypothetical protein
FUN_008379	contig_14	206197	207132	+		hypothetical protein
FUN_008380	contig_14	207552	208898	+		hypothetical protein
FUN_008381	contig_14	209397	210104	+		hypothetical protein
FUN_008382	contig_14	210683	211786	+		hypothetical protein
FUN_008383	contig_14	212746	215223	+		hypothetical protein
FUN_008384	contig_14	215720	217078	+	RIT1	tRNA A64-2'-O-ribosylphosphate transferase
FUN_008385	contig_14	217964	220228	-		hypothetical protein
FUN_008386	contig_14	220634	222999	+	BRE1	E3 ubiquitin-protein ligase bre1
FUN_008387	contig_14	223657	224979	-	POS5	NADH kinase pos5
FUN_008388	contig_14	225694	227125	+		hypothetical protein
FUN_008389	contig_14	227713	229939	+		hypothetical protein
FUN_008390	contig_14	230860	232719	+		hypothetical protein
FUN_008391	contig_14	233493	235411	-	GAL83	galactose metabolism-related protein
FUN_008393	contig_14	239558	240708	+	CSH1_3	CSG1/SUR1-like protein
FUN_008394	contig_14	241869	242978	-	KAE1	putative tRNA threonylcarbamoyladenosine biosynthesis protein kae1
FUN_008395	contig_14	243343	244912	+	RMS1	Ribosomal lysine N-methyltransferase 4
FUN_008396	contig_14	245370	247131	-	EPL1	Enhancer of polycomb-like protein 1
FUN_008397	contig_14	252911	254679	+		hypothetical protein
FUN_008398	contig_14	255901	257851	+		hypothetical protein
FUN_008400	contig_14	264045	266825	-		hypothetical protein
FUN_008401	contig_14	267490	269301	+	TRM6	tRNA (adenine(58)-N(1))-methyltransferase non-catalytic subunit trm6
FUN_008402	contig_14	269550	270125	-	ARC18	subunit of the Arp2/3 complex
FUN_008403	contig_14	270503	271787	+	RFC3	Subunit of heteropentameric Replication factor C (RF-C)
FUN_008406	contig_14	293343	296437	-		hypothetical protein
FUN_008407	contig_14	296763	299793	+		hypothetical protein
FUN_008411	contig_14	309036	310873	-		hypothetical protein
FUN_008412	contig_14	311610	313413	-	BUR1	serine/threonine protein kinase, CMGC, CDC2/CDK sub
FUN_008413	contig_14	314397	315448	-		hypothetical protein
FUN_008415	contig_14	322572	332103	-		hypothetical protein
FUN_008416	contig_14	333735	335009	+	DYS1	Deoxyhypusine synthase
FUN_008417	contig_14	335833	340104	+		hypothetical protein
FUN_008418	contig_14	343387	344505	+		hypothetical protein
FUN_008419	contig_14	344721	346217	-		hypothetical protein
FUN_008420	contig_14	346863	349186	+		hypothetical protein
FUN_008421	contig_14	349387	350232	-		hypothetical protein
FUN_008422	contig_14	355507	357009	-		hypothetical protein
FUN_008424	contig_14	361655	362707	+	sap49	Spliceosome-associated protein 49

Gene ID	Scaffold	Start	Stop	Strand	Name	Product
FUN_008425	contig_14	363231	363827	-		hypothetical protein
FUN_008426	contig_14	364281	364703	+		hypothetical protein
FUN_008428	contig_14	367220	368544	-		hypothetical protein
FUN_008430	contig_14	372366	373393	-	PAN5_1	2-dehydropantoate 2-reductase (Ketopantoate reductase) (KPA reductase) (KPR)
FUN_008431	contig_14	377182	378282	+		hypothetical protein
FUN_008432	contig_14	379823	381179	-		hypothetical protein
FUN_008434	contig_14	401047	402687	-	LYS9	saccharopine dehydrogenase (NADP+, L-glutamate-forming)
FUN_008435	contig_14	403028	405564	+	SEC15	Rab GTPase-binding exocyst subunit S15
FUN_008436	contig_14	405793	407433	-		hypothetical protein
FUN_008437	contig_14	407823	409260	-	RAI1	decapping endonuclease targeting mRNA
FUN_008439	contig_14	412667	414634	+		hypothetical protein
FUN_008440	contig_14	421353	421992	+	RMP1	Ribonuclease MRP protein subunit rmp1
FUN_008441	contig_14	422496	423641	+		hypothetical protein
FUN_008442	contig_14	423800	425181	-	RBG2	Ribosome-interacting GTPase 2
FUN_008443	contig_14	425716	427025	+		hypothetical protein
FUN_008445	contig_14	429191	436219	-	RLR1	THO2 plays a role in transcriptional elongation
FUN_008446	contig_14	436729	438677	-	ALV1	5-aminolevulinate synthase, mitochondrial
FUN_008448	contig_14	442016	443235	+	ADI1_1	1,2-dihydroxy-3-keto-5-methylthiopentene dioxygenase
FUN_008449	contig_14	444996	446067	-		hypothetical protein
FUN_008450	contig_14	448691	452078	-		hypothetical protein
FUN_008451	contig_14	453948	455524	-	GSH2	Glutathione synthetase
FUN_008452	contig_14	455784	457353	+	MMM1	ERMES complex subunit mmm1
FUN_008454	contig_14	462965	464502	+		hypothetical protein
FUN_008456	contig_14	470593	473111	-		hypothetical protein
FUN_008457	contig_14	473779	475714	-	VPS45	vacuolar protein sorting-associated protein 45
FUN_008458	contig_14	476474	477564	+		hypothetical protein
FUN_008461	contig_14	480531	484320	-		hypothetical protein
FUN_008462	contig_14	484619	487060	+		hypothetical protein
FUN_008466	contig_14	493625	494779	-	YOP1	ER membrane protein DP1/Yop1
FUN_008467	contig_14	495634	497958	+		hypothetical protein
FUN_008468	contig_14	498501	499943	-		hypothetical protein
FUN_008469	contig_14	504753	505266	+		hypothetical protein
FUN_008471	contig_14	506653	508193	-		hypothetical protein
FUN_008472	contig_14	511920	513035	+		hypothetical protein
FUN_008473	contig_14	514175	515101	+	ANKRD17	Ankyrin repeat domain-containing protein 17
FUN_008474	contig_14	517228	519399	-		hypothetical protein
FUN_008475	contig_14	520160	521797	+		hypothetical protein
FUN_008477	contig_14	527105	527910	+		hypothetical protein
FUN_008478	contig_14	529903	531638	-		hypothetical protein
FUN_008479	contig_14	533374	537858	+		hypothetical protein
FUN_008482	contig_14	542229	542812	+	PHO88	phosphate transporter (Pho88)
FUN_008483	contig_14	544732	545349	-		hypothetical protein
FUN_008484	contig_14	545736	547916	+		hypothetical protein
FUN_008485	contig_14	548917	551123	+	AMO1	peroxisomal copper amine oxidase
FUN_008486	contig_14	551682	552774	+		hypothetical protein
FUN_008491	contig_14	560283	561015	+		hypothetical protein
FUN_008497	contig_14	573755	574751	+		hypothetical protein
FUN_008498	contig_14	575914	578451	-	CLA4	Protein kinase
FUN_008499	contig_14	580406	582227	-		hypothetical protein
FUN_008500	contig_14	582611	584774	+	ain1	alpha-actinin
FUN_008502	contig_14	599763	601505	+	POP2	CCR4-NOT core DEDD RNase subunit
FUN_008503	contig_14	603154	604621	-	RPF1	Ribosome production factor 1
FUN_008504	contig_14	605138	607609	+	GRS1	Glycine--tRNA ligase 1, mitochondrial
FUN_008505	contig_14	609913	611435	+		hypothetical protein
FUN_008506	contig_14	613127	614018	-	HIS6	Enzyme that catalyzes the fourth step in the histidine pathway

Gene ID	Scaffold	Start	Stop	Strand	Name	Product
FUN_008507	contig_14	614176	615078	+	FAF1	pre-rRNA processing and 40S ribosomal subunit assembly
FUN_008508	contig_14	615548	619793	-	SNF2	transcriptional regulator
FUN_008509	contig_14	621012	622178	-	CIP2	carbohydrate-binding module 1
FUN_008510	contig_14	623818	624326	+	TXNL4A	Thioredoxin-like 4A
FUN_008511	contig_14	624939	627866	-		hypothetical protein
FUN_008514	contig_14	633902	634593	-		hypothetical protein
FUN_008515	contig_14	636587	637677	+		hypothetical protein
FUN_008516	contig_14	639841	641344	+		hypothetical protein
FUN_008517	contig_14	641490	643723	-		hypothetical protein
FUN_008518	contig_14	644847	648504	-	CTR9	protein required for normal CLN1 and CLN2 G1 cyclin expression
FUN_008519	contig_14	649043	652499	+	SEC8	exocyst subunit
FUN_008520	contig_14	652673	654261	-		hypothetical protein
FUN_008521	contig_14	654804	656286	-		hypothetical protein
FUN_008522	contig_14	656779	657888	-		hypothetical protein
FUN_008523	contig_14	658197	661217	-		hypothetical protein
FUN_008524	contig_14	662558	664090	-	SAT4	serine/threonine-protein kinase HAL4/sat4
FUN_008525	contig_14	667673	669722	-		hypothetical protein
FUN_008526	contig_14	669929	671434	+	LTV1	Protein ltv1
FUN_008527	contig_14	671761	672327	-	bet5	Trafficking protein particle complex subunit BET5
FUN_008528	contig_14	672676	675309	+		hypothetical protein
FUN_008529	contig_14	680879	682087	+	gsk3	glycogen synthase kinase 3
FUN_008530	contig_14	683873	687346	+	cbc1	Nuclear cap-binding protein subunit 1
FUN_008531	contig_14	687981	690215	+		hypothetical protein
FUN_008532	contig_14	698306	699988	+	TOS9	Gluconate transport-inducing protein required for gluconate-H+ symport
FUN_008533	contig_14	702324	703684	-	OLE1_1	stearoyl-CoA 9-desaturase
FUN_008535	contig_14	707688	708661	+		hypothetical protein
FUN_008536	contig_14	709167	713733	+	PEP3	tethering complex subunit
FUN_008538	contig_14	717475	717923	+		hypothetical protein
FUN_008539	contig_14	718091	719678	-		hypothetical protein
FUN_008540	contig_14	720161	721447	+		hypothetical protein
FUN_008541	contig_14	722399	724057	-		hypothetical protein
FUN_008542	contig_14	725245	726908	-		hypothetical protein
FUN_008544	contig_14	730849	731595	-		hypothetical protein
FUN_008545	contig_14	732101	732958	-		hypothetical protein
FUN_008548	contig_14	736167	737764	+	HRT2	Hairy/enhancer-of-split with YRPW motif protein 2
FUN_008550	contig_14	741644	742429	+		hypothetical protein
FUN_008551	contig_14	742585	743783	-		hypothetical protein
FUN_008552	contig_14	744545	745582	+		hypothetical protein
FUN_008553	contig_14	745713	747119	-		hypothetical protein
FUN_008554	contig_14	747739	748521	-		hypothetical protein
FUN_008559	contig_14	762263	764721	+		hypothetical protein
FUN_008560	contig_14	766560	768015	+		hypothetical protein
FUN_008561	contig_14	768216	768899	-		hypothetical protein
FUN_008562	contig_14	769437	772364	-		hypothetical protein
FUN_008563	contig_14	773474	774072	+		hypothetical protein
FUN_008564	contig_14	774702	776462	-		hypothetical protein
FUN_008565	contig_14	778296	779298	+		hypothetical protein
FUN_008566	contig_14	779582	780322	-	ubc1	Ubiquitin-conjugating enzyme E2 1
FUN_008567	contig_14	780992	782589	+	RPT2	ATPase of 26S proteasome regulatory subunit 4
FUN_008569	contig_14	786090	787415	-		hypothetical protein
FUN_008570	contig_14	787729	790028	-	DUR3_1	urea active transporter
FUN_008571	contig_14	791692	793326	+		hypothetical protein
FUN_008575	contig_14	800609	801688	+		hypothetical protein
FUN_008578	contig_14	807557	808686	-		hypothetical protein
FUN_008579	contig_14	809496	810570	-		hypothetical protein
FUN_008580	contig_14	813447	814270	-		hypothetical protein

Gene ID	Scaffold	Start	Stop	Strand	Name	Product
FUN_008581	contig_14	814634	816247	+	RPA49	DNA-directed RNA polymerase I subunit rpa49
FUN_008583	contig_14	817133	818330	-		hypothetical protein
FUN_008584	contig_14	820141	823703	-		hypothetical protein
FUN_008585	contig_14	824434	826834	+		hypothetical protein
FUN_008586	contig_14	827975	830541	-		hypothetical protein
FUN_008587	contig_14	831258	836402	+	YCF1	ATP-binding cassette glutathione S-conjugate transporter ycf1
FUN_008588	contig_14	838175	840262	+		hypothetical protein
FUN_008589	contig_14	841120	845055	+	SAC3	actin cytoskeleton and mitosis protein
FUN_008591	contig_14	851246	852608	+	AOS1	E1 ubiquitin-activating protein aos1
FUN_008592	contig_14	852924	854788	-	PBN1	protease B nonderepressible form
FUN_008593	contig_14	855064	856011	+		hypothetical protein
FUN_008596	contig_14	859450	861762	-		hypothetical protein
FUN_008597	contig_14	863095	864252	-		hypothetical protein
FUN_008599	contig_14	867832	869323	+	STE3	a-factor receptor
FUN_008600	contig_14	869946	874073	+		hypothetical protein
FUN_008602	contig_14	875451	876717	-	SIS1	Molecular chaperone (DnaJ super)
FUN_008603	contig_14	877198	878849	-	CBF5	centromere/microtubule-binding protein cbf5
FUN_008605	contig_14	883792	884921	-	UFD1	ubiquitin fusion degradation protein
FUN_008606	contig_14	885362	886627	+		hypothetical protein
FUN_008607	contig_14	888851	890737	+	PIN4_2	Peptidyl-prolyl cis-trans isomerase pin4
FUN_008609	contig_14	893530	894066	-	HTZ1	histone H2A.Z
FUN_008610	contig_14	895357	898030	+	ADI1_2	1,2-dihydroxy-3-keto-5-methylthiopentene dioxygenase
FUN_008611	contig_14	899217	901547	+	CTA8	Heat shock transcription factor
FUN_008612	contig_14	901864	902444	-	ADI1_3	1,2-dihydroxy-3-keto-5-methylthiopentene dioxygenase
FUN_008613	contig_14	903039	903883	-		hypothetical protein
FUN_008614	contig_14	904480	905776	+	VRG4	GDP-mannose transporter into the lumen of the Golgi
FUN_008618	contig_14	916466	917552	-	INO80_1	Putative DNA helicase ino80
FUN_008619	contig_14	919811	921132	+		hypothetical protein
FUN_008620	contig_14	921906	926579	-	INO80_2	Putative DNA helicase ino80
FUN_008621	contig_14	928279	929405	+	BRX1	Ribosome bioproteinsis protein brx1
FUN_008622	contig_14	929911	930984	-		hypothetical protein
FUN_008623	contig_14	932349	939596	-		hypothetical protein
FUN_008625	contig_14	943751	945280	+	SMI1	Cell wall assembly regulator
FUN_008626	contig_14	946161	946811	-		hypothetical protein
FUN_008627	contig_14	947480	949195	+		hypothetical protein
FUN_008628	contig_14	949585	950164	+	RIM1	ssDNA-binding protein, mitochondrial
FUN_008629	contig_14	950660	952772	+	MTO1	Mitochondrial Translation Optimization
FUN_008630	contig_14	953030	954428	-		hypothetical protein
FUN_008632	contig_14	958102	960107	+		hypothetical protein
FUN_008633	contig_14	962018	964367	-	MST12	Transcription factor mst12
FUN_008634	contig_14	969509	970101	+	RPL39	60S ribosomal protein L39
FUN_008635	contig_14	970679	971841	-		hypothetical protein
FUN_008636	contig_14	972287	975018	+		hypothetical protein
FUN_008638	contig_14	977424	979234	+		hypothetical protein
FUN_008639	contig_14	979471	980126	-		hypothetical protein
FUN_008643	contig_14	988975	991359	-		hypothetical protein
FUN_008645	contig_14	995723	996756	-		hypothetical protein
FUN_008647	contig_14	999730	1000904	+		hypothetical protein
FUN_008649	contig_14	1003576	1005096	-	CWC27	Peptidyl-prolyl isomerase cwc27
FUN_008650	contig_14	1005470	1007405	+	ale1	Lysophospholipid acyltransferase
FUN_008652	contig_14	1015093	1017027	-	SNF3_2	Plasma membrane low glucose sensor
FUN_008653	contig_14	1018739	1021028	-	NOP2	rRNA (cytosine-C5-)-methyltransferase nop2
FUN_008654	contig_14	1021490	1022173	+		hypothetical protein
FUN_008655	contig_14	1022541	1023225	-		hypothetical protein
FUN_008656	contig_14	1023870	1025747	-	HSP60	chaperonin
FUN_008662	contig_14	1039915	1041902	-		hypothetical protein
FUN_008663	contig_14	1044806	1045669	+		hypothetical protein
FUN_008664	contig_14	1046263	1047416	+		hypothetical protein

Gene ID	Scaffold	Start	Stop	Strand	Name	Product
FUN_008665	contig_14	1049400	1051567	-		hypothetical protein
FUN_008667	contig_14	1055980	1058203	+		hypothetical protein
FUN_008668	contig_14	1058940	1059895	+		hypothetical protein
FUN_008669	contig_14	1061337	1062323	-		hypothetical protein
FUN_008670	contig_14	1063161	1064753	-		hypothetical protein
FUN_008671	contig_14	1066615	1069597	+		hypothetical protein
FUN_008674	contig_14	1078393	1080125	-		hypothetical protein
FUN_008676	contig_14	1083813	1086119	-		hypothetical protein
FUN_008678	contig_14	1092143	1092691	+		hypothetical protein
FUN_008680	contig_14	1094720	1098519	+		hypothetical protein
FUN_008681	contig_14	1098940	1102198	+		hypothetical protein
FUN_008682	contig_14	1102820	1103170	-		hypothetical protein
FUN_008683	contig_14	1107170	1108111	-		hypothetical protein
FUN_008685	contig_14	1112581	1115732	-		hypothetical protein
FUN_008686	contig_14	1119869	1120895	+		hypothetical protein
FUN_008690	contig_14	1127786	1128875	-		hypothetical protein
FUN_008691	contig_14	1130219	1131655	+		hypothetical protein
FUN_008692	contig_14	1134023	1135231	-	XDJ1_1	DnaJ-like protein xdj1
FUN_008693	contig_14	1140197	1142093	+		hypothetical protein
FUN_008694	contig_14	1145468	1147380	-		hypothetical protein
FUN_008695	contig_14	1148091	1149524	-		hypothetical protein
FUN_008696	contig_14	1150395	1151956	-		hypothetical protein
FUN_008697	contig_14	1152681	1153480	+		hypothetical protein
FUN_008698	contig_14	1153614	1154950	-	ade1	Bifunctional purine biosynthetic protein ADE1
FUN_008700	contig_14	1157370	1160429	-		hypothetical protein
FUN_008701	contig_14	1160752	1161524	+		hypothetical protein
FUN_008702	contig_14	1161918	1162987	-		hypothetical protein
FUN_008703	contig_14	1167023	1167982	-		hypothetical protein
FUN_008704	contig_14	1171486	1172665	-		hypothetical protein
FUN_008706	contig_15	4705	8638	+		hypothetical protein
FUN_008707	contig_15	13241	15827	+		hypothetical protein
FUN_008708	contig_15	16532	17239	-		hypothetical protein
FUN_008709	contig_15	19204	20544	+		hypothetical protein
FUN_008710	contig_15	32067	32901	+	LAP1_1	Leucine aminopeptidase 1
FUN_008711	contig_15	33115	34047	-		hypothetical protein
FUN_008712	contig_15	35467	37370	+		hypothetical protein
FUN_008713	contig_15	37476	38414	-		hypothetical protein
FUN_008714	contig_15	39124	40075	-		hypothetical protein
FUN_008715	contig_15	41374	42521	-		hypothetical protein
FUN_008716	contig_15	44120	45785	-		hypothetical protein
FUN_008717	contig_15	46048	49660	-		hypothetical protein
FUN_008721	contig_15	60501	61928	+		hypothetical protein
FUN_008723	contig_15	64537	66144	-		hypothetical protein
FUN_008724	contig_15	67193	68071	-	RRP15	pre-60S ribosomal particles component
FUN_008725	contig_15	68280	69905	+	SRP54	Signal recognition particle
FUN_008727	contig_15	72970	74587	+		hypothetical protein
FUN_008730	contig_15	85236	88668	-		hypothetical protein
FUN_008731	contig_15	88848	89784	-		hypothetical protein
FUN_008732	contig_15	90400	91896	+	FMO1_2	monooxygenase
FUN_008733	contig_15	92495	95667	-		hypothetical protein
FUN_008734	contig_15	100887	102834	+		hypothetical protein
FUN_008735	contig_15	104199	106569	-	PRD1_1	metalloendopeptidase
FUN_008736	contig_15	108299	110281	-		hypothetical protein
FUN_008737	contig_15	115356	118918	+		hypothetical protein
FUN_008738	contig_15	120772	121725	-		hypothetical protein
FUN_008739	contig_15	123594	125289	+		hypothetical protein
FUN_008740	contig_15	126265	128121	+		hypothetical protein
FUN_008741	contig_15	128674	131117	-		hypothetical protein
FUN_008742	contig_15	132848	139649	-		hypothetical protein

Gene ID	Scaffold	Start	Stop	Strand	Name	Product
FUN_008743	contig_15	140375	142049	-		hypothetical protein
FUN_008744	contig_15	144223	145666	-		hypothetical protein
FUN_008746	contig_15	151135	151968	-		hypothetical protein
FUN_008748	contig_15	157431	160135	-		hypothetical protein
FUN_008749	contig_15	160371	161675	+	KTR4	putative mannosyltransferase ktr4
FUN_008750	contig_15	162279	163017	-	COX6	Cytochrome c oxidase subunit 6
FUN_008751	contig_15	163595	165308	-		hypothetical protein
FUN_008752	contig_15	170108	171883	+		hypothetical protein
FUN_008754	contig_15	177894	179642	+		hypothetical protein
FUN_008756	contig_15	182188	183902	+	HSX11	Ceramide glucosyltransferase
FUN_008758	contig_15	189359	191218	+		hypothetical protein
FUN_008759	contig_15	191315	197405	-		hypothetical protein
FUN_008760	contig_15	201250	203189	-		hypothetical protein
FUN_008761	contig_15	205320	206168	-		hypothetical protein
FUN_008765	contig_15	216957	219658	-		hypothetical protein
FUN_008767	contig_15	221818	222386	+		hypothetical protein
FUN_008768	contig_15	222682	223875	-		hypothetical protein
FUN_008771	contig_15	232936	239858	+		hypothetical protein
FUN_008774	contig_15	246454	247846	+		hypothetical protein
FUN_008775	contig_15	248133	249302	-	SGT1	Cochaperone protein
FUN_008776	contig_15	249892	250839	-		hypothetical protein
FUN_008778	contig_15	254871	255690	-		hypothetical protein
FUN_008779	contig_15	255924	258119	+		hypothetical protein
FUN_008782	contig_15	265326	267119	+		hypothetical protein
FUN_008783	contig_15	267453	269405	-	cut9	anaphase-promoting complex subunit Cut9
FUN_008784	contig_15	269741	270598	+	cwf16_2	Pre-mRNA-splicing factor cwf16
FUN_008785	contig_15	271351	272379	+		hypothetical protein
FUN_008786	contig_15	273781	276450	-		hypothetical protein
FUN_008787	contig_15	276997	278386	-		hypothetical protein
FUN_008789	contig_15	284894	286306	-		hypothetical protein
FUN_008790	contig_15	291056	291809	-		hypothetical protein
FUN_008793	contig_15	297553	299272	-		hypothetical protein
FUN_008795	contig_15	303070	304165	-		hypothetical protein
FUN_008796	contig_15	305425	306503	-		hypothetical protein
FUN_008797	contig_15	307188	307751	-		hypothetical protein
FUN_008798	contig_15	308764	310472	+		hypothetical protein
FUN_008799	contig_15	312202	313648	+		hypothetical protein
FUN_008800	contig_15	314617	316324	-		hypothetical protein
FUN_008801	contig_15	318337	320322	+	NRP1	Asparagine-rich protein (ARP protein)
FUN_008802	contig_15	321540	325106	-	YCG1	chromosome condensation complex Condensin, subunit G
FUN_008803	contig_15	325342	326539	+	CYS17	Cysteine synthase 1
FUN_008804	contig_15	326620	327950	-	NPY1	NADH pyrophosphatase
FUN_008805	contig_15	328284	329199	+		hypothetical protein
FUN_008806	contig_15	329274	330255	-		hypothetical protein
FUN_008807	contig_15	331532	334144	-		hypothetical protein
FUN_008808	contig_15	334417	336274	+		hypothetical protein
FUN_008810	contig_15	338452	340637	-		hypothetical protein
FUN_008811	contig_15	342182	344204	+		hypothetical protein
FUN_008812	contig_15	344490	347844	+		hypothetical protein
FUN_008814	contig_15	350847	352184	-		hypothetical protein
FUN_008817	contig_15	401315	402693	+		hypothetical protein
FUN_008818	contig_15	402973	404601	-	TXNDC17	Thioredoxin domain-containing protein 17
FUN_008820	contig_15	426826	428000	+		hypothetical protein
FUN_008823	contig_15	435130	436062	-	RHB1_1	GTP-binding protein
FUN_008825	contig_15	437475	438963	-		hypothetical protein
FUN_008827	contig_15	440969	443104	-		hypothetical protein
FUN_008828	contig_15	444792	446488	-		hypothetical protein
FUN_008829	contig_15	448285	449564	+		hypothetical protein

Gene ID	Scaffold	Start	Stop	Strand	Name	Product
FUN_008830	contig_15	449990	450808	+		hypothetical protein
FUN_008831	contig_15	452311	454535	+	MCM5	minichromosome maintenance protein 5
FUN_008832	contig_15	455437	457343	-		hypothetical protein
FUN_008833	contig_15	465325	467089	+		hypothetical protein
FUN_008835	contig_15	469086	471137	-		hypothetical protein
FUN_008836	contig_15	473405	475417	+		hypothetical protein
FUN_008837	contig_15	475533	476903	-		hypothetical protein
FUN_008838	contig_15	478327	479589	-		hypothetical protein
FUN_008839	contig_15	480043	481059	+		hypothetical protein
FUN_008840	contig_15	481643	482497	+		hypothetical protein
FUN_008845	contig_15	486801	488095	-	FAL1	RNA helicase
FUN_008846	contig_15	488464	490006	+	sap61	Pre-mRNA-splicing factor sap61
FUN_008847	contig_15	490364	491584	-		hypothetical protein
FUN_008849	contig_15	503488	505125	+		hypothetical protein
FUN_008850	contig_15	507344	508058	+		hypothetical protein
FUN_008852	contig_15	510030	510818	-		hypothetical protein
FUN_008853	contig_15	511528	513380	-		hypothetical protein
FUN_008855	contig_15	516996	519031	-	MET12	methylenetetrahydrofolate reductase 1
FUN_008856	contig_15	519362	520426	+		hypothetical protein
FUN_008857	contig_15	520471	522283	-	DLD2	D-lactate ferricytochrome c oxidoreductase
FUN_008858	contig_15	522551	523515	+	PRE6	Proteasome subunit alpha type-4
FUN_008859	contig_15	523696	524377	-		hypothetical protein
FUN_008860	contig_15	524936	528670	+		hypothetical protein
FUN_008862	contig_15	539468	545431	-		hypothetical protein
FUN_008863	contig_15	547009	549904	+		hypothetical protein
FUN_008864	contig_15	550911	553239	+	GSY1	glycogen synthase isoform 1
FUN_008865	contig_15	554422	557047	+	TRK1	low affinity potassium transporter
FUN_008866	contig_15	558214	560189	+		hypothetical protein
FUN_008867	contig_15	560438	562311	-		hypothetical protein
FUN_008868	contig_15	563119	565046	+		hypothetical protein
FUN_008869	contig_15	567873	569157	+		hypothetical protein
FUN_008870	contig_15	576097	577905	+		hypothetical protein
FUN_008871	contig_15	579532	581967	+		hypothetical protein
FUN_008873	contig_15	585160	586810	-	ACT1_2	actin
FUN_008874	contig_15	587367	589583	-		hypothetical protein
FUN_008877	contig_15	595103	596978	+		hypothetical protein
FUN_008878	contig_15	597137	598825	-		hypothetical protein
FUN_008879	contig_15	600960	601386	+		hypothetical protein
FUN_008881	contig_15	606494	608102	-	SPO11	endodeoxyribonuclease
FUN_008882	contig_15	608660	610572	+	RLF2	chromatin assembly factor-I (CAF-I) p90 subunit
FUN_008883	contig_15	612844	614335	+		hypothetical protein
FUN_008887	contig_15	622727	623395	+		hypothetical protein
FUN_008889	contig_15	625767	627152	+		hypothetical protein
FUN_008890	contig_15	632229	633333	+	RBK1	Putative ribokinase
FUN_008893	contig_15	640196	642033	+		hypothetical protein
FUN_008895	contig_15	648660	651998	-	UBP15	ubiquitin-specific protease ubp15
FUN_008896	contig_15	652677	656402	+	PKP2	[Pyruvate dehydrogenase (acetyl-transferring)] kinase 2, mitochondrial
FUN_008897	contig_15	657298	657706	+		hypothetical protein
FUN_008898	contig_15	658450	660210	-		hypothetical protein
FUN_008899	contig_15	661394	663394	-		hypothetical protein
FUN_008900	contig_15	664980	665789	-		hypothetical protein
FUN_008901	contig_15	666314	667495	-		hypothetical protein
FUN_008902	contig_15	668910	671021	-		hypothetical protein
FUN_008904	contig_15	673698	674808	+		hypothetical protein
FUN_008906	contig_15	677815	682524	-	RTS1	serine/threonine-protein phosphatase 2A 56 kDa regulatory subunit delta isoform
FUN_008907	contig_15	683269	685360	+	TFC1	tau 95 subunit of transcription factor TFIIIC
FUN_008908	contig_15	685670	689776	+	ADE6	phosphoribosylformylglycinamidine synthase

Gene ID	Scaffold	Start	Stop	Strand	Name	Product
FUN_008909	contig_15	691641	703181	+	TRA1	transcription-associated protein 1
FUN_008911	contig_15	711222	713141	-	MET7_3	Folylpolyglutamate synthetase
FUN_008912	contig_15	713800	718670	+		hypothetical protein
FUN_008913	contig_15	719035	719823	-	ndk1	nucleoside diphosphate kinase Ndk1
FUN_008914	contig_15	720053	723143	+	ROT2	glucosidase II
FUN_008917	contig_15	726158	728300	-		hypothetical protein
FUN_008918	contig_15	728900	730357	+		hypothetical protein
FUN_008919	contig_15	730822	732121	-		hypothetical protein
FUN_008920	contig_15	736798	737538	+		hypothetical protein
FUN_008922	contig_15	739521	741110	+		hypothetical protein
FUN_008925	contig_15	745183	747082	+		hypothetical protein
FUN_008930	contig_15	756364	760814	-		hypothetical protein
FUN_008931	contig_15	761541	762634	-		hypothetical protein
FUN_008932	contig_15	762834	763806	+		hypothetical protein
FUN_008933	contig_15	764214	765014	+	CAB5	Dephospho-CoA kinase cab5
FUN_008938	contig_15	776426	777346	-		hypothetical protein
FUN_008940	contig_15	779921	780847	-		hypothetical protein
FUN_008941	contig_15	781401	782889	+		hypothetical protein
FUN_008944	contig_15	789366	790461	-		hypothetical protein
FUN_008945	contig_15	791360	792195	+		hypothetical protein
FUN_008947	contig_15	793493	795015	-		hypothetical protein
FUN_008949	contig_15	796103	797417	-		hypothetical protein
FUN_008950	contig_15	797832	799577	-		hypothetical protein
FUN_008951	contig_15	800022	802010	-		hypothetical protein
FUN_008952	contig_15	803787	804717	+		hypothetical protein
FUN_008953	contig_15	804955	806786	-		hypothetical protein
FUN_008954	contig_15	807135	807686	-		hypothetical protein
FUN_008955	contig_15	808313	809642	+		hypothetical protein
FUN_008956	contig_15	810027	811307	+		hypothetical protein
FUN_008964	contig_15	826644	828619	+	CNA1	3',5'-cyclic-nucleotide phosphodiesterase (PDEase) (3':5'-CNP)
FUN_008966	contig_15	832144	836118	-		hypothetical protein
FUN_008968	contig_15	838867	839749	+	SPC3	Signal peptidase complex subunit
FUN_008969	contig_15	841962	844429	+		hypothetical protein
FUN_008970	contig_15	845354	845969	+		hypothetical protein
FUN_008972	contig_15	848370	849543	-		hypothetical protein
FUN_008973	contig_15	850115	853025	-	SWA2	auxilin-like clathrin-binding protein required for normal clathrin function
FUN_008974	contig_15	855440	857067	+		hypothetical protein
FUN_008975	contig_15	857765	859316	+	GTR1	GTP-binding protein gtr1
FUN_008976	contig_15	861572	864478	-		hypothetical protein
FUN_008977	contig_15	866749	867785	+		hypothetical protein
FUN_008978	contig_15	868155	868841	-	YAR1	ankyrin repeat-containing protein
FUN_008980	contig_15	876968	881278	-	exo2	exonuclease II Exo2
FUN_008981	contig_15	881649	883393	+	HAT2	Histone acetyltransferase type B subunit 2
FUN_008982	contig_15	883692	884549	+		hypothetical protein
FUN_008983	contig_15	888024	890850	-		hypothetical protein
FUN_008985	contig_15	897030	899488	-	LSK1	serine/threonine protein kinase, CMGC, CDC2/CDK sub
FUN_008986	contig_15	902747	903809	+	GCH1	GTP cyclohydrolase 1
FUN_008991	contig_15	913943	915174	+		hypothetical protein
FUN_008993	contig_15	917242	919116	-		hypothetical protein
FUN_008994	contig_15	921406	922857	+	taf6	histone H4-like TAF Taf6, SAGA complex subunit
FUN_008996	contig_15	925209	929784	-	SNQ2_2	ATP-binding cassette transporter snq2
FUN_008997	contig_15	933735	935534	-	OPI1	transcriptional regulator opi1
FUN_008998	contig_15	937159	938997	-		hypothetical protein
FUN_008999	contig_15	939254	940773	-		hypothetical protein
FUN_009000	contig_15	942179	943018	+		hypothetical protein
FUN_009001	contig_15	943443	949303	-		hypothetical protein
FUN_009002	contig_15	954829	955773	+		hypothetical protein

Gene ID	Scaffold	Start	Stop	Strand	Name	Product
FUN_009003	contig_15	955872	956822	-		hypothetical protein
FUN_009004	contig_15	957450	958543	-	ERG20	Farnesyl pyrophosphate synthetase
FUN_009005	contig_15	958766	960795	+	cut23	Anaphase-promoting complex subunit 8
FUN_009006	contig_15	961054	962142	+		hypothetical protein
FUN_009007	contig_15	965950	967558	+		hypothetical protein
FUN_009009	contig_15	977328	981989	-	RAD9	radiation sensitive protein rad9
FUN_009010	contig_15	983385	984003	-		hypothetical protein
FUN_009011	contig_15	984676	986019	+		hypothetical protein
FUN_009012	contig_15	986479	987434	+		hypothetical protein
FUN_009013	contig_15	990035	992070	-		hypothetical protein
FUN_009014	contig_15	992890	994833	-		hypothetical protein
FUN_009016	contig_15	997045	998502	-		hypothetical protein
FUN_009018	contig_15	1000469	1003245	-		hypothetical protein
FUN_009019	contig_15	1004636	1006639	-		hypothetical protein
FUN_009020	contig_15	1009761	1011286	+	CEL1_2	Esterase/lipase/thioesterase
FUN_009021	contig_15	1012476	1014151	+	CFD1_1	cytosolic Fe-S cluster assembly factor cfd1
FUN_009022	contig_15	1015194	1018202	-	CFD1_2	cytosolic Fe-S cluster assembly factor cfd1
FUN_009023	contig_15	1018672	1020337	-		hypothetical protein
FUN_009024	contig_15	1020758	1022705	+		hypothetical protein
FUN_009025	contig_15	1023733	1027689	+		hypothetical protein
FUN_009026	contig_15	1028632	1029480	+		hypothetical protein
FUN_009027	contig_15	1029942	1032397	+		hypothetical protein
FUN_009028	contig_15	1032816	1035018	+		hypothetical protein
FUN_009030	contig_15	1043022	1044404	-	PPX1	Exopolyphosphatase
FUN_009031	contig_15	1044895	1045585	-		hypothetical protein
FUN_009032	contig_15	1046253	1046831	+		hypothetical protein
FUN_009033	contig_15	1047255	1050756	+	SYG1	Xenotropic and polytropic retrovirus receptor 1
FUN_009035	contig_15	1056466	1059414	+		hypothetical protein
FUN_009037	contig_15	1068370	1071126	+		hypothetical protein
FUN_009038	contig_16	1208	2318	-		hypothetical protein
FUN_009040	contig_16	6128	6465	-		hypothetical protein
FUN_009041	contig_16	6819	7637	-		hypothetical protein
FUN_009042	contig_16	8001	8760	+		hypothetical protein
FUN_009043	contig_16	11211	11593	+		hypothetical protein
FUN_009044	contig_16	14878	16659	-		hypothetical protein
FUN_009045	contig_16	17835	18671	-		hypothetical protein
FUN_009047	contig_16	28337	30159	-		hypothetical protein
FUN_009049	contig_16	32457	34143	+		hypothetical protein
FUN_009050	contig_16	34283	36359	-	FAT1_2	long-chain fatty acid transporter fat1
FUN_009052	contig_16	42170	43248	-	sed5	Integral membrane protein SED5
FUN_009055	contig_16	56185	58602	+		hypothetical protein
FUN_009056	contig_16	59368	60391	-		hypothetical protein
FUN_009058	contig_16	65200	67627	-		hypothetical protein
FUN_009060	contig_16	72923	74696	-		hypothetical protein
FUN_009061	contig_16	75390	76715	+		hypothetical protein
FUN_009064	contig_16	80900	81724	-		hypothetical protein
FUN_009066	contig_16	84283	86208	-	UGA2_2	succinate semialdehyde dehydrogenase NADP+ linked
FUN_009069	contig_16	89441	89849	-		hypothetical protein
FUN_009070	contig_16	90164	91126	-		hypothetical protein
FUN_009071	contig_16	91690	94193	+		hypothetical protein
FUN_009072	contig_16	98484	102067	+		hypothetical protein
FUN_009073	contig_16	102700	103817	+		hypothetical protein
FUN_009074	contig_16	105535	106836	+		hypothetical protein
FUN_009076	contig_16	111053	112164	-		hypothetical protein
FUN_009077	contig_16	112954	114764	-		hypothetical protein
FUN_009078	contig_16	115091	116405	-		hypothetical protein
FUN_009079	contig_16	116689	116884	-		hypothetical protein
FUN_009080	contig_16	119302	121290	-	CDH1	substrate-specific activator of APC-dependent proteolysis

Gene ID	Scaffold	Start	Stop	Strand	Name	Product
FUN_009081	contig_16	122174	124048	+		hypothetical protein
FUN_009082	contig_16	124297	125460	+	PHO13	p-nitrophenyl phosphatase
FUN_009083	contig_16	125744	127352	+	PRI2	DNA primase subunit pri2
FUN_009084	contig_16	130022	132514	-		hypothetical protein
FUN_009085	contig_16	133744	134794	+	AQY1_3	Aquaporin-1
FUN_009086	contig_16	136596	137636	-		hypothetical protein
FUN_009089	contig_16	142000	144425	+		hypothetical protein
FUN_009090	contig_16	152600	153505	-	MED8	mediator of RNA polymerase II transcription subunit 8
FUN_009092	contig_16	159039	162224	-	SPT5	transcription elongation factor spt5
FUN_009093	contig_16	165491	167101	-		hypothetical protein
FUN_009094	contig_16	168419	169566	-		hypothetical protein
FUN_009095	contig_16	169923	172151	+		hypothetical protein
FUN_009096	contig_16	173443	174731	-		hypothetical protein
FUN_009097	contig_16	177176	179316	-	pzh1	serine/threonine protein phosphatase Pzh1
FUN_009098	contig_16	181351	182689	-	ALD2	mitochondrial aldehyde dehydrogenase
FUN_009099	contig_16	183374	184093	+		hypothetical protein
FUN_009101	contig_16	185638	187498	-		hypothetical protein
FUN_009102	contig_16	190594	192379	-		hypothetical protein
FUN_009103	contig_16	192936	194960	-		hypothetical protein
FUN_009104	contig_16	199512	200495	+		hypothetical protein
FUN_009105	contig_16	201109	203452	-		hypothetical protein
FUN_009107	contig_16	211546	212945	+		hypothetical protein
FUN_009108	contig_16	213548	214687	-	CAB2	Phosphopantothenate--cysteine ligase cab2
FUN_009109	contig_16	215679	218352	+		hypothetical protein
FUN_009110	contig_16	218528	219319	-		hypothetical protein
FUN_009111	contig_16	220676	221587	+		hypothetical protein
FUN_009112	contig_16	223797	225122	+		hypothetical protein
FUN_009113	contig_16	225441	228422	-	PMT1	Dolichyl-phosphate-mannose--protein mannosyltransferase 1
FUN_009114	contig_16	229084	230640	+	PRP21	SF3a splicing factor complex subunit
FUN_009115	contig_16	231395	232065	+		hypothetical protein
FUN_009117	contig_16	235081	236440	+		hypothetical protein
FUN_009118	contig_16	239407	242548	-	PDE2	3',5'-cyclic-nucleotide phosphodiesterase
FUN_009119	contig_16	245044	246310	-		hypothetical protein
FUN_009120	contig_16	246865	248898	+		hypothetical protein
FUN_009121	contig_16	249072	250307	-		hypothetical protein
FUN_009122	contig_16	251161	251848	-		hypothetical protein
FUN_009123	contig_16	253298	254838	-		hypothetical protein
FUN_009124	contig_16	256494	258968	+		hypothetical protein
FUN_009126	contig_16	261134	262270	+		hypothetical protein
FUN_009128	contig_16	269824	273100	-	LOS1	pre-tRNA nuclear export protein
FUN_009129	contig_16	273886	274901	-		hypothetical protein
FUN_009130	contig_16	275397	276021	-	RPO26	subunit common to RNA polymerases I, II, and III
FUN_009131	contig_16	276319	278113	+		hypothetical protein
FUN_009132	contig_16	278642	281284	-		hypothetical protein
FUN_009133	contig_16	281534	283310	+	QRI7	Mitochondrial tRNAs modification protein
FUN_009135	contig_16	287721	289555	-		hypothetical protein
FUN_009137	contig_16	292399	293409	+		hypothetical protein
FUN_009138	contig_16	293828	297064	+		hypothetical protein
FUN_009139	contig_16	298901	301240	-		hypothetical protein
FUN_009140	contig_16	306642	308325	-		hypothetical protein
FUN_009141	contig_16	308689	312907	-	HST6	ATP-dependent permease
FUN_009142	contig_16	313465	314092	-		hypothetical protein
FUN_009143	contig_16	314274	317951	+	WDR6	WD repeat-containing protein 6
FUN_009144	contig_16	318068	320566	+	POB3	FACT complex subunit
FUN_009145	contig_16	321532	323680	+		hypothetical protein
FUN_009146	contig_16	328857	329896	+		hypothetical protein
FUN_009147	contig_16	334275	335539	-		hypothetical protein
FUN_009148	contig_16	340868	343645	+	UTP4	U3 small nucleolar RNA-associated protein

Gene ID	Scaffold	Start	Stop	Strand	Name	Product
FUN_009149	contig_16	345250	346578	+	png1_1	Protein png1
FUN_009150	contig_16	348337	349407	+		hypothetical protein
FUN_009151	contig_16	350759	351377	-		hypothetical protein
FUN_009152	contig_16	352155	354010	-	ERG4_2	C-24(28) sterol reductase
FUN_009153	contig_16	354578	355798	-		hypothetical protein
FUN_009155	contig_16	364064	365148	+		hypothetical protein
FUN_009156	contig_16	366456	369114	-	RGD1	Rho GTPase-activating protein
FUN_009157	contig_16	369493	370360	+		hypothetical protein
FUN_009158	contig_16	370980	371736	-		hypothetical protein
FUN_009160	contig_16	376159	380400	-	SRB8	RNA polymerase II mediator complex subunit
FUN_009161	contig_16	382908	383541	+	SUI1	Eukaryotic translation initiation factor eIF-1
FUN_009162	contig_16	386016	386997	-	RIM2	Pyrimidine nucleotide transporter, mitochondrial
FUN_009163	contig_16	387297	388148	+		hypothetical protein
FUN_009164	contig_16	388819	391261	+	apc5	APC5 protein
FUN_009165	contig_16	391345	391958	-		hypothetical protein
FUN_009166	contig_16	392228	393777	+	APN2	Class II abasic (AP) endonuclease
FUN_009168	contig_16	396185	397235	-	ADE1	Bifunctional purine biosynthetic protein ade1
FUN_009169	contig_16	397481	399137	-	MET1	uroporphyrin-III C-methyltransferase
FUN_009170	contig_16	399540	400548	-		hypothetical protein
FUN_009171	contig_16	401738	402767	+	SUR7	Eisosomes component
FUN_009172	contig_16	403361	406283	-		hypothetical protein
FUN_009173	contig_16	407094	409390	-		hypothetical protein
FUN_009174	contig_16	412008	413814	+		hypothetical protein
FUN_009176	contig_16	415917	418806	-	TGL5	Lipase 5
FUN_009177	contig_16	420281	421248	+		hypothetical protein
FUN_009178	contig_16	421817	424802	+		hypothetical protein
FUN_009179	contig_16	425338	427594	-		hypothetical protein
FUN_009180	contig_16	435086	437688	+		hypothetical protein
FUN_009182	contig_16	440812	441814	-		hypothetical protein
FUN_009183	contig_16	442140	443454	+	GPI14	GPI mannosyltransferase 1
FUN_009184	contig_16	448948	449723	+		hypothetical protein
FUN_009185	contig_16	449806	451595	-		hypothetical protein
FUN_009186	contig_16	452943	454333	-		hypothetical protein
FUN_009187	contig_16	455237	455573	+		hypothetical protein
FUN_009188	contig_16	456190	458352	-		hypothetical protein
FUN_009189	contig_16	459194	460711	+		hypothetical protein
FUN_009190	contig_16	461398	462657	+		hypothetical protein
FUN_009191	contig_16	463069	464187	-		hypothetical protein
FUN_009192	contig_16	465302	469150	-		hypothetical protein
FUN_009193	contig_16	471336	472291	-		hypothetical protein
FUN_009194	contig_16	472657	476026	-		hypothetical protein
FUN_009195	contig_16	476927	478360	-	SLU7	mRNA splicing protein
FUN_009196	contig_16	478714	483844	+	REV3	DNA polymerase zeta
FUN_009197	contig_16	485238	486132	-		hypothetical protein
FUN_009198	contig_16	488607	489817	-		hypothetical protein
FUN_009199	contig_16	491168	494169	+	ADP1	FAD-dependent urate hydroxylase
FUN_009200	contig_16	497041	498435	-		hypothetical protein
FUN_009201	contig_16	500630	502067	+		hypothetical protein
FUN_009202	contig_16	502466	503164	-		hypothetical protein
FUN_009203	contig_16	503762	505717	+		hypothetical protein
FUN_009205	contig_16	509010	510869	+		hypothetical protein
FUN_009207	contig_16	513772	515758	-		hypothetical protein
FUN_009209	contig_16	519090	519874	-		hypothetical protein
FUN_009210	contig_16	520267	521139	+	SHY1	surf-like protein
FUN_009211	contig_16	521179	523005	-		hypothetical protein
FUN_009212	contig_16	523195	524123	+	cms1	Protein cms1
FUN_009213	contig_16	525505	526377	+	RAC1	Rho GTPase protein rac1
FUN_009214	contig_16	527028	528635	+		hypothetical protein
FUN_009215	contig_16	529112	532015	+		hypothetical protein

Gene ID	Scaffold	Start	Stop	Strand	Name	Product
FUN_009216	contig_16	534205	535125	-		hypothetical protein
FUN_009217	contig_16	535665	536996	-		hypothetical protein
FUN_009218	contig_16	537801	541076	+	ago1_1	Protein argonaute
FUN_009219	contig_16	543444	545137	+		hypothetical protein
FUN_009220	contig_16	545412	547197	-		hypothetical protein
FUN_009222	contig_16	552468	554982	+	CAC2	Chromatin assembly factor 1 subunit
FUN_009223	contig_16	555948	557804	+	FGR2	Filamentous Growth Regulator
FUN_009224	contig_16	558058	561443	-		hypothetical protein
FUN_009225	contig_16	561875	563164	-		hypothetical protein
FUN_009227	contig_16	566382	567325	-		hypothetical protein
FUN_009228	contig_16	568045	569777	-		hypothetical protein
FUN_009229	contig_16	570156	571894	-		hypothetical protein
FUN_009231	contig_16	577628	579204	+	CHT4_1	Chitinase 4
FUN_009233	contig_16	582857	585282	-		hypothetical protein
FUN_009236	contig_16	590560	591457	-		hypothetical protein
FUN_009237	contig_16	591694	592401	+		hypothetical protein
FUN_009238	contig_16	593887	595522	+		hypothetical protein
FUN_009239	contig_16	595834	596868	-		hypothetical protein
FUN_009242	contig_16	605014	608387	-		hypothetical protein
FUN_009243	contig_16	608920	610576	+		hypothetical protein
FUN_009244	contig_16	611301	614979	-	KIP1	Kinesin-related motor protein
FUN_009245	contig_16	615591	617226	+		hypothetical protein
FUN_009246	contig_16	617626	620788	+	ISM1	isoleucine-tRNA ligase
FUN_009247	contig_16	621170	622536	-	GPI17_1	GPI transamidase component
FUN_009248	contig_16	623309	625084	-	GPI17_2	GPI transamidase component
FUN_009249	contig_16	626026	626815	+		hypothetical protein
FUN_009250	contig_16	627051	627650	-		hypothetical protein
FUN_009251	contig_16	628456	630518	-		hypothetical protein
FUN_009253	contig_16	633331	634277	-		hypothetical protein
FUN_009254	contig_16	634838	635467	+	YAE1	Essential protein Yae1, N terminal
FUN_009255	contig_16	635557	637477	-		hypothetical protein
FUN_009256	contig_16	637827	639861	+		hypothetical protein
FUN_009257	contig_16	640484	641551	+		hypothetical protein
FUN_009258	contig_16	641629	643391	-		hypothetical protein
FUN_009260	contig_16	647326	648990	-		hypothetical protein
FUN_009261	contig_16	649499	651011	+		hypothetical protein
FUN_009262	contig_16	651952	652938	+		hypothetical protein
FUN_009263	contig_16	654650	655231	-		hypothetical protein
FUN_009264	contig_16	656256	662824	-	ESP1	separin protein
FUN_009267	contig_16	666753	668191	-		hypothetical protein
FUN_009268	contig_16	668402	669911	+		hypothetical protein
FUN_009269	contig_16	670653	672322	+		hypothetical protein
FUN_009273	contig_16	680756	681433	-		hypothetical protein
FUN_009274	contig_16	681975	682654	-		hypothetical protein
FUN_009275	contig_16	684533	685648	+		hypothetical protein
FUN_009276	contig_16	685973	687310	-		hypothetical protein
FUN_009277	contig_16	687917	688816	+		hypothetical protein
FUN_009278	contig_16	689155	690198	+	TRIM7	E3 ubiquitin-protein ligase
FUN_009279	contig_16	690377	691647	-		hypothetical protein
FUN_009281	contig_16	694345	694911	+		hypothetical protein
FUN_009283	contig_16	698244	699250	-		hypothetical protein
FUN_009284	contig_16	702232	702921	+		hypothetical protein
FUN_009285	contig_16	707855	709208	-		hypothetical protein
FUN_009286	contig_16	714011	714705	-	RPL21A	60S ribosomal protein L21A
FUN_009287	contig_16	714889	715692	+	RPS9B	ribosomal 40S subunit protein S9B
FUN_009288	contig_16	716016	717383	-		hypothetical protein
FUN_009289	contig_16	719687	720532	-	RHO3	Rho GTPase
FUN_009290	contig_16	722558	727251	-		hypothetical protein
FUN_009291	contig_16	728013	728779	-		hypothetical protein

Gene ID	Scaffold	Start	Stop	Strand	Name	Product
FUN_009293	contig_16	731710	733043	-		hypothetical protein
FUN_009294	contig_16	733867	734575	+		hypothetical protein
FUN_009296	contig_16	738377	740355	+	vps5	Vacuolar protein sorting-associated protein vps5
FUN_009297	contig_16	740810	742467	-		hypothetical protein
FUN_009298	contig_16	744327	746670	+		hypothetical protein
FUN_009299	contig_16	747137	748183	+		hypothetical protein
FUN_009300	contig_16	749130	751061	+		hypothetical protein
FUN_009301	contig_16	751306	752165	-		hypothetical protein
FUN_009302	contig_16	754070	755163	+		hypothetical protein
FUN_009305	contig_16	758737	759791	-		hypothetical protein
FUN_009309	contig_16	770702	772277	-		hypothetical protein
FUN_009311	contig_16	776597	779508	-		hypothetical protein
FUN_009313	contig_16	781766	782287	+		hypothetical protein
FUN_009319	contig_16	798373	799260	-		hypothetical protein
FUN_009321	contig_16	805175	806155	+	APA2	bifunctional AP-4-A phosphorylase/ADP sulfurylase
FUN_009322	contig_16	809405	810625	+		hypothetical protein
FUN_009323	contig_16	812243	814420	+		hypothetical protein
FUN_009324	contig_16	815229	819465	+		hypothetical protein
FUN_009325	contig_16	823863	826052	-		hypothetical protein
FUN_009326	contig_16	826587	827925	+		hypothetical protein
FUN_009327	contig_16	828417	829634	-		hypothetical protein
FUN_009328	contig_16	831574	836870	+		hypothetical protein
FUN_009330	contig_16	838938	841629	-		hypothetical protein
FUN_009331	contig_16	843573	844687	-	SCO1	Cu-binding protein
FUN_009332	contig_16	845168	847073	-		hypothetical protein
FUN_009333	contig_16	847920	849725	-		hypothetical protein
FUN_009336	contig_16	862765	863641	-	ATP16	delta subunit of the central stalk of mitochondrial F1F0 ATP synthase, atp16
FUN_009337	contig_16	865708	867812	+		hypothetical protein
FUN_009340	contig_16	872307	873260	-		hypothetical protein
FUN_009341	contig_16	873713	875337	+	NPT1	nicotinate phosphoribosyltransferase
FUN_009342	contig_16	876108	877202	+		hypothetical protein
FUN_009343	contig_16	877865	881912	+		hypothetical protein
FUN_009346	contig_16	884432	885890	-		hypothetical protein
FUN_009347	contig_16	886541	887941	+		hypothetical protein
FUN_009348	contig_16	888136	889177	-		hypothetical protein
FUN_009349	contig_16	890356	891389	-	PHO85	negative regulator of the PHO system
FUN_009350	contig_16	892555	896042	+		hypothetical protein
FUN_009351	contig_16	897009	901557	+	CWC22	pre-mRNA-splicing factor cwc22
FUN_009354	contig_16	910342	913724	-		hypothetical protein
FUN_009356	contig_16	917280	918576	+	PDX1	pyridoxine biosynthesis protein
FUN_009357	contig_16	919712	921979	+	AGC1	mitochondrial aspartate-glutamate transporter agc1
FUN_009358	contig_16	923705	925160	+		hypothetical protein
FUN_009360	contig_16	931097	932080	+		hypothetical protein
FUN_009361	contig_16	932700	933230	+	SNX3	Sorting nexin-3
FUN_009362	contig_16	935125	936237	+		hypothetical protein
FUN_009363	contig_16	936888	937950	+	PLP2	Proteolipid protein 2
FUN_009364	contig_16	941917	942947	-		hypothetical protein
FUN_009366	contig_16	950896	953784	-		hypothetical protein
FUN_009367	contig_16	954428	956510	-	ERF2	Eukaryotic peptide chain release factor GTP-binding subunit
FUN_009370	contig_16	959259	960770	-		hypothetical protein
FUN_009371	contig_16	961588	962504	+	RPS2	40S ribosomal protein
FUN_009372	contig_16	962870	964628	-		hypothetical protein
FUN_009373	contig_16	966794	971731	+	YTA7	TAT-binding protein-like protein 7, AAA ATPase
FUN_009376	contig_16	976573	977515	-		hypothetical protein
FUN_009377	contig_16	978261	979049	+		hypothetical protein
FUN_009379	contig_16	980491	980969	-	APC11	ubiquitin-protein ligase Anaphase Promoting Complex
FUN_009380	contig_16	981562	982627	+		hypothetical protein

Gene ID	Scaffold	Start	Stop	Strand	Name	Product
FUN_009381	contig_16	983970	985309	-		hypothetical protein
FUN_009382	contig_16	988205	990307	-	HSP82	Hsp90 chaperone hsp82
FUN_009384	contig_16	992817	993730	+	LPMO9B	AA9 lytic polysaccharide monooxygenase B
FUN_009386	contig_16	995073	996848	-		hypothetical protein
FUN_009387	contig_16	997637	999020	+		hypothetical protein
FUN_009388	contig_16	1004877	1009223	+		hypothetical protein
FUN_009389	contig_16	1009267	1011314	-		hypothetical protein
FUN_009390	contig_16	1012765	1015288	+		hypothetical protein
FUN_009391	contig_16	1017095	1019023	+	PYK1	Pyruvate kinase
FUN_009392	contig_16	1019732	1021057	-		hypothetical protein
FUN_009393	contig_16	1022661	1026419	+	RPN2	proteasome regulatory particle base subunit
FUN_009394	contig_16	1026909	1028423	+	COX10	Protoheme IX farnesyltransferase, mitochondrial
FUN_009395	contig_16	1029519	1030409	+	LPMO9H	AA9 lytic polysaccharide monooxygenase H
FUN_009399	contig_16	1044751	1046768	-		hypothetical protein
FUN_009400	contig_16	1048762	1050606	-		hypothetical protein
FUN_009402	contig_16	1053401	1054261	-		hypothetical protein
FUN_009403	contig_16	1054557	1060997	+		hypothetical protein
FUN_009405	contig_17	10556	11468	-		hypothetical protein
FUN_009406	contig_17	12087	12820	-		hypothetical protein
FUN_009407	contig_17	14544	15964	+		hypothetical protein
FUN_009409	contig_17	42629	43574	+		hypothetical protein
FUN_009410	contig_17	44543	45480	-	GNAT2	Guanine nucleotide-binding protein G(t) subunit alpha-2
FUN_009411	contig_17	48318	49075	+		hypothetical protein
FUN_009412	contig_17	52366	53394	-		hypothetical protein
FUN_009413	contig_17	54305	56139	+		hypothetical protein
FUN_009414	contig_17	56715	57795	+		hypothetical protein
FUN_009415	contig_17	59178	60583	+	TLL1	Dorsal-ventral patterning tolloid-like protein 1
FUN_009416	contig_17	63270	67442	-		hypothetical protein
FUN_009417	contig_17	68018	69987	-		hypothetical protein
FUN_009418	contig_17	70660	71398	-		hypothetical protein
FUN_009419	contig_17	72218	73446	-		hypothetical protein
FUN_009420	contig_17	74169	75654	-		hypothetical protein
FUN_009421	contig_17	76464	79196	+		hypothetical protein
FUN_009422	contig_17	80487	81963	+	DOT1	Nucleosomal histone H3-Lys79 methylase
FUN_009424	contig_17	84859	85649	-		hypothetical protein
FUN_009425	contig_17	87090	87454	-	TIM9	protein transporter tim9
FUN_009426	contig_17	88548	91289	+	SAC7	GTPase activating protein (GAP) for Rho1p
FUN_009428	contig_17	95783	96734	+	TPI1	triosephosphate isomerase
FUN_009429	contig_17	97649	98841	+		hypothetical protein
FUN_009430	contig_17	100655	102932	+		hypothetical protein
FUN_009431	contig_17	103997	104872	-		hypothetical protein
FUN_009432	contig_17	105394	107195	+		hypothetical protein
FUN_009434	contig_17	112406	113932	+		hypothetical protein
FUN_009435	contig_17	117277	118032	+	GCV3	glycine cleavage system H-protein subunit
FUN_009436	contig_17	118180	127299	-	TEL1	Serine/threonine-protein kinase tel1
FUN_009437	contig_17	127656	132753	+		hypothetical protein
FUN_009438	contig_17	133453	134457	+		hypothetical protein
FUN_009441	contig_17	138736	139522	+	URE2_2	Transcriptional regulator ure2
FUN_009442	contig_17	141035	142978	-		hypothetical protein
FUN_009443	contig_17	143690	150843	-		hypothetical protein
FUN_009444	contig_17	152153	153304	+		hypothetical protein
FUN_009445	contig_17	153914	155741	+		hypothetical protein
FUN_009446	contig_17	156508	158188	+		hypothetical protein
FUN_009447	contig_17	158578	159497	-		hypothetical protein
FUN_009449	contig_17	162830	167971	-	DNF3	drs2 neo1 protein
FUN_009450	contig_17	169136	170977	-		hypothetical protein
FUN_009451	contig_17	172350	174467	+		hypothetical protein
FUN_009453	contig_17	182522	184371	-	GDA1	Guanosine-diphosphatase
FUN_009455	contig_17	186442	187195	-		hypothetical protein

Gene ID	Scaffold	Start	Stop	Strand	Name	Product
FUN_009456	contig_17	194399	195250	-		hypothetical protein
FUN_009457	contig_17	197949	199092	-	STE4	G protein subunit beta
FUN_009458	contig_17	199927	201457	+		hypothetical protein
FUN_009461	contig_17	206987	208901	+		hypothetical protein
FUN_009462	contig_17	210991	213252	+		hypothetical protein
FUN_009463	contig_17	219942	227196	-		hypothetical protein
FUN_009465	contig_17	230032	231610	+	AIM6_2	Altered inheritance of mitochondria protein 6
FUN_009466	contig_17	232018	234792	-		hypothetical protein
FUN_009467	contig_17	235350	238286	-	msp1	mitochondrial dynamin GTPase Msp1
FUN_009468	contig_17	238645	239661	+		hypothetical protein
FUN_009470	contig_17	243867	244658	+		hypothetical protein
FUN_009471	contig_17	249535	253152	-	IRR1	cohesin complex subunit
FUN_009472	contig_17	253480	254508	-		hypothetical protein
FUN_009473	contig_17	255213	256071	-		hypothetical protein
FUN_009474	contig_17	256914	258214	-	DUG3	glutamine amidotransferase subunit
FUN_009475	contig_17	259246	261066	-	cut15	Importin subunit alpha-1
FUN_009476	contig_17	261390	262926	+		hypothetical protein
FUN_009477	contig_17	263634	264425	+		hypothetical protein
FUN_009479	contig_17	270883	273079	-	POL12	DNA-directed DNA polymerase alpha subunit pol12
FUN_009480	contig_17	273504	274748	+	PDR16	Phosphatidylinositol transfer protein (PITP)
FUN_009481	contig_17	275283	277436	-		hypothetical protein
FUN_009482	contig_17	278812	280070	+		hypothetical protein
FUN_009483	contig_17	280464	281475	-		hypothetical protein
FUN_009484	contig_17	282270	283109	+		hypothetical protein
FUN_009485	contig_17	283324	285559	-		hypothetical protein
FUN_009486	contig_17	285960	287582	+		hypothetical protein
FUN_009487	contig_17	291267	292330	+		hypothetical protein
FUN_009488	contig_17	293076	295441	+		hypothetical protein
FUN_009489	contig_17	297859	298618	-	MRT4	mRNA turnover and ribosome assembly protein
FUN_009490	contig_17	298850	300132	+	MDM10	Mitochondrial distribution and morphology protein 10
FUN_009491	contig_17	302190	304056	+		hypothetical protein
FUN_009492	contig_17	306234	308237	+	LCB2	serine palmitoyltransferase component
FUN_009495	contig_17	318768	320932	-		hypothetical protein
FUN_009497	contig_17	324353	325187	-	RRP46	exosome non-catalytic core subunit rrp46
FUN_009498	contig_17	325429	326009	+		hypothetical protein
FUN_009500	contig_17	331022	332817	-		hypothetical protein
FUN_009501	contig_17	333509	337012	-	PSD2	phosphatidylserine decarboxylase
FUN_009502	contig_17	338590	339715	-		hypothetical protein
FUN_009503	contig_17	341753	343011	-	GPA1	guanine nucleotide-binding protein subunit alpha
FUN_009505	contig_17	350353	352972	+	TKL1	Transketolase
FUN_009507	contig_17	355695	357396	-	INO1	Myo-inositol-1-phosphate synthase
FUN_009508	contig_17	360176	362179	+		hypothetical protein
FUN_009509	contig_17	363392	364232	-		hypothetical protein
FUN_009510	contig_17	365355	367276	+		hypothetical protein
FUN_009512	contig_17	368487	369107	-	CSL4	exosome 3'->5 exonuclease subunit ski4 (Csl4)
FUN_009513	contig_17	369628	370778	-	TEM1	Ras GTPase tem1
FUN_009514	contig_17	371238	372811	+	rrb1	Ribosome assembly protein rrb1
FUN_009516	contig_17	377192	378133	-	GPI12	N-acetylglucosaminyl-phosphatidylinositol de-N-acetylase
FUN_009518	contig_17	379117	380404	-		hypothetical protein
FUN_009520	contig_17	382636	384582	+		hypothetical protein
FUN_009521	contig_17	385375	385980	+		hypothetical protein
FUN_009522	contig_17	386594	387512	-		hypothetical protein
FUN_009523	contig_17	388848	389458	-		hypothetical protein
FUN_009524	contig_17	393342	394528	+	SSU1	Plasma membrane sulfite pump involved in sulfite metabolism
FUN_009525	contig_17	395362	398019	+	CDC48	AAA ATPase cdc48
FUN_009527	contig_17	400340	402007	+		hypothetical protein
FUN_009528	contig_17	408221	410359	+		hypothetical protein

Gene ID	Scaffold	Start	Stop	Strand	Name	Product
FUN_009530	contig_17	412975	414692	+	TIF3	Eukaryotic translation initiation factor 4B
FUN_009532	contig_17	417606	418448	+		hypothetical protein
FUN_009533	contig_17	419083	420324	+		hypothetical protein
FUN_009534	contig_17	420608	421852	+		hypothetical protein
FUN_009535	contig_17	422372	423007	+		hypothetical protein
FUN_009536	contig_17	424282	427204	+		hypothetical protein
FUN_009537	contig_17	428315	433769	-	RRP5	rRNA bioproteinsis protein rrp5
FUN_009538	contig_17	434050	436131	+		hypothetical protein
FUN_009539	contig_17	443900	446652	+		hypothetical protein
FUN_009541	contig_17	451813	452749	-	PHB1	Prohibitin-1, subunit of the prohibitin complex (Phb1p-Phb2p)
FUN_009542	contig_17	453596	454636	-		hypothetical protein
FUN_009543	contig_17	455250	456270	-		hypothetical protein
FUN_009544	contig_17	456522	457546	-		hypothetical protein
FUN_009545	contig_17	458649	459371	+		hypothetical protein
FUN_009546	contig_17	459574	460286	-		hypothetical protein
FUN_009547	contig_17	460640	462340	-		hypothetical protein
FUN_009548	contig_17	464128	469154	+		hypothetical protein
FUN_009549	contig_17	469751	471071	-		hypothetical protein
FUN_009550	contig_17	471845	475228	+	RIA1	Cytoplasmic GTPase/eEF2-like protein (ribosomal bioproteinsis)
FUN_009551	contig_17	476263	478367	+		hypothetical protein
FUN_009552	contig_17	479402	481295	-		hypothetical protein
FUN_009553	contig_17	481426	482396	-	HNT3	aprataxin-like protein
FUN_009554	contig_17	482713	487036	+	ucp12	Putative ATP-dependent RNA helicase ucp12
FUN_009555	contig_17	488934	490852	-		hypothetical protein
FUN_009556	contig_17	491606	492530	+	IRC22	Increased recombination centers protein 22
FUN_009559	contig_17	497933	500548	+		hypothetical protein
FUN_009560	contig_17	501228	502472	+		hypothetical protein
FUN_009561	contig_17	502776	504743	+		hypothetical protein
FUN_009564	contig_17	509287	510317	-		hypothetical protein
FUN_009565	contig_17	516591	518305	+		hypothetical protein
FUN_009566	contig_17	521510	522854	+		hypothetical protein
FUN_009567	contig_17	524250	525314	+	SUA7_2	transcription initiation factor IIB
FUN_009568	contig_17	525754	527826	-		hypothetical protein
FUN_009569	contig_17	529394	530582	+		hypothetical protein
FUN_009570	contig_17	530841	531884	-		hypothetical protein
FUN_009571	contig_17	532161	533162	-		hypothetical protein
FUN_009572	contig_17	533462	535078	-		hypothetical protein
FUN_009573	contig_17	535825	538447	-		hypothetical protein
FUN_009574	contig_17	539601	541586	+		hypothetical protein
FUN_009575	contig_17	542383	543230	-		hypothetical protein
FUN_009576	contig_17	543836	545971	-		hypothetical protein
FUN_009577	contig_17	547588	548036	+		hypothetical protein
FUN_009579	contig_17	549175	550693	+		hypothetical protein
FUN_009580	contig_17	551245	553278	+		hypothetical protein
FUN_009581	contig_17	553978	555650	+		hypothetical protein
FUN_009582	contig_17	556036	562615	-	ATG2	autophagy-related protein 2
FUN_009583	contig_17	563217	564988	+		hypothetical protein
FUN_009587	contig_17	572871	575018	-		hypothetical protein
FUN_009588	contig_17	576137	577506	+		hypothetical protein
FUN_009589	contig_17	577884	579186	-		hypothetical protein
FUN_009591	contig_17	584671	588038	-		hypothetical protein
FUN_009592	contig_17	589804	591604	-	aif1	Apoptosis-inducing factor 1
FUN_009593	contig_17	592501	594502	+		hypothetical protein
FUN_009595	contig_17	596556	597275	-		hypothetical protein
FUN_009596	contig_17	598785	600968	-		hypothetical protein
FUN_009600	contig_17	608734	609349	+	NAS2	putative 26S proteasome regulatory subunit
FUN_009601	contig_17	609950	610873	+	GSP1	GTP-binding nuclear protein gsp1/Ran

Gene ID	Scaffold	Start	Stop	Strand	Name	Product
FUN_009602	contig_17	611805	614053	-		hypothetical protein
FUN_009603	contig_17	616127	617520	-		hypothetical protein
FUN_009604	contig_17	618322	619339	+		hypothetical protein
FUN_009605	contig_17	623122	624306	-		hypothetical protein
FUN_009606	contig_17	626728	628494	-		hypothetical protein
FUN_009607	contig_17	630077	639352	+		hypothetical protein
FUN_009608	contig_17	639570	640374	-		hypothetical protein
FUN_009609	contig_17	640742	642582	-		hypothetical protein
FUN_009611	contig_17	646924	648315	-		hypothetical protein
FUN_009612	contig_17	650663	653885	+		hypothetical protein
FUN_009613	contig_17	655302	656332	-	BMH1	14-3-3 protein
FUN_009614	contig_17	657780	658190	+	INH1	ATPase inhibitor
FUN_009615	contig_17	658518	660918	-	NSP1	FG-nucleoporin nsp1
FUN_009616	contig_17	661416	663110	+		hypothetical protein
FUN_009617	contig_17	664581	668570	+	NIK1	histidine kinase osmosensor
FUN_009618	contig_17	669856	670566	-		hypothetical protein
FUN_009619	contig_17	670934	672865	-		hypothetical protein
FUN_009620	contig_17	676474	677223	+		hypothetical protein
FUN_009622	contig_17	683096	686645	+		hypothetical protein
FUN_009623	contig_17	687404	688163	-	SDH4	membrane anchor subunit of succinate dehydrogenase, Sdh4
FUN_009624	contig_17	688434	690097	+	MAS1	Mitochondrial-processing peptidase subunit beta
FUN_009625	contig_17	690365	692183	-		hypothetical protein
FUN_009626	contig_17	692665	693410	-	YET3	Endoplasmic reticulum transmembrane protein 3
FUN_009627	contig_17	693975	696246	+	DUS3	tRNA-dihydrouridine synthase 3
FUN_009628	contig_17	697005	700547	-		hypothetical protein
FUN_009629	contig_17	701633	703170	-		hypothetical protein
FUN_009631	contig_17	704832	705936	-		hypothetical protein
FUN_009632	contig_17	708111	711272	-	SFP1	Transcription factor sfp1
FUN_009633	contig_17	715817	717373	+	TOA1	transcription factor IIA subunit alpha
FUN_009634	contig_17	719207	720650	-	GCN5	histone acetyltransferase
FUN_009635	contig_17	721420	722582	+	rad1	checkpoint clamp complex protein Rad1
FUN_009636	contig_17	726860	732122	-	RPO21	DNA-directed RNA polymerase II core subunit rpo21
FUN_009637	contig_17	732775	734368	+	SWD1	chromatin binding protein
FUN_009638	contig_17	734765	737878	+		hypothetical protein
FUN_009639	contig_17	738493	739908	+		hypothetical protein
FUN_009641	contig_17	742119	743750	+		hypothetical protein
FUN_009644	contig_17	749406	751199	-		hypothetical protein
FUN_009645	contig_17	751895	753854	+		hypothetical protein
FUN_009647	contig_17	756310	757824	+		hypothetical protein
FUN_009651	contig_17	767139	768557	+		hypothetical protein
FUN_009652	contig_17	768861	770468	+		hypothetical protein
FUN_009653	contig_17	770799	771563	+		hypothetical protein
FUN_009654	contig_17	771991	774536	-		hypothetical protein
FUN_009655	contig_17	777493	778687	+		hypothetical protein
FUN_009656	contig_17	781255	785558	+		hypothetical protein
FUN_009658	contig_17	787827	789359	+		hypothetical protein
FUN_009660	contig_17	790905	791692	-		hypothetical protein
FUN_009663	contig_17	799869	800761	+	FGAQP3	putative aquaporin-3
FUN_009665	contig_17	803114	803910	-		hypothetical protein
FUN_009666	contig_17	805139	806008	-		hypothetical protein
FUN_009667	contig_17	808612	810893	-	gel4_2	1 3-beta-glucanosyltransferase gel4
FUN_009668	contig_17	812018	813180	+		hypothetical protein
FUN_009670	contig_17	814426	815491	-		hypothetical protein
FUN_009672	contig_17	818848	819978	+		hypothetical protein
FUN_009676	contig_17	829605	831775	-		hypothetical protein
FUN_009678	contig_17	834553	838268	+	SLN1	Histidine kinase osmosensor
FUN_009679	contig_17	838949	841110	+		hypothetical protein
FUN_009680	contig_17	842521	843521	-		hypothetical protein

Gene ID	Scaffold	Start	Stop	Strand	Name	Product
FUN_009681	contig_17	844241	845234	+		hypothetical protein
FUN_009684	contig_17	849434	851259	-		hypothetical protein
FUN_009685	contig_17	852822	854434	+		hypothetical protein
FUN_009687	contig_17	856897	857709	+		hypothetical protein
FUN_009688	contig_17	858249	859934	+		hypothetical protein
FUN_009690	contig_17	863014	863881	+		hypothetical protein
FUN_009691	contig_17	863992	864934	-		hypothetical protein
FUN_009693	contig_17	867238	873045	-	ned1	lipin Ned1
FUN_009694	contig_17	873690	878420	+		hypothetical protein
FUN_009695	contig_17	879125	880230	+		hypothetical protein
FUN_009696	contig_17	880338	882074	-		hypothetical protein
FUN_009697	contig_17	883348	885019	-		hypothetical protein
FUN_009698	contig_17	885571	891362	+		hypothetical protein
FUN_009699	contig_17	891413	892279	-	DHG2	L-rhamnose-1-dehydrogenase
FUN_009700	contig_17	892658	893839	-	LRA2	L-rhamnono-gamma-lactonase
FUN_009701	contig_17	898434	900079	-		hypothetical protein
FUN_009702	contig_17	906837	909095	+	GLR1_2	Glutathione reductase
FUN_009703	contig_17	909548	913112	+	ROK1	RNA-dependent ATPase rok1
FUN_009704	contig_17	913364	915629	-		hypothetical protein
FUN_009705	contig_17	916051	920984	-		hypothetical protein
FUN_009706	contig_17	922557	923566	+		hypothetical protein
FUN_009707	contig_17	925742	926710	-	thp1	uracil DNA N-glycosylase Thp1
FUN_009708	contig_17	927290	928862	-	wc2	white collar 2 type of transcription factor
FUN_009709	contig_17	929024	933604	+	RAD50	DNA repair protein rad50
FUN_009710	contig_17	935144	936748	+	EXG2_2	glucan exo-1,3-beta-glucosidase
FUN_009711	contig_17	938424	939467	+		hypothetical protein
FUN_009712	contig_17	942136	942976	-		hypothetical protein
FUN_009713	contig_17	944309	945232	+		hypothetical protein
FUN_009715	contig_17	951886	953475	+	cyp6	Peptidyl-prolyl cis-trans isomerase-like 4
FUN_009716	contig_17	955123	958944	-	RPB2	DNA-dependent RNA polymerase II
FUN_009717	contig_17	959649	960603	+		hypothetical protein
FUN_009718	contig_17	960866	961978	-		hypothetical protein
FUN_009719	contig_17	962500	964170	+		hypothetical protein
FUN_009720	contig_17	964687	967009	-		hypothetical protein
FUN_009721	contig_17	970118	973322	-		hypothetical protein
FUN_009722	contig_17	973346	974896	-		hypothetical protein
FUN_009723	contig_17	975438	976479	+		hypothetical protein
FUN_009724	contig_17	976549	978732	-		hypothetical protein
FUN_009725	contig_17	979018	983868	-		hypothetical protein
FUN_009726	contig_17	985105	987100	+		hypothetical protein
FUN_009727	contig_17	987883	989735	-		hypothetical protein
FUN_009728	contig_17	990554	992910	-		hypothetical protein
FUN_009729	contig_17	993471	995760	+	slp1	WD repeat-containing protein slp1
FUN_009730	contig_17	997796	998623	-		hypothetical protein
FUN_009731	contig_17	1025860	1027926	+		hypothetical protein
FUN_009733	contig_18	15888	18394	+		hypothetical protein
FUN_009734	contig_18	45970	47157	+		hypothetical protein
FUN_009735	contig_18	49748	50340	+		hypothetical protein
FUN_009736	contig_18	50668	57039	-		hypothetical protein
FUN_009737	contig_18	57699	59583	-		hypothetical protein
FUN_009738	contig_18	59918	63770	+		hypothetical protein
FUN_009741	contig_18	69566	71165	-		hypothetical protein
FUN_009742	contig_18	71892	75188	-		hypothetical protein
FUN_009743	contig_18	76036	77641	-		hypothetical protein
FUN_009744	contig_18	78474	80065	-		hypothetical protein
FUN_009745	contig_18	80772	81578	-		hypothetical protein
FUN_009746	contig_18	83027	84406	-		hypothetical protein
FUN_009749	contig_18	89814	90513	+		hypothetical protein
FUN_009752	contig_18	103066	106146	+	srs2	ATP-dependent DNA helicase srs2

Gene ID	Scaffold	Start	Stop	Strand	Name	Product
FUN_009755	contig_18	109796	110974	+		hypothetical protein
FUN_009756	contig_18	111464	113376	-		hypothetical protein
FUN_009757	contig_18	116714	117808	+	mug86	Meiotically up-regulated protein 86 protein
FUN_009758	contig_18	118832	120731	-		hypothetical protein
FUN_009759	contig_18	121022	121494	-	uvi31	BolA domain UV induced protein Uvi31
FUN_009760	contig_18	122114	123949	+		hypothetical protein
FUN_009761	contig_18	130231	131616	-		hypothetical protein
FUN_009762	contig_18	132325	132996	-	ERV14	COPII-coated vesicle protein
FUN_009763	contig_18	133687	135040	-	fma1	Methionine aminopeptidase 1
FUN_009764	contig_18	135521	137096	+	BNA4	kynurenine 3-monooxygenase, mitochondrial precursor
FUN_009766	contig_18	143952	151178	+	MDM12_2	Mitochondrial distribution and morphology protein 12
FUN_009767	contig_18	152794	154422	+	ERG9	bifunctional farnesyl-diphosphate farnesyltransferase/squalene synthase
FUN_009768	contig_18	155095	155943	+	TIF45	eukaryotic translation initiation factor 4E
FUN_009769	contig_18	156607	158459	+	CAJ1	DnaJ-like protein
FUN_009770	contig_18	159035	160977	-	ILV1	threonine deaminase
FUN_009773	contig_18	165110	167268	+	NOG2	GTPase required for pre-60S ribosomal subunit nuclear export and maturation
FUN_009774	contig_18	167574	168548	+		hypothetical protein
FUN_009775	contig_18	168602	169863	-		hypothetical protein
FUN_009776	contig_18	170241	171850	+		hypothetical protein
FUN_009777	contig_18	172513	175227	-		hypothetical protein
FUN_009780	contig_18	179165	181448	-		hypothetical protein
FUN_009787	contig_18	201221	202855	-		hypothetical protein
FUN_009789	contig_18	206677	207849	-		hypothetical protein
FUN_009790	contig_18	208804	209946	+		hypothetical protein
FUN_009791	contig_18	210064	211262	-	LYS12	homoisocitrate dehydrogenase
FUN_009792	contig_18	211711	213255	-		hypothetical protein
FUN_009795	contig_18	217702	220525	-		hypothetical protein
FUN_009796	contig_18	221799	223096	+		hypothetical protein
FUN_009798	contig_18	231368	233725	-		hypothetical protein
FUN_009799	contig_18	234335	235828	+		hypothetical protein
FUN_009800	contig_18	236088	237080	+	GPI2	glycosylphosphatidylinositol anchor biosynthesis
FUN_009803	contig_18	239755	241919	+		hypothetical protein
FUN_009804	contig_18	244456	246313	-		hypothetical protein
FUN_009805	contig_18	246522	248480	-		hypothetical protein
FUN_009806	contig_18	249046	251165	+		hypothetical protein
FUN_009807	contig_18	252316	253378	+		hypothetical protein
FUN_009811	contig_18	259428	261027	-		hypothetical protein
FUN_009812	contig_18	261387	264724	-		hypothetical protein
FUN_009813	contig_18	266366	267286	-		hypothetical protein
FUN_009814	contig_18	267683	269818	+		hypothetical protein
FUN_009815	contig_18	270645	271636	+	TSA1	cTPxI
FUN_009816	contig_18	272236	273722	-	EPT1	Phosphotransferase
FUN_009817	contig_18	274165	275211	-		hypothetical protein
FUN_009818	contig_18	275856	276833	-		hypothetical protein
FUN_009819	contig_18	278186	278629	+		hypothetical protein
FUN_009820	contig_18	279233	280189	+		hypothetical protein
FUN_009821	contig_18	280426	282014	+		hypothetical protein
FUN_009823	contig_18	285437	286981	-		hypothetical protein
FUN_009824	contig_18	289748	290866	-		hypothetical protein
FUN_009825	contig_18	293935	295685	+		hypothetical protein
FUN_009826	contig_18	297107	298849	+		hypothetical protein
FUN_009827	contig_18	301780	303712	-	IPI3	Pre-rRNA-processing protein ipi3
FUN_009828	contig_18	304012	304860	+		hypothetical protein
FUN_009829	contig_18	305035	306715	-	RPN5	proteasome regulatory particle subunit
FUN_009830	contig_18	307793	312407	+	KEL2	Negative regulator of mitotic exit
FUN_009831	contig_18	313450	319220	+		hypothetical protein
FUN_009832	contig_18	319472	323356	+	RAD2	DNA repair protein rad2

Gene ID	Scaffold	Start	Stop	Strand	Name	Product
FUN_009833	contig_18	325090	328322	+		hypothetical protein
FUN_009834	contig_18	328889	332613	+		hypothetical protein
FUN_009836	contig_18	342617	343779	+		hypothetical protein
FUN_009837	contig_18	344837	346239	+	LSP1	lipid-binding protein
FUN_009838	contig_18	347383	349311	-		hypothetical protein
FUN_009840	contig_18	353026	354815	-		hypothetical protein
FUN_009841	contig_18	355983	357935	+		hypothetical protein
FUN_009842	contig_18	358277	359564	-	ZRT2	low-affinity Zn(2+) transporter zrt2
FUN_009843	contig_18	361141	362165	+		hypothetical protein
FUN_009844	contig_18	364754	365348	+		hypothetical protein
FUN_009845	contig_18	365588	367372	-		hypothetical protein
FUN_009846	contig_18	368536	369855	+		hypothetical protein
FUN_009848	contig_18	377132	377677	+		hypothetical protein
FUN_009849	contig_18	377949	379519	-		hypothetical protein
FUN_009850	contig_18	381051	383384	-		hypothetical protein
FUN_009852	contig_18	389741	392382	-		hypothetical protein
FUN_009853	contig_18	392863	394704	+	MSY1	tyrosyl-tRNA synthetase
FUN_009854	contig_18	396268	397907	+	MSD1	aspartate--tRNA ligase msd1
FUN_009855	contig_18	398143	398771	-		hypothetical protein
FUN_009856	contig_18	399106	399533	+		hypothetical protein
FUN_009857	contig_18	399667	401397	+		hypothetical protein
FUN_009858	contig_18	402913	405259	-		hypothetical protein
FUN_009859	contig_18	406364	407434	-	RPS4	40S ribosomal protein S4
FUN_009860	contig_18	407716	408982	-	HOM2	aspartate-semialdehyde dehydrogenase
FUN_009861	contig_18	409377	411631	-	DLD1_1	D-lactate ferricytochrome c oxidoreductase
FUN_009863	contig_18	417552	422871	+		hypothetical protein
FUN_009864	contig_18	425214	427000	+		hypothetical protein
FUN_009865	contig_18	427742	429208	-		hypothetical protein
FUN_009866	contig_18	429910	432478	-		hypothetical protein
FUN_009867	contig_18	433603	435365	-		hypothetical protein
FUN_009868	contig_18	436870	438328	+		hypothetical protein
FUN_009869	contig_18	439042	440586	+		hypothetical protein
FUN_009870	contig_18	441483	443676	+		hypothetical protein
FUN_009873	contig_18	449032	451506	-		hypothetical protein
FUN_009874	contig_18	451959	456028	+	KOG1	Target of rapamycin complex 1 subunit kog1
FUN_009875	contig_18	457048	458766	+		hypothetical protein
FUN_009876	contig_18	459511	460342	-	RRP40	exosome non-catalytic core subunit rrp40
FUN_009877	contig_18	460982	462473	+	SSZ1	Hsp70 protein that interacts with Zuo1p
FUN_009878	contig_18	462973	464330	-		hypothetical protein
FUN_009879	contig_18	464743	466704	-		hypothetical protein
FUN_009880	contig_18	471283	473503	-		hypothetical protein
FUN_009881	contig_18	475560	477221	+	PHR1_1	DNA photolyase phr1
FUN_009882	contig_18	478276	479549	-		hypothetical protein
FUN_009883	contig_18	480413	481872	-		hypothetical protein
FUN_009884	contig_18	482719	487431	-	HRR25	serine/threonine protein kinase
FUN_009885	contig_18	491220	493082	+		hypothetical protein
FUN_009886	contig_18	493811	495038	+		hypothetical protein
FUN_009887	contig_18	495166	496829	-		hypothetical protein
FUN_009888	contig_18	497453	498401	-		hypothetical protein
FUN_009890	contig_18	500864	504275	-	TIF32	eukaryotic translation initiation factor 3 subunit A
FUN_009891	contig_18	504973	505362	+	GNG1	Guanine nucleotide-binding protein subunit gamma
FUN_009892	contig_18	506785	508899	+		hypothetical protein
FUN_009893	contig_18	510032	510955	+		hypothetical protein
FUN_009895	contig_18	514002	515144	-		hypothetical protein
FUN_009896	contig_18	515634	516664	-		hypothetical protein
FUN_009897	contig_18	517012	518135	+		hypothetical protein
FUN_009898	contig_18	518968	520261	+	MMT2	mitochondrial metal transporter
FUN_009899	contig_18	520653	522492	+		hypothetical protein
FUN_009900	contig_18	527004	528626	+		hypothetical protein

Gene ID	Scaffold	Start	Stop	Strand	Name	Product
FUN_009901	contig_18	531469	532627	-		hypothetical protein
FUN_009902	contig_18	533636	535244	-	PAN5_2	2-dehydropantoate 2-reductase (Ketopantoate reductase) (KPA reductase) (KPR)
FUN_009903	contig_18	535568	536527	+		hypothetical protein
FUN_009904	contig_18	537353	539595	-	SEC2	rab guanine nucleotide exchange factor S2
FUN_009905	contig_18	540733	541426	+		hypothetical protein
FUN_009906	contig_18	541573	543206	-		hypothetical protein
FUN_009907	contig_18	543762	544293	-	MRP2	40S ribosomal protein mrp2, mitochondrial
FUN_009909	contig_18	547655	548607	+	MET16	3'-phosphoadenylsulfate reductase
FUN_009910	contig_18	549298	551473	+	MET3	Sulfate adenylyltransferase
FUN_009911	contig_18	551745	553093	-		hypothetical protein
FUN_009912	contig_18	553290	554223	+	COQ4	Ubiquinone biosynthesis protein
FUN_009913	contig_18	556148	561223	+		hypothetical protein
FUN_009916	contig_18	566621	567260	+		hypothetical protein
FUN_009918	contig_18	576248	578694	+		hypothetical protein
FUN_009919	contig_18	580720	581886	+	GLC7	type 1 serine/threonine-protein phosphatase catalytic subunit glc7
FUN_009920	contig_18	586515	587979	+		hypothetical protein
FUN_009921	contig_18	589116	590978	-		hypothetical protein
FUN_009922	contig_18	592539	594460	+		hypothetical protein
FUN_009924	contig_18	601005	604308	-		hypothetical protein
FUN_009925	contig_18	606159	607152	-		hypothetical protein
FUN_009926	contig_18	608814	612316	+	ASD1	aspartate-semialdehyde dehydrogenase-like protein
FUN_009927	contig_18	614383	615423	-		hypothetical protein
FUN_009929	contig_18	623489	624430	+		hypothetical protein
FUN_009932	contig_18	629082	629849	-	LPMO9C	AA9 lytic polysaccharide monooxygenase C
FUN_009933	contig_18	630838	632311	-	JIP5	WD repeat-containing protein jip5
FUN_009934	contig_18	633013	636198	+	PMU1_2	putative phosphoglycerate mutase pmu1
FUN_009935	contig_18	637114	638606	-		hypothetical protein
FUN_009936	contig_18	640214	645154	-		hypothetical protein
FUN_009937	contig_18	645509	646790	-		hypothetical protein
FUN_009939	contig_18	648577	650427	-		hypothetical protein
FUN_009940	contig_18	653108	653732	+	SOD2_2	Superoxide dismutase [Mn], mitochondrial
FUN_009941	contig_18	656097	656972	+		hypothetical protein
FUN_009942	contig_18	659684	661140	+		hypothetical protein
FUN_009943	contig_18	661731	662516	-		hypothetical protein
FUN_009947	contig_18	672692	673994	+		hypothetical protein
FUN_009949	contig_18	678918	681206	-		hypothetical protein
FUN_009950	contig_18	682798	685970	-		hypothetical protein
FUN_009951	contig_18	686529	687434	-		hypothetical protein
FUN_009952	contig_18	688123	688982	-		hypothetical protein
FUN_009953	contig_18	690718	691435	-		hypothetical protein
FUN_009954	contig_18	697016	699938	+	CHT2_4	Chitinase 2
FUN_009955	contig_18	700689	702926	+		hypothetical protein
FUN_009957	contig_18	704440	705938	+	ARO8_2	Aromatic/aminoadipate aminotransferase 1
FUN_009958	contig_18	706166	706812	+		hypothetical protein
FUN_009960	contig_18	708587	709649	+		hypothetical protein
FUN_009961	contig_18	709934	711149	-		hypothetical protein
FUN_009964	contig_18	723893	725938	-		hypothetical protein
FUN_009965	contig_18	726822	727526	+		hypothetical protein
FUN_009967	contig_18	730506	733000	-	MEF1	Elongation factor G, mitochondrial
FUN_009971	contig_18	749619	752641	+		hypothetical protein
FUN_009972	contig_18	753419	755089	-		hypothetical protein
FUN_009974	contig_18	762456	764576	+		hypothetical protein
FUN_009976	contig_18	768806	770924	-		hypothetical protein
FUN_009978	contig_18	778380	780962	+		hypothetical protein
FUN_009979	contig_18	781879	782706	+		hypothetical protein
FUN_009980	contig_18	784219	785346	+		hypothetical protein
FUN_009982	contig_18	787822	789409	-		hypothetical protein

Gene ID	Scaffold	Start	Stop	Strand	Name	Product
FUN_009983	contig_18	790517	793523	-	SEC21	coatomer subunit gamma
FUN_009984	contig_18	793936	795174	+		hypothetical protein
FUN_009985	contig_18	796181	798146	-		hypothetical protein
FUN_009986	contig_18	798723	800631	-		hypothetical protein
FUN_009987	contig_18	800960	801589	+	naa20	N-alpha-acetyltransferase 20
FUN_009988	contig_18	802079	804532	-		hypothetical protein
FUN_009989	contig_18	806570	807227	-		hypothetical protein
FUN_009991	contig_18	813812	814743	+		hypothetical protein
FUN_009992	contig_18	815169	816961	-		hypothetical protein
FUN_009993	contig_18	818696	819439	+	PRX1	peroxiredoxin 1
FUN_009994	contig_18	821344	824031	+		hypothetical protein
FUN_009997	contig_18	829838	830431	+		hypothetical protein
FUN_009998	contig_18	830985	833799	-		hypothetical protein
FUN_010000	contig_18	836945	838730	-	NMT1	glycylpeptide N-tetradecanoyltransferase
FUN_010002	contig_18	841718	843651	-	RTC5	Restriction of telomere capping protein 5
FUN_010003	contig_18	843999	845269	+		hypothetical protein
FUN_010005	contig_18	847047	848740	-		hypothetical protein
FUN_010006	contig_18	849359	853344	+	SMC1	Structural maintenance of chromosomes protein 1
FUN_010009	contig_18	861894	862673	-	SOL1	suppressor of los1-1
FUN_010010	contig_18	869534	871055	-		hypothetical protein
FUN_010011	contig_18	872612	873852	+		hypothetical protein
FUN_010012	contig_18	874157	876501	-		hypothetical protein
FUN_010013	contig_18	879438	879917	-		hypothetical protein
FUN_010015	contig_18	885037	885900	-		hypothetical protein
FUN_010016	contig_18	886622	887960	+		hypothetical protein
FUN_010017	contig_18	888094	889632	-		hypothetical protein
FUN_010018	contig_18	890145	892064	-	HIP1_1	histidine permease
FUN_010022	contig_18	901370	902777	+		hypothetical protein
FUN_010023	contig_18	903909	905270	+		hypothetical protein
FUN_010025	contig_18	909023	911068	-		hypothetical protein
FUN_010026	contig_18	912867	914989	-		hypothetical protein
FUN_010027	contig_18	916411	918587	+		hypothetical protein
FUN_010028	contig_18	919260	922051	-		hypothetical protein
FUN_010029	contig_18	923874	926033	+		hypothetical protein
FUN_010031	contig_18	930065	930887	-		hypothetical protein
FUN_010033	contig_19	43326	44741	-		hypothetical protein
FUN_010034	contig_19	45788	46891	+		hypothetical protein
FUN_010035	contig_19	46948	49143	-		hypothetical protein
FUN_010036	contig_19	49449	50198	-		hypothetical protein
FUN_010038	contig_19	54879	58080	-		hypothetical protein
FUN_010039	contig_19	58758	60482	+		hypothetical protein
FUN_010040	contig_19	61133	62697	+		hypothetical protein
FUN_010041	contig_19	63696	65080	-		hypothetical protein
FUN_010043	contig_19	68714	69949	+		hypothetical protein
FUN_010044	contig_19	70489	72921	-		hypothetical protein
FUN_010045	contig_19	74430	76556	-		hypothetical protein
FUN_010046	contig_19	77129	79031	+		hypothetical protein
FUN_010047	contig_19	82368	83183	+		hypothetical protein
FUN_010049	contig_19	85559	86673	-		hypothetical protein
FUN_010050	contig_19	87357	87743	-		hypothetical protein
FUN_010051	contig_19	91579	92493	+		hypothetical protein
FUN_010053	contig_19	94755	95735	+		hypothetical protein
FUN_010054	contig_19	96708	98532	+	HXT5_3	hexose transporter hxt5
FUN_010055	contig_19	98871	99857	+		hypothetical protein
FUN_010058	contig_19	104286	105329	-		hypothetical protein
FUN_010059	contig_19	106096	107083	+		hypothetical protein
FUN_010060	contig_19	108292	109342	+		hypothetical protein
FUN_010061	contig_19	109733	112978	+		hypothetical protein
FUN_010063	contig_19	116356	117468	-		hypothetical protein

Gene ID	Scaffold	Start	Stop	Strand	Name	Product
FUN_010066	contig_19	123409	124980	-		hypothetical protein
FUN_010067	contig_19	126272	129373	+		hypothetical protein
FUN_010068	contig_19	134063	135238	-		hypothetical protein
FUN_010069	contig_19	136302	137956	+	GCD11	eukaryotic translation initiation factor 2 subunit gamma
FUN_010070	contig_19	138941	140291	+		hypothetical protein
FUN_010071	contig_19	141737	144329	-	mcm7	DNA replication licensing factor MCM7
FUN_010072	contig_19	144683	148450	+	PEX1	Peroxisome biosynthesis protein pex1
FUN_010073	contig_19	148671	149282	-		hypothetical protein
FUN_010074	contig_19	149819	150824	-	smb1	Small nuclear ribonucleoprotein-associated protein B
FUN_010075	contig_19	151351	151949	+	RPL11	60S ribosomal protein L11
FUN_010078	contig_19	161912	163734	-	ENO1	phosphopyruvate hydratase
FUN_010079	contig_19	164984	165711	+	PRE1	Proteasome subunit beta type-4
FUN_010080	contig_19	166479	167965	-		hypothetical protein
FUN_010081	contig_19	169301	170262	+		hypothetical protein
FUN_010083	contig_19	172780	174058	-	RPT4	26S proteasome subunit rpt4
FUN_010084	contig_19	174432	176222	+	RRP9	pre-rRNA processing protein
FUN_010085	contig_19	176676	178235	-	ubx2	UBX domain protein Ubx2
FUN_010086	contig_19	179108	185028	+	ECM29	proteasome component M29
FUN_010087	contig_19	185150	185521	-		hypothetical protein
FUN_010088	contig_19	185793	187862	-	cyp15	Peptidyl-prolyl cis-trans isomerase cyp15
FUN_010091	contig_19	194579	197134	-		hypothetical protein
FUN_010093	contig_19	199535	200186	-	CSH3	Protein csh3
FUN_010094	contig_19	200738	201727	-		hypothetical protein
FUN_010095	contig_19	202673	206042	+		hypothetical protein
FUN_010096	contig_19	209136	209977	-	PRE7	Proteasome subunit beta type-6
FUN_010097	contig_19	210470	211253	-		hypothetical protein
FUN_010098	contig_19	211508	212440	+	PRE4	Proteasome subunit beta type-7
FUN_010099	contig_19	212734	213717	+	AXS2	UDP-D-apiose/UDP-D-xylose synthase 2
FUN_010100	contig_19	213748	213975	+		hypothetical protein
FUN_010101	contig_19	215449	217428	-	CDC6	AAA ATPase
FUN_010102	contig_19	217978	221218	-		hypothetical protein
FUN_010103	contig_19	222186	224937	-		hypothetical protein
FUN_010104	contig_19	230701	236890	-	STT4	Phosphatidylinositol 4-kinase stt4
FUN_010105	contig_19	237403	239884	-		hypothetical protein
FUN_010106	contig_19	240333	243443	+		hypothetical protein
FUN_010109	contig_19	253291	256224	-	KIP2	Kinesin-like protein kip2
FUN_010112	contig_19	260232	262996	+	utp13	U3 small nucleolar RNA-associated protein 13
FUN_010113	contig_19	263065	263880	-		hypothetical protein
FUN_010114	contig_19	264454	265556	+		hypothetical protein
FUN_010117	contig_19	273869	278453	-	BEM3	Rho GTPase activating protein
FUN_010118	contig_19	282061	283251	+	CPR2	Peptidyl-prolyl cis-trans isomerase B
FUN_010119	contig_19	283940	286093	+	DED1	DEAD-box ATP-dependent RNA helicase
FUN_010120	contig_19	287367	289755	+		hypothetical protein
FUN_010121	contig_19	290074	291795	-	cdc1	DNA polymerase delta small subunit Cdc1
FUN_010123	contig_19	293961	295324	-		hypothetical protein
FUN_010124	contig_19	295635	296343	-	URA6	bifunctional uridylate/adenylate kinase
FUN_010127	contig_19	309992	312090	+		hypothetical protein
FUN_010129	contig_19	316940	317743	+		hypothetical protein
FUN_010131	contig_19	320653	322186	-	STE24	zinc metalloprotease
FUN_010132	contig_19	323399	325214	+	ERG12	Mevalonate kinase
FUN_010133	contig_19	325548	326783	-	PXR1	telomerase inhibitor
FUN_010135	contig_19	328170	330729	-	RIM8	ph-response sensor protein
FUN_010136	contig_19	332552	337084	-		hypothetical protein
FUN_010137	contig_19	337630	338419	-		hypothetical protein
FUN_010138	contig_19	339273	339961	-		hypothetical protein
FUN_010139	contig_19	340184	341329	+	arc1	ARP2/3 actin-organizing complex subunit Sop2
FUN_010140	contig_19	341473	342893	-		hypothetical protein
FUN_010141	contig_19	345403	347312	+		hypothetical protein
FUN_010144	contig_19	353353	354430	+		hypothetical protein

Gene ID	Scaffold	Start	Stop	Strand	Name	Product
FUN_010146	contig_19	360427	362073	+		hypothetical protein
FUN_010147	contig_19	366044	367717	-	ERG11	Lanosterol 14-alpha-demethylase
FUN_010148	contig_19	368181	370436	-	DUN1	serine/threonine protein kinase
FUN_010149	contig_19	373161	374195	+	SDH2	succinate dehydrogenase complex, subunit B
FUN_010150	contig_19	376762	378505	+		hypothetical protein
FUN_010151	contig_19	378677	380212	-	RSA4	ribosome assembly
FUN_010152	contig_19	380463	383410	+	MSH4	MutS protein msh4
FUN_010153	contig_19	383870	385193	+		hypothetical protein
FUN_010154	contig_19	385412	386264	-	RRS1	Rhodanese-related sulfurtransferase
FUN_010155	contig_19	386475	387960	+		hypothetical protein
FUN_010158	contig_19	394111	396833	+		hypothetical protein
FUN_010159	contig_19	397377	400490	-		hypothetical protein
FUN_010160	contig_19	400808	405533	-	GDB1	bifunctional 4-alpha-glucanotransferase/amylo-alpha-1,6-glucosidase
FUN_010161	contig_19	406617	408728	-		hypothetical protein
FUN_010162	contig_19	410088	411166	+	BET2	Rab geranylgeranyltransferase
FUN_010164	contig_19	416090	419100	+		hypothetical protein
FUN_010166	contig_19	421923	422337	+		hypothetical protein
FUN_010167	contig_19	423903	429225	+	SNQ2_3	ATP-binding cassette transporter snq2
FUN_010168	contig_19	429458	430321	-		hypothetical protein
FUN_010169	contig_19	430864	432086	+	MRI1	S-methyl-5-thioribose-1-phosphate isomerase
FUN_010171	contig_19	432770	437123	+	PAN1	actin organization and endocytosis protein
FUN_010172	contig_19	437882	438481	+	RPB11	DNA-directed RNA polymerase II core subunit
FUN_010174	contig_19	443616	444992	-		hypothetical protein
FUN_010175	contig_19	445281	445963	+	COQ10	Coenzyme Q-binding protein coq10, mitochondrial
FUN_010176	contig_19	446188	449801	-	IRE1	bifunctional endoribonuclease/protein kinase ire1
FUN_010177	contig_19	450921	452673	-	CCT2	T-complex protein 1 subunit beta
FUN_010178	contig_19	452874	454568	+	ubp10	Ubiquitin carboxyl-terminal hydrolase 10
FUN_010179	contig_19	454968	455575	+		hypothetical protein
FUN_010182	contig_19	463025	464903	+		hypothetical protein
FUN_010183	contig_19	465163	465701	-		hypothetical protein
FUN_010184	contig_19	467083	468923	-		hypothetical protein
FUN_010187	contig_19	477315	478449	-	CYT1	cytochrome c1
FUN_010188	contig_19	479009	485059	-	ATG1	Serine/threonine-protein kinase
FUN_010189	contig_19	486987	488936	-	SEC59	dolichol kinase
FUN_010190	contig_19	490588	493346	-	APE2_2	Aminopeptidase 2 mitochondrial
FUN_010191	contig_19	494842	498265	-	PSH1	E3 ubiquitin ligase
FUN_010193	contig_19	504872	505971	-		hypothetical protein
FUN_010195	contig_19	508163	509903	-		hypothetical protein
FUN_010196	contig_19	510447	513697	-		hypothetical protein
FUN_010197	contig_19	514079	515310	-		hypothetical protein
FUN_010198	contig_19	515794	517395	+		hypothetical protein
FUN_010199	contig_19	520777	522019	+	GPD1	glycerol-3-phosphate dehydrogenase
FUN_010200	contig_19	523495	525641	+		hypothetical protein
FUN_010201	contig_19	526601	528550	-		hypothetical protein
FUN_010203	contig_19	530983	532749	-	UME6_1	DNA-binding transcriptional regulator ume6
FUN_010204	contig_19	536484	539083	+		hypothetical protein
FUN_010205	contig_19	541229	541642	-		hypothetical protein
FUN_010206	contig_19	543170	544896	+	MSM1	methionyl-tRNA synthetase
FUN_010207	contig_19	545018	547623	-		hypothetical protein
FUN_010208	contig_19	549047	549741	-		hypothetical protein
FUN_010209	contig_19	555554	558413	+	RCY1	F-box protein: endocytic membrane traffic, recycling ReCYcling 1
FUN_010210	contig_19	558698	559835	+		hypothetical protein
FUN_010212	contig_19	562103	562802	+	TPM2	tropomyosin-2
FUN_010213	contig_19	563180	563886	-		hypothetical protein
FUN_010214	contig_19	564273	565598	-	CDC43	geranylgeranyl transferase type-1 subunit beta
FUN_010215	contig_19	566240	568197	+	rga8	Rho-GTPase-activating protein 8
FUN_010217	contig_19	570713	574652	+	syj1	Inositol-1,4,5-trisphosphate 5-phosphatase 1

Gene ID	Scaffold	Start	Stop	Strand	Name	Product
FUN_010218	contig_19	574917	577215	-	PRT1	Translation initiation factor 3 subunit b
FUN_010219	contig_19	578049	579915	-	HIP1_2	histidine permease
FUN_010220	contig_19	583850	586513	-		hypothetical protein
FUN_010221	contig_19	587079	588128	-		hypothetical protein
FUN_010222	contig_19	588692	590296	-		hypothetical protein
FUN_010223	contig_19	591164	594346	+	CYM1	Mitochondrial presequence protease
FUN_010224	contig_19	595935	597325	-		hypothetical protein
FUN_010225	contig_19	598028	598558	+	YIP3	Prenylated Rab acceptor 1
FUN_010227	contig_19	600719	601564	+		hypothetical protein
FUN_010229	contig_19	603587	605667	-	GTS1	Protein gts1
FUN_010231	contig_19	613258	614641	+		hypothetical protein
FUN_010234	contig_19	621778	622550	+	FCF1	rRNA-processing protein fcf1
FUN_010236	contig_19	626990	628528	+	IZH3	inc metabolism membrane protein
FUN_010237	contig_19	629155	631509	-		hypothetical protein
FUN_010238	contig_19	632464	634350	-		hypothetical protein
FUN_010239	contig_19	635197	636790	+	CAR2	ornithine aminotransferase
FUN_010240	contig_19	638719	643222	+	YOR1_1	ATP-binding cassette transporter yor1
FUN_010242	contig_19	648401	650478	-		hypothetical protein
FUN_010243	contig_19	652093	653726	-		hypothetical protein
FUN_010244	contig_19	653938	656227	+		hypothetical protein
FUN_010246	contig_19	662634	663770	+	HIS1	ATP phosphoribosyltransferase (ATP-PRTase) (ATP-PRT)
FUN_010247	contig_19	664207	664770	-		hypothetical protein
FUN_010248	contig_19	670536	671772	-	ARO3	3-deoxy-7-phosphoheptulonate synthase
FUN_010249	contig_19	672647	673699	+		hypothetical protein
FUN_010251	contig_19	676879	678744	-		hypothetical protein
FUN_010252	contig_19	679143	680953	-		hypothetical protein
FUN_010253	contig_19	682388	684073	+		hypothetical protein
FUN_010254	contig_19	684849	686061	-		hypothetical protein
FUN_010256	contig_19	688568	691506	-	TPS3	Trehalose-6-P synthase/phosphatase complex subunit
FUN_010261	contig_19	706083	709814	+		hypothetical protein
FUN_010262	contig_19	710169	713512	+		hypothetical protein
FUN_010263	contig_19	714338	714952	+	DPH4	Diphthamide biosynthesis protein 4
FUN_010264	contig_19	717035	719798	-		hypothetical protein
FUN_010265	contig_19	721562	723781	+		hypothetical protein
FUN_010266	contig_19	726284	730141	+		hypothetical protein
FUN_010267	contig_19	731559	732374	+		hypothetical protein
FUN_010268	contig_19	733305	734740	-	SPE1	Ornithine decarboxylase
FUN_010270	contig_19	741336	742262	+		hypothetical protein
FUN_010271	contig_19	742718	743564	-		hypothetical protein
FUN_010272	contig_19	745229	746590	+		hypothetical protein
FUN_010275	contig_19	750858	752246	-	spp2	DNA primase large subunit Spp2
FUN_010276	contig_19	752960	755064	-	MDM31	Mitochondrial distribution and morphology protein 31, mitochondrial precursor
FUN_010277	contig_19	755596	756543	+	PNO1	pre-rRNA-processing protein pno1
FUN_010278	contig_19	756670	758926	-		hypothetical protein
FUN_010279	contig_19	760720	762539	-		hypothetical protein
FUN_010280	contig_19	768344	769549	-	txl1	Thioredoxin-like protein 1
FUN_010281	contig_19	769955	771611	+		hypothetical protein
FUN_010282	contig_19	772951	773672	-	rpl36	ribosomal protein L36
FUN_010283	contig_19	774018	774961	-		hypothetical protein
FUN_010284	contig_19	775355	777116	+		hypothetical protein
FUN_010287	contig_19	781578	782815	+	SHP1_1	protein phosphatase regulator
FUN_010289	contig_19	785946	790855	+		hypothetical protein
FUN_010291	contig_19	792395	793111	+		hypothetical protein
FUN_010294	contig_19	801051	801727	+		hypothetical protein
FUN_010296	contig_19	805790	807251	-		hypothetical protein
FUN_010297	contig_19	808255	808928	-		hypothetical protein
FUN_010298	contig_19	810908	812352	+		hypothetical protein

Gene ID	Scaffold	Start	Stop	Strand	Name	Product
FUN_010301	contig_19	817205	818308	+		hypothetical protein
FUN_010305	contig_19	827165	829399	-		hypothetical protein
FUN_010306	contig_19	830725	831576	+		hypothetical protein
FUN_010307	contig_19	832829	833682	-		hypothetical protein
FUN_010308	contig_19	834446	835655	+		hypothetical protein
FUN_010309	contig_19	836607	838347	+	TPO5_2	polyamine transporter tpo5
FUN_010311	contig_19	841500	843846	-		hypothetical protein
FUN_010312	contig_19	844462	845645	-	XYN1	Glycoside hydrolase, 10
FUN_010313	contig_20	222	2758	-		hypothetical protein
FUN_010316	contig_20	12423	13469	-		hypothetical protein
FUN_010317	contig_20	26193	29606	+		hypothetical protein
FUN_010318	contig_20	42763	44108	-		hypothetical protein
FUN_010319	contig_20	54774	55909	+		hypothetical protein
FUN_010320	contig_20	66489	69412	+		hypothetical protein
FUN_010327	contig_20	114161	115666	+		hypothetical protein
FUN_010328	contig_20	121533	124317	+	ANKRA2	Ankyrin repeat A protein 2
FUN_010329	contig_20	127254	128279	-	FANK1_1	Fibronectin type 3 and ankyrin repeat domains protein 1
FUN_010332	contig_20	150478	150897	+		hypothetical protein
FUN_010333	contig_20	158114	159984	-		hypothetical protein
FUN_010335	contig_20	165627	166531	+	HPC2_5	HIR complex subunit
FUN_010336	contig_20	168160	169144	+		hypothetical protein
FUN_010337	contig_20	169380	169658	+		hypothetical protein
FUN_010340	contig_20	176830	177286	-	BLM3_5	Proteasome activator BLM10
FUN_010343	contig_20	200566	200901	+		hypothetical protein
FUN_010344	contig_20	201040	201666	+		hypothetical protein
FUN_010347	contig_20	211240	211566	-		hypothetical protein
FUN_010349	contig_20	237565	238479	-		hypothetical protein
FUN_010350	contig_20	239311	239869	+		hypothetical protein
FUN_010351	contig_20	246547	246872	-		hypothetical protein
FUN_010354	contig_20	252013	252684	+		hypothetical protein
FUN_010355	contig_20	260083	261674	+		hypothetical protein
FUN_010356	contig_20	263488	263720	+		hypothetical protein
FUN_010357	contig_20	271253	272445	-		hypothetical protein
FUN_010360	contig_20	285954	286807	-		hypothetical protein
FUN_010361	contig_20	287834	289448	+		hypothetical protein
FUN_010362	contig_20	291240	291677	-		hypothetical protein
FUN_010363	contig_20	295583	296079	+		hypothetical protein
FUN_010364	contig_20	324805	325947	+		hypothetical protein
FUN_010365	contig_20	326008	326505	+		hypothetical protein
FUN_010366	contig_20	329251	330630	-		hypothetical protein
FUN_010367	contig_20	331482	332034	+		hypothetical protein
FUN_010368	contig_20	339696	340481	-		hypothetical protein
FUN_010369	contig_20	341114	342578	-		hypothetical protein
FUN_010370	contig_20	342994	343407	-		hypothetical protein
FUN_010371	contig_20	354944	356137	-		hypothetical protein
FUN_010372	contig_20	372734	373093	+		hypothetical protein
FUN_010373	contig_20	373995	378182	+		hypothetical protein
FUN_010375	contig_20	383746	384126	+		hypothetical protein
FUN_010376	contig_20	384771	386424	-		hypothetical protein
FUN_010377	contig_20	393434	395183	+		hypothetical protein
FUN_010378	contig_20	407166	407858	-		hypothetical protein
FUN_010381	contig_20	414743	416238	-		hypothetical protein
FUN_010382	contig_20	417867	418771	-	HPC2_6	HIR complex subunit
FUN_010385	contig_20	451621	452536	+		hypothetical protein
FUN_010387	contig_20	465771	467124	-		hypothetical protein
FUN_010388	contig_20	481244	481522	-		hypothetical protein
FUN_010389	contig_20	482469	482859	-	SMT3_3	SUMO protein smt3
FUN_010390	contig_20	511633	514985	+		hypothetical protein
FUN_010393	contig_20	530624	531484	-		hypothetical protein

Gene ID	Scaffold	Start	Stop	Strand	Name	Product
FUN_010395	contig_20	555786	556152	-		hypothetical protein
FUN_010396	contig_20	563083	564485	+	MET7_4	Folylpolyglutamate synthetase
FUN_010397	contig_20	564687	565351	+	PCL1_6	PHO85 cyclin-1
FUN_010398	contig_20	566083	566578	+	RPD3_1	histone deacetylase
FUN_010400	contig_20	572143	573300	+		hypothetical protein
FUN_010401	contig_20	574097	574982	+		hypothetical protein
FUN_010405	contig_20	596595	597994	+	AZF1_3	DNA-binding transcription factor
FUN_010406	contig_20	599420	600021	+		hypothetical protein
FUN_010407	contig_20	615523	615876	-		hypothetical protein
FUN_010409	contig_20	629202	629941	+		hypothetical protein
FUN_010411	contig_20	644393	645613	+		hypothetical protein
FUN_010412	contig_20	658788	659264	+	MIF2_4	mitotic fidelity of chromosome transmission-related protein
FUN_010413	contig_20	660426	660779	-		hypothetical protein
FUN_010414	contig_20	673046	674227	-		hypothetical protein
FUN_010415	contig_20	676941	677783	+		hypothetical protein
FUN_010417	contig_20	716971	717548	+		hypothetical protein
FUN_010419	contig_20	738516	739554	+		hypothetical protein
FUN_010421	contig_20	743309	743500	+		hypothetical protein
FUN_010422	contig_20	744193	745524	-		hypothetical protein
FUN_010423	contig_20	745991	747787	+		hypothetical protein
FUN_010426	contig_20	751425	751955	-		hypothetical protein
FUN_010427	contig_20	753989	754300	-		hypothetical protein
FUN_010429	contig_20	767866	768558	-		hypothetical protein
FUN_010430	contig_20	774097	774734	-	RPD3_2	histone deacetylase
FUN_010432	contig_20	792710	796106	+		hypothetical protein
FUN_010433	contig_20	797272	798057	+		hypothetical protein
FUN_010434	contig_20	798453	799451	-		hypothetical protein
FUN_010435	contig_20	804820	805725	+		hypothetical protein
FUN_010436	contig_20	806864	808204	+		hypothetical protein
FUN_010439	contig_20	816925	820275	-		hypothetical protein
FUN_010440	contig_21	7470	8767	+		hypothetical protein
FUN_010441	contig_21	9766	11655	-		hypothetical protein
FUN_010442	contig_21	12378	12908	-		hypothetical protein
FUN_010445	contig_21	24549	26329	+		hypothetical protein
FUN_010446	contig_21	26470	27188	-		hypothetical protein
FUN_010447	contig_21	27595	29598	+		hypothetical protein
FUN_010448	contig_21	31910	33140	-		hypothetical protein
FUN_010452	contig_21	39773	41731	-		hypothetical protein
FUN_010454	contig_21	47500	48472	+		hypothetical protein
FUN_010455	contig_21	49091	51504	+		hypothetical protein
FUN_010456	contig_21	51637	53618	-		hypothetical protein
FUN_010457	contig_21	53942	55290	-		hypothetical protein
FUN_010458	contig_21	56226	56732	+		hypothetical protein
FUN_010459	contig_21	58891	59796	+		hypothetical protein
FUN_010460	contig_21	60223	61029	+		hypothetical protein
FUN_010461	contig_21	61805	62084	-		hypothetical protein
FUN_010462	contig_21	62572	62973	+		hypothetical protein
FUN_010463	contig_21	63844	64814	+		hypothetical protein
FUN_010464	contig_21	65929	67544	-		hypothetical protein
FUN_010465	contig_21	71405	71848	+		hypothetical protein
FUN_010467	contig_21	82387	83432	+		hypothetical protein
FUN_010468	contig_21	83764	84430	-		hypothetical protein
FUN_010469	contig_21	85074	86552	+		hypothetical protein
FUN_010470	contig_21	89575	90895	+	CDC73	accessory factor associated with RNA polymerase II
FUN_010471	contig_21	91747	94168	-	LEU1	3-isopropylmalate dehydratase
FUN_010472	contig_21	94715	96184	-	ALG7	tunicamycin resistance protein
FUN_010473	contig_21	96422	97796	+		hypothetical protein
FUN_010475	contig_21	109304	111118	+		hypothetical protein

Gene ID	Scaffold	Start	Stop	Strand	Name	Product
FUN_010478	contig_21	115351	117245	+		hypothetical protein
FUN_010480	contig_21	121905	123698	-		hypothetical protein
FUN_010481	contig_21	124748	125295	+	cdc4	myosin II light chain
FUN_010483	contig_21	127168	127840	+	HNT2	Dinucleoside triphosphate hydrolase
FUN_010487	contig_21	138259	139308	-	TMP1	Thymidylate synthase
FUN_010491	contig_21	150696	152544	+		hypothetical protein
FUN_010492	contig_21	154804	158065	-		hypothetical protein
FUN_010493	contig_21	161377	163101	-		hypothetical protein
FUN_010494	contig_21	163617	164930	+		hypothetical protein
FUN_010495	contig_21	165385	169589	-		hypothetical protein
FUN_010496	contig_21	170073	170630	+		hypothetical protein
FUN_010498	contig_21	173900	175047	+		hypothetical protein
FUN_010499	contig_21	181050	181629	-		hypothetical protein
FUN_010500	contig_21	182638	183351	-		hypothetical protein
FUN_010502	contig_21	186733	190230	-	HRQ1	ATP-dependent 3'-5' DNA helicase
FUN_010503	contig_21	191033	192967	-	OPT7	OPT super
FUN_010504	contig_21	194542	200901	+	SMC2_2	Structural maintenance of chromosomes protein 2
FUN_010505	contig_21	201057	203550	-		hypothetical protein
FUN_010506	contig_21	203949	204792	+		hypothetical protein
FUN_010508	contig_21	207284	210022	-		hypothetical protein
FUN_010511	contig_21	220882	227394	-		hypothetical protein
FUN_010512	contig_21	228130	229049	+		hypothetical protein
FUN_010513	contig_21	233848	236579	+	URA9	Dihydroorotate dehydrogenase (quinone), mitochondrial
FUN_010514	contig_21	256161	256667	+		hypothetical protein
FUN_010515	contig_21	256923	258509	-		hypothetical protein
FUN_010517	contig_21	264618	267801	+		hypothetical protein
FUN_010518	contig_21	267882	269243	-		hypothetical protein
FUN_010520	contig_21	273225	273938	-		hypothetical protein
FUN_010521	contig_21	274473	276323	-		hypothetical protein
FUN_010524	contig_21	280230	281188	-	TIF35	translation initiation factor eIF3 subunit g
FUN_010525	contig_21	281925	282814	+		hypothetical protein
FUN_010526	contig_21	283270	285637	+	AKR1_1	palmitoyltransferase akr1
FUN_010527	contig_21	285999	287874	+	AKR1_2	palmitoyltransferase akr1
FUN_010528	contig_21	296385	298639	+		hypothetical protein
FUN_010535	contig_21	316037	317645	-	CYS4	cystathionine beta-synthase
FUN_010536	contig_21	319769	320785	-		hypothetical protein
FUN_010537	contig_21	322130	323449	-		hypothetical protein
FUN_010540	contig_21	350165	351879	-		hypothetical protein
FUN_010541	contig_21	353462	355157	+		hypothetical protein
FUN_010543	contig_21	358026	358967	+		hypothetical protein
FUN_010544	contig_21	359186	359753	-		hypothetical protein
FUN_010545	contig_21	360761	362072	-		hypothetical protein
FUN_010548	contig_21	382247	383835	+		hypothetical protein
FUN_010549	contig_21	384615	386099	+		hypothetical protein
FUN_010550	contig_21	387077	388887	+		hypothetical protein
FUN_010551	contig_21	390400	394399	+		hypothetical protein
FUN_010552	contig_21	394598	395619	-		hypothetical protein
FUN_010553	contig_21	395961	398298	+	ARG2	Amino-acid acetyltransferase, mitochondrial
FUN_010554	contig_21	401159	404741	+		hypothetical protein
FUN_010555	contig_21	405901	409966	+		hypothetical protein
FUN_010556	contig_21	410231	411352	-		hypothetical protein
FUN_010557	contig_21	411868	413116	-	LAP1_2	Leucine aminopeptidase 1
FUN_010559	contig_21	420210	422228	-		hypothetical protein
FUN_010560	contig_21	423096	423807	-	CDC36	transcriptional regulator
FUN_010561	contig_21	424285	425926	-		hypothetical protein
FUN_010562	contig_21	429292	430299	+	MRPL4	54S ribosomal protein L4 mitochondrial
FUN_010563	contig_21	430836	432578	+	GDI1	Rab GDP dissociation inhibitor alpha
FUN_010564	contig_21	432955	436202	+	MCD4	Glycosyl phosphatidyl inositol anchor synthesis
FUN_010566	contig_21	438634	441615	-	SEC27	Coatomer subunit beta'

Gene ID	Scaffold	Start	Stop	Strand	Name	Product
FUN_010568	contig_21	452918	454511	-	PRO1	Glutamate 5-kinase
FUN_010569	contig_21	454770	455501	+	ATP23	Mitochondrial inner membrane protease atp23
FUN_010570	contig_21	455768	456487	-	TIM21	mitochondrial import inner membrane translocase subunit tim21
FUN_010571	contig_21	456701	458235	+	MSP1	mitochondrial dynamin GTPase Msp1
FUN_010572	contig_21	458816	461301	-		hypothetical protein
FUN_010573	contig_21	462624	463519	-		hypothetical protein
FUN_010574	contig_21	463846	465151	+	IPL1	spindle assembly checkpoint kinase
FUN_010575	contig_21	465624	466125	+		hypothetical protein
FUN_010576	contig_21	466318	468182	-		hypothetical protein
FUN_010577	contig_21	468681	470174	-	OLE1_2	stearoyl-CoA 9-desaturase
FUN_010578	contig_21	472679	474601	+		hypothetical protein
FUN_010579	contig_21	475514	477758	-	FAA4	long-chain fatty acid-CoA ligase
FUN_010580	contig_21	478335	479381	+	RKM5	Ribosomal protein lysine methyltransferase
FUN_010581	contig_21	481668	483823	-		hypothetical protein
FUN_010582	contig_21	485566	488526	+	SET3	SET domain-containing protein 3
FUN_010583	contig_21	489466	490770	-	PHO80_2	Pho80p cyclin
FUN_010584	contig_21	491932	493447	-		hypothetical protein
FUN_010585	contig_21	494966	496674	+		hypothetical protein
FUN_010586	contig_21	496990	497655	-		hypothetical protein
FUN_010587	contig_21	501201	502553	-		hypothetical protein
FUN_010588	contig_21	503409	504532	+		hypothetical protein
FUN_010589	contig_21	511543	512718	-		hypothetical protein
FUN_010590	contig_21	521548	522524	-		hypothetical protein
FUN_010591	contig_21	525627	526790	-		hypothetical protein
FUN_010592	contig_21	526899	528835	-	CTF1	Transcriptional activator of fatty acid utilization
FUN_010593	contig_21	529475	530075	-		hypothetical protein
FUN_010594	contig_21	530792	532197	-		hypothetical protein
FUN_010595	contig_21	532822	533908	+		hypothetical protein
FUN_010597	contig_21	536416	538236	+		hypothetical protein
FUN_010598	contig_21	538940	540829	+	LAP2_2	Leucyl aminopeptidase yscIV
FUN_010599	contig_21	541005	541825	-		hypothetical protein
FUN_010600	contig_21	542147	544357	+		hypothetical protein
FUN_010601	contig_21	545075	545840	+		hypothetical protein
FUN_010602	contig_21	547674	547967	+		hypothetical protein
FUN_010603	contig_21	548002	548517	+		hypothetical protein
FUN_010604	contig_21	549662	550186	+	ibp1	Cdc25 phosphatase Ibp1
FUN_010605	contig_21	556829	558739	+		hypothetical protein
FUN_010607	contig_21	562683	565048	+	PRD1_2	metalloendopeptidase
FUN_010610	contig_21	573297	574903	-	ADA2	Transcriptional adapter ada2
FUN_010611	contig_21	575602	577760	+	YPK2	Serine/threonine-protein kinase
FUN_010612	contig_21	578591	580435	+	PGI1	glucose-6-phosphate isomerase
FUN_010614	contig_21	582403	583137	-		hypothetical protein
FUN_010615	contig_21	583568	585746	+		hypothetical protein
FUN_010616	contig_21	586460	588233	+	LGD1	L-galactonate dehydratase
FUN_010617	contig_21	588752	590040	-		hypothetical protein
FUN_010618	contig_21	591404	591871	+	ERG28	ergosterol biosynthesis protein
FUN_010619	contig_21	592250	593546	-	GAL7	galactose-1-phosphate uridyl transferase
FUN_010620	contig_21	593868	594805	+		hypothetical protein
FUN_010621	contig_21	596079	597874	+		hypothetical protein
FUN_010622	contig_21	601298	603265	-	BEM1	bud emergence protein 1
FUN_010623	contig_21	605223	606851	-		hypothetical protein
FUN_010624	contig_21	607204	609108	+	VPS36	Vacuolar protein-sorting-associated protein 36
FUN_010625	contig_21	609388	610308	-		hypothetical protein
FUN_010626	contig_21	613981	616053	-		hypothetical protein
FUN_010628	contig_21	617556	619600	+		hypothetical protein
FUN_010629	contig_21	619769	620583	-		hypothetical protein
FUN_010630	contig_21	623442	624713	-		hypothetical protein
FUN_010631	contig_21	625634	626933	+		hypothetical protein

Gene ID	Scaffold	Start	Stop	Strand	Name	Product
FUN_010632	contig_21	631694	634230	+		hypothetical protein
FUN_010633	contig_21	635693	636961	+	GVP36	BAR domain-containing protein
FUN_010634	contig_21	637806	638625	-		hypothetical protein
FUN_010635	contig_21	639913	640842	+		hypothetical protein
FUN_010636	contig_21	643300	643832	+	FPR1A	FK506-binding protein 1A
FUN_010637	contig_21	652385	653254	+		hypothetical protein
FUN_010642	contig_21	672319	673786	-	aat2	Aspartate aminotransferase, cytoplasmic
FUN_010643	contig_21	674571	676052	-		hypothetical protein
FUN_010644	contig_21	680077	681076	-		hypothetical protein
FUN_010645	contig_21	681940	682616	-	RPS11B	40S ribosomal protein S11-B
FUN_010646	contig_21	682826	683915	+		hypothetical protein
FUN_010647	contig_21	684729	686243	+		hypothetical protein
FUN_010648	contig_21	688531	690704	+		hypothetical protein
FUN_010651	contig_21	711551	712741	-	MPD1	protein disulfide isomerase (PDI) protein
FUN_010652	contig_21	713696	715367	-	PNS1	pH nine-sensitive protein 1
FUN_010654	contig_21	717969	718883	+	NPC2_1	Phosphatidylglycerol/phosphatidylinositol transfer protein
FUN_010655	contig_21	719666	721387	+		hypothetical protein
FUN_010656	contig_21	723533	724287	-	SDA1_2	Severe Depolymerization of Actin
FUN_010658	contig_21	726481	727225	-	FOL1	trifunctional dihydropteroate synthetase
FUN_010659	contig_21	728461	729638	+		hypothetical protein
FUN_010660	contig_21	738462	740268	-		hypothetical protein
FUN_010661	contig_21	740851	742055	+		hypothetical protein
FUN_010662	contig_21	743208	745012	-		hypothetical protein
FUN_010665	contig_21	747827	748478	+	NPC2_2	Phosphatidylglycerol/phosphatidylinositol transfer protein
FUN_010666	contig_21	748900	749437	-	VMA7	H(+)-transporting V1 sector ATPase subunit F
FUN_010667	contig_21	749699	751183	+		hypothetical protein
FUN_010668	contig_21	753678	755131	-		hypothetical protein
FUN_010669	contig_21	756020	756684	-		hypothetical protein
FUN_010670	contig_21	757193	758015	+		hypothetical protein
FUN_010671	contig_21	758219	761284	+		hypothetical protein
FUN_010672	contig_21	761869	762928	+		hypothetical protein
FUN_010673	contig_21	763357	763770	+		hypothetical protein
FUN_010674	contig_21	763849	764676	-		hypothetical protein
FUN_010675	contig_21	766069	767166	-		hypothetical protein
FUN_010676	contig_21	768091	769266	+		hypothetical protein
FUN_010677	contig_21	769649	770932	-		hypothetical protein
FUN_010682	contig_22	13901	15533	-		hypothetical protein
FUN_010684	contig_22	18336	19968	-	FDH1_2	formate dehydrogenase (NAD+)
FUN_010685	contig_22	20548	23562	-		hypothetical protein
FUN_010688	contig_22	36524	38122	-		hypothetical protein
FUN_010689	contig_22	38608	41241	-		hypothetical protein
FUN_010691	contig_22	115793	117044	+		hypothetical protein
FUN_010692	contig_22	117707	118832	-		hypothetical protein
FUN_010693	contig_22	119616	121021	+		hypothetical protein
FUN_010694	contig_22	121726	123042	-		hypothetical protein
FUN_010695	contig_22	125131	126961	+		hypothetical protein
FUN_010696	contig_22	128159	129976	-		hypothetical protein
FUN_010697	contig_22	130525	130954	+		hypothetical protein
FUN_010698	contig_22	131692	133930	-		hypothetical protein
FUN_010699	contig_22	137138	138384	+		hypothetical protein
FUN_010700	contig_22	142825	143946	+		hypothetical protein
FUN_010701	contig_22	144191	146618	-		hypothetical protein
FUN_010704	contig_22	158640	160631	+		hypothetical protein
FUN_010705	contig_22	170299	172695	+		hypothetical protein
FUN_010706	contig_22	173370	174452	+		hypothetical protein
FUN_010709	contig_22	181745	183075	-		hypothetical protein
FUN_010710	contig_22	183501	186829	+	MIP1	DNA-directed DNA polymerase gamma mip1
FUN_010711	contig_22	188651	197053	-		hypothetical protein
FUN_010714	contig_22	203713	204801	+		hypothetical protein

Gene ID	Scaffold	Start	Stop	Strand	Name	Product
FUN_010715	contig_22	207215	208885	+		hypothetical protein
FUN_010716	contig_22	209733	210291	-	CUP5	vacuolar ATPase V0 domain subunit c
FUN_010717	contig_22	212308	216144	-		hypothetical protein
FUN_010718	contig_22	218945	220183	-		hypothetical protein
FUN_010719	contig_22	228184	228597	+		hypothetical protein
FUN_010721	contig_22	234053	234992	+		hypothetical protein
FUN_010722	contig_22	235095	236096	-		hypothetical protein
FUN_010725	contig_22	240566	242085	-		hypothetical protein
FUN_010728	contig_22	247163	248317	+		hypothetical protein
FUN_010729	contig_22	249248	250712	+	GAS5	1,3-beta-glucanosyltransferase
FUN_010730	contig_22	251163	252714	+		hypothetical protein
FUN_010731	contig_22	252997	254544	-	PEX29	Peroxisome size and maintenance regulator
FUN_010732	contig_22	255893	260685	+	POM1	serine/threonine protein kinase, CMGC, dual-specificity
FUN_010733	contig_22	262034	263584	+	BNA2	Indoleamine 2,3-dioxygenase
FUN_010734	contig_22	264084	264994	+		hypothetical protein
FUN_010735	contig_22	266786	268053	+		hypothetical protein
FUN_010736	contig_22	269638	270644	+		hypothetical protein
FUN_010737	contig_22	271080	274566	-		hypothetical protein
FUN_010738	contig_22	278060	278750	-		hypothetical protein
FUN_010739	contig_22	281362	282074	-		hypothetical protein
FUN_010740	contig_22	282409	283448	+		hypothetical protein
FUN_010741	contig_22	284188	285031	+	ARF6	ADP-ribosylation factor, Arf Arf6
FUN_010742	contig_22	285797	287542	-		hypothetical protein
FUN_010743	contig_22	288141	289408	+	PMI1	Mannose-6-phosphate isomerase
FUN_010744	contig_22	289866	290744	+		hypothetical protein
FUN_010745	contig_22	291159	292094	+		hypothetical protein
FUN_010746	contig_22	292280	293116	-		hypothetical protein
FUN_010747	contig_22	311071	314874	+		hypothetical protein
FUN_010748	contig_22	315265	316323	-		hypothetical protein
FUN_010749	contig_22	323949	327461	+		hypothetical protein
FUN_010750	contig_22	330321	331236	-		hypothetical protein
FUN_010751	contig_22	331831	332418	+	VAS2	AP-1 adaptor complex sigma subunit Aps1
FUN_010752	contig_22	333507	334653	+	MAF1	RNA polymerase III-inhibiting protein maf1
FUN_010753	contig_22	343853	346579	+		hypothetical protein
FUN_010754	contig_22	348646	350600	+		hypothetical protein
FUN_010756	contig_22	353392	354692	-	NTH1_3	alpha,alpha-trehalase nth1
FUN_010757	contig_22	355060	356904	+		hypothetical protein
FUN_010758	contig_22	357211	357464	+	HCS1	ATP-dependent 5'-3' DNA helicase hcs1
FUN_010762	contig_22	369277	369733	+		hypothetical protein
FUN_010763	contig_22	373736	374855	+		hypothetical protein
FUN_010764	contig_22	375175	376561	-		hypothetical protein
FUN_010765	contig_22	377316	379289	+	REX3	RNA exonuclease 3
FUN_010766	contig_22	383497	384777	+		hypothetical protein
FUN_010767	contig_22	385197	386552	-		hypothetical protein
FUN_010768	contig_22	387416	389350	+	PFA3	palmitoyltransferase for Vac8p
FUN_010769	contig_22	394044	395008	-		hypothetical protein
FUN_010770	contig_22	395895	398396	+		hypothetical protein
FUN_010771	contig_22	398525	400845	-		hypothetical protein
FUN_010772	contig_22	401632	403584	-	UTR1	NAD(+) kinase
FUN_010773	contig_22	405670	406699	-	SPE3	putrescine aminopropyltransferase
FUN_010775	contig_22	408596	409643	+		hypothetical protein
FUN_010776	contig_22	411318	412502	+		hypothetical protein
FUN_010777	contig_22	412758	414337	+	TUB4	gamma-tubulin
FUN_010781	contig_22	421540	423501	+		hypothetical protein
FUN_010783	contig_22	430648	432091	-	ARP2	Actin-related protein 2
FUN_010784	contig_22	432685	433809	+	RPL5	60S ribosomal protein L5
FUN_010785	contig_22	434837	438601	+	MHP1	Microtubules assembly and stabilization protein
FUN_010786	contig_22	441012	442761	-		hypothetical protein
FUN_010787	contig_22	443255	446742	+		hypothetical protein

Gene ID	Scaffold	Start	Stop	Strand	Name	Product
FUN_010788	contig_22	447564	454062	-		hypothetical protein
FUN_010789	contig_22	454972	456676	+		hypothetical protein
FUN_010790	contig_22	458841	459443	-		hypothetical protein
FUN_010791	contig_22	460009	461898	-	SIT1	ferrioxamine B transporter
FUN_010792	contig_22	462677	463817	-		hypothetical protein
FUN_010793	contig_22	464124	467585	-	MRPL35	mitochondrial 54S ribosomal protein YmL35
FUN_010794	contig_22	467727	469595	+	yml6	54S ribosomal protein yml6, mitochondrial
FUN_010795	contig_22	470370	474082	+		hypothetical protein
FUN_010796	contig_22	474522	475295	-		hypothetical protein
FUN_010797	contig_22	475577	476478	+	ATG16	autophagy protein 16, interacts with Atg12p-Atg5p
FUN_010798	contig_22	476733	477849	+	RRI1	COP9 signalosome catalytic subunit rri1
FUN_010799	contig_22	478056	479820	-	MSE1	Glutamate--tRNA ligase mitochondrial
FUN_010800	contig_22	480115	481573	+	XDJ1_2	DnaJ-like protein xdj1
FUN_010801	contig_22	481769	482204	-		hypothetical protein
FUN_010803	contig_22	485909	486444	+		hypothetical protein
FUN_010805	contig_22	489875	492530	+	SPB1	AdoMet-dependent rRNA methyltransferase spb1
FUN_010808	contig_22	496428	497606	+		hypothetical protein
FUN_010810	contig_22	503123	505063	+		hypothetical protein
FUN_010811	contig_22	507159	508561	+		hypothetical protein
FUN_010813	contig_22	515057	515656	+		hypothetical protein
FUN_010814	contig_22	515970	517918	-		hypothetical protein
FUN_010817	contig_22	521138	524438	-		hypothetical protein
FUN_010818	contig_22	527881	528926	-		hypothetical protein
FUN_010819	contig_22	533645	535449	-		hypothetical protein
FUN_010820	contig_22	539218	541171	+		hypothetical protein
FUN_010821	contig_22	542253	542884	+	GPX2	Glutathione peroxidase 2
FUN_010822	contig_22	543101	544416	-	RRD2	Serine/threonine-protein phosphatase 2A activator 2
FUN_010823	contig_22	545544	547224	-		hypothetical protein
FUN_010824	contig_22	548001	550178	-	MBP1	Transcription factor mbp1
FUN_010825	contig_22	550422	550829	+		hypothetical protein
FUN_010826	contig_22	551208	552997	-		hypothetical protein
FUN_010828	contig_22	555627	558826	+		hypothetical protein
FUN_010829	contig_22	559116	560147	+		hypothetical protein
FUN_010832	contig_22	568661	570881	+	HXT5_4	hexose transporter hxt5
FUN_010833	contig_22	572979	574358	-	NOP16	Nucleolar protein 16
FUN_010834	contig_22	574610	576300	+		hypothetical protein
FUN_010835	contig_22	577034	578865	+		hypothetical protein
FUN_010836	contig_22	584243	586529	-		hypothetical protein
FUN_010839	contig_23	6381	7815	-		hypothetical protein
FUN_010840	contig_23	10809	11734	-		hypothetical protein
FUN_010844	contig_23	25854	27323	-		hypothetical protein
FUN_010845	contig_23	28036	30371	+		hypothetical protein
FUN_010846	contig_23	31112	31931	+		hypothetical protein
FUN_010847	contig_23	35002	35626	-		hypothetical protein
FUN_010849	contig_23	38931	39391	-		hypothetical protein
FUN_010850	contig_23	39566	40846	+	TRM10	tRNA (guanine(9)-N(1))-methyltransferase
FUN_010851	contig_23	41423	42418	+	OXR1	oxidation resistance protein 1
FUN_010852	contig_23	43210	44133	+		hypothetical protein
FUN_010853	contig_23	49023	49882	+		hypothetical protein
FUN_010855	contig_23	58443	58821	+		hypothetical protein
FUN_010856	contig_23	58953	64229	+		hypothetical protein
FUN_010857	contig_23	68205	69240	-		hypothetical protein
FUN_010858	contig_23	70265	70984	-	FCR1	Fluconazole resistance protein 1
FUN_010859	contig_23	79953	82171	-		hypothetical protein
FUN_010860	contig_23	83174	83544	+		hypothetical protein
FUN_010861	contig_23	84094	85251	+		hypothetical protein
FUN_010862	contig_23	85818	88318	+	MON1	Vacuolar fusion protein mon1
FUN_010863	contig_23	89397	90276	+		hypothetical protein
FUN_010864	contig_23	90999	91892	+		hypothetical protein

Gene ID	Scaffold	Start	Stop	Strand	Name	Product
FUN_010865	contig_23	92826	95108	-		hypothetical protein
FUN_010867	contig_23	98334	99146	+		hypothetical protein
FUN_010868	contig_23	100386	102359	-		hypothetical protein
FUN_010870	contig_23	105939	106812	-		hypothetical protein
FUN_010871	contig_23	107841	110746	-	ago1_2	Protein argonaute
FUN_010872	contig_23	110922	111298	-	ago1_3	Protein argonaute
FUN_010873	contig_23	113026	116267	-	RNA14	mRNA 3'-end-processing protein rna14
FUN_010874	contig_23	116807	117792	+		hypothetical protein
FUN_010875	contig_23	118441	121512	-	IFM1	translation initiation factor IF-2
FUN_010877	contig_23	123208	123955	+		hypothetical protein
FUN_010878	contig_23	124246	125078	+		hypothetical protein
FUN_010880	contig_23	126595	127441	-		hypothetical protein
FUN_010881	contig_23	128111	129105	-		hypothetical protein
FUN_010884	contig_23	137380	139362	+		hypothetical protein
FUN_010885	contig_23	145563	147121	-	MCA1	Ca(2+)-dependent cysteine protease
FUN_010886	contig_23	149577	154411	-		hypothetical protein
FUN_010888	contig_23	157385	160808	-	CWH43	Protein cwh43
FUN_010889	contig_23	161036	162408	+	UTP6	U3 snoRNP protein
FUN_010890	contig_23	162550	163100	-		hypothetical protein
FUN_010891	contig_23	163541	166171	+	NAB3	nuclear polyadenylated RNA-binding protein 3
FUN_010892	contig_23	166950	168110	+		hypothetical protein
FUN_010893	contig_23	168375	170605	-	GRC3	Polynucleotide 5'-hydroxyl-kinase grc3
FUN_010894	contig_23	170832	172085	+		hypothetical protein
FUN_010895	contig_23	172993	177581	+		hypothetical protein
FUN_010898	contig_23	185658	187463	-		hypothetical protein
FUN_010899	contig_23	187811	190505	+	COG3	Golgi transport complex subunit 3
FUN_010900	contig_23	190859	192417	+	SHM2	Serine hydroxymethyltransferase, cytosolic
FUN_010901	contig_23	192832	194127	-		hypothetical protein
FUN_010902	contig_23	197379	198746	+		hypothetical protein
FUN_010903	contig_23	199119	200933	-		hypothetical protein
FUN_010904	contig_23	201966	203838	+		hypothetical protein
FUN_010905	contig_23	204398	206113	-	PDI1	protein disulfide-isomerase precursor
FUN_010907	contig_23	209218	211341	-		hypothetical protein
FUN_010908	contig_23	212247	214700	+	HRD1	E3 ubiquitin-protein ligase hrd1
FUN_010911	contig_23	219858	221597	+		hypothetical protein
FUN_010912	contig_23	221908	226331	+	SMC4	Structural maintenance of chromosomes protein 4
FUN_010913	contig_23	227684	229394	+		hypothetical protein
FUN_010914	contig_23	230423	231946	-		hypothetical protein
FUN_010916	contig_23	242793	246083	-		hypothetical protein
FUN_010917	contig_23	246659	247914	+		hypothetical protein
FUN_010919	contig_23	252569	253316	-		hypothetical protein
FUN_010920	contig_23	258458	261462	-		hypothetical protein
FUN_010921	contig_23	277174	283777	+	PKS1	polyketide synthase
FUN_010922	contig_23	287947	288379	+		hypothetical protein
FUN_010923	contig_23	291206	292858	-		hypothetical protein
FUN_010924	contig_23	293894	295002	-		hypothetical protein
FUN_010925	contig_23	296628	297258	+		hypothetical protein
FUN_010926	contig_23	297559	298081	+		hypothetical protein
FUN_010927	contig_23	301082	302802	+		hypothetical protein
FUN_010928	contig_23	302908	303474	-		hypothetical protein
FUN_010929	contig_23	304027	306153	+		hypothetical protein
FUN_010932	contig_23	310352	311348	-	tgs1	putative diacylglycerol O-acyltransferase tgs1
FUN_010933	contig_23	311821	313131	+		hypothetical protein
FUN_010935	contig_23	314803	316128	-		hypothetical protein
FUN_010936	contig_23	317772	318848	+		hypothetical protein
FUN_010937	contig_23	321119	322357	+		hypothetical protein
FUN_010938	contig_23	323402	324336	-		hypothetical protein
FUN_010939	contig_23	325074	326321	-		hypothetical protein
FUN_010940	contig_23	327263	328915	+		hypothetical protein

Gene ID	Scaffold	Start	Stop	Strand	Name	Product
FUN_010941	contig_23	329139	331403	-	TAF5	Transcription initiation factor TFIID subunit 5
FUN_010942	contig_23	332234	332761	+		hypothetical protein
FUN_010943	contig_23	334660	337587	-		hypothetical protein
FUN_010944	contig_23	339661	341032	+	RPT3	26S proteasome regulatory subunit 6B
FUN_010945	contig_23	342753	345595	+	MSS4	Phosphatidylinositol-4-phosphate 5-kinase
FUN_010946	contig_23	347570	348787	-		hypothetical protein
FUN_010947	contig_23	349954	353363	-		hypothetical protein
FUN_010948	contig_23	354299	355352	-		hypothetical protein
FUN_010949	contig_23	356776	358388	-	DHH1	DExD/H-box ATP-dependent RNA helicase dhh1
FUN_010950	contig_23	359408	360232	+		hypothetical protein
FUN_010951	contig_23	361357	363345	-		hypothetical protein
FUN_010952	contig_23	364510	367969	-	SPT7	Transcriptional activator spt7
FUN_010953	contig_23	370460	371008	-	RPS26A	40S ribosomal protein S26
FUN_010954	contig_23	371529	373609	+		hypothetical protein
FUN_010955	contig_23	374346	377784	+	PSE1	importin subunit beta-3
FUN_010956	contig_23	378577	379145	-	YKE2	Prefoldin subunit 6
FUN_010957	contig_23	379579	381141	+	VPS4	Vacuolar protein sorting-associated protein 4
FUN_010959	contig_23	386541	387888	+		hypothetical protein
FUN_010960	contig_23	388231	389499	+		hypothetical protein
FUN_010961	contig_23	389562	390476	-	EFM5_1	Protein-lysine N-methyltransferase efm5
FUN_010962	contig_23	390655	391590	+		hypothetical protein
FUN_010966	contig_23	396681	397005	-		hypothetical protein
FUN_010967	contig_23	399236	400145	+		hypothetical protein
FUN_010968	contig_23	400859	405254	-		hypothetical protein
FUN_010969	contig_23	405789	406673	+	PUP1	proteasome core particle subunit beta 2
FUN_010970	contig_23	406983	408926	-	GUP1	glycerol transporter
FUN_010971	contig_23	409216	410723	+	ARP4	Actin-related protein 4
FUN_010972	contig_23	411222	412593	-	MP65	Cell surface mannoprotein mp65
FUN_010973	contig_23	415980	416884	+		hypothetical protein
FUN_010974	contig_23	417430	419051	-	GAD2	Glutamate decarboxylase 2
FUN_010975	contig_23	419366	421570	-	NOP9	Nucleolar protein 9
FUN_010976	contig_23	422003	424853	+		hypothetical protein
FUN_010978	contig_23	427493	428927	-		hypothetical protein
FUN_010980	contig_23	434127	434719	-		hypothetical protein
FUN_010981	contig_23	436028	437115	+		hypothetical protein
FUN_010982	contig_23	437389	440168	-		hypothetical protein
FUN_010984	contig_23	441817	442543	+		hypothetical protein
FUN_010985	contig_23	442995	445535	-		hypothetical protein
FUN_010986	contig_23	456036	457689	-	STR2	Cystathionine gamma-synthase
FUN_010987	contig_23	457980	461624	+	UTP22	U3 snoRNP protein
FUN_010988	contig_23	461696	462605	-		hypothetical protein
FUN_010989	contig_23	466836	467681	-		hypothetical protein
FUN_010990	contig_23	469976	472704	+	SLM2	phosphatidylinositol 4,5-bisphosphate-binding protein
FUN_010991	contig_23	473071	474255	-		hypothetical protein
FUN_010992	contig_23	474713	476736	-		hypothetical protein
FUN_010994	contig_23	481027	482638	+		hypothetical protein
FUN_010995	contig_23	484772	486097	+		hypothetical protein
FUN_010996	contig_23	486355	487269	-		hypothetical protein
FUN_010997	contig_23	488791	492561	-	UBP2	ubiquitin-specific protease ubp2
FUN_010998	contig_23	493025	493763	+		hypothetical protein
FUN_010999	contig_23	495098	496630	+		hypothetical protein
FUN_011000	contig_23	496677	498041	+		hypothetical protein
FUN_011001	contig_23	499181	500188	+	UNG1	uracil DNA glycosylase
FUN_011002	contig_23	501151	502636	+		hypothetical protein
FUN_011003	contig_23	504336	506002	+		hypothetical protein
FUN_011004	contig_23	506425	507546	+		hypothetical protein
FUN_011005	contig_23	508619	510438	-		hypothetical protein
FUN_011006	contig_23	512280	513099	+	NEP1	18S rRNA pseudouridine methyltransferase
FUN_011007	contig_23	514377	516375	+		hypothetical protein

Gene ID	Scaffold	Start	Stop	Strand	Name	Product
FUN_011008	contig_23	517631	521702	+	smc6	Structural maintenance of chromosomes protein 6
FUN_011009	contig_23	521968	525135	+		hypothetical protein
FUN_011010	contig_23	525934	527357	+	ASF1	Histone chaperone asf1
FUN_011011	contig_23	528244	531039	+		hypothetical protein
FUN_011012	contig_23	531455	532405	+		hypothetical protein
FUN_011013	contig_23	532629	534789	-	ATG22_2	Autophagy protein 22
FUN_011014	contig_23	534848	539233	+	RGR1	mediator complex subunit
FUN_011015	contig_23	539569	540433	-		hypothetical protein
FUN_011016	contig_23	540675	543153	+	45931	Mitochondrial intermediate peptidase
FUN_011017	contig_23	544588	548349	-	TFC4	transcription factor TFIIIC subunit tfc4
FUN_011018	contig_23	550548	551293	+		hypothetical protein
FUN_011019	contig_23	554266	556028	+	SLM5	asparaginyl-tRNA synthetase
FUN_011020	contig_23	556806	561012	+		hypothetical protein
FUN_011021	contig_23	561762	564187	-	SQS1	squalene synthetase-like protein
FUN_011022	contig_23	564761	566194	+	ATG4	Cysteine protease atg4
FUN_011023	contig_23	566961	567555	-	HTB1	histone H2B
FUN_011024	contig_23	567978	568661	+	HTA1	histone H2A
FUN_011025	contig_23	569168	569673	-		hypothetical protein
FUN_011026	contig_23	569959	572384	+	KGD2	2-oxoglutarate dehydrogenase complex E2 component
FUN_011027	contig_24	6105	9650	-		hypothetical protein
FUN_011031	contig_24	41437	43523	-		hypothetical protein
FUN_011033	contig_24	49274	50184	+		hypothetical protein
FUN_011036	contig_24	62075	62576	+		hypothetical protein
FUN_011037	contig_24	66402	69382	+		hypothetical protein
FUN_011038	contig_24	70435	72117	-		hypothetical protein
FUN_011039	contig_24	72300	73233	+		hypothetical protein
FUN_011041	contig_24	79110	79496	+		hypothetical protein
FUN_011043	contig_24	110093	110475	-		hypothetical protein
FUN_011044	contig_24	111082	111410	-		hypothetical protein
FUN_011047	contig_24	126020	126394	-		hypothetical protein
FUN_011048	contig_24	132559	133253	+		hypothetical protein
FUN_011050	contig_24	149170	150500	-		hypothetical protein
FUN_011052	contig_24	162544	163907	+		hypothetical protein
FUN_011053	contig_24	164038	164648	-		hypothetical protein
FUN_011054	contig_24	165032	165451	+		hypothetical protein
FUN_011055	contig_24	166231	166711	-		hypothetical protein
FUN_011057	contig_24	174483	175100	+		hypothetical protein
FUN_011059	contig_24	201673	202872	-		hypothetical protein
FUN_011060	contig_24	203516	204285	+		hypothetical protein
FUN_011061	contig_24	209086	209793	-		hypothetical protein
FUN_011063	contig_24	237291	237527	-		hypothetical protein
FUN_011066	contig_24	263825	264890	+		hypothetical protein
FUN_011067	contig_24	269894	270526	-		hypothetical protein
FUN_011068	contig_24	284400	286063	+		hypothetical protein
FUN_011069	contig_24	291098	291823	-		hypothetical protein
FUN_011071	contig_24	308704	309732	+		hypothetical protein
FUN_011072	contig_24	318727	319917	-		hypothetical protein
FUN_011073	contig_24	319939	320208	-		hypothetical protein
FUN_011074	contig_24	320980	321990	-		hypothetical protein
FUN_011075	contig_24	322464	323467	+		hypothetical protein
FUN_011077	contig_24	328844	329035	+		hypothetical protein
FUN_011079	contig_24	341716	342015	+		hypothetical protein
FUN_011080	contig_24	343782	344452	-		hypothetical protein
FUN_011081	contig_24	347256	347825	+		hypothetical protein
FUN_011082	contig_24	360751	361191	+	PCL1_7	PHO85 cyclin-1
FUN_011083	contig_24	374164	376101	+		hypothetical protein
FUN_011085	contig_24	398524	399924	+		hypothetical protein
FUN_011088	contig_24	426668	427424	-	HPC2_7	HIR complex subunit
FUN_011090	contig_24	445298	446479	+		hypothetical protein

Gene ID	Scaffold	Start	Stop	Strand	Name	Product
FUN_011092	contig_24	465892	467823	+		hypothetical protein
FUN_011093	contig_24	467954	468726	+		hypothetical protein
FUN_011095	contig_24	472285	474196	+		hypothetical protein
FUN_011096	contig_24	475793	476958	-	KMT2D	Lysine Methyltransferase 2D
FUN_011097	contig_24	480236	481299	+		hypothetical protein
FUN_011098	contig_24	485918	487452	+	DCL2_2	Dicer-like protein 2
FUN_011099	contig_24	489760	490053	+		hypothetical protein
FUN_011100	contig_24	501020	501406	+		hypothetical protein
FUN_011101	contig_24	502547	503316	-	AZF1_4	DNA-binding transcription factor
FUN_011102	contig_24	530161	533314	-		hypothetical protein
FUN_011104	contig_24	540119	540493	-		hypothetical protein
FUN_011107	contig_25	19351	19734	-		hypothetical protein
FUN_011108	contig_25	20974	21873	+	BLM3_6	Proteasome activator BLM10
FUN_011109	contig_25	22081	24326	-		hypothetical protein
FUN_011110	contig_25	25975	26507	-		hypothetical protein
FUN_011111	contig_25	27510	28785	+		hypothetical protein
FUN_011112	contig_25	29775	29958	-		hypothetical protein
FUN_011113	contig_25	35866	36260	+		hypothetical protein
FUN_011114	contig_25	49974	50789	+		hypothetical protein
FUN_011116	contig_25	64442	64858	+		hypothetical protein
FUN_011118	contig_25	74691	76355	+		hypothetical protein
FUN_011120	contig_25	118150	118482	+		hypothetical protein
FUN_011123	contig_25	141536	142245	+		hypothetical protein
FUN_011124	contig_25	147429	149751	-		hypothetical protein
FUN_011125	contig_25	152023	153042	+		hypothetical protein
FUN_011126	contig_25	156649	158489	-		hypothetical protein
FUN_011127	contig_25	158705	159175	+		hypothetical protein
FUN_011129	contig_25	177793	178029	+		hypothetical protein
FUN_011130	contig_25	183268	184110	-		hypothetical protein
FUN_011133	contig_25	201543	202092	-		hypothetical protein
FUN_011136	contig_25	208864	209661	+		hypothetical protein
FUN_011137	contig_25	213162	215654	+		hypothetical protein
FUN_011141	contig_25	231630	232444	+		hypothetical protein
FUN_011142	contig_25	256500	258218	+		hypothetical protein
FUN_011146	contig_25	287475	288278	-		hypothetical protein
FUN_011148	contig_25	293865	295385	-	MET7_5	Folylpolyglutamate synthetase
FUN_011151	contig_25	304626	305018	-		hypothetical protein
FUN_011152	contig_25	308068	308868	-		hypothetical protein
FUN_011153	contig_25	326839	327854	+	KIN3_3	G2-specific serine/threonine protein kinase
FUN_011154	contig_25	340561	341119	-		hypothetical protein
FUN_011159	contig_25	376456	378495	-		hypothetical protein
FUN_011160	contig_25	379715	380850	+		hypothetical protein
FUN_011161	contig_25	386554	389159	+		hypothetical protein
FUN_011163	contig_25	398417	399127	-		hypothetical protein
FUN_011166	contig_25	435870	437779	+		hypothetical protein
FUN_011167	contig_25	437909	439121	-		hypothetical protein
FUN_011168	contig_25	448937	449575	-	RAD53_6	Protein kinase protein rad53
FUN_011170	contig_25	473498	478695	+	BLM3_7	Proteasome activator BLM10
FUN_011173	contig_26	31134	33081	+	DLD1_2	D-lactate ferricytochrome c oxidoreductase
FUN_011174	contig_26	33303	35124	+	NPR2	Nitrogen permease regulator 2
FUN_011176	contig_26	40534	42668	-		hypothetical protein
FUN_011177	contig_26	43243	45094	+		hypothetical protein
FUN_011178	contig_26	45574	46206	+		hypothetical protein
FUN_011179	contig_26	46291	47500	-		hypothetical protein
FUN_011180	contig_26	47924	49202	+		hypothetical protein
FUN_011181	contig_26	49420	50543	+		hypothetical protein
FUN_011184	contig_26	58083	60114	+		hypothetical protein
FUN_011185	contig_26	62550	64880	+	DUR3_2	urea active transporter
FUN_011186	contig_26	66750	67550	-		hypothetical protein

Gene ID	Scaffold	Start	Stop	Strand	Name	Product
FUN_011187	contig_26	68326	69533	+	RAX1	Bud site selection protein, Revert to axial protein 1
FUN_011188	contig_26	70210	71965	+	sif3	Sad1-interacting factor 3
FUN_011190	contig_26	80597	81204	+	UBC11	Ubiquitin-conjugating enzyme E2 11
FUN_011193	contig_26	83822	85569	-		hypothetical protein
FUN_011194	contig_26	89290	90555	+		hypothetical protein
FUN_011195	contig_26	90627	91274	+		hypothetical protein
FUN_011196	contig_26	93971	95296	+		hypothetical protein
FUN_011198	contig_26	98540	100264	-		hypothetical protein
FUN_011199	contig_26	102027	104302	+		hypothetical protein
FUN_011200	contig_26	106981	107575	-		hypothetical protein
FUN_011201	contig_26	111377	113690	+		hypothetical protein
FUN_011202	contig_26	114651	116874	+		hypothetical protein
FUN_011203	contig_26	117351	119570	-		hypothetical protein
FUN_011204	contig_26	119742	121640	+		hypothetical protein
FUN_011205	contig_26	122598	124332	+		hypothetical protein
FUN_011206	contig_26	128110	129570	+		hypothetical protein
FUN_011208	contig_26	132820	136117	-		hypothetical protein
FUN_011209	contig_26	137006	140847	+		hypothetical protein
FUN_011210	contig_26	142363	145806	-		hypothetical protein
FUN_011211	contig_26	146571	147380	-		hypothetical protein
FUN_011213	contig_26	150183	151313	+		hypothetical protein
FUN_011214	contig_26	151393	153491	-		hypothetical protein
FUN_011215	contig_26	156496	158050	-		hypothetical protein
FUN_011216	contig_26	159400	160400	+		hypothetical protein
FUN_011217	contig_26	160810	161606	-		hypothetical protein
FUN_011219	contig_26	165351	167149	+		hypothetical protein
FUN_011222	contig_26	171338	172078	-		hypothetical protein
FUN_011223	contig_26	173106	174777	+		hypothetical protein
FUN_011224	contig_26	175385	176464	-		hypothetical protein
FUN_011225	contig_26	177424	184673	+		hypothetical protein
FUN_011226	contig_26	185047	186894	-		hypothetical protein
FUN_011228	contig_26	191130	191484	-	TIM10	protein transporter tim10
FUN_011229	contig_26	191721	193254	+	GWT1	Glucosaminyl phosphatidylinositol (GlcN-PI) nositol acylation protein
FUN_011230	contig_26	193310	194668	-		hypothetical protein
FUN_011231	contig_26	195118	195951	+		hypothetical protein
FUN_011232	contig_26	196578	200814	-		hypothetical protein
FUN_011233	contig_26	204240	205291	+		hypothetical protein
FUN_011235	contig_26	209263	210778	-		hypothetical protein
FUN_011236	contig_26	211160	211923	+		hypothetical protein
FUN_011237	contig_26	213102	215451	+		hypothetical protein
FUN_011238	contig_26	217288	218852	-		hypothetical protein
FUN_011239	contig_26	220676	222435	-		hypothetical protein
FUN_011240	contig_26	223983	224504	-		hypothetical protein
FUN_011241	contig_26	225634	228582	-		hypothetical protein
FUN_011242	contig_26	229660	232785	+		hypothetical protein
FUN_011244	contig_26	236352	237983	-		hypothetical protein
FUN_011246	contig_26	240405	241790	-		hypothetical protein
FUN_011248	contig_26	243922	244697	-		hypothetical protein
FUN_011249	contig_26	246446	251536	-	SWR1	swr1 complex component
FUN_011250	contig_26	252098	252581	+		hypothetical protein
FUN_011252	contig_26	256232	257343	-		hypothetical protein
FUN_011253	contig_26	258212	260136	+		hypothetical protein
FUN_011254	contig_26	261945	262813	+		hypothetical protein
FUN_011255	contig_26	271672	272560	-		hypothetical protein
FUN_011256	contig_26	274787	275280	+		hypothetical protein
FUN_011257	contig_26	275937	277290	+	TGL2	lipase 2
FUN_011258	contig_26	277819	279425	+		hypothetical protein
FUN_011260	contig_26	283158	283661	+		hypothetical protein

Gene ID	Scaffold	Start	Stop	Strand	Name	Product
FUN_011261	contig_26	285270	286982	-		hypothetical protein
FUN_011262	contig_26	290343	292116	-		hypothetical protein
FUN_011263	contig_26	293187	293521	+		hypothetical protein
FUN_011265	contig_26	299524	302163	-		hypothetical protein
FUN_011266	contig_26	302631	304060	+		hypothetical protein
FUN_011269	contig_26	308830	313355	-		hypothetical protein
FUN_011270	contig_26	314748	315285	+		hypothetical protein
FUN_011271	contig_26	315995	317306	+		hypothetical protein
FUN_011273	contig_26	320748	323831	+		hypothetical protein
FUN_011274	contig_26	324087	326049	+		hypothetical protein
FUN_011277	contig_26	331754	335806	+		hypothetical protein
FUN_011278	contig_26	337614	341894	+		hypothetical protein
FUN_011279	contig_26	343043	344712	+		hypothetical protein
FUN_011283	contig_26	354006	355306	+		hypothetical protein
FUN_011284	contig_26	359355	360285	+		hypothetical protein
FUN_011285	contig_26	361375	363384	+		hypothetical protein
FUN_011286	contig_26	364163	366105	+		hypothetical protein
FUN_011287	contig_26	366911	370384	+		hypothetical protein
FUN_011288	contig_26	371106	372871	-		hypothetical protein
FUN_011292	contig_26	385119	385993	-		hypothetical protein
FUN_011293	contig_26	386829	387919	-	CRH1_2	transglycosylase
FUN_011296	contig_26	395337	396700	-		hypothetical protein
FUN_011299	contig_26	402807	403967	-		hypothetical protein
FUN_011300	contig_26	404518	406291	+		hypothetical protein
FUN_011301	contig_26	406783	407758	+		hypothetical protein
FUN_011302	contig_26	408294	410523	+		hypothetical protein
FUN_011303	contig_26	410578	411520	-		hypothetical protein
FUN_011304	contig_26	412084	413238	+		hypothetical protein
FUN_011305	contig_26	414242	416081	-		hypothetical protein
FUN_011306	contig_26	417141	418583	+		hypothetical protein
FUN_011307	contig_26	419993	421104	+		hypothetical protein
FUN_011309	contig_26	424215	426902	-		hypothetical protein
FUN_011310	contig_26	427897	429288	-		hypothetical protein
FUN_011315	contig_26	441608	443322	+		hypothetical protein
FUN_011316	contig_26	443718	445353	+		hypothetical protein
FUN_011317	contig_26	446055	448150	+		hypothetical protein
FUN_011318	contig_26	452812	453424	+		hypothetical protein
FUN_011319	contig_26	454229	455683	+		hypothetical protein
FUN_011320	contig_26	457261	458046	+		hypothetical protein
FUN_011321	contig_26	458188	459633	-		hypothetical protein
FUN_011322	contig_26	460092	461082	-		hypothetical protein
FUN_011323	contig_26	464218	465639	+		hypothetical protein
FUN_011326	contig_27	8792	10869	-		hypothetical protein
FUN_011329	contig_27	20671	21275	-		hypothetical protein
FUN_011330	contig_27	21772	23991	+		hypothetical protein
FUN_011331	contig_27	26755	28703	+		hypothetical protein
FUN_011333	contig_27	32956	34069	-		hypothetical protein
FUN_011334	contig_27	35039	38653	-	HOS3	histone deacetylase
FUN_011335	contig_27	39778	41664	+		hypothetical protein
FUN_011337	contig_27	43688	45639	-		hypothetical protein
FUN_011338	contig_27	51021	51848	+		hypothetical protein
FUN_011340	contig_27	55021	56802	-		hypothetical protein
FUN_011341	contig_27	57603	59744	+		hypothetical protein
FUN_011342	contig_27	59889	60959	-		hypothetical protein
FUN_011343	contig_27	61607	63452	-		hypothetical protein
FUN_011344	contig_27	64012	65400	+		hypothetical protein
FUN_011345	contig_27	65868	67916	+		hypothetical protein
FUN_011346	contig_27	68342	71776	+		hypothetical protein
FUN_011347	contig_27	72500	73117	-		hypothetical protein

Gene ID	Scaffold	Start	Stop	Strand	Name	Product
FUN_011348	contig_27	73791	75999	-		hypothetical protein
FUN_011349	contig_27	76776	79259	-		hypothetical protein
FUN_011350	contig_27	79702	80508	+		hypothetical protein
FUN_011351	contig_27	81004	82277	+		hypothetical protein
FUN_011352	contig_27	82509	83744	-		hypothetical protein
FUN_011353	contig_27	84020	85765	-		hypothetical protein
FUN_011354	contig_27	86382	87563	-		hypothetical protein
FUN_011355	contig_27	87876	89946	-		hypothetical protein
FUN_011357	contig_27	94568	95790	-		hypothetical protein
FUN_011358	contig_27	96876	97874	-		hypothetical protein
FUN_011359	contig_27	98944	100566	+	APE3	Aminopeptidase Y
FUN_011360	contig_27	100990	102607	-		hypothetical protein
FUN_011365	contig_27	115281	118725	-	CHS2	Chitin synthase, class 2
FUN_011366	contig_27	119193	120844	-		hypothetical protein
FUN_011367	contig_27	121674	122142	-		hypothetical protein
FUN_011368	contig_27	123315	125984	+		hypothetical protein
FUN_011369	contig_27	130150	131148	-		hypothetical protein
FUN_011370	contig_27	131659	131871	+		hypothetical protein
FUN_011371	contig_27	132250	133905	-		hypothetical protein
FUN_011372	contig_27	134916	137184	+		hypothetical protein
FUN_011375	contig_27	142051	143080	-		hypothetical protein
FUN_011376	contig_27	143393	144429	-	AYR1_2	NADPH-dependent 1-acyl dihydroxyacetone phosphate reductase
FUN_011377	contig_27	148522	149291	+		hypothetical protein
FUN_011381	contig_27	170427	172034	-		hypothetical protein
FUN_011382	contig_27	172487	173350	-		hypothetical protein
FUN_011383	contig_27	173854	177597	+	BBC1	assembly of actin patch protein
FUN_011387	contig_27	187957	189870	+		hypothetical protein
FUN_011391	contig_27	217545	218360	-		hypothetical protein
FUN_011395	contig_27	227057	228168	+		hypothetical protein
FUN_011396	contig_27	230344	231397	-		hypothetical protein
FUN_011398	contig_27	235934	236809	-		hypothetical protein
FUN_011399	contig_27	239354	240523	+		hypothetical protein
FUN_011400	contig_27	242014	242867	+		hypothetical protein
FUN_011401	contig_27	243276	244209	-		hypothetical protein
FUN_011403	contig_27	247721	248711	-		hypothetical protein
FUN_011404	contig_27	249312	251170	-		hypothetical protein
FUN_011405	contig_27	255457	256935	-		hypothetical protein
FUN_011406	contig_27	257361	259821	+	ORC4	origin recognition complex subunit 4
FUN_011407	contig_27	260816	262058	-		hypothetical protein
FUN_011408	contig_27	270267	273188	+	FOX2	bifunctional hydroxyacyl-CoA dehydrogenase/enoyl-CoA hydratase fox2
FUN_011409	contig_27	273507	275036	+		hypothetical protein
FUN_011410	contig_27	277030	279825	-		hypothetical protein
FUN_011411	contig_27	282979	286291	-	PIM1	ATP-dependent Lon protease pim1
FUN_011412	contig_27	288082	289057	+		hypothetical protein
FUN_011414	contig_27	289794	290642	-		hypothetical protein
FUN_011415	contig_27	292168	293696	+		hypothetical protein
FUN_011416	contig_27	294174	294819	+	RPS17B	40S ribosomal protein S17.e.B
FUN_011418	contig_27	298427	301818	-		hypothetical protein
FUN_011419	contig_27	302312	303425	-		hypothetical protein
FUN_011421	contig_27	310518	311652	-		hypothetical protein
FUN_011422	contig_27	313430	314667	+	DDI1	DNA damage-inducible protein 1
FUN_011423	contig_27	316480	318111	-	RRP8	25S rRNA (adenine645-N1)-methyltransferase
FUN_011426	contig_27	323091	323658	-	KRE9_1	Cell wall synthesis protein kre9 precursor
FUN_011427	contig_27	323737	324128	-	KRE9_2	Cell wall synthesis protein kre9 precursor
FUN_011428	contig_27	324803	325678	-		hypothetical protein
FUN_011429	contig_27	327964	330445	+		hypothetical protein
FUN_011431	contig_27	332573	333918	-		hypothetical protein

Gene ID	Scaffold	Start	Stop	Strand	Name	Product
FUN_011432	contig_27	338726	341915	-	MTR10	Nuclear import receptor
FUN_011433	contig_27	342331	342759	+	SME1	mRNA splicing protein sme1
FUN_011434	contig_27	342888	343695	-		hypothetical protein
FUN_011435	contig_27	344076	344965	+	ROT1	Reversal of tor2 lethality
FUN_011436	contig_27	345630	345815	+		hypothetical protein
FUN_011437	contig_27	345925	347261	+		hypothetical protein
FUN_011439	contig_27	353050	353521	-		hypothetical protein
FUN_011440	contig_27	354999	358170	-	BRO1	bck1-like resistance to osmotic shock
FUN_011441	contig_27	359182	360823	-	MDJ1	mdj1 protein precursor
FUN_011444	contig_27	366699	370660	-		hypothetical protein
FUN_011445	contig_27	379721	381219	-	STR3	cystathionine beta-lyase
FUN_011446	contig_27	381792	384038	-		hypothetical protein
FUN_011447	contig_27	384691	386815	+	SSC1	Hsp70 ATPase ssc1
FUN_011448	contig_27	388382	390038	-	GLO3_1	ADP-ribosylation factor GTPase activating protein, ER-Golgi transport
FUN_011449	contig_27	390528	391315	+		hypothetical protein
FUN_011450	contig_27	391337	393421	-	GLO3_2	ADP-ribosylation factor GTPase activating protein, ER-Golgi transport
FUN_011451	contig_27	394232	396285	+	NOG1	Nucleolar GTP-binding protein 1
FUN_011453	contig_27	400465	401215	-	TIM23	Mitochondrial import inner membrane translocase subunit tim23
FUN_011454	contig_27	401715	402623	+	PYR1	Orotidine 5'-phosphate decarboxylase
FUN_011455	contig_27	403295	405124	+		hypothetical protein
FUN_011456	contig_27	405301	407252	-		hypothetical protein
FUN_011458	contig_27	410966	412856	+		hypothetical protein
FUN_011459	contig_27	413320	414586	-		hypothetical protein
FUN_011460	contig_27	416640	417779	-		hypothetical protein
FUN_011462	contig_27	420687	421454	-		hypothetical protein
FUN_011463	contig_27	422093	424121	+	MET13	methylenetetrahydrofolate reductase (NAD(P)H) met13
FUN_011464	contig_27	425002	427300	+		hypothetical protein
FUN_011465	contig_27	427360	428022	-		hypothetical protein
FUN_011466	contig_27	428685	432554	+		hypothetical protein
FUN_011468	contig_28	15900	17721	+	GGA2	ARF-binding protein
FUN_011471	contig_28	36025	36891	-		hypothetical protein
FUN_011473	contig_28	39452	40348	+	JID1	J domain-containing protein 1
FUN_011474	contig_28	41467	43200	-	CHT4_2	Chitinase 4
FUN_011475	contig_28	44771	48883	+		hypothetical protein
FUN_011476	contig_28	49700	51356	-		hypothetical protein
FUN_011477	contig_28	52042	54774	+	ACO1	Aconitate hydratase mitochondrial
FUN_011478	contig_28	55360	57438	-		hypothetical protein
FUN_011479	contig_28	58695	59714	-		hypothetical protein
FUN_011480	contig_28	59835	61285	+	COG5	Conserved oligomeric Golgi complex subunit
FUN_011481	contig_28	62427	64370	+	VTS1	Flap-structured DNA-binding and RNA-binding protein
FUN_011482	contig_28	65082	66279	+	BNA6_1	nicotinate-nucleotide diphosphorylase (carboxylating)
FUN_011483	contig_28	68777	71061	+		hypothetical protein
FUN_011484	contig_28	71391	73892	-	THS1	threonyl-tRNA synthetase
FUN_011485	contig_28	74231	75124	+	GAR1	H/ACA snoRNP pseudouridylase subunit
FUN_011486	contig_28	75706	77033	+	CHO1	CDP-diacylglycerol-serine O-phosphatidyltransferase
FUN_011487	contig_28	77378	79921	-		hypothetical protein
FUN_011488	contig_28	81113	82902	+		hypothetical protein
FUN_011490	contig_28	86860	88600	-	LCB1	serine palmitoyltransferase component
FUN_011491	contig_28	89093	95023	-	FKS1	1,3-beta-D-glucan synthase
FUN_011492	contig_28	97954	99670	+		hypothetical protein
FUN_011493	contig_28	99956	100705	-		hypothetical protein
FUN_011494	contig_28	101697	104632	+		hypothetical protein
FUN_011496	contig_28	106745	108348	-		hypothetical protein
FUN_011497	contig_28	109210	110464	-		hypothetical protein
FUN_011498	contig_28	111759	113545	-		hypothetical protein
FUN_011499	contig_28	115194	116896	-	SDS23	cell separation during budding

Gene ID	Scaffold	Start	Stop	Strand	Name	Product
FUN_011500	contig_28	117635	119225	-		hypothetical protein
FUN_011501	contig_28	120856	122835	+		hypothetical protein
FUN_011502	contig_28	124514	125364	+		hypothetical protein
FUN_011503	contig_28	125757	126704	+	BNA6_2	nicotinate-nucleotide diphosphorylase (carboxylating)
FUN_011505	contig_28	128383	129783	-	INT6	eukaryotic translation initiation factor 3 subunit E
FUN_011506	contig_28	130050	131258	+	img2	54S ribosomal protein img2, mitochondrial
FUN_011508	contig_28	139967	144744	-		hypothetical protein
FUN_011509	contig_28	146428	148514	-		hypothetical protein
FUN_011510	contig_28	150289	151725	-	RBT7	Ribonuclease T2 precursor (RNase T2)
FUN_011511	contig_28	152557	155482	-		hypothetical protein
FUN_011512	contig_28	156169	158050	-	CCT6	T-complex protein 1 subunit zeta
FUN_011513	contig_28	158370	160535	+	nop56	Nucleolar protein 56
FUN_011514	contig_28	163793	165397	-	CYP8B1	sterol 12-alpha-hydroxylase activity
FUN_011515	contig_28	166016	167654	+	SPT10_2	Protein spt10
FUN_011516	contig_28	168359	169544	-		hypothetical protein
FUN_011517	contig_28	169886	171316	-		hypothetical protein
FUN_011521	contig_28	176815	178622	-	HPC2_8	HIR complex subunit
FUN_011522	contig_28	180853	182163	+		hypothetical protein
FUN_011523	contig_28	182539	184228	+		hypothetical protein
FUN_011524	contig_28	185291	186070	-	MTQ2	S-adenosylmethionine-dependent methyltransferase
FUN_011525	contig_28	186323	186807	+		hypothetical protein
FUN_011527	contig_28	188299	188934	+		hypothetical protein
FUN_011528	contig_28	189468	190940	+		hypothetical protein
FUN_011529	contig_28	191794	192745	-		hypothetical protein
FUN_011531	contig_28	196865	198356	+		hypothetical protein
FUN_011532	contig_28	198731	202257	+		hypothetical protein
FUN_011535	contig_28	209526	212630	+	SRO7	Lethal(2) giant larvae sro7
FUN_011536	contig_28	213232	214494	+		hypothetical protein
FUN_011537	contig_28	214959	215516	-	smd1	Sm snRNP core protein Smd1
FUN_011538	contig_28	216354	217430	+		hypothetical protein
FUN_011539	contig_28	217916	219353	-		hypothetical protein
FUN_011540	contig_28	220220	221734	+		hypothetical protein
FUN_011541	contig_28	222066	223883	-		hypothetical protein
FUN_011542	contig_28	225245	233352	+		hypothetical protein
FUN_011544	contig_28	235052	235648	-		hypothetical protein
FUN_011545	contig_28	236134	242939	+	POL2	DNA polymerase epsilon catalytic subunit
FUN_011546	contig_28	243247	245761	-	RRP6	exosome nuclease subunit
FUN_011547	contig_28	246014	246452	+	MDM35	Mitochondrial distribution and morphology protein 35
FUN_011548	contig_28	246865	247525	+		hypothetical protein
FUN_011549	contig_28	248241	249725	+		hypothetical protein
FUN_011550	contig_28	250065	251555	-		hypothetical protein
FUN_011551	contig_28	252246	253949	+		hypothetical protein
FUN_011552	contig_28	255491	256271	-		hypothetical protein
FUN_011553	contig_28	257858	259852	-		hypothetical protein
FUN_011554	contig_28	261457	266829	+	BCK1	mitogen-activated protein kinase kinase kinase
FUN_011556	contig_28	270982	273153	+		hypothetical protein
FUN_011558	contig_28	279421	280930	-		hypothetical protein
FUN_011559	contig_28	281928	282912	-		hypothetical protein
FUN_011560	contig_28	283718	285297	+		hypothetical protein
FUN_011562	contig_28	287520	289143	-	gpi1	pig-Q
FUN_011563	contig_28	290388	292453	+		hypothetical protein
FUN_011564	contig_28	292848	293396	-		hypothetical protein
FUN_011565	contig_28	294948	296239	-		hypothetical protein
FUN_011569	contig_28	303780	310872	-		hypothetical protein
FUN_011570	contig_28	312625	313565	+		hypothetical protein
FUN_011571	contig_28	314526	319160	+		hypothetical protein
FUN_011572	contig_28	322374	323174	+		hypothetical protein
FUN_011573	contig_28	323872	324738	+		hypothetical protein
FUN_011574	contig_28	324948	326081	-		hypothetical protein

Gene ID	Scaffold	Start	Stop	Strand	Name	Product
FUN_011575	contig_28	331378	332493	-	ANT1	ADP/ATP carrier protein
FUN_011576	contig_28	333163	336470	+	RIM13	cysteine protease
FUN_011577	contig_28	337277	339040	+		hypothetical protein
FUN_011578	contig_28	341962	343002	-		hypothetical protein
FUN_011579	contig_28	345625	346301	+	LPMO9T	AA9 lytic polysaccharide monooxygenase T
FUN_011580	contig_28	349583	351460	-		hypothetical protein
FUN_011581	contig_28	351834	355364	+		hypothetical protein
FUN_011582	contig_28	355976	357580	+		hypothetical protein
FUN_011583	contig_28	357816	361832	-		hypothetical protein
FUN_011584	contig_28	362964	364568	+		hypothetical protein
FUN_011585	contig_28	365304	367135	-		hypothetical protein
FUN_011586	contig_28	368059	369267	-		hypothetical protein
FUN_011587	contig_28	370155	371416	+		hypothetical protein
FUN_011589	contig_28	373762	375707	-		hypothetical protein
FUN_011590	contig_28	377267	380937	+	ENA2_2	P-type ATPase
FUN_011591	contig_28	381260	381834	+	HNT1	Adenosine 5'-monophosphoramidase
FUN_011592	contig_28	381898	382845	-		hypothetical protein
FUN_011594	contig_28	384916	386256	-	MTM1	Carrier protein, mitochondrial
FUN_011595	contig_28	386767	388359	+		hypothetical protein
FUN_011596	contig_28	388689	390362	-	CCT7	T-complex protein 1 subunit eta
FUN_011598	contig_28	392152	393492	-		hypothetical protein
FUN_011599	contig_28	395113	395571	-	RPS20	40S ribosomal protein S20
FUN_011600	contig_28	395852	397892	+		hypothetical protein
FUN_011601	contig_28	399470	400599	-		hypothetical protein
FUN_011602	contig_28	401731	402785	-		hypothetical protein
FUN_011604	contig_28	407002	408357	-		hypothetical protein
FUN_011605	contig_29	8960	14482	-		hypothetical protein
FUN_011606	contig_29	15233	16847	-		hypothetical protein
FUN_011607	contig_29	17564	19101	-		hypothetical protein
FUN_011608	contig_29	20163	20741	+		hypothetical protein
FUN_011609	contig_29	23040	23953	+		hypothetical protein
FUN_011610	contig_29	27450	27941	-		hypothetical protein
FUN_011614	contig_29	39659	40924	+		hypothetical protein
FUN_011615	contig_29	41061	42395	-		hypothetical protein
FUN_011616	contig_29	42770	45424	-		hypothetical protein
FUN_011623	contig_29	64088	64909	+	TMA20	translation machinery-associated protein 20
FUN_011624	contig_29	65041	66275	-	sgt2	Small glutamine-rich tetratricopeptide repeat-containing protein 2
FUN_011625	contig_29	66673	68092	-		hypothetical protein
FUN_011626	contig_29	69154	74356	+		hypothetical protein
FUN_011630	contig_29	80276	82395	+		hypothetical protein
FUN_011631	contig_29	82666	84003	-	SLP2	Synaptotagmin-like protein 2
FUN_011636	contig_29	94076	97633	+		hypothetical protein
FUN_011637	contig_29	98473	99430	+		hypothetical protein
FUN_011638	contig_29	99847	100377	-		hypothetical protein
FUN_011639	contig_29	101491	103707	-		hypothetical protein
FUN_011640	contig_29	106380	108215	-	TIM44	protein translocase subunit
FUN_011641	contig_29	108327	108963	+		hypothetical protein
FUN_011642	contig_29	109180	110085	-		hypothetical protein
FUN_011643	contig_29	110631	112118	+	OLA1	Obg-like ATPase
FUN_011644	contig_29	112344	115392	-	sin4	Mediator of RNA polymerase II transcription subunit 16
FUN_011645	contig_29	116418	118602	+		hypothetical protein
FUN_011646	contig_29	119023	120603	+		hypothetical protein
FUN_011649	contig_29	127838	128654	-		hypothetical protein
FUN_011651	contig_29	130800	132720	-		hypothetical protein
FUN_011653	contig_29	133799	135356	-		hypothetical protein
FUN_011656	contig_29	140016	141673	+		hypothetical protein
FUN_011657	contig_29	143648	145084	-		hypothetical protein
FUN_011658	contig_29	145916	149510	-		hypothetical protein

Gene ID	Scaffold	Start	Stop	Strand	Name	Product
FUN_011659	contig_29	150404	151534	-		hypothetical protein
FUN_011660	contig_29	151740	152532	+	GIR2	Protein gir2
FUN_011661	contig_29	152611	153551	-		hypothetical protein
FUN_011662	contig_29	153931	154544	-	MMS2	E2 ubiquitin-conjugating protein mms2
FUN_011663	contig_29	154990	160277	+	MON2	Endocytosis and vacuole integrity protein
FUN_011664	contig_29	161008	162422	+		hypothetical protein
FUN_011665	contig_29	163865	166903	-		hypothetical protein
FUN_011666	contig_29	169731	172859	+		hypothetical protein
FUN_011667	contig_29	176569	179556	+		hypothetical protein
FUN_011668	contig_29	182756	186628	+		hypothetical protein
FUN_011670	contig_29	192594	193943	-		hypothetical protein
FUN_011672	contig_29	197356	198177	-	wos2	p23 chaperone protein wos2
FUN_011673	contig_29	198690	199422	+	VPS24	Vacuolar protein-sorting-associated protein 24
FUN_011674	contig_29	199587	200360	-		hypothetical protein
FUN_011675	contig_29	200661	201573	+		hypothetical protein
FUN_011676	contig_29	203786	204997	+		hypothetical protein
FUN_011677	contig_29	205365	206913	+		hypothetical protein
FUN_011678	contig_29	207277	208761	-		hypothetical protein
FUN_011679	contig_29	210711	212669	+		hypothetical protein
FUN_011680	contig_29	213284	213892	+	FRM2	type II nitroreductase
FUN_011682	contig_29	222483	223912	-		hypothetical protein
FUN_011685	contig_29	228176	232030	-		hypothetical protein
FUN_011686	contig_29	233083	234304	-		hypothetical protein
FUN_011687	contig_29	235062	236514	+		hypothetical protein
FUN_011688	contig_29	238052	240574	+		hypothetical protein
FUN_011690	contig_29	246482	248777	-	cwf19	Pre-mRNA-splicing factor cwf19
FUN_011691	contig_29	249091	250404	+		hypothetical protein
FUN_011692	contig_29	255748	256692	-		hypothetical protein
FUN_011693	contig_29	264472	269741	-		hypothetical protein
FUN_011694	contig_29	272148	274654	-	PRP40	U1 snRNP protein
FUN_011695	contig_29	274912	276788	+		hypothetical protein
FUN_011696	contig_29	277271	278387	+		hypothetical protein
FUN_011697	contig_29	279055	280440	+	PGK1	phosphoglycerate kinase
FUN_011698	contig_29	280774	281201	-	SEM1	26S proteasome complex subunit
FUN_011699	contig_29	282008	284472	+	IML2	Mitochondrial outer membrane protein iml2
FUN_011700	contig_29	286351	287405	+		hypothetical protein
FUN_011701	contig_29	292399	294567	-	RLI1	Fe-S cluster-binding ribosome biosynthesis protein
FUN_011702	contig_29	295471	300619	+	hrp3	ATP-dependent DNA helicase Hrp3
FUN_011703	contig_29	301398	303730	+	PPN1	Endopolyphosphatase
FUN_011704	contig_29	304721	305916	+		hypothetical protein
FUN_011705	contig_29	306276	308126	-	UME6_2	DNA-binding transcriptional regulator ume6
FUN_011706	contig_29	308692	310877	+		hypothetical protein
FUN_011707	contig_29	311475	314255	+		hypothetical protein
FUN_011708	contig_29	314808	315721	-		hypothetical protein
FUN_011709	contig_29	319439	321338	+		hypothetical protein
FUN_011710	contig_29	321455	322475	-		hypothetical protein
FUN_011711	contig_29	323081	324575	-	MNN2	mannosyltransferase
FUN_011712	contig_29	326074	326997	+		hypothetical protein
FUN_011714	contig_29	336627	337989	-		hypothetical protein
FUN_011715	contig_29	342831	343592	-		hypothetical protein
FUN_011716	contig_29	344687	346489	-		hypothetical protein
FUN_011717	contig_29	346870	355076	-		hypothetical protein
FUN_011718	contig_29	356927	357549	+		hypothetical protein
FUN_011719	contig_29	357845	361129	-		hypothetical protein
FUN_011720	contig_29	361474	363090	+		hypothetical protein
FUN_011722	contig_29	366291	368125	-		hypothetical protein
FUN_011724	contig_29	369922	371213	-	TRP4	anthranilate phosphoribosyltransferase
FUN_011725	contig_29	371617	374379	+	MAK21	RNA-binding ribosome biosynthesis protein mak21
FUN_011726	contig_29	374795	376087	+		hypothetical protein

Gene ID	Scaffold	Start	Stop	Strand	Name	Product
FUN_011727	contig_29	376758	380509	+	npr3	Nitrogen permease regulator 3
FUN_011729	contig_29	385301	385903	-		hypothetical protein
FUN_011732	contig_30	10108	11445	+		hypothetical protein
FUN_011733	contig_30	11773	12382	-	ubc7	ubiquitin conjugating enzyme Ubc7/UbcP3
FUN_011734	contig_30	12938	14647	-		hypothetical protein
FUN_011735	contig_30	15280	16605	+	SEN2	tRNA splicing endonuclease subunit sen2
FUN_011740	contig_30	25153	26717	+		hypothetical protein
FUN_011741	contig_30	27989	29642	-		hypothetical protein
FUN_011742	contig_30	30306	36006	-		hypothetical protein
FUN_011744	contig_30	38695	40655	-		hypothetical protein
FUN_011747	contig_30	55748	58072	-		hypothetical protein
FUN_011748	contig_30	59284	60664	-	SHP1_2	protein phosphatase regulator
FUN_011749	contig_30	61175	61626	+		hypothetical protein
FUN_011750	contig_30	61868	63347	-		hypothetical protein
FUN_011751	contig_30	71108	72122	-	CDC8	Thymidylate kinase
FUN_011752	contig_30	72903	74201	+		hypothetical protein
FUN_011753	contig_30	74241	75917	-		hypothetical protein
FUN_011754	contig_30	76849	79198	+		hypothetical protein
FUN_011755	contig_30	80933	83699	-		hypothetical protein
FUN_011756	contig_30	84775	86225	+		hypothetical protein
FUN_011758	contig_30	90115	92460	+	ERG7A	Lanosterol synthase erg7A
FUN_011759	contig_30	93540	94322	-		hypothetical protein
FUN_011760	contig_30	94353	95122	-		hypothetical protein
FUN_011761	contig_30	96097	97139	+		hypothetical protein
FUN_011762	contig_30	98173	102203	-		hypothetical protein
FUN_011763	contig_30	107273	109515	-		hypothetical protein
FUN_011764	contig_30	111538	114346	+		hypothetical protein
FUN_011766	contig_30	119320	120657	+	nob1	20S-pre-rRNA D-site endonuclease nob1
FUN_011767	contig_30	120842	121831	-		hypothetical protein
FUN_011768	contig_30	122192	122997	+		hypothetical protein
FUN_011769	contig_30	125114	127230	+		hypothetical protein
FUN_011772	contig_30	137398	139972	-	CYR1	cysteinyl-tRNA synthetase
FUN_011773	contig_30	140295	141869	+	TRM61	tRNA (adenine-N(1)-)-methyltransferase catalytic subunit trm61
FUN_011774	contig_30	143874	146498	-		hypothetical protein
FUN_011775	contig_30	149443	150601	+		hypothetical protein
FUN_011776	contig_30	150714	151639	-		hypothetical protein
FUN_011777	contig_30	152181	153036	-	MRPL2	54S ribosomal protein L2 mitochondrial
FUN_011779	contig_30	158665	161020	+	VMA1	H(+)-transporting V1 sector ATPase subunit A
FUN_011780	contig_30	161400	163896	+	VPS1	vacuolar protein sorting-associated protein 1
FUN_011781	contig_30	164464	165338	+		hypothetical protein
FUN_011782	contig_30	165393	165937	-		hypothetical protein
FUN_011783	contig_30	166703	170216	+	STE23_2	metalloprotease
FUN_011785	contig_30	172470	173174	+		hypothetical protein
FUN_011786	contig_30	173878	174630	+		hypothetical protein
FUN_011787	contig_30	175485	176080	-		hypothetical protein
FUN_011788	contig_30	176558	177377	-		hypothetical protein
FUN_011789	contig_30	177954	179444	+		hypothetical protein
FUN_011790	contig_30	186336	187329	-		hypothetical protein
FUN_011796	contig_30	196928	197347	+		hypothetical protein
FUN_011798	contig_30	202710	207138	+	YOR1_2	ATP-binding cassette transporter yor1
FUN_011799	contig_30	207424	209301	-		hypothetical protein
FUN_011800	contig_30	210392	212053	+		hypothetical protein
FUN_011804	contig_30	225321	228074	-	PWP2	U3 snoRNP protein
FUN_011805	contig_30	228457	229853	+	spn4	Septin spn4
FUN_011806	contig_30	233866	236973	+		hypothetical protein
FUN_011811	contig_30	253492	254429	+		hypothetical protein
FUN_011812	contig_30	255340	257889	+		hypothetical protein
FUN_011813	contig_30	258880	259103	+	NOP10	snoRNP complex protein

Gene ID	Scaffold	Start	Stop	Strand	Name	Product
FUN_011815	contig_30	263295	264518	+		hypothetical protein
FUN_011816	contig_30	265956	266404	+		hypothetical protein
FUN_011817	contig_30	267113	274064	+		hypothetical protein
FUN_011818	contig_30	274305	274922	-		hypothetical protein
FUN_011820	contig_30	280942	282179	-		hypothetical protein
FUN_011822	contig_30	290699	292493	-		hypothetical protein
FUN_011823	contig_30	293253	295058	-		hypothetical protein
FUN_011824	contig_30	295448	296230	+		hypothetical protein
FUN_011825	contig_30	296647	297259	-		hypothetical protein
FUN_011826	contig_30	299223	300518	+		hypothetical protein
FUN_011827	contig_30	300959	302568	+	apm1	AP-1 adaptor complex mu subunit Apm1
FUN_011828	contig_30	303693	305059	+		hypothetical protein
FUN_011829	contig_30	306397	308847	-		hypothetical protein
FUN_011830	contig_30	309808	310706	-		hypothetical protein
FUN_011831	contig_30	311054	313444	-	SSL2	DNA repair helicase RAD25
FUN_011832	contig_30	313890	319528	+		hypothetical protein
FUN_011833	contig_30	321028	322593	+	SPE2	spermidine resistance protein
FUN_011834	contig_30	323495	325037	+	YOS9_2	Protein OS-9
FUN_011835	contig_30	325661	329857	-	png1_2	Protein png1
FUN_011837	contig_30	331887	332999	-		hypothetical protein
FUN_011838	contig_30	333699	334708	+		hypothetical protein
FUN_011841	contig_30	340329	341446	+		hypothetical protein
FUN_011842	contig_30	341653	343308	-		hypothetical protein
FUN_011843	contig_30	343710	345408	-		hypothetical protein
FUN_011844	contig_30	347306	349123	-	LAP4	vacuolar aminopeptidase 1
FUN_011845	contig_30	350040	350959	+		hypothetical protein
FUN_011846	contig_30	352283	353304	+		hypothetical protein
FUN_011847	contig_30	354873	358245	+		hypothetical protein
FUN_011848	contig_30	358635	359583	-		hypothetical protein
FUN_011849	contig_30	360313	361008	+		hypothetical protein
FUN_011853	contig_31	15149	16333	+	TSR3	ribosome bioproteinsis protein tsr3
FUN_011854	contig_31	16829	18644	+		hypothetical protein
FUN_011857	contig_31	24815	25789	+		hypothetical protein
FUN_011858	contig_31	25926	26741	-		hypothetical protein
FUN_011859	contig_31	26954	27745	+		hypothetical protein
FUN_011860	contig_31	29100	30802	-		hypothetical protein
FUN_011862	contig_31	40706	44823	-		hypothetical protein
FUN_011863	contig_31	45123	47012	+		hypothetical protein
FUN_011864	contig_31	47515	53679	-	APC1	Anaphase-promoting complex subunit 1
FUN_011868	contig_31	60173	62385	-	EXO1	Rad2 nuclease
FUN_011869	contig_31	62733	63327	+	TMA16	translation machinery-associated protein 16
FUN_011873	contig_31	69794	72540	+		hypothetical protein
FUN_011874	contig_31	73358	74515	-		hypothetical protein
FUN_011875	contig_31	75142	76133	+	MAM33	Mitochondrial acidic protein mam33
FUN_011876	contig_31	76526	77613	-		hypothetical protein
FUN_011877	contig_31	78584	80031	-	PBP2	PAB1 binding protein
FUN_011879	contig_31	86441	89288	-		hypothetical protein
FUN_011882	contig_31	99754	100987	-	SEC14	cytosolic factor, phosphatidylinositol/phosphatidylcholine transfer protein
FUN_011883	contig_31	101824	104158	-	CCR4	Glucose-repressible alcohol dehydrogenase transcriptional effector
FUN_011884	contig_31	105404	106794	+		hypothetical protein
FUN_011885	contig_31	107776	108974	+		hypothetical protein
FUN_011886	contig_31	110421	113430	+		hypothetical protein
FUN_011887	contig_31	114894	116213	-		hypothetical protein
FUN_011888	contig_31	119407	119934	-		hypothetical protein
FUN_011889	contig_31	122857	123647	+		hypothetical protein
FUN_011890	contig_31	124299	125806	+	ADE12	Adenylosuccinate synthase
FUN_011891	contig_31	126422	127939	-	CSR1_1	phosphatidylinositol transfer protein csr1

Gene ID	Scaffold	Start	Stop	Strand	Name	Product
FUN_011892	contig_31	128824	129194	+	DAM1	DASH complex subunit dam1
FUN_011893	contig_31	130175	132115	-		hypothetical protein
FUN_011894	contig_31	134642	135616	-		hypothetical protein
FUN_011895	contig_31	136433	137383	+		hypothetical protein
FUN_011896	contig_31	139725	140630	-	wsc1	Protein SLG1
FUN_011898	contig_31	145395	146069	-		hypothetical protein
FUN_011899	contig_31	146561	147843	+		hypothetical protein
FUN_011901	contig_31	150554	151190	-		hypothetical protein
FUN_011902	contig_31	151378	152678	+		hypothetical protein
FUN_011903	contig_31	153100	156771	+		hypothetical protein
FUN_011904	contig_31	159727	161381	+		hypothetical protein
FUN_011907	contig_31	164250	165216	-	YMC1	carrier protein ymc1
FUN_011908	contig_31	166051	167111	-		hypothetical protein
FUN_011909	contig_31	169082	172431	-		hypothetical protein
FUN_011910	contig_31	173543	174905	+	MRP4	37S ribosomal protein, mitochondrial
FUN_011911	contig_31	175745	177944	-		hypothetical protein
FUN_011912	contig_31	179200	180945	-	VAMP8	Vesicle-Associated Membrane Protein 8
FUN_011913	contig_31	181245	185743	+		hypothetical protein
FUN_011916	contig_31	189616	190209	-		hypothetical protein
FUN_011917	contig_31	190731	192701	-		hypothetical protein
FUN_011918	contig_31	193797	196243	+		hypothetical protein
FUN_011920	contig_31	202280	203208	-		hypothetical protein
FUN_011921	contig_31	203393	206011	-		hypothetical protein
FUN_011922	contig_31	206426	208416	-	PHO5_2	acid phosphatase pho5
FUN_011926	contig_31	216189	218584	+	prh1	Salivary acidic proline-rich phosphoprotein 1/2
FUN_011927	contig_31	218761	220827	-	KEL3	Kelch repeat-containing protein 3
FUN_011928	contig_31	221190	222619	+		hypothetical protein
FUN_011929	contig_31	223186	223653	-		hypothetical protein
FUN_011930	contig_31	224081	225735	+		hypothetical protein
FUN_011931	contig_31	226625	228113	+	cmk1	Calcium/calmodulin-dependent protein kinase type I
FUN_011932	contig_31	228562	229865	-		hypothetical protein
FUN_011933	contig_31	230762	232158	+		hypothetical protein
FUN_011935	contig_31	237044	238427	-		hypothetical protein
FUN_011937	contig_31	243423	244765	-		hypothetical protein
FUN_011938	contig_31	245275	245961	-	URE2_3	Transcriptional regulator ure2
FUN_011940	contig_31	252578	253833	-		hypothetical protein
FUN_011941	contig_31	254321	255537	+		hypothetical protein
FUN_011943	contig_31	257861	259322	-		hypothetical protein
FUN_011944	contig_31	260060	262043	+		hypothetical protein
FUN_011945	contig_31	262070	263283	-		hypothetical protein
FUN_011946	contig_31	265692	266783	+		hypothetical protein
FUN_011948	contig_31	276192	278015	-	HEX1	woronin body major protein
FUN_011949	contig_31	279616	280917	-		hypothetical protein
FUN_011950	contig_31	281329	281978	-	RPL12	60S ribosomal protein L12
FUN_011952	contig_31	292946	294739	+		hypothetical protein
FUN_011955	contig_31	302445	303561	+		hypothetical protein
FUN_011956	contig_31	305406	306164	-		hypothetical protein
FUN_011959	contig_31	310687	312380	+	TPN1_2	Vitamin B6 transporter
FUN_011960	contig_31	312863	313925	-		hypothetical protein
FUN_011961	contig_31	318523	319749	-		hypothetical protein
FUN_011962	contig_31	320334	321731	+		hypothetical protein
FUN_011963	contig_31	327281	327772	-		hypothetical protein
FUN_011964	contig_31	331123	331805	+		hypothetical protein
FUN_011965	contig_31	341050	342087	-		hypothetical protein
FUN_011966	contig_31	342510	342929	-		hypothetical protein
FUN_011967	contig_31	344066	345442	-		hypothetical protein
FUN_011968	contig_31	346502	348040	-		hypothetical protein
FUN_011969	contig_31	349990	351866	-		hypothetical protein
FUN_011970	contig_31	355964	357190	-		hypothetical protein

Gene ID	Scaffold	Start	Stop	Strand	Name	Product
FUN_011971	contig_31	358665	360054	+		hypothetical protein
FUN_011973	contig_31	361717	362853	-		hypothetical protein
FUN_011974	contig_31	363371	364468	-		hypothetical protein
FUN_011975	contig_31	365056	367757	+		hypothetical protein
FUN_011977	contig_32	16	369	-		hypothetical protein
FUN_011979	contig_32	20094	21736	-		hypothetical protein
FUN_011980	contig_32	21987	23873	+		hypothetical protein
FUN_011981	contig_32	24902	26559	+		hypothetical protein
FUN_011982	contig_32	31063	32021	+	PGX1	Exopolygalacturonase
FUN_011983	contig_32	32290	34446	-		hypothetical protein
FUN_011984	contig_32	41462	42872	-		hypothetical protein
FUN_011985	contig_32	45950	46736	+		hypothetical protein
FUN_011987	contig_32	49816	51551	+		hypothetical protein
FUN_011989	contig_32	53243	55288	-	RPD3_3	histone deacetylase
FUN_011990	contig_32	55672	56930	+	RPN11	multicatalytic endopeptidase
FUN_011991	contig_32	57202	58777	-		hypothetical protein
FUN_011992	contig_32	60850	63863	-	hmt1	ATP-binding cassette-type vacuolar membrane transporter Hmt1
FUN_011993	contig_32	64394	69092	+	RTC1	SEA (Seh1-associated) complex subunit
FUN_011994	contig_32	72008	72372	-	RPS21	40S ribosomal protein S21
FUN_011996	contig_32	74742	75371	-		hypothetical protein
FUN_011997	contig_32	76964	78418	-	UTR2	putative glycosidase CRH2
FUN_011998	contig_32	79521	81398	-	IMD1	inosine-5'-monophosphate dehydrogenase
FUN_011999	contig_32	82701	87931	+	IQG1	iqgap-related protein
FUN_012000	contig_32	88398	89354	-		hypothetical protein
FUN_012001	contig_32	89626	91674	-		hypothetical protein
FUN_012005	contig_32	101530	102232	+		hypothetical protein
FUN_012007	contig_32	104179	105520	-		hypothetical protein
FUN_012008	contig_32	106654	108907	+		hypothetical protein
FUN_012009	contig_32	110999	112389	-		hypothetical protein
FUN_012010	contig_32	116190	117589	+		hypothetical protein
FUN_012014	contig_32	126586	128052	+		hypothetical protein
FUN_012015	contig_32	128188	129600	-		hypothetical protein
FUN_012016	contig_32	130653	131464	+		hypothetical protein
FUN_012020	contig_32	140256	141154	-		hypothetical protein
FUN_012021	contig_32	142251	142843	-		hypothetical protein
FUN_012022	contig_32	143376	144178	-	APT1	adenine phosphoribosyltransferase
FUN_012023	contig_32	145127	147265	-		hypothetical protein
FUN_012024	contig_32	148167	150817	+		hypothetical protein
FUN_012027	contig_32	155192	156515	-		hypothetical protein
FUN_012028	contig_32	157050	158626	-		hypothetical protein
FUN_012029	contig_32	159716	161538	+		hypothetical protein
FUN_012030	contig_32	163260	164329	+		hypothetical protein
FUN_012033	contig_32	171437	173281	+		hypothetical protein
FUN_012034	contig_32	174123	175677	-		hypothetical protein
FUN_012035	contig_32	178129	179562	+		hypothetical protein
FUN_012036	contig_32	181003	181794	+		hypothetical protein
FUN_012037	contig_32	182543	183469	+		hypothetical protein
FUN_012038	contig_32	183555	184460	-		hypothetical protein
FUN_012041	contig_32	191930	194704	-		hypothetical protein
FUN_012046	contig_32	210186	210652	+	RPL23A	60S ribosomal protein L23A
FUN_012048	contig_32	213545	215520	+		hypothetical protein
FUN_012049	contig_32	215898	217742	-		hypothetical protein
FUN_012050	contig_32	218105	219493	-	ENT3	Epsin-3, clathrin recruitment and traffic between the Golgi and endosome
FUN_012051	contig_32	220832	221624	+	CSR1_2	phosphatidylinositol transfer protein csr1
FUN_012052	contig_32	222186	223427	+		hypothetical protein
FUN_012054	contig_32	224347	225841	+	PUT1	proline dehydrogenase
FUN_012057	contig_32	234069	235864	-		hypothetical protein

Gene ID	Scaffold	Start	Stop	Strand	Name	Product
FUN_012059	contig_32	238989	241104	+	MAE1	NAD-dependent malic enzyme, mitochondrial
FUN_012061	contig_32	244169	251320	+	PRP8	pre-mRNA-splicing factor 8
FUN_012063	contig_32	255874	258747	+		hypothetical protein
FUN_012064	contig_32	260048	262401	-		hypothetical protein
FUN_012065	contig_32	264402	265820	-		hypothetical protein
FUN_012066	contig_32	267142	268333	+	RPC34	34-kDa subunit of RNA polymerase III (C)
FUN_012067	contig_32	268964	270119	+		hypothetical protein
FUN_012068	contig_32	270680	272729	-	HOS2	histone deacetylase
FUN_012069	contig_32	275766	278581	+	SPA2	component of the polarisome
FUN_012070	contig_32	280694	281635	+		hypothetical protein
FUN_012071	contig_32	283854	285679	-	PHO8	vacuolar alkaline phosphatase
FUN_012072	contig_32	287011	287945	+		hypothetical protein
FUN_012073	contig_32	288094	288878	-		hypothetical protein
FUN_012074	contig_32	289335	291076	+		hypothetical protein
FUN_012078	contig_32	298147	300145	-		hypothetical protein
FUN_012081	contig_32	304969	308320	-		hypothetical protein
FUN_012082	contig_32	309502	310491	+		hypothetical protein
FUN_012083	contig_32	311163	311912	+		hypothetical protein
FUN_012085	contig_32	314105	315952	+		hypothetical protein
FUN_012086	contig_32	316291	318558	+		hypothetical protein
FUN_012087	contig_32	324107	325173	+		hypothetical protein
FUN_012088	contig_32	325671	326217	-		hypothetical protein
FUN_012089	contig_32	327355	328965	+		hypothetical protein
FUN_012091	contig_32	332235	333788	-	PEX2	peroxisome assembly protein (Peroxin-2)
FUN_012092	contig_32	334183	337539	+		hypothetical protein
FUN_012093	contig_32	337958	340448	+		hypothetical protein
FUN_012095	contig_32	348349	349521	-		hypothetical protein
FUN_012096	contig_32	350549	352493	+		hypothetical protein
FUN_012097	contig_32	352671	353822	-		hypothetical protein
FUN_012098	contig_32	355985	356545	+		hypothetical protein
FUN_012099	contig_33	20702	21122	-		hypothetical protein
FUN_012101	contig_33	22939	24116	+		hypothetical protein
FUN_012102	contig_33	24493	28150	-		hypothetical protein
FUN_012103	contig_33	29313	31156	+		hypothetical protein
FUN_012104	contig_33	33092	33779	-	mxr1	Peptide methionine sulfoxide reductase
FUN_012105	contig_33	36410	37080	+		hypothetical protein
FUN_012106	contig_33	37406	38347	-		hypothetical protein
FUN_012107	contig_33	41709	43176	+	MNN4_2	mannosyltransferase
FUN_012108	contig_33	43401	45615	-		hypothetical protein
FUN_012111	contig_33	51127	52425	+		hypothetical protein
FUN_012112	contig_33	52735	55868	-	KIP3	tubulin-dependent ATPase kip3
FUN_012113	contig_33	58017	59031	+		hypothetical protein
FUN_012114	contig_33	59334	62301	-		hypothetical protein
FUN_012115	contig_33	65583	69251	-	HMG1	3-hydroxy-3-methylglutaryl-coenzyme A (HMG-CoA) reductase isozyme
FUN_012116	contig_33	70326	71099	-		hypothetical protein
FUN_012118	contig_33	73808	74529	-		hypothetical protein
FUN_012120	contig_33	79467	80141	+		hypothetical protein
FUN_012121	contig_33	82028	83458	+		hypothetical protein
FUN_012122	contig_33	84701	85556	+		hypothetical protein
FUN_012123	contig_33	85844	87504	-		hypothetical protein
FUN_012124	contig_33	89037	89603	-		hypothetical protein
FUN_012126	contig_33	92706	93866	+		hypothetical protein
FUN_012127	contig_33	94478	96942	+	dpp5_2	Dipeptidyl-peptidase 5
FUN_012128	contig_33	97975	99921	-	STI1	Hsp90 cochaperone
FUN_012129	contig_33	100265	100992	+		hypothetical protein
FUN_012130	contig_33	103024	104565	+	UGP1	UTP-glucose-1-phosphate uridylyltransferase
FUN_012132	contig_33	108306	113178	+	TCB2	Tricalbin-2
FUN_012134	contig_33	119357	121109	-		hypothetical protein

Gene ID	Scaffold	Start	Stop	Strand	Name	Product
FUN_012135	contig_33	122990	125072	-		hypothetical protein
FUN_012136	contig_33	126173	126754	+		hypothetical protein
FUN_012137	contig_33	126889	127893	-	COX4	Cytochrome c oxidase subunit 4
FUN_012138	contig_33	128113	128834	+	PNC1	NAD(+) salvage pathway protein
FUN_012139	contig_33	129406	131467	+	OSH6	Oxysterol-binding protein OBPa
FUN_012141	contig_33	140260	141026	+		hypothetical protein
FUN_012142	contig_33	144923	145432	+		hypothetical protein
FUN_012145	contig_33	165926	167481	+		hypothetical protein
FUN_012147	contig_33	170292	172208	+		hypothetical protein
FUN_012148	contig_33	172778	175247	-		hypothetical protein
FUN_012149	contig_33	177078	179036	-		hypothetical protein
FUN_012150	contig_33	179915	181736	-		hypothetical protein
FUN_012151	contig_33	182104	183049	+		hypothetical protein
FUN_012152	contig_33	183139	184072	-		hypothetical protein
FUN_012153	contig_33	184874	186389	+		hypothetical protein
FUN_012155	contig_33	188784	190693	-		hypothetical protein
FUN_012156	contig_33	191260	191859	-		hypothetical protein
FUN_012157	contig_33	193310	194471	-		hypothetical protein
FUN_012158	contig_33	195007	196021	-		hypothetical protein
FUN_012159	contig_33	196366	197778	+		hypothetical protein
FUN_012160	contig_33	198233	199441	+		hypothetical protein
FUN_012163	contig_33	203546	204370	-		hypothetical protein
FUN_012164	contig_33	206072	208131	-		hypothetical protein
FUN_012166	contig_33	211391	213313	-		hypothetical protein
FUN_012168	contig_33	217728	219314	+		hypothetical protein
FUN_012169	contig_33	219764	220067	-		hypothetical protein
FUN_012170	contig_33	220925	224149	+		hypothetical protein
FUN_012171	contig_33	228304	228698	+	RPS19A	Protein component of the small (40S) ribosomal subunit
FUN_012172	contig_33	229100	231304	-		hypothetical protein
FUN_012173	contig_33	233758	235887	+		hypothetical protein
FUN_012177	contig_33	244932	245999	-	SET6	Histone-lysine N-methyltransferase set-6
FUN_012178	contig_33	246762	247714	+		hypothetical protein
FUN_012179	contig_33	248268	249715	-		hypothetical protein
FUN_012180	contig_33	252237	254477	+	srk1	MAPK-activated protein kinase Srk1
FUN_012181	contig_33	255360	256153	-		hypothetical protein
FUN_012183	contig_33	257821	258438	-		hypothetical protein
FUN_012184	contig_33	259032	260240	+		hypothetical protein
FUN_012186	contig_33	264062	265926	-		hypothetical protein
FUN_012187	contig_33	269197	270612	+		hypothetical protein
FUN_012188	contig_33	273303	275075	+		hypothetical protein
FUN_012190	contig_33	278634	279437	+		hypothetical protein
FUN_012191	contig_33	279899	280467	-		hypothetical protein
FUN_012193	contig_33	290928	292127	-		hypothetical protein
FUN_012196	contig_33	308598	311258	+	SLX4	5'-flap endonuclease
FUN_012197	contig_33	314171	315781	-		hypothetical protein
FUN_012198	contig_33	316158	317201	+		hypothetical protein
FUN_012199	contig_33	317814	319417	+		hypothetical protein
FUN_012200	contig_33	320319	324494	+		hypothetical protein
FUN_012201	contig_33	324946	325965	-		hypothetical protein
FUN_012202	contig_33	326802	328700	-		hypothetical protein
FUN_012204	contig_33	331751	333706	+	PHR1_2	DNA photolyase phr1
FUN_012205	contig_33	334683	335954	-		hypothetical protein
FUN_012207	contig_33	341089	342055	-		hypothetical protein
FUN_012208	contig_34	20408	24535	-		hypothetical protein
FUN_012209	contig_34	27057	27449	-		hypothetical protein
FUN_012210	contig_34	42611	44522	+		hypothetical protein
FUN_012211	contig_34	54872	55579	+		hypothetical protein
FUN_012212	contig_34	69420	71701	-	MET7_6	Folylpolyglutamate synthetase
FUN_012213	contig_34	78381	79772	+		hypothetical protein

Gene ID	Scaffold	Start	Stop	Strand	Name	Product
FUN_012215	contig_34	90472	90750	+		hypothetical protein
FUN_012216	contig_34	95307	95657	+		hypothetical protein
FUN_012218	contig_34	99572	102830	+		hypothetical protein
FUN_012219	contig_34	104342	105790	-		hypothetical protein
FUN_012223	contig_34	134940	135577	-	RPD3_4	histone deacetylase
FUN_012224	contig_34	150123	150542	+		hypothetical protein
FUN_012227	contig_34	164772	165755	+		hypothetical protein
FUN_012228	contig_34	170333	172226	-		hypothetical protein
FUN_012229	contig_34	172757	173191	+		hypothetical protein
FUN_012230	contig_34	177431	178262	-		hypothetical protein
FUN_012234	contig_34	199955	201215	-		hypothetical protein
FUN_012235	contig_34	206518	207044	+		hypothetical protein
FUN_012236	contig_34	210941	211805	-		hypothetical protein
FUN_012237	contig_34	216958	217435	+		hypothetical protein
FUN_012239	contig_34	228246	228785	-		hypothetical protein
FUN_012240	contig_34	229522	230592	+		hypothetical protein
FUN_012243	contig_34	241670	242986	+	OXSR1	Serine/threonine-protein kinase OSR1
FUN_012244	contig_34	247921	250554	-		hypothetical protein
FUN_012245	contig_34	251662	254295	+		hypothetical protein
FUN_012247	contig_34	257080	258034	-		hypothetical protein
FUN_012249	contig_34	277873	279183	+		hypothetical protein
FUN_012251	contig_34	280800	281825	-	FANK1_2	Fibronectin type 3 and ankyrin repeat domains protein 1
FUN_012252	contig_34	283698	284589	+		hypothetical protein
FUN_012256	contig_34	304515	305687	-		hypothetical protein
FUN_012257	contig_34	306623	307926	+	AZF1_5	DNA-binding transcription factor
FUN_012258	contig_34	308820	309596	-		hypothetical protein
FUN_012259	contig_34	317270	317660	-	SMT3_4	SUMO protein smt3
FUN_012260	contig_34	321142	322543	+	MET7_7	Folylpolyglutamate synthetase
FUN_012261	contig_34	323728	324366	+	PCL1_8	PHO85 cyclin-1
FUN_012262	contig_34	328268	328486	-		hypothetical protein
FUN_012263	contig_35	60951	62486	+		hypothetical protein
FUN_012264	contig_35	64894	67147	-	ATM1	Iron-sulfur clusters transporter atm1, mitochondrial
FUN_012265	contig_35	67294	68533	+	AIM24	Altered inheritance of mitochondria protein 24, mitochondrial
FUN_012266	contig_35	69431	70864	-		hypothetical protein
FUN_012267	contig_35	71608	72732	+		hypothetical protein
FUN_012268	contig_35	73105	74493	-		hypothetical protein
FUN_012269	contig_35	77175	79728	-		hypothetical protein
FUN_012270	contig_35	80529	80956	+		hypothetical protein
FUN_012271	contig_35	81238	81894	-		hypothetical protein
FUN_012272	contig_35	82289	90195	-	UTP20	U3 snoRNP protein
FUN_012273	contig_35	90389	92255	+		hypothetical protein
FUN_012274	contig_35	95615	96346	+		hypothetical protein
FUN_012277	contig_35	101294	103546	-		hypothetical protein
FUN_012278	contig_35	103828	104478	+		hypothetical protein
FUN_012280	contig_35	112687	114603	-		hypothetical protein
FUN_012282	contig_35	116496	117978	+		hypothetical protein
FUN_012283	contig_35	118288	119353	-	CRC1	carnitine transporter
FUN_012284	contig_35	120600	121725	+		hypothetical protein
FUN_012285	contig_35	122758	123984	+	RMT2_2	Arginine N-methyltransferase 2
FUN_012286	contig_35	124100	125431	-		hypothetical protein
FUN_012287	contig_35	126330	127988	-		hypothetical protein
FUN_012290	contig_35	135002	136652	+		hypothetical protein
FUN_012291	contig_35	138175	138771	+	CTR3	Copper Transporter integral membrane protein that functions in high affinity copper transport
FUN_012292	contig_35	140448	142001	+		hypothetical protein
FUN_012293	contig_35	148176	149233	+		hypothetical protein
FUN_012294	contig_35	149704	151372	-		hypothetical protein
FUN_012295	contig_35	151676	153694	-		hypothetical protein

Gene ID	Scaffold	Start	Stop	Strand	Name	Product
FUN_012296	contig_35	154196	154861	-		hypothetical protein
FUN_012297	contig_35	155422	156457	-		hypothetical protein
FUN_012298	contig_35	157471	159233	+		hypothetical protein
FUN_012300	contig_35	162426	163425	+		hypothetical protein
FUN_012301	contig_35	164676	166551	+		hypothetical protein
FUN_012302	contig_35	167173	167961	+		hypothetical protein
FUN_012303	contig_35	169089	171082	-		hypothetical protein
FUN_012304	contig_35	172025	173974	-		hypothetical protein
FUN_012305	contig_35	174351	175546	-		hypothetical protein
FUN_012306	contig_35	176211	178222	-		hypothetical protein
FUN_012307	contig_35	181059	181934	+		hypothetical protein
FUN_012308	contig_35	183841	184796	+		hypothetical protein
FUN_012309	contig_35	185048	187470	-		hypothetical protein
FUN_012310	contig_35	190932	192859	-	AIP1	WD40 repeat-like protein
FUN_012311	contig_35	193043	195642	+	CHL1	ATP-dependent DNA helicase chl1
FUN_012312	contig_35	195898	197839	-		hypothetical protein
FUN_012313	contig_35	197979	198700	+		hypothetical protein
FUN_012314	contig_35	202954	205568	-		hypothetical protein
FUN_012315	contig_35	206528	207882	-		hypothetical protein
FUN_012316	contig_35	208186	209302	+	PEX7	peroxisomal targeting signal 2 receptor
FUN_012317	contig_35	210259	211481	+		hypothetical protein
FUN_012318	contig_35	213533	215196	-		hypothetical protein
FUN_012319	contig_35	218858	219820	+		hypothetical protein
FUN_012320	contig_35	221633	224280	-	MNR2	CorA metal ion transporter
FUN_012321	contig_35	225039	226764	-		hypothetical protein
FUN_012323	contig_35	229436	231655	-		hypothetical protein
FUN_012326	contig_35	233979	235109	-		hypothetical protein
FUN_012328	contig_35	237260	238180	+	NUO24	NADH:ubiquinone oxidoreductase 24
FUN_012329	contig_35	238480	239917	+		hypothetical protein
FUN_012330	contig_35	243649	245261	-		hypothetical protein
FUN_012331	contig_35	245488	246706	-		hypothetical protein
FUN_012332	contig_35	248861	250915	-		hypothetical protein
FUN_012333	contig_35	252583	254264	+		hypothetical protein
FUN_012334	contig_35	254441	255376	-		hypothetical protein
FUN_012335	contig_35	256244	257224	+	ncs1	Calcium-binding protein NCS-1
FUN_012336	contig_35	257788	259775	+	TMN2	Transmembrane 9 super member 2
FUN_012338	contig_35	268323	270402	-	PTR2_4	peptide transporter ptr2
FUN_012339	contig_35	271251	273367	-	AOX1	Alternative oxidase, mitochondrial precursor
FUN_012340	contig_35	275535	276717	-	AOX2	inducible alternative oxidase 2
FUN_012341	contig_35	278806	284071	+		hypothetical protein
FUN_012343	contig_35	286848	287885	-		hypothetical protein
FUN_012344	contig_35	288518	289285	+		hypothetical protein
FUN_012345	contig_35	293204	295184	+	lcc1_4	laccase, multicopper oxidase, benzenediol:oxygen oxidorectuctase
FUN_012346	contig_35	296357	297576	+		hypothetical protein
FUN_012349	contig_35	304058	305985	+		hypothetical protein
FUN_012350	contig_35	306287	307817	-		hypothetical protein
FUN_012351	contig_35	309390	310007	-	imp3	U3 small nucleolar ribonucleoprotein imp3
FUN_012352	contig_35	310240	312010	+	HIS7	Histidine biosynthesis bifunctional protein hisB
FUN_012353	contig_35	312132	313409	-	DIM1	Dimethyladenosine transferase
FUN_012354	contig_35	314028	315543	+	APN1	DNA-(apurinic or apyrimidinic site) lyase
FUN_012355	contig_35	315985	317798	-		hypothetical protein
FUN_012358	contig_36	11550	12084	-		hypothetical protein
FUN_012359	contig_36	12364	13262	-		hypothetical protein
FUN_012360	contig_36	14196	15382	-		hypothetical protein
FUN_012361	contig_36	15786	17947	-		hypothetical protein
FUN_012362	contig_36	18666	19444	-		hypothetical protein
FUN_012363	contig_36	19888	22049	-		hypothetical protein
FUN_012364	contig_36	22950	23572	-		hypothetical protein

Gene ID	Scaffold	Start	Stop	Strand	Name	Product
FUN_012365	contig_36	24054	24350	+		hypothetical protein
FUN_012366	contig_36	24694	25952	+		hypothetical protein
FUN_012367	contig_36	26078	26909	-		hypothetical protein
FUN_012368	contig_36	27509	28348	+		hypothetical protein
FUN_012369	contig_36	28518	29507	-		hypothetical protein
FUN_012370	contig_36	29770	31614	-		hypothetical protein
FUN_012371	contig_36	32131	33867	+		hypothetical protein
FUN_012372	contig_36	34154	35470	-		hypothetical protein
FUN_012374	contig_36	37736	38991	-		hypothetical protein
FUN_012376	contig_36	40444	41539	-		hypothetical protein
FUN_012379	contig_36	51481	51933	-	FPR2	Peptidyl-prolyl cis-trans isomerase fpr2
FUN_012383	contig_36	59818	61074	-		hypothetical protein
FUN_012384	contig_36	61394	62485	-		hypothetical protein
FUN_012385	contig_36	63058	64665	+		hypothetical protein
FUN_012387	contig_36	67619	69460	-		hypothetical protein
FUN_012388	contig_36	70650	72551	+		hypothetical protein
FUN_012390	contig_36	76469	79160	-		hypothetical protein
FUN_012391	contig_36	80057	82315	+		hypothetical protein
FUN_012392	contig_36	82565	83527	-		hypothetical protein
FUN_012394	contig_36	85321	86314	-		hypothetical protein
FUN_012395	contig_36	86925	87968	+		hypothetical protein
FUN_012397	contig_36	89221	90093	-	SOD4_2	Cell surface superoxide dismutase [Cu-Zn] 4
FUN_012398	contig_36	92137	95732	+		hypothetical protein
FUN_012400	contig_36	97380	98534	-		hypothetical protein
FUN_012401	contig_36	99185	100486	+		hypothetical protein
FUN_012402	contig_36	103273	106505	-	SPT16	FACT complex subunit spt16
FUN_012403	contig_36	106857	108065	+		hypothetical protein
FUN_012405	contig_36	113024	113929	+		hypothetical protein
FUN_012406	contig_36	117282	118460	+		hypothetical protein
FUN_012407	contig_36	119120	120894	+		hypothetical protein
FUN_012410	contig_36	129224	131024	-		hypothetical protein
FUN_012411	contig_36	131949	133049	+		hypothetical protein
FUN_012412	contig_36	133125	134298	-		hypothetical protein
FUN_012413	contig_36	134825	136687	+		hypothetical protein
FUN_012415	contig_36	139801	143582	+	MSH6	DNA mismatch repair protein msh6
FUN_012417	contig_36	145411	146867	-		hypothetical protein
FUN_012418	contig_36	147228	149342	+	NOP7	mRNA-binding ribosome synthesis protein nop7
FUN_012420	contig_36	150590	152299	+		hypothetical protein
FUN_012421	contig_36	153688	155595	-		hypothetical protein
FUN_012424	contig_36	162847	163662	+		hypothetical protein
FUN_012425	contig_36	166473	167322	-		hypothetical protein
FUN_012428	contig_36	174189	175354	+		hypothetical protein
FUN_012430	contig_36	181902	186144	-		hypothetical protein
FUN_012432	contig_36	189254	191266	-	MIC60	MICOS complex subunit mic60
FUN_012433	contig_36	192297	192974	+		hypothetical protein
FUN_012435	contig_36	198103	201940	-	agn1_1	Glucan endo-1,3-alpha-glucosidase agn1
FUN_012436	contig_36	203724	205963	-	SAC1	Phosphoinositide phosphatase sac1
FUN_012437	contig_36	206321	207176	+	YPT52	GTP-binding protein of the rab/ypt
FUN_012439	contig_36	211326	211649	-		hypothetical protein
FUN_012442	contig_36	239345	240376	-		hypothetical protein
FUN_012443	contig_36	240725	241379	+	RCF1	Respiratory supercomplex factor 1, mitochondrial
FUN_012444	contig_36	241655	243122	-		hypothetical protein
FUN_012445	contig_36	243661	246676	+		hypothetical protein
FUN_012446	contig_36	248248	249759	-	CGR1_2	rRNA-processing protein cgr1
FUN_012447	contig_36	251573	254118	+		hypothetical protein
FUN_012448	contig_36	254527	256835	+		hypothetical protein
FUN_012449	contig_36	258156	260723	+		hypothetical protein
FUN_012450	contig_36	264695	265853	+		hypothetical protein
FUN_012451	contig_36	269047	271174	-		hypothetical protein

Gene ID	Scaffold	Start	Stop	Strand	Name	Product
FUN_012452	contig_36	271453	272308	+		hypothetical protein
FUN_012453	contig_36	274248	276624	-		hypothetical protein
FUN_012454	contig_36	280642	281988	+		hypothetical protein
FUN_012455	contig_36	286887	288180	+		hypothetical protein
FUN_012457	contig_37	9783	11004	-		hypothetical protein
FUN_012458	contig_37	11996	15354	-		hypothetical protein
FUN_012459	contig_37	15573	18971	+	MSU1	3'-5' RNA exonuclease complex component
FUN_012460	contig_37	20288	23503	+		hypothetical protein
FUN_012462	contig_37	32312	33633	-		hypothetical protein
FUN_012463	contig_37	34036	37560	-		hypothetical protein
FUN_012464	contig_37	39297	40283	+		hypothetical protein
FUN_012466	contig_37	43475	47235	+		hypothetical protein
FUN_012467	contig_37	47609	48906	-		hypothetical protein
FUN_012468	contig_37	50316	57565	-	agn1_2	Glucan endo-1,3-alpha-glucosidase agn1
FUN_012470	contig_37	62747	63946	+		hypothetical protein
FUN_012471	contig_37	65249	66839	+	FCY21	Purine-cytosine permease fcy21
FUN_012472	contig_37	68487	70246	+		hypothetical protein
FUN_012474	contig_37	72990	73673	-		hypothetical protein
FUN_012477	contig_37	78953	84932	+		hypothetical protein
FUN_012480	contig_37	88179	89466	-		hypothetical protein
FUN_012481	contig_37	91988	93442	-	NBP2	HOG (high osmolarity glycerol) pathway protein
FUN_012482	contig_37	95228	96155	+	SSU72	RNA polymerase II subunit A C-terminal domain phosphatase
FUN_012483	contig_37	97958	99832	+		hypothetical protein
FUN_012484	contig_37	101094	103402	+		hypothetical protein
FUN_012485	contig_37	106762	109871	+		hypothetical protein
FUN_012486	contig_37	110958	112257	+		hypothetical protein
FUN_012487	contig_37	112876	114791	-		hypothetical protein
FUN_012489	contig_37	120143	121894	-	TIM50	mitochondrial inner membrane protein required for protein import
FUN_012490	contig_37	122340	125553	+		hypothetical protein
FUN_012491	contig_37	127023	128372	-		hypothetical protein
FUN_012492	contig_37	129043	129907	-		hypothetical protein
FUN_012495	contig_37	134419	136208	-	TPN1_3	Vitamin B6 transporter
FUN_012496	contig_37	137495	139183	+		hypothetical protein
FUN_012497	contig_37	147026	148336	-		hypothetical protein
FUN_012498	contig_37	153784	155171	+		hypothetical protein
FUN_012499	contig_37	159692	164041	+		hypothetical protein
FUN_012500	contig_37	164749	166542	+		hypothetical protein
FUN_012501	contig_37	168057	172773	+		hypothetical protein
FUN_012502	contig_37	172916	175738	-	MEP2	ammonium transporter
FUN_012506	contig_37	184034	186259	+		hypothetical protein
FUN_012508	contig_37	190180	191483	+	DOM34	Translation factor pelota
FUN_012510	contig_37	196767	197622	+		hypothetical protein
FUN_012511	contig_37	198181	200074	+		hypothetical protein
FUN_012514	contig_37	207022	209985	+		hypothetical protein
FUN_012515	contig_37	210777	211655	-	SKI6	Exosome non-catalytic core component
FUN_012516	contig_37	212013	215294	+		hypothetical protein
FUN_012517	contig_37	215435	216658	-		hypothetical protein
FUN_012518	contig_37	217238	219056	-		hypothetical protein
FUN_012519	contig_37	219522	221616	+		hypothetical protein
FUN_012520	contig_37	226485	229295	-		hypothetical protein
FUN_012521	contig_37	229994	231113	-		hypothetical protein
FUN_012522	contig_37	236010	237223	+		hypothetical protein
FUN_012523	contig_37	237348	238168	-		hypothetical protein
FUN_012527	contig_37	248357	250105	+		hypothetical protein
FUN_012528	contig_37	250782	253138	+		hypothetical protein
FUN_012529	contig_37	253284	254672	-		hypothetical protein
FUN_012530	contig_37	255663	257295	+		hypothetical protein

Gene ID	Scaffold	Start	Stop	Strand	Name	Product
FUN_012531	contig_37	259261	261697	-		hypothetical protein
FUN_012534	contig_37	276568	278160	-		hypothetical protein
FUN_012535	contig_37	279245	280974	+		hypothetical protein
FUN_012536	contig_37	283470	284992	+		hypothetical protein
FUN_012538	contig_38	9866	11315	-		hypothetical protein
FUN_012539	contig_38	13357	14821	-		hypothetical protein
FUN_012540	contig_38	18387	20071	-		hypothetical protein
FUN_012541	contig_38	20194	21758	-		hypothetical protein
FUN_012542	contig_38	23007	24038	+	rrp4	Exosome complex component rrp4
FUN_012543	contig_38	24552	26243	-		hypothetical protein
FUN_012544	contig_38	27589	28150	+		hypothetical protein
FUN_012545	contig_38	28582	29435	-		hypothetical protein
FUN_012547	contig_38	31670	32876	+		hypothetical protein
FUN_012548	contig_38	32978	35646	-	HMLALPHA2_3	homeodomain mating type protein alpha2
FUN_012549	contig_38	36994	39117	-		hypothetical protein
FUN_012550	contig_38	39433	41121	+	utp15	U3 small nucleolar RNA-associated protein 15
FUN_012551	contig_38	41746	46026	-	PRP24	Splicing factor
FUN_012552	contig_38	46672	47828	+	mrpl22	39S ribosomal protein L22, mitochondrial
FUN_012553	contig_38	49342	50446	+	emi5	Succinate dehydrogenase assembly factor 2 mitochondrial
FUN_012554	contig_38	51044	52035	+		hypothetical protein
FUN_012561	contig_38	69638	70468	-		hypothetical protein
FUN_012562	contig_38	71037	74961	+		hypothetical protein
FUN_012566	contig_38	83693	84976	+		hypothetical protein
FUN_012569	contig_38	90358	93577	-		hypothetical protein
FUN_012570	contig_38	94431	98134	-		hypothetical protein
FUN_012571	contig_38	98640	99728	-		hypothetical protein
FUN_012572	contig_38	100143	101318	+	SER2	Phosphoserine phosphatase
FUN_012573	contig_38	101549	104050	+		hypothetical protein
FUN_012574	contig_38	104277	105951	-		hypothetical protein
FUN_012575	contig_38	114939	115855	+		hypothetical protein
FUN_012576	contig_38	118334	120029	-		hypothetical protein
FUN_012578	contig_38	125507	129257	+		hypothetical protein
FUN_012582	contig_38	136461	138206	-		hypothetical protein
FUN_012583	contig_38	139055	140196	+		hypothetical protein
FUN_012584	contig_38	141098	141940	-		hypothetical protein
FUN_012585	contig_38	146678	147454	-		hypothetical protein
FUN_012587	contig_38	149486	152132	-		hypothetical protein
FUN_012588	contig_38	153533	153868	-		hypothetical protein
FUN_012589	contig_38	155672	156721	-		hypothetical protein
FUN_012590	contig_38	157329	158287	-		hypothetical protein
FUN_012592	contig_38	160196	160751	+		hypothetical protein
FUN_012593	contig_38	161610	162795	+	PGN1	glycoside hydrolase 28 protein
FUN_012596	contig_38	170173	170700	+		hypothetical protein
FUN_012597	contig_38	170932	172064	+		hypothetical protein
FUN_012599	contig_38	174920	175776	-		hypothetical protein
FUN_012601	contig_38	181108	182439	-		hypothetical protein
FUN_012602	contig_38	182681	184489	-		hypothetical protein
FUN_012603	contig_38	185870	187831	-	SED2	Tripeptidyl-peptidase sed2
FUN_012605	contig_38	194296	196682	+		hypothetical protein
FUN_012607	contig_38	200417	201862	-		hypothetical protein
FUN_012608	contig_38	204429	207026	-		hypothetical protein
FUN_012609	contig_38	208086	210295	-		hypothetical protein
FUN_012610	contig_38	210412	211472	+		hypothetical protein
FUN_012614	contig_38	224510	225572	+		hypothetical protein
FUN_012616	contig_38	227805	229896	+		hypothetical protein
FUN_012619	contig_38	233489	235107	-		hypothetical protein
FUN_012621	contig_38	237789	238997	-		hypothetical protein
FUN_012622	contig_38	239240	241214	+	KRS1	lysyl-tRNA synthetase

Gene ID	Scaffold	Start	Stop	Strand	Name	Product
FUN_012623	contig_38	244433	247080	+		hypothetical protein
FUN_012624	contig_38	249433	250754	-		hypothetical protein
FUN_012625	contig_38	251697	253800	-		hypothetical protein
FUN_012628	contig_38	259924	261754	-		hypothetical protein
FUN_012631	contig_39	12647	13867	-		hypothetical protein
FUN_012632	contig_39	17844	18696	-		hypothetical protein
FUN_012634	contig_39	21023	24238	-		hypothetical protein
FUN_012635	contig_39	27715	28352	-		hypothetical protein
FUN_012636	contig_39	32794	36989	-	VPS41	Vacuolar protein sorting-associated protein 41
FUN_012637	contig_39	37212	38633	+		hypothetical protein
FUN_012638	contig_39	39282	39861	+		hypothetical protein
FUN_012639	contig_39	41177	42092	-	RBD2	Putative rhomboid protease
FUN_012640	contig_39	42318	44336	+	GAA1	Glycosyl phosphatidyl inositol protein transamidase complex subunit
FUN_012641	contig_39	44677	45935	-	RPN9	26S proteasome regulatory subunit
FUN_012642	contig_39	46633	47572	+	EFM5_2	Protein-lysine N-methyltransferase efm5
FUN_012643	contig_39	48014	48732	+		hypothetical protein
FUN_012645	contig_39	53951	56290	-		hypothetical protein
FUN_012646	contig_39	60225	62402	+		hypothetical protein
FUN_012648	contig_39	68560	69423	-		hypothetical protein
FUN_012649	contig_39	72005	74751	+		hypothetical protein
FUN_012650	contig_39	75849	76933	-		hypothetical protein
FUN_012651	contig_39	77839	78612	-		hypothetical protein
FUN_012653	contig_39	81184	81918	-		hypothetical protein
FUN_012656	contig_39	86735	88222	-		hypothetical protein
FUN_012657	contig_39	89592	90199	+		hypothetical protein
FUN_012658	contig_39	90599	91215	-	SKI3_1	Superkiller protein 3
FUN_012659	contig_39	91558	94798	+	SKI3_2	Superkiller protein 3
FUN_012661	contig_39	99048	101625	+		hypothetical protein
FUN_012662	contig_39	102604	105444	-		hypothetical protein
FUN_012663	contig_39	106243	107232	-	OTU1	ubiquitin-specific protease otu1
FUN_012664	contig_39	107757	110335	+		hypothetical protein
FUN_012665	contig_39	110875	112456	+	LDB17	pre-rRNA processing
FUN_012666	contig_39	114107	116460	+		hypothetical protein
FUN_012667	contig_39	126157	127915	-		hypothetical protein
FUN_012668	contig_39	132661	133383	-		hypothetical protein
FUN_012669	contig_39	135027	136766	-		hypothetical protein
FUN_012670	contig_39	137164	138738	-		hypothetical protein
FUN_012671	contig_39	139120	141522	+	EGC1	endo-1,4-beta-glucanase
FUN_012672	contig_39	142200	146055	+	MSN5	karyopherin
FUN_012673	contig_39	146836	148034	+		hypothetical protein
FUN_012675	contig_39	156797	161688	+		hypothetical protein
FUN_012676	contig_39	161933	163054	-		hypothetical protein
FUN_012677	contig_39	163515	167189	-		hypothetical protein
FUN_012678	contig_39	167799	169178	-		hypothetical protein
FUN_012680	contig_39	172067	174639	+		hypothetical protein
FUN_012681	contig_39	174989	177603	-		hypothetical protein
FUN_012684	contig_39	181706	182568	-		hypothetical protein
FUN_012686	contig_39	183949	185910	-		hypothetical protein
FUN_012690	contig_39	191072	192096	-		hypothetical protein
FUN_012691	contig_39	193811	195186	-		hypothetical protein
FUN_012692	contig_39	195473	197233	+		hypothetical protein
FUN_012693	contig_39	197574	199379	-		hypothetical protein
FUN_012694	contig_39	200197	200805	+		hypothetical protein
FUN_012695	contig_39	204692	209879	-		hypothetical protein
FUN_012696	contig_39	211299	212153	-		hypothetical protein
FUN_012697	contig_39	214840	216250	-		hypothetical protein
FUN_012698	contig_39	216927	218993	-		hypothetical protein
FUN_012699	contig_39	219687	220634	+		hypothetical protein

Gene ID	Scaffold	Start	Stop	Strand	Name	Product
FUN_012700	contig_39	221213	222851	+	HXK2	Hexokinase isoenzyme 2
FUN_012701	contig_39	223325	225165	+		hypothetical protein
FUN_012702	contig_39	225216	226071	-		hypothetical protein
FUN_012703	contig_39	226270	226953	+		hypothetical protein
FUN_012705	contig_39	228030	229946	+		hypothetical protein
FUN_012706	contig_39	230813	231881	-		hypothetical protein
FUN_012707	contig_39	233364	234960	+		hypothetical protein
FUN_012708	contig_39	236311	237768	+		hypothetical protein
FUN_012711	contig_39	244251	244840	-		hypothetical protein
FUN_012712	contig_39	247257	251897	-		hypothetical protein
FUN_012716	contig_40	11387	12337	+		hypothetical protein
FUN_012717	contig_40	12361	14151	-		hypothetical protein
FUN_012718	contig_40	20683	22690	+		hypothetical protein
FUN_012719	contig_40	22837	24795	-		hypothetical protein
FUN_012720	contig_40	25126	26336	+		hypothetical protein
FUN_012721	contig_40	32565	33275	-	FYV4	telomere length regulation protein
FUN_012722	contig_40	33701	38257	+		hypothetical protein
FUN_012723	contig_40	38820	43138	-	PYC1	pyruvate carboxylase
FUN_012724	contig_40	44553	50164	-	CHC1	Clathrin heavy chain
FUN_012725	contig_40	51247	52225	-	HCR1	Translation initiation factor 3 subunit J component
FUN_012727	contig_40	59182	61817	+		hypothetical protein
FUN_012729	contig_40	67227	68226	+	PAM17	TIM23 complex component
FUN_012730	contig_40	69206	71816	-		hypothetical protein
FUN_012731	contig_40	74159	75454	-		hypothetical protein
FUN_012733	contig_40	79586	80363	+		hypothetical protein
FUN_012734	contig_40	80878	82784	+		hypothetical protein
FUN_012736	contig_40	86327	87516	+		hypothetical protein
FUN_012737	contig_40	87651	89472	-		hypothetical protein
FUN_012739	contig_40	98673	100166	-		hypothetical protein
FUN_012740	contig_40	100687	101193	-		hypothetical protein
FUN_012741	contig_40	101731	104569	-	PAK6	Serine/threonine-protein kinase PAK 6
FUN_012742	contig_40	106269	109037	-		hypothetical protein
FUN_012743	contig_40	110186	112810	-	EIS1	Eisosome assembly protein
FUN_012744	contig_40	113189	114064	+		hypothetical protein
FUN_012745	contig_40	114286	116702	+	MT2	Sphingolipid C9-methyltransferase 2
FUN_012746	contig_40	117268	118233	+	PMM1	Phosphomannomutase 1
FUN_012747	contig_40	125831	128343	-		hypothetical protein
FUN_012748	contig_40	129101	132978	-	EST2	Telomerase reverse transcriptase
FUN_012749	contig_40	134106	140530	+		hypothetical protein
FUN_012752	contig_40	151034	151466	+		hypothetical protein
FUN_012753	contig_40	152027	153544	-		hypothetical protein
FUN_012754	contig_40	153909	154257	+	LSM6	U4/U6-U5 snRNP complex subunit lsm6
FUN_012756	contig_40	160767	163177	+		hypothetical protein
FUN_012757	contig_40	163219	164460	-	oca3	Inositol phosphatase SIW14
FUN_012758	contig_40	164867	168111	+	APL5	AP-3 complex subunit delta
FUN_012759	contig_40	169465	170987	-	YTM1_1	ribosome bioproteinsis protein ytm1
FUN_012760	contig_40	171199	173794	-	YTM1_2	ribosome bioproteinsis protein ytm1
FUN_012761	contig_40	174096	177936	+		hypothetical protein
FUN_012762	contig_40	178898	181546	-		hypothetical protein
FUN_012763	contig_40	181901	182418	+	LSM5	RNA-binding protein lsm5
FUN_012764	contig_40	184539	185525	+		hypothetical protein
FUN_012765	contig_40	188206	190043	+		hypothetical protein
FUN_012767	contig_40	196278	198841	+		hypothetical protein
FUN_012768	contig_40	202256	203707	+		hypothetical protein
FUN_012770	contig_40	211833	213617	-		hypothetical protein
FUN_012771	contig_40	214543	216065	+		hypothetical protein
FUN_012772	contig_40	216192	217955	-		hypothetical protein
FUN_012773	contig_40	218611	223548	-	FRP1_2	ferric-chelate reductase Frp1
FUN_012775	contig_40	227877	229831	+		hypothetical protein

Gene ID	Scaffold	Start	Stop	Strand	Name	Product
FUN_012776	contig_40	232317	233647	-		hypothetical protein
FUN_012777	contig_40	236293	237536	+		hypothetical protein
FUN_012778	contig_41	14412	15656	-		hypothetical protein
FUN_012779	contig_41	20415	21578	-	MSW1	Tryptophan--tRNA ligase, mitochondrial
FUN_012780	contig_41	21815	22753	+	hob3	BAR adaptor protein Hob3
FUN_012781	contig_41	23890	24686	-	CDC34	Ubiquitin-conjugating enzyme subunit
FUN_012782	contig_41	25844	27814	-		hypothetical protein
FUN_012783	contig_41	28904	29307	+		hypothetical protein
FUN_012784	contig_41	29707	30207	-	RBX1	RING-box protein 1
FUN_012785	contig_41	31128	32774	+		hypothetical protein
FUN_012786	contig_41	34385	39731	-	YBT1	Transporter of the ATP-binding cassette (ABC)
FUN_012787	contig_41	42522	44198	+	PGS1	CDP-diacylglycerol--glycerol-3-phosphate 3-phosphatidyltransferase
FUN_012788	contig_41	44508	46204	-	SMP3	alpha 1,2 mannosyltransferase
FUN_012789	contig_41	47415	47975	+		hypothetical protein
FUN_012791	contig_41	49250	52302	-	LAS21	major facilitator super transporter protein
FUN_012792	contig_41	53444	56990	-	PDR6	member of the karyopherin-beta
FUN_012793	contig_41	57713	59699	+		hypothetical protein
FUN_012794	contig_41	60006	61198	-	SNZ1	Pyridoxal 5'-phosphate synthase subunit snz1
FUN_012795	contig_41	62242	67526	-	GCN2	eukaryotic translation initiation factor 2-alpha kinase
FUN_012796	contig_41	68085	69886	+		hypothetical protein
FUN_012800	contig_41	77213	80200	-		hypothetical protein
FUN_012801	contig_41	81021	82222	-	RAD14	DNA repair protein rad14
FUN_012802	contig_41	82776	83756	+		hypothetical protein
FUN_012803	contig_41	84099	85670	-		hypothetical protein
FUN_012804	contig_41	86026	87536	+		hypothetical protein
FUN_012805	contig_41	87927	89238	-		hypothetical protein
FUN_012806	contig_41	91970	93087	-		hypothetical protein
FUN_012807	contig_41	94155	95065	-		hypothetical protein
FUN_012808	contig_41	96812	98038	+	CWC2	Pre-mRNA-splicing factor
FUN_012809	contig_41	99520	101142	-		hypothetical protein
FUN_012810	contig_41	101932	106901	-	MYO2	Myosin type-2 heavy chain 1
FUN_012811	contig_41	107686	109427	-	SER33	D-3-phosphoglycerate dehydrogenase 2
FUN_012812	contig_41	110366	112387	-	ZWF1	Glucose-6-phosphate 1-dehydrogenase
FUN_012813	contig_41	115241	116931	-	SES1	Cytosolic seryl-tRNA synthetase
FUN_012814	contig_41	117823	119017	-	RHB1_2	GTP-binding protein
FUN_012815	contig_41	120232	123525	-		hypothetical protein
FUN_012816	contig_41	124302	124980	+	FUR1	Uracil phosphoribosyltransferase, synthesizes UMP from uracil
FUN_012817	contig_41	135346	135893	-		hypothetical protein
FUN_012818	contig_41	136393	138398	+	URC1	Putative GTP cyclohydrolase
FUN_012819	contig_41	143121	144035	+	RPS1	ribosomal 40S subunit protein S1B
FUN_012820	contig_41	146858	148884	-		hypothetical protein
FUN_012821	contig_41	149741	150887	+	HAM1	nucleoside triphosphate pyrophosphohydrolase ham1
FUN_012822	contig_41	151031	151765	-	FAP7	factor activating pos9
FUN_012823	contig_41	153121	154503	-	NAP1	histone chaperone
FUN_012824	contig_41	155389	157182	-		hypothetical protein
FUN_012827	contig_41	166045	167562	-		hypothetical protein
FUN_012828	contig_41	168893	169949	+		hypothetical protein
FUN_012829	contig_41	170208	171442	-		hypothetical protein
FUN_012832	contig_41	182656	184019	-	PHA2	prephenate dehydratase
FUN_012833	contig_41	184969	186320	-	ERF1	translation termination factor eRF1
FUN_012834	contig_41	187002	189078	+		hypothetical protein
FUN_012835	contig_41	189684	189993	+	TIM8	Mitochondrial import inner membrane translocase subunit tim8
FUN_012836	contig_41	190320	191456	-	RPL25	60S ribosomal protein L25
FUN_012837	contig_41	191658	192533	+	BMT2	25S rRNA (adenine2142-N1)-methyltransferase
FUN_012838	contig_41	192920	196259	-		hypothetical protein
FUN_012839	contig_41	196837	198570	+		hypothetical protein

Gene ID	Scaffold	Start	Stop	Strand	Name	Product
FUN_012840	contig_41	198678	201210	-		hypothetical protein
FUN_012842	contig_41	203578	205442	-		hypothetical protein
FUN_012844	contig_41	209122	210971	+		hypothetical protein
FUN_012846	contig_41	216949	219913	-		hypothetical protein
FUN_012848	contig_41	223832	226590	+	DCW1	hydrolase 76 protein
FUN_012849	contig_41	227322	228213	+		hypothetical protein
FUN_012850	contig_41	229502	232256	-		hypothetical protein
FUN_012851	contig_41	233446	235603	+		hypothetical protein
FUN_012852	contig_42	2900	3680	+		hypothetical protein
FUN_012853	contig_42	4684	6235	-	HSP31_2	plasma membrane heat shock protein
FUN_012854	contig_42	6989	9349	-		hypothetical protein
FUN_012855	contig_42	9836	12681	+		hypothetical protein
FUN_012856	contig_42	13169	14485	+		hypothetical protein
FUN_012857	contig_42	15228	16286	+		hypothetical protein
FUN_012858	contig_42	16426	17310	-		hypothetical protein
FUN_012859	contig_42	17562	19155	-		hypothetical protein
FUN_012860	contig_42	21334	22435	+		hypothetical protein
FUN_012861	contig_42	23850	25904	-		hypothetical protein
FUN_012862	contig_42	28339	31294	+		hypothetical protein
FUN_012867	contig_42	39055	42338	-		hypothetical protein
FUN_012868	contig_42	42767	48759	-	mug81	Putative steryl acetyl hydrolase mug81
FUN_012869	contig_42	49108	50439	+	YKT6	palmitoyltransferase
FUN_012872	contig_42	58934	62820	-		hypothetical protein
FUN_012876	contig_42	76554	78425	+		hypothetical protein
FUN_012877	contig_42	78467	79484	-		hypothetical protein
FUN_012878	contig_42	79918	80589	-	RPS14B	ribosomal 40S subunit protein S14B
FUN_012879	contig_42	80826	82661	+	TYR1	prephenate dehydrogenase (NADP(+))
FUN_012881	contig_42	85376	86724	+	RPL7_2	60S ribosomal protein L7
FUN_012882	contig_42	89324	90697	+		hypothetical protein
FUN_012883	contig_42	94573	95039	-		hypothetical protein
FUN_012884	contig_42	97261	99972	-		hypothetical protein
FUN_012885	contig_42	100841	101479	-	GOT1	Golgi Transport
FUN_012886	contig_42	102216	102797	-	IES6	chromatin-remodeling complex subunit ies6
FUN_012887	contig_42	103833	106239	+	ARP5	Actin-related protein 5
FUN_012888	contig_42	108157	114772	-		hypothetical protein
FUN_012889	contig_42	115340	116480	+		hypothetical protein
FUN_012890	contig_42	116972	118204	-	ARO4	3-deoxy-7-phosphoheptulonate synthase
FUN_012892	contig_42	122712	125240	-		hypothetical protein
FUN_012893	contig_42	127220	130471	-		hypothetical protein
FUN_012896	contig_42	136232	137991	-		hypothetical protein
FUN_012897	contig_42	139303	141163	-		hypothetical protein
FUN_012898	contig_42	141856	143679	-	ATG15	Putative lipase atg15
FUN_012899	contig_42	144198	146397	+		hypothetical protein
FUN_012900	contig_42	146751	149281	+		hypothetical protein
FUN_012903	contig_42	158408	160074	-		hypothetical protein
FUN_012904	contig_42	160634	161612	-	NAG1_2	Glucosamine-6-phosphate isomerase (Glucosamine-6-phosphate deaminase) (GNPDA) (GlcN6P deaminase)
FUN_012905	contig_42	163069	164680	+		hypothetical protein
FUN_012906	contig_42	166221	168002	+	NAG5	N-acetylglucosamine kinase 1
FUN_012907	contig_42	172222	173916	-		hypothetical protein
FUN_012908	contig_42	175103	178165	-		hypothetical protein
FUN_012909	contig_42	178737	180110	+	NAG2	N-acetyl-glucosamine-6-phosphate deacetylase
FUN_012910	contig_42	181123	182269	-		hypothetical protein
FUN_012911	contig_42	183244	184824	+	CYB2	Cytochrome b2, mitochondrial precursor
FUN_012912	contig_42	185145	186328	+	SFH5	Non-classical phosphatidylinositol transfer protein (PITP)
FUN_012913	contig_42	187519	188563	+		hypothetical protein
FUN_012915	contig_42	190618	192554	+		hypothetical protein
FUN_012916	contig_42	193077	193996	+	vip1	Protein vip1

Gene ID	Scaffold	Start	Stop	Strand	Name	Product
FUN_012918	contig_42	199638	201145	+		hypothetical protein
FUN_012919	contig_42	202981	203943	-		hypothetical protein
FUN_012920	contig_42	204606	206363	+		hypothetical protein
FUN_012921	contig_42	207528	212070	-		hypothetical protein
FUN_012922	contig_42	214859	217773	+		hypothetical protein
FUN_012923	contig_43	1723	3524	+		hypothetical protein
FUN_012924	contig_43	6128	7471	-		hypothetical protein
FUN_012925	contig_43	8749	9975	-		hypothetical protein
FUN_012926	contig_43	10711	13152	+		hypothetical protein
FUN_012927	contig_43	14325	17998	-		hypothetical protein
FUN_012928	contig_43	18325	21067	+		hypothetical protein
FUN_012930	contig_43	22605	27032	-		hypothetical protein
FUN_012931	contig_43	28083	29411	+		hypothetical protein
FUN_012932	contig_43	31063	32226	-		hypothetical protein
FUN_012933	contig_43	32645	34582	+		hypothetical protein
FUN_012934	contig_43	35149	37285	+		hypothetical protein
FUN_012935	contig_43	37378	41014	-		hypothetical protein
FUN_012936	contig_43	41701	43575	-		hypothetical protein
FUN_012937	contig_43	46903	48489	-	RVB1	RuvB ATP-dependent DNA helicase pontin
FUN_012938	contig_43	49731	52384	-	BDF1	transcription initiation at TATA-containing promoter protein
FUN_012939	contig_43	53556	55071	+	UTP5	Small subunit (SSU) processome component
FUN_012940	contig_43	55853	58191	+	PXA2	ATP-binding cassette long-chain fatty acid transporter pxa2
FUN_012941	contig_43	59083	65315	+	DCK1	Deoxycytidine kinase 1
FUN_012942	contig_43	65870	69706	+	RRP12	pre-rRNA processing protein
FUN_012944	contig_43	72233	73765	+	JEN1_2	Carboxylic acid transporter
FUN_012946	contig_43	76808	78785	+		hypothetical protein
FUN_012947	contig_43	79234	80302	-		hypothetical protein
FUN_012948	contig_43	80773	81753	+	PRE8	Proteasome subunit alpha type-2
FUN_012949	contig_43	81919	83098	-		hypothetical protein
FUN_012950	contig_43	83240	84545	-		hypothetical protein
FUN_012951	contig_43	86817	90605	-	PRM1	plasma membrane fusion protein prm1
FUN_012953	contig_43	97378	99652	-		hypothetical protein
FUN_012954	contig_43	101860	103034	+		hypothetical protein
FUN_012955	contig_43	104269	105803	+	RDS2	Transcription factor
FUN_012956	contig_43	106323	109941	+	SPO22	sporulation-specific protein 22
FUN_012957	contig_43	110136	112166	-		hypothetical protein
FUN_012959	contig_43	116779	117571	+		hypothetical protein
FUN_012960	contig_43	118346	120320	+		hypothetical protein
FUN_012963	contig_43	131123	133479	+		hypothetical protein
FUN_012964	contig_43	135578	136833	-		hypothetical protein
FUN_012965	contig_43	137291	138563	-		hypothetical protein
FUN_012966	contig_43	138946	141465	-	POP1	Ribonucleases P/MRP protein subunit pop1
FUN_012968	contig_43	143735	144922	-		hypothetical protein
FUN_012969	contig_43	145177	146309	+		hypothetical protein
FUN_012970	contig_43	146401	147717	-		hypothetical protein
FUN_012971	contig_43	148271	150050	+		hypothetical protein
FUN_012972	contig_43	150187	151800	-		hypothetical protein
FUN_012973	contig_43	153542	155782	+		hypothetical protein
FUN_012974	contig_43	155878	157622	-		hypothetical protein
FUN_012976	contig_43	158908	159875	-		hypothetical protein
FUN_012977	contig_43	160920	164635	+		hypothetical protein
FUN_012981	contig_44	1479	6403	+		hypothetical protein
FUN_012982	contig_44	7539	8985	+		hypothetical protein
FUN_012983	contig_44	9061	13221	-		hypothetical protein
FUN_012984	contig_44	14547	15887	-		hypothetical protein
FUN_012985	contig_44	16207	17760	+		hypothetical protein
FUN_012986	contig_44	18146	22130	+		hypothetical protein

Gene ID	Scaffold	Start	Stop	Strand	Name	Product
FUN_012987	contig_44	23048	28871	+		hypothetical protein
FUN_012991	contig_44	38836	47889	-		hypothetical protein
FUN_012992	contig_44	48946	51041	-		hypothetical protein
FUN_012993	contig_44	52564	54415	-		hypothetical protein
FUN_012997	contig_44	67281	68553	+		hypothetical protein
FUN_012998	contig_44	69621	71981	-		hypothetical protein
FUN_012999	contig_44	72333	75792	-		hypothetical protein
FUN_013001	contig_44	78113	78837	-		hypothetical protein
FUN_013002	contig_44	80257	83131	+		hypothetical protein
FUN_013003	contig_44	84126	85510	-	CTU1	cytosolic thiouridylase subunit Ctu1
FUN_013004	contig_44	86002	87032	+		hypothetical protein
FUN_013005	contig_44	87461	89198	+		hypothetical protein
FUN_013009	contig_44	102771	103973	-		hypothetical protein
FUN_013011	contig_44	109198	111721	+		hypothetical protein
FUN_013012	contig_44	113833	117521	-	DPH2	Diphthamide biosynthesis protein 2
FUN_013013	contig_44	117728	119892	-	PLB1	Lysophospholipase 1
FUN_013016	contig_44	130607	132259	-		hypothetical protein
FUN_013018	contig_44	137010	137887	-		hypothetical protein
FUN_013019	contig_44	137991	138805	-		hypothetical protein
FUN_013020	contig_44	139370	143038	-		hypothetical protein
FUN_013021	contig_44	143778	145522	+	REI1	pre-60S factor rei1
FUN_013023	contig_44	151059	153215	+		hypothetical protein
FUN_013024	contig_44	154951	157152	+		hypothetical protein
FUN_013025	contig_44	162581	165600	-	OSH3	Oxysterol-binding protein 3
FUN_013026	contig_44	167126	169571	-	QNS1	glutamine-dependent NAD(+) synthetase
FUN_013027	contig_44	170582	174582	+		hypothetical protein
FUN_013028	contig_44	175105	176562	-		hypothetical protein
FUN_013029	contig_44	177350	178462	+		hypothetical protein
FUN_013030	contig_44	178524	181268	-		hypothetical protein
FUN_013032	contig_45	8039	11447	-		hypothetical protein
FUN_013033	contig_45	11924	13882	+		hypothetical protein
FUN_013035	contig_45	17120	18312	+		hypothetical protein
FUN_013036	contig_45	18399	19081	-	COX11	Cytochrome c oxidase assembly protein cox11, mitochondrial
FUN_013037	contig_45	19616	21126	+		hypothetical protein
FUN_013040	contig_45	26062	28652	-		hypothetical protein
FUN_013041	contig_45	31050	33167	+	VMA2	Vacuolar ATP synthase subunit B
FUN_013042	contig_45	33289	34564	-		hypothetical protein
FUN_013043	contig_45	35491	37473	+		hypothetical protein
FUN_013044	contig_45	38185	40751	-		hypothetical protein
FUN_013045	contig_45	41766	42830	+		hypothetical protein
FUN_013047	contig_45	47670	49737	+		hypothetical protein
FUN_013049	contig_45	52802	54101	-		hypothetical protein
FUN_013050	contig_45	54989	56101	+		hypothetical protein
FUN_013055	contig_45	70562	71522	+		hypothetical protein
FUN_013057	contig_45	73271	75486	+	TRP2	anthranilate synthase component 1
FUN_013058	contig_45	76339	77368	+		hypothetical protein
FUN_013059	contig_45	90609	92991	+	TRP3_2	anthranilate synthase / indole-3-glycerol phosphate synthase
FUN_013060	contig_45	93124	96436	+	PHO91	low-affinity phosphate transporter
FUN_013062	contig_45	99680	101689	-		hypothetical protein
FUN_013068	contig_45	111978	113523	-		hypothetical protein
FUN_013069	contig_45	114186	119386	-	PDC1	Pyruvate decarboxylase 1
FUN_013070	contig_45	119831	120620	-		hypothetical protein
FUN_013071	contig_45	120871	121647	+		hypothetical protein
FUN_013072	contig_45	122108	124422	+		hypothetical protein
FUN_013079	contig_45	141351	142132	-		hypothetical protein
FUN_013080	contig_45	144733	146719	+		hypothetical protein
FUN_013081	contig_45	146966	147849	-		hypothetical protein

Gene ID	Scaffold	Start	Stop	Strand	Name	Product
FUN_013086	contig_45	158071	159947	-		hypothetical protein
FUN_013087	contig_45	160681	161592	+		hypothetical protein
FUN_013091	contig_46	12959	16935	-		hypothetical protein
FUN_013092	contig_46	17591	19267	-		hypothetical protein
FUN_013093	contig_46	25372	27100	+		hypothetical protein
FUN_013094	contig_46	28033	28772	+		hypothetical protein
FUN_013095	contig_46	29456	33034	+	HUL5	ubiquitin-protein ligase (E3)
FUN_013096	contig_46	33782	35549	+		hypothetical protein
FUN_013097	contig_46	35945	38127	-		hypothetical protein
FUN_013099	contig_46	41033	42415	+		hypothetical protein
FUN_013103	contig_46	55695	59075	-		hypothetical protein
FUN_013104	contig_46	62012	64770	+		hypothetical protein
FUN_013105	contig_46	65442	66704	+		hypothetical protein
FUN_013106	contig_46	68047	69373	+		hypothetical protein
FUN_013107	contig_46	69797	72589	+		hypothetical protein
FUN_013108	contig_46	73098	77132	-	SIT4_2	sporulation-induced protein
FUN_013109	contig_46	77951	82930	+	cta3	potassium/sodium eff
FUN_013110	contig_46	84359	85909	+		hypothetical protein
FUN_013115	contig_47	16363	17597	-		hypothetical protein
FUN_013116	contig_47	18258	19627	-		hypothetical protein
FUN_013117	contig_47	20412	22054	+		hypothetical protein
FUN_013118	contig_47	22487	24364	+		hypothetical protein
FUN_013119	contig_47	28853	29393	+		hypothetical protein
FUN_013120	contig_47	30153	31744	+		hypothetical protein
FUN_013121	contig_47	33788	36179	-		hypothetical protein
FUN_013123	contig_47	44718	46386	+		hypothetical protein
FUN_013124	contig_47	49293	50702	-		hypothetical protein
FUN_013125	contig_47	51424	53047	+		hypothetical protein
FUN_013127	contig_48	6435	9305	+		hypothetical protein
FUN_013129	contig_48	13150	16686	+	PEX3	peroxin
FUN_013132	contig_48	21871	22452	+		hypothetical protein
FUN_013133	contig_48	23502	25096	-		hypothetical protein
FUN_013134	contig_48	25591	26664	+		hypothetical protein
FUN_013135	contig_48	32264	36556	-		hypothetical protein
FUN_013137	contig_48	41833	43386	-		hypothetical protein
FUN_013138	contig_48	44883	46184	+		hypothetical protein
FUN_013156	contig_52	11123	12481	+		hypothetical protein
FUN_013157	contig_56	2194	3455	-		hypothetical protein
FUN_013158	contig_56	4833	7480	+		hypothetical protein
FUN_013160	contig_57	3532	4809	+		hypothetical protein
FUN_013161	contig_57	6737	8667	+		hypothetical protein
FUN_013162	contig_57	9559	13361	+	CDR1_5	Multidrug resistance protein
FUN_013163	contig_58	989	2091	-		hypothetical protein
FUN_013164	contig_58	2486	3609	-		hypothetical protein
FUN_013165	contig_66	1077	1417	-	MET7_8	Folylpolyglutamate synthetase

Table S2 KEGG-based functional annotation of predicted genes in E00680

Gene ID	Description	Name	EC	KEGG_ko	KEGG_Pathway
FUN_000002	Dehydrogenase	-	1.1.1.170	ko:K07748	ko00100, ko01100, ko01130, map00100, map01100, map01130
FUN_000005	Taurine catabolism dioxygenase TauD, TfdA family	-	-	-	-
FUN_000006	Glycosyl hydrolase family 61	-	-	-	-
FUN_000007	Voltage-dependent anion channel	-	-	-	-
FUN_000009	Protein of unknown function (DUF3830)	-	-	-	-
FUN_000010	GAL4-like Zn(II)2Cys6 (or C6 zinc) binuclear cluster DNA-binding domain	-	-	-	-
FUN_000011	Amino acid permease	-	-	ko:K16261	-
FUN_000013	Pal1 cell morphology protein	-	-	-	-
FUN_000014	Phosphodiesterase responsible for the U6 snRNA 3' end processing. Acts as an exoribonuclease (RNase) responsible for trimming the poly(U) tract of the last nucleotides in the pre-U6 snRNA molecule, leading to the formation of mature U6	USB1	-	-	-
FUN_000016	NPL4 family, putative zinc binding region	NPL4	-	ko:K14015	ko04141, map04141
FUN_000017	NPL4 family, putative zinc binding region	NPL4	-	ko:K14015	ko04141, map04141
FUN_000018	Domain of unknown function (DUF3844)	-	-	-	-
FUN_000021	Fungal specific transcription factor domain	DAL81	-	-	-
FUN_000023	ATPase domain of DNA mismatch repair MUTS family	msh1	-	-	-
FUN_000024	Belongs to the uroporphyrinogen decarboxylase family	HEM12	4.1.1.37	ko:K01599	ko00860, ko01100, ko01110, map00860, map01100, map01110
FUN_000025	MafB19-like deaminase	FCY1	3.5.4.1	ko:K01485	ko00240, ko00330, ko01100, map00240, map00330, map01100
FUN_000026	DnaJ central domain	YDJ1	-	ko:K09503	ko04141, map04141
FUN_000029	Belongs to the peptidase M10A family	-	-	-	-
FUN_000030	Fungal specific transcription factor domain	-	-	-	-
FUN_000031	Enoyl-(Acyl carrier protein) reductase	-	-	-	-
FUN_000035	Heterokaryon incompatibility protein (HET)	-	-	-	-
FUN_000039	Benzoate 4-monooxygenase cytochrome P450	-	-	-	-
FUN_000040	Cytochrome p-450	-	-	-	-
FUN_000041	Ankyrin repeats (many copies)	-	-	-	-
FUN_000042	RNA 3'-terminal phosphate cyclase (RTC), insert domain	-	-	ko:K11108	ko03008, map03008
FUN_000043	RTC4-like domain	-	-	-	-
FUN_000044	Phosphotransferase enzyme family	-	-	-	-
FUN_000047	ethyl tert-butyl ether degradation	-	-	-	-
FUN_000048	AMP-binding enzyme C-terminal domain	-	-	-	-
FUN_000050	Glycosyltransferase sugar-binding region containing DXD motif	-	2.4.1.232	ko:K05528	ko00513, ko01100, map00513, map01100
FUN_000051	Belongs to the cytochrome P450 family	-	-	-	-
FUN_000052	Endoplasmic reticulum vesicle transporter	ERV46	-	ko:K20367	-
FUN_000053	Sulfate permease family	-	-	ko:K14708	-
FUN_000054	Domain in Tre-2, BUB2p, and Cdc16p. Probable Rab-GAPs.	cdc16	-	ko:K02179	ko04111, map04111
FUN_000056	Belongs to the peptidase S8 family	KEX2	3.4.21.61	ko:K01341	-
FUN_000058	Heterokaryon incompatibility protein (HET)	-	-	-	-
FUN_000059	Enoyl-(Acyl carrier protein) reductase	-	-	-	-

FUN_000060	Indoleamine 2, 3-dioxygenase	-	-	-	-
FUN_000061	Alcohol dehydrogenase GroES-like domain	-	1.1.1.2	ko:K00002	ko00010, ko00040, ko00561, ko00930, ko01100, ko01110, ko01120, ko01130, ko01220, map00010, map00040, map00561, map00930, map01100, map01110, map01120, map01130, map01220
FUN_000062	Belongs to the mitochondrial carrier (TC 2.A.29) family	-	-	ko:K15100	-
FUN_000063	Protein of unknown function (DUF917)	-	-	-	-
FUN_000064	Permease for cytosine/purines, uracil, thiamine, allantoin	-	-	ko:K03457	-
FUN_000068	RNase H domain protein	-	3.1.26.4	ko:K03469	ko03030, map03030
FUN_000069	Heterokaryon incompatibility protein (HET)	-	-	-	-
FUN_000070	Glutathione S-transferase, N-terminal domain	-	-	-	-
FUN_000074	Hydantoinase	-	-	-	-
FUN_000075	Vacuolar effluxer which mediate the efflux of amino acids resulting from autophagic degradation. The release of autophagic amino acids allows the maintenance of protein synthesis and viability during nitrogen starvation	-	-	-	-
FUN_000076	positive regulation of ergosterol biosynthetic process by positive regulation of transcription from RNA polymerase II promoter	-	-	-	-
FUN_000077	FAD dependent oxidoreductase	-	-	-	-
FUN_000079	Transmembrane amino acid transporter protein	-	-	-	-
FUN_000080	to Saccharomyces cerevisiae GAL80 (YML051W)	GAL80	-	-	-
FUN_000081	taurine catabolism dioxygenase	-	-	-	-
FUN_000082	MmgE/PrpD family	-	-	-	-
FUN_000084	Protein of unknown function (DUF2985)	-	-	-	-
FUN_000085	Belongs to the cytochrome P450 family	-	-	-	-
FUN_000086	integral membrane protein	-	-	-	-
FUN_000087	KR domain	-	-	-	-
FUN_000091	Belongs to the protein kinase superfamily	CMK2	2.7.11.17	ko:K08794	ko04921, ko04925, map04921, map04925
FUN_000092	bromo domain	-	-	-	-
FUN_000094	Glycoside hydrolase family 17 protein	btgE	-	-	-
FUN_000095	NADPH-dependent methylglyoxal reductase	GRE2	1.1.1.283	ko:K17741	ko00620, ko00640, ko04011, map00620, map00640, map04011
FUN_000096	FAD binding domain	-	-	-	-
FUN_000097	Uncharacterized alpha/beta hydrolase domain (DUF2235)	-	-	-	-
FUN_000098	Amino acid permease	-	-	ko:K16261	-
FUN_000099	Amino acid permease	-	-	ko:K16261	-
FUN_000100	Six-hairpin glycosidase	-	-	-	-
FUN_000102	Belongs to the CDP-alcohol phosphatidyltransferase class-I family	-	2.7.8.1	ko:K00993	ko00440, ko00564, ko00565, ko01100, ko01110, map00440, map00564, map00565, map01100, map01110
FUN_000103	Bacterial regulatory helix-turn-helix proteins, AraC family	-	-	-	-
FUN_000104	DNA mismatch repair protein, C-terminal domain	-	-	-	-
FUN_000105	116 kDa U5 small nuclear ribonucleoprotein component N-terminus	SNU114	-	ko:K12852, ko:K12853	ko03040, map03040

FUN_000106	Belongs to the thiolase family	ERG10	2.3.1.9	ko:K00626	ko00071, ko00072, ko00280, ko00310, ko00362, ko00380, ko00620, ko00630, ko00640, ko00650, ko00720, ko00900, ko01100, ko01110, ko01120, ko01130, ko01200, ko01212, ko02020, map00071, map00072, map00280, map00310, map00362, map00380, map00620, map00630, map00640, map00650, map00720, map00900, map01100, map01110, map01120, map01130, map01200, map01212, map02020
FUN_000107	Glycoside hydrolase family 16 protein	-	-	-	-
FUN_000109	Zinc finger C-x8-C-x5-C-x3-H type (and similar)	YTH1	-	ko:K14404	ko03015, ko05164, map03015, map05164
FUN_000110	Belongs to the glycosyl hydrolase 18 family	-	3.2.1.14	ko:K01183	ko00520, ko01100, map00520, map01100
FUN_000111	Vps53-like, N-terminal	VPS53	-	ko:K20299	-
FUN_000113	Multicopper oxidase	-	-	-	-
FUN_000115	F-actin capping protein, beta subunit	CAP2	-	ko:K10365	ko04144, map04144
FUN_000116	Major facilitator superfamily transporter	-	-	-	-
FUN_000117	Zinc carboxypeptidase	-	-	-	-
FUN_000118	Beta-lactamase domain-containing protein	-	-	-	-
FUN_000119	Major Facilitator Superfamily	-	-	-	-
FUN_000120	Prolyl 4-hydroxylase alpha subunit homologues.	-	1.14.11.2	ko:K00472	ko00330, ko01100, map00330, map01100
FUN_000121	Pleckstrin homology domain.	-	-	ko:K12609	ko03018, map03018
FUN_000124	Arsenite-resistance protein 2	-	-	-	-
FUN_000125	PX-associated	-	-	-	-
FUN_000126	Nop14-like family	NOP14	-	ko:K14766	-
FUN_000128	Belongs to the DEAD box helicase family	TIF1	-	ko:K03257	ko03013, map03013
FUN_000129	A domain family that is part of the cupin metalloenzyme superfamily.	-	-	-	-
FUN_000130	PhoX homologous domain, present in p47phox and p40phox.	SNX4	-	ko:K17919	ko04144, map04144
FUN_000131	Involved in maintaining the homeostasis of cellular nucleotides by catalyzing the interconversion of nucleoside phosphates. Has GTP AMP phosphotransferase and ITP AMP phosphotransferase activities	ADK2	2.7.4.10, 2.7.4.3	ko:K00939, ko:K00944	ko00230, ko00730, ko01100, ko01110, ko01130, map00230, map00730, map01100, map01110, map01130
FUN_000132	LETM1-like protein	YLH47	-	ko:K17800	ko04139, map04139
FUN_000133	NUDIX domain	PCD1	-	-	-
FUN_000134	C-terminal domain of vertebrate Tap protein	MEX67	-	ko:K14284	ko03008, ko03013, ko03015, ko05164, ko05168, map03008, map03013, map03015, map05164, map05168
FUN_000135	Translation machinery-associated protein 22	TMA22	-	-	-
FUN_000136	Prefoldin subunit	GIM4	-	ko:K09549	-
FUN_000137	Histone-like transcription factor (CBF/NF-Y) and archaeal histone	HAP3	-	ko:K08065	ko04612, ko05152, ko05166, map04612, map05152, map05166
FUN_000138	Glycosyltransferase family 22 protein	ECM39	2.4.1.260	ko:K03847	ko00510, ko00513, ko01100, map00510, map00513, map01100
FUN_000139	Mediates the reversible addition of palmitate to target proteins, thereby regulating their membrane association and biological function	PFA4	2.3.1.225	ko:K18932	-
FUN_000140	Nineteen complex-related protein 2	-	-	-	-
FUN_000141	Ecdysteroid kinase	-	-	-	-
FUN_000142	CLIP1 zinc knuckle	-	-	-	-
FUN_000143	HELICc3	brr2	3.6.4.13	ko:K12854	ko03040, map03040

FUN_000144	Dynamitin	-	-	ko:K18599	-
FUN_000145	DUF4187	-	-	-	-
FUN_000146	Protein of unknown function (DUF3712)	-	-	-	-
FUN_000147	Origin recognition complex (ORC) subunit 3 N-terminus	ORC3	-	ko:K02605	ko04110, ko04111, ko04113, map04110, map04111, map04113
FUN_000148	Catalyzes the oxidative ring opening of 3- hydroxyanthranilate to 2-amino-3-carboxymuconate semialdehyde, which spontaneously cyclizes to quinolinate	BNA1	1.13.11.6	ko:K00452	ko00380, ko01100, map00380, map01100
FUN_000149	Ctr copper transporter family	CTR2	-	ko:K14686	ko01524, ko04978, map01524, map04978
FUN_000150	FAD dependent oxidoreductase	OSM2	-	ko:K18561	-
FUN_000151	Calcineurin-like phosphoesterase	-	-	-	-
FUN_000152	abscission	-	-	-	-
FUN_000153	Functions as actin-binding component of the Arp2 3 complex which is involved in regulation of actin polymerization and together with an activating nucleation-promoting factor (NPF) mediates the formation of branched actin networks	ARC35	-	ko:K05758	ko04144, ko04666, ko04810, ko05100, ko05130, ko05131, ko05132, map04144, map04666, map04810, map05100, map05130, map05131, map05132
FUN_000154	Dip2/Utp12 Family	DIP2	-	ko:K14556	ko03008, map03008
FUN_000155	Transcription factor S-II (TFIIS), central domain	tfs1	-	ko:K03145	-
FUN_000156	Histone acetyltransferase subunit NuA4	-	-	ko:K11344	-
FUN_000157	C2H2-type zinc finger	-	-	-	-
FUN_000158	PAS fold	-	-	-	-
FUN_000161	Belongs to the peptidase C19 family	creB	3.4.19.12	ko:K11842, ko:K11872	-
FUN_000162	SWIRM domain	-	1.5.3.14, 1.5.3.16	ko:K11450, ko:K13366, ko:K20182	ko00330, ko00410, ko01100, ko04138, ko04714, map00330, map00410, map01100, map04138, map04714
FUN_000163	endonuclease activity	lcl3	-	-	-
FUN_000164	Carbamoyl-phosphate synthetase large chain, oligomerisation domain	CPA2	6.3.5.5	ko:K01955	ko00240, ko00250, ko01100, map00240, map00250, map01100
FUN_000167	Autophagy protein Apg6	atg6	-	ko:K08334	ko04136, ko04137, ko04138, ko04140, ko04215, ko04371, ko05167, map04136, map04137, map04138, map04140, map04215, map04371, map05167
FUN_000169	Flavodoxin-like fold	PST2	1.6.5.2	ko:K03809	ko00130, ko01110, map00130, map01110
FUN_000170	CDF family cation efflux system protein	ZRC1	-	ko:K14688	ko04978, map04978
FUN_000171	Belongs to the peptidase A1 family	-	-	-	-
FUN_000172	Belongs to the sodium solute symporter (SSF) (TC 2.A.21) family	-	-	ko:K20989	-
FUN_000173	Beta-flanking protein	-	-	-	-
FUN_000175	Belongs to the complex I 49 kDa subunit family	NdufS2	1.6.5.3, 1.6.99.3	ko:K03935	ko00190, ko01100, ko04714, ko04723, ko04932, ko05010, ko05012, ko05016, map00190, map01100, map04714, map04723, map04932, map05010, map05012, map05016
FUN_000176	RNA polymerases D	RPC40	-	ko:K03027	ko00230, ko00240, ko01100, ko03020, ko04623, ko05169, map00230, map00240, map01100, map03020, map04623, map05169
FUN_000177	ATP synthase subunit gamma	ATP3	-	ko:K02136	ko00190, ko01100, ko04714, ko05010, ko05012, ko05016, map00190, map01100, map04714, map05010, map05012, map05016
FUN_000178	DNA Topoisomerase I (eukaryota)	TOP1	5.99.1.2	ko:K03163	-
FUN_000179	RmlD substrate binding domain	-	-	-	-

FUN_000180	Belongs to the TRAFAC class dynamin-like GTPase superfamily. Dynamin Fzo YdjA family	-	-	-	-
FUN_000181	Thioredoxin	-	5.3.4.1	ko:K09584	ko04141, map04141
FUN_000182	Vta1 like	-	-	ko:K12199	ko04144, map04144
FUN_000183	Belongs to the STXBP unc-18 SEC1 family	VPS33	-	ko:K20182	ko04138, map04138
FUN_000184	Belongs to the glycosyl hydrolase 31 family	-	-	-	-
FUN_000187	NADH:flavin oxidoreductase / NADH oxidase family	-	-	-	-
FUN_000188	family decarboxylase	-	-	-	-
FUN_000189	Fungal trichothecene efflux pump (TRI12)	-	-	-	-
FUN_000190	Fungal trichothecene efflux pump (TRI12)	-	-	-	-
FUN_000191	Small s protein	-	-	-	-
FUN_000192	Belongs to the heat shock protein 70 family	SSB1	-	ko:K03283	ko03040, ko04010, ko04141, ko04144, ko04213, ko04612, ko04915, ko05134, ko05145, ko05162, ko05164, ko05169, map03040, map04010, map04141, map04144, map04213, map04612, map04915, map05134, map05145, map05162, map05164, map05169
FUN_000194	Belongs to the universal ribosomal protein uS15 family	RPS13	-	ko:K02953	ko03010, map03010
FUN_000195	Putative TOS1-like glycosyl hydrolase (DUF2401)	TOS1	-	-	-
FUN_000196	Peroxin 13, N-terminal region	PEX13	-	ko:K13344	ko04146, map04146
FUN_000197	Belongs to the ubiquitin-conjugating enzyme family	-	2.3.2.25	ko:K10688	ko04120, map04120
FUN_000198	Splicing factor 3B subunit 1	prp10	-	ko:K12828	ko03040, map03040
FUN_000199	Belongs to the class-I aminoacyl-tRNA synthetase family	-	6.1.1.9	ko:K01873	ko00970, map00970
FUN_000200	Belongs to the protein kinase superfamily	CKA2	2.7.11.1	ko:K03097	ko03008, ko04064, ko04137, ko04139, ko04310, ko04520, ko04712, ko05162, ko05168, ko05169, map03008, map04064, map04137, map04139, map04310, map04520, map04712, map05162, map05168, map05169
FUN_000201	RNase P subunit p30	RPP1	3.1.26.5	ko:K03539	ko03008, ko03013, map03008, map03013
FUN_000203	Belongs to the mitochondrial carrier (TC 2.A.29) family	PIC2	-	ko:K15102	-
FUN_000204	Catalyzes the dehydration of methylthioribulose-1- phosphate (MTRu-1-P) into 2, 3-diketo-5-methylthiopentyl-1- phosphate (DK-MTP-1-P)	MDE1	4.2.1.109	ko:K08964	ko00270, ko01100, map00270, map01100
FUN_000205	Beta-lactamase	-	-	-	-
FUN_000206	hydrolase	-	-	-	-
FUN_000208	tRNA synthetases class II core domain (F)	FRS2	6.1.1.20	ko:K01889	ko00970, map00970
FUN_000209	RNA cytidine acetyltransferase with specificity toward both 18S rRNA and tRNAs. Catalyzes the formation of N(4)- acetylcytidine (ac4C) in 18S rRNA. Required for early nucleolar cleavages of precursor rRNA at sites A0, A1 and A2 during 18S rRNA synthesis. Catalyzes the formation of ac4C in serine and leucine tRNAs. Requires the tRNA-binding adapter protein TAN1 for full tRNA acetyltransferase activity but not for 18S rRNA [illegible]	NAT10	-	ko:K14521	ko03008, map03008
FUN_000210	IQ calmodulin-binding motif protein	-	-	-	-
FUN_000211	Protein of unknown function (DUF3659)	-	-	-	-
FUN_000212	Major Facilitator Superfamily	-	-	-	-
FUN_000213	Golgi CORVET complex core vacuolar protein 8	VPS8	-	ko:K20178	ko04138, map04138
FUN_000214	Protein of unknown function (DUF3752)	-	-	-	-

FUN_000216	Iron-sulfur cluster assembly protein	-	-	-	-
FUN_000217	Fungal specific transcription factor domain	-	-	-	-
FUN_000220	VRR_NUC	-	3.1.4.1	ko:K15363	ko03460, map03460
FUN_000223	Dihydrouridine synthase (Dus)	dus2	1.3.1.91	ko:K05543	-
FUN_000224	Belongs to the TRAFAC class myosin-kinesin ATPase superfamily. Myosin family	MYO1	-	ko:K10356	-
FUN_000225	Glycoside hydrolase family 3 protein	-	3.2.1.21	ko:K05349	ko00460, ko00500, ko00940, ko01100, ko01110, map00460, map00500, map00940, map01100, map01110
FUN_000226	Thioredoxin	-	-	-	-
FUN_000227	Histone deacetylase (HDAC) interacting	SIN3	-	ko:K11644	ko04139, ko04919, ko05016, ko05202, map04139, map04919, map05016, map05202
FUN_000228	Catalyzes the attachment of alanine to tRNA(Ala) in a two-step reaction alanine is first activated by ATP to form Ala- AMP and then transferred to the acceptor end of tRNA(Ala). Also edits incorrectly charged tRNA(Ala) via its editing domain	ALA1	6.1.1.7	ko:K01872	ko00970, map00970
FUN_000230	Chitin synthesis regulation, resistance to Congo red	-	-	-	-
FUN_000231	Bromo adjacent homology domain	SNT2	-	-	-
FUN_000232	helicase superfamily c-terminal domain	MPH1	3.6.4.12	ko:K14635	-
FUN_000233	Endoplasmic Reticulum Oxidoreductin 1 (ERO1)	ERO1	-	-	-
FUN_000234	Rhodanese Homology Domain	-	-	ko:K18064	-
FUN_000235	Belongs to the TRAFAC class myosin-kinesin ATPase superfamily. Myosin family	MYO1	-	ko:K10356	-
FUN_000236	Optic atrophy 3 protein (OPA3)	-	-	-	-
FUN_000237	POT family	PTR2	-	ko:K03305	-
FUN_000238	lysosomal cobalamin transporter. Required to export cobalamin from lysosomes allowing its conversion to cofactors (By similarity)	-	-	ko:K14617	ko04977, map04977
FUN_000239	CPSF A subunit region	CFT1	-	ko:K14401	ko03015, map03015
FUN_000240	Lea domain protein	-	3.1.1.45	ko:K01061	ko00361, ko00364, ko00623, ko01100, ko01110, ko01120, ko01130, map00361, map00364, map00623, map01100, map01110, map01120, map01130
FUN_000242	Fungal trichothecene efflux pump (TRI12)	-	-	-	-
FUN_000243	Cortical protein marker for cell polarity	-	-	-	-
FUN_000244	Essential subunit of the N-oligosaccharyl transferase (OST) complex which catalyzes the transfer of a high mannose oligosaccharide from a lipid-linked oligosaccharide donor to an asparagine residue within an Asn-X-Ser Thr consensus motif in nascent	OST2	-	ko:K12668	ko00510, ko00513, ko01100, ko04141, map00510, map00513, map01100, map04141
FUN_000245	Alanine-glyoxylate amino-transferase	ARO8	2.6.1.27, 2.6.1.39, 2.6.1.5, 2.6.1.57	ko:K00838	ko00130, ko00270, ko00300, ko00350, ko00360, ko00380, ko00400, ko00401, ko00950, ko00960, ko01100, ko01110, ko01130, ko01210, ko01230, map00130, map00270, map00300, map00350, map00360, map00380, map00400, map00401, map00950, map00960, map01100, map01110, map01130, map01210, map01230
FUN_000246	Sel1-like repeats.	CHS4	-	ko:K07126	-
FUN_000247	Glycosyltransferase family 2 protein	CHS3	2.4.1.16	ko:K00698	ko00520, map00520
FUN_000248	WD40 repeats	-	-	-	-
FUN_000249	TAP-like protein	-	-	-	-
FUN_000250	RNA recognition motif. (a.k.a. RRM, RBD, or RNP domain)	-	-	-	-
FUN_000252	Inorganic pyrophosphatase	IPP1	3.6.1.1	ko:K01507	ko00190, map00190
FUN_000253	Sec20	SEC20	-	ko:K08497	ko04130, map04130

FUN_000255	transcription initiation factor tfIID	taf2	-	ko:K03128	ko03022, map03022
FUN_000256	Tim17/Tim22/Tim23/Pmp24 family	-	-	ko:K13350	ko04146, map04146
FUN_000257	Belongs to the mitochondrial carrier (TC 2.A.29) family	ORT1	-	ko:K15114	-
FUN_000258	Involved in nucleolar integrity and required for processing of the pre-rRNA for the 60S ribosome subunit	CGR1	-	ko:K14822	-
FUN_000263	Lipase (class 3)	-	-	-	-
FUN_000264	zinc finger	-	-	-	-
FUN_000266	Metallo-beta-lactamase superfamily	-	-	-	-
FUN_000268	Sugar (and other) transporter	-	-	-	-
FUN_000270	Glycosyltransferase family 34 protein	MNN10	-	ko:K05531	ko00513, ko01100, map00513, map01100
FUN_000274	Bromodomain associated	-	-	ko:K14650	ko03022, ko05168, map03022, map05168
FUN_000275	TAFII55 protein conserved region	-	-	ko:K03132	ko03022, map03022
FUN_000276	Belongs to the universal ribosomal protein uS19 family	RPS15	-	ko:K02958	ko03010, map03010
FUN_000277	Belongs to the eukaryotic ribosomal protein P1 P2 family	RPP2B	-	ko:K02943	ko03010, map03010
FUN_000278	SPT2 chromatin protein	-	-	-	-
FUN_000279	Fungal specific transcription factor domain	-	-	-	-
FUN_000280	FAD binding domain	-	-	-	-
FUN_000282	Produces ATP from ADP in the presence of a proton gradient across the membrane	ATP1	-	ko:K02132	ko00190, ko01100, ko04714, ko05010, ko05012, ko05016, map00190, map01100, map04714, map05010, map05012, map05016
FUN_000283	Protein of unknown function (DUF962)	-	-	-	-
FUN_000284	SHS2 domain found in N terminus of Rpb7p/Rpc25p/MJ0397	RPB7	-	ko:K03015	ko00230, ko00240, ko01100, ko03020, ko05016, ko05169, map00230, map00240, map01100, map03020, map05016, map05169
FUN_000285	Type IV dipeptidyl-peptidase which removes N-terminal dipeptides sequentially from polypeptides having unsubstituted N- termini provided that the penultimate residue is proline	DAPB	-	ko:K01282	-
FUN_000286	Catalyzes the dehydration of the S-form of NAD(P)HX at the expense of ATP, which is converted to ADP. Together with NAD(P)HX epimerase, which catalyzes the epimerization of the S- and R-forms, the enzyme allows the repair of both epimers of NAD(P)HX, a damaged form of NAD(P)H that is a result of enzymatic or heat-dependent hydration	-	4.2.1.93	ko:K17757	-
FUN_000287	Exocyst complex component Sec10	SEC10	-	ko:K19984	-
FUN_000288	Putative undecaprenyl diphosphate synthase	-	2.5.1.87	ko:K19177	ko00900, ko01110, map00900, map01110
FUN_000289	Transcription initiation factor IIF, beta subunit	-	3.6.4.12	ko:K03139	ko03022, map03022
FUN_000290	motif in proteasome subunits, Int-6, Nip-1 and TRIP-15	-	-	ko:K12178	-
FUN_000291	Ribosomal prokaryotic L21 protein	-	-	-	-
FUN_000292	Ribosomal protein S8e	NSA2	-	ko:K14842	-
FUN_000295	Mitochondrial ATP synthase B chain precursor (ATP-synt_B)	ATP4	-	ko:K02127	ko00190, ko01100, ko04714, ko05010, ko05012, ko05016, map00190, map01100, map04714, map05010, map05012, map05016
FUN_000296	CBF/Mak21 family	NOC4	-	ko:K14771	-
FUN_000297	Sel1-like repeats.	-	-	-	-
FUN_000298	basic region leucin zipper	-	-	-	-
FUN_000299	pre-mRNA processing factor 4 (PRP4) like	-	-	ko:K12662	ko03040, map03040
FUN_000300	Protein phosphatase 2C	PTC7	3.1.3.16	ko:K17508	-

FUN_000301	Protein tyrosine kinase	-	2.7.12.1	ko:K08866	ko04110, ko04111, map04110, map04111
FUN_000302	mRNA cap-binding component of the eukaryotic translation initiation factor 3 (eIF-3) complex, which is involved in protein synthesis of a specialized repertoire of mRNAs and, together with other initiation factors, stimulates binding of mRNA and methionyl-tRNAi to the 40S ribosome. The eIF-3 complex specifically targets and initiates translation of a subset of mRNAs involved in cell proliferation. In the eIF-3 complex, eif3d specifically recognizes and binds the 7-methylguanosine cap of a subset of	-	-	ko:K03251	ko03013, map03013
FUN_000304	Type I phosphodiesterase / nucleotide pyrophosphatase	-	-	-	-
FUN_000305	Belongs to the peptidase A1 family	-	3.4.23.34, 3.4.23.5	ko:K01379, ko:K01382	ko04071, ko04140, ko04142, ko04210, ko04915, ko05152, map04071, map04140, map04142, map04210, map04915, map05152
FUN_000306	Belongs to the cytochrome P450 family	-	-	-	-
FUN_000307	Ribosomal protein P0 is the functional equivalent of E.coli protein L10	RPP0	-	ko:K02941	ko03010, map03010
FUN_000308	Required for the formation of N(7)-methylguanine at position 46 (m7G46) in tRNA. In the complex, it is required to stabilize and induce conformational changes of the catalytic subunit	TRM82	-	ko:K15443	-
FUN_000309	Belongs to the class-II pyridine nucleotide-disulfide oxidoreductase family	TRR1	1.6.5.5, 1.8.1.9	ko:K00344, ko:K00384	ko00450, map00450
FUN_000310	Vacuolar sorting 38 and autophagy-related subunit 14	-	-	-	-
FUN_000311	DegT/DnrJ/EryC1/StrS aminotransferase family	CYS3	4.4.1.1	ko:K01758	ko00260, ko00270, ko00450, ko01100, ko01130, ko01230, map00260, map00270, map00450, map01100, map01130, map01230
FUN_000312	Rad17 cell cycle checkpoint protein	rad17	-	ko:K06662	ko04111, ko04113, map04111, map04113
FUN_000314	Gamma-glutamyl cyclotransferase, AIG2-like	-	-	-	-
FUN_000315	WLM domain	-	-	-	-
FUN_000316	Fungal family of unknown function (DUF1776)	-	-	-	-
FUN_000317	Sel1-like repeats.	-	-	-	-
FUN_000319	Specifically methylates the N1 position of guanosine-37 in various cytoplasmic and mitochondrial tRNAs. Methylation is not dependent on the nature of the nucleoside 5' of the target nucleoside. This is the first step in the biosynthesis of wybutosine (yW), a modified base adjacent to the anticodon of tRNAs and required for accurate decoding	TRM5	2.1.1.228	ko:K15429	-
FUN_000320	Belongs to the TRAFAC class myosin-kinesin ATPase superfamily. Kinesin family	-	-	ko:K10392	-
FUN_000321	Acetyl-CoA acetyltransferase	-	-	-	-
FUN_000322	Telomere length regulation protein	TEL2	-	ko:K11137	ko03460, ko04150, map03460, map04150
FUN_000323	E2_bind	uba3	6.2.1.45	ko:K10686	ko04120, map04120
FUN_000326	endo-1, 3(4)-beta-glucanase	-	-	-	-
FUN_000328	D-isomer specific 2-hydroxyacid dehydrogenase, NAD binding domain	-	-	-	-
FUN_000329	Golgi membrane protein involved in vesicular trafficking	TVP23	-	-	-
FUN_000330	HpcH/HpaI aldolase/citrate lyase family	-	-	ko:K11390	-
FUN_000331	Arginine methyltransferase involved in the assembly or stability of mitochondrial NADH ubiquinone oxidoreductase complex (complex I)	-	-	-	-
FUN_000332	Pyridoxal-phosphate dependent enzyme	cys12	2.5.1.47	ko:K01738	ko00270, ko00920, ko01100, ko01110, ko01120, ko01130, ko01200, ko01230, map00270, map00920, map01100, map01110, map01120, map01130, map01200, map01230
FUN_000333	Prolyl oligopeptidase family	-	-	-	-

FUN_000334	Belongs to the protein kinase superfamily	ENV7	2.7.11.1	ko:K08856	-
FUN_000335	Protein of unknown function (DUF726)	-	-	ko:K14773	-
FUN_000336	Fcf1	-	-	ko:K14773	-
FUN_000337	Demethylates proteins that have been reversibly carboxymethylated	PPE1	3.1.1.89	ko:K13617	-
FUN_000338	Region of unknown function (DUF2417)	-	-	-	-
FUN_000339	Mitochondrial ribosomal protein L37	-	-	ko:K17435	-
FUN_000341	Nucleoporin Nup120/160	PWP1	-	ko:K14791	-
FUN_000342	Helicase associated domain (HA2) Add an annotation	-	3.6.4.13	ko:K12815	ko03040, map03040
FUN_000343	PWWP domain	-	-	-	-
FUN_000344	metallo-beta-lactamase	-	-	-	-
FUN_000346	Protein of unknown function (DUF3807)	-	-	-	-
FUN_000347	Cystathionine beta-synthase	-	-	-	-
FUN_000348	Stage II sporulation protein E (SpoIIE)	-	3.1.3.16	ko:K17508	-
FUN_000349	PWI domain	-	-	ko:K13171	ko03013, ko03015, map03013, map03015
FUN_000351	Fumarylacetoacetate (FAA) hydrolase family	-	3.7.1.5	ko:K01557	ko00350, ko01100, ko01120, map00350, map01100, map01120
FUN_000353	DUF1771	-	-	-	-
FUN_000354	SNARE associated Golgi protein	TVP38	-	-	-
FUN_000355	SNF2 family N-terminal domain	FUN30	3.6.4.12	ko:K14439	ko04550, map04550
FUN_000356	Required for the assembly of the V0 complex of the vacuolar ATPase (V-ATPase) in the endoplasmic reticulum	VMA21	-	-	-
FUN_000357	Uncharacterised protein (DUF2406)	-	-	-	-
FUN_000358	Aldehyde dehydrogenase family	ALD6	1.2.1.18, 1.2.1.27	ko:K00140	ko00280, ko00410, ko00562, ko00640, ko01100, ko01200, map00280, map00410, map00562, map00640, map01100, map01200
FUN_000359	Calreticulin family	-	-	ko:K08054	ko04141, ko04145, ko04612, ko04918, ko05166, map04141, map04145, map04612, map04918, map05166
FUN_000360	Fungal protein of unknown function (DUF1752)	REG1	-	-	-
FUN_000361	Fungal trichothecene efflux pump (TRI12)	-	-	-	-
FUN_000363	NADH-ubiquinone oxidoreductase 12 kda subunit	-	-	-	-
FUN_000364	BRO1-like domain	-	-	ko:K14650	ko03022, ko05168, map03022, map05168
FUN_000365	Amino acid permease	GPT1	-	-	-
FUN_000366	Saccharomyces cerevisiae YFR016C	-	-	-	-
FUN_000367	basic region leucin zipper	-	-	-	-
FUN_000368	Aldo/keto reductase family	-	-	-	-
FUN_000370	Alcohol dehydrogenase, zinc-containing	-	1.6.5.5	ko:K00344	-
FUN_000371	Maltose acetyltransferase	-	-	-	-
FUN_000372	ZIP Zinc transporter	-	-	ko:K14709	-
FUN_000373	Catalyzes the cleavage of glutathione into 5-oxo-L- proline and a Cys-Gly dipeptide. Acts specifically on glutathione, but not on other gamma-glutamyl peptides	-	-	ko:K07232	-

FUN_000374	FAD-dependent monooxygenase required for the C5-ring hydroxylation during ubiquinone biosynthesis. Catalyzes the hydroxylation of 3-polyprenyl-4-hydroxybenzoic acid to 3- polyprenyl-4, 5-dihydroxybenzoic acid. The electrons required for the hydroxylation reaction may be funneled indirectly from NADPH via a ferredoxin ferredoxin reductase system to COQ6	COQ6	-	ko:K06126	ko00130, ko01100, ko01110, map00130, map01100, map01110
FUN_000377	PX domain-containing protein	-	-	-	-
FUN_000378	Something about silencing, SAS, complex subunit 4	-	-	-	-
FUN_000379	Glutaredoxin	GRX3	-	-	-
FUN_000380	Brix	RPF2	-	ko:K14847	-
FUN_000381	Tetrahydrofolate dehydrogenase/cyclohydrolase, NAD(P)-binding domain	mtd1	1.5.1.15	ko:K00295	ko00670, ko01100, map00670, map01100
FUN_000382	Dos2-interacting transcription regulator of RNA-Pol-II	-	-	ko:K15075	-
FUN_000383	Belongs to the major facilitator superfamily. Sugar transporter (TC 2.A.1.1) family	-	-	-	-
FUN_000385	KR domain	-	-	-	-
FUN_000387	8-oxoguanine DNA glycosylase, N-terminal domain	OGG1	4.2.99.18	ko:K03660	ko03410, map03410
FUN_000388	U2 snRNP auxiliary factor large subunit	-	-	ko:K12837	ko03040, map03040
FUN_000389	Survival protein SurE	-	3.1.3.5	ko:K03787	ko00230, ko00240, ko00760, ko01100, ko01110, map00230, map00240, map00760, map01100, map01110
FUN_000390	cfem domain-containing protein	-	-	-	-
FUN_000391	Carbohydrate esterase family 1 protein	-	-	-	-
FUN_000393	Cytochrome p-450	-	-	-	-
FUN_000395	Beta-lactamase	-	-	-	-
FUN_000396	AAA domain	-	-	-	-
FUN_000399	Common central domain of tyrosinase	-	1.14.18.1	ko:K00505	ko00350, ko00950, ko00965, ko01100, ko01110, ko04916, map00350, map00950, map00965, map01100, map01110, map04916
FUN_000402	S-adenosyl-L-methionine-dependent methyltransferase	-	-	-	-
FUN_000403	Sugar (and other) transporter	-	-	-	-
FUN_000405	Enoyl-(Acyl carrier protein) reductase	-	-	-	-
FUN_000406	Belongs to the iron ascorbate-dependent oxidoreductase family	-	-	-	-
FUN_000411	cytochrome p450	-	-	-	-
FUN_000412	Endonuclease Exonuclease phosphatase family protein	-	-	ko:K19619	-
FUN_000413	NADH:flavin oxidoreductase / NADH oxidase family	-	1.6.99.1	ko:K00354	-
FUN_000414	Aldehyde oxidase and xanthine dehydrogenase, a/b hammerhead domain	-	1.17.1.4, 1.17.3.2	ko:K00106	ko00230, ko00232, ko00983, ko01100, ko01110, ko01120, ko04146, map00230, map00232, map00983, map01100, map01110, map01120, map04146
FUN_000415	Fungal specific transcription factor domain	-	-	-	-
FUN_000416	Nucleotide-sugar transporter	-	-	-	-
FUN_000417	Major facilitator superfamily transporter	-	-	-	-
FUN_000418	Amidase	-	3.5.1.4	ko:K01426	ko00330, ko00360, ko00380, ko00627, ko00643, ko01120, map00330, map00360, map00380, map00627, map00643, map01120
FUN_000419	Phenol hydroxylase, C-terminal dimerisation domain	-	-	-	-
FUN_000420	Polysaccharide deacetylase	-	-	-	-
FUN_000421	FAD binding domain	-	1.14.13.1	ko:K00480	ko00621, ko00624, ko00626, ko01100, ko01120, ko01220, map00621, map00624, map00626, map01100, map01120, map01220

FUN_000422	Spherulation-specific family 4	-	-	-	-
FUN_000423	Belongs to the major facilitator superfamily. Sugar transporter (TC 2.A.1.1) family	-	-	-	-
FUN_000424	Belongs to the iron ascorbate-dependent oxidoreductase family	-	-	-	-
FUN_000425	sterol 14-demethylase activity	-	1.14.13.96, 1.14.14.23,	ko:K00489, ko:K07430, ko:K07431	ko00120, ko00140, ko01100, ko03320, ko04976, ko04979, map00120, map00140, map01100, map03320, map04976, map04979
FUN_000427	Methyltransferase domain	-	-	ko:K15340	-
FUN_000428	Belongs to the ribulose-phosphate 3-epimerase family	RPE1	5.1.3.1	ko:K01783	ko00030, ko00040, ko00710, ko01100, ko01110, ko01120, ko01130, ko01200, ko01230, map00030, map00040, map00710, map01100, map01110, map01120, map01130, map01200, map01230
FUN_000430	alpha-amylase	-	3.2.1.1	ko:K01176	ko00500, ko01100, ko04973, map00500, map01100, map04973
FUN_000435	Methyltransferase domain	-	2.1.1.144	ko:K00598	-
FUN_000436	GTPase-activator protein for Rho-like GTPases	rga1	-	-	-
FUN_000437	DNA-directed RNA-polymerase II subunit	RPB4	-	ko:K03012	ko00230, ko00240, ko01100, ko03020, ko05016, ko05169, map00230, map00240, map01100, map03020, map05016, map05169
FUN_000438	Common central domain of tyrosinase	-	1.14.18.1	ko:K00505	ko00350, ko00950, ko00965, ko01100, ko01110, ko04916, map00350, map00950, map00965, map01100, map01110, map04916
FUN_000439	Lanthionine synthetase C-like protein	-	-	-	-
FUN_000441	oxidoreductase activity	-	-	-	-
FUN_000442	Cupin	-	4.1.1.2	ko:K01569	ko00630, ko01100, map00630, map01100
FUN_000443	selenium binding protein	-	-	ko:K17285	-
FUN_000444	Multidrug resistance protein	-	3.6.3.44	ko:K05658	ko02010, ko04976, ko05206, ko05226, map02010, map04976, map05206, map05226
FUN_000445	DNA repair metallo-beta-lactamase	pso2	-	ko:K03251, ko:K15340	ko03013, map03013
FUN_000446	NADH:flavin oxidoreductase / NADH oxidase family	-	-	-	-
FUN_000447	N-terminal domain of oxidoreductase	-	-	ko:K07119	-
FUN_000449	NADH-ubiquinone oxidoreductase 178 kDa subunit	-	-	-	-
FUN_000450	Phosphoinositide phospholipase C	-	-	-	-
FUN_000451	RNA polymerase III subunit Rpc25	rpc25	-	ko:K03022	ko00230, ko00240, ko01100, ko03020, ko04623, ko05169, map00230, map00240, map01100, map03020, map04623, map05169
FUN_000452	Nuclear fragile X mental retardation-interacting protein 1 (NUFIP1)	-	-	-	-
FUN_000454	Isochorismatase family	-	-	-	-
FUN_000456	Belongs to the major facilitator superfamily. Sugar transporter (TC 2.A.1.1) family	SNF3	-	ko:K08139	ko04113, map04113
FUN_000457	Phosphomethylpyrimidine kinase	BUD16	2.7.1.35	ko:K00868	ko00750, ko01100, map00750, map01100
FUN_000458	This protein is an auxiliary protein of DNA polymerase delta and is involved in the control of eukaryotic DNA replication by increasing the polymerase's processibility during elongation of the leading strand	POL30	-	ko:K04802	ko03030, ko03410, ko03420, ko03430, ko04110, ko04530, ko05161, ko05166, map03030, map03410, map03420, map03430, map04110, map04530, map05161, map05166
FUN_000459	Mysoin-binding motif of peroxisomes	-	-	-	-
FUN_000460	Belongs to the type-B carboxylesterase lipase family	-	-	-	-
FUN_000462	Domain of unknown function (DUF3453)	-	-	ko:K06100	ko03015, ko04530, map03015, map04530

FUN_000463	Coiled-coil domain-containing protein 56	-	-	ko:K18176	-
FUN_000465	Belongs to the TPP enzyme family	-	-	ko:K12261	ko04146, map04146
FUN_000468	Belongs to the GST superfamily	-	-	ko:K03233	ko05134, map05134
FUN_000469	Bacterial protein of unknown function (DUF924)	-	-	-	-
FUN_000470	Adenosine/AMP deaminase	AMD1	3.5.4.6	ko:K01490	ko00230, ko01100, ko01110, ko01130, map00230, map01100, map01110, map01130
FUN_000471	RNA recognition motif. (a.k.a. RRM, RBD, or RNP domain)	-	-	-	-
FUN_000472	Tetratricopeptide repeat	PEX5	-	ko:K12261, ko:K13342	ko04146, map04146
FUN_000473	Belongs to the RNase T2 family	-	3.1.27.1	ko:K01166	-
FUN_000474	Beta-lactamase superfamily domain	-	-	-	-
FUN_000476	Glycosyltransferase family 39 protein	PMT4	2.4.1.109	ko:K00728	ko00514, ko00515, ko01100, map00514, map00515, map01100
FUN_000477	Glycosyltransferase family 39 protein	PMT4	2.4.1.109	ko:K00728	ko00514, ko00515, ko01100, map00514, map00515, map01100
FUN_000478	Belongs to the phosphatase 2A regulatory subunit B family	CDC55	-	ko:K04354	ko03015, ko04071, ko04111, ko04151, ko04152, ko04261, ko04390, ko04391, ko04530, ko04728, ko05142, ko05160, ko05165, map03015, map04071, map04111, map04151, map04152, map04261, map04390, map04391, map04530, map04728, map05142, map05160, map05165
FUN_000479	Belongs to the universal ribosomal protein uL3 family	RPL3	-	ko:K02925	ko03010, map03010
FUN_000480	Flavoprotein (FP) subunit of succinate dehydrogenase (SDH) that is involved in complex II of the mitochondrial electron transport chain and is responsible for transferring electrons from succinate to ubiquinone (coenzyme Q)	SDH1	1.3.5.1	ko:K00234	ko00020, ko00190, ko01100, ko01110, ko01120, ko01130, ko01200, ko04714, ko04932, ko05010, ko05012, ko05016, map00020, map00190, map01100, map01110, map01120, map01130, map01200, map04714, map04932, map05010, map05012, map05016
FUN_000481	Glycosyltransferase family 32 protein	-	2.4.1.232	ko:K05528	ko00513, ko01100, map00513, map01100
FUN_000482	Protein tyrosine kinase	-	-	-	-
FUN_000484	pentatricopeptide repeat protein	-	-	-	-
FUN_000485	Cytochrome domain of cellobiose dehydrogenase	-	-	-	-
FUN_000487	Domain of unknown function (DUF1708)	-	-	-	-
FUN_000488	MADS	RLM1	-	ko:K09265, ko:K19807	ko04011, map04011
FUN_000489	EF-hand domain pair	-	-	-	-
FUN_000490	SUR7/PalI family	-	-	-	-
FUN_000493	ELMO/CED-12 family	-	-	ko:K12366	ko04062, ko05100, ko05131, map04062, map05100, map05131
FUN_000495	assists the folding of proteins upon ATP hydrolysis	CCT4	-	ko:K09496	-
FUN_000496	Belongs to the AAA ATPase family	RPT1	-	ko:K03061	ko03050, ko05169, map03050, map05169
FUN_000497	RNA polymerase I specific transcription initiation factor RRN3	RRN3	-	ko:K15216	-
FUN_000498	PPIases accelerate the folding of proteins. It catalyzes the cis-trans isomerization of proline imidic peptide bonds in oligopeptides	-	5.2.1.8	ko:K09567	ko03040, map03040
FUN_000499	RING-like zinc finger	-	-	-	-
FUN_000501	aaa family atpase	-	-	-	-
FUN_000503	Belongs to the TRAFAC class dynamin-like GTPase superfamily. Dynamin Fzo YdjA family	-	-	-	-

FUN_000504	Dihydrodipicolinate synthetase family	-	4.1.3.16	ko:K18123	ko00330, ko00630, ko01100, map00330, map00630, map01100
FUN_000506	F-box domain containing protein	-	-	-	-
FUN_000507	Cofilin/tropomyosin-type actin-binding protein	ABP1	-	ko:K20520	-
FUN_000508	Serine hydrolase (FSH1)	-	-	-	-
FUN_000509	Kelch	-	-	-	-
FUN_000513	Peptidase dimerisation domain	-	-	-	-
FUN_000514	Forkhead associated domain	RAD53	2.7.11.1	ko:K02831, ko:K06641	ko04110, ko04111, ko04113, ko04115, ko04218, ko05166, map04110, map04111, map04113, map04115, map04218, map05166
FUN_000515	Glucosidase II beta subunit-like protein	YOS9	-	ko:K10088	ko04141, map04141
FUN_000516	Enoyl-(Acyl carrier protein) reductase	-	3.5.4.5	ko:K01489	ko00240, ko00983, ko01100, map00240, map00983, map01100
FUN_000517	This enzyme scavenges exogenous and endogenous cytidine and 2'-deoxycytidine for UMP synthesis	-	3.5.4.5	ko:K01489	ko00240, ko00983, ko01100, map00240, map00983, map01100
FUN_000518	HSCB C-terminal oligomerisation domain	JAC1	-	ko:K04082	-
FUN_000522	EXOIII	-	-	ko:K14570	ko03008, map03008
FUN_000523	Glycosyltransferase family 66 protein	STT3	2.4.99.18	ko:K07151	ko00510, ko00513, ko01100, ko04141, map00510, map00513, map01100, map04141
FUN_000524	zinc finger	-	-	-	-
FUN_000525	The EMC seems to be required for efficient folding of proteins in the endoplasmic reticulum (ER)	-	-	-	-
FUN_000526	Microsomal signal peptidase 25 kDa subunit (SPC25)	-	-	-	-
FUN_000528	zinc finger	-	-	-	-
FUN_000529	zinc finger	-	-	-	-
FUN_000533	Polysaccharide biosynthesis protein	GAL102	4.2.1.46	ko:K01710	ko00521, ko00523, ko00525, ko01055, ko01130, map00521, map00523, map00525, map01055, map01130
FUN_000534	Helical region found in SNAREs	SEC9	-	ko:K19950	-
FUN_000535	Glycoside hydrolase family 38 protein	AMS1	3.2.1.24	ko:K01191	ko00511, map00511
FUN_000537	Extension to Ser/Thr-type protein kinases	RIM15	2.7.11.1	ko:K12767	ko04113, ko04138, ko04213, map04113, map04138, map04213
FUN_000538	Protein of unknown function (DUF2945)	-	-	-	-
FUN_000542	Belongs to the TRAFAC class dynamin-like GTPase superfamily. Dynamin Fzo YdjA family	DNM1	3.6.5.5	ko:K17065	ko04139, ko04214, ko04217, ko04621, ko04668, map04139, map04214, map04217, map04621, map04668
FUN_000543	Phosphatidylethanolamine-binding protein	-	-	-	-
FUN_000544	ATP synthase E chain	TIM11	-	ko:K01549	ko00190, map00190
FUN_000545	WD40 repeats	-	-	ko:K10415	ko04145, ko04962, ko05132, map04145, map04962, map05132
FUN_000546	Ser-Thr-rich glycosyl-phosphatidyl-inositol-anchored membrane family	-	-	-	-
FUN_000548	SET (Su(var)3-9, Enhancer-of-zeste, Trithorax) domain	-	-	ko:K11426	-
FUN_000549	Right handed beta helix region	-	-	-	-
FUN_000550	Functions as a sorting receptor in the Golgi compartment required for the intracellular sorting and delivery of soluble vacuolar proteins, like carboxypeptidase Y (CPY) and proteinase A	VPS10	-	-	-
FUN_000551	Belongs to the peptidase S8 family	-	3.4.21.48	ko:K01336	ko04138, map04138
FUN_000552	Ankyrin repeats (3 copies)	-	-	-	-

FUN_000555	RNA recognition motif	NOP12	-	ko:K14837	-
FUN_000556	Sds3-like	-	-	-	-
FUN_000557	Fungal specific transcription factor domain	-	-	-	-
FUN_000558	helix loop helix domain	-	-	-	-
FUN_000559	CCAAT-Binding transcription Factor	HAP2	-	ko:K08064	ko04612, ko05152, map04612, map05152
FUN_000562	Major Facilitator Superfamily	-	-	-	-
FUN_000563	SLA1 homology domain 1, SHD1	SLA1	-	ko:K20046	-
FUN_000564	NB-ARC domain	-	-	-	-
FUN_000565	Partial alpha/beta-hydrolase lipase region	-	-	-	-
FUN_000566	ATPase family associated with various cellular activities (AAA)	PEX6	-	ko:K13339	ko04146, map04146
FUN_000568	KR domain	-	-	-	-
FUN_000572	Major Facilitator Superfamily	-	-	-	-
FUN_000574	Cytochrome P450	-	-	-	-
FUN_000575	Fungal specific transcription factor domain	-	-	-	-
FUN_000576	Fungal specific transcription factor domain	-	-	-	-
FUN_000577	Belongs to the mitochondrial carrier (TC 2.A.29) family	-	-	ko:K15110	-
FUN_000578	CoA-transferase family III	-	2.8.3.13	ko:K18703	-
FUN_000579	homogentisate 1, 2-dioxygenase	-	1.13.11.5	ko:K00451	ko00350, ko00643, ko01100, ko01120, map00350, map00643, map01100, map01120
FUN_000580	Adenylosuccinate lyase C-terminus	-	-	-	-
FUN_000581	Belongs to the thiolase family	-	2.3.1.16	ko:K07513	ko00071, ko00280, ko00592, ko01040, ko01100, ko01110, ko01130, ko01212, ko03320, ko04146, map00071, map00280, map00592, map01040, map01100, map01110, map01130, map01212, map03320, map04146
FUN_000582	Iron-containing alcohol dehydrogenase	-	-	-	-
FUN_000583	Key enzyme for ketone body catabolism. Transfers the CoA moiety from succinate to acetoacetate. Formation of the enzyme-CoA intermediate proceeds via an unstable anhydride species formed between the carboxylate groups of the enzyme and substrate	-	2.8.3.5	ko:K01027	ko00072, ko00280, ko00650, map00072, map00280, map00650
FUN_000584	NAD(P)H-binding	-	-	-	-
FUN_000585	NAD-binding of NADP-dependent 3-hydroxyisobutyrate dehydrogenase	-	1.1.1.31	ko:K00020	ko00280, ko01100, map00280, map01100
FUN_000586	Enoyl-(Acyl carrier protein) reductase	-	-	-	-
FUN_000587	Belongs to the type-B carboxylesterase lipase family	-	-	-	-
FUN_000588	Metallo-beta-lactamase superfamily	-	-	-	-
FUN_000589	Cytochrome P450	-	-	-	-
FUN_000590	Glycoside hydrolase family 3 protein	-	3.2.1.37	ko:K15920	ko00520, ko01100, map00520, map01100
FUN_000592	acetylcholinesterase activity	-	3.1.1.7	ko:K01049	ko00564, ko04725, map00564, map04725
FUN_000593	Oxoglutarate and iron-dependent oxygenase degradation C-term	NUA3	-	-	-
FUN_000594	Belongs to the mitochondrial carrier (TC 2.A.29) family	TPC1	-	ko:K15108	-
FUN_000595	Taurine catabolism dioxygenase TauD, TfdA family	-	1.14.11.1	ko:K00471	ko00310, map00310
FUN_000596	GAL4-like Zn(II)2Cys6 (or C6 zinc) binuclear cluster DNA-binding domain	-	-	-	-
FUN_000597	Arylsulfotransferase (ASST)	-	-	-	-
FUN_000598	Belongs to the purine-cytosine permease (2.A.39) family	-	-	-	-
FUN_000599	HECT-like Ubiquitin-conjugating enzyme (E2)-binding	-	2.3.2.26	ko:K20803	-

FUN_000600	Polysaccharide biosynthesis protein	ERG26	1.1.1.170	ko:K07748	ko00100, ko01100, ko01130, map00100, map01100, map01130
FUN_000601	Belongs to the ubiquitin-conjugating enzyme family	-	2.3.2.23	ko:K10585	ko04120, map04120
FUN_000602	Sybindin-like family	-	-	ko:K20303	-
FUN_000603	Ssl1-like	RPN10	-	ko:K03029	ko03050, ko05169, map03050, map05169
FUN_000605	zinc finger	-	-	-	-
FUN_000606	Major Facilitator Superfamily	-	-	-	-
FUN_000609	helix loop helix domain	-	-	-	-
FUN_000611	RNA recognition motif	NSR1	-	ko:K11294	ko05130, map05130
FUN_000612	Belongs to the ALG6 ALG8 glucosyltransferase family	ALG6	2.4.1.267	ko:K03848	ko00510, ko01100, map00510, map01100
FUN_000613	Leucine Rich repeat	rna1	-	ko:K14319	ko03013, map03013
FUN_000615	Mechanosensitive ion channel	-	-	ko:K22048	-
FUN_000616	Tubulin is the major constituent of microtubules. It binds two moles of GTP, one at an exchangeable site on the beta chain and one at a non-exchangeable site on the alpha chain	TUB1	-	ko:K07374	ko04145, ko04210, ko04530, ko04540, ko05130, map04145, map04210, map04530, map04540, map05130
FUN_000617	Protein of unknown function (DUF4243)	-	-	-	-
FUN_000618	Uncharacterised conserved protein (DUF2156)	DPS1	6.1.1.12	ko:K01876, ko:K22503	ko00970, map00970
FUN_000619	Belongs to the cytochrome P450 family	-	1.14.13.12	ko:K07824	ko00362, ko00627, ko01100, ko01120, ko01220, map00362, map00627, map01100, map01120, map01220
FUN_000621	protein serine/threonine kinase activity	-	2.7.11.1	ko:K20875	-
FUN_000623	Heavy-metal-associated domain	-	-	-	-
FUN_000624	GRF zinc finger	-	-	-	-
FUN_000625	galactosyl transferase GMA12/MNN10 family	-	-	ko:K05531	ko00513, ko01100, map00513, map01100
FUN_000627	Glycosyl hydrolase family 61	-	-	-	-
FUN_000631	Beta-eliminating lyase	-	4.1.2.48	ko:K01620	ko00260, ko01100, ko01110, ko01120, ko01130, ko01230, map00260, map01100, map01110, map01120, map01130, map01230
FUN_000635	Methyltransferase domain	-	-	-	-
FUN_000636	FAD binding domain	-	-	-	-
FUN_000637	Zinc-binding dehydrogenase	-	1.1.1.2	ko:K00002	ko00010, ko00040, ko00561, ko00930, ko01100, ko01110, ko01120, ko01130, ko01220, map00010, map00040, map00561, map00930, map01100, map01110, map01120, map01130, map01220
FUN_000638	ankyrin repeat protein	-	-	-	-
FUN_000644	MmgE/PrpD family	-	4.1.1.6	ko:K17724	ko00660, map00660
FUN_000645	Belongs to the pirin family	PRN1	-	ko:K06911	-
FUN_000648	oxidoreductase activity	-	-	-	-
FUN_000649	FAD dependent oxidoreductase	-	-	-	-
FUN_000651	ankyrin repeat protein	-	-	-	-
FUN_000652	2OG-Fe(II) oxygenase superfamily	-	-	-	-
FUN_000654	Fungal specific transcription factor domain	-	-	-	-
FUN_000656	Peroxidase, family 2	-	-	-	-
FUN_000657	RTA1 like protein	-	-	-	-
FUN_000659	Belongs to the glycosyl hydrolase 31 family	-	-	-	-

FUN_000660	Common central domain of tyrosinase	-	1.14.18.1	ko:K00505	ko00350, ko00950, ko00965, ko01100, ko01110, ko04916, map00350, map00950, map00965, map01100, map01110, map04916
FUN_000661	'Conserved protein	-	-	-	-
FUN_000662	cytochrome P450	-	-	-	-
FUN_000669	Thioesterase superfamily	-	2.7.1.25	ko:K00860	ko00230, ko00920, ko01100, ko01120, map00230, map00920, map01100, map01120
FUN_000671	DNA replication factor Dna2	dna2	3.6.4.12	ko:K10742	ko03030, map03030
FUN_000672	Belongs to the mitochondrial carrier (TC 2.A.29) family	-	-	ko:K15110	-
FUN_000673	KR domain	TSC10	1.1.1.102, 1.1.1.56	ko:K00039, ko:K04708	ko00040, ko00600, ko01100, map00040, map00600, map01100
FUN_000674	Sas10 C-terminal domain	SAS10	-	ko:K14767	-
FUN_000675	Membrane magnesium transporter	-	-	-	-
FUN_000676	ammonium transporter	-	-	ko:K03320	-
FUN_000677	Serine/Threonine protein kinases, catalytic domain	SSK2	2.7.11.25	ko:K11230	ko02020, ko04011, map02020, map04011
FUN_000678	histidyl-tRNA synthetase	HTS1	6.1.1.21	ko:K01892	ko00970, map00970
FUN_000679	tRNA ligase	trl1	6.5.1.3	ko:K14679	-
FUN_000680	homogentisate 1, 2-dioxygenase	-	1.13.11.5	ko:K00451	ko00350, ko00643, ko01100, ko01120, map00350, map00643, map01100, map01120
FUN_000681	Nonessential protein required for the fusion of transport vesicles derived from the endocytic pathway with the Golgi complex	SFT2	-	-	-
FUN_000682	helix loop helix domain	-	-	-	-
FUN_000684	NADH:flavin oxidoreductase / NADH oxidase family	OYE32	1.3.1.42	ko:K05894	ko00592, ko01100, ko01110, map00592, map01100, map01110
FUN_000685	Plays a complex role in regulating the basal catalytic activity of the alpha subunit	CKB2	-	ko:K03115	ko03008, ko04064, ko04137, ko04139, ko04310, ko04520, ko04712, ko05162, ko05168, ko05169, map03008, map04064, map04137, map04139, map04310, map04520, map04712, map05162, map05168, map05169
FUN_000688	DNA replication regulator SLD3	-	-	ko:K10731	-
FUN_000689	peroxin 20	-	-	-	-
FUN_000690	Nucleoside-diphosphate-sugar epimerase	-	-	-	-
FUN_000691	rta1 domain protein	-	-	-	-
FUN_000692	Fungal specific transcription factor domain	-	-	-	-
FUN_000693	short chain dehydrogenase	-	-	-	-
FUN_000694	Zinc-binding domain present in Lin-11, Isl-1, Mec-3.	-	-	ko:K05760	ko04062, ko04370, ko04510, ko04670, ko04810, ko05100, ko05165, ko05203, ko05205, map04062, map04370, map04510, map04670, map04810, map05100, map05165, map05203, map05205
FUN_000695	Fungal specific transcription factor domain	-	-	-	-
FUN_000696	Ribosomal Proteins L2, RNA binding domain	RML2	-	-	-
FUN_000697	Belongs to the cyclin family	CLB2	-	ko:K02220	ko04011, ko04111, map04011, map04111
FUN_000698	ribosomal protein	-	-	ko:K02864	ko03010, map03010
FUN_000699	Anticodon binding domain	PRS	6.1.1.15	ko:K01881	ko00970, map00970
FUN_000700	RXT2-like, N-terminal	-	-	-	-
FUN_000701	Gryzun, putative trafficking through Golgi	-	-	ko:K20308	-

FUN_000702	Mitochondrial carrier required for the biosynthesis of heme, possibly by facilitating 5-aminolevulinate (ALA) production. May act by importing glycine into mitochondria or by exchanging glycine for ALA across the mitochondrial inner membrane	-	-	ko:K15118	-
FUN_000704	Chloride channel protein	-	-	ko:K05012	-
FUN_000705	pre-mrna splicing factor clf1	-	-	-	-
FUN_000706	HpcH/HpaI aldolase/citrate lyase family	-	4.1.2.52	ko:K02510	ko00350, ko01120, map00350, map01120
FUN_000707	Belongs to the glutaredoxin family	-	-	-	-
FUN_000708	CHORD	-	-	-	-
FUN_000709	Major Facilitator Superfamily	-	-	-	-
FUN_000710	14-3-3 protein	-	-	-	-
FUN_000711	Prp31 C terminal domain	PRP31	-	ko:K12844	ko03040, map03040
FUN_000712	HAT (Half-A-TPR) repeat	CLF1	-	ko:K12869	ko03040, map03040
FUN_000713	Belongs to the D-isomer specific 2-hydroxyacid dehydrogenase family	GOR1	1.1.1.26	ko:K00015	ko00630, ko01100, ko01110, ko01120, map00630, map01100, map01110, map01120
FUN_000714	GAL4-like Zn(II)2Cys6 (or C6 zinc) binuclear cluster DNA-binding domain	-	-	ko:K21632	-
FUN_000715	Belongs to the mitochondrial carrier (TC 2.A.29) family	-	-	ko:K15111	-
FUN_000718	Domain of unknown function (DUF4452)	-	-	-	-
FUN_000719	Belongs to the cyclin family	SSN8	-	ko:K15161	-
FUN_000720	Belongs to the mitochondrial carrier (TC 2.A.29) family	-	-	ko:K14684	-
FUN_000721	Belongs to the GroES chaperonin family	hsp10	-	ko:K04078	-
FUN_000722	PP-loop family	-	6.3.4.19	ko:K04075	-
FUN_000723	TRAF-type zinc finger	-	-	-	-
FUN_000724	SNARE domain	-	-	ko:K08501	ko04130, map04130
FUN_000725	Heterokaryon incompatibility protein Het-C	-	-	-	-
FUN_000728	Pleckstrin homology domain	-	-	-	-
FUN_000730	component of the eukaryotic translation initiation factor 3 (eIF-3) complex, which is involved in protein synthesis of a specialized repertoire of mRNAs and, together with other initiation factors, stimulates binding of mRNA and methionyl-tRNAi to the 40S ribosome. The eIF-3 complex specifically targets and initiates translation of a subset of mRNAs involved in cell proliferation	-	-	ko:K03247	ko03013, ko05162, map03013, map05162
FUN_000731	RIC1	RIC1	-	ko:K20476	-
FUN_000733	Peptidase inhibitor I78 family	-	-	-	-
FUN_000734	Glutathione S-transferase, N-terminal domain	-	2.5.1.18	ko:K00799	ko00480, ko00980, ko00982, ko00983, ko01524, ko05200, ko05204, ko05225, ko05418, map00480, map00980, map00982, map00983, map01524, map05200, map05204, map05225, map05418
FUN_000735	Required for replication-independent chromatin assembly and for the periodic repression of histone gene transcription during the cell cycle	HIR1	-	ko:K11293	-
FUN_000736	U1 zinc finger	-	1.6.99.1	ko:K00354, ko:K13220	-
FUN_000739	CPL (NUC119) domain	puf6	-	ko:K14844	-
FUN_000740	Ankyrin repeats (many copies)	-	-	ko:K06867	-
FUN_000741	PAP2 superfamily	-	-	-	-
FUN_000742	Required both for recombination and for the repair of DNA damage caused by X-rays	rhp51	-	ko:K04482	ko03440, ko03460, ko05200, ko05212, map03440, map03460, map05200, map05212

FUN_000743	Nucleoporin Nup120/160	-	-	ko:K14303	ko03013, map03013
FUN_000744	Belongs to the ubiquitin-conjugating enzyme family	-	2.3.2.23	ko:K10583	ko04120, map04120
FUN_000746	Importin-beta N-terminal domain	-	-	ko:K20224	-
FUN_000747	HORMA domain	-	-	ko:K02537, ko:K13728	ko04110, ko04111, ko04113, ko04114, ko04914, ko05100, ko05131, ko05166, map04110, map04111, map04113, map04114, map04914, map05100, map05131, map05166
FUN_000748	RING finger protein	-	2.3.2.31	ko:K11976	-
FUN_000750	Threonine synthase N terminus	THR4	4.2.3.1	ko:K01733	ko00260, ko00750, ko01100, ko01110, ko01120, ko01230, map00260, map00750, map01100, map01110, map01120, map01230
FUN_000751	Protein tyrosine kinase	PKC1	2.7.11.13	ko:K02677, ko:K18050	ko01521, ko04010, ko04011, ko04012, ko04014, ko04015, ko04020, ko04022, ko04066, ko04070, ko04071, ko04072, ko04139, ko04150, ko04151, ko04261, ko04270, ko04310, ko04360, ko04370, ko04371, ko04510, ko04530, ko04540, ko04650, ko04664, ko04666, ko04670, ko04713, ko04720, ko04723, ko04724, ko04725, ko04726, ko04727, ko04728, ko04730, ko04745, ko04750, ko04911, ko04912, ko04916, ko04918, ko04919, ko04921, ko04925, ko04926, ko04930, ko04931, ko04933, ko04960, ko04961, ko04970, ko04971, ko04972, ko05031, ko05032, ko05110, ko05130, ko05143, ko05146, ko05161, ko05164, ko05200, ko05205, ko05206, ko05214, ko05223, ko05225, ko05231, map01521, map04010, map04011, map04012, map04014, map04015, map04020, map04022, map04066, map04070, map04071, map04072, map04139, map04150, map04151, map04261, map04270, map04310, map04360, map04370, map04371, map04510, map04530, map04540, map04650, map04664, map04666, map04670, map04713, map04720, map04723, map04724, map04725, map04726, map04727, map04728, map04730, map04745, map04750, map04911, map04912, map04916, map04918, map04919, map04921, map04925, map04926, map04930, map04931, map04933, map04960, map04961, map04970, map04971, map04972, map05031, map05032, map05110, map05130, map05143, map05146, map05161, map05164, map05200, map05205, map05206, map05214, map05223, map05225, map05231
FUN_000754	Aldo/keto reductase family	-	1.1.1.156	ko:K18097	ko00561, ko01100, map00561, map01100
FUN_000755	N-terminal domain of NEFA-interacting nuclear protein NIP30	-	-	-	-
FUN_000756	Carbohydrate-binding module family 18	-	3.5.1.41	ko:K01452	ko00520, ko01100, map00520, map01100
FUN_000757	Sulfate permease family	-	-	ko:K03321	-
FUN_000758	GINS complex subunit 1	PSF1	-	ko:K10732	-
FUN_000759	HMG (high mobility group) box	-	-	-	-
FUN_000762	Beta-galactosidase	-	-	-	-
FUN_000763	Domain of unknown function (DUF3336)	TGL3	3.1.1.3	ko:K14675	ko00561, ko01100, map00561, map01100
FUN_000764	Cyclic phosphodiesterase-like protein	-	3.1.4.37	ko:K15435	-

FUN_000765	GLE1-like protein	-	-	ko:K18723	-
FUN_000766	RNB	SSD1	-	ko:K18748	-
FUN_000767	Ferritin-like domain	-	-	-	-
FUN_000768	Major Facilitator Superfamily	-	-	-	-
FUN_000769	Alpha/beta hydrolase family	-	-	-	-
FUN_000772	Allergen	-	-	-	-
FUN_000773	ATP citrate lyase citrate-binding	acl2	2.3.3.8	ko:K01648	ko00020, ko00720, ko01100, ko01110, ko01120, ko01130, map00020, map00720, map01100, map01110, map01120, map01130
FUN_000774	Citrate synthase, C-terminal domain	acl1	2.3.3.8	ko:K01648	ko00020, ko00720, ko01100, ko01110, ko01120, ko01130, map00020, map00720, map01100, map01110, map01120, map01130
FUN_000775	Belongs to the histidine acid phosphatase family	-	-	-	-
FUN_000777	AMP-binding enzyme	-	-	-	-
FUN_000781	Belongs to the aldehyde dehydrogenase family	ALD5	1.2.1.3	ko:K00128	ko00010, ko00053, ko00071, ko00280, ko00310, ko00330, ko00340, ko00380, ko00410, ko00561, ko00620, ko00625, ko00903, ko00981, ko01100, ko01110, ko01120, ko01130, map00010, map00053, map00071, map00280, map00310, map00330, map00340, map00380, map00410, map00561, map00620, map00625, map00903, map00981, map01100, map01110, map01120, map01130
FUN_000783	Vacuolar protein sorting protein 11 C terminal	VPS11	-	ko:K20179	ko04138, map04138
FUN_000785	G protein-coupled glucose receptor regulating Gpa2 C-term	-	-	-	-
FUN_000786	Sorting nexin C terminal	TRM8	-	ko:K17887	-
FUN_000787	KR domain	RDH1	1.3.1.33	ko:K00218	ko00860, ko01100, ko01110, map00860, map01100, map01110
FUN_000789	Belongs to the cytochrome P450 family	-	1.14.14.23	ko:K00489	ko00120, ko00140, ko01100, ko03320, ko04976, ko04979, map00120, map00140, map01100, map03320, map04976, map04979
FUN_000790	Enoyl-(Acyl carrier protein) reductase	-	1.1.1.300	ko:K11153	ko00830, ko01100, map00830, map01100
FUN_000791	Belongs to the WD repeat SEC13 family	SEC13	-	ko:K14004	ko03013, ko04141, ko04150, map03013, map04141, map04150
FUN_000793	Glucose sorbosone dehydrogenase	-	-	-	-
FUN_000795	ATP-binding cassette, sub-family F, member 3	GCN20	-	ko:K06158	-
FUN_000796	ABC transporter	GCN20	-	ko:K06158	-
FUN_000797	Adenosylhomocysteinase	SAH1	3.3.1.1	ko:K01251	ko00270, ko01100, map00270, map01100
FUN_000798	3-beta hydroxysteroid dehydrogenase/isomerase family	-	-	-	-
FUN_000799	SNF5 / SMARCB1 / INI1	SFH1	-	ko:K11770	-
FUN_000802	SURF4 family	ERV29	-	ko:K20369	-
FUN_000803	Inosine-uridine preferring nucleoside hydrolase	-	-	-	-
FUN_000805	CFEM domain	-	-	-	-
FUN_000806	WD domain, G-beta repeat	PRP46	-	ko:K11723, ko:K12862	ko03040, ko05225, map03040, map05225
FUN_000807	BAH domain	ORC1	-	ko:K02603	ko04110, ko04111, ko04113, map04110, map04111, map04113
FUN_000808	basic region leucin zipper	-	-	-	-
FUN_000809	DDHD	-	-	-	-

FUN_000812	Belongs to the glycosyl hydrolase 17 family	BGL2	3.2.1.58	ko:K01210	ko00500, map00500
FUN_000813	Epoxide hydrolase N terminus	-	-	-	-
FUN_000814	Heterokaryon incompatibility protein (HET)	-	-	-	-
FUN_000815	PHD-finger	-	2.7.7.6	ko:K00960	-
FUN_000816	Ion transport protein	CCH1	-	ko:K21864	-
FUN_000817	Arrestin (or S-antigen), N-terminal domain	-	-	-	-
FUN_000818	Histone-like transcription factor (CBF/NF-Y) and archaeal histone	-	2.7.7.7	ko:K02326	ko00230, ko00240, ko01100, ko03030, ko03410, ko03420, ko05166, map00230, map00240, map01100, map03030, map03410, map03420, map05166
FUN_000822	Beta-lactamase	-	-	-	-
FUN_000823	protein lysine methyltransferase SET5	SET5	-	ko:K07117, ko:K11426	-
FUN_000824	FAD binding domain	-	-	-	-
FUN_000827	short chain dehydrogenase	-	-	-	-
FUN_000830	Brix	IMP4	-	ko:K14561	ko03008, map03008
FUN_000831	Helicase associated domain (HA2) Add an annotation	PRP43	3.6.4.13	ko:K12820	ko03040, map03040
FUN_000832	Belongs to the class I-like SAM-binding methyltransferase superfamily	-	2.1.1.320	ko:K02516	ko03013, ko04011, ko04111, map03013, map04011, map04111
FUN_000834	integral membrane protein	-	-	-	-
FUN_000835	Flavin containing amine oxidoreductase	-	-	-	-
FUN_000837	glucosylceramidase activity	-	3.2.1.45	ko:K01201	ko00511, ko00600, ko01100, ko04142, map00511, map00600, map01100, map04142
FUN_000839	Subunit of the peripheral V1 complex of vacuolar ATPase. Subunit C is necessary for the assembly of the catalytic sector of the enzyme and is likely to have a specific function in its catalytic activity. V-ATPase is responsible for acidifying a variety of intracellular compartments in eukaryotic cells	VMA5	-	ko:K02148	ko00190, ko01100, ko04145, ko04150, ko04721, ko04966, ko05110, ko05120, ko05323, map00190, map01100, map04145, map04150, map04721, map04966, map05110, map05120, map05323
FUN_000840	The glycine cleavage system catalyzes the degradation of glycine	GCV1	2.1.2.10	ko:K00605	ko00260, ko00630, ko00670, ko01100, ko01110, ko01130, ko01200, map00260, map00630, map00670, map01100, map01110, map01130, map01200
FUN_000841	Heterokaryon incompatibility protein (HET)	-	-	-	-
FUN_000842	Pyridine nucleotide-disulphide oxidoreductase	-	-	ko:K22124	-
FUN_000843	Alpha/beta hydrolase family	-	-	-	-
FUN_000845	Protein of unknown function (DUF3605)	-	-	-	-
FUN_000846	Belongs to the short-chain dehydrogenases reductases (SDR) family	-	1.1.1.100	ko:K00059	ko00061, ko00333, ko00780, ko01040, ko01100, ko01130, ko01212, map00061, map00333, map00780, map01040, map01100, map01130, map01212
FUN_000847	Ubiquitin-like 1-activating enzyme E1 B	UBA2	6.2.1.45	ko:K10685	ko04120, map04120
FUN_000848	Potential Queuosine, Q, salvage protein family	-	-	-	-
FUN_000849	Domain of unknown function (DUF3337)	-	-	ko:K15361	ko03460, map03460
FUN_000850	TAP-like protein	-	-	-	-
FUN_000852	Belongs to the TRAFAC class myosin-kinesin ATPase superfamily. Kinesin family	-	-	ko:K10395	-
FUN_000853	HIT zinc finger	BCD1	-	-	-
FUN_000854	Domain found in a variety of signalling proteins, always encircled by uDENN and	-	-	-	-
FUN_000855	Glycoside hydrolase family 16 protein	-	-	-	-
FUN_000856	Belongs to the peptidase M49 family	YOL057	3.4.14.4	ko:K01277	-
FUN_000858	-o-)-methyltransferase	-	-	-	-

FUN_000859	cytochrome P450	-	-	-	-
FUN_000860	Tetratricopeptide repeat	-	-	-	-
FUN_000861	Nodulin-like	MCH1	-	ko:K10882	ko03440, ko03460, map03440, map03460
FUN_000862	3' exoribonuclease family, domain 1	RRP42	-	ko:K12589	ko03018, map03018
FUN_000866	G-beta repeat protein	-	-	-	-
FUN_000867	Belongs to the MT-A70-like family	-	-	-	-
FUN_000868	Belongs to the mitochondrial carrier (TC 2.A.29) family	LEU5	-	ko:K15084	-
FUN_000870	serine threonine-protein phosphatase	SIT4	3.1.3.16	ko:K15427, ko:K15498	-
FUN_000871	Smg-4/UPF3 family	-	-	ko:K14328	ko03013, ko03015, map03013, map03015
FUN_000872	Serine/Threonine protein kinases, catalytic domain	IME2	2.7.11.1	ko:K12765	ko04113, map04113
FUN_000873	Belongs to the NARF family	NAR1	-	-	-
FUN_000874	Glycosyltransferase family 25 protein	-	-	-	-
FUN_000875	Carbon-nitrogen hydrolase	NIT2	3.5.1.6	ko:K01431, ko:K11206	ko00240, ko00410, ko00770, ko00983, ko01100, map00240, map00410, map00770, map00983, map01100
FUN_000876	TATA element modulatory factor 1 DNA binding	-	-	ko:K20286	-
FUN_000877	Ankyrin repeat	NAS6	-	ko:K06694	-
FUN_000879	Major Facilitator Superfamily	MDR1	-	ko:K08158	-
FUN_000880	Sugar (and other) transporter	-	-	-	-
FUN_000881	helix loop helix domain	-	-	-	-
FUN_000882	Component of the NOP7 complex, which is required for maturation of the 25S and 5.8S ribosomal RNAs and formation of the 60S ribosome	ERB1	-	ko:K14824	-
FUN_000883	helicase superfamily c-terminal domain	PRP5	3.6.4.13	ko:K12811	ko03040, map03040
FUN_000884	Glucose sorbosone	-	-	-	-
FUN_000885	Voltage-dependent anion channel	-	-	-	-
FUN_000886	Major intrinsic protein	-	-	ko:K03441	-
FUN_000887	Vacuolar protein sorting-associated protein 26	vps26	-	ko:K18466	ko04144, map04144
FUN_000888	Belongs to the gluconokinase GntK GntV family	-	2.7.1.12	ko:K00851	ko00030, ko01100, ko01110, ko01120, ko01130, ko01200, map00030, map01100, map01110, map01120, map01130, map01200
FUN_000889	Dihydrolipoamide acetyltransferase component of pyruvate dehydrogenase complex	-	2.3.1.168	ko:K09699	ko00280, ko00640, ko01100, ko01110, ko01130, map00280, map00640, map01100, map01110, map01130
FUN_000890	ESCRT-II complex subunit	-	-	ko:K12189	ko04144, map04144
FUN_000891	Regulator of Ty1 transposition protein 107 BRCT domain	ESC4	-	ko:K20780	-
FUN_000892	Alpha/beta hydrolase family	-	-	-	-
FUN_000893	Nuclear pore assembly and biogenesis	-	-	-	-
FUN_000894	Belongs to the RNR ribonuclease family	DIS3	-	ko:K12585	ko03018, map03018
FUN_000895	V-ATPase subunit H	VMA13	-	ko:K02144	ko00190, ko01100, ko04142, ko04145, ko04150, ko04721, ko05110, ko05120, ko05152, ko05323, map00190, map01100, map04142, map04145, map04150, map04721, map05110, map05120, map05152, map05323
FUN_000896	Chromatin remodelling complex Rsc7/Swp82 subunit	-	-	ko:K11761	-
FUN_000897	Pre-mRNA-splicing factor SF3a complex subunit 2 (Prp11)	sap62	-	ko:K12826	ko03040, map03040

FUN_000898	CAP_GLY	-	-	ko:K21768	-
FUN_000901	Amidase	-	3.5.1.4	ko:K01426	ko00330, ko00360, ko00380, ko00627, ko00643, ko01120, map00330, map00360, map00380, map00627, map00643, map01120
FUN_000902	Amidase	-	3.5.1.4	ko:K01426	ko00330, ko00360, ko00380, ko00627, ko00643, ko01120, map00330, map00360, map00380, map00627, map00643, map01120
FUN_000903	Aldo/keto reductase family	-	1.1.1.2	ko:K00002	ko00010, ko00040, ko00561, ko00930, ko01100, ko01110, ko01120, ko01130, ko01220, map00010, map00040, map00561, map00930, map01100, map01110, map01120, map01130, map01220
FUN_000904	Glutathione S-transferase, C-terminal domain	-	-	-	-
FUN_000908	Modifier of rudimentary (Mod(r)) protein	-	-	-	-
FUN_000909	intracellular protein transport-like protein	-	-	-	-
FUN_000910	Extension to Ser/Thr-type protein kinases	CBK1	-	ko:K08286	-
FUN_000913	Belongs to the ABC transporter superfamily. ABCG family. PDR (TC 3.A.1.205) subfamily	CDR1	-	ko:K08711, ko:K08712	ko02010, map02010
FUN_000915	Polysaccharide lyase family 3 protein	plyE	4.2.2.2	ko:K01728	ko00040, ko02024, map00040, map02024
FUN_000916	vegetative cell wall protein gp1	-	-	-	-
FUN_000918	Major Facilitator Superfamily	-	-	-	-
FUN_000919	Integral membrane protein DUF92	-	-	-	-
FUN_000920	Integral membrane protein DUF92	-	-	-	-
FUN_000921	Ubiquitin-2 like Rad60 SUMO-like	-	-	-	-
FUN_000925	Src homology 3 domains	-	-	-	-
FUN_000929	zinc finger	SUR1	-	-	-
FUN_000931	NMDA receptor-regulated protein 1	-	-	ko:K20792	-
FUN_000932	Formamidopyrimidine-DNA glycosylase N-terminal domain	-	3.2.2.23, 4.2.99.18	ko:K10563	ko03410, map03410
FUN_000933	Belongs to the arginase family	-	3.5.3.11	ko:K01480	ko00330, ko01100, map00330, map01100
FUN_000935	DNA polymerase X family	-	2.7.7.7	ko:K03512, ko:K10981	ko03410, ko03450, map03410, map03450
FUN_000936	ATP10 protein	ATP10	-	ko:K18192	-
FUN_000937	Involved in transport from the ER to the Golgi apparatus as well as in intra-Golgi transport. It belongs to a super-family of proteins called t-SNAREs or soluble NSF (N-ethylmaleimide- sensitive factor) attachment protein receptor	GOS1	-	ko:K08495	ko04130, map04130
FUN_000938	Ribosomal L29 protein	rpl35	-	ko:K02918	ko03010, map03010
FUN_000939	Belongs to the GHMP kinase family. Mevalonate kinase subfamily	ERG8	2.7.4.2	ko:K00938	ko00900, ko01100, ko01110, ko01130, map00900, map01100, map01110, map01130
FUN_000940	Pentacotripeptide-repeat region of PRORP	-	-	-	-
FUN_000942	Sir2 family	SIR2	-	ko:K11121	ko00760, ko01100, ko04213, map00760, map01100, map04213
FUN_000943	Ribonuclease P 40kDa (Rpp40) subunit	-	3.1.26.5	ko:K14530	ko03008, ko03013, map03008, map03013
FUN_000945	Belongs to the multicopper oxidase family	lcc1	-	-	-
FUN_000947	Ubiquitin homologues	-	-	-	-

FUN_000948	DNA ligase	cdc17	6.5.1.1, 6.5.1.6, 6.5.1.7	ko:K10747	ko03030, ko03410, ko03420, ko03430, map03030, map03410, map03420, map03430
FUN_000949	Caffeine-induced death protein 2	-	-	-	-
FUN_000950	Belongs to the universal ribosomal protein uL1 family	RPL10A	-	ko:K02865	ko03010, map03010
FUN_000951	Cell differentiation family, Rcd1-like	rcd1	-	ko:K12606	ko03018, map03018
FUN_000952	Serine threonine protein kinase	KIN2	2.7.11.1	ko:K19852	-
FUN_000953	Protein of unknown function (DUF1604)	-	-	ko:K13123	-
FUN_000955	integral membrane protein	-	-	-	-
FUN_000957	C2H2-type zinc finger	-	-	-	-
FUN_000958	Aldo/keto reductase family	-	1.1.1.65	ko:K05275	ko00750, ko01100, ko01120, map00750, map01100, map01120
FUN_000959	Ceramidase	-	-	ko:K04711	ko00600, map00600
FUN_000960	Domain of unknown function (DUF4139)	-	-	-	-
FUN_000962	Belongs to the small GTPase superfamily. Arf family	ARF1	-	ko:K07937, ko:K07977	ko04072, ko04144, ko05110, ko05134, map04072, map04144, map05110, map05134
FUN_000963	Mob1/phocein family	-	-	-	-
FUN_000964	Leo1-like protein	LEO1	-	ko:K15177	-
FUN_000965	homogentisate 1, 2-dioxygenase	-	1.13.11.5	ko:K00451	ko00350, ko00643, ko01100, ko01120, map00350, map00643, map01100, map01120
FUN_000966	Fumarylacetoacetase N-terminal	-	3.7.1.2	ko:K01555	ko00350, ko00643, ko01100, ko01120, map00350, map00643, map01100, map01120
FUN_000967	Cytochrome P450	-	1.14.14.54	ko:K10437	ko00360, ko00643, ko01100, ko01120, map00360, map00643, map01100, map01120
FUN_000968	TRAM, LAG1 and CLN8 homology domains.	-	-	-	-
FUN_000969	RNA recognition motif	-	-	ko:K13093	-
FUN_000970	Copper/zinc superoxide dismutase (SODC)	SOD4	1.15.1.1	ko:K04565	ko04146, ko04213, ko05014, ko05016, ko05020, map04146, map04213, map05014, map05016, map05020
FUN_000972	O-methyltransferase	-	-	-	-
FUN_000973	Permease family	-	-	ko:K06901	-
FUN_000975	AhpC/TSA antioxidant enzyme	-	-	-	-
FUN_000977	axonemal central apparatus assembly	-	-	ko:K18626	-
FUN_000978	Belongs to the major facilitator superfamily. Sugar transporter (TC 2.A.1.1) family	-	-	-	-
FUN_000979	Prefoldin subunit	-	-	ko:K09548	-
FUN_000980	Tubulin is the major constituent of microtubules. It binds two moles of GTP, one at an exchangeable site on the beta chain and one at a non-exchangeable site on the alpha	TUB2	-	ko:K07375	ko04145, ko04540, ko05130, map04145, map04540, map05130
FUN_000981	Catalyzes the second two steps of the methylation pathway of phosphatidylcholine biosynthesis, the SAM-dependent methylation of phosphatidylmonomethylethanolamine (PMME) to phosphatidyldimethylethanolamine (PDME) and of PDME to phosphatidylcholine (PC)	OPI3	2.1.1.17, 2.1.1.71	ko:K00551	ko00564, ko01100, ko01110, map00564, map01100, map01110
FUN_000982	Transmembrane amino acid transporter protein	-	-	-	-
FUN_000983	Protein tyrosine kinase	ppk32	-	ko:K17541	-
FUN_000984	Belongs to the class-IV pyridoxal-phosphate-dependent aminotransferase family	BAT2	2.6.1.42	ko:K00826	ko00270, ko00280, ko00290, ko00770, ko01100, ko01110, ko01130, ko01210, ko01230, map00270, map00280, map00290, map00770, map01100, map01110, map01130, map01210, map01230

FUN_000985	HMG-box domain	-	-	-	-
FUN_000986	Protein tyrosine kinase	-	2.7.11.1	ko:K08860, ko:K16194, ko:K16195	ko04137, ko04140, ko04141, ko04210, ko04214, ko04217, ko04932, ko05010, ko05160, ko05162, ko05164, ko05165, ko05167, ko05168, ko05169, ko05203, map04137, map04140, map04141, map04210, map04214, map04217, map04932, map05010, map05160, map05162, map05164, map05165, map05167, map05168, map05169, map05203
FUN_000988	Amino acid permease	AGP2	-	ko:K16261	-
FUN_000989	tRNA-splicing endonuclease subunit sen54 N-term	SEN54	-	ko:K15326	-
FUN_000990	KR domain	-	-	-	-
FUN_000991	Belongs to the inositol phosphokinase (IPK) family	KCS1	2.7.4.21	ko:K07756	ko04070, ko04138, map04070, map04138
FUN_000993	Leucine-rich repeats, outliers	NUD1	-	ko:K20237	ko04392, map04392
FUN_000994	Myosin-binding striated muscle assembly central	SHE4	-	ko:K21991	-
FUN_000995	Belongs to the EF-1-beta EF-1-delta family	-	-	ko:K03232	-
FUN_000996	Belongs to the ClpA ClpB family	HSP78	3.6.1.3	ko:K01509, ko:K03695	ko00230, ko04213, ko04742, map00230, map04213, map04742
FUN_000997	ATPase domain of DNA mismatch repair MUTS family	-	-	ko:K08741	-
FUN_000998	Aflatoxin regulatory protein	-	-	-	-
FUN_001000	Belongs to the major facilitator superfamily. Sugar transporter (TC 2.A.1.1) family	-	-	-	-
FUN_001001	Required for nuclear transport of RNA pol II C-terminus 1	-	-	-	-
FUN_001002	LSM domain	-	-	ko:K11098	ko03040, map03040
FUN_001003	Pyridoxamine phosphate oxidase	-	-	-	-
FUN_001005	Eukaryotic protein of unknown function (DUF866)	-	-	-	-
FUN_001007	Sugar (and other) transporter	-	-	ko:K08157	-
FUN_001009	XAP5, circadian clock regulator	-	-	ko:K13119	-
FUN_001010	Glycerophosphoryl diester phosphodiesterase family	GDE1	3.1.4.46	ko:K18696	ko00564, map00564
FUN_001012	Haloacid dehalogenase-like hydrolase	-	3.1.3.96	ko:K17623	-
FUN_001013	Glycoside hydrolase family 2 protein	-	3.2.1.25	ko:K01192	ko00511, ko04142, map00511, map04142
FUN_001015	NAD binding domain of 6-phosphogluconate dehydrogenase	-	-	-	-
FUN_001017	myosin class II heavy chain	-	-	-	-
FUN_001018	Major Facilitator Superfamily	-	-	-	-
FUN_001019	Casein kinase II beta 2 subunit	-	-	-	-
FUN_001020	Domain in the RNA-binding Lupus La protein; unknown function	-	-	ko:K18757	-
FUN_001023	Diacylglycerol kinase catalytic domain (presumed)	LCB4	2.7.1.91	ko:K04718	ko00600, ko01100, ko04020, ko04071, ko04072, ko04370, ko04371, ko04666, ko05152, map00600, map01100, map04020, map04071, map04072, map04370, map04371, map04666, map05152
FUN_001024	The coatomer is a cytosolic protein complex that binds to dilysine motifs and reversibly associates with Golgi non- clathrin-coated vesicles, which further mediate biosynthetic protein transport from the ER, via the Golgi up to the trans Golgi network. Coatomer complex is required for budding from Golgi membranes, and is essential for the retrograde Golgi-to-ER transport of dilysine-tagged proteins	SEC26	-	ko:K17301	-
FUN_001025	Mago nashi protein	-	-	ko:K12877	ko03013, ko03015, ko03040, map03013, map03015, map03040
FUN_001026	Vacuolar import and degradation protein	-	-	-	-

FUN_001027	Catalyzes the formation of N(7)-methylguanine at position 46 (m7G46) in tRNA	TRM8	2.1.1.33	ko:K03439, ko:K17887	-
FUN_001028	Nucleolar protein, Nop52	-	-	ko:K14849	-
FUN_001029	Pre-SET motif	-	2.1.1.43	ko:K11419	ko00310, map00310
FUN_001030	Glycoside hydrolase family 105 protein	-	3.2.1.172	ko:K15532	-
FUN_001031	Vacuolar sorting protein 9 (VPS9) domain	-	-	ko:K02948	ko03010, map03010
FUN_001032	Belongs to the oxygen-dependent FAD-linked oxidoreductase family	-	-	-	-
FUN_001033	Belongs to the class I-like SAM-binding methyltransferase superfamily. RNA M5U methyltransferase family	TRM2	2.1.1.35	ko:K15331	-
FUN_001034	Belongs to the universal ribosomal protein uS5 family	MRPS5	-	ko:K02988	ko03010, map03010
FUN_001035	Serine/Threonine protein kinases, catalytic domain	NRC2	-	ko:K08286	-
FUN_001037	Belongs to the MRE11 RAD32 family	MRE11	-	ko:K10865	ko03440, ko03450, ko04218, map03440, map03450, map04218
FUN_001038	Belongs to the universal ribosomal protein uS19 family	RSM19	-	-	-
FUN_001039	Functions as a component of the DNA-binding general transcription factor complex TFIID and the transcription regulatory histone acetylation (HAT) complexes SAGA and SLIK. Binding of TFIID to a promoter (with or without TATA element) is the initial step in preinitiation complex (PIC) formation. TFIID plays a key role in the regulation of gene expression by RNA polymerase II through different activities such as transcription activator interaction, core promoter recognition and selectivity, TFIIA and TFIIB interaction, chromatin modification (histone acetylation), facilitation of DNA opening and initiation of transcription. SAGA is required for recruitment of the	-	-	ko:K03134	ko03022, ko05168, map03022, map05168
FUN_001041	Belongs to the actin family	ARP3	-	ko:K18584	ko04138, ko04530, map04138, map04530
FUN_001042	Asparaginase	-	-	ko:K08657	-
FUN_001043	Transcription factor Tfb4	TFB4	-	ko:K03143	ko03022, ko03420, ko05203, map03022, map03420, map05203
FUN_001044	Pyridine nucleotide-disulphide oxidoreductase	-	1.14.13.22	ko:K03379	ko00930, ko01120, ko01220, map00930, map01120, map01220
FUN_001045	Plays a complex role in regulating the basal catalytic activity of the alpha subunit	-	-	-	-
FUN_001046	Plays a complex role in regulating the basal catalytic activity of the alpha subunit	CKB1	-	ko:K03115	ko03008, ko04064, ko04137, ko04139, ko04310, ko04520, ko04712, ko05162, ko05168, ko05169, map03008, map04064, map04137, map04139, map04310, map04520, map04712, map05162, map05168, map05169
FUN_001048	Enoyl-(Acyl carrier protein) reductase	-	-	-	-
FUN_001049	ribosomal protein	RPL26A	-	ko:K02898	ko03010, map03010
FUN_001050	DNA binding domain with preference for A/T rich regions	-	-	-	-
FUN_001051	DRG Family Regulatory Proteins, Tma46	TMA46	-	-	-
FUN_001052	Phenol hydroxylase, C-terminal dimerisation domain	-	-	-	-
FUN_001053	cytochrome P450 family	-	-	-	-
FUN_001054	Domain in Tre-2, BUB2p, and Cdc16p. Probable Rab-GAPs.	-	-	ko:K20176	-
FUN_001055	SprT homologues.	-	-	-	-
FUN_001056	Ribosomal protein L23	MRP20	-	ko:K02892	ko03010, map03010
FUN_001058	Complex I intermediate-associated protein 30 (CIA30)	-	-	-	-
FUN_001059	GAL4-like Zn(II)2Cys6 (or C6 zinc) binuclear cluster DNA-binding domain	-	-	-	-

FUN_001060	basic region leucin zipper	GCN4	-	ko:K09464	ko04138, map04138
FUN_001061	Heterokaryon incompatibility protein (HET)	-	-	-	-
FUN_001062	Domain of unknown function (DUF202)	-	-	-	-
FUN_001063	Replication protein A C terminal	ssb2	-	ko:K10739	ko03030, ko03420, ko03430, ko03440, ko03460, map03030, map03420, map03430, map03440, map03460
FUN_001064	atp-dependent rna helicase dbp10	DBP10	3.6.4.13	ko:K14808	-
FUN_001065	Required for 40S ribosome biogenesis. Involved in nucleolar processing of pre-18S ribosomal RNA and ribosome assembly	KRR1	-	ko:K06961	-
FUN_001066	Domain of Kin17 curved DNA-binding protein	-	-	ko:K13102	-
FUN_001067	SET (Su(var)3-9, Enhancer-of-zeste, Trithorax) domain	-	-	-	-
FUN_001070	Belongs to the glycosyl hydrolase 47 family	-	3.2.1.113	ko:K01230	ko00510, ko00513, ko01100, ko04141, map00510, map00513, map01100, map04141
FUN_001071	Prefoldin subunit	GIM5	-	ko:K04797	-
FUN_001072	Capsule polysaccharide biosynthesis protein	-	-	-	-
FUN_001073	Cupin-like domain	-	-	-	-
FUN_001075	NAD dependent epimerase dehydratase family protein	-	-	-	-
FUN_001076	Binding domain of DNA repair protein Ercc1 (rad10/Swi10)	RAD10	-	ko:K10849	ko01524, ko03420, ko03460, map01524, map03420, map03460
FUN_001077	Rox3 mediator complex subunit	-	-	-	-
FUN_001079	RNA helicase	DBP4	3.6.4.13	ko:K14776	-
FUN_001080	DnaJ domain	spf31	-	ko:K09528	-
FUN_001081	COG0454 Histone acetyltransferase HPA2 and related acetyltransferases	-	-	-	-
FUN_001082	Belongs to the oxygen-dependent FAD-linked oxidoreductase family	-	-	-	-
FUN_001084	Belongs to the cytochrome P450 family	-	1.14.14.54	ko:K10437	ko00360, ko00643, ko01100, ko01120, map00360, map00643, map01100, map01120
FUN_001087	Arrestin (or S-antigen), C-terminal domain	-	-	-	-
FUN_001088	PPIases accelerate the folding of proteins. It catalyzes the cis-trans isomerization of proline imidic peptide bonds in oligopeptides	CYP10	5.2.1.8	ko:K01802, ko:K12734	-
FUN_001089	Duplicated domain in the epidermal growth factor- and elongation factor-1alpha-binding protein Zpr1. Also present in archaeal proteins.	ZPR1	-	ko:K06874	-
FUN_001090	RNAse P Rpr2/Rpp21/SNM1 subunit domain	-	-	ko:K14531	ko03008, map03008
FUN_001091	Lariat debranching enzyme, C-terminal domain	DBR1	-	ko:K18328	-
FUN_001092	Major Facilitator Superfamily	-	-	-	-
FUN_001093	Belongs to the aldehyde dehydrogenase family	PUT2	1.2.1.88	ko:K00294	ko00250, ko00330, ko01100, map00250, map00330, map01100
FUN_001094	GNL3L/Grn1 putative GTPase	NUG1	-	ko:K14538	ko03008, map03008
FUN_001095	Haloacid dehalogenase-like hydrolase	GPP1	3.1.3.21, 3.1.3.68	ko:K01111, ko:K06116, ko:K06117	ko00561, ko01100, map00561, map01100
FUN_001096	Homeodomain	-	-	-	-
FUN_001097	Catalyzes the synthesis of activated sulfate	MET14	2.7.1.25	ko:K00860	ko00230, ko00920, ko01100, ko01120, map00230, map00920, map01100, map01120
FUN_001099	SYF2 splicing factor	syf2	-	ko:K12868	ko03040, map03040
FUN_001100	pre-rrna-processing protein esf2	ESF2	-	ko:K14785	-
FUN_001101	N-terminal domain of CBF1 interacting co-repressor CIR	CWC25	-	-	-

FUN_001102	WD40 repeats	-	-	-	-
FUN_001103	Belongs to the mitochondrial carrier (TC 2.A.29) family	PET8	-	ko:K15111	-
FUN_001106	Origin recognition complex subunit 6 (ORC6)	-	-	ko:K02608	ko04110, ko04111, ko04113, map04110, map04111, map04113
FUN_001108	pre-mRNA splicing factor component	CEF1	-	ko:K12860	ko03040, map03040
FUN_001109	Domain of unknown function (DUF3337)	-	-	ko:K15361	ko03460, map03460
FUN_001112	polyketide synthase	-	-	-	-
FUN_001113	alpha-1, 3-glucan synthase	ags1	2.4.1.183	ko:K00749	-
FUN_001114	FAD binding domain	-	-	-	-
FUN_001115	ABC transporter transmembrane region	MDL1	3.6.1.3	ko:K01509, ko:K02021, ko:K05657	ko00230, ko02010, ko04742, map00230, map02010, map04742
FUN_001116	Cdc37 C terminal domain	CDC37	-	ko:K09554	ko04151, map04151
FUN_001117	RPR	RTT103	-	ko:K15559	-
FUN_001118	Clathrin is the major protein of the polyhedral coat of coated pits and vesicles	clc1	-	-	-
FUN_001120	Glutathione-dependent formaldehyde-activating enzyme	-	-	-	-
FUN_001121	Calcineurin-like phosphoesterase	-	-	-	-
FUN_001122	Cation efflux family	-	-	ko:K14688	ko04978, map04978
FUN_001123	Acyltransferase family	-	-	-	-
FUN_001124	Glutathione S-transferase, N-terminal domain	-	1.8.5.7	ko:K07393	-
FUN_001125	protein import into mitochondrial matrix	TIMM17A	-	ko:K16608, ko:K17795, ko:K19382	-
FUN_001126	NADH-ubiquinone oxidoreductase 9.5 kDa subunit	N19M	-	-	-
FUN_001127	Essential subunit of the N-oligosaccharyl transferase (OST) complex which catalyzes the transfer of a high mannose oligosaccharide from a lipid-linked oligosaccharide donor to an asparagine residue within an Asn-X-Ser Thr consensus motif in nascent	WBP1	-	ko:K12670	ko00510, ko00513, ko01100, ko04141, map00510, map00513, map01100, map04141
FUN_001128	parasitic phase-specific protein psp-1	-	-	-	-
FUN_001130	Serine aminopeptidase, S33	-	3.4.11.5	ko:K01259	ko00330, map00330
FUN_001131	Alcohol dehydrogenase GroES-like domain	-	-	-	-
FUN_001133	Stealth protein CR1, conserved region 1	-	-	-	-
FUN_001134	Enoyl-CoA hydratase/isomerase	-	-	-	-
FUN_001135	Belongs to the acyl-CoA oxidase family	-	1.3.3.6	ko:K00232	ko00071, ko00592, ko01040, ko01100, ko01110, ko01212, ko03320, ko04024, ko04146, map00071, map00592, map01040, map01100, map01110, map01212, map03320, map04024, map04146
FUN_001136	Cytoplasmic phospholipase A2, catalytic subunit	-	-	-	-
FUN_001137	AMP-binding enzyme C-terminal domain	-	-	ko:K18660	ko00280, map00280
FUN_001140	Glycoside hydrolase family 64 protein	-	-	-	-
FUN_001141	Protein of unknown function (DUF1593)	-	-	-	-
FUN_001142	Belongs to the mitochondrial carrier (TC 2.A.29) family	-	3.1.1.23	ko:K13704	-
FUN_001143	NUDE protein, C-terminal conserved region	NDE1	-	ko:K16738	-

FUN_001144	Acetyl-CoA carboxylase, central region	ACC1	2.1.3.15, 6.3.4.14, 6.4.1.2	ko:K11262	ko00061, ko00254, ko00620, ko00640, ko01100, ko01110, ko01212, ko04152, ko04910, ko04922, map00061, map00254, map00620, map00640, map01100, map01110, map01212, map04152, map04910, map04922
FUN_001145	alpha/beta hydrolase fold	-	-	-	-
FUN_001146	KR domain	-	1.1.1.100	ko:K00059	ko00061, ko00333, ko00780, ko01040, ko01100, ko01130, ko01212, map00061, map00333, map00780, map01040, map01100, map01130, map01212
FUN_001147	belongs to the flavoprotein pyridine nucleotide cytochrome reductase family	CBR1	1.6.2.2	ko:K00326	ko00520, map00520
FUN_001150	Anaphase-promoting complex, cyclosome, subunit 3	CDC27	-	ko:K03350	ko04110, ko04111, ko04113, ko04114, ko04120, ko04914, ko05166, map04110, map04111, map04113, map04114, map04120, map04914, map05166
FUN_001151	Protein of unknown function (DUF788)	-	-	-	-
FUN_001152	Eukaryotic mitochondrial regulator protein	-	-	-	-
FUN_001153	RNA recognition motif	WHI3	-	-	-
FUN_001154	Belongs to the class-III pyridoxal-phosphate-dependent aminotransferase family	-	-	-	-
FUN_001155	GAL4-like Zn(II)2Cys6 (or C6 zinc) binuclear cluster DNA-binding domain	-	-	-	-
FUN_001156	Four repeated domains in the Fasciclin I family of proteins, present in many other contexts.	-	-	-	-
FUN_001158	Cytochrome P450 6A1	-	1.14.13.70	ko:K05917	ko00100, ko01100, ko01110, ko01130, map00100, map01100, map01110, map01130
FUN_001159	Glyoxalase/Bleomycin resistance protein/Dioxygenase superfamily	-	-	-	-
FUN_001160	Dehydrogenase	-	-	-	-
FUN_001161	domain protein	-	-	-	-
FUN_001165	Belongs to the 1-acyl-sn-glycerol-3-phosphate acyltransferase family	SLC1	2.3.1.51	ko:K13509	ko00561, ko00564, ko01100, ko01110, ko04072, ko04975, map00561, map00564, map01100, map01110, map04072, map04975
FUN_001166	Cytochrome P450	-	-	ko:K21293	-
FUN_001169	Belongs to the PAL histidase family	-	4.3.1.24	ko:K10775	ko00360, ko00940, ko01100, ko01110, map00360, map00940, map01100, map01110
FUN_001170	HET domain protein	-	-	-	-

FUN_001171	MAP kinase kinase kinase activity	-	2.7.12.2	ko:K04368	ko01521, ko01522, ko04010, ko04012, ko04013, ko04014, ko04015, ko04022, ko04024, ko04062, ko04066, ko04068, ko04071, ko04072, ko04114, ko04140, ko04150, ko04151, ko04210, ko04218, ko04270, ko04320, ko04370, ko04371, ko04380, ko04510, ko04540, ko04550, ko04620, ko04626, ko04650, ko04660, ko04662, ko04664, ko04666, ko04668, ko04720, ko04722, ko04725, ko04726, ko04730, ko04810, ko04910, ko04912, ko04914, ko04915, ko04916, ko04917, ko04919, ko04921, ko04926, ko04934, ko05020, ko05034, ko05161, ko05164, ko05165, ko05167, ko05200, ko05205, ko05206, ko05210, ko05211, ko05212, ko05213, ko05214, ko05215, ko05216, ko05218, ko05219, ko05220, ko05221, ko05223, ko05224, ko05225, ko05226, ko05230, ko05231, map01521, map01522, map04010, map04012, map04013, map04014, map04015, map04022, map04024, map04062, map04066, map04068, map04071, map04072, map04114, map04140, map04150, map04151, map04210, map04218, map04270, map04320, map04370, map04371, map04380, map04510, map04540, map04550, map04620, map04626, map04650, map04660, map04662, map04664, map04666, map04668, map04720, map04722, map04725, map04726, map04730, map04810, map04910, map04912, map04914, map04915, map04916, map04917, map04919, map04921, map04926, map04934, map05020, map05034, map05161, map05164, map05165, map05167, map05200, map05205, map05206, map05210, map05211, map05212, map05213, map05214, map05215, map05216, map05218, map05219, map05220, map05221, map05223, map05224, map05225, map05226, map05230, map05231
FUN_001173	Amino acid permease	-	-	-	-
FUN_001174	NACHT domain	-	-	-	-
FUN_001177	Nuclear cap-binding protein subunit 2	CBC2	-	ko:K12883	ko03013, ko03015, ko03040, map03013, map03015, map03040
FUN_001178	4'-phosphopantetheinyl transferase superfamily	-	-	ko:K06133	ko00770, map00770
FUN_001180	FAD dependent oxidoreductase	-	-	-	-
FUN_001187	Protein of unknown function (DUF952)	-	-	-	-
FUN_001188	Phytanoyl-CoA dioxygenase family protein	-	-	-	-
FUN_001189	Major Facilitator Superfamily	-	-	-	-
FUN_001190	NAD(P)H-binding	-	-	-	-
FUN_001191	reductase	-	1.1.1.100	ko:K00059	ko00061, ko00333, ko00780, ko01040, ko01100, ko01130, ko01212, map00061, map00333, map00780, map01040, map01100, map01130, map01212
FUN_001192	Belongs to the D-isomer specific 2-hydroxyacid dehydrogenase family	-	1.1.1.26	ko:K00015	ko00630, ko01100, ko01110, ko01120, map00630, map01100, map01110, map01120
FUN_001193	Belongs to the major facilitator superfamily. Sugar transporter (TC 2.A.1.1) family	-	-	-	-

FUN_001195	Ras subfamily of RAS small GTPases	RSR1	-	ko:K04353, ko:K07836, ko:K07837	ko04010, ko04014, ko04015, ko04024, ko04062, ko04510, ko04530, ko04611, ko04670, ko04720, ko04722, ko04934, ko04972, ko05211, map04010, map04014, map04015, map04024, map04062, map04510, map04530, map04611, map04670, map04720, map04722, map04934, map04972, map05211
FUN_001196	Protein of unknown function (DUF1620)	-	-	-	-
FUN_001197	Enoyl-(Acyl carrier protein) reductase	-	-	-	-
FUN_001198	Enoyl-(Acyl carrier protein) reductase	-	-	-	-
FUN_001200	Pyridoxal-phosphate dependent enzyme	-	-	-	-
FUN_001201	Heterokaryon incompatibility protein (HET)	-	-	-	-
FUN_001202	Possible catecholamine-binding domain present in a variety of eukaryotic proteins.	-	-	-	-
FUN_001203	Conserved glutamic acid rich protein	-	-	-	-
FUN_001204	Amino acid permease	-	-	-	-
FUN_001205	Isochorismatase family	-	-	-	-
FUN_001206	Core component of nucleosome. Nucleosomes wrap and compact DNA into chromatin, limiting DNA accessibility to the cellular machineries which require DNA as a template. Histones thereby play a central role in transcription regulation, DNA repair, DNA replication and chromosomal stability. DNA accessibility is regulated via a complex set of post-translational modifications of histones, also called histone code, and nucleosome remodeling	HHF1	-	ko:K11254	ko05034, ko05203, ko05322, map05034, map05203, map05322
FUN_001207	Belongs to the GST superfamily	-	2.5.1.18	ko:K00799	ko00480, ko00980, ko00982, ko00983, ko01524, ko05200, ko05204, ko05225, ko05418, map00480, map00980, map00982, map00983, map01524, map05200, map05204, map05225, map05418
FUN_001209	Ankyrin and HET domain-containing protein	-	-	-	-
FUN_001210	Heterokaryon incompatibility protein (HET)	-	-	-	-
FUN_001212	Biotin carboxylase C-terminal domain	-	6.4.1.4	ko:K01968	ko00280, ko01100, map00280, map01100
FUN_001214	Ribosomal protein L24e	-	-	-	-
FUN_001216	54S ribosomal protein L31, mitochondrial	-	-	-	-
FUN_001217	Sedlin, N-terminal conserved region	TRS20	-	ko:K20301	-
FUN_001218	The RING-variant domain is a C4HC3 zinc-finger like motif found in a number of cellular and viral proteins. Some of these proteins have been shown both in vivo and in vitro to have ubiquitin E3 ligase activity.	-	2.3.2.27	ko:K10661	ko04141, map04141
FUN_001221	Belongs to the protein kinase superfamily	-	-	ko:K08286	-
FUN_001222	Domain of unknown function	-	2.7.11.1	ko:K16315	-
FUN_001223	cheY-homologous receiver domain	MgSsk1	-	ko:K11233	ko02020, ko04011, ko04139, map02020, map04011, map04139

FUN_001224	Extension to Ser/Thr-type protein kinases	PKA4	2.7.11.1, 2.7.11.11	ko:K04345, ko:K08282, ko:K19584	ko01522, ko04010, ko04014, ko04020, ko04024, ko04062, ko04113, ko04114, ko04138, ko04140, ko04211, ko04213, ko04261, ko04270, ko04310, ko04340, ko04341, ko04371, ko04530, ko04540, ko04611, ko04713, ko04714, ko04720, ko04723, ko04724, ko04725, ko04726, ko04727, ko04728, ko04740, ko04742, ko04750, ko04910, ko04911, ko04912, ko04913, ko04914, ko04915, ko04916, ko04918, ko04919, ko04921, ko04922, ko04923, ko04924, ko04925, ko04926, ko04927, ko04934, ko04961, ko04962, ko04970, ko04971, ko04976, ko05012, ko05020, ko05030, ko05031, ko05032, ko05110, ko05146, ko05165, ko05166, ko05169, ko05200, ko05203, ko05205, ko05414, map01522, map04010, map04014, map04020, map04024, map04062, map04113, map04114, map04138, map04140, map04211, map04213, map04261, map04270, map04310, map04340, map04341, map04371, map04530, map04540, map04611, map04713, map04714, map04720, map04723, map04724, map04725, map04726, map04727, map04728, map04740, map04742, map04750, map04910, map04911, map04912, map04913, map04914, map04915, map04916, map04918, map04919, map04921, map04922, map04923, map04924, map04925, map04926, map04927, map04934, map04961, map04962, map04970, map04971, map04976, map05012, map05020, map05030, map05031, map05032, map05110, map05146, map05165, map05166, map05169, map05200, map05203, map05205, map05414
FUN_001225	Proton-conducting pore forming subunit of the membrane integral V0 complex of vacuolar ATPase. V-ATPase is responsible for acidifying a variety of intracellular compartments in eukaryotic cells	VMA11	-	ko:K02155	ko00190, ko01100, ko04142, ko04145, ko04721, ko04966, ko05110, ko05120, ko05152, ko05323, map00190, map01100, map04142, map04145, map04721, map04966, map05110, map05120, map05152, map05323
FUN_001226	Domain found in NIK1-like kinases, mouse citron and yeast ROM1, ROM2	ROM2	-	ko:K19842	ko04011, map04011
FUN_001229	spermine spermidine synthase	-	-	ko:K08496	ko04130, map04130
FUN_001230	Coiled-coil domain containing protein (DUF2052)	-	-	-	-
FUN_001231	Major Facilitator Superfamily	-	-	-	-
FUN_001232	Amino acid permease	DIP5	-	ko:K16261	-
FUN_001233	Flavin-binding monooxygenase-like	-	-	-	-
FUN_001234	Flavin-binding monooxygenase-like	-	-	-	-
FUN_001235	Domain found in NIK1-like kinases, mouse citron and yeast ROM1, ROM2	TUS1	-	ko:K19842, ko:K19843	ko04011, map04011
FUN_001236	Fungal specific transcription factor domain	-	-	-	-
FUN_001237	Platelet-activating factor acetylhydrolase, isoform II	-	-	-	-
FUN_001238	Major Facilitator Superfamily	-	-	ko:K02429	-
FUN_001239	Major Facilitator Superfamily	-	-	-	-
FUN_001240	Inositol polyphosphate phosphatase, catalytic domain homologues	-	6.5.1.4	ko:K01974	-
FUN_001241	KR domain	ARDH	1.1.1.250	ko:K17738	ko00040, ko01100, map00040, map01100

FUN_001242	Domain in Tre-2, BUB2p, and Cdc16p. Probable Rab-GAPs.	GYP2	-	ko:K19951	-
FUN_001243	AMP-binding enzyme C-terminal domain	-	-	-	-
FUN_001245	Amino acid permease	-	-	-	-
FUN_001247	Glycosyltransferase family 32 protein	och1	2.4.1.232	ko:K05528, ko:K05534	ko00513, ko01100, map00513, map01100
FUN_001248	Annexin repeats	-	-	ko:K17095	-
FUN_001249	N-Acetylglucosaminyltransferase-IV (GnT-IV) conserved region	-	-	-	-
FUN_001251	Glutathione S-transferase, N-terminal domain	-	2.5.1.18	ko:K00799	ko00480, ko00980, ko00982, ko00983, ko01524, ko05200, ko05204, ko05225, ko05418, map00480, map00980, map00982, map00983, map01524, map05200, map05204, map05225, map05418
FUN_001252	Fungal family of unknown function (DUF1776)	-	-	-	-
FUN_001254	RNA 3'-terminal phosphate cyclase	-	6.5.1.4	ko:K01974	-
FUN_001255	Belongs to the GMC oxidoreductase family	-	-	-	-
FUN_001256	Arsenical pump membrane protein	-	-	-	-
FUN_001257	DUF1212 domain membrane protein	-	-	-	-
FUN_001258	Major Facilitator Superfamily	-	-	-	-
FUN_001259	Fungal specific transcription factor domain	-	-	-	-
FUN_001261	Basic region leucine zipper	-	-	ko:K04450, ko:K09047	ko04010, ko04013, ko04022, ko04024, ko04151, ko04152, ko04211, ko04261, ko04624, ko04668, ko04714, ko04725, ko04728, ko04911, ko04915, ko04918, ko04922, ko04925, ko04926, ko04927, ko04931, ko04934, ko04962, ko05016, ko05030, ko05031, ko05034, ko05161, ko05164, ko05165, ko05166, ko05169, ko05203, ko05215, map04010, map04013, map04022, map04024, map04151, map04152, map04211, map04261, map04624, map04668, map04714, map04725, map04728, map04911, map04915, map04918, map04922, map04925, map04926, map04927, map04931, map04934, map04962, map05016, map05030, map05031, map05034, map05161, map05164, map05165, map05166, map05169, map05203, map05215
FUN_001262	Indole-diterpene biosynthesis protein	-	-	-	-
FUN_001263	Fungal specific transcription factor domain	-	-	-	-
FUN_001264	Glycosyl hydrolase family 61	-	-	-	-
FUN_001265	Amino acid permease	-	-	-	-
FUN_001268	Uracil phosphoribosyltransferase	-	-	-	-
FUN_001269	Major Facilitator Superfamily	-	-	-	-
FUN_001270	WD domain, G-beta repeat	-	-	ko:K13137	ko03013, map03013
FUN_001273	Alpha/beta hydrolase family	-	3.3.2.9	ko:K01253	ko00980, ko04976, ko05204, map00980, map04976, map05204
FUN_001274	non-ribosomal peptide synthetase	-	-	-	-
FUN_001278	Glycoside hydrolase family 17 protein	btgC	3.2.1.58	ko:K01210	ko00500, map00500
FUN_001279	serine-type endopeptidase activity	-	3.4.21.48	ko:K01336	ko04138, map04138
FUN_001281	Belongs to the iron ascorbate-dependent oxidoreductase family	-	-	-	-
FUN_001282	Eukaryotic elongation factor 5A hypusine, DNA-binding OB fold	-	-	-	-

FUN_001283	Glyco_18	-	3.2.1.14	ko:K01183, ko:K03859	ko00520, ko00563, ko01100, map00520, map00563, map01100
FUN_001284	Protein of unknown function (DUF1682)	-	-	-	-
FUN_001287	Large-conductance mechanosensitive channel, MscL	-	-	ko:K03282	-
FUN_001293	impB/mucB/samB family	RAD30	2.7.7.7	ko:K03509	ko01524, ko03460, map01524, map03460
FUN_001295	Carboxypeptidase activation peptide	MCPA	-	-	-
FUN_001296	Fungal specific transcription factor domain	-	-	-	-
FUN_001298	PLC-like phosphodiesterase	-	-	-	-
FUN_001301	AAA domain	-	3.6.4.13	ko:K11701	-
FUN_001302	WD domain, G-beta repeat	SPT8	-	ko:K11360	-
FUN_001303	Phosphorylates Ins(1, 3, 4, 5, 6)P5 at position 2 to form Ins(1, 2, 3, 4, 5, 6)P6 (InsP6 or phytate)	IPK1	2.7.1.158	ko:K19786	ko00562, ko01100, ko04070, map00562, map01100, map04070
FUN_001304	Component of the ERMES MDM complex, which serves as a molecular tether to connect the endoplasmic reticulum and mitochondria. Components of this complex are involved in the control of mitochondrial shape and protein biogenesis and may function in phospholipid exchange. mdm34 is required for the interaction of the ER-resident membrane protein mmm1 and the outer mitochondrial membrane-resident beta-barrel	MDM34	-	ko:K17775	ko04139, map04139
FUN_001307	Belongs to the TRAFAC class myosin-kinesin ATPase superfamily. Myosin family	MYO1	-	ko:K10352	ko04530, map04530
FUN_001309	Zinc finger C-x8-C-x5-C-x3-H type (and similar)	-	-	-	-
FUN_001310	Enoyl-(Acyl carrier protein) reductase	-	-	-	-
FUN_001311	Transaldolase is important for the balance of metabolites in the pentose-phosphate pathway	TAL1	2.2.1.2	ko:K00616	ko00030, ko01100, ko01110, ko01120, ko01130, ko01200, ko01230, map00030, map01100, map01110, map01120, map01130, map01200, map01230
FUN_001312	Destroys radicals which are normally produced within the cells and which are toxic to biological systems	SOD1	1.15.1.1	ko:K04565	ko04146, ko04213, ko05014, ko05016, ko05020, map04146, map04213, map05014, map05016, map05020
FUN_001313	Tudor domain	-	-	ko:K15979	ko05169, ko05203, map05169, map05203
FUN_001316	Belongs to the class-I aminoacyl-tRNA synthetase family	SYR1	6.1.1.19	ko:K01887	ko00970, map00970
FUN_001317	Putative lipopolysaccharide-modifying enzyme.	-	-	-	-
FUN_001319	Domain of unknown function (DUF4360)	-	-	-	-
FUN_001320	ATPase family associated with various cellular activities (AAA)	-	-	-	-
FUN_001322	NAD(P) transhydrogenase beta subunit	-	1.6.1.2	ko:K00323	ko00760, ko01100, map00760, map01100
FUN_001324	PHD-finger	CTI6	-	-	-
FUN_001325	Occurs in almost all aerobically respiring organisms and serves to protect cells from the toxic effects of hydrogen peroxide	-	1.11.1.6	ko:K03781	ko00380, ko00630, ko01110, ko01130, ko01200, ko04011, ko04016, ko04068, ko04146, ko04211, ko04212, ko04213, ko05014, map00380, map00630, map01110, map01130, map01200, map04011, map04016, map04068, map04146, map04211, map04212, map04213, map05014
FUN_001331	helicase superfamily c-terminal domain	DBP9	3.6.4.13	ko:K14810	-
FUN_001332	protein localization to T-tubule	-	-	-	-
FUN_001342	Pectinolytic enzyme consist of four classes of enzymes pectin lyase, polygalacturonase, pectin methylesterase and rhamnogalacturonase. Among pectinolytic enzymes, pectin lyase is the most important in depolymerization of pectin, since it cleaves internal glycosidic bonds of highly methylated pectins. Favors pectate, the anion, over pectin, the methyl ester	plyE	4.2.2.2	ko:K01728	ko00040, ko02024, map00040, map02024

FUN_001343	Belongs to the cytochrome P450 family	-	-	-	-
FUN_001352	Belongs to the cytochrome P450 family	-	-	-	-
FUN_001353	FAD binding domain	-	-	-	-
FUN_001354	HET domain-containing protein	-	-	-	-
FUN_001355	Belongs to the peptidase S10 family	-	3.4.16.6	ko:K01288	-
FUN_001356	Antibiotic biosynthesis monooxygenase	-	-	-	-
FUN_001357	Pfam:Chitin_bind_3	-	-	-	-
FUN_001359	ser Thr protein phosphatase family protein	-	-	-	-
FUN_001362	Ankyrin repeat	-	-	-	-
FUN_001366	Domain of unknown function (DUF4149)	-	-	-	-
FUN_001367	Carbohydrate esterase family 12 protein	-	-	-	-
FUN_001368	Podospora anserina S mat genomic DNA chromosome	-	-	-	-
FUN_001369	integral membrane protein	-	-	-	-
FUN_001371	Acetyltransferase (GNAT) domain	-	-	-	-
FUN_001372	Nitronate monooxygenase	-	-	-	-
FUN_001373	DJ-1/PfpI family	-	-	-	-
FUN_001374	Belongs to the metallo-dependent hydrolases superfamily. Peptidase M19 family	-	3.4.13.19	ko:K01273	-
FUN_001375	alcohol dehydrogenase	-	1.1.99.1	ko:K00108	ko00260, ko01100, map00260, map01100
FUN_001376	Belongs to the oxygen-dependent FAD-linked oxidoreductase family	-	-	-	-
FUN_001378	Multicopper oxidase	-	-	-	-
FUN_001379	dipeptidyl-peptidase activity	-	-	ko:K06978	-
FUN_001380	X-Pro dipeptidyl-peptidase C-terminal non-catalytic domain	-	-	ko:K06978	-
FUN_001381	Retinal pigment epithelial membrane protein	-	-	ko:K11159	-
FUN_001382	Aldehyde dehydrogenase family	-	-	-	-
FUN_001383	Acylpyruvase FAHD1	-	3.7.1.5	ko:K01557	ko00350, ko01100, ko01120, map00350, map01100, map01120
FUN_001384	Cupin domain	-	1.13.11.4	ko:K00450	ko00350, ko01100, ko01120, map00350, map01100, map01120
FUN_001385	Fungal specific transcription factor domain	-	-	-	-
FUN_001386	FAD binding domain	-	1.14.13.1	ko:K00480	ko00621, ko00624, ko00626, ko01100, ko01120, ko01220, map00621, map00624, map00626, map01100, map01120, map01220
FUN_001387	Protein of unknown function (DUF3237)	-	-	-	-
FUN_001388	Major Facilitator Superfamily	-	-	-	-
FUN_001390	Complex1_LYR-like	-	-	-	-
FUN_001392	Sugar (and other) transporter	-	-	ko:K08176	-
FUN_001395	Amino acid permease	-	-	-	-
FUN_001396	Isocitrate/isopropylmalate dehydrogenase	-	-	-	-
FUN_001397	uridine kinase activity	-	2.7.1.31	ko:K15918	ko00260, ko00561, ko00630, ko01100, ko01110, ko01130, ko01200, map00260, map00561, map00630, map01100, map01110, map01130, map01200
FUN_001399	NAD(P)H-binding	-	-	-	-
FUN_001400	Glycoside hydrolase family 35 protein	-	-	-	-
FUN_001401	DEAD-box RNA helicase-like protein required for pre-18S rRNA processing, specifically at sites A0, A1, and A2	UTP25	-	ko:K14774	-

FUN_001402	component of the eukaryotic translation initiation factor 3 (eIF-3) complex, which is involved in protein synthesis of a specialized repertoire of mRNAs and, together with other initiation factors, stimulates binding of mRNA and methionyl-tRNAi to the 40S ribosome. The eIF-3 complex specifically targets and initiates translation of a subset of mRNAs involved in cell proliferation	-	-	ko:K03249	ko03013, map03013
FUN_001403	Transcription initiation factor IID, 18kD subunit	-	-	ko:K03127	ko03022, ko05168, map03022, map05168
FUN_001405	Protoglobin	-	-	-	-
FUN_001406	Acyl-CoA dehydrogenase, middle domain	-	-	-	-
FUN_001410	MutL C terminal dimerisation domain	MLH3	-	ko:K08739	ko03430, map03430
FUN_001411	Heterokaryon incompatibility protein (HET)	-	-	-	-
FUN_001415	Carbohydrate esterase family 4 protein	-	-	-	-
FUN_001419	YCII-related domain	-	-	ko:K09780	-
FUN_001420	Provides the precursors necessary for DNA synthesis. Catalyzes the biosynthesis of deoxyribonucleotides from the corresponding ribonucleotides	RNR1	1.17.4.1	ko:K10807	ko00230, ko00240, ko00480, ko00983, ko01100, map00230, map00240, map00480, map00983, map01100
FUN_001421	Belongs to the bacterial ribosomal protein bL17 family	mrpl8	-	ko:K02879	ko03010, map03010
FUN_001422	Gelsolin repeat	SEC23	-	ko:K14006	ko04141, map04141
FUN_001424	ER associated DnaJ chaperone	HLJ1	-	ko:K09518	ko04141, map04141
FUN_001425	Ubiquitin elongating factor core	UFD2	2.3.2.27	ko:K10597	ko04120, ko04141, map04120, map04141
FUN_001426	Belongs to the small GTPase superfamily. Rho family	RHO1	-	ko:K04513, ko:K07975	ko04011, ko04014, ko04015, ko04022, ko04024, ko04062, ko04071, ko04072, ko04144, ko04150, ko04270, ko04310, ko04350, ko04360, ko04510, ko04520, ko04530, ko04611, ko04621, ko04660, ko04670, ko04722, ko04810, ko04921, ko04972, ko05100, ko05130, ko05133, ko05152, ko05200, ko05203, ko05205, ko05206, ko05210, ko05418, map04011, map04014, map04015, map04022, map04024, map04062, map04071, map04072, map04144, map04150, map04270, map04310, map04350, map04360, map04510, map04520, map04530, map04611, map04621, map04660, map04670, map04722, map04810, map04921, map04972, map05100, map05130, map05133, map05152, map05200, map05203, map05205, map05206, map05210, map05418
FUN_001427	Belongs to the peptidase A1 family	CTSD	3.4.23.25, 3.4.23.5	ko:K01379, ko:K01381	ko04071, ko04138, ko04140, ko04142, ko04210, ko04915, ko05152, map04071, map04138, map04140, map04142, map04210, map04915, map05152
FUN_001428	Fungal domain of unknown function (DUF1712)	-	-	-	-
FUN_001429	Formyl transferase	FMT1	2.1.2.9	ko:K00604	ko00670, ko00970, map00670, map00970
FUN_001435	Belongs to the diphosphomevalonate decarboxylase family	MVD1	4.1.1.33	ko:K01597	ko00900, ko01100, ko01110, ko01130, map00900, map01100, map01110, map01130
FUN_001436	Links covalently the heme group to the apoprotein of cytochrome c	CYT2	4.4.1.17	ko:K01764	ko00860, map00860
FUN_001437	Belongs to the syntaxin family	PEP12	-	-	-
FUN_001438	Nucleoporin protein Ndc1-Nup	-	-	ko:K14315	ko03013, map03013
FUN_001442	Protein tyrosine kinase	CEX1	-	ko:K08876	-
FUN_001444	Ion transport protein	-	-	-	-
FUN_001445	RNA recognition motif. (a.k.a. RRM, RBD, or RNP domain)	-	-	ko:K14396	ko03015, ko05164, map03015, map05164

FUN_001446	Belongs to the ubiquitin-conjugating enzyme family	hus5	-	ko:K10577	ko03013, ko04064, ko04120, ko05206, map03013, map04064, map04120, map05206
FUN_001447	Pex19 protein family	PEX19	-	ko:K13337	ko04146, map04146
FUN_001449	Carbohydrate-binding module family 1 protein	-	1.14.99.54	ko:K19356	-
FUN_001452	Common central domain of tyrosinase	-	1.14.18.1	ko:K00505	ko00350, ko00950, ko00965, ko01100, ko01110, ko04916, map00350, map00950, map00965, map01100, map01110, map04916
FUN_001453	Extracellular dioxygenase	-	-	-	-
FUN_001455	Prolyl 4-hydroxylase alpha subunit homologues.	-	1.14.11.2	ko:K00472	ko00330, ko01100, map00330, map01100
FUN_001456	intramolecular oxidoreductase activity, transposing S-S bonds	-	5.3.4.1	ko:K09580	ko04141, map04141
FUN_001458	Involved in nucleolar processing of pre-18S ribosomal RNA	MPP10	-	ko:K14559	ko03008, map03008
FUN_001459	GPI anchored protein	-	-	-	-
FUN_001460	GMC oxidoreductase	-	-	-	-
FUN_001468	Domain of unknown function (DUF1996)	-	-	-	-
FUN_001469	The amino acid sequence converted into a structural homology model presents the general folding and conserved residues forming the active site of a typical dye-	DyP1	-	-	-
FUN_001470	Glycosyltransferase family 32 protein	-	-	-	-
FUN_001471	Acyltransferase family	-	-	-	-
FUN_001472	Glycosyltransferase family 8 protein	-	-	-	-
FUN_001474	short chain dehydrogenase	-	-	-	-
FUN_001476	Belongs to the oxygen-dependent FAD-linked oxidoreductase family	-	-	-	-
FUN_001478	WD repeat-containing protein	TUP1	-	ko:K06666	ko04011, ko04111, map04011, map04111
FUN_001479	Belongs to the TPP enzyme family	-	4.1.1.1	ko:K01568	ko00010, ko01100, ko01110, ko01130, map00010, map01100, map01110, map01130
FUN_001480	aflatoxin efflux	-	-	-	-
FUN_001481	cfem domain-containing protein	-	-	-	-
FUN_001482	Belongs to the cytochrome P450 family	-	-	-	-
FUN_001483	NAD dependent epimerase dehydratase	-	-	-	-
FUN_001489	Methyltransferase domain	-	-	-	-
FUN_001490	Required for the post-translational delivery of tail- anchored (TA) proteins to the endoplasmic reticulum. Acts as a membrane receptor for soluble get3, which recognizes and selectively binds the transmembrane domain of TA proteins in the	GET1	-	ko:K22384	-
FUN_001493	AMP-binding enzyme C-terminal domain	-	-	ko:K18660	ko00280, map00280
FUN_001495	Belongs to the acyl-CoA oxidase family	-	1.3.3.6	ko:K00232	ko00071, ko00592, ko01040, ko01100, ko01110, ko01212, ko03320, ko04024, ko04146, map00071, map00592, map01040, map01100, map01110, map01212, map03320, map04024, map04146
FUN_001496	Male sterility protein	-	-	-	-
FUN_001497	Belongs to the cytochrome P450 family	-	1.14.14.1, 1.6.2.4	ko:K14338	ko00071, ko00380, ko00627, ko01120, map00071, map00380, map00627, map01120
FUN_001498	transcription factor activity, RNA polymerase II proximal promoter sequence-specific DNA binding	-	-	-	-
FUN_001500	Cytidylyltransferase-like	MUQ1	2.7.7.14	ko:K00967	ko00440, ko00564, ko01100, map00440, map00564, map01100
FUN_001502	FR47-like protein	ARD1	2.3.1.255	ko:K20791	-
FUN_001504	glucose-methanol-choline oxidoreductase	-	-	-	-

FUN_001505	Glucose-methanol-choline oxidoreductase	-	-	-	-
FUN_001506	Dienelactone hydrolase family	-	-	-	-
FUN_001507	Fungal specific transcription factor domain	-	-	-	-
FUN_001508	Enoyl-CoA hydratase/isomerase	-	-	-	-
FUN_001509	Cytochrome domain of cellobiose dehydrogenase	-	-	-	-
FUN_001510	Pyridine nucleotide-disulphide oxidoreductase	-	-	-	-
FUN_001512	Heterokaryon incompatibility protein (HET)	-	-	-	-
FUN_001513	Belongs to the class-IV pyridoxal-phosphate-dependent aminotransferase family	-	2.6.1.42	ko:K00826	ko00270, ko00280, ko00290, ko00770, ko01100, ko01110, ko01130, ko01210, ko01230, map00270, map00280, map00290, map00770, map01100, map01110, map01130, map01210, map01230
FUN_001514	tail-anchored membrane protein insertion into ER membrane	-	-	ko:K22384	-
FUN_001516	saf domain-containing protein	-	-	-	-
FUN_001517	cfem domain-containing protein	-	-	-	-
FUN_001518	Belongs to the oxygen-dependent FAD-linked oxidoreductase family	-	-	-	-
FUN_001519	CorA-like Mg2+ transporter protein	-	-	-	-
FUN_001521	AMP-binding enzyme C-terminal domain	-	6.2.1.8	ko:K22133	ko00630, ko01100, map00630, map01100
FUN_001522	glycine rich nucleic binding domain	-	-	ko:K12840	ko03040, map03040
FUN_001523	Mur ligase middle domain	FOL3	6.3.2.12	ko:K20457	ko00790, ko01100, map00790, map01100
FUN_001525	)-reductase	-	-	-	-
FUN_001526	Type I 3-dehydroquinase	-	4.2.1.10	ko:K03785	ko00400, ko01100, ko01110, ko01130, ko01230, map00400, map01100, map01110, map01130, map01230
FUN_001527	Fungal specific transcription factor domain	-	4.2.1.10	ko:K03785	ko00400, ko01100, ko01110, ko01130, ko01230, map00400, map01100, map01110, map01130, map01230
FUN_001528	Belongs to the class-I pyridine nucleotide-disulfide oxidoreductase family	GLR1	1.8.1.7	ko:K00383	ko00480, ko04918, map00480, map04918
FUN_001529	Belongs to the mitochondrial carrier (TC 2.A.29) family	-	-	-	-
FUN_001530	acyl-CoA thioester hydrolase	-	-	ko:K17361	-
FUN_001532	Bacterial signalling protein N terminal repeat	-	-	-	-
FUN_001533	Essential component of the cytosolic iron-sulfur (Fe S) protein assembly machinery. Required for the maturation of extramitochondrial Fe S proteins	CIA1	-	-	-
FUN_001534	Cytidylyltransferase-like	PCT1	2.7.7.15	ko:K00968	ko00440, ko00564, ko01100, ko05231, map00440, map00564, map01100, map05231
FUN_001535	T-complex protein 11	SOK1	-	-	-
FUN_001537	Ring finger domain	-	-	-	-
FUN_001538	Belongs to the lyase 1 family. Adenylosuccinate lyase subfamily	ADE13	4.3.2.2	ko:K01756	ko00230, ko00250, ko01100, ko01110, ko01130, map00230, map00250, map01100, map01110, map01130
FUN_001539	SUR7/PalI family	-	-	-	-
FUN_001541	Belongs to the mitochondrial carrier (TC 2.A.29) family	-	-	ko:K15115	-
FUN_001542	Component of the ubiquinol-cytochrome c reductase complex (complex III or cytochrome b-c1 complex), which is part of the mitochondrial respiratory chain	QCR7	-	ko:K00417	ko00190, ko01100, ko04260, ko04714, ko04932, ko05010, ko05012, ko05016, map00190, map01100, map04260, map04714, map04932, map05010, map05012, map05016
FUN_001543	endonuclease III	MAG1	3.2.2.21	ko:K01247	ko03410, map03410
FUN_001544	GPI-anchored cell wall organization protein	ecm33	-	-	-

FUN_001545	Glycosyltransferase family 62 protein	-	-	-	-
FUN_001546	Belongs to the major facilitator superfamily. Sugar transporter (TC 2.A.1.1) family	HXT5	-	ko:K08139	ko04113, map04113
FUN_001547	Belongs to the thiolase family	-	2.3.1.176	ko:K08764	ko00120, ko01100, ko03320, ko04146, map00120, map01100, map03320, map04146
FUN_001548	Major Facilitator Superfamily	-	-	-	-
FUN_001549	Gpi anchored serine-threonine rich protein	-	-	-	-
FUN_001550	E3 ubiquitin-protein ligase	hulA	2.3.2.26	ko:K10591	ko04011, ko04120, ko04144, ko04530, ko05169, map04011, map04120, map04144, map04530, map05169
FUN_001551	ABC1 family	-	-	ko:K08869	-
FUN_001552	Glycerophosphoryl diester phosphodiesterase family	PHO81	-	ko:K06653	ko04111, map04111
FUN_001553	CRM1 C terminal	CRM1	-	ko:K14290	ko03008, ko03013, ko04013, ko05164, ko05166, ko05169, map03008, map03013, map04013, map05164, map05166, map05169
FUN_001554	Protein phosphatase inhibitor	YPI1	-	ko:K17553	-
FUN_001555	DASH complex subunit Dad2	DAD2	-	ko:K11567	-
FUN_001556	Shugoshin C terminus	-	-	-	-
FUN_001559	Zinc finger, C2H2 type	-	-	-	-
FUN_001560	Tctex-1 family	-	-	-	-
FUN_001561	Basic region leucine zipper	-	-	-	-
FUN_001562	Kinetochore protein CHL4 like	CHL4	-	ko:K11551	-
FUN_001564	Zinc-finger domain of monoamine-oxidase A repressor R1	-	-	-	-
FUN_001565	Catalyzes the prenylation of para-hydroxybenzoate (PHB) with an all-trans polyprenyl group. Mediates the second step in the final reaction sequence of coenzyme Q (CoQ) biosynthesis, which is the condensation of the polyisoprenoid side chain with PHB, generating the first membrane-bound Q intermediate	COQ2	2.5.1.39	ko:K06125	ko00130, ko01100, ko01110, map00130, map01100, map01110
FUN_001566	TatD related DNase	pi038	-	ko:K03424	-
FUN_001567	AAA-ATPase Vps4-associated protein 1	-	-	-	-
FUN_001568	zinc finger	-	-	-	-
FUN_001569	zinc finger	-	-	-	-
FUN_001570	Dual specificity phosphatase, catalytic domain	YVH1	3.1.3.16, 3.1.3.48	ko:K14819	-
FUN_001571	Aconitase family (aconitate hydratase)	ACO2	-	ko:K17450	ko00300, ko01100, ko01120, ko01130, ko01210, ko01230, map00300, map01100, map01120, map01130, map01210, map01230
FUN_001572	Aconitase family (aconitate hydratase)	ACO2	-	ko:K17450	ko00300, ko01100, ko01120, ko01130, ko01210, ko01230, map00300, map01100, map01120, map01130, map01210, map01230
FUN_001573	Small subunit of acetolactate synthase	ILV6	2.2.1.6	ko:K01653	ko00290, ko00650, ko00660, ko00770, ko01100, ko01110, ko01130, ko01210, ko01230, map00290, map00650, map00660, map00770, map01100, map01110, map01130, map01210, map01230
FUN_001574	VTC domain	-	-	-	-
FUN_001575	Belongs to the FAD-dependent glycerol-3-phosphate dehydrogenase family	GUT2	1.1.5.3	ko:K00111	ko00564, ko01110, map00564, map01110
FUN_001576	Vitamin B6 photo-protection and homoeostasis	-	-	-	-
FUN_001578	RAM signalling pathway protein	SOG2	-	-	-
FUN_001579	pre-rrna processing protein	-	-	-	-

FUN_001580	Belongs to the ATCase OTCase family	ARG3	2.1.3.3	ko:K00611	ko00220, ko01100, ko01110, ko01130, ko01230, map00220, map01100, map01110, map01130, map01230
FUN_001581	Domain of unknown function (DUF1857)	-	-	-	-
FUN_001582	Nuclear cap-binding protein subunit 3	-	-	-	-
FUN_001583	Aldehyde dehydrogenase family	-	1.2.1.24	ko:K00139	ko00250, ko00650, ko01100, ko01120, map00250, map00650, map01100, map01120
FUN_001584	Enoyl-(Acyl carrier protein) reductase	-	-	-	-
FUN_001585	KR domain	-	-	-	-
FUN_001586	glucose import	-	-	-	-
FUN_001587	Glycoside hydrolase family 63 protein	CWH41	3.2.1.106	ko:K01228	ko00510, ko01100, ko04141, map00510, map01100, map04141
FUN_001588	phosphoribulokinase uridine kinase	-	-	-	-
FUN_001589	3-oxo-5-alpha-steroid 4-dehydrogenase	-	1.3.1.22, 3.6.4.12	ko:K10901, ko:K12343	ko00140, ko03440, ko03460, map00140, map03440, map03460
FUN_001590	multi-organism process	-	-	-	-
FUN_001593	cell wall protein	-	-	-	-
FUN_001594	Repeated motif present between transmembrane helices in cystinosin, yeast ERS1p, mannose-P-dolichol utilization defect 1, and other hypothetical proteins.	-	-	-	-
FUN_001595	Dystroglycan-type cadherin-like domains.	AXL2	-	ko:K18637	-
FUN_001596	Domain present in ubiquitin-regulatory proteins	-	-	-	-
FUN_001597	MSP (Major sperm protein) domain	SCS2	-	-	-
FUN_001598	Aluminium induced protein	ASN1	6.3.5.4	ko:K01953	ko00250, ko01100, ko01110, map00250, map01100, map01110
FUN_001599	SANT/Myb-like domain of DAMP1	SWC4	-	ko:K11324	-
FUN_001600	Belongs to the protein kinase superfamily	YCK2	2.7.11.1	ko:K02218	ko04011, ko04392, map04011, map04392
FUN_001601	Non-repetitive/WGA-negative nucleoporin C-terminal	-	-	ko:K14300	ko03013, map03013
FUN_001602	STF2-like protein	-	-	-	-
FUN_001603	Component of the cytosolic iron-sulfur (Fe-S) protein assembly (CIA) machinery. Required for the maturation of extramitochondrial Fe-S proteins. Part of an electron transfer chain functioning in an early step of cytosolic Fe-S biogenesis. Electrons are transferred to the Fe-S cluster from NADPH via the FAD- and FMN-containing protein TAH18. Has anti-apoptotic effects in the cell. Involved in negative control of H(2)O(2)-induced cell death	DRE2	-	-	-
FUN_001604	Belongs to the major facilitator superfamily. Sugar transporter (TC 2.A.1.1) family	-	-	-	-
FUN_001605	Putative cyclase	-	-	-	-
FUN_001606	Belongs to the short-chain dehydrogenases reductases (SDR) family	-	-	-	-
FUN_001607	emp24/gp25L/p24 family/GOLD	ERP1	-	ko:K20346	-
FUN_001609	Belongs to the glutaredoxin family. Monothiol subfamily	GRX5	-	ko:K07390	-
FUN_001611	Belongs to the peroxidase family	CCP1	1.11.1.5	ko:K00428	-
FUN_001613	Major Facilitator Superfamily	TNA1	-	-	-
FUN_001614	Disordered region of unknown function (DUF5315)	-	-	-	-
FUN_001615	Belongs to the universal ribosomal protein uS12 family	rps23	-	ko:K02973	ko03010, map03010
FUN_001618	Involved in protein N-glycosylation. Essential for the second step of the dolichol-linked oligosaccharide pathway. Anchors the catalytic subunit ALG13 to the ER	ALG14	2.4.1.141	ko:K07441	ko00510, ko00513, ko01100, map00510, map00513, map01100
FUN_001619	Belongs to the eIF-2B alpha beta delta subunits family	GCD7	-	ko:K03754	ko03013, map03013

FUN_001621	Polysaccharide deacetylase	-	-	-	-
FUN_001622	allergen asp	-	-	-	-
FUN_001623	Med18 protein	srb5	-	ko:K15136	-
FUN_001624	Polynucleotide kinase 3 phosphatase	pnk1	2.7.1.78, 3.1.3.32	ko:K08073, ko:K08075	-
FUN_001625	Only prolin and serin are matching in the corresponding protein	-	-	-	-
FUN_001626	Protein of unknown function (DUF3984)	-	-	-	-
FUN_001627	RTA1 domain protein	-	-	-	-
FUN_001628	Fungal specific transcription factor domain	-	-	-	-
FUN_001629	Glycoside hydrolase family 92 protein	-	-	-	-
FUN_001630	Protein of unknown function (DUF1761)	-	-	-	-
FUN_001631	RNA recognition motif. (a.k.a. RRM, RBD, or RNP domain)	NOP15	-	ko:K14838	-
FUN_001633	Serine-threonine rich protein	-	-	-	-
FUN_001635	Extracellular dioxygenase	-	-	-	-
FUN_001636	Rhodanese Homology Domain	-	-	-	-
FUN_001637	Component of the MICOS complex, a large protein complex of the mitochondrial inner membrane that plays crucial roles in the maintenance of crista junctions, inner membrane architecture, and formation of contact sites to the outer membrane	-	-	ko:K17787	-
FUN_001639	Flavoprotein	-	2.3.1.47, 4.1.1.36	ko:K00652, ko:K01598	ko00770, ko00780, ko01100, map00770, map00780, map01100
FUN_001640	Aldo/keto reductase family	-	-	-	-
FUN_001641	Peptidase family S41	-	-	-	-
FUN_001642	Kinase-like	chk1	2.7.11.1	ko:K02216	ko04110, ko04111, ko04113, ko04115, ko04218, ko05166, ko05203, map04110, map04111, map04113, map04115, map04218, map05166, map05203
FUN_001643	Regulator of G protein signalling domain	SST2	-	ko:K19838	ko04011, map04011
FUN_001646	60Kd inner membrane protein	-	-	ko:K17797	ko04714, map04714
FUN_001647	Glycosyltransferase family 4 protein	ALG11	2.4.1.131	ko:K03844	ko00510, ko00513, ko01100, map00510, map00513, map01100
FUN_001648	GPI anchored cell wall protein	-	-	-	-
FUN_001649	Domain always found downstream of DENN domain, found in a variety of signalling proteins	-	-	-	-
FUN_001650	Protein of unknown function (DUF974)	-	-	ko:K20310	-
FUN_001651	Protein of unknown function (DUF2408)	-	-	-	-
FUN_001652	Belongs to the eukaryotic ribosomal protein P1 P2 family	-	-	ko:K02942	ko03010, map03010
FUN_001653	Belongs to the ALAD family	HEM2	4.2.1.24	ko:K01698	ko00860, ko01100, ko01110, ko01120, map00860, map01100, map01110, map01120
FUN_001658	Protein of unknown function (DUF1279)	NAT2	-	ko:K20815	-
FUN_001659	Peptide N-acetyl-beta-D-glucosaminyl asparaginase amidase A	-	-	-	-
FUN_001660	EamA-like transporter family	-	-	-	-
FUN_001662	T-complex protein 11	-	-	-	-
FUN_001664	HET domain-containing protein	-	-	-	-
FUN_001665	Glycosyltransferase family 22 protein	GPI10	-	ko:K05286	ko00563, ko01100, map00563, map01100

FUN_001666	Protein tyrosine kinase	-	2.7.11.1, 2.7.11.17	ko:K07359, ko:K21157, ko:K21158	ko04138, ko04140, ko04152, ko04211, ko04920, ko04921, ko05034, map04138, map04140, map04152, map04211, map04920, map04921, map05034
FUN_001667	Cytochrome oxidase complex assembly protein 1	COA1	-	ko:K18173	ko04714, map04714
FUN_001669	Amino acid permease	UGA4	-	ko:K09885	-
FUN_001670	Sugar (and other) transporter	-	-	-	-
FUN_001671	Binds specifically to cytosolic chaperonin (c-CPN) and transfers target proteins to it. Binds to nascent polypeptide chain and promotes folding in an environment in which there are many competing pathways for nonnative proteins	-	-	ko:K09550	-
FUN_001672	Vacuolar sorting protein 39 domain 2	-	-	ko:K20177	ko04138, map04138
FUN_001673	CASP C terminal	-	-	ko:K09313	-
FUN_001674	Protein of unknown function (DUF3455)	-	-	-	-
FUN_001676	Conidiation-specific protein	-	-	-	-
FUN_001677	Catalyzes the oxidation of 3-carboxy-2-hydroxy-4- methylpentanoate (3-isopropylmalate) to 3-carboxy-4-methyl-2- oxopentanoate. The product decarboxylates to 4-methyl-2 oxopentanoate	LEU2	1.1.1.85	ko:K00052	ko00290, ko00660, ko01100, ko01110, ko01210, ko01230, map00290, map00660, map01100, map01110, map01210, map01230
FUN_001681	CPSF A subunit region	RSE1	-	ko:K12830	ko03040, map03040
FUN_001682	Regulatory subunit of the poly(A)-nuclease (PAN) deadenylation complex, one of two cytoplasmic mRNA deadenylases involved in mRNA turnover. PAN specifically shortens poly(A) tails of RNA and the activity is stimulated by poly(A)-binding protein PAB1. PAN deadenylation is followed by rapid degradation of the shortened mRNA tails by the CCR4-NOT complex. Deadenylated mRNAs are then degraded by two alternative mechanisms, namely exosome- mediated 3'-5' exonucleolytic degradation, or deadenlyation- dependent mRNA decaping and subsequent 5'-3' exonucleolytic degradation by XRN1. May also be involved in post-transcriptional maturation of mRNA poly(A) tails. PAN3 acts as a positive regulator for PAN activity, recruiting the	PAN3	-	ko:K12572	ko03018, map03018
FUN_001683	Adenosine kinase	ADO1	2.7.1.20	ko:K00856	ko00230, ko01100, map00230, map01100
FUN_001684	zinc finger	-	-	-	-
FUN_001685	Telomerase activating protein Est1	-	-	-	-
FUN_001686	UAS	ucp10	-	ko:K18726	-
FUN_001689	NACHT domain	-	-	-	-
FUN_001690	Belongs to the histidine acid phosphatase family	phyA	3.1.3.8	ko:K01083	ko00562, map00562
FUN_001691	Amino acid permease	-	-	-	-
FUN_001693	Glutathione S-transferase, C-terminal domain	URE2	2.5.1.18	ko:K00799	ko00480, ko00980, ko00982, ko00983, ko01524, ko05200, ko05204, ko05225, ko05418, map00480, map00980, map00982, map00983, map01524, map05200, map05204, map05225, map05418
FUN_001694	Belongs to the cytochrome P450 family	-	1.14.13.78	ko:K21292	ko00904, ko01100, map00904, map01100
FUN_001696	cytochrome P450	-	1.14.13.78	ko:K21292	ko00904, ko01100, map00904, map01100
FUN_001697	UMTA methyltransferase family protein	-	-	-	-
FUN_001698	Major Facilitator Superfamily	-	-	-	-
FUN_001700	DnaJ molecular chaperone homology domain	dnaJ2	-	ko:K03686, ko:K05516	-
FUN_001701	Chaperone protein dnaJ 11	-	-	-	-

FUN_001703	CFEM domain	-	-	-	-
FUN_001704	Belongs to the cytochrome P450 family	-	-	-	-
FUN_001705	Phytanoyl-CoA dioxygenase (PhyH)	-	-	-	-
FUN_001707	Fungal specific transcription factor domain	-	-	-	-
FUN_001708	FAD dependent oxidoreductase	-	-	-	-
FUN_001709	GMC oxidoreductase	-	1.1.99.1	ko:K00108	ko00260, ko01100, map00260, map01100
FUN_001710	Fungal specific transcription factor domain	-	-	-	-
FUN_001712	Beta-lactamase	-	-	-	-
FUN_001713	HET domain-containing protein	-	-	-	-
FUN_001714	short-chain dehydrogenase	-	-	-	-
FUN_001715	Belongs to the major facilitator superfamily. Sugar transporter (TC 2.A.1.1) family	-	-	-	-
FUN_001716	Belongs to the major facilitator superfamily. Sugar transporter (TC 2.A.1.1) family	-	-	ko:K08150	-
FUN_001717	LsmAD	PBP1	-	-	-
FUN_001718	Actin interacting protein 3	BUD6	-	-	-
FUN_001719	WD domain, G-beta repeat	LST8	-	ko:K08266	ko04136, ko04138, ko04140, ko04150, ko04151, ko04714, map04136, map04138, map04140, map04150, map04151, map04714
FUN_001720	FMN-dependent dehydrogenase	-	-	-	-
FUN_001721	Sigma factor PP2C-like phosphatases	PTC2	3.1.3.16	ko:K14803	-
FUN_001722	Belongs to the acyl-CoA oxidase family	-	1.3.3.6	ko:K00232	ko00071, ko00592, ko01040, ko01100, ko01110, ko01212, ko03320, ko04024, ko04146, map00071, map00592, map01040, map01100, map01110, map01212, map03320, map04024, map04146
FUN_001723	Pfam:Y_phosphatase3C	-	-	-	-
FUN_001724	Eukaryotic porin	POR1	-	ko:K15040	ko04020, ko04022, ko04216, ko04217, ko04218, ko04621, ko04979, ko05012, ko05016, ko05166, map04020, map04022, map04216, map04217, map04218, map04621, map04979, map05012, map05016, map05166
FUN_001725	Cotranslationally removes the N-terminal methionine from nascent proteins. The N-terminal methionine is often cleaved when the second residue in the primary sequence is small and uncharged (Met-Ala-, Cys, Gly, Pro, Ser, Thr, or Val)	MAP2	3.4.11.18	ko:K01265	-
FUN_001726	Belongs to the ubiquitin-conjugating enzyme family	eff	2.3.2.23	ko:K06689	ko04013, ko04120, ko04141, ko04624, map04013, map04120, map04141, map04624
FUN_001727	Belongs to the VPS29 family	vps29	-	ko:K07095, ko:K18467	ko04144, map04144
FUN_001728	Inositol monophosphatase family	-	3.1.3.7	ko:K01082	ko00920, ko01100, ko01120, ko01130, map00920, map01100, map01120, map01130
FUN_001730	Aminotransferase class-V	-	2.6.1.44, 2.6.1.45, 2.6.1.51	ko:K00830	ko00250, ko00260, ko00630, ko00680, ko01100, ko01110, ko01120, ko01130, ko01200, ko04146, map00250, map00260, map00630, map00680, map01100, map01110, map01120, map01130, map01200, map04146
FUN_001731	Acts as subunit of the RNA polymerase II elongator complex, which is a histone acetyltransferase component of the RNA polymerase II (Pol II) holoenzyme and is involved in transcriptional elongation	ELP1	-	ko:K11373	-
FUN_001732	DSHCT	MTR4	3.6.4.13	ko:K11593, ko:K12598	ko03018, map03018

FUN_001733	Involved in ubiquitin-mediated protein degradation. Regulatory factor in the ubiquitin proteasome pathway that controls the turnover of proteasome substrates. Targets proteasomes to the nucleus and facilitates the degradation of nuclear proteins	STS1	-	ko:K11888	-
FUN_001736	Belongs to the glycosyl hydrolase 31 family	-	3.2.1.20	ko:K01187	ko00052, ko00500, ko01100, map00052, map00500, map01100
FUN_001740	polyketide synthase	-	-	ko:K15415	-
FUN_001741	The pathway begins with the synthesis of atrochrysone thioester by the polyketide synthase (PKS)	-	-	ko:K15318	-
FUN_001742	Domain of unknown function (DUF1772)	-	-	-	-
FUN_001743	EthD domain	-	-	-	-
FUN_001744	EthD domain	-	-	-	-
FUN_001747	Aflatoxin regulatory protein	-	-	-	-
FUN_001748	Fungal specific transcription factor domain	-	-	-	-
FUN_001750	Domain of unknown function (DUF4360)	-	-	-	-
FUN_001751	S-adenosyl-L-methionine-dependent protein-lysine N- methyltransferase that methylates elongation factor 1-alpha	EFM6	-	ko:K21804	-
FUN_001752	Na( ) H( ) antiporter	CNH1	-	ko:K03316	-
FUN_001753	Ammonium Transporter Family	-	-	ko:K03320	-
FUN_001754	Fungal specific transcription factor domain	-	-	-	-
FUN_001755	Chorismate synthase	ARO2	4.2.3.5	ko:K01736	ko00400, ko01100, ko01110, ko01130, ko01230, map00400, map01100, map01110, map01130, map01230
FUN_001756	Divergent CRAL/TRIO domain	-	-	ko:K18470	-
FUN_001758	Transcriptional repressor TCF25	-	-	-	-
FUN_001759	Sigma factor PP2C-like phosphatases	PTC5	3.1.3.43	ko:K01102	-
FUN_001761	Belongs to the short-chain dehydrogenases reductases (SDR) family	-	-	ko:K14729	-
FUN_001764	Sphingolipid Delta4-desaturase (DES)	DES1	1.14.18.5, 1.14.19.17	ko:K04712	ko00600, ko01100, ko04071, map00600, map01100, map04071
FUN_001765	37S ribosomal protein S25, mitochondrial	RSM25	-	ko:K17402	-
FUN_001766	PPR repeat	-	-	-	-
FUN_001767	Putative oxalocrotonate tautomerase enzyme	-	-	-	-
FUN_001768	NAD(P)H-binding	-	-	-	-
FUN_001770	Major Facilitator Superfamily	-	-	-	-
FUN_001771	Belongs to the D-isomer specific 2-hydroxyacid dehydrogenase family	-	-	-	-
FUN_001772	Sugar (and other) transporter	-	-	-	-
FUN_001773	alpha/beta hydrolase fold	-	-	-	-
FUN_001774	Sugar (and other) transporter	-	-	-	-
FUN_001775	Fungal specific transcription factor domain	-	-	-	-
FUN_001776	SUR7/PalI family	-	-	-	-
FUN_001777	Sugar (and other) transporter	-	-	ko:K08176	-
FUN_001782	Retinal pigment epithelial membrane protein	-	1.13.11.59	ko:K17842	ko00906, ko01100, map00906, map01100
FUN_001785	Fungal specific transcription factor domain	-	-	-	-
FUN_001786	FAD binding	-	-	-	-
FUN_001787	Belongs to the multicopper oxidase family	lcc1	-	-	-

FUN_001789	Glycosyltransferase family 25 protein	-	-	-	-
FUN_001790	Belongs to the mitochondrial carrier (TC 2.A.29) family	MIR1	-	ko:K15102	-
FUN_001792	Sugar (and other) transporter	-	-	-	-
FUN_001794	WD40 domain-containing protein	-	-	-	-
FUN_001795	Protein of unknown function (DUF1348)	-	-	-	-
FUN_001796	Eukaryotic cytochrome b561	-	-	-	-
FUN_001798	Protein of unknown function (DUF3445)	-	-	-	-
FUN_001799	Ribosomal protein L7Ae/L30e/S12e/Gadd45 family	SNU13	-	ko:K12845	ko03008, ko03040, map03008, map03040
FUN_001800	tRNA pseudouridine synthase D (TruD)	PUS7	5.4.99.27	ko:K06176	-
FUN_001801	Glycoside hydrolase family 55	-	3.2.1.58	ko:K01210	ko00500, map00500
FUN_001802	Aminotransferase class I and II	YEY2	-	-	-
FUN_001803	Choriogenin Hminor	-	-	-	-
FUN_001804	mitochondrial transport protein	fsf1	-	-	-
FUN_001805	Belongs to the heat shock protein 70 family	LHS1	-	ko:K09486	ko04141, map04141
FUN_001806	Major Facilitator Superfamily	-	-	-	-
FUN_001807	Domain in SPla and the RYanodine Receptor.	ssh4	-	-	-
FUN_001808	HEAT-like repeat	kap104	-	ko:K18752	-
FUN_001809	ubiquitin carboxyl-terminal hydrolase	-	3.4.19.12	ko:K05610	-
FUN_001816	Cupin domain	-	-	-	-
FUN_001817	ubiquitin carboxyl-terminal hydrolase	-	3.4.19.12	ko:K05609	-
FUN_001818	Amidohydrolase family	-	-	-	-
FUN_001821	Succinylglutamate desuccinylase / Aspartoacylase family	-	-	-	-
FUN_001825	Translation initiation factor	GCD1	-	ko:K03241	ko03013, map03013
FUN_001826	Peroxin 22-like protein	-	-	-	-
FUN_001827	Heterokaryon incompatibility protein (HET)	-	-	-	-
FUN_001829	Bacteriocin-protection, YdeI or OmpD-Associated	-	-	-	-
FUN_001830	integral membrane protein	-	-	-	-
FUN_001831	Belongs to the oxygen-dependent FAD-linked oxidoreductase family	-	-	-	-
FUN_001832	High affinity copper transporter	-	-	ko:K14686	ko01524, ko04978, map01524, map04978
FUN_001833	Ferric reductase like transmembrane component	-	-	-	-
FUN_001835	oxidoreductase	-	-	-	-
FUN_001836	Transmembrane amino acid transporter protein	-	-	ko:K14997	-
FUN_001837	Domain of unknown function (DUF1996)	-	-	-	-
FUN_001838	2, 3-dihydroxybenzoate decarboxylase	-	4.1.1.46	ko:K14333	ko00362, ko00627, ko01120, map00362, map00627, map01120
FUN_001840	Pyridine nucleotide-disulphide oxidoreductase	-	1.8.5.8	ko:K22470	ko00920, map00920
FUN_001841	Metallo-beta-lactamase domain protein	-	-	-	-
FUN_001842	Belongs to the oxygen-dependent FAD-linked oxidoreductase family	-	-	-	-
FUN_001843	Polysaccharide lyase family 4 protein	-	4.2.2.23	ko:K18195	-
FUN_001845	Kelch motif	-	-	-	-
FUN_001846	WD domain, G-beta repeat	rec14	-	ko:K12601, ko:K12602	ko03018, map03018
FUN_001847	Guanylate kinase homologues.	GUK1	2.7.4.8	ko:K00942	ko00230, ko01100, map00230, map01100

FUN_001848	Anaphase-promoting complex subunit 4 WD40 domain	GLE2	-	ko:K14298	ko03013, ko05164, map03013, map05164
FUN_001849	Rad52/22 family double-strand break repair protein	RAD52	-	ko:K10873	ko03440, map03440
FUN_001850	Protease with a carboxypeptidase B-like function involved in the C-terminal processing of the lysine and arginine residues from protein precursors. Promotes cell fusion and is involved in the programmed cell death (By similarity)	KEX1	3.4.16.6	ko:K01288	-
FUN_001851	Predicted SAM-dependent RNA methyltransferase	-	-	-	-
FUN_001852	Plays an essential role in initiation of the G0 program by preventing the degradation of specific nutrient-regulated mRNAs via the 5'-3' mRNA decay pathway	-	-	-	-
FUN_001853	PRELI-like family	UPS2	-	-	-
FUN_001855	Heavy-metal-associated domain	ATX1	-	ko:K07213	ko04978, map04978
FUN_001856	BRO1-like domain	RIM20	-	ko:K12200	ko04144, map04144
FUN_001858	C2H2-type zinc finger	CRZ1	-	ko:K21543	-
FUN_001859	Acyl transferase domain	MCT1	2.3.1.39	ko:K00645	ko00061, ko00333, ko01100, ko01130, ko01212, map00061, map00333, map01100, map01130, map01212
FUN_001860	KR domain	-	-	-	-
FUN_001861	Mediator complex subunit Med5	NUT1	-	ko:K15147	-
FUN_001863	Aldo/keto reductase family	-	-	-	-
FUN_001864	Belongs to the Glu Leu Phe Val dehydrogenases family	GDH3	1.4.1.4	ko:K00262	ko00220, ko00250, ko00910, ko01100, map00220, map00250, map00910, map01100
FUN_001867	cwf18 pre-mRNA splicing factor	-	-	ko:K12871	ko03040, map03040
FUN_001868	SUZ domain	-	-	-	-
FUN_001869	alpha/beta hydrolase fold	-	-	-	-
FUN_001870	UDP binding domain	-	1.1.1.22	ko:K00012	ko00040, ko00053, ko00520, ko01100, map00040, map00053, map00520, map01100
FUN_001871	UDP binding domain	-	1.1.1.22	ko:K00012	ko00040, ko00053, ko00520, ko01100, map00040, map00053, map00520, map01100
FUN_001874	Redoxin	DOT5	1.11.1.15	ko:K03564	-
FUN_001875	Transmembrane amino acid transporter protein	-	-	-	-
FUN_001877	Glycosyltransferase family 59 protein	ALG10	2.4.1.256	ko:K03850	ko00510, ko01100, map00510, map01100
FUN_001878	DNA replication complex GINS protein PSF2	PSF2	-	ko:K10733	-

FUN_001879	Guanine nucleotide exchange factor for Ras-like GTPases; N-terminal motif	BUD5	-	ko:K03099	ko01521, ko01522, ko04010, ko04012, ko04013, ko04014, ko04062, ko04068, ko04072, ko04150, ko04151, ko04320, ko04510, ko04540, ko04630, ko04650, ko04660, ko04662, ko04664, ko04714, ko04722, ko04810, ko04910, ko04912, ko04915, ko04917, ko04926, ko05034, ko05160, ko05165, ko05200, ko05205, ko05206, ko05210, ko05211, ko05213, ko05214, ko05215, ko05220, ko05221, ko05223, ko05224, ko05225, ko05226, ko05231, map01521, map01522, map04010, map04012, map04013, map04014, map04062, map04068, map04072, map04150, map04151, map04320, map04510, map04540, map04630, map04650, map04660, map04662, map04664, map04714, map04722, map04810, map04910, map04912, map04915, map04917, map04926, map05034, map05160, map05165, map05200, map05205, map05206, map05210, map05211, map05213, map05214, map05215, map05220, map05221, map05223, map05224, map05225, map05226, map05231
FUN_001880	Zinc-ribbon	-	-	ko:K19619	-
FUN_001881	60S ribosomal protein	RPL31B	-	ko:K02910	ko03010, map03010
FUN_001882	AFG1-like ATPase	-	3.6.4.7	ko:K18798	-
FUN_001883	Belongs to the complex I 20 kDa subunit family	-	1.6.5.3, 1.6.99.3	ko:K03940	ko00190, ko01100, ko04714, ko04723, ko04932, ko05010, ko05012, ko05016, map00190, map01100, map04714, map04723, map04932, map05010, map05012, map05016
FUN_001884	Glycosyltransferase family 4 protein	SPT14	2.4.1.198	ko:K03857	ko00563, ko01100, map00563, map01100
FUN_001885	helicase superfamily c-terminal domain	DBP6	3.6.4.13	ko:K14807	-
FUN_001886	Belongs to the peptidase A1 family	pepA	3.4.23.18	ko:K06004	-
FUN_001887	SRP19 protein	SEC65	-	ko:K03105	ko03060, map03060
FUN_001888	Belongs to the class-III pyridoxal-phosphate-dependent aminotransferase family	-	-	-	-
FUN_001889	Serine/Threonine protein kinases, catalytic domain	LKH1	2.7.12.1	ko:K08287	-
FUN_001890	Mis12 protein	-	-	ko:K11559	-
FUN_001891	Choline kinase N terminus	-	2.7.1.32, 2.7.1.82	ko:K00866, ko:K00894	ko00564, ko01100, map00564, map01100
FUN_001892	Membrane transport protein	-	-	ko:K07088	-
FUN_001893	Protein tyrosine kinase	wis1	2.7.12.2	ko:K11227	ko04011, ko04139, map04011, map04139
FUN_001894	Sec63 Brl domain	SEC63	-	ko:K09540	ko03060, ko04141, map03060, map04141
FUN_001897	PHD-finger	-	-	-	-
FUN_001899	Altered inheritance of mitochondria protein 3	-	-	-	-
FUN_001900	Very-long-chain 3-oxoacyl-CoA synthase	-	-	-	-
FUN_001901	Anaphase-promoting complex, subunit 10 (APC10)	-	-	ko:K03357	ko04110, ko04111, ko04113, ko04114, ko04120, ko04914, ko05166, map04110, map04111, map04113, map04114, map04120, map04914, map05166
FUN_001903	Belongs to the OMP decarboxylase family	URA3	2.4.2.10, 4.1.1.23	ko:K01591, ko:K13421	ko00240, ko00983, ko01100, map00240, map00983, map01100
FUN_001905	Catalytic LigB subunit of aromatic ring-opening dioxygenase	-	-	-	-

FUN_001906	Belongs to the multicopper oxidase family	-	-	ko:K19791	-
FUN_001907	Belongs to the multicopper oxidase family	-	-	ko:K19791	-
FUN_001908	Iron permease FTR1 family	FTR1	-	ko:K07243	-
FUN_001910	von Willebrand factor (vWF) type A domain	-	-	-	-
FUN_001911	Component of the SWR1 complex which mediates the ATP- dependent exchange of histone H2A for the H2A variant HZT1 leading to transcriptional regulation of selected genes by chromatin remodeling. Involved in chromosome stability	SWC5	-	-	-
FUN_001912	Macrophage migration inhibitory factor (MIF)	-	-	-	-
FUN_001913	CENP-S associating Centromere protein X	-	-	-	-
FUN_001915	Glycoside hydrolase family 2 protein	-	3.2.1.25	ko:K01192	ko00511, ko04142, map00511, map04142
FUN_001916	Glycoside hydrolase family 2 protein	-	3.2.1.25	ko:K01192	ko00511, ko04142, map00511, map04142
FUN_001917	Fungal specific transcription factor domain	-	-	-	-
FUN_001918	Ceramide hydroxylase involved in the alpha-hydroxylation of sphingolipid-associated very long chain fatty acids. Hydroxylates the very long chain fatty acid of ceramides at C2 and C3	SCS7	1.14.18.6	ko:K19703	-
FUN_001919	linker between RRM2 and RRM3 domains in RBM39 protein	rsd1	-	ko:K13091	-
FUN_001921	TAP-like protein	-	-	-	-
FUN_001923	EF-hand domain pair	CNB1	-	ko:K06268	ko04010, ko04020, ko04022, ko04114, ko04218, ko04310, ko04360, ko04370, ko04380, ko04650, ko04658, ko04659, ko04660, ko04662, ko04720, ko04724, ko04921, ko04922, ko04924, ko05010, ko05014, ko05031, ko05152, ko05166, ko05167, map04010, map04020, map04022, map04114, map04218, map04310, map04360, map04370, map04380, map04650, map04658, map04659, map04660, map04662, map04720, map04724, map04921, map04922, map04924, map05010, map05014, map05031, map05152, map05166, map05167
FUN_001924	Fungal protein of unknown function (DUF1770)	-	-	-	-
FUN_001925	Metal dependent phosphohydrolase	-	-	-	-
FUN_001926	dienelactone hydrolase	-	-	-	-
FUN_001927	Ferritin-like domain	-	-	-	-
FUN_001928	SNF2 family N-terminal domain	RDH54	-	ko:K10877	ko03440, map03440
FUN_001929	Phytanoyl-CoA dioxygenase	-	-	-	-
FUN_001930	RNA polymerase	-	-	-	-
FUN_001931	Belongs to the TCTP family	-	-	-	-
FUN_001932	Belongs to the sterol desaturase family	SUR2	1.14.18.5	ko:K04713	ko00600, ko01100, map00600, map01100
FUN_001938	Belongs to the class I-like SAM-binding methyltransferase superfamily. RsmB NOP	-	2.1.1.311	ko:K15264	-
FUN_001939	Uncharacterized conserved protein (DUF2183)	-	-	-	-
FUN_001940	GAT domain	-	-	-	-
FUN_001941	negative regulation of cell growth	-	-	-	-
FUN_001942	btb poz domain-containing protein	-	-	-	-
FUN_001943	Glutathione S-transferase, N-terminal domain	GTT1	2.5.1.18	ko:K00799	ko00480, ko00980, ko00982, ko00983, ko01524, ko05200, ko05204, ko05225, ko05418, map00480, map00980, map00982, map00983, map01524, map05200, map05204, map05225, map05418

FUN_001944	Opioid growth factor receptor (OGFr) conserved region	-	-	-	-
FUN_001945	Alpha amylase, catalytic domain	-	3.2.1.1	ko:K01176	ko00500, ko01100, ko04973, map00500, map01100, map04973
FUN_001948	Enoyl-(Acyl carrier protein) reductase	-	-	-	-
FUN_001949	A Receptor for Ubiquitination Targets	-	-	-	-
FUN_001952	FAD binding domain	-	-	-	-
FUN_001953	PhoD-like phosphatase, N-terminal domain	-	3.1.3.1	ko:K01113	ko00790, ko01100, ko02020, map00790, map01100, map02020
FUN_001954	to Saccharomyces cerevisiae BNI4 (YNL233W)	BNI4	-	ko:K18638	-
FUN_001955	ribosomal protein	RPS25	-	ko:K02975	ko03010, map03010
FUN_001956	Belongs to the cytochrome b5 family	-	-	-	-
FUN_001957	Gtr1/RagA G protein conserved region	GTR2	-	ko:K16186	ko04140, ko04150, map04140, map04150
FUN_001959	Signal recognition particle receptor beta subunit	ARL3	-	ko:K07952	-
FUN_001961	Dihydrouridine synthase (Dus)	DUS1	1.3.1.88	ko:K05542	-
FUN_001962	Protein present in Fab1, YOTB, Vac1, and EEA1	-	-	-	-
FUN_001963	Long-chain-alcohol oxidase	-	-	-	-
FUN_001964	acetyltransferase	-	-	-	-
FUN_001966	Belongs to the universal ribosomal protein uS13 family	RPS18	-	ko:K02964	ko03010, map03010
FUN_001968	SBDS protein C-terminal domain	-	-	ko:K14574	ko03008, map03008
FUN_001969	Vacuolar carboxypeptidase involved in degradation of small peptides. Digests preferentially peptides containing an aliphatic or hydrophobic residue in P1' position, as well as methionine, leucine or phenylalanine in P1 position of ester substrate (By	CPYA	3.4.16.5	ko:K13289	ko04142, ko04614, map04142, map04614
FUN_001970	Pyridine nucleotide-disulphide oxidoreductase	-	-	-	-
FUN_001971	Zinc-finger of mitochondrial splicing suppressor 51	MSS51	-	ko:K17656	-
FUN_001973	Belongs to the universal ribosomal protein uS8 family	RPS22	-	ko:K02957	ko03010, map03010
FUN_001976	Belongs to the glycosyl hydrolase 1 family	-	3.2.1.21	ko:K01188	ko00460, ko00500, ko00940, ko01100, ko01110, map00460, map00500, map00940, map01100, map01110
FUN_001977	Belongs to the ClpA ClpB family	HSP104	-	ko:K03695	ko04213, map04213
FUN_001978	Serine/threonine phosphatases, family 2C, catalytic domain	PTC6	3.1.3.16, 3.1.3.43	ko:K19708	ko04139, map04139
FUN_001980	Importin-beta N-terminal domain	kap95	-	ko:K14293	ko03013, map03013
FUN_001981	Ribonucleotide reductase inhibitor	-	-	-	-
FUN_001982	Fibrillarin	NOP1	-	ko:K14563	ko03008, map03008
FUN_001983	Glycoside hydrolase family 128 protein	-	-	-	-
FUN_001986	Polyketide cyclase / dehydrase and lipid transport	-	-	-	-
FUN_001987	Bestrophin, RFP-TM, chloride channel	-	-	ko:K08994	-
FUN_001990	Spt20 family	-	-	ko:K11361	-
FUN_001991	Belongs to the small heat shock protein (HSP20) family	-	-	ko:K13993	ko04141, map04141
FUN_001992	C2H2 type zinc-finger (2 copies)	-	-	ko:K14816	-
FUN_001993	Domain of unknown function (DUF4419)	-	-	-	-
FUN_001994	Belongs to the major facilitator superfamily. Sugar transporter (TC 2.A.1.1) family	-	-	-	-
FUN_001995	Predicted membrane protein (DUF2306)	-	-	-	-
FUN_001996	Enoyl-(Acyl carrier protein) reductase	-	-	-	-

FUN_001997	EF-hand domain	-	-	-	-
FUN_001998	cAMP phosphodiesterases class-II	TEF4	3.1.4.17	ko:K01120, ko:K03233	ko00230, ko05134, map00230, map05134
FUN_001999	Glutathione S-transferase, C-terminal domain	TEF4	3.1.4.17	ko:K01120, ko:K03233	ko00230, ko05134, map00230, map05134
FUN_002000	Belongs to the universal ribosomal protein uS9 family	MRPS9	-	ko:K02996	ko03010, map03010
FUN_002003	Domain of unknown function (DUF4267)	-	-	-	-
FUN_002007	Major Facilitator Superfamily	-	-	-	-
FUN_002008	RING-type zinc-finger	-	-	-	-
FUN_002009	Isochorismatase family	-	-	-	-
FUN_002012	Methyltransferase domain	-	-	-	-
FUN_002013	Belongs to the peptidase A1 family	-	-	-	-
FUN_002014	Beta-lactamase	-	-	-	-
FUN_002017	Belongs to the small heat shock protein (HSP20) family	-	-	-	-
FUN_002018	Heterokaryon incompatibility protein (HET)	-	-	-	-
FUN_002019	extracellular serine-rich protein	-	-	-	-
FUN_002020	Permease for cytosine/purines, uracil, thiamine, allantoin	-	-	ko:K03457	-
FUN_002021	Belongs to the iron ascorbate-dependent oxidoreductase family	-	-	-	-
FUN_002023	Amidohydrolase family	-	-	-	-
FUN_002024	Permease family	-	-	-	-
FUN_002026	Belongs to the MIP aquaporin (TC 1.A.8) family	AQY1	-	ko:K09885	-
FUN_002028	Belongs to the cytochrome P450 family	-	1.14.13.174	ko:K17645	ko00254, map00254
FUN_002029	Belongs to the purine-cytosine permease (2.A.39) family	TPN1	-	-	-
FUN_002032	Heterokaryon incompatibility protein (HET)	-	-	-	-
FUN_002034	Serine/Threonine protein kinases, catalytic domain	CDC15	2.7.11.1	ko:K06683	ko04111, ko04113, ko04392, map04111, map04113, map04392
FUN_002036	KR domain	-	-	-	-
FUN_002037	Belongs to the AAA ATPase family	-	-	ko:K08900	-
FUN_002038	Domain with 2 conserved Trp (W) residues	-	-	-	-
FUN_002039	Zinc-binding dehydrogenase	-	-	-	-
FUN_002040	Domain of unknown function (DUF1996)	-	-	-	-
FUN_002042	Heterokaryon incompatibility protein (HET)	-	-	-	-
FUN_002045	Glycoside hydrolase family 94 protein	-	2.4.1.321	ko:K18786	-
FUN_002047	Lipoate-protein ligase	-	6.3.1.20	ko:K03800	ko00785, ko01100, map00785, map01100
FUN_002048	FAD binding domain	-	-	-	-
FUN_002050	GRIP-related Arf-binding domain	-	-	-	-
FUN_002051	Ribosomal L32p protein family	-	-	ko:K02911	ko03010, map03010
FUN_002052	Belongs to the TRAFAC class TrmE-Era-EngA-EngB-Septin- like GTPase superfamily. Septin GTPase family	CDC10	-	ko:K16938	ko05100, map05100
FUN_002053	Essential protein Yae1, N terminal	-	-	-	-
FUN_002054	Belongs to the class-I aminoacyl-tRNA synthetase family	-	6.1.1.10	ko:K01874	ko00450, ko00970, map00450, map00970
FUN_002055	Maltose acetyltransferase	-	-	ko:K21632	-

FUN_002056	Ribosomal protein L3	-	-	ko:K02906	ko03010, map03010
FUN_002058	Uncharacterised protein family (UPF0160)	-	-	-	-
FUN_002059	ICE2	-	-	-	-
FUN_002060	Podospora anserina S mat genomic DNA chromosome	-	-	-	-
FUN_002061	Heterokaryon incompatibility protein (HET)	-	-	-	-
FUN_002062	Involved in protein N-glycosylation. Essential for the second step of the dolichol-linked oligosaccharide pathway	-	-	-	-
FUN_002063	Major Facilitator Superfamily	-	-	-	-
FUN_002067	Histone chaperone Rttp106-like	RTT106	-	-	-
FUN_002068	associated protein	-	-	-	-
FUN_002070	Conserved hypothetical protein	-	-	-	-
FUN_002071	Belongs to the MIP aquaporin (TC 1.A.8) family	AQY1	-	ko:K09885	-
FUN_002072	50S ribosome-binding GTPase	-	-	-	-
FUN_002073	50S ribosome-binding GTPase	-	-	-	-
FUN_002074	50S ribosome-binding GTPase	-	-	-	-
FUN_002075	50S ribosome-binding GTPase	-	-	-	-
FUN_002077	Belongs to the glycosyl hydrolase 43 family	-	-	-	-
FUN_002078	Ring hydroxylating alpha subunit (catalytic domain)	-	-	-	-
FUN_002079	Protein of unknown function (DUF3405)	-	-	-	-
FUN_002080	AAA family	-	-	-	-
FUN_002081	Heterokaryon incompatibility protein (HET)	-	-	-	-
FUN_002083	Belongs to the short-chain dehydrogenases reductases (SDR) family	-	-	-	-
FUN_002084	Cobalamin-independent synthase, N-terminal domain	MET6	2.1.1.14	ko:K00549	ko00270, ko00450, ko01100, ko01110, ko01230, map00270, map00450, map01100, map01110, map01230
FUN_002085	Major Facilitator Superfamily	-	-	-	-
FUN_002087	Methyltransferase domain	-	-	-	-
FUN_002088	NAD biosynthesis via nicotinamide riboside salvage pathway	-	2.7.7.1	ko:K19785	ko00760, ko01100, map00760, map01100
FUN_002089	nicotinamide-nucleotide adenylyltransferase activity	-	2.7.7.1	ko:K19785	ko00760, ko01100, map00760, map01100
FUN_002091	Belongs to the cytochrome P450 family	-	1.14.14.23	ko:K00489	ko00120, ko00140, ko01100, ko03320, ko04976, ko04979, map00120, map00140, map01100, map03320, map04976, map04979
FUN_002092	Heterokaryon incompatibility protein (HET)	-	-	-	-
FUN_002093	Belongs to the cytochrome P450 family	-	-	-	-
FUN_002096	nadh-ubiquinone oxidoreductase	-	-	-	-
FUN_002098	Belongs to the glycosyl hydrolase 5 (cellulase A) family	eglB	3.2.1.4	ko:K01179	ko00500, ko01100, map00500, map01100
FUN_002100	NAD(P)H-binding	-	-	-	-
FUN_002101	Antibiotic biosynthesis monooxygenase	-	-	-	-
FUN_002102	fad binding domain-containing protein	-	-	-	-
FUN_002108	Orotidine 5'-phosphate decarboxylase	URA3	2.4.2.10, 4.1.1.23	ko:K01591, ko:K13421	ko00240, ko00983, ko01100, map00240, map00983, map01100
FUN_002109	Domain of unknown function (DUF1996)	-	-	-	-
FUN_002110	PAS fold	-	-	-	-
FUN_002111	Belongs to the tannase family	faeB-1	3.1.1.73	ko:K09252	-

FUN_002114	ethyl tert-butyl ether degradation	-	-	-	-
FUN_002116	Domain of unknown function (DUF3328)	-	-	-	-
FUN_002117	Phosphoenolpyruvate phosphomutase	-	5.4.2.9	ko:K01841	ko00440, ko01100, ko01120, ko01130, map00440, map01100, map01120, map01130
FUN_002118	Domain of unknown function (DUF1996)	-	-	-	-
FUN_002119	Serine aminopeptidase, S33	-	3.5.1.101	ko:K18457	-
FUN_002122	Capsular polysaccharide synthesis protein	-	-	-	-
FUN_002124	Heterokaryon incompatibility protein	-	-	-	-
FUN_002125	Class II Aldolase and Adducin N-terminal domain	-	-	-	-
FUN_002127	Fungal specific transcription factor domain	-	-	-	-
FUN_002128	Belongs to the OSBP family	-	-	ko:K20456	-
FUN_002129	Belongs to the OSBP family	-	-	ko:K20456	-
FUN_002130	Belongs to the short-chain dehydrogenases reductases (SDR) family	-	-	-	-
FUN_002131	BTB/POZ domain	-	-	-	-
FUN_002133	NRDE-2, necessary for RNA interference	-	-	-	-
FUN_002134	BP28CT (NUC211) domain	UTP10	-	ko:K14550	ko03008, map03008
FUN_002135	LeuA allosteric (dimerisation) domain	LEU4	2.3.3.13	ko:K01649	ko00290, ko00620, ko01100, ko01110, ko01210, ko01230, map00290, map00620, map01100, map01110, map01210, map01230
FUN_002136	Uncharacterized conserved protein (DUF2347)	-	-	-	-
FUN_002137	KR domain	-	-	-	-
FUN_002138	Domain of unknown function (DUF1989)	-	-	-	-
FUN_002139	Catalyzes the hydrolytic deamination of adenine to hypoxanthine. Plays an important role in the purine salvage pathway and in nitrogen catabolism	AAH1	3.5.4.4	ko:K01488	ko00230, ko01100, ko05340, map00230, map01100, map05340
FUN_002140	Fungal specific transcription factor domain	-	-	-	-
FUN_002141	Belongs to the group II decarboxylase family	-	4.1.1.15	ko:K01580	ko00250, ko00410, ko00430, ko00650, ko01100, ko01110, ko01120, ko02024, ko04727, ko04940, map00250, map00410, map00430, map00650, map01100, map01110, map01120, map02024, map04727, map04940
FUN_002143	Catalyzes the formation of phosphatidylethanolamine (PtdEtn) from phosphatidylserine (PtdSer). Plays a central role in phospholipid metabolism and in the interorganelle trafficking of phosphatidylserine	PSD1	4.1.1.65	ko:K01613	ko00564, ko01100, ko01110, map00564, map01100, map01110
FUN_002145	Acyl-CoA thioesterase	TES1	3.1.2.2, 3.1.2.27	ko:K01068, ko:K11992	ko00062, ko00120, ko01040, ko01100, ko01110, ko04146, map00062, map00120, map01040, map01100, map01110, map04146
FUN_002146	DMAP1-binding Domain	-	-	-	-
FUN_002147	Ribosomal Proteins L2, RNA binding domain	RPL2	-	ko:K02938	ko03010, map03010
FUN_002151	snRNP Sm proteins	lsm7	-	ko:K12626	ko03018, ko03040, map03018, map03040
FUN_002152	GAL4-like Zn(II)2Cys6 (or C6 zinc) binuclear cluster DNA-binding domain	-	-	-	-
FUN_002154	Fungal specific transcription factor domain	-	-	-	-
FUN_002155	Major Facilitator Superfamily	-	-	-	-
FUN_002156	Alpha/beta hydrolase family	-	-	-	-
FUN_002157	Glycosyltransferase family 71 protein	-	-	ko:K05535	ko00513, ko01100, map00513, map01100
FUN_002158	Short chain dehydrogenase	-	-	-	-
FUN_002159	Starch synthase catalytic domain	-	2.4.1.183	ko:K00749	-

FUN_002160	Major facilitator superfamily transporter	-	-	-	-
FUN_002163	Methyltransferase domain	-	-	-	-
FUN_002165	Zinc-binding dehydrogenase	-	-	-	-
FUN_002167	Membrane-associating domain	-	-	-	-
FUN_002168	Glycosyl hydrolase family 65, N-terminal domain	-	-	-	-
FUN_002170	Alpha/beta hydrolase family	-	-	-	-
FUN_002172	Amidase	-	3.5.1.4	ko:K01426	ko00330, ko00360, ko00380, ko00627, ko00643, ko01120, map00330, map00360, map00380, map00627, map00643, map01120
FUN_002174	Catalyzes the removal of a penultimate prolyl residue from the N-termini of peptides	PEPP	3.4.13.9	ko:K14213	-
FUN_002176	Tubulin domain	DML1	-	-	-
FUN_002177	Prp18 domain	-	-	ko:K12817	ko03040, map03040
FUN_002178	Saccharomyces cerevisiae YJR098c	-	-	-	-
FUN_002182	Carbon-nitrogen hydrolase	-	3.5.5.1	ko:K01501	ko00380, ko00460, ko00627, ko00643, ko00910, ko01120, map00380, map00460, map00627, map00643, map00910, map01120
FUN_002184	integral membrane protein	-	-	-	-
FUN_002185	Oxidoreductase family, NAD-binding Rossmann fold	-	-	-	-
FUN_002187	Major Facilitator Superfamily	-	-	-	-
FUN_002189	Belongs to the major facilitator superfamily. Sugar transporter (TC 2.A.1.1) family	-	-	-	-
FUN_002192	Belongs to the cytochrome P450 family	-	1.14.14.23	ko:K00489	ko00120, ko00140, ko01100, ko03320, ko04976, ko04979, map00120, map00140, map01100, map03320, map04976, map04979
FUN_002194	Amino acid permease	-	-	-	-
FUN_002195	Belongs to the D-isomer specific 2-hydroxyacid dehydrogenase family	-	1.1.1.29	ko:K00018	ko00260, ko00630, ko00680, ko01100, ko01110, ko01120, ko01130, ko01200, map00260, map00630, map00680, map01100, map01110, map01120, map01130, map01200
FUN_002197	SDA1	SDA1	-	ko:K14856	-
FUN_002198	Dimerisation domain of Zinc Transporter	-	-	-	-
FUN_002200	Flavin reductase like domain	-	-	-	-
FUN_002201	alpha/beta hydrolase fold	-	2.3.1.31	ko:K00641	ko00270, ko01100, ko01130, map00270, map01100, map01130
FUN_002202	Poly(A) polymerase predicted RNA binding domain	PAP1	2.7.7.19	ko:K14376	ko03015, map03015
FUN_002203	Transcription initiation factor TFIID component TAF4 family	-	-	-	-
FUN_002204	CPSF73-100_C	YSH1	-	ko:K14403	ko03015, map03015
FUN_002206	Belongs to the small GTPase superfamily. Arf family	-	-	ko:K07943	-
FUN_002208	Domain of unknown function (DUF4045)	-	-	-	-
FUN_002209	Lytic polysaccharide monooxygenase	-	1.14.99.54	ko:K19356	-
FUN_002210	Belongs to the heat shock protein 70 family	-	-	-	-
FUN_002212	Belongs to the CDP-alcohol phosphatidyltransferase class-I family	pis1	2.7.8.11	ko:K00999	ko00562, ko00564, ko01100, ko04070, map00562, map00564, map01100, map04070
FUN_002213	helicase superfamily c-terminal domain	DRS1	3.6.4.13	ko:K13181	-
FUN_002214	protein side chain deglutamylation	-	2.3.2.31	ko:K11971, ko:K11976	-
FUN_002215	Belongs to the histidine acid phosphatase family	-	3.1.3.2	ko:K01078	ko00730, ko00740, ko01100, map00730, map00740, map01100

FUN_002216	PPR repeat family	-	-	-	-
FUN_002217	bZIP transcription factor	-	-	ko:K21452	-
FUN_002218	tRNase Z endonuclease	-	3.1.26.11	ko:K00784	ko03013, map03013
FUN_002219	Domain of unknown function (DUF3328)	-	-	-	-
FUN_002220	Methyltransferase domain	-	-	-	-
FUN_002221	Amidase	-	-	-	-
FUN_002222	N2227	-	2.1.1.22	ko:K19787	ko00340, map00340
FUN_002223	Autophagy-related protein 27	ATG27	-	ko:K21141	ko04138, map04138
FUN_002224	Hydrolyzes a variety of simple alpha-D-galactoside as well as more complex molecules such as oligosaccharides and polysaccharides	aglB	3.2.1.22	ko:K07407	ko00052, ko00561, ko00600, ko00603, map00052, map00561, map00600, map00603
FUN_002225	Emopamil binding protein	-	5.3.3.5	ko:K01824	ko00100, ko01100, ko01110, map00100, map01100, map01110
FUN_002228	D-arabinono-1, 4-lactone oxidase	ALO1	1.1.3.37, 1.1.3.8	ko:K00103, ko:K00107	ko00053, ko01100, map00053, map01100
FUN_002231	Transmembrane alpha-helix domain-containing protein	-	-	-	-
FUN_002233	short chain dehydrogenase	-	-	-	-
FUN_002236	Acid phosphatase homologues	-	3.1.3.4, 3.1.3.81	ko:K18693	ko00561, ko00564, ko01110, map00561, map00564, map01110
FUN_002237	Molybdenum Cofactor Synthesis C	-	4.1.99.22, 4.6.1.17	ko:K03639, ko:K20967	ko00790, ko01100, ko04122, map00790, map01100, map04122
FUN_002240	FAD binding domain	-	1.14.13.1	ko:K00480	ko00621, ko00624, ko00626, ko01100, ko01120, ko01220, map00621, map00624, map00626, map01100, map01120, map01220
FUN_002241	Belongs to the cytochrome P450 family	-	-	-	-
FUN_002243	GMC oxidoreductase	-	-	-	-
FUN_002244	Permease for cytosine/purines, uracil, thiamine, allantoin	-	-	ko:K03457	-
FUN_002245	2OG-Fe(II) oxygenase superfamily	-	-	-	-
FUN_002246	The coatomer is a cytosolic protein complex that binds to dilysine motifs and reversibly associates with Golgi non- clathrin-coated vesicles, which further mediate biosynthetic protein transport from the ER, via the Golgi up to the trans Golgi network. Coatomer complex is required for budding from Golgi membranes, and is essential for the retrograde Golgi-to-ER transport of dilysine-tagged proteins	RET2	-	ko:K20471	-
FUN_002248	Magnesium transporter NIPA	-	-	-	-
FUN_002249	Anaphase-promoting complex, cyclosome, subunit 4	-	-	ko:K03351	ko04110, ko04111, ko04113, ko04114, ko04120, ko04914, ko05166, map04110, map04111, map04113, map04114, map04120, map04914, map05166
FUN_002251	Glycoside hydrolase family 125 protein	-	-	ko:K09704	-
FUN_002253	FAD-binding domain	-	-	-	-
FUN_002254	Belongs to the glycosyl hydrolase 11 (cellulase G) family	-	3.2.1.8	ko:K01181	-
FUN_002255	GAL4-like Zn(II)2Cys6 (or C6 zinc) binuclear cluster DNA-binding domain	-	-	-	-
FUN_002256	Key enzyme for ketone body catabolism. Transfers the CoA moiety from succinate to acetoacetate. Formation of the enzyme-CoA intermediate proceeds via an unstable anhydride species formed between the carboxylate groups of the enzyme and substrate	-	2.8.3.5	ko:K01027	ko00072, ko00280, ko00650, map00072, map00280, map00650
FUN_002257	O-methyltransferase	-	-	-	-

FUN_002258	Adds the first Dol-P-Man derived mannose in an alpha-1, 3 linkage to Man(5)GlcNAc(2)-PP-Dol	ALG3	2.4.1.258	ko:K03845	ko00510, ko00513, ko01100, map00510, map00513, map01100
FUN_002259	Ion channel regulatory protein UNC-93	-	-	-	-
FUN_002260	Possible catecholamine-binding domain present in a variety of eukaryotic proteins.	-	-	-	-
FUN_002261	Inositol phosphatase	-	-	ko:K21798	ko00562, ko01100, ko04070, map00562, map01100, map04070
FUN_002262	Belongs to the major facilitator superfamily. Sugar transporter (TC 2.A.1.1) family	HGT1	-	-	-
FUN_002264	Belongs to the GMC oxidoreductase family	-	-	-	-
FUN_002266	Amino acid permease	-	-	-	-
FUN_002268	Amidohydrolase family	-	-	-	-
FUN_002270	cell wall protein	-	-	-	-
FUN_002273	Glycoside hydrolase family 61 protein	-	-	-	-
FUN_002274	extracellular serine-rich protein	-	-	-	-
FUN_002275	Enoyl-(Acyl carrier protein) reductase	-	-	-	-
FUN_002276	resistance protein	ATR1	-	ko:K08165	-
FUN_002277	Alcohol dehydrogenase GroES-like domain	lad1	1.1.1.14	ko:K00008	ko00040, ko00051, ko01100, map00040, map00051, map01100
FUN_002278	Alcohol dehydrogenase GroES-like domain	-	-	-	-
FUN_002279	tRNA synthetases class II core domain (F)	MSK1	6.1.1.6	ko:K04567	ko00970, map00970
FUN_002280	Alcohol dehydrogenase GroES-like domain	xdhA	1.1.1.14, 1.1.1.9	ko:K00008, ko:K05351	ko00040, ko00051, ko01100, map00040, map00051, map01100
FUN_002281	PRO41 protein	-	-	-	-
FUN_002282	FAD-binding domain	-	-	ko:K08008	ko04380, ko04933, ko05418, map04380, map04933, map05418
FUN_002283	Alcohol dehydrogenase GroES-like domain	xdhA	1.1.1.14, 1.1.1.9	ko:K00008, ko:K05351	ko00040, ko00051, ko01100, map00040, map00051, map01100
FUN_002284	Component of the ESCRT-I complex (endosomal sorting complex required for transport I), a regulator of vesicular trafficking process	VPS28	-	ko:K12184	ko04144, map04144
FUN_002285	Noc2p family	NOC2	-	ko:K14833	-
FUN_002286	ABC transporter transmembrane region 2	PXA1	-	ko:K15628	ko02010, ko04146, map02010, map04146
FUN_002287	Transcription factor	-	-	-	-
FUN_002288	RING-like zinc finger	-	-	-	-
FUN_002289	Histone acetyltransferases subunit 3	NGG1	-	ko:K11315	ko05165, map05165
FUN_002290	Belongs to the aldehyde dehydrogenase family	-	-	-	-
FUN_002291	Domain of unknown function (DUF4360)	-	-	-	-
FUN_002293	Acyltransferase family	-	-	ko:K03135	ko03022, map03022
FUN_002294	clavaminate synthase-like protein	-	-	-	-
FUN_002295	FRG1-like domain	-	-	ko:K13122	-
FUN_002296	Epoxide hydrolase N terminus	-	-	-	-
FUN_002297	Histidine biosynthesis trifunctional protein	HIS4	1.1.1.23, 3.5.4.19, 3.6.1.31	ko:K14152	ko00340, ko01100, ko01110, ko01230, map00340, map01100, map01110, map01230
FUN_002298	CBM1-GH10. carbohydrate-binding module family 1	-	3.2.1.8	ko:K01181	-

FUN_002299	Rab subfamily of small GTPases	-	-	ko:K07879, ko:K07880	ko04144, map04144
FUN_002300	DNA repair protein	-	-	-	-
FUN_002302	motif in proteasome subunits, Int-6, Nip-1 and TRIP-15	-	-	ko:K12180	-
FUN_002303	Heavy-metal-associated domain	CCC2	3.6.3.54	ko:K17686	ko01524, ko04016, map01524, map04016
FUN_002304	phospholipase	-	3.1.1.47	ko:K01062	ko00565, ko01100, ko01110, ko01130, map00565, map01100, map01110, map01130
FUN_002305	SAD/SRA domain	-	-	-	-
FUN_002307	Sel1-like repeats.	HRD3	-	ko:K14023, ko:K14026	ko04141, map04141
FUN_002308	Sugar (and other) transporter	-	-	-	-
FUN_002309	helicase activity	-	3.6.4.13	ko:K11594, ko:K20096	ko04622, ko05161, ko05203, map04622, map05161, map05203
FUN_002311	Plant family of unknown function (DUF810)	-	-	-	-
FUN_002312	helix loop helix domain	-	-	-	-
FUN_002313	Saccharomyces cerevisiae YIL110w	-	2.1.1.85	ko:K18803	-
FUN_002315	Anticodon binding domain	MST1	6.1.1.3	ko:K01868	ko00970, map00970
FUN_002316	Protein tyrosine kinase	-	-	-	-
FUN_002317	Belongs to the FPP GGPP synthase family	BTS1	2.5.1.1, 2.5.1.10, 2.5.1.29	ko:K00804	ko00900, ko01100, ko01110, ko01130, map00900, map01100, map01110, map01130
FUN_002320	GDP/GTP exchange factor Sec2p	-	-	-	-
FUN_002321	Src homology 3 domains	BUD14	-	-	-
FUN_002322	Protein of unknown function (DUF1479)	-	-	-	-
FUN_002323	Belongs to the glycosyl hydrolase 1 family	-	3.2.1.21	ko:K01188	ko00460, ko00500, ko00940, ko01100, ko01110, map00460, map00500, map00940, map01100, map01110
FUN_002324	basic region leucin zipper	-	-	-	-
FUN_002325	Belongs to the iron ascorbate-dependent oxidoreductase family	-	-	-	-
FUN_002326	Senescence-associated protein	-	-	ko:K19366	ko04144, map04144
FUN_002327	Cation transport protein	-	-	-	-
FUN_002330	NFX1-type zinc finger-containing protein 1	-	-	-	-
FUN_002331	AAA domain	-	-	-	-
FUN_002332	geranylgeranyl pyrophosphate synthetase	-	-	-	-
FUN_002333	BTB/POZ domain	-	-	-	-
FUN_002334	BTB/POZ domain	-	-	-	-
FUN_002335	PCI domain	CSN12	-	-	-
FUN_002336	Belongs to the eukaryotic ribosomal protein eL20 family	RPL20B	-	ko:K02882	ko03010, map03010
FUN_002337	Enoyl-CoA hydratase/isomerase	-	-	-	-
FUN_002339	Acyclic terpene utilisation family protein AtuA	-	-	-	-
FUN_002341	Common central domain of tyrosinase	-	1.14.18.1	ko:K00505	ko00350, ko00950, ko00965, ko01100, ko01110, ko04916, map00350, map00950, map00965, map01100, map01110, map04916
FUN_002342	Fungal specific transcription factor domain	-	-	-	-

FUN_002343	Mandelate racemase muconate lactonizing enzyme	-	4.2.1.90	ko:K12661	ko00051, ko01120, map00051, map01120
FUN_002344	UTP--glucose-1-phosphate uridylyltransferase	UAP1	2.7.7.23, 2.7.7.83	ko:K00972	ko00520, ko01100, ko01130, map00520, map01100, map01130
FUN_002345	Belongs to the RecA family	-	-	ko:K10872, ko:K14545	ko03008, ko04113, map03008, map04113
FUN_002347	MAP kinase kinase kinase activity	-	-	-	-
FUN_002350	FAD dependent oxidoreductase	-	1.4.3.3	ko:K00273	ko00260, ko00311, ko00330, ko00472, ko01100, ko01130, ko04146, map00260, map00311, map00330, map00472, map01100, map01130, map04146
FUN_002351	Occurs in almost all aerobically respiring organisms and serves to protect cells from the toxic effects of hydrogen peroxide	cat1	1.11.1.6	ko:K03781	ko00380, ko00630, ko01110, ko01130, ko01200, ko04011, ko04016, ko04068, ko04146, ko04211, ko04212, ko04213, ko05014, map00380, map00630, map01110, map01130, map01200, map04011, map04016, map04068, map04146, map04211, map04212, map04213, map05014
FUN_002352	Component of the ERMES MDM complex, which serves as a molecular tether to connect the endoplasmic reticulum and mitochondria. Components of this complex are involved in the control of mitochondrial shape and protein biogenesis and may function in phospholipid exchange. mdm12 is required for the interaction of the ER-resident membrane protein mmm1 and the outer mitochondrial membrane-resident beta-barrel protein mdm10. The mdm12-mmm1 subcomplex functions in the major beta-barrel assembly pathway that is responsible for biogenesis of all mitochondrial outer membrane beta-barrel proteins, and acts in a late step after the SAM complex. The mdm10-mdm12-mmm1 subcomplex further acts in the tom40-specific pathway after the action of the mdm12-mmm1 complex. Essential for establishing and maintaining the	MDM12	-	ko:K17765	ko04139, map04139
FUN_002353	Heterokaryon incompatibility protein Het-C	-	-	-	-
FUN_002354	Nup85 Nucleoporin	-	-	-	-
FUN_002355	Las17-binding protein actin regulator	-	-	-	-
FUN_002356	Ribosomal RNA-processing protein 7 (RRP7)	-	-	ko:K14545	ko03008, map03008
FUN_002357	arrestin	-	-	-	-
FUN_002359	Acyl-CoA dehydrogenase, C-terminal domain	-	-	-	-
FUN_002363	Methyltransferase domain	laeA	-	-	-
FUN_002364	Sodium Bile acid symporter family	ARR3	-	ko:K03325	-
FUN_002365	Bacterial protein of unknown function (DUF899)	-	-	-	-
FUN_002366	Major Facilitator Superfamily	-	-	-	-
FUN_002369	Glycoside hydrolase family 29 protein	-	3.2.1.51	ko:K01206	ko00511, map00511
FUN_002373	Aromatic acid exporter family member 2	-	-	-	-
FUN_002374	Utp8 family	-	-	-	-
FUN_002375	ATPase family associated with various cellular activities (AAA)	-	-	ko:K11134, ko:K17922	-
FUN_002376	Alb1	-	-	-	-
FUN_002378	Hydroxyacylglutathione hydrolase C-terminus	GLO2	3.1.2.6	ko:K01069	ko00620, map00620
FUN_002381	Signal recognition particle 9 kDa protein (SRP9)	-	-	-	-
FUN_002382	Protein of unknown function (DUF1308)	-	-	-	-
FUN_002383	NAD(P)H-binding	-	-	-	-

FUN_002385	Alkali metal cation/H+ antiporter Nha1 C terminus	CNH1	-	ko:K03316	-
FUN_002386	Arrestin (or S-antigen), C-terminal domain	-	-	ko:K20061	-
FUN_002387	twin BRCT domain	DPB11	-	ko:K03507	-
FUN_002388	Belongs to the iron ascorbate-dependent oxidoreductase family	-	-	-	-
FUN_002389	Temperature dependent protein affecting M2 dsRNA replication	-	-	-	-
FUN_002390	Heterokaryon incompatibility protein (HET)	-	-	-	-
FUN_002391	Carbohydrate esterase family 1 protein	-	-	-	-
FUN_002392	Microtubule associated protein (MAP65/ASE1 family)	ASE1	-	ko:K16732	-
FUN_002394	PalH/RIM21	RIM21	-	-	-
FUN_002395	PhoX homologous domain, present in p47phox and p40phox.	MVP1	-	ko:K17922	-
FUN_002396	Neddylation of cullins play an essential role in the regulation of SCF-type complexes activity	DCN1	-	ko:K17822	-
FUN_002397	Ribosomal_L32e	RPL32	-	ko:K02912	ko03010, map03010
FUN_002400	Glycosyl hydrolase family 61	-	-	-	-
FUN_002401	AMP-binding enzyme	-	6.2.1.16	ko:K01907	ko00280, ko00650, map00280, map00650
FUN_002402	Major Facilitator Superfamily	-	-	-	-
FUN_002403	lipoamidase activity	SIR2rp2	-	ko:K11414	-
FUN_002404	RTA1 like protein	-	-	-	-
FUN_002405	GAL4-like Zn(II)2Cys6 (or C6 zinc) binuclear cluster DNA-binding domain	-	-	-	-
FUN_002408	Domain of unknown function (DUF1929)	-	-	-	-
FUN_002409	Belongs to the glycosyl hydrolase 18 family	CHT2	3.2.1.14	ko:K01183	ko00520, ko01100, map00520, map01100
FUN_002410	SET (Su(var)3-9, Enhancer-of-zeste, Trithorax) domain	-	-	-	-
FUN_002411	Peroxidase	-	-	-	-
FUN_002412	Responsible for synthesis of pseudouridine from uracil	RIB2	5.4.99.28	ko:K14655, ko:K15454	ko00740, ko01100, map00740, map01100
FUN_002413	Oxidoreductase family, NAD-binding Rossmann fold	-	1.1.99.28	ko:K00118	-
FUN_002414	Major Facilitator Superfamily	-	-	-	-
FUN_002415	trichothecene efflux pump	-	-	-	-
FUN_002416	Aldo/keto reductase family	-	-	-	-
FUN_002417	EthD domain	-	-	-	-
FUN_002418	Acyclic terpene utilisation family protein AtuA	-	-	-	-
FUN_002419	cytochrome p450	-	-	-	-
FUN_002420	oxidation-reduction process	-	-	-	-
FUN_002422	Glyoxalase/Bleomycin resistance protein/Dioxygenase superfamily	-	-	-	-
FUN_002423	alcohol dehydrogenase	-	1.1.1.1	ko:K13953	ko00010, ko00071, ko00350, ko00625, ko00626, ko00830, ko00980, ko00982, ko01100, ko01110, ko01120, ko01130, ko01220, map00010, map00071, map00350, map00625, map00626, map00830, map00980, map00982, map01100, map01110, map01120, map01130, map01220
FUN_002424	Dehydrogenase	-	-	-	-
FUN_002425	Enoyl-(Acyl carrier protein) reductase	-	-	-	-
FUN_002428	Belongs to the class-III pyridoxal-phosphate-dependent aminotransferase family	-	-	-	-
FUN_002429	aminopeptidase	APE2	-	ko:K13721	-

FUN_002431	Major Facilitator Superfamily	-	-	-	-
FUN_002432	Heterokaryon incompatibility protein (HET)	-	-	-	-
FUN_002433	TAP-like protein	-	-	-	-
FUN_002435	Eukaryotic rRNA processing protein EBP2	ebp2	-	ko:K14823	-
FUN_002436	Heat shock factor binding protein 1	-	-	ko:K19765	ko04212, map04212
FUN_002437	Belongs to the protein disulfide isomerase family	tigA	5.3.4.1	ko:K09584	ko04141, map04141
FUN_002438	Thioredoxin-like domain	-	-	ko:K03671	ko04621, ko05418, map04621, map05418
FUN_002439	WD domain, G-beta repeat	-	-	ko:K20241	-
FUN_002440	Belongs to the FMO family	-	1.14.13.8	ko:K00485	ko00982, ko01120, map00982, map01120
FUN_002441	Ribonucleotide reductase, small chain	RNR2	1.17.4.1	ko:K10808	ko00230, ko00240, ko00480, ko00983, ko01100, ko04115, map00230, map00240, map00480, map00983, map01100, map04115
FUN_002442	Cytochrome P450	-	-	-	-
FUN_002443	Methyltransferase domain-containing protein	-	-	-	-
FUN_002445	Kinesin-associated protein (KAP)	VAC8	-	ko:K08332	-
FUN_002446	Protein tyrosine kinase	-	-	-	-
FUN_002447	Domain of unknown function (DUF5127)	-	-	-	-
FUN_002450	Transport protein particle (TRAPP) component	trs31	-	ko:K20280	-
FUN_002451	COMPASS (Complex proteins associated with Set1p) component shg1	-	-	-	-
FUN_002452	Nadh-ubiquinone oxidoreductase 14 kda subunit	-	-	-	-
FUN_002453	Uncharacterised protein family UPF0052	-	-	-	-
FUN_002457	Gpi-anchored cell surface glycoprotein	-	-	-	-
FUN_002458	OHCU decarboxylase	-	-	-	-
FUN_002460	Belongs to the mitochondrial carrier (TC 2.A.29) family	OAC1	-	ko:K15117	-
FUN_002461	WD domain, G-beta repeat	-	-	ko:K14962	ko03015, map03015
FUN_002464	RTA1 like protein	-	-	-	-
FUN_002465	Plug domain of Sec61p	SEC61	-	ko:K10956	ko03060, ko04141, ko04145, ko05110, map03060, map04141, map04145, map05110
FUN_002466	Acetyltransferase (GNAT) domain	-	-	-	-
FUN_002467	BTB/POZ domain	-	-	-	-
FUN_002468	Belongs to the glycosyl hydrolase 31 family	-	3.2.1.20	ko:K01187	ko00052, ko00500, ko01100, map00052, map00500, map01100
FUN_002469	Carbon-nitrogen hydrolase	NIT3	3.5.1.3	ko:K13566	ko00250, map00250
FUN_002472	belongs to the metallo- dependent hydrolases superfamily. Urease alpha subunit family	URE1	3.5.1.5	ko:K01427	ko00220, ko00230, ko00791, ko01100, ko01120, map00220, map00230, map00791, map01100, map01120
FUN_002473	high mobility group	-	-	-	-
FUN_002475	BrlA, abaA and wetA are pivotal regulators of conidiophore development and conidium maturation (By similarity). They act individually and together to regulate their own expression and that of numerous other sporulation-specific genes (By similarity). Binds to the sequence 5'-CATTCY-3', where Y is a pyrimidine, making both major- and minor-groove contacts (By similarity)	abaA	-	ko:K09448	ko04011, ko04390, ko04391, ko04392, map04011, map04390, map04391, map04392
FUN_002476	RNA recognition motif	MRD1	-	ko:K14787	-
FUN_002477	Dynein light chain type 1	DYN2	-	ko:K10418	ko04962, map04962
FUN_002480	RNA recognition motif	-	-	-	-

FUN_002481	scytalone dehydratase	-	4.2.1.94	ko:K17740	-
FUN_002483	Maf-like protein	-	-	ko:K06287	-
FUN_002484	N-terminal domain of oxidoreductase	-	-	ko:K07119	-
FUN_002488	belongs to the flavoprotein pyridine nucleotide cytochrome reductase family	MCR1	1.6.2.2	ko:K00326	ko00520, map00520
FUN_002490	Capsule polysaccharide synthase Cps1	-	-	-	-
FUN_002493	GAL4-like Zn(II)2Cys6 (or C6 zinc) binuclear cluster DNA-binding domain	ARO80	-	-	-
FUN_002494	Allantoate permease	-	-	-	-
FUN_002496	Methyltransferase domain	-	-	-	-
FUN_002497	Alpha and gamma adaptin binding protein p34	-	-	-	-
FUN_002498	Mitochondrial PGP phosphatase	-	3.1.3.27	ko:K01094	ko00564, ko01100, map00564, map01100
FUN_002499	Belongs to the peptidase M16 family	MAS2	3.4.24.64	ko:K01412	-
FUN_002500	Forkhead associated domain	-	-	ko:K08994	-
FUN_002502	Bestrophin, RFP-TM, chloride channel	-	-	ko:K08994	-
FUN_002503	SprT-like family	-	-	-	-
FUN_002504	Belongs to the peroxisomal membrane protein PXMP2 4 family	-	-	-	-
FUN_002506	von Willebrand factor (vWF) type A domain	-	-	-	-
FUN_002508	ATP-NAD kinase	-	2.7.1.23	ko:K00858	ko00760, ko01100, map00760, map01100
FUN_002509	PLAC8 family	-	-	-	-
FUN_002511	TATA-binding protein interacting (TIP20)	TIP120	-	ko:K17263	-
FUN_002512	Uncharacterized protein conserved in bacteria (DUF2263)	-	-	-	-
FUN_002513	Porphobilinogen deaminase, dipyromethane cofactor binding domain	HEM3	2.5.1.61	ko:K01749	ko00860, ko01100, ko01110, ko01120, map00860, map01100, map01110, map01120
FUN_002514	Permease for cytosine/purines, uracil, thiamine, allantoin	fur4	-	ko:K03457	-
FUN_002517	Ribosomal L22e protein family	rpl22	-	ko:K02891	ko03010, map03010
FUN_002518	KR domain	-	-	-	-
FUN_002520	Cell division control protein 14, SIN component	-	-	-	-
FUN_002521	PAP2 superfamily	-	-	-	-
FUN_002523	Arrestin_N terminal like	LDB19	-	ko:K20059	-
FUN_002525	Glycoside hydrolase family 16 protein	-	-	-	-

FUN_002526	Guanine nucleotide exchange factor for Ras-like GTPases; N-terminal motif	CDC25	-	ko:K03099	ko01521, ko01522, ko04010, ko04012, ko04013, ko04014, ko04062, ko04068, ko04072, ko04150, ko04151, ko04320, ko04510, ko04540, ko04630, ko04650, ko04660, ko04662, ko04664, ko04714, ko04722, ko04810, ko04910, ko04912, ko04915, ko04917, ko04926, ko05034, ko05160, ko05165, ko05200, ko05205, ko05206, ko05210, ko05211, ko05213, ko05214, ko05215, ko05220, ko05221, ko05223, ko05224, ko05225, ko05226, ko05231, map01521, map01522, map04010, map04012, map04013, map04014, map04062, map04068, map04072, map04150, map04151, map04320, map04510, map04540, map04630, map04650, map04660, map04662, map04664, map04714, map04722, map04810, map04910, map04912, map04915, map04917, map04926, map05034, map05160, map05165, map05200, map05205, map05206, map05210, map05211, map05213, map05214, map05215, map05220, map05221, map05223, map05224, map05225, map05226, map05231
FUN_002527	Fungal specific transcription factor domain	-	-	-	-
FUN_002528	Ribosomal protein L7Ae/L30e/S12e/Gadd45 family	-	-	ko:K11129	ko03008, map03008
FUN_002529	Amino acid permease	-	-	-	-
FUN_002531	DnaJ molecular chaperone homology domain	-	-	ko:K09537	-
FUN_002532	TruB family pseudouridylate synthase (N terminal domain)	PUS4	5.4.99.25	ko:K03177	-
FUN_002533	Solute carrier family 35	-	-	ko:K15289	-
FUN_002534	Gtr1/RagA G protein conserved region	-	-	ko:K07897	ko04137, ko04138, ko04140, ko04144, ko04145, ko05132, ko05146, ko05152, map04137, map04138, map04140, map04144, map04145, map05132, map05146, map05152
FUN_002535	Belongs to the peptidase S10 family	-	3.4.16.6	ko:K01288	-
FUN_002536	Major Facilitator Superfamily	-	-	-	-
FUN_002538	Alpha-kinase family	-	-	-	-
FUN_002540	spectrin binding	-	-	ko:K15503	-
FUN_002541	Phenol hydroxylase, C-terminal dimerisation domain	-	1.14.13.7	ko:K03380	ko00623, ko00627, ko01120, map00623, map00627, map01120
FUN_002542	GPI biosynthesis protein family Pig-F	GPI11	-	ko:K05287	ko00563, ko01100, map00563, map01100
FUN_002543	Translin family	tsn1	-	ko:K05287	ko00563, ko01100, map00563, map01100
FUN_002546	Dak2	-	2.7.1.28, 2.7.1.29, 4.6.1.15	ko:K00863	ko00051, ko00561, ko00680, ko01100, ko01120, ko01200, ko04622, map00051, map00561, map00680, map01100, map01120, map01200, map04622
FUN_002547	Chitin synthase III catalytic subunit	CHS7	-	-	-
FUN_002548	Domain of unknown function (DUF3425)	-	-	-	-
FUN_002549	Steryl acetyl hydrolase	-	-	-	-
FUN_002552	Flavin-binding monooxygenase-like	-	-	-	-
FUN_002554	Protein of unknown function (DUF4246)	-	-	-	-
FUN_002555	Belongs to the major facilitator superfamily. Sugar transporter (TC 2.A.1.1) family	-	-	-	-
FUN_002557	Guanine deaminase	-	3.5.4.3	ko:K01487	ko00230, ko01100, map00230, map01100

FUN_002559	Belongs to the major facilitator superfamily. Sugar transporter (TC 2.A.1.1) family	-	-	-	-
FUN_002560	Protein of unknown function (DUF1479)	-	-	-	-
FUN_002563	HIRAN	RAD5	-	ko:K15505	-
FUN_002564	Squalene/phytoene synthase	-	-	ko:K18163	ko04714, map04714
FUN_002565	Belongs to the cytochrome P450 family	-	-	-	-
FUN_002567	This protein promotes the GTP-dependent binding of aminoacyl-tRNA to the A-site of ribosomes during protein biosynthesis	TUF1	-	ko:K02358	-
FUN_002568	Belongs to the enoyl-CoA hydratase isomerase family	EHD3	3.1.2.4	ko:K05605	ko00280, ko00410, ko00640, ko01100, ko01200, map00280, map00410, map00640, map01100, map01200
FUN_002569	Vps16, N-terminal region	vps16	-	ko:K20180	ko04138, map04138
FUN_002570	Belongs to the TRAFAC class myosin-kinesin ATPase superfamily. Kinesin family	-	-	-	-
FUN_002571	SpoU rRNA Methylase family	-	2.1.1.34	ko:K15333	-
FUN_002572	Catalyzes the conversion of methylglyoxal (MG) to D- lactate in a single glutathione (GSH)-independent step. May play a role in detoxifying endogenously produced glyoxals. Involved in protection against reactive oxygen species (ROS)	HSP31	4.2.1.130	ko:K22211	ko00620, ko01120, map00620, map01120
FUN_002576	Sugar (and other) transporter	-	-	-	-
FUN_002578	Belongs to the peptidase A1 family	-	-	-	-
FUN_002580	epoxide hydrolase	-	-	-	-
FUN_002582	Putative cyclase	-	-	-	-
FUN_002584	cytochrome P450	-	-	-	-
FUN_002585	G-protein coupled peptide receptor activity	-	-	-	-
FUN_002586	2OG-Fe(II) oxygenase superfamily	-	-	-	-
FUN_002587	Heterokaryon incompatibility protein (HET)	-	-	-	-
FUN_002588	Major Facilitator Superfamily	-	-	-	-
FUN_002589	Phosphoinositide phospholipase C	-	3.1.4.11	ko:K05857	ko00562, ko01100, ko04020, ko04070, ko04919, ko04933, map00562, map01100, map04020, map04070, map04919, map04933
FUN_002594	XPG I-region	-	-	ko:K15339	-
FUN_002595	c6 transcription factor	-	-	-	-
FUN_002598	Iron-containing alcohol dehydrogenase	-	-	-	-
FUN_002599	Aminomethyltransferase folate-binding domain	CAF17	-	ko:K22073	-
FUN_002601	Lung seven transmembrane receptor	PTM1	-	-	-
FUN_002602	Plays a central role in 2-thiolation of mcm(5)S(2)U at tRNA wobble positions of tRNA(Lys), tRNA(Glu) and tRNA(Gln). May act by forming a heterodimer with NCS6 that ligates sulfur from thiocarboxylated URM1 onto the uridine of tRNAs at wobble position. Prior mcm(5) tRNA modification by the elongator complex is required for 2-thiolation. May also be involved in protein urmylation	CTU2	-	ko:K14169	ko04122, map04122
FUN_002603	Domain of unknown function (DUF4440)	-	-	-	-
FUN_002604	Domain of unknown function (DUF4112)	-	-	-	-
FUN_002605	Dcp2, box A domain	DCP2	3.6.1.62	ko:K12613, ko:K22073	ko03018, map03018
FUN_002607	Serine threonine protein kinase	-	2.7.11.1	ko:K08884	-
FUN_002608	Belongs to the glycosyl hydrolase 43 family	abnA	3.2.1.99	ko:K06113	-
FUN_002611	Alpha beta hydrolase	-	-	-	-

FUN_002613	salicylate hydroxylase	-	1.14.13.1	ko:K00480	ko00621, ko00624, ko00626, ko01100, ko01120, ko01220, map00621, map00624, map00626, map01100, map01120, map01220
FUN_002614	extracellular serine-rich protein	-	-	-	-
FUN_002615	UbiA prenyltransferase family	-	-	-	-
FUN_002619	Belongs to the GMC oxidoreductase family	-	-	-	-
FUN_002621	Sugar (and other) transporter	-	-	-	-
FUN_002622	Phytanoyl-CoA dioxygenase (PhyH)	-	-	-	-
FUN_002623	Belongs to the major facilitator superfamily. Sugar transporter (TC 2.A.1.1) family	HXT4	-	-	-
FUN_002624	Alpha/beta hydrolase family	-	-	-	-
FUN_002625	NAD dependent epimerase/dehydratase family	-	-	-	-
FUN_002626	Alpha/beta hydrolase family	-	-	-	-
FUN_002627	NAD(P)H-binding	-	-	-	-
FUN_002628	Isoflavone reductase family protein	-	-	-	-
FUN_002631	Vacuolar ATP synthase subunit S1 (ATP6S1)	-	-	-	-
FUN_002632	R3H domain	FAP1	-	ko:K12236	ko05165, map05165
FUN_002633	Belongs to the LDH MDH superfamily	-	1.1.1.27	ko:K00016	ko00010, ko00270, ko00620, ko00640, ko01100, ko01110, ko01120, ko01130, ko04922, map00010, map00270, map00620, map00640, map01100, map01110, map01120, map01130, map04922
FUN_002635	Belongs to the metallo-dependent hydrolases superfamily. Peptidase M19 family	-	3.4.13.19	ko:K01273	-
FUN_002636	Increased loss of mitochondrial DNA protein 1	-	-	-	-
FUN_002637	Pyridoxal-phosphate dependent enzyme	CHA1	4.3.1.17, 4.3.1.19	ko:K17989	ko00260, ko00270, ko00290, ko01100, ko01110, ko01130, ko01200, ko01230, map00260, map00270, map00290, map01100, map01110, map01130, map01200, map01230
FUN_002638	FAD binding domain	-	-	-	-
FUN_002639	Catalytic component of the COMPASS (Set1C) complex that specifically mono-, di- and trimethylates histone H3 to form H3K4me1 2 3, which subsequently plays a role in telomere length maintenance and transcription elongation regulation	SET1	2.1.1.43	ko:K11422	ko00310, map00310
FUN_002640	Catalyzes the formation of 6, 7-dimethyl-8- ribityllumazine by condensation of 5-amino-6-(D- ribitylamino)uracil with 3, 4-dihydroxy-2-butanone 4-phosphate. This is the penultimate step in the biosynthesis of riboflavin	RIB4	2.5.1.78	ko:K00794	ko00740, ko01100, ko01110, map00740, map01100, map01110
FUN_002641	SIS domain	GFA1	2.6.1.16	ko:K00820	ko00250, ko00520, ko01100, ko01130, ko04931, map00250, map00520, map01100, map01130, map04931
FUN_002642	Transmembrane amino acid transporter protein	-	-	ko:K14209	ko04138, map04138
FUN_002643	Solute carrier family 12	-	-	ko:K14429	-
FUN_002644	WD40 repeats	MAK11	-	ko:K14830	-
FUN_002645	HSA	eaf1	-	ko:K11397	-
FUN_002646	Protein prenyltransferase alpha subunit repeat	RAM2	2.5.1.58, 2.5.1.59	ko:K05955	ko00900, ko01130, map00900, map01130
FUN_002647	Belongs to the short-chain dehydrogenases reductases (SDR) family	-	-	-	-
FUN_002648	Pex2 / Pex12 amino terminal region	PEX10	-	ko:K13346	ko04146, map04146
FUN_002652	EXS family	ERD1	-	-	-
FUN_002654	Lysin motif	-	-	-	-

FUN_002655	chitin catabolic process	-	3.2.1.14	ko:K01183	ko00520, ko01100, map00520, map01100
FUN_002656	Diacylglycerol acyltransferase	DGA1	2.3.1.22	ko:K14457, ko:K14458	ko00561, ko04975, map00561, map04975
FUN_002657	Serine-threonine protein kinase 19	-	-	-	-
FUN_002658	spectrin binding	-	-	-	-
FUN_002661	Alpha/beta hydrolase family	-	-	-	-
FUN_002662	Transporter	-	-	-	-
FUN_002663	Taurine catabolism dioxygenase TauD, TfdA family	-	-	-	-
FUN_002664	Oxidoreductase family, NAD-binding Rossmann fold	-	-	-	-
FUN_002665	Carbohydrate-binding module family 1 protein	-	1.14.99.54	ko:K19356	-
FUN_002668	biotrophy-associated secreted protein 2	-	-	-	-
FUN_002669	Zinc-binding dehydrogenase	-	-	-	-
FUN_002673	Transmembrane adaptor Erv26	SVP26	-	-	-
FUN_002674	Methyltransferase involved in Williams-Beuren syndrome	BUD23	2.1.1.309	ko:K19306	-
FUN_002675	Isocitrate dehydrogenase NAD subunit 1, mitochondrial	IDH1	1.1.1.41	ko:K00030	ko00020, ko01100, ko01110, ko01120, ko01130, ko01200, ko01210, ko01230, map00020, map01100, map01110, map01120, map01130, map01200, map01210, map01230
FUN_002676	NAD-binding of NADP-dependent 3-hydroxyisobutyrate dehydrogenase	-	-	-	-
FUN_002678	Belongs to the eukaryotic ribosomal protein eL38 family	RPL38	-	ko:K02923	ko03010, map03010
FUN_002679	Beta-lactamase superfamily domain	-	-	-	-
FUN_002681	Belongs to the glycosyl hydrolase 47 family	MNS1	3.2.1.113	ko:K01230	ko00510, ko00513, ko01100, ko04141, map00510, map00513, map01100, map04141
FUN_002683	)transporter	-	-	-	-
FUN_002684	integral membrane protein	-	-	-	-
FUN_002686	Phosphatidylethanolamine-binding protein	-	-	-	-
FUN_002687	LCCL domain	-	-	-	-
FUN_002689	Lysine methyltransferase	-	-	-	-
FUN_002690	CobW/HypB/UreG, nucleotide-binding domain	-	-	-	-
FUN_002693	Involved in cytoplasm to vacuole transport (Cvt) and autophagic vesicle formation	atg5	-	ko:K08339	ko04136, ko04137, ko04138, ko04140, ko04211, ko04213, ko04216, ko04621, ko04622, ko05131, map04136, map04137, map04138, map04140, map04211, map04213, map04216, map04621, map04622, map05131
FUN_002694	Component of the post-replicative DNA mismatch repair system (MMR)	MSH3	-	ko:K08736	ko01524, ko03430, ko05200, ko05210, map01524, map03430, map05200, map05210
FUN_002695	WD domain, G-beta repeat	-	-	ko:K10570	ko03420, ko04120, map03420, map04120
FUN_002697	Mnd1 family	-	-	-	-
FUN_002698	Ring finger	-	2.3.2.27	ko:K22381	-
FUN_002699	Velvet factor	velB	-	-	-
FUN_002700	YT521-B-like domain	-	-	-	-
FUN_002701	Serine aminopeptidase, S33	bem46	-	ko:K06889	-
FUN_002702	Proteasome subunit beta type	PRE3	3.4.25.1	ko:K02738	ko03050, map03050
FUN_002703	Cyclin	PCL7	-	-	-
FUN_002704	Prolyl 4-hydroxylase alpha subunit homologues.	-	1.14.11.2	ko:K00472	ko00330, ko01100, map00330, map01100
FUN_002707	Spherulation-specific family 4	-	-	-	-

FUN_002709	Belongs to the DEAD box helicase family	PRP28	3.6.4.13	ko:K12858	ko03040, map03040
FUN_002710	Proteasome subunit beta	PUP3	3.4.25.1	ko:K02735	ko03050, map03050
FUN_002713	Proteasome subunit alpha type	PRE10	3.4.25.1	ko:K02727	ko03050, map03050
FUN_002714	Phosphoglycerate mutase family	-	-	-	-
FUN_002717	integral membrane protein	-	-	-	-
FUN_002718	integral membrane protein	-	-	-	-
FUN_002720	AMP-binding enzyme	-	-	-	-
FUN_002721	Belongs to the GMC oxidoreductase family	-	-	-	-
FUN_002722	Carboxylesterase family	-	-	-	-
FUN_002723	integral membrane protein	-	-	-	-
FUN_002724	Fungal trichothecene efflux pump (TRI12)	-	-	-	-
FUN_002725	Prion-inhibition and propagation	-	-	-	-
FUN_002727	Major Facilitator Superfamily	-	-	-	-
FUN_002728	Pyridine nucleotide-disulphide oxidoreductase	-	-	-	-
FUN_002730	Belongs to the major facilitator superfamily. Sugar transporter (TC 2.A.1.1) family	-	-	-	-
FUN_002731	Belongs to the DapA family	-	4.1.2.53, 4.1.3.16	ko:K18123, ko:K18339	ko00051, ko00330, ko00630, ko01100, ko01120, map00051, map00330, map00630, map01100, map01120
FUN_002733	Cytochrome P450	-	-	-	-
FUN_002734	Ankyrin repeats (many copies)	-	-	-	-
FUN_002735	acid phosphatase	-	-	ko:K22390	-
FUN_002736	Belongs to the major facilitator superfamily. Sugar transporter (TC 2.A.1.1) family	-	-	ko:K08150	-
FUN_002737	cytochrome P450	-	-	-	-
FUN_002738	FAD binding domain	-	1.6.2.4	ko:K00327	-
FUN_002739	zn 2cys6 transcription factor	-	-	-	-
FUN_002740	Oxidoreductase family, C-terminal alpha/beta domain	-	-	-	-
FUN_002741	Fungal specific transcription factor domain	-	-	-	-
FUN_002744	Gelsolin repeat-containing protein	-	-	ko:K05768	ko04666, ko04810, ko05203, map04666, map04810, map05203
FUN_002745	Major Facilitator Superfamily	-	-	-	-
FUN_002748	AMP-binding enzyme	-	-	-	-
FUN_002752	Glycoside hydrolase family 10 protein	-	3.2.1.8	ko:K01181	-
FUN_002753	integral membrane protein	-	-	-	-
FUN_002754	Alpha/beta hydrolase family	-	-	-	-
FUN_002755	Belongs to the GMC oxidoreductase family	-	-	-	-
FUN_002757	Cyclohexanone monooxygenase	-	1.14.13.22	ko:K03379	ko00930, ko01120, ko01220, map00930, map01120, map01220
FUN_002758	Belongs to the Ca(2) cation antiporter (CaCA) (TC 2.A.19) family	-	-	ko:K07300	-
FUN_002759	Sugar (and other) transporter	-	-	-	-
FUN_002763	N-acetyltransferase	-	-	-	-
FUN_002764	NMT1/THI5 like	-	-	-	-
FUN_002768	FAD linked oxidases, C-terminal domain	-	-	-	-
FUN_002772	Peroxidase, family 2	-	1.11.2.1	ko:K21820	-
FUN_002774	Glycoside hydrolase family 2 protein	-	3.2.1.23	ko:K01190	ko00052, ko00511, ko00600, ko01100, map00052, map00511, map00600, map01100

FUN_002775	Belongs to the major facilitator superfamily. Sugar transporter (TC 2.A.1.1) family	-	-	-	-
FUN_002776	Amino acid permease	-	-	ko:K16261	-
FUN_002778	D-xylose 1-dehydrogenase (NADP+) activity	-	-	-	-
FUN_002779	Est1 DNA/RNA binding domain	-	-	-	-
FUN_002781	Protein of unknown function (DUF3638)	-	-	-	-
FUN_002782	exonucleolytic nuclear-transcribed mRNA catabolic process involved in deadenylation-dependent decay	-	-	-	-
FUN_002783	Alpha beta hydrolase fold-3 domain-containing protein	-	-	-	-
FUN_002784	DNA ligase	-	6.5.1.1	ko:K10777	ko03450, map03450
FUN_002786	Cyclin	PCL1	-	ko:K06656, ko:K06657	ko04111, map04111
FUN_002787	methyltransferase	-	-	-	-
FUN_002788	RNA dependent RNA polymerase	-	2.7.7.48	ko:K11699	-
FUN_002791	Carbohydrate/starch-binding module (family 21)	-	-	-	-
FUN_002792	Belongs to the TRAFAC class dynamin-like GTPase superfamily. Dynamin Fzo YdjA family	-	-	-	-
FUN_002793	Alpha beta hydrolase fold-3 domain-containing protein	-	-	-	-
FUN_002795	Belongs to the TRAFAC class dynamin-like GTPase superfamily. Dynamin Fzo YdjA family	-	-	-	-
FUN_002796	Zinc finger, C2H2 type	AZF1	-	-	-
FUN_002807	Cryptococcal mannosyltransferase 1	-	-	-	-
FUN_002810	A domain family that is part of the cupin metalloenzyme superfamily.	-	-	-	-
FUN_002814	domain) protein	-	-	-	-
FUN_002817	von Willebrand factor (vWF) type A domain	-	-	-	-
FUN_002820	von Willebrand factor (vWF) type A domain	-	-	-	-
FUN_002821	ABC transporter transmembrane region	-	-	-	-
FUN_002823	Serine aminopeptidase, S33	-	3.1.1.23	ko:K13704	-
FUN_002824	Protein of unknown function (DUF3638)	-	-	-	-
FUN_002825	Est1 DNA/RNA binding domain	-	-	-	-
FUN_002830	Belongs to the glycosyl hydrolase 47 family	-	3.2.1.113	ko:K01230	ko00510, ko00513, ko01100, ko04141, map00510, map00513, map01100, map04141
FUN_002833	DDE superfamily endonuclease	-	-	-	-
FUN_002834	Belongs to the glycosyl hydrolase 47 family	-	3.2.1.113	ko:K01230	ko00510, ko00513, ko01100, ko04141, map00510, map00513, map01100, map04141
FUN_002836	Ankyrin repeat	-	-	-	-
FUN_002846	von Willebrand factor (vWF) type A domain	-	-	-	-
FUN_002847	Belongs to the Ca(2) cation antiporter (CaCA) (TC 2.A.19) family	-	-	ko:K07300	-
FUN_002848	Uracil phosphoribosyltransferase	-	-	-	-
FUN_002849	FtsJ-like methyltransferase	-	-	-	-
FUN_002853	Belongs to the iron ascorbate-dependent oxidoreductase family	-	-	-	-
FUN_002855	Encoded by	-	-	-	-
FUN_002856	C6 zinc finger domain-containing protein	-	-	-	-
FUN_002857	von Willebrand factor (vWF) type A domain	-	-	-	-
FUN_002858	domain-containing protein	-	-	-	-

FUN_002864	Serine threonine-protein kinase sgk2	-	-	-	-
FUN_002865	Domain present in PSD-95, Dlg, and ZO-1/2.	NMA111	-	-	-
FUN_002870	Belongs to the iron ascorbate-dependent oxidoreductase family	-	-	-	-
FUN_002871	domain) protein	-	-	-	-
FUN_002872	Serine aminopeptidase, S33	-	3.1.1.23	ko:K13704	-
FUN_002875	Tetratricopeptide repeat	-	-	-	-
FUN_002877	C6 zinc finger domain-containing protein	-	-	-	-
FUN_002884	nonribosomal peptide	-	-	ko:K22152	-
FUN_002887	Belongs to the glycosyl hydrolase 47 family	-	3.2.1.113	ko:K01230	ko00510, ko00513, ko01100, ko04141, map00510, map00513, map01100, map04141
FUN_002888	domain) protein	-	-	-	-
FUN_002890	NACHT domain	-	-	-	-
FUN_002897	von Willebrand factor (vWF) type A domain	-	-	-	-
FUN_002898	Protein of unknown function (DUF3645)	-	-	-	-
FUN_002899	Belongs to the glycosyl hydrolase 47 family	-	3.2.1.113	ko:K01230	ko00510, ko00513, ko01100, ko04141, map00510, map00513, map01100, map04141
FUN_002900	Belongs to the glycosyl hydrolase 47 family	-	3.2.1.113	ko:K01230	ko00510, ko00513, ko01100, ko04141, map00510, map00513, map01100, map04141
FUN_002905	Kinetochore CENP-C fungal homologue, Mif2, N-terminal	MIF2	-	ko:K11497	-
FUN_002909	GPI-anchored cell wall beta-1, 3-endoglucanase EglC	-	3.2.1.39	ko:K01199	ko00500, map00500
FUN_002910	HPC2 and ubinuclein domain	HPC2	-	-	-
FUN_002911	DNA ligase N terminus	-	6.5.1.1	ko:K10777	ko03450, map03450
FUN_002912	Tetratricopeptide repeat	-	-	-	-
FUN_002913	Encoded by	-	-	-	-
FUN_002915	Domain present in PSD-95, Dlg, and ZO-1/2.	NMA111	-	-	-
FUN_002916	Pro-apoptotic serine protease NMA111	NMA111	-	-	-
FUN_002917	NB-ARC domain	-	-	-	-
FUN_002918	Reverse transcriptase (RNA-dependent DNA polymerase)	-	-	-	-
FUN_002919	GPI-anchored cell wall beta-1, 3-endoglucanase EglC	-	3.2.1.39	ko:K01199	ko00500, map00500
FUN_002921	Cyclin	PCL1	-	ko:K06656, ko:K06657	ko04111, map04111
FUN_002922	von Willebrand factor (vWF) type A domain	-	-	-	-
FUN_002926	Est1 DNA/RNA binding domain	-	-	-	-
FUN_002927	domain) protein	-	-	-	-
FUN_002931	to PDZ domain protein	NMA111	-	-	-
FUN_002935	NACHT domain	-	-	-	-
FUN_002936	Belongs to the TRAFAC class dynamin-like GTPase superfamily. Dynamin Fzo YdjA family	-	-	-	-
FUN_002937	GPI-anchored cell wall beta-1, 3-endoglucanase EglC	-	3.2.1.39	ko:K01199	ko00500, map00500
FUN_002940	WD domain, G-beta repeat	-	-	-	-
FUN_002941	Tetratricopeptide repeat	-	-	-	-
FUN_002942	phosphatidylglycerol acyl-chain remodeling	-	-	-	-
FUN_002943	domain protein	-	-	-	-

FUN_002948	Catalyzes conversion of folates to polyglutamate derivatives allowing concentration of folate compounds in the cell and the intracellular retention of these cofactors, which are important substrates for most of the folate-dependent enzymes that are involved in one-carbon transfer reactions involved in purine, pyrimidine and amino acid synthesis	MET7	6.3.2.17	ko:K01930	ko00790, ko01100, ko01523, map00790, map01100, map01523
FUN_002949	FabD lysophospholipase-like protein	-	-	-	-
FUN_002950	Belongs to the TRAFAC class dynamin-like GTPase superfamily. Dynamin Fzo YdjA family	-	-	-	-
FUN_002953	kinesin light chain	-	-	-	-
FUN_002955	DDE superfamily endonuclease	-	-	-	-
FUN_002956	Mechanosensitive ion channel	-	-	ko:K22048	-
FUN_002957	domain) protein	-	-	-	-
FUN_002959	Encoded by	-	-	-	-
FUN_002962	Cyclin	PCL1	-	ko:K06656, ko:K06657	ko04111, map04111
FUN_002964	Belongs to the glycosyl hydrolase 47 family	-	3.2.1.113	ko:K01230	ko00510, ko00513, ko01100, ko04141, map00510, map00513, map01100, map04141
FUN_002968	DDE superfamily endonuclease	-	-	-	-
FUN_002972	Phosphorylase superfamily	-	-	-	-
FUN_002973	Fungal trichothecene efflux pump (TRI12)	-	-	-	-
FUN_002974	nerve growth factor signaling pathway	-	-	-	-
FUN_002979	ABC transporter transmembrane region	-	-	-	-
FUN_002981	Conserved glycine-rich protein	-	-	-	-
FUN_002983	NB-ARC domain	-	-	-	-
FUN_002985	cytochrome P450	-	-	-	-
FUN_002986	Fungal trichothecene efflux pump (TRI12)	-	-	-	-
FUN_002987	Belongs to the SKP1 family	sconC	-	ko:K03094	ko04110, ko04111, ko04114, ko04120, ko04141, ko04310, ko04341, ko04350, ko04710, ko05168, ko05200, map04110, map04111, map04114, map04120, map04141, map04310, map04341, map04350, map04710, map05168, map05200
FUN_002991	DNA ligase	-	6.5.1.1	ko:K10777	ko03450, map03450
FUN_002992	DDE superfamily endonuclease	-	-	-	-
FUN_002993	NB-ARC domain	-	-	-	-
FUN_002998	C6 zinc finger domain-containing protein	-	-	-	-
FUN_002999	Cryptococcal mannosyltransferase 1	-	-	-	-
FUN_003000	Belongs to the iron ascorbate-dependent oxidoreductase family	-	-	-	-
FUN_003002	Domain present in PSD-95, Dlg, and ZO-1/2.	NMA111	-	-	-
FUN_003004	Ubiquitin homologues	SMT3	-	ko:K12160	ko03013, ko05418, map03013, map05418
FUN_003005	Belongs to the SKP1 family	sconC	-	ko:K03094	ko04110, ko04111, ko04114, ko04120, ko04141, ko04310, ko04341, ko04350, ko04710, ko05168, ko05200, map04110, map04111, map04114, map04120, map04141, map04310, map04341, map04350, map04710, map05168, map05200
FUN_003008	Fungal trichothecene efflux pump (TRI12)	-	-	-	-
FUN_003009	Cysteine-rich motif following a subset of SET domains	-	2.1.1.43, 2.3.1.225	ko:K06101, ko:K16675	ko00310, ko04391, map00310, map04391

FUN_003010	Encoded by	-	-	-	-
FUN_003011	Est1 DNA/RNA binding domain	-	-	-	-
FUN_003013	NACHT domain	-	-	-	-
FUN_003015	DNA ligase N terminus	-	6.5.1.1	ko:K10777	ko03450, map03450
FUN_003018	HPC2 and ubinuclein domain	HPC2	-	-	-
FUN_003019	Fungal trichothecene efflux pump (TRI12)	-	-	-	-
FUN_003022	nonribosomal peptide	-	-	ko:K22152	-
FUN_003023	Zinc finger, C2H2 type	AZF1	-	-	-
FUN_003024	Heterokaryon incompatibility protein (HET)	-	-	-	-
FUN_003025	spectrin binding	-	-	-	-
FUN_003026	DNA ligase	-	6.5.1.1	ko:K10777	ko03450, map03450
FUN_003029	Belongs to the glycosyl hydrolase 47 family	-	3.2.1.113	ko:K01230	ko00510, ko00513, ko01100, ko04141, map00510, map00513, map01100, map04141
FUN_003031	Vegetative incompatibility protein het-e-1	-	-	-	-
FUN_003032	kinesin light chain	-	-	-	-
FUN_003034	RNA dependent RNA polymerase	-	2.7.7.48	ko:K11699	-
FUN_003037	Tetratricopeptide repeat	-	-	-	-
FUN_003038	Conserved glycine-rich protein	-	-	-	-
FUN_003039	Ubiquitin-activating enzyme	UBA2	6.2.1.45	ko:K10685	ko04120, map04120
FUN_003040	Belongs to the glycosyl hydrolase 47 family	-	3.2.1.113	ko:K01230	ko00510, ko00513, ko01100, ko04141, map00510, map00513, map01100, map04141
FUN_003041	ABC transporter, transmembrane region	-	-	ko:K06147	-
FUN_003042	ABC transporter	-	-	-	-
FUN_003049	incompatibility protein	-	-	-	-
FUN_003051	Cryptococcal mannosyltransferase 1	-	-	-	-
FUN_003052	HPC2 and ubinuclein domain	HPC2	-	-	-
FUN_003053	Ankyrin repeat protein	-	-	-	-
FUN_003056	N-terminal domain of NWD NACHT-NTPase	-	-	-	-
FUN_003057	Belongs to the SKP1 family	sconC	-	ko:K03094	ko04110, ko04111, ko04114, ko04120, ko04141, ko04310, ko04341, ko04350, ko04710, ko05168, ko05200, map04110, map04111, map04114, map04120, map04141, map04310, map04341, map04350, map04710, map05168, map05200
FUN_003058	Est1 DNA/RNA binding domain	-	-	-	-
FUN_003059	Tetratricopeptide repeat	-	-	-	-
FUN_003060	thiol-dependent ubiquitin-specific protease activity	-	-	-	-
FUN_003061	Middle or third domain of peptidase_M16	STE23	3.4.24.56	ko:K01408	ko05010, map05010
FUN_003062	Protein of unknown function (DUF3431)	-	-	-	-
FUN_003067	Fungal trichothecene efflux pump (TRI12)	-	-	-	-
FUN_003069	Tetratricopeptide repeat	-	-	-	-
FUN_003071	O-methyltransferase	-	-	-	-
FUN_003074	Belongs to the argonaute family	-	-	ko:K11593	-
FUN_003077	Isochorismatase family	-	-	-	-
FUN_003081	Mechanosensitive ion channel	-	-	ko:K22048	-
FUN_003083	Domain present in PSD-95, Dlg, and ZO-1/2.	NMA111	-	-	-

FUN_003084	Phosphorylase superfamily	-	-	-	-
FUN_003086	A domain family that is part of the cupin metalloenzyme superfamily.	-	-	-	-
FUN_003089	Belongs to the SKP1 family	sconC	-	ko:K03094	ko04110, ko04111, ko04114, ko04120, ko04141, ko04310, ko04341, ko04350, ko04710, ko05168, ko05200, map04110, map04111, map04114, map04120, map04141, map04310, map04341, map04350, map04710, map05168, map05200
FUN_003091	Kinetochore CENP-C fungal homologue, Mif2, N-terminal	MIF2	-	ko:K11497	-
FUN_003093	Alpha beta hydrolase fold-3 domain-containing protein	-	-	-	-
FUN_003094	Ankyrin repeat	-	-	-	-
FUN_003095	Adenosine/AMP deaminase	-	3.5.4.4	ko:K19572	ko00230, ko01100, map00230, map01100
FUN_003099	Taurine catabolism dioxygenase TauD, TfdA family	-	-	-	-
FUN_003100	Belongs to the TRAFAC class dynamin-like GTPase superfamily. Dynamin Fzo YdjA family	-	-	-	-
FUN_003101	vegetative cell wall protein gp1	-	-	-	-
FUN_003103	Protein of unknown function (DUF3455)	-	-	-	-
FUN_003107	Alpha beta hydrolase fold-3 domain-containing protein	-	-	-	-
FUN_003108	In Between Ring fingers	-	-	-	-
FUN_003110	O-methyltransferase	-	-	-	-
FUN_003111	domain) protein	-	-	-	-
FUN_003112	domain) protein	-	-	-	-
FUN_003113	Belongs to the TRAFAC class dynamin-like GTPase superfamily. Dynamin Fzo YdjA family	-	-	-	-
FUN_003114	Ankyrin repeat protein	-	-	-	-
FUN_003115	HPC2 and ubinuclein domain	HPC2	-	-	-
FUN_003116	von Willebrand factor (vWF) type A domain	-	-	-	-
FUN_003123	Cyclin	PCL1	-	ko:K06656, ko:K06657	ko04111, map04111
FUN_003124	Catalyzes conversion of folates to polyglutamate derivatives allowing concentration of folate compounds in the cell and the intracellular retention of these cofactors, which are important substrates for most of the folate-dependent enzymes that are involved in one-carbon transfer reactions involved in purine, pyrimidine and amino acid synthesis	MET7	6.3.2.17	ko:K01930	ko00790, ko01100, ko01523, map00790, map01100, map01523
FUN_003126	O-methyltransferase	-	-	-	-
FUN_003127	exonucleolytic nuclear-transcribed mRNA catabolic process involved in deadenylation-dependent decay	-	-	-	-
FUN_003134	Dyp-type peroxidase family	-	-	ko:K07223	-
FUN_003135	-o-)-methyltransferase	-	-	-	-
FUN_003136	Major Facilitator Superfamily	-	-	-	-
FUN_003137	integral membrane protein	-	-	-	-
FUN_003138	Endoribonuclease L-PSP	-	-	-	-
FUN_003139	Flavin containing amine oxidoreductase	-	-	-	-
FUN_003140	Involved in maceration and soft-rotting of plant tissue	-	3.1.1.11, 3.2.1.15	ko:K01051, ko:K01184	ko00040, ko01100, map00040, map01100

FUN_003144	Amidase	-	3.5.1.4	ko:K01426	ko00330, ko00360, ko00380, ko00627, ko00643, ko01120, map00330, map00360, map00380, map00627, map00643, map01120
FUN_003146	P-loop containing nucleoside triphosphate hydrolase protein	-	-	-	-
FUN_003147	Catalyzes the reversible phosphorylation of S-methyl-5'- thioadenosine (MTA) to adenine and 5-methylthioribose-1-phosphate. Involved in the breakdown of MTA, a major by-product of polyamine biosynthesis. Responsible for the first step in the methionine salvage pathway after MTA has been generated from S-adenosylmethionine. Has broad substrate specificity with 6- aminopurine nucleosides as	MEU1	2.4.2.28	ko:K00772	ko00270, ko01100, map00270, map01100
FUN_003150	NAD(P)H-binding	-	-	-	-
FUN_003151	Pyridine nucleotide-disulphide oxidoreductase	-	-	-	-
FUN_003153	WD domain, G-beta repeat	-	-	-	-
FUN_003154	Flavin-binding monooxygenase-like	-	-	-	-
FUN_003155	Major Facilitator Superfamily	-	-	-	-
FUN_003156	FAD binding domain-containing protein	-	-	-	-
FUN_003157	Belongs to the cytochrome P450 family	-	-	-	-
FUN_003159	short chain dehydrogenase reductase	-	-	-	-
FUN_003160	Belongs to the type-B carboxylesterase lipase family	-	-	-	-
FUN_003162	Glycoside hydrolase family 16	-	-	-	-
FUN_003163	Apoptosis antagonizing transcription factor	BFR2	-	ko:K14782	-
FUN_003164	ATPase MipZ	-	-	ko:K03593	-
FUN_003168	Steryl acetyl hydrolase	-	-	-	-
FUN_003169	Heterokaryon incompatibility protein (HET)	-	-	-	-
FUN_003171	Glycoside hydrolase family 13	-	3.2.1.1	ko:K01176	ko00500, ko01100, ko04973, map00500, map01100, map04973
FUN_003172	microtubule motor activity	-	-	ko:K10393	-
FUN_003173	Heterokaryon incompatibility protein (HET)	-	-	-	-
FUN_003174	Flavoprotein (FP) subunit of succinate dehydrogenase (SDH) that is involved in complex II of the mitochondrial electron transport chain and is responsible for transferring electrons from succinate to ubiquinone (coenzyme Q)	-	1.3.5.1	ko:K00234	ko00020, ko00190, ko01100, ko01110, ko01120, ko01130, ko01200, ko04714, ko04932, ko05010, ko05012, ko05016, map00020, map00190, map01100, map01110, map01120, map01130, map01200, map04714, map04932, map05010, map05012, map05016
FUN_003176	Belongs to the D-isomer specific 2-hydroxyacid dehydrogenase family	-	1.1.1.26	ko:K00015	ko00630, ko01100, ko01110, ko01120, map00630, map01100, map01110, map01120
FUN_003177	FAD dependent oxidoreductase	-	-	-	-
FUN_003178	Sugar (and other) transporter	-	-	-	-
FUN_003179	Multicopper oxidase	-	-	-	-
FUN_003180	Epoxide hydrolase N terminus	-	-	-	-
FUN_003182	Glycoside-hydrolase family GH114	-	-	-	-
FUN_003183	RTA1 like protein	-	-	-	-
FUN_003184	ABC-2 type transporter	-	-	-	-
FUN_003185	Iron-containing alcohol dehydrogenase	-	1.1.99.24	ko:K11173	-
FUN_003187	cyclin-dependent protein serine/threonine kinase activity	-	2.7.11.22, 2.7.11.23	ko:K02208	-
FUN_003188	Pleckstrin homology domain.	-	-	-	-

FUN_003189	Cupin superfamily (DUF985)	-	-	ko:K09705	-
FUN_003190	Activator of mitotic machinery Cdc14 phosphatase activation C-term	-	-	ko:K18626	-
FUN_003191	Mitochondrial GTPase involved in mitochondrial trafficking	GEM1	-	ko:K07870	ko04137, ko04214, map04137, map04214
FUN_003192	WD40 repeats	creC	-	-	-
FUN_003193	Arrestin (or S-antigen), C-terminal domain	creD	-	ko:K20062	-
FUN_003194	Glycoside hydrolase family 18 protein	CDC28	2.7.11.22	ko:K04563	ko04011, ko04111, ko04113, map04011, map04111, map04113
FUN_003195	Glycoside hydrolase family 18 protein	CDC28	2.7.11.22	ko:K04563	ko04011, ko04111, ko04113, map04011, map04111, map04113
FUN_003196	2OG-Fe(II) oxygenase superfamily	-	3.5.1.4, 4.2.99.18	ko:K01426, ko:K10765	ko00330, ko00360, ko00380, ko00627, ko00643, ko01120, map00330, map00360, map00380, map00627, map00643, map01120
FUN_003197	Family of unknown function (DUF572)	saf4	-	ko:K13115	-
FUN_003198	Elongation factor Tu C-terminal domain	SUP35	-	ko:K03267	ko03015, map03015
FUN_003199	RanGTP-binding protein	YRB30	-	-	-
FUN_003200	domain in transcription factors and synapse-associated proteins	-	-	-	-
FUN_003201	Pre-rRNA-processing protein TSR2	TSR2	-	ko:K14800	-
FUN_003203	Belongs to the multicopper oxidase family	-	-	ko:K19791	-
FUN_003204	SET (Su(var)3-9, Enhancer-of-zeste, Trithorax) domain	-	-	-	-
FUN_003206	Major Facilitator Superfamily	-	-	-	-
FUN_003207	KR domain	-	-	-	-
FUN_003208	Fungal specific transcription factor domain	-	-	-	-
FUN_003209	Fructose-bisphosphate aldolase class-II	-	4.1.2.13	ko:K01624	ko00010, ko00030, ko00051, ko00680, ko00710, ko01100, ko01110, ko01120, ko01130, ko01200, ko01230, map00010, map00030, map00051, map00680, map00710, map01100, map01110, map01120, map01130, map01200, map01230
FUN_003211	Belongs to the glycosyl hydrolase 47 family	-	3.2.1.113	ko:K01230	ko00510, ko00513, ko01100, ko04141, map00510, map00513, map01100, map04141
FUN_003212	pyridoxamine phosphate oxidase family protein	-	-	-	-
FUN_003213	Fungal specific transcription factor domain	-	-	-	-
FUN_003214	Broad-Complex, Tramtrack and Bric a brac	-	-	-	-
FUN_003216	Belongs to the major facilitator superfamily. Sugar transporter (TC 2.A.1.1) family	-	-	-	-
FUN_003218	BUD22	-	1.6.5.3, 1.6.99.3	ko:K03936	ko00190, ko01100, ko04714, ko04723, ko04932, ko05010, ko05012, ko05016, map00190, map01100, map04714, map04723, map04932, map05010, map05012, map05016
FUN_003219	Belongs to the thiolase family	-	2.3.1.176	ko:K08764	ko00120, ko01100, ko03320, ko04146, map00120, map01100, map03320, map04146
FUN_003221	Protein of unknown function (DUF1698)	-	-	-	-
FUN_003222	Copper amine oxidase, N2 domain	-	1.4.3.21	ko:K00276	ko00260, ko00350, ko00360, ko00410, ko00950, ko00960, ko01100, ko01110, map00260, map00350, map00360, map00410, map00950, map00960, map01100, map01110
FUN_003223	Ferroportin1 (FPN1)	-	-	ko:K14685	ko04216, ko04978, map04216, map04978
FUN_003224	Belongs to the glycosyl hydrolase 7 (cellulase C) family	-	3.2.1.91	ko:K01225	ko00500, ko01100, map00500, map01100
FUN_003225	Belongs to the iron ascorbate-dependent oxidoreductase family	-	-	-	-
FUN_003226	Belongs to the GMC oxidoreductase family	-	1.1.99.18	ko:K19069	-

FUN_003227	Protein of unknown function (DUF2433)	-	-	-	-
FUN_003228	Importin-beta N-terminal domain	kap123	-	ko:K20221	-
FUN_003229	Niemann-Pick C1 N terminus	NCR1	-	ko:K12385	ko04142, ko04979, map04142, map04979
FUN_003231	Alcohol dehydrogenase GroES-like domain	-	-	-	-
FUN_003242	Late endosomal/lysosomal adaptor and MAPK and MTOR activator	-	-	-	-
FUN_003244	ABC transporter transmembrane region	-	-	-	-
FUN_003246	Belongs to the cytochrome P450 family	-	-	-	-
FUN_003259	coreceptor activity involved in Wnt signaling pathway, planar cell polarity pathway	-	-	-	-
FUN_003261	EthD domain	-	-	-	-
FUN_003263	Arylsulfotransferase (ASST)	-	-	-	-
FUN_003264	Domain of unknown function (DUF1772)	-	-	-	-
FUN_003265	Splits internally a 1, 3-beta-glucan molecule and transfers the newly generated reducing end (the donor) to the non- reducing end of another 1, 3-beta-glucan molecule (the acceptor) forming a 1, 3-beta linkage, resulting in the elongation of 1, 3- beta-glucan chains in the cell wall	gel4	-	-	-
FUN_003266	Bacterial alpha-L-rhamnosidase 6 hairpin glycosidase domain	-	-	-	-
FUN_003268	Nucleotide-diphospho-sugar transferase	-	-	-	-
FUN_003269	Fungal potassium channel	KCH1	-	-	-
FUN_003271	Belongs to the heat shock protein 70 family	KAR2	-	ko:K09490	ko03060, ko04141, ko04918, ko05020, map03060, map04141, map04918, map05020
FUN_003272	NADH:flavin oxidoreductase / NADH oxidase family	OYE32	1.3.1.42	ko:K05894	ko00592, ko01100, ko01110, map00592, map01100, map01110
FUN_003274	Fungal trichothecene efflux pump (TRI12)	-	-	-	-
FUN_003276	Belongs to the glycosyl hydrolase 47 family	-	3.2.1.113	ko:K01230	ko00510, ko00513, ko01100, ko04141, map00510, map00513, map01100, map04141
FUN_003277	Methylates the 2'-O-ribose of nucleotides at positions 32 and 34 of the tRNA anticodon loop of substrate tRNAs	TRM7	2.1.1.205	ko:K14864	-
FUN_003278	helicase superfamily c-terminal domain	MAK5	3.6.4.13	ko:K14805	-
FUN_003279	Gpi16 subunit, GPI transamidase component	GPI16	-	ko:K05292	ko00563, ko01100, map00563, map01100
FUN_003280	LUC7 N_terminus	LUC7	-	-	-
FUN_003281	Belongs to the mitochondrial carrier (TC 2.A.29) family	-	-	ko:K15104	-
FUN_003282	Glycoside hydrolase family 92 protein	-	-	-	-
FUN_003287	F-actin-capping proteins bind in a Ca(2)-independent manner to the fast growing ends of actin filaments (barbed end) thereby blocking the exchange of subunits at these ends. Unlike other capping proteins (such as gelsolin and severin), these proteins do not sever actin filaments	CAP1	-	ko:K10364	ko04144, map04144
FUN_003290	VHS domain	-	-	ko:K20524	-
FUN_003292	Methyltransferase domain	-	-	-	-
FUN_003293	K homology RNA-binding domain	SCP160	-	-	-
FUN_003294	GAT domain	HSE1	-	ko:K04705	ko04144, ko04630, map04144, map04630
FUN_003295	P21-Rho-binding domain	STE20	2.7.11.1	ko:K04409	ko04010, ko04011, ko04012, ko04014, ko04024, ko04062, ko04360, ko04392, ko04510, ko04650, ko04660, ko04666, ko04810, ko05120, ko05205, ko05211, map04010, map04011, map04012, map04014, map04024, map04062, map04360, map04392, map04510, map04650, map04660, map04666, map04810, map05120, map05205, map05211

FUN_003296	PAS fold	-	-	-	-
FUN_003297	Flavoprotein	-	-	-	-
FUN_003298	Belongs to the cytochrome b5 family	-	-	-	-
FUN_003299	NUC153 domain	ENP2	-	ko:K14788	-
FUN_003300	Component of the SRB8-11 complex. The SRB8-11 complex is a regulatory module of the Mediator complex which is itself involved in regulation of basal and activated RNA polymerase II- dependent transcription. The SRB8-11 complex may be involved in the transcriptional repression of a subset of genes regulated by Mediator. It may inhibit the association of the Mediator complex with RNA polymerase II to form the holoenzyme complex	SSN2	-	ko:K15165	-
FUN_003301	Belongs to the DHHC palmitoyltransferase family	SWF1	2.3.1.225	ko:K20003	-
FUN_003302	Belongs to the DHHC palmitoyltransferase family	SWF1	2.3.1.225	ko:K20003	-
FUN_003303	TRF2-interacting telomeric protein/Rap1 - C terminal domain	-	-	ko:K11113	-
FUN_003304	Belongs to the oxygen-dependent FAD-linked oxidoreductase family	-	-	-	-
FUN_003305	Belongs to the cytochrome P450 family	-	1.14.14.23	ko:K00489	ko00120, ko00140, ko01100, ko03320, ko04976, ko04979, map00120, map00140, map01100, map03320, map04976, map04979
FUN_003308	Acetyltransferase (GNAT) family	-	-	-	-
FUN_003309	Domain of unknown function (DUF3471)	-	-	-	-
FUN_003311	Belongs to the peptidase S8 family	alp	3.4.21.63	ko:K18549	-
FUN_003312	Major Facilitator Superfamily	-	-	-	-
FUN_003314	Mo-co oxidoreductase dimerisation domain	-	1.8.3.1	ko:K00387	ko00920, ko01100, ko01120, map00920, map01100, map01120
FUN_003318	ethyl tert-butyl ether degradation	-	-	-	-
FUN_003319	Carbohydrate esterase family 1 protein	-	-	-	-
FUN_003320	Belongs to the glycosyl hydrolase 30 family	-	-	-	-
FUN_003323	Belongs to the iron ascorbate-dependent oxidoreductase family	-	-	-	-
FUN_003326	Belongs to the cytochrome P450 family	-	1.14.14.23	ko:K00489	ko00120, ko00140, ko01100, ko03320, ko04976, ko04979, map00120, map00140, map01100, map03320, map04976, map04979
FUN_003327	Belongs to the tannase family	-	-	-	-
FUN_003328	bnr asp-box repeat	-	-	-	-
FUN_003330	Putative threonine/serine exporter	-	-	-	-
FUN_003331	FAD binding domain	-	-	-	-
FUN_003333	Belongs to the major facilitator superfamily. Sugar transporter (TC 2.A.1.1) family	HXT5	-	ko:K08139	ko04113, map04113
FUN_003335	Major Facilitator Superfamily	-	-	-	-
FUN_003336	Chromo shadow domain	-	-	ko:K11585	-
FUN_003337	assists the folding of proteins upon ATP hydrolysis	CCT5	-	ko:K09497	-
FUN_003338	CHY zinc finger	-	-	-	-
FUN_003339	Protein of unknown function (DUF3295)	-	-	-	-
FUN_003340	Aldo/keto reductase family	-	-	-	-
FUN_003341	Fungal specific transcription factor domain	-	-	-	-
FUN_003342	Heterokaryon incompatibility protein (HET)	-	-	-	-
FUN_003343	Asparaginase	-	3.4.19.5, 3.5.1.1	ko:K01424, ko:K13051	ko00250, ko00460, ko01100, ko01110, map00250, map00460, map01100, map01110

FUN_003344	Amidase	-	3.5.1.4	ko:K01426	ko00330, ko00360, ko00380, ko00627, ko00643, ko01120, map00330, map00360, map00380, map00627, map00643, map01120
FUN_003346	TORC1 subunit TCO89	-	-	-	-
FUN_003348	Las17-binding protein actin regulator	-	-	ko:K20523	-
FUN_003350	ABC transporter	-	-	ko:K06158	-
FUN_003351	(ABC) transporter	-	-	-	-
FUN_003352	NAD dependent epimerase/dehydratase family	-	-	-	-
FUN_003353	NACHT domain	-	-	-	-
FUN_003354	Cellulose-growth-specific protein	-	-	-	-
FUN_003357	fumarate reductase	-	-	ko:K18561	-
FUN_003358	Protein of unknown function (DUF1769)	-	-	-	-
FUN_003359	Alpha/beta hydrolase family	-	-	-	-
FUN_003360	to Saccharomyces cerevisiae GFD2 (YCL036W) and YDR514C	-	-	-	-
FUN_003361	Belongs to the MIP aquaporin (TC 1.A.8) family	-	-	ko:K03441	-
FUN_003363	Alpha/beta hydrolase of unknown function (DUF1100)	-	-	-	-
FUN_003367	Belongs to the peptidase S10 family	-	-	-	-
FUN_003369	alpha/beta hydrolase fold	-	-	-	-
FUN_003370	Glycosyltransferase sugar-binding region containing DXD motif	-	-	ko:K11806	-
FUN_003372	Component of the ESCRT-0 complex which is the sorting receptor for ubiquitinated cargo proteins at the multivesicular body (MVB) and recruits ESCRT-I to the MVB outer membrane	VPS27	-	ko:K12182	ko04144, ko04145, map04144, map04145
FUN_003373	Ubiquitin carboxyl-terminal hydrolase	DOA4	3.4.19.12	ko:K11839	ko04137, ko04144, ko04934, map04137, map04144, map04934
FUN_003374	Arginosuccinate synthase	ARG1	6.3.4.5	ko:K01940	ko00220, ko00250, ko01100, ko01110, ko01130, ko01230, ko05418, map00220, map00250, map01100, map01110, map01130, map01230, map05418
FUN_003376	Belongs to the glycosyl hydrolase 2 family	-	3.2.1.31	ko:K01195	ko00040, ko00531, ko00860, ko00944, ko00983, ko01100, ko01110, ko04142, map00040, map00531, map00860, map00944, map00983, map01100, map01110, map04142
FUN_003377	Uncharacterised MFS-type transporter YbfB	-	-	-	-
FUN_003378	Serine aminopeptidase, S33	-	-	-	-
FUN_003379	cytochrome P450	-	-	-	-
FUN_003380	FAD binding domain	-	-	-	-
FUN_003382	Peptidase dimerisation domain	-	-	ko:K14262	-
FUN_003384	Starch binding domain	gla1	3.2.1.3	ko:K01178	ko00500, ko01100, map00500, map01100
FUN_003390	Belongs to the DapA family	-	4.3.3.7	ko:K01714	ko00261, ko00300, ko01100, ko01110, ko01120, ko01130, ko01230, map00261, map00300, map01100, map01110, map01120, map01130, map01230
FUN_003391	Major Facilitator Superfamily	-	-	-	-
FUN_003392	phosphorelay sensor kinase activity	-	-	-	-
FUN_003396	POT family	PTR2	-	ko:K03305	-
FUN_003397	proline-specific peptidase protein	-	3.5.1.101	ko:K18457	-
FUN_003398	amino acid	-	-	-	-
FUN_003401	Thioredoxin-like domain	-	-	-	-

FUN_003402	Poly(ADP-ribose) polymerase and DNA-Ligase Zn-finger region	-	4.1.1.44	ko:K01607	ko00362, ko01100, ko01120, ko01220, map00362, map01100, map01120, map01220
FUN_003403	Involved in maceration and soft-rotting of plant tissue	-	3.1.1.11, 3.2.1.15	ko:K01051, ko:K01184	ko00040, ko01100, map00040, map01100
FUN_003404	Acetyltransferase (GNAT) domain	-	-	-	-
FUN_003405	Glycoside hydrolase family 2 protein	-	-	-	-
FUN_003406	Protein tyrosine kinase	-	-	-	-
FUN_003408	Splits internally a 1, 3-beta-glucan molecule and transfers the newly generated reducing end (the donor) to the non- reducing end of another 1, 3-beta-glucan molecule (the acceptor) forming a 1, 3-beta linkage, resulting in the elongation of 1, 3- beta-glucan chains in the cell wall	-	-	-	-
FUN_003409	RNA polymerase II transcription elongation factor	-	-	-	-
FUN_003410	Required for the assembly and or stability of the 40S ribosomal subunit. Required for the processing of the 20S rRNA- precursor to mature 18S rRNA in a late step of the maturation of 40S ribosomal subunits	RPS0	-	ko:K02998	ko03010, map03010
FUN_003411	Ribosomal_S15	-	-	-	-
FUN_003412	Belongs to the peroxidase family	-	-	-	-
FUN_003413	Catalyzes the hydrolytic deamination of adenine to hypoxanthine. Plays an important role in the purine salvage pathway and in nitrogen catabolism	AAH1	3.5.4.4	ko:K01488	ko00230, ko01100, ko05340, map00230, map01100, map05340
FUN_003414	GrpB protein	-	-	-	-
FUN_003416	PLAC8 family	-	-	-	-
FUN_003419	RNA polymerase Rpb4	-	-	-	-
FUN_003420	NOSIC (NUC001) domain	NOP58	-	ko:K14565	ko03008, map03008
FUN_003421	Belongs to the actin family	-	-	ko:K16576	-
FUN_003423	Alpha/beta hydrolase family	-	-	-	-
FUN_003424	Belongs to the sulfatase family	-	-	-	-
FUN_003428	pyroglutamyl-peptidase activity	-	3.4.19.3	ko:K01304	-
FUN_003430	The AROM polypeptide catalyzes 5 consecutive enzymatic reactions in prechorismate polyaromatic amino acid biosynthesis	ARO1	1.1.1.25, 2.5.1.19, 2.7.1.71, 4.2.1.10, 4.2.3.4	ko:K13830	ko00400, ko01100, ko01110, ko01130, ko01230, map00400, map01100, map01110, map01130, map01230
FUN_003432	Phosphoglycerate mutase family	PMU1	-	-	-
FUN_003433	Belongs to the isocitrate and isopropylmalate dehydrogenases family	IDP1	1.1.1.42	ko:K00031	ko00020, ko00480, ko00720, ko01100, ko01110, ko01120, ko01130, ko01200, ko01210, ko01230, ko04146, map00020, map00480, map00720, map01100, map01110, map01120, map01130, map01200, map01210, map01230, map04146
FUN_003435	Helix-turn-helix	MBF1	-	ko:K03627	-
FUN_003437	Formate--tetrahydrofolate ligase	ADE3	1.5.1.5, 3.5.4.9, 6.3.4.3	ko:K00288	ko00670, ko01100, map00670, map01100

FUN_003439	component of the eukaryotic translation initiation factor 3 (eIF-3) complex, which is involved in protein synthesis of a specialized repertoire of mRNAs and, together with other initiation factors, stimulates binding of mRNA and methionyl-tRNAi to the 40S ribosome. The eIF-3 complex specifically targets and initiates translation of a subset of mRNAs involved in cell proliferation	TIF34	-	ko:K03246	ko03013, map03013
FUN_003440	Alpha-trehalose glucohydrolase	NTH1	3.2.1.28	ko:K01194	ko00500, ko01100, map00500, map01100
FUN_003442	Glycosyl hydrolase family 61	-	-	-	-
FUN_003445	Zinc finger, C2H2 type	-	-	-	-
FUN_003447	Protein of unknown function (DUF3645)	-	-	-	-
FUN_003448	Sugar (and other) transporter	-	-	-	-
FUN_003449	cytochrome P450	-	-	-	-
FUN_003450	Aldehyde dehydrogenase family	-	-	-	-
FUN_003452	Belongs to the major facilitator superfamily. Sugar transporter (TC 2.A.1.1) family	-	-	-	-
FUN_003453	Promotes mitochondrial protein synthesis. May act as a fidelity factor of the translation reaction, by catalyzing a one- codon backward translocation of tRNAs on improperly translocated ribosomes. Binds to mitochondrial ribosomes in a GTP-dependent manner	GUF1	-	ko:K21594	-
FUN_003454	HORMA domain	MAD2	-	ko:K02537	ko04110, ko04111, ko04113, ko04114, ko04914, ko05166, map04110, map04111, map04113, map04114, map04914, map05166
FUN_003455	Glycosyl transferase family group 2	-	-	-	-
FUN_003456	DNA-dependent RNA polymerase catalyzes the transcription of DNA into RNA using the four ribonucleoside triphosphates as substrates	RPO31	2.7.7.6	ko:K03018, ko:K21594	ko00230, ko00240, ko01100, ko03020, ko04623, ko05169, map00230, map00240, map01100, map03020, map04623, map05169
FUN_003459	Alcohol dehydrogenase GroES-like domain	-	1.1.1.2	ko:K00002	ko00010, ko00040, ko00561, ko00930, ko01100, ko01110, ko01120, ko01130, ko01220, map00010, map00040, map00561, map00930, map01100, map01110, map01120, map01130, map01220
FUN_003460	Fungal trichothecene efflux pump (TRI12)	-	-	-	-
FUN_003462	Neutral/alkaline non-lysosomal ceramidase, N-terminal	-	3.5.1.23	ko:K12349	ko00600, ko01100, ko04071, map00600, map01100, map04071
FUN_003463	Breast carcinoma amplified sequence 2 (BCAS2)	-	-	ko:K12861	ko03040, map03040
FUN_003465	KR domain	-	-	-	-
FUN_003466	DJ-1/PfpI family	-	-	-	-
FUN_003467	Integral membrane protein	-	-	-	-
FUN_003468	Transferrin receptor-like dimerisation domain	-	3.4.17.21	ko:K01301	-
FUN_003469	SelR domain	-	1.8.4.12	ko:K07305	-
FUN_003473	Belongs to the 5'-nucleotidase family	-	3.1.3.5	ko:K01081	ko00230, ko00240, ko00760, ko01100, ko01110, map00230, map00240, map00760, map01100, map01110
FUN_003479	Belongs to the peptidase C2 family	-	-	-	-
FUN_003482	Biotin carboxylase C-terminal domain	-	6.4.1.1	ko:K01958	ko00020, ko00620, ko00720, ko01100, ko01120, ko01200, ko01230, map00020, map00620, map00720, map01100, map01120, map01200, map01230
FUN_003483	Carboxyl transferase domain	-	-	-	-
FUN_003485	Transketolase, pyrimidine binding domain	-	1.2.4.4	ko:K00167	ko00280, ko00640, ko01100, ko01110, ko01130, map00280, map00640, map01100, map01110, map01130
FUN_003486	Domain of unknown function DUF21	MAM3	-	ko:K16302	-

FUN_003487	iron ion binding	-	-	-	-
FUN_003488	Acyltransferase family	-	-	-	-
FUN_003489	hexose biosynthetic process	ERT1	-	-	-
FUN_003490	Riboflavin kinase	FMN1	2.7.1.26	ko:K00861	ko00740, ko01100, ko01110, map00740, map01100, map01110
FUN_003491	Aldo/keto reductase family	-	-	-	-
FUN_003493	FF domain	-	-	ko:K12824	ko03040, ko04212, map03040, map04212
FUN_003494	Proteasome subunit alpha type	PRE9	3.4.25.1	ko:K02728	ko03050, map03050
FUN_003496	Mixed-linked glucanase involved in the degradation of complex natural cellulosic	-	-	-	-
FUN_003497	SPOC domain	BYE1	-	-	-
FUN_003499	Putative GTP-ase activating proteins for the small GTPase, ARF	AGE2	-	ko:K12486	ko04144, map04144
FUN_003500	SNARE complex subunit protein	-	-	ko:K08504	ko04130, map04130
FUN_003501	Belongs to the oxygen-dependent FAD-linked oxidoreductase family	-	-	-	-
FUN_003505	Pyruvate ferredoxin/flavodoxin oxidoreductase	MET10	1.8.1.2	ko:K00380	ko00920, ko01100, ko01120, map00920, map01100, map01120
FUN_003506	Belongs to the glycosyl hydrolase 7 (cellulase C) family	-	3.2.1.4, 3.2.1.91	ko:K01225, ko:K19357	ko00500, ko01100, map00500, map01100
FUN_003507	Protein fmp-52, mitochondrial	FMP52	-	ko:K17290	-
FUN_003508	Belongs to the D-isomer specific 2-hydroxyacid dehydrogenase family	-	1.1.1.399, 1.1.1.95	ko:K00058	ko00260, ko00680, ko01100, ko01120, ko01130, ko01200, ko01230, map00260, map00680, map01100, map01120, map01130, map01200, map01230
FUN_003509	Catalyzes the conversion of hemimercaptal, formed from methylglyoxal and glutathione, to S-lactoylglutathione	GLO1	4.4.1.5	ko:K01759	ko00620, map00620
FUN_003511	3-alpha domain	-	-	-	-
FUN_003513	Zinc-finger of the MIZ type in Nse subunit	-	-	-	-
FUN_003514	Belongs to the GST superfamily	-	2.5.1.18	ko:K00799	ko00480, ko00980, ko00982, ko00983, ko01524, ko05200, ko05204, ko05225, ko05418, map00480, map00980, map00982, map00983, map01524, map05200, map05204, map05225, map05418
FUN_003515	Sugar (and other) transporter	-	-	-	-
FUN_003516	Sulfatase	-	3.1.6.1	ko:K01130	ko00140, ko00600, map00140, map00600
FUN_003518	Belongs to the glycosyl hydrolase family 6	-	3.2.1.91	ko:K19668	ko00500, ko01100, ko02020, map00500, map01100, map02020
FUN_003520	Belongs to the major facilitator superfamily. Sugar transporter (TC 2.A.1.1) family	-	-	-	-
FUN_003521	glycoside hydrolase family	-	3.2.1.25	ko:K01192	ko00511, ko04142, map00511, map04142
FUN_003522	A Receptor for Ubiquitination Targets	-	-	-	-
FUN_003523	Belongs to the major facilitator superfamily. Sugar transporter (TC 2.A.1.1) family	-	-	-	-
FUN_003524	Belongs to the major facilitator superfamily. Sugar transporter (TC 2.A.1.1) family	-	-	-	-
FUN_003525	Transketolase, thiamine diphosphate binding domain	-	2.2.1.3	ko:K17100	ko00680, ko01120, ko01200, map00680, map01120, map01200
FUN_003526	Cytochrome oxidase assembly protein	COX15	-	ko:K02259	ko00190, ko00860, ko01100, ko01110, ko02020, ko04714, map00190, map00860, map01100, map01110, map02020, map04714
FUN_003527	Broad-Complex, Tramtrack and Bric a brac	-	-	-	-
FUN_003532	alpha/beta hydrolase fold	-	-	-	-
FUN_003533	Uncharacterized alpha/beta hydrolase domain (DUF2235)	-	-	-	-
FUN_003534	Zinc alcohol dehydrogenase	-	-	-	-

FUN_003535	PhoD-like phosphatase, N-terminal domain	-	3.1.3.1	ko:K01113	ko00790, ko01100, ko02020, map00790, map01100, map02020
FUN_003536	Uncharacterized alpha/beta hydrolase domain (DUF2235)	-	-	-	-
FUN_003537	Component of the Mediator complex, a coactivator involved in the regulated transcription of nearly all RNA polymerase II-dependent genes. Mediator functions as a bridge to convey information from gene-specific regulatory proteins to the basal RNA polymerase II transcription machinery	MED7	-	ko:K15148	-
FUN_003538	Zinc-binding dehydrogenase	-	-	-	-
FUN_003540	SMR domain-containing protein	-	-	-	-
FUN_003541	Domain Homologous to E6-AP Carboxyl Terminus with	UFD4	2.3.2.26	ko:K10590	ko04120, map04120
FUN_003542	Belongs to the eukaryotic NMN adenylyltransferase family	NMA1	2.7.7.1, 2.7.7.18	ko:K06210	ko00760, ko01100, map00760, map01100
FUN_003543	Glucan 1, 3-beta-glucosidase	exgD	3.2.1.58	ko:K01210	ko00500, map00500
FUN_003545	NAD-P-binding protein	-	-	-	-
FUN_003547	Sugar (and other) transporter	GIT2	-	-	-
FUN_003549	Peroxisomal biogenesis factor 11 (PEX11)	PEX11	-	ko:K13352	ko04146, map04146
FUN_003550	Belongs to the class I-like SAM-binding methyltransferase superfamily. mRNA cap 0 methyltransferase family	ABD1	2.1.1.56	ko:K00565	ko03015, map03015
FUN_003551	26S proteasome subunit RPN7	RPN7	-	ko:K03037	ko03050, ko05169, map03050, map05169
FUN_003552	Aminotransferase class I and II	ALT1	2.6.1.2	ko:K00814	ko00220, ko00250, ko00710, ko01100, ko01120, ko01200, ko01210, ko01230, map00220, map00250, map00710, map01100, map01120, map01200, map01210, map01230
FUN_003553	FAD binding domain	-	-	-	-
FUN_003554	tam domain methyltransferase protein	-	-	-	-
FUN_003558	Protein kinase C conserved region 2 (CalB)	-	-	-	-
FUN_003561	Extracellular tail, of 10TM putative phosphate transporter	PHM7	-	ko:K21989	-
FUN_003562	Protein tyrosine kinase	-	-	-	-
FUN_003563	Belongs to the peptidase A1 family	-	-	-	-
FUN_003570	Membrane transport protein	-	-	-	-
FUN_003571	Ras family	-	-	-	-
FUN_003573	Glycosyl hydrolase family 61	-	-	-	-
FUN_003575	Belongs to the oxygen-dependent FAD-linked oxidoreductase family	-	-	-	-
FUN_003576	LVIVD repeat	-	-	-	-
FUN_003577	amine dehydrogenase activity	-	-	-	-
FUN_003578	FAD binding domain	-	1.14.13.1	ko:K00480	ko00621, ko00624, ko00626, ko01100, ko01120, ko01220, map00621, map00624, map00626, map01100, map01120, map01220
FUN_003579	Phytanoyl-CoA dioxygenase (PhyH)	-	-	-	-
FUN_003580	Sugar (and other) transporter	-	-	-	-
FUN_003581	X-Pro dipeptidyl-peptidase C-terminal non-catalytic domain	-	-	ko:K06978	-
FUN_003582	Phytanoyl-CoA dioxygenase (PhyH)	-	-	-	-
FUN_003584	Enoyl-(Acyl carrier protein) reductase	RDH1	1.3.1.33	ko:K00218	ko00860, ko01100, ko01110, map00860, map01100, map01110
FUN_003586	Glycoside hydrolase family 2 protein	-	-	-	-

FUN_003590	PPIases accelerate the folding of proteins. It catalyzes the cis-trans isomerization of proline imidic peptide bonds in oligopeptides	RRD1	-	ko:K17605	ko04931, map04931
FUN_003592	HBS1 N-terminus	-	-	ko:K12595, ko:K14416	ko03015, ko03018, ko05134, map03015, map03018, map05134
FUN_003593	Spinocerebellar ataxia type 10 protein domain	-	2.3.1.225	ko:K18932, ko:K19323, ko:K20003	-
FUN_003594	Prefoldin subunit	-	-	ko:K17560	-
FUN_003595	CDK-activating kinase assembly factor MAT1	TFB3	-	ko:K10842	ko03022, ko03420, map03022, map03420
FUN_003598	Pyridoxamine 5'-phosphate oxidase	-	-	-	-
FUN_003600	Major Facilitator Superfamily	-	-	-	-
FUN_003601	Thiopurine S-methyltransferase (TPMT)	-	2.1.1.165	ko:K21552	-
FUN_003602	OTU-like cysteine protease	OTU2	3.4.19.12	ko:K18342	-
FUN_003604	Major Facilitator Superfamily	-	-	-	-
FUN_003606	alpha-amylase	-	3.2.1.1	ko:K01176	ko00500, ko01100, ko04973, map00500, map01100, map04973
FUN_003607	Belongs to the type-B carboxylesterase lipase family	-	-	-	-
FUN_003608	Major Facilitator Superfamily	-	-	-	-
FUN_003610	NAD(P)H-binding	-	-	-	-
FUN_003611	Sterile alpha motif.	-	-	-	-
FUN_003612	Phospholipase D Active site motif	SPO14	3.1.4.4	ko:K01115	ko00564, ko00565, ko01100, ko01110, ko04014, ko04024, ko04071, ko04072, ko04144, ko04666, ko04724, ko04912, ko05231, map00564, map00565, map01100, map01110, map04014, map04024, map04071, map04072, map04144, map04666, map04724, map04912, map05231
FUN_003615	BNR repeat-like domain	-	-	-	-
FUN_003616	Belongs to the glycosyl hydrolase 5 (cellulase A) family	-	3.2.1.78	ko:K19355	ko00051, map00051
FUN_003618	cAMP-dependent protein kinase regulatory subunit	PKAR	-	ko:K04739	ko04910, map04910
FUN_003619	Carbohydrate-binding module family 48 protein	CRP1	-	-	-
FUN_003622	Vacuolar ATPase is responsible for acidifying a variety of intracellular compartments in eukaryotic cells. The active enzyme consists of a catalytic V1 domain attached to an integral membrane V0 proton pore complex. This subunit is a non-integral membrane component of the membrane pore domain and is required for proper assembly of the V0 sector. Might be involved in the regulated assembly of V1 subunits onto the membrane sector or alternatively may prevent the passage of protons through V0 pores	VMA6	-	ko:K02146	ko00190, ko01100, ko04142, ko04145, ko04721, ko04966, ko05110, ko05120, ko05152, ko05203, ko05323, map00190, map01100, map04142, map04145, map04721, map04966, map05110, map05120, map05152, map05203, map05323
FUN_003623	Ubiquitin homologues	-	-	ko:K13113	ko04212, map04212
FUN_003624	tRNA pseudouridine synthase	DEG1	5.4.99.45	ko:K01855	-
FUN_003625	Vault protein inter-alpha-trypsin domain	-	-	-	-
FUN_003626	Phosphoribosyl synthetase-associated domain	PRS5	2.7.6.1	ko:K00948	ko00030, ko00230, ko01100, ko01110, ko01120, ko01130, ko01200, ko01230, map00030, map00230, map01100, map01110, map01120, map01130, map01200, map01230
FUN_003627	family decarboxylase	-	-	-	-
FUN_003629	Belongs to the isocitrate lyase PEP mutase superfamily. Isocitrate lyase family	ICL1	4.1.3.1	ko:K01637	ko00630, ko01100, ko01110, ko01120, ko01200, map00630, map01100, map01110, map01120, map01200

FUN_003630	Protein of unknown function (DUF1365)	-	-	-	-
FUN_003631	Animal haem peroxidase	-	-	-	-
FUN_003634	GTPase-activator protein for Ras-like GTPases	BUD2	-	-	-
FUN_003635	Cyclophilin type peptidyl-prolyl cis-trans isomerase/CLD	CPR6	5.2.1.8	ko:K05864	ko04217, ko04218, map04217, map04218
FUN_003636	Interferon-related developmental regulator (IFRD)	-	-	-	-
FUN_003637	Not1 N-terminal domain, CCR4-Not complex component	NOT5	-	ko:K12580	ko03018, map03018
FUN_003638	deacylase	DTD1	-	ko:K07560	-
FUN_003639	His(2)-Cys(2) zinc finger	SPT10	-	-	-
FUN_003640	O-methyltransferase	-	-	-	-
FUN_003641	Pyridoxamine 5'-phosphate oxidase	-	-	ko:K07005	-
FUN_003642	cytochrome P450	-	1.7.1.14	ko:K15877	ko00910, ko01120, map00910, map01120
FUN_003644	Proteasome subunit alpha type	SCL1	3.4.25.1	ko:K02730	ko03050, map03050
FUN_003647	Alcohol dehydrogenase GroES-like domain	-	-	-	-
FUN_003648	Fungal specific transcription factor domain	-	-	-	-
FUN_003649	Carboxymuconolactone decarboxylase family	-	4.1.1.44	ko:K01607	ko00362, ko01100, ko01120, ko01220, map00362, map01100, map01120, map01220
FUN_003650	NAD-binding of NADP-dependent 3-hydroxyisobutyrate dehydrogenase	-	1.1.1.31	ko:K00020	ko00280, ko01100, map00280, map01100
FUN_003651	Metallo-beta-lactamase superfamily	-	-	-	-
FUN_003652	Metallo-beta-lactamase superfamily	-	-	-	-
FUN_003653	Major Facilitator Superfamily	-	-	-	-
FUN_003654	NAD-binding of NADP-dependent 3-hydroxyisobutyrate dehydrogenase	-	1.1.1.31	ko:K00020	ko00280, ko01100, map00280, map01100
FUN_003657	Belongs to the aldehyde dehydrogenase family	HFD1	1.2.1.3, 1.2.1.5, 1.2.1.82	ko:K00128, ko:K00129, ko:K17819	ko00010, ko00053, ko00071, ko00280, ko00310, ko00330, ko00340, ko00350, ko00360, ko00380, ko00410, ko00561, ko00620, ko00625, ko00903, ko00906, ko00980, ko00981, ko00982, ko01100, ko01110, ko01120, ko01130, ko05204, map00010, map00053, map00071, map00280, map00310, map00330, map00340, map00350, map00360, map00380, map00410, map00561, map00620, map00625, map00903, map00906, map00980, map00981, map00982, map01100, map01110, map01120, map01130, map05204
FUN_003659	salicylate hydroxylase	-	1.14.13.1	ko:K00480	ko00621, ko00624, ko00626, ko01100, ko01120, ko01220, map00621, map00624, map00626, map01100, map01120, map01220
FUN_003660	O-methyltransferase	-	-	-	-
FUN_003661	polyketide synthase	-	-	-	-
FUN_003662	Belongs to the cytochrome P450 family	-	-	-	-
FUN_003663	GAL4-like Zn(II)2Cys6 (or C6 zinc) binuclear cluster DNA-binding domain	-	-	-	-
FUN_003664	polyketide synthase	-	-	-	-
FUN_003665	polyketide synthase	-	-	-	-
FUN_003668	Phosphoglycerate mutase family	-	-	-	-
FUN_003669	Protein of unknown function (DUF1168)	-	-	-	-
FUN_003670	Heterokaryon incompatibility protein (HET)	-	-	-	-
FUN_003671	Heterokaryon incompatibility protein (HET)	-	-	-	-
FUN_003672	Belongs to the ubiquitin-conjugating enzyme family	-	2.3.2.24	ko:K10581	ko04120, map04120

FUN_003673	Dor1-like family	-	-	ko:K20295	-
FUN_003676	Fungal trichothecene efflux pump (TRI12)	-	-	-	-
FUN_003677	NAD(P)H-binding	-	-	-	-
FUN_003681	integral membrane protein	-	-	-	-
FUN_003682	beta-lactamase	-	-	-	-
FUN_003683	Sugar (and other) transporter	-	-	ko:K08157	-
FUN_003686	Fungal trichothecene efflux pump (TRI12)	-	-	-	-
FUN_003689	Sugar (and other) transporter	-	-	-	-
FUN_003690	cyclin-dependent protein serine/threonine kinase activity	-	2.7.11.22, 2.7.11.23	ko:K02208	-
FUN_003693	FAD binding domain	-	-	-	-
FUN_003697	Belongs to the short-chain dehydrogenases reductases (SDR) family	-	-	-	-
FUN_003698	Glycoside hydrolase family 16 protein	-	-	-	-
FUN_003699	Zinc-binding dehydrogenase	-	-	-	-
FUN_003701	Organic solute transporter Ostalpha	-	-	-	-
FUN_003703	assists the folding of proteins upon ATP hydrolysis	CCT3	-	ko:K09495	-
FUN_003705	glycoside hydrolase family 13	-	3.2.1.1	ko:K01176	ko00500, ko01100, ko04973, map00500, map01100, map04973
FUN_003706	Belongs to the protein kinase superfamily	STE7	2.7.12.2	ko:K11226	ko04011, map04011
FUN_003707	Plays a role in maintenance of chromatin structure during RNA polymerase II transcription elongation thereby repressing transcription initiation from cryptic promoters. Mediates the reassembly of nucleosomes onto the promoters of at least a selected set of genes during repression	SPT6	-	ko:K11292	-
FUN_003708	Endonuclease/Exonuclease/phosphatase family	-	-	ko:K19619	-
FUN_003709	Tetratricopeptide repeat	-	-	-	-
FUN_003711	Protein of unknown function (DUF3602)	-	-	-	-
FUN_003712	Belongs to the actin family	ARP1	-	ko:K16575	-
FUN_003713	Hydrolyzes a variety of simple alpha-D-galactoside as well as more complex molecules such as oligosaccharides and polysaccharides	-	3.2.1.22	ko:K07407	ko00052, ko00561, ko00600, ko00603, map00052, map00561, map00600, map00603
FUN_003714	Belongs to the major facilitator superfamily. Sugar transporter (TC 2.A.1.1) family	-	-	-	-
FUN_003717	Major Facilitator Superfamily	-	-	-	-
FUN_003718	2OG-Fe(II) oxygenase superfamily	-	-	-	-
FUN_003719	FORKHEAD	-	-	-	-
FUN_003720	Belongs to the SNF7 family	SNF7	-	ko:K12194	ko04144, ko04217, map04144, map04217
FUN_003721	Telomere capping, CST complex subunit	-	-	-	-
FUN_003722	Amidohydrolase family	-	-	-	-
FUN_003726	Major Facilitator Superfamily	-	-	-	-
FUN_003727	Belongs to the major facilitator superfamily. Sugar transporter (TC 2.A.1.1) family	-	-	-	-
FUN_003731	PRELI-like family	-	4.1.3.38	ko:K02619	ko00790, map00790
FUN_003732	Belongs to the class-I aminoacyl-tRNA synthetase family	WRS1	6.1.1.2	ko:K01867	ko00970, map00970
FUN_003733	Belongs to the major facilitator superfamily. Sugar transporter (TC 2.A.1.1) family	-	-	ko:K08141	-
FUN_003734	Pfam:Y_phosphatase3C	-	-	-	-
FUN_003735	spindle pole body component	SPC98	-	ko:K16570	-

FUN_003736	Zinc knuckle	-	-	-	-
FUN_003737	Glycosyltransferase family 2 protein	-	2.4.1.16	ko:K00698	ko00520, map00520
FUN_003738	Low molecular weight phosphatase family	stp1	3.1.3.2, 3.1.3.48	ko:K14394	ko00730, ko00740, ko01100, map00730, map00740, map01100
FUN_003740	Major Facilitator Superfamily	-	-	-	-
FUN_003741	Serine threonine protein kinase	SNF1	2.7.11.1	ko:K12761	ko04113, ko04138, map04113, map04138
FUN_003742	Amino-transferase class IV	ABZ2	4.1.3.38	ko:K02619	ko00790, map00790
FUN_003743	Tetratricopeptide repeat	TOM70	-	ko:K17768	-
FUN_003746	Heterokaryon incompatibility protein (HET)	-	-	-	-
FUN_003747	Cytochrome P450	-	-	-	-
FUN_003748	Protein of unknown function (DUF3455)	-	-	-	-
FUN_003750	polyketide synthase	-	-	-	-
FUN_003751	Belongs to the major facilitator superfamily. Sugar transporter (TC 2.A.1.1) family	-	-	-	-
FUN_003752	Opy2 protein	-	-	ko:K19850	ko04011, map04011
FUN_003754	Aft1 HRR domain	SKO1	-	ko:K09051	ko04011, map04011
FUN_003757	Belongs to the glycosyl hydrolase 32 family	-	3.2.1.26	ko:K01193	ko00052, ko00500, ko01100, map00052, map00500, map01100
FUN_003759	Fungal trichothecene efflux pump (TRI12)	-	-	-	-
FUN_003762	RNA helicase	HAS1	3.6.4.13	ko:K13179	-
FUN_003763	NAD-P-binding protein	-	-	-	-
FUN_003765	FAD binding domain	-	-	-	-
FUN_003766	Condensation domain	-	-	-	-
FUN_003767	ABC transporter transmembrane region	-	-	-	-
FUN_003769	Belongs to the major facilitator superfamily. Sugar transporter (TC 2.A.1.1) family	-	-	-	-
FUN_003771	Mitochondrial import inner membrane translocase subunit	TIM17	-	ko:K17795	-
FUN_003772	Belongs to the TRAFAC class dynamin-like GTPase superfamily. Dynamin Fzo YdjA family	-	-	-	-
FUN_003773	Steryl acetyl hydrolase	-	-	-	-
FUN_003774	Peptidase family S58	-	3.4.11.19	ko:K01266	-
FUN_003775	to multidrug resistance protein 1	-	3.6.3.44	ko:K05658	ko02010, ko04976, ko05206, ko05226, map02010, map04976, map05206, map05226
FUN_003777	ML-like domain	FLC3	-	-	-
FUN_003782	7alpha-cephem-methoxylase P8 chain	-	-	-	-
FUN_003783	Belongs to the short-chain dehydrogenases reductases (SDR) family	-	-	-	-
FUN_003790	Belongs to the peroxidase family. Peroxidase catalase subfamily	katG	1.11.1.21	ko:K03782	ko00360, ko00380, ko00940, ko00983, ko01100, ko01110, map00360, map00380, map00940, map00983, map01100, map01110
FUN_003793	ABC transporter transmembrane region	-	3.6.3.44	ko:K05658	ko02010, ko04976, ko05206, ko05226, map02010, map04976, map05206, map05226
FUN_003794	3'-5' exonuclease	-	-	ko:K18740	-
FUN_003795	Domain of unknown function (DUF1996)	-	-	-	-
FUN_003796	Domain of unknown function (DUF1996)	-	-	-	-
FUN_003799	Belongs to the oxygen-dependent FAD-linked oxidoreductase family	-	-	-	-
FUN_003800	Cytochrome P450	-	-	-	-
FUN_003802	Heterokaryon incompatibility protein (HET)	-	-	-	-

FUN_003803	Belongs to the peptidase M28 family	-	3.4.11.15	ko:K01264	-
FUN_003804	Permease for cytosine/purines, uracil, thiamine, allantoin	-	-	ko:K03457	-
FUN_003807	Sugar (and other) transporter	-	-	-	-
FUN_003809	Alpha-trehalose glucohydrolase	NTH1	3.2.1.28	ko:K01194	ko00500, ko01100, map00500, map01100
FUN_003813	Fungal trichothecene efflux pump (TRI12)	-	-	-	-
FUN_003815	Glycoside hydrolase family 35 protein	lacB	3.2.1.23	ko:K01190	ko00052, ko00511, ko00600, ko01100, map00052, map00511, map00600, map01100
FUN_003816	Arylsulfotransferase (ASST)	-	-	-	-
FUN_003817	Major facilitator superfamily transporter	-	-	-	-
FUN_003818	Major Facilitator Superfamily	-	-	-	-
FUN_003819	Zn_pept	-	-	-	-
FUN_003821	Isochorismatase family	-	-	-	-
FUN_003823	O-methyl transferase B	-	-	-	-
FUN_003825	NAD(P)H-binding	-	-	-	-
FUN_003830	Flavoprotein (FP) subunit of succinate dehydrogenase (SDH) that is involved in complex II of the mitochondrial electron transport chain and is responsible for transferring electrons from succinate to ubiquinone (coenzyme Q)	-	1.3.5.1	ko:K00234	ko00020, ko00190, ko01100, ko01110, ko01120, ko01130, ko01200, ko04714, ko04932, ko05010, ko05012, ko05016, map00020, map00190, map01100, map01110, map01120, map01130, map01200, map04714, map04932, map05010, map05012, map05016
FUN_003831	tetraspanin	-	-	-	-
FUN_003832	Heterokaryon incompatibility protein (HET)	-	-	-	-
FUN_003833	NUDIX domain	-	3.6.1.52	ko:K07766	-
FUN_003834	Arginine-tRNA-protein transferase, N terminus	ATE1	2.3.2.8	ko:K00685	-
FUN_003835	3-oxo-5-alpha-steroid 4-dehydrogenase	TSC13	1.3.1.93	ko:K10258	ko00062, ko01040, ko01110, ko01212, map00062, map01040, map01110, map01212
FUN_003836	Animal haem peroxidase	-	1.13.11.60, 1.14.99.1, 5.4.4.5, 5.4.4.6	ko:K11987, ko:K17863, ko:K17864	ko00590, ko00591, ko01100, ko04064, ko04370, ko04657, ko04668, ko04723, ko04726, ko04913, ko04921, ko04923, ko05140, ko05165, ko05167, ko05200, ko05204, ko05206, ko05222, map00590, map00591, map01100, map04064, map04370, map04657, map04668, map04723, map04726, map04913, map04921, map04923, map05140, map05165, map05167, map05200, map05204, map05206, map05222
FUN_003840	protein localization to T-tubule	-	-	-	-
FUN_003841	Belongs to the FMO family	-	1.14.13.8	ko:K00485	ko00982, ko01120, map00982, map01120
FUN_003842	ubiE/COQ5 methyltransferase family	-	-	-	-
FUN_003843	Glycosyltransferase sugar-binding region containing DXD motif	-	-	-	-
FUN_003844	Transcriptional regulation of mitochondrial recombination	-	-	-	-
FUN_003845	Vacuolar sorting 38 and autophagy-related subunit 14	-	-	ko:K21249	ko04140, map04140
FUN_003846	TRAM1-like protein	LAG1	2.3.1.24	ko:K04709	ko00600, ko01100, map00600, map01100
FUN_003847	Belongs to the type-B carboxylesterase lipase family	-	-	-	-
FUN_003848	Glutamate-cysteine ligase	GSH1	6.3.2.2	ko:K11204	ko00270, ko00480, ko01100, ko04212, ko04216, map00270, map00480, map01100, map04212, map04216
FUN_003849	zinc finger binding to DNA consensus sequence [AT]GATA[AG]	-	-	-	-
FUN_003850	Domain of unknown function (DUF2014)	CPH2	-	-	-

FUN_003851	Haloacid dehalogenase-like hydrolase	-	3.3.2.9	ko:K01253	ko00980, ko04976, ko05204, map00980, map04976, map05204
FUN_003853	Lipopolysaccharide kinase (Kdo/WaaP) family	BUD32	2.7.11.1	ko:K08851	-
FUN_003855	Amino acid permease	-	-	ko:K16261	-
FUN_003856	amino acid	-	-	-	-
FUN_003857	Belongs to the type-B carboxylesterase lipase family	-	-	-	-
FUN_003858	Eukaryotic protein of unknown function (DUF829)	-	-	-	-
FUN_003859	Belongs to the peroxisomal membrane protein PXMP2 4 family	-	-	-	-
FUN_003860	Terpene synthase family protein	-	4.2.3.19, 5.5.1.13	ko:K20657	ko00904, ko01100, ko01110, map00904, map01100, map01110
FUN_003861	Belongs to the cytochrome P450 family	-	-	-	-
FUN_003862	FAD linked oxidase-like protein	-	-	-	-
FUN_003863	EVE domain	-	-	-	-
FUN_003864	FAD binding domain	-	-	-	-
FUN_003865	Belongs to the type-B carboxylesterase lipase family	-	-	-	-
FUN_003867	Peptidase family M48	OMA1	-	-	-
FUN_003868	Pleckstrin homology domain	-	-	-	-
FUN_003869	Belongs to the class VI-like SAM-binding methyltransferase superfamily. Isoprenylcysteine carboxyl methyltransferase family	STE14	2.1.1.100	ko:K00587	ko00900, ko01130, map00900, map01130
FUN_003870	Cryptococcal mannosyltransferase 1	-	-	-	-
FUN_003871	ZIP Zinc transporter	ZRT3	-	ko:K07238	-
FUN_003872	PhoX homologous domain, present in p47phox and p40phox.	ATG20	-	ko:K21996	-
FUN_003874	Putative death-receptor fusion protein (DUF2428)	-	-	-	-
FUN_003876	Amidase	-	3.5.1.4	ko:K01426	ko00330, ko00360, ko00380, ko00627, ko00643, ko01120, map00330, map00360, map00380, map00627, map00643, map01120
FUN_003877	Plays a role in vesicular protein sorting	VPS17	-	-	-
FUN_003878	Mitochondrial GTPase that mediates the disassembly of ribosomes from messenger RNA at the termination of mitochondrial protein biosynthesis. Not involved in the GTP-dependent ribosomal translocation step during translation elongation	MEF2	-	ko:K02355	-
FUN_003879	O-acyltransferase	-	2.3.1.20, 2.3.1.75, 2.3.1.76	ko:K11155	ko00561, ko00830, ko01100, ko04975, map00561, map00830, map01100, map04975
FUN_003880	WD domain, G-beta repeat	PFS2	-	ko:K15542	ko03015, map03015
FUN_003881	domain present in translation initiation factor eIF2B and eIF5	TIF5	-	ko:K03262	ko03013, ko04214, map03013, map04214
FUN_003882	Thiamine monophosphate synthase	THI6	2.5.1.3, 2.7.1.50	ko:K14154	ko00730, ko01100, map00730, map01100
FUN_003883	PHD zinc finger	-	-	-	-
FUN_003884	Saccharopine dehydrogenase NADP binding domain	-	-	-	-
FUN_003885	CRT-like, chloroquine-resistance transporter-like	-	-	ko:K15287	-
FUN_003887	Component of IIS longevity pathway SMK-1	PSY2	-	ko:K17491	ko04212, ko04922, map04212, map04922
FUN_003888	Belongs to the glycosyl hydrolase 12 (cellulase H) family	-	3.2.1.151	ko:K18576	-
FUN_003889	Belongs to the dihydrofolate reductase family	-	1.5.1.3	ko:K00287	ko00670, ko00790, ko01100, ko01523, map00670, map00790, map01100, map01523

FUN_003890	MAPEG family	-	2.5.1.18	ko:K00799	ko00480, ko00980, ko00982, ko00983, ko01524, ko05200, ko05204, ko05225, ko05418, map00480, map00980, map00982, map00983, map01524, map05200, map05204, map05225, map05418
FUN_003892	Cid1 family poly A polymerase	-	-	-	-
FUN_003893	Isochorismatase family	-	-	-	-
FUN_003894	Acetate kinase	-	2.7.2.1	ko:K00925	ko00430, ko00620, ko00640, ko00680, ko00720, ko01100, ko01120, ko01200, map00430, map00620, map00640, map00680, map00720, map01100, map01120, map01200
FUN_003895	Phosphoketolase	-	4.1.2.22, 4.1.2.9	ko:K01621	ko00030, ko00710, ko01100, ko01120, map00030, map00710, map01100, map01120
FUN_003896	Enoyl-(Acyl carrier protein) reductase	-	-	-	-
FUN_003897	Belongs to the type-B carboxylesterase lipase family	-	3.1.1.7, 3.1.1.8	ko:K01049, ko:K01050	ko00564, ko04725, map00564, map04725
FUN_003899	Major Facilitator Superfamily	-	-	-	-
FUN_003900	FAD binding domain	-	1.14.13.1	ko:K00480	ko00621, ko00624, ko00626, ko01100, ko01120, ko01220, map00621, map00624, map00626, map01100, map01120, map01220
FUN_003901	Belongs to the tannase family	-	3.1.1.73	ko:K09252	-
FUN_003902	Protein of unknown function (DUF3500)	-	-	-	-
FUN_003903	Protein of unknown function (DUF3176)	-	-	-	-
FUN_003905	Belongs to the histidine acid phosphatase family	-	3.1.3.2	ko:K01078	ko00730, ko00740, ko01100, map00730, map00740, map01100
FUN_003906	Fungal specific transcription factor domain	-	-	-	-
FUN_003907	Enoyl-(Acyl carrier protein) reductase	-	1.1.1.352	ko:K17652	ko00254, map00254
FUN_003908	Alcohol acetyltransferase	ATF1	-	-	-
FUN_003909	transferase activity, transferring nitrogenous groups	-	-	ko:K22121	-
FUN_003911	Histone-lysine N-methyltransferase	set9	2.1.1.43	ko:K11429	ko00310, map00310
FUN_003912	Belongs to the universal ribosomal protein uL14 family	mrpl38	-	-	-
FUN_003913	RNA polymerases M/15 Kd subunit	-	-	ko:K03017	ko00230, ko00240, ko01100, ko03020, ko05016, ko05169, map00230, map00240, map01100, map03020, map05016, map05169
FUN_003914	peptidyl-prolyl cis-trans isomerase	pin1	5.2.1.8	ko:K09578	ko04622, map04622
FUN_003916	PH domain	BUD4	-	-	-
FUN_003917	helicase superfamily c-terminal domain	MRH4	3.6.4.13	ko:K17678	-
FUN_003918	N-terminal domain of oxidoreductase	QOR1	-	ko:K07119	-
FUN_003919	Aldo/keto reductase family	xyl1	1.1.1.307	ko:K17743	ko00040, ko01100, map00040, map01100
FUN_003921	Glycine D-amino acid oxidases (deaminating)	-	1.4.3.3	ko:K00273	ko00260, ko00311, ko00330, ko00472, ko01100, ko01130, ko04146, map00260, map00311, map00330, map00472, map01100, map01130, map04146
FUN_003924	Amino acid permease	-	-	-	-
FUN_003926	Belongs to the peptidase C19 family	-	3.4.19.12	ko:K11853	-
FUN_003927	Signal-recognition-particle assembly has a crucial role in targeting secretory proteins to the rough endoplasmic reticulum membrane	srp72	-	ko:K03108	ko03060, map03060

FUN_003930	The pyruvate dehydrogenase complex catalyzes the overall conversion of pyruvate to acetyl-CoA and CO(2)	PDA1	1.2.4.1	ko:K00161	ko00010, ko00020, ko00620, ko01100, ko01110, ko01120, ko01130, ko01200, ko04066, ko04922, ko05230, map00010, map00020, map00620, map01100, map01110, map01120, map01130, map01200, map04066, map04922, map05230
FUN_003931	Belongs to the type-B carboxylesterase lipase family	-	-	-	-
FUN_003932	Belongs to the mitochondrial carrier (TC 2.A.29) family	PIC2	-	ko:K15102	-
FUN_003934	A Receptor for Ubiquitination Targets	-	-	ko:K03361	ko04111, ko04120, map04111, map04120
FUN_003935	Glycoside hydrolase family 61 protein	-	-	-	-
FUN_003936	alpha/beta hydrolase fold	cys2	2.3.1.31	ko:K00641	ko00270, ko01100, ko01130, map00270, map01100, map01130
FUN_003937	Belongs to the major facilitator superfamily. Sugar transporter (TC 2.A.1.1) family	-	-	-	-
FUN_003938	Ku70 and Ku80 are 70kDa and 80kDa subunits of the Lupus Ku autoantigen	KU70	-	ko:K10884	ko03450, map03450
FUN_003939	Catalyzes the ferrous insertion into protoporphyrin IX	HEM15	4.99.1.1, 4.99.1.9	ko:K01772	ko00860, ko01100, ko01110, map00860, map01100, map01110
FUN_003941	transcription initiation factor IIE subunit alpha	-	-	ko:K03136	ko03022, ko05169, ko05203, map03022, map05169, map05203
FUN_003942	tRNA pseudouridine synthase	PUS1	5.4.99.12, 5.4.99.44	ko:K06173, ko:K15452	-
FUN_003944	NADH:flavin oxidoreductase / NADH oxidase family	OYE32	1.3.1.42	ko:K05894	ko00592, ko01100, ko01110, map00592, map01100, map01110
FUN_003945	Belongs to the tannase family	-	-	-	-
FUN_003946	Major Facilitator Superfamily	-	-	-	-
FUN_003947	Est1 DNA/RNA binding domain	-	-	-	-
FUN_003948	Chromatin associated protein KTI12	-	-	-	-
FUN_003949	Glycosyltransferase family 2 protein	ALG5	2.4.1.117	ko:K00729	ko00510, ko01100, map00510, map01100
FUN_003953	malate L-lactate dehydrogenase	-	-	-	-
FUN_003954	Dehydratase family	-	4.2.1.9	ko:K01687	ko00290, ko00770, ko01100, ko01110, ko01130, ko01210, ko01230, map00290, map00770, map01100, map01110, map01130, map01210, map01230
FUN_003957	Belongs to the amidase family	-	3.5.1.4, 6.3.5.6, 6.3.5.7	ko:K01426, ko:K02433	ko00330, ko00360, ko00380, ko00627, ko00643, ko00970, ko01100, ko01120, map00330, map00360, map00380, map00627, map00643, map00970, map01100, map01120
FUN_003958	PrpF protein	-	-	-	-
FUN_003959	FAD binding domain	-	-	-	-
FUN_003960	Major Facilitator Superfamily	-	-	-	-
FUN_003962	DNA ligase	LIG4	6.5.1.1	ko:K10777	ko03450, map03450
FUN_003963	increased rdna silencing protein 4	IRS4	-	-	-
FUN_003964	A domain family that is part of the cupin metalloenzyme superfamily.	-	-	ko:K19219	-
FUN_003965	Mitochondrial inner membrane protease subunit	-	-	ko:K09647	ko03060, map03060
FUN_003969	Aldehyde dehydrogenase family protein	-	-	-	-
FUN_003970	Catalyzes the transfer of a dimethylallyl group onto the adenine at position 37	tit1	2.5.1.75	ko:K00791	ko00908, ko01100, ko01110, map00908, map01100, map01110
FUN_003971	ABC transporter	CAF16	-	ko:K12608	ko03018, map03018
FUN_003973	Heme oxygenase	-	-	-	-
FUN_003975	Mitochondrial import receptor subunit Tom22	tom22	-	ko:K17769	-

FUN_003976	Eukaryotic porin	TOM40	-	ko:K11518	ko05014, map05014
FUN_003977	6-phosphofructo-2-kinase	-	2.7.1.105, 3.1.3.46	ko:K19030	ko00051, ko04152, map00051, map04152
FUN_003978	Belongs to the ubiquitin-conjugating enzyme family	-	2.3.2.23	ko:K10578	ko04120, ko04141, ko05012, map04120, map04141, map05012
FUN_003980	FabD lysophospholipase-like protein	-	-	-	-
FUN_003981	Glycosyltransferase family 31 protein	-	-	-	-
FUN_003982	Alcohol dehydrogenase GroES-like domain	-	-	-	-
FUN_003984	PKS_DH	-	-	-	-
FUN_003985	Fungal trichothecene efflux pump (TRI12)	-	-	-	-
FUN_003986	Carbohydrate esterase family 4 protein	-	-	-	-
FUN_003988	Domain of unknown function (DUF427)	-	-	-	-
FUN_003990	FAD binding domain	-	-	-	-
FUN_003993	Alcohol dehydrogenase GroES-like domain	-	1.1.1.1	ko:K13953	ko00010, ko00071, ko00350, ko00625, ko00626, ko00830, ko00980, ko00982, ko01100, ko01110, ko01120, ko01130, ko01220, map00010, map00071, map00350, map00625, map00626, map00830, map00980, map00982, map01100, map01110, map01120, map01130, map01220
FUN_003994	Pyridine nucleotide-disulphide oxidoreductase	-	-	-	-
FUN_003996	FAD binding domain	-	-	-	-
FUN_003998	Amb_all	plyA	4.2.2.2	ko:K01728	ko00040, ko02024, map00040, map02024
FUN_004003	Enoyl-(Acyl carrier protein) reductase	-	-	-	-
FUN_004004	Sugar (and other) transporter	-	-	-	-
FUN_004005	Alcohol dehydrogenase GroES-like domain	-	-	-	-
FUN_004006	O-methyltransferase	-	-	-	-
FUN_004007	FAD binding domain	-	-	-	-
FUN_004008	Belongs to the type-B carboxylesterase lipase family	-	-	-	-
FUN_004011	FAD linked oxidases, C-terminal domain	-	-	-	-
FUN_004012	FAD binding domain	-	1.14.13.1	ko:K00480	ko00621, ko00624, ko00626, ko01100, ko01120, ko01220, map00621, map00624, map00626, map01100, map01120, map01220
FUN_004013	AMP-binding enzyme	-	-	-	-
FUN_004014	Domain of unknown function (DUF1993)	-	-	ko:K09983	-
FUN_004015	Glycoside hydrolase family 76 protein	-	-	-	-
FUN_004016	Fungal specific transcription factor domain	-	-	-	-
FUN_004017	Major Facilitator Superfamily	-	-	-	-
FUN_004018	Beta-lactamase	-	-	-	-
FUN_004019	Elongation factor Tu domain 2	-	-	-	-
FUN_004020	Indoleamine 2, 3-dioxygenase	-	1.13.11.52	ko:K00463	ko00380, ko01100, ko05143, map00380, map01100, map05143
FUN_004021	RNA ligase	-	-	-	-
FUN_004022	Microfibril-associated/Pre-mRNA processing	-	-	ko:K13110	-
FUN_004025	Major Facilitator Superfamily	-	-	-	-
FUN_004026	Transferase family	-	-	-	-

FUN_004027	Belongs to the ABC transporter superfamily. ABCG family. PDR (TC 3.A.1.205) subfamily	CDR1	-	ko:K08711, ko:K08712	ko02010, map02010
FUN_004028	Belongs to the ABC transporter superfamily. ABCG family. PDR (TC 3.A.1.205) subfamily	CDR1	-	ko:K08711, ko:K08712	ko02010, map02010
FUN_004029	Arylsulfotransferase (ASST)	-	-	-	-
FUN_004030	alpha/beta hydrolase fold	-	3.3.2.9	ko:K01253	ko00980, ko04976, ko05204, map00980, map04976, map05204
FUN_004032	Glyoxalase/Bleomycin resistance protein/Dioxygenase superfamily	-	4.4.1.5	ko:K01759	ko00620, map00620
FUN_004033	Domain in cystathionine beta-synthase and other proteins.	SNF4	-	ko:K07200	ko04068, ko04152, ko04211, ko04213, ko04371, ko04530, ko04710, ko04714, ko04910, ko04920, ko04921, ko04922, ko04931, ko04932, ko05410, map04068, map04152, map04211, map04213, map04371, map04530, map04710, map04714, map04910, map04920, map04921, map04922, map04931, map04932, map05410
FUN_004034	Serine/threonine phosphatases, family 2C, catalytic domain	MgPP2CL-1	3.1.3.16	ko:K19704	ko04011, map04011
FUN_004035	Belongs to the SNF7 family	DID4	-	ko:K12191	ko04144, ko04217, map04144, map04217
FUN_004036	Belongs to the TRAFAC class dynamin-like GTPase superfamily. Dynamin Fzo YdjA family	FZO1	-	ko:K06030	ko04137, ko04214, ko04621, map04137, map04214, map04621
FUN_004037	Belongs to the TRAFAC class dynamin-like GTPase superfamily. Dynamin Fzo YdjA family	FZO1	-	ko:K06030	ko04137, ko04214, ko04621, map04137, map04214, map04621
FUN_004038	zinc finger	-	2.3.1.225	ko:K18932	-
FUN_004039	Rhomboid-like protein	-	2.3.1.225	ko:K20028	-
FUN_004040	Catalyzes the reaction of cyanate with bicarbonate to produce ammonia and carbon	cyn1	4.2.1.104	ko:K01725	ko00910, map00910
FUN_004041	Phospholipase D Active site motif	-	3.1.4.4	ko:K01115	ko00564, ko00565, ko01100, ko01110, ko04014, ko04024, ko04071, ko04072, ko04144, ko04666, ko04724, ko04912, ko05231, map00564, map00565, map01100, map01110, map04014, map04024, map04071, map04072, map04144, map04666, map04724, map04912, map05231
FUN_004042	Eukaryotic family of unknown function (DUF1754)	-	-	ko:K13120	-
FUN_004043	Dual specificity protein phosphatase, N-terminal half	CDC14	3.1.3.16, 3.1.3.48	ko:K06639	ko04110, ko04111, ko04113, map04110, map04111, map04113
FUN_004044	Set domain-containing protein	-	-	-	-
FUN_004045	Fungal specific transcription factor domain	CAT8	-	ko:K09240	-
FUN_004046	Chromatin associated protein KTI12	KTI12	-	ko:K15456	-
FUN_004048	F-box domain-containing protein	-	-	-	-
FUN_004049	Protein of unknown function (DUF3431)	-	-	-	-
FUN_004053	Dak2	dak1	2.7.1.28, 2.7.1.29, 4.6.1.15	ko:K00863	ko00051, ko00561, ko00680, ko01100, ko01120, ko01200, ko04622, map00051, map00561, map00680, map01100, map01120, map01200, map04622
FUN_004054	LSM domain	SMD2	-	ko:K11096	ko03040, map03040
FUN_004055	GATA transcription factor	-	-	-	-
FUN_004057	RNA recognition motif. (a.k.a. RRM, RBD, or RNP domain)	PUB1	-	ko:K13201	-
FUN_004058	Survival protein SurE	-	-	-	-

FUN_004059	Enoyl-(Acyl carrier protein) reductase	-	1.1.1.100	ko:K00059	ko00061, ko00333, ko00780, ko01040, ko01100, ko01130, ko01212, map00061, map00333, map00780, map01040, map01100, map01130, map01212
FUN_004060	Protein of unknown function (DUF1749)	-	-	-	-
FUN_004061	FDF	EDC3	-	ko:K12615	ko03018, map03018
FUN_004062	Transglutaminase-like superfamily	-	-	-	-
FUN_004063	GAL4-like Zn(II)2Cys6 (or C6 zinc) binuclear cluster DNA-binding domain	-	-	-	-
FUN_004064	Belongs to the cyclin family	CLB4	-	ko:K02220, ko:K06659	ko04011, ko04111, ko04113, map04011, map04111, map04113
FUN_004065	Uncharacterised protein family, YAP/Alf4/glomulin	-	-	-	-
FUN_004067	Bromodomain extra-terminal - transcription regulation	tfg3	-	ko:K03140, ko:K15306	ko03022, ko05166, ko05203, map03022, map05166, map05203
FUN_004068	Acyclic terpene utilisation family protein AtuA	-	-	-	-
FUN_004069	3-oxoacyl- acyl-carrier-protein reductase	-	-	-	-
FUN_004073	Glycosyltransferase family 62 protein	MNN9	2.4.1.232	ko:K05529	ko00513, ko01100, map00513, map01100
FUN_004074	Domain of unknown function (DUF202)	-	-	-	-
FUN_004075	Belongs to the DEAD box helicase family	DBP5	3.6.4.13	ko:K18655	-
FUN_004076	Protein kinase C conserved region 1 (C1) domains (Cysteine-rich domains)	bzz1	-	ko:K20121	-
FUN_004077	Transmembrane amino acid transporter protein	-	-	-	-
FUN_004078	Protein kinase C conserved region 1 (C1) domains (Cysteine-rich domains)	bzz1	-	ko:K20121	-
FUN_004079	Transcription factor	-	-	-	-
FUN_004080	S-adenosyl-L-methionine-dependent protein methyltransferase that trimethylates the N-terminal glycine 'Gly- 2' of elongation factor 1-alpha, before also catalyzing the mono- and dimethylation of 'Lys-3'	EFM7	2.1.1.1	ko:K17878	-
FUN_004084	Ran-binding domain	sbp1	-	ko:K15306	ko05166, ko05203, map05166, map05203
FUN_004086	Glycosyltransferase family 15 protein	omh4	-	ko:K03854	-
FUN_004087	Domain of unknown function (DUF4604)	-	-	ko:K14536	ko03008, map03008
FUN_004090	Phosphoinositide phospholipase C	-	3.1.4.11	ko:K05857	ko00562, ko01100, ko04020, ko04070, ko04919, ko04933, map00562, map01100, map04020, map04070, map04919, map04933
FUN_004092	Haloacid dehalogenase-like hydrolase	-	-	-	-
FUN_004093	Multicopper oxidase	-	-	-	-
FUN_004094	DSBA-like thioredoxin domain	-	-	-	-
FUN_004095	BAR	hob1	-	ko:K12562	ko04144, ko04666, map04144, map04666
FUN_004096	Beta-eliminating lyase	NFS1	2.8.1.7	ko:K04487	ko00730, ko01100, ko04122, map00730, map01100, map04122
FUN_004097	UEV domain	STP22	-	ko:K12183	ko04144, map04144
FUN_004098	Glycine-rich cell wall structural protein 1	-	-	-	-
FUN_004099	Cobalamin-independent synthase, Catalytic domain	-	-	-	-
FUN_004101	Fungal pheromone mating factor STE2 GPCR	STE2	-	ko:K11213	ko04011, map04011
FUN_004102	Belongs to the CRISP family	-	-	-	-
FUN_004103	Serine/Threonine protein kinases, catalytic domain	SSN3	2.7.11.22, 2.7.11.23	ko:K02208	-
FUN_004104	Belongs to the major facilitator superfamily. Sugar transporter (TC 2.A.1.1) family	HGT20	-	-	-

FUN_004105	GTP cyclohydrolase II	RIB1	3.5.4.25	ko:K01497	ko00740, ko00790, ko01100, ko01110, ko02024, map00740, map00790, map01100, map01110, map02024
FUN_004106	Nucleoporin subcomplex protein binding to Pom34	NUP188	-	ko:K14311	ko03013, map03013
FUN_004107	Trifunctional enzyme bearing the Gln amidotransferase (GATase) domain of anthranilate synthase, indole-glycerolphosphate synthase, and phosphoribosylanthranilate isomerase activities	TRP3	4.1.1.48, 4.1.3.27, 5.3.1.24	ko:K01656, ko:K13501	ko00400, ko01100, ko01110, ko01130, ko01230, map00400, map01100, map01110, map01130, map01230
FUN_004109	Homeodomain	YOX1	-	ko:K12411	-
FUN_004110	isoflavone reductase family protein	-	-	-	-
FUN_004111	breast cancer carboxy-terminal domain	-	-	-	-
FUN_004112	Endoplasmic Reticulum-Golgi Intermediate Compartment (ERGIC)	-	-	ko:K20366	-
FUN_004117	Metallo-beta-lactamase superfamily	-	-	-	-
FUN_004118	Major Facilitator Superfamily	TNA1	-	-	-
FUN_004120	Major Facilitator Superfamily	-	-	-	-
FUN_004121	Cyclophilin type peptidyl-prolyl cis-trans isomerase/CLD	cyp8	5.2.1.8	ko:K10598	ko04120, map04120
FUN_004122	palmitoyl-(protein) hydrolase activity	-	-	-	-
FUN_004123	Alcohol dehydrogenase GroES-like domain	-	-	-	-
FUN_004125	Vacuolar effluxer which mediate the efflux of amino acids resulting from autophagic degradation. The release of autophagic amino acids allows the maintenance of protein synthesis and viability during nitrogen starvation	ATG22	-	ko:K06902	ko04138, map04138
FUN_004126	Ribosomal proteins 50S-L15, 50S-L18e, 60S-L27A	MRPL10	-	ko:K02876	ko03010, map03010
FUN_004127	Cyclin	-	-	-	-
FUN_004128	Repeated motif present between transmembrane helices in cystinosin, yeast ERS1p, mannose-P-dolichol utilization defect 1, and other hypothetical proteins.	-	-	-	-
FUN_004130	NmrA-like family	-	-	-	-
FUN_004131	Domain of unknown function (DUF427)	-	-	-	-
FUN_004132	Pyridine nucleotide-disulphide oxidoreductase	NDE1	1.6.5.9	ko:K17871	-
FUN_004134	Bystin	ENP1	-	ko:K14797	-
FUN_004135	Set1 complex component ash2	ASH2	-	ko:K14967	-
FUN_004136	Pyridoxal-dependent decarboxylase conserved domain	-	4.1.1.105, 4.1.1.28	ko:K01593	ko00350, ko00360, ko00380, ko00901, ko00950, ko00965, ko01100, ko01110, ko04726, ko04728, ko05030, ko05031, ko05034, map00350, map00360, map00380, map00901, map00950, map00965, map01100, map01110, map04726, map04728, map05030, map05031, map05034
FUN_004137	Peptidase family M13	-	3.4.24.71	ko:K01415	-
FUN_004138	Belongs to the oxygen-dependent FAD-linked oxidoreductase family	-	-	-	-
FUN_004140	Heterokaryon incompatibility protein (HET)	-	-	-	-
FUN_004141	WD domain, G-beta repeat	ELP2	-	ko:K11374, ko:K18178	ko04714, map04714

FUN_004142	Acts as a sulfur carrier required for 2-thiolation of mcm(5)S(2)U at tRNA wobble positions of cytosolic tRNA(Lys), tRNA(Glu) and tRNA(Gln). Serves as sulfur donor in tRNA 2- thiolation reaction by being thiocarboxylated (-COSH) at its C- terminus by the MOCS3 homolog UBA4. The sulfur is then transferred to tRNA to form 2-thiolation of mcm(5)S(2)U. Prior mcm(5) tRNA modification by the elongator complex is required for 2- thiolation. Also acts as a ubiquitin-like protein (UBL) that is covalently conjugated via an isopeptide bond to lysine residues of target proteins such as AHP1. The thiocarboxylated form serves as substrate for conjugation and oxidative stress	URM1	-	ko:K12161	ko04122, map04122
FUN_004143	Tyrosyl-DNA phosphodiesterase	-	-	ko:K10862	-
FUN_004145	Beta-L-arabinofuranosidase, GH127	-	-	-	-
FUN_004147	glycoside hydrolase family 3 protein	-	3.2.1.21	ko:K05349	ko00460, ko00500, ko00940, ko01100, ko01110, map00460, map00500, map00940, map01100, map01110
FUN_004148	Amidohydrolase	-	-	-	-
FUN_004149	DNA polymerase	POL3	2.7.7.7	ko:K02327	ko00230, ko00240, ko01100, ko03030, ko03410, ko03420, ko03430, ko03440, ko05166, map00230, map00240, map01100, map03030, map03410, map03420, map03430, map03440, map05166
FUN_004150	Copper-translocating P-type ATPase	-	3.6.3.4	ko:K01533	-
FUN_004153	NAD dependent epimerase/dehydratase family	-	-	-	-
FUN_004154	Chitosanase catalyzing the endo-type cleavage of chitosan, the deacylated form of chitin. Chitosanase may be crucial in the degradation of the deacetylated portion of chitin in the fungal cell wall	csn	3.2.1.132	ko:K01233	ko00520, ko01100, map00520, map01100
FUN_004155	NAD(P)H-binding	-	-	-	-
FUN_004157	Membrane bound O-acyl transferase family	-	-	-	-
FUN_004158	RNA pol II promoter Fmp27 protein domain	FMP27	-	-	-
FUN_004159	Belongs to the GMC oxidoreductase family	-	-	-	-
FUN_004160	FAD dependent oxidoreductase	-	-	-	-
FUN_004161	Belongs to the glycosyl hydrolase 43 family	-	3.2.1.37	ko:K01198	ko00520, ko01100, map00520, map01100
FUN_004162	Belongs to the glycosyl hydrolase 12 (cellulase H) family	-	3.2.1.151	ko:K18576	-
FUN_004163	Belongs to the isocitrate lyase PEP mutase superfamily. Isocitrate lyase family	-	4.1.3.1	ko:K01637	ko00630, ko01100, ko01110, ko01120, ko01200, map00630, map01100, map01110, map01120, map01200
FUN_004164	Major Facilitator Superfamily	-	-	-	-
FUN_004165	peptidase activity	-	-	-	-
FUN_004166	Aminotransferase class I and II	YEY2	-	-	-
FUN_004167	5-oxoprolinase	-	3.5.2.9	ko:K01469	ko00480, map00480
FUN_004168	Serine carboxypeptidase	-	-	-	-
FUN_004171	Belongs to the iron ascorbate-dependent oxidoreductase family	-	-	-	-
FUN_004173	cytochrome P450	-	-	-	-
FUN_004174	cytochrome P450	-	-	-	-
FUN_004175	Glycoside hydrolase family 20 protein	-	3.2.1.52	ko:K12373	ko00511, ko00513, ko00520, ko00531, ko00603, ko00604, ko01100, ko04142, map00511, map00513, map00520, map00531, map00603, map00604, map01100, map04142
FUN_004177	Enoyl-(Acyl carrier protein) reductase	-	-	-	-

FUN_004178	Permease for cytosine/purines, uracil, thiamine, allantoin	-	-	ko:K03457	-
FUN_004179	Belongs to the iron ascorbate-dependent oxidoreductase family	-	-	-	-
FUN_004182	Belongs to the type-B carboxylesterase lipase family	-	-	-	-
FUN_004185	Amino acid transporter	-	-	-	-
FUN_004186	Domain of unknown function (DUF4188)	-	-	-	-
FUN_004187	Cys/Met metabolism PLP-dependent enzyme	-	2.3.1.47	ko:K00652	ko00780, ko01100, map00780, map01100
FUN_004188	polyketide synthase	-	-	-	-
FUN_004189	Fungal trichothecene efflux pump (TRI12)	-	-	-	-
FUN_004190	Belongs to the cytochrome P450 family	-	1.14.13.12	ko:K07824	ko00362, ko00627, ko01100, ko01120, ko01220, map00362, map00627, map01100, map01120, map01220
FUN_004192	Polysaccharide biosynthesis protein	-	-	-	-
FUN_004193	Haloacid dehalogenase, type II	-	-	-	-
FUN_004194	Belongs to the type-B carboxylesterase lipase family	-	-	-	-
FUN_004195	Lysine methyltransferase	-	-	-	-
FUN_004196	Extracellular tail, of 10TM putative phosphate transporter	PHM7	-	ko:K21989	-
FUN_004198	Peroxin 26	-	-	-	-
FUN_004199	CAF1 family ribonuclease	-	3.1.13.4	ko:K01148, ko:K17427	ko03018, map03018
FUN_004201	Major Facilitator Superfamily	-	-	ko:K08157	-
FUN_004202	GDSL-like Lipase/Acylhydrolase family	-	-	-	-
FUN_004203	Transcription factor	-	-	ko:K21752	-
FUN_004204	Phosphate acyltransferases	TAZ1	-	ko:K13511	ko00564, map00564
FUN_004205	C6 zinc finger	-	-	-	-
FUN_004206	Provides the precursors necessary for DNA synthesis. Catalyzes the biosynthesis of deoxyribonucleotides from the corresponding ribonucleotides	RNR1	1.17.4.1	ko:K10807	ko00230, ko00240, ko00480, ko00983, ko01100, map00230, map00240, map00480, map00983, map01100
FUN_004207	Ring finger	-	-	ko:K13125	-
FUN_004210	50S ribosome-binding GTPase	mtg1	-	ko:K19828	-
FUN_004211	KR domain	-	-	-	-
FUN_004213	Putative GTP-ase activating proteins for the small GTPase, ARF	GCS1	-	ko:K12492	ko04144, map04144
FUN_004214	WSC domain	-	-	ko:K11244	ko04011, ko04139, map04011, map04139
FUN_004215	Enoyl-(Acyl carrier protein) reductase	SPS19	1.3.1.34	ko:K13237	ko04146, map04146
FUN_004217	Belongs to the mitochondrial carrier (TC 2.A.29) family	SFC1	-	ko:K15100	-
FUN_004218	Zn_pept	ECM14	-	ko:K08783	-
FUN_004219	Sec7 domain	-	-	-	-
FUN_004220	Belongs to the glycosyl hydrolase 47 family	-	3.2.1.113	ko:K01230	ko00510, ko00513, ko01100, ko04141, map00510, map00513, map01100, map04141
FUN_004222	Putative RRM domain	-	-	-	-
FUN_004223	Glycosyltransferase family 57 protein	ALG8	2.4.1.265	ko:K03849	ko00510, ko01100, map00510, map01100
FUN_004224	SCA7, zinc-binding domain	sgf73	-	ko:K11365	-
FUN_004225	Pfam:DUF1762	-	-	-	-
FUN_004226	Nucleoside transporter	-	-	ko:K15014	-
FUN_004229	C-terminal domain of 1-Cys peroxiredoxin	-	1.11.1.15	ko:K03386	ko04214, map04214

FUN_004230	Zinc finger, C3HC4 type (RING finger)	-	-	-	-
FUN_004231	Nudix domain-containing protein	-	-	-	-
FUN_004234	NACHT domain	-	-	-	-
FUN_004235	Protein tyrosine kinase	-	2.1.1.205	ko:K08286, ko:K14864	-
FUN_004237	Belongs to the small GTPase superfamily. Rho family	cdc42	-	ko:K04392, ko:K04393	ko04010, ko04011, ko04013, ko04014, ko04015, ko04024, ko04062, ko04071, ko04144, ko04145, ko04151, ko04310, ko04360, ko04370, ko04380, ko04510, ko04520, ko04530, ko04620, ko04650, ko04660, ko04662, ko04664, ko04666, ko04670, ko04722, ko04810, ko04912, ko04932, ko04933, ko04972, ko05014, ko05100, ko05120, ko05130, ko05131, ko05132, ko05165, ko05167, ko05200, ko05203, ko05205, ko05210, ko05211, ko05212, ko05231, ko05416, ko05418, map04010, map04011, map04013, map04014, map04015, map04024, map04062, map04071, map04144, map04145, map04151, map04310, map04360, map04370, map04380, map04510, map04520, map04530, map04620, map04650, map04660, map04662, map04664, map04666, map04670, map04722, map04810, map04912, map04932, map04933, map04972, map05014, map05100, map05120, map05130, map05131, map05132, map05165, map05167, map05200, map05203, map05205, map05210, map05211, map05212, map05231, map05416, map05418
FUN_004238	GAL4-like Zn(II)2Cys6 (or C6 zinc) binuclear cluster DNA-binding domain	-	-	-	-
FUN_004239	Glycoside hydrolase family 3 protein	-	3.2.1.21	ko:K05349	ko00460, ko00500, ko00940, ko01100, ko01110, map00460, map00500, map00940, map01100, map01110
FUN_004240	Carbamoyl-phosphate synthase arginine-specific small chain	CPA1	6.3.5.5	ko:K01956	ko00240, ko00250, ko01100, map00240, map00250, map01100
FUN_004243	Domain of unknown function (DUF4782)	-	-	-	-
FUN_004244	SAP domain	-	-	ko:K18732	-
FUN_004246	Alcohol dehydrogenase GroES-like domain	-	-	-	-
FUN_004247	Choline dehydrogenase	-	1.1.99.1	ko:K00108	ko00260, ko01100, map00260, map01100
FUN_004249	Alpha/beta hydrolase family	-	-	-	-
FUN_004251	Zinc finger C-x8-C-x5-C-x3-H type (and similar)	CWC24	2.3.1.258	ko:K13127, ko:K20793	-
FUN_004252	Meiotic cell cortex C-terminal pleckstrin homology	-	-	ko:K17978	-
FUN_004253	Isy1-like splicing family	ISY1	-	ko:K12870	ko03040, map03040
FUN_004254	39S mitochondrial ribosomal protein L46	-	-	ko:K17427	-
FUN_004255	TCP-1/cpn60 chaperonin family	CCT8	-	ko:K09500	-
FUN_004257	Belongs to the cation transport ATPase (P-type) (TC 3.A.3) family. Type IV subfamily	DRS2	3.6.3.1	ko:K14802	-
FUN_004258	NmrA-like family	-	-	-	-
FUN_004259	Protein of unknown function (DUF3445)	-	-	-	-
FUN_004260	Pyridine nucleotide-disulphide oxidoreductase	-	-	-	-
FUN_004263	Maintenance of mitochondrial structure and function	-	-	ko:K12179	-

FUN_004264	Alcohol dehydrogenase GroES-like domain	-	1.1.1.14	ko:K00008	ko00040, ko00051, ko01100, map00040, map00051, map01100
FUN_004265	alpha/beta hydrolase fold	-	3.1.3.76, 3.3.2.10	ko:K08726	ko00590, ko00625, ko01100, ko01120, ko04146, map00590, map00625, map01100, map01120, map04146
FUN_004266	pinin/SDK/memA/ protein conserved region	-	-	-	-
FUN_004267	PHF5-like protein	ini1	-	ko:K12834	ko03040, map03040
FUN_004268	Autophagy-related protein 3	ATG3	-	ko:K08343	ko04136, ko04138, ko04140, ko05167, map04136, map04138, map04140, map05167
FUN_004269	DNA polymerase X family	-	2.7.7.7	ko:K03512, ko:K03513, ko:K10981	ko03410, ko03450, map03410, map03450
FUN_004271	Ca2+ insensitive EF hand	CMD1	-	ko:K02183	ko04014, ko04015, ko04016, ko04020, ko04022, ko04024, ko04070, ko04114, ko04218, ko04261, ko04270, ko04371, ko04626, ko04713, ko04720, ko04722, ko04728, ko04740, ko04744, ko04745, ko04750, ko04910, ko04912, ko04915, ko04916, ko04921, ko04922, ko04924, ko04925, ko04970, ko04971, ko05010, ko05031, ko05034, ko05133, ko05152, ko05167, ko05200, ko05214, ko05418, map04014, map04015, map04016, map04020, map04022, map04024, map04070, map04114, map04218, map04261, map04270, map04371, map04626, map04713, map04720, map04722, map04728, map04740, map04744, map04745, map04750, map04910, map04912, map04915, map04916, map04921, map04922, map04924, map04925, map04970, map04971, map05010, map05031, map05034, map05133, map05152, map05167, map05200, map05214, map05418
FUN_004272	Belongs to the syntaxin family	tlg2	-	ko:K08489	ko04130, map04130
FUN_004274	ThiF family	-	-	ko:K22132	-
FUN_004275	DNA/RNA non-specific endonuclease	NUC1	-	ko:K01173	ko04210, map04210
FUN_004276	Legume-like lectin family	-	-	ko:K10082	ko04141, map04141
FUN_004277	Common central domain of tyrosinase	-	1.14.18.1	ko:K00505	ko00350, ko00950, ko00965, ko01100, ko01110, ko04916, map00350, map00950, map00965, map01100, map01110, map04916
FUN_004278	Transmembrane alpha-helix domain-containing protein	-	-	-	-
FUN_004279	Nucleolar complex-associated protein	-	-	ko:K14834	-
FUN_004280	Required for proper 27S pre-rRNA processing and 60S ribosome subunit assembly	NIP7	-	ko:K07565	-
FUN_004283	Catalyzes the reversible decarboxylation of aromatic carboxylic acids like ferulic acid, p-coumaric acid or cinnamic acid, producing the corresponding vinyl derivatives 4-vinylphenol, 4-vinylguaiacol, and styrene, respectively, which play the role of aroma metabolites	FDC1	4.1.1.102	ko:K20039	ko00940, map00940
FUN_004284	Flavin prenyltransferase that catalyzes the synthesis of the prenylated FMN cofactor (prenyl-FMN) for the ferulic acid decarboxylase FDC1. The prenyltransferase is metal-independent and links a dimethylallyl moiety from dimethylallyl monophosphate (DMAP) to the flavin N5 and C6 atoms of FMN	PAD1	2.5.1.129	ko:K03186	ko00130, ko00627, ko00940, ko01100, ko01110, ko01120, ko01220, map00130, map00627, map00940, map01100, map01110, map01120, map01220
FUN_004286	Cwf15/Cwc15 cell cycle control protein	CWC15	-	ko:K12863	ko03040, map03040
FUN_004287	3' exoribonuclease family, domain 2	RRP45	-	ko:K03678	ko03018, map03018

FUN_004288	Domain of unknown function (DUF4336)	-	-	-	-
FUN_004289	Retinoic acid induced 16-like protein	-	-	-	-
FUN_004290	Uncharacterised ACR, YagE family COG1723	RMD1	-	-	-
FUN_004291	Belongs to the PI3 PI4-kinase family	PIK1	2.7.1.67	ko:K19801	ko00562, ko01100, ko04070, map00562, map01100, map04070
FUN_004292	Pyridine nucleotide-disulphide oxidoreductase	-	-	-	-
FUN_004294	Alkaline phosphatase	-	3.1.3.1	ko:K01077	ko00730, ko00790, ko01100, ko02020, map00730, map00790, map01100, map02020
FUN_004295	Fungal-type cellulose-binding domain	-	-	-	-
FUN_004296	cytochrome P-450	-	-	-	-
FUN_004297	Belongs to the glycosyl hydrolase 28 family	-	3.2.1.67	ko:K01213	ko00040, ko01100, map00040, map01100
FUN_004302	Amidase	-	-	-	-
FUN_004303	Belongs to the WD repeat coronin family	CRN1	-	ko:K13886	-
FUN_004305	Heterokaryon incompatibility protein (HET)	-	-	-	-
FUN_004306	FAD binding domain	-	1.14.13.1	ko:K00480	ko00621, ko00624, ko00626, ko01100, ko01120, ko01220, map00621, map00624, map00626, map01100, map01120, map01220
FUN_004307	Nse4 C-terminal	-	-	-	-
FUN_004308	Belongs to the PI3 PI4-kinase family	VPS34	2.7.1.137	ko:K00914	ko00562, ko01100, ko04070, ko04136, ko04138, ko04140, ko04145, ko04371, ko05152, ko05167, map00562, map01100, map04070, map04136, map04138, map04140, map04145, map04371, map05152, map05167
FUN_004309	Protein of unknown function (DUF2439)	-	-	-	-
FUN_004310	BAR domain	-	-	-	-
FUN_004314	Heterokaryon incompatibility protein (HET)	-	-	-	-
FUN_004316	Calcineurin-like phosphoesterase	-	3.6.1.41	ko:K01525	ko00230, map00230
FUN_004318	Belongs to the short-chain dehydrogenases reductases (SDR) family	-	-	-	-
FUN_004322	occurring C-terminal to leucine-rich repeats	sds22	-	ko:K17550	-
FUN_004323	catalytic domain of ctd-like phosphatases	-	3.1.3.16	ko:K15731	-
FUN_004324	Phosphate acyltransferases	vps66	-	-	-
FUN_004325	Belongs to the TRAFAC class myosin-kinesin ATPase superfamily. Kinesin family	KAR3	-	ko:K10405, ko:K17945	-
FUN_004326	Catalyzes the ATP-dependent amination of UTP to CTP with either L-glutamine or ammonia as the source of nitrogen	URA7	6.3.4.2	ko:K01937	ko00240, ko01100, map00240, map01100
FUN_004327	Velvet factor	-	-	-	-
FUN_004328	Bicoid-interacting protein 3 (Bin3)	-	-	ko:K15190	-
FUN_004329	Protein of unknown function (DUF1349)	-	-	-	-
FUN_004331	Squalene epoxidase	ERG1	1.14.14.17	ko:K00511	ko00100, ko00909, ko01100, ko01110, ko01130, map00100, map00909, map01100, map01110, map01130
FUN_004333	NUDIX domain	YSA1	3.6.1.13	ko:K01515	ko00230, map00230
FUN_004334	Belongs to the cytochrome P450 family	-	1.14.14.1	ko:K00493	ko00071, ko00380, ko00627, ko01120, map00071, map00380, map00627, map01120
FUN_004336	Mitotic checkpoint regulator, MAD2B-interacting	-	-	-	-
FUN_004337	SAPK-interacting protein 1 (Sin1), Pleckstrin-homology	AVO1	-	ko:K20410	ko04150, map04150
FUN_004338	Belongs to the peptidase C19 family	-	3.4.19.12	ko:K11835	-

FUN_004339	Belongs to the peptidase C19 family	-	3.4.19.12	ko:K11835	-
FUN_004340	Voltage gated chloride channel	-	-	ko:K05012	-
FUN_004341	Coiled-coil domain-containing protein 55 (DUF2040)	-	-	-	-
FUN_004342	to Saccharomyces cerevisiae YKR041W	-	-	-	-
FUN_004343	Belongs to the glycosyl hydrolase 11 (cellulase G) family	-	3.2.1.8	ko:K01181	-
FUN_004345	Flavonol synthase	-	1.14.11.13	ko:K04125	ko00904, ko01110, map00904, map01110
FUN_004346	TAP-like protein	-	-	-	-
FUN_004347	Proline racemase	-	4.2.1.77	ko:K18384	ko00330, map00330
FUN_004348	GMC oxidoreductase	-	1.1.99.1	ko:K00108	ko00260, ko01100, map00260, map01100
FUN_004350	Pyridine nucleotide-disulphide oxidoreductase	-	-	-	-
FUN_004354	oligopeptide transporter	PTR2	-	ko:K03305	-
FUN_004362	Isochorismatase family	-	-	-	-
FUN_004366	zinc finger	-	-	-	-
FUN_004367	Electron transfer flavoprotein domain	CIR1	-	ko:K03521	-
FUN_004368	Component of the cytosolic iron-sulfur (Fe-S) protein assembly (CIA) machinery. Required for the maturation of extramitochondrial Fe-S proteins. Part of an electron transfer chain functioning in an early step of cytosolic Fe-S biogenesis. Transfers electrons from NADPH to the Fe-S cluster of DRE2. Positively controls H(2)O(2)-	TAH18	-	-	-
FUN_004369	RNA recognition motif	-	-	-	-
FUN_004370	Belongs to the iron ascorbate-dependent oxidoreductase family	-	-	-	-
FUN_004371	ZIP Zinc transporter	-	-	ko:K14715	-
FUN_004372	Catalytic component of the histone acetylase B (HAT-B) complex. Has intrinsic substrate specificity that modifies lysine in recognition sequence GXGKXG. Involved in DNA double-strand break repair	HAT1	2.3.1.48	ko:K11303	ko05034, map05034
FUN_004373	Formyl transferase	ade5	2.1.2.2	ko:K00601	ko00230, ko00670, ko01100, ko01110, ko01130, map00230, map00670, map01100, map01110, map01130
FUN_004375	Belongs to the NiCoT transporter (TC 2.A.52) family	-	-	ko:K07241	-
FUN_004376	Belongs to the beta-ketoacyl-ACP synthases family	CEM1	2.3.1.179	ko:K09458	ko00061, ko00780, ko01100, ko01212, map00061, map00780, map01100, map01212
FUN_004377	alpha/beta hydrolase fold	-	2.3.1.31	ko:K00641	ko00270, ko01100, ko01130, map00270, map01100, map01130
FUN_004380	Emopamil binding protein	-	-	-	-
FUN_004381	Carbohydrate-binding module family 21 protein	-	-	-	-
FUN_004382	HEAT repeat protein	-	-	ko:K20403	ko04150, map04150
FUN_004383	sister chromatid cohesion	PDS5	-	ko:K11267	-
FUN_004384	Ubiquinol-cytochrome C chaperone	cbp3	2.3.1.179	ko:K09458, ko:K17662	ko00061, ko00780, ko01100, ko01212, map00061, map00780, map01100, map01212
FUN_004385	Second step of mRNA capping. Transfer of the GMP moiety of GTP to the 5'-end of RNA via an enzyme-GMP covalent reaction intermediate	CEG1	2.7.7.50	ko:K00987	-
FUN_004387	Pumilio-family RNA binding repeat	-	-	ko:K17974	-
FUN_004388	YL1 nuclear protein	-	-	ko:K11664	-
FUN_004389	Belongs to the eukaryotic ribosomal protein eS24 family	-	-	ko:K02974	ko03010, map03010
FUN_004390	Ribosomal protein L11 methyltransferase (PrmA)	-	-	-	-
FUN_004392	Zinc-binding domain, present in Dystrophin, CREB-binding protein.	-	2.3.2.27	ko:K10644	-

FUN_004394	Belongs to the class VI-like SAM-binding methyltransferase superfamily. Isoprenylcysteine carboxyl methyltransferase family	STE14	2.1.1.100	ko:K00587	ko00900, ko01130, map00900, map01130
FUN_004398	Glycoside hydrolase family 17 protein	-	3.2.1.39	ko:K01199	ko00500, map00500
FUN_004399	Dehydratase family	ILV3	4.2.1.9	ko:K01687	ko00290, ko00770, ko01100, ko01110, ko01130, ko01210, ko01230, map00290, map00770, map01100, map01110, map01130, map01210, map01230
FUN_004401	Fungal specific transcription factor domain	-	-	-	-
FUN_004402	G-protein coupled peptide receptor activity	-	-	-	-
FUN_004403	Belongs to the iron ascorbate-dependent oxidoreductase family	-	-	-	-
FUN_004404	Acyl-coenzyme A:6-aminopenicillanic acid acyl-transferase	-	2.3.1.164, 3.4.14.9	ko:K01279, ko:K10852, ko:K19200	ko00311, ko01100, ko01130, ko04142, map00311, map01100, map01130, map04142
FUN_004405	Uncharacterized protein conserved in bacteria (DUF2334)	-	-	-	-
FUN_004406	Amino acid permease	-	-	ko:K16261	-
FUN_004410	Cerato-platanin	-	-	-	-
FUN_004412	NAD(P)-binding Rossmann-like domain	-	-	-	-
FUN_004417	Zinc-binding dehydrogenase	-	-	-	-
FUN_004418	dihydrokaempferol 4-reductase activity	-	-	ko:K20308	-
FUN_004420	FAD binding	-	-	-	-
FUN_004422	paf acetylhydrolase family protein	-	-	-	-
FUN_004424	mRNA binding	NSRP1	3.4.17.22, 3.6.4.12	ko:K07752, ko:K11414, ko:K13206, ko:K14437, ko:K20526	-
FUN_004425	1-deoxy-D-xylulose-5-phosphate synthase	-	2.2.1.3	ko:K17100	ko00680, ko01120, ko01200, map00680, map01120, map01200
FUN_004426	Belongs to the glycosyl hydrolase 3 family	-	3.2.1.21	ko:K05349	ko00460, ko00500, ko00940, ko01100, ko01110, map00460, map00500, map00940, map01100, map01110
FUN_004427	Membrane transport protein	-	-	-	-
FUN_004428	alpha/beta hydrolase fold	-	-	-	-
FUN_004430	Belongs to the pyrroline-5-carboxylate reductase family	PRO3	1.5.1.2	ko:K00286	ko00330, ko01100, ko01110, ko01130, ko01230, map00330, map01100, map01110, map01130, map01230
FUN_004431	CTD kinase subunit gamma CTK3 C-terminus	-	-	ko:K15565	-
FUN_004432	Ribosomal protein L7/L12 dimerisation domain	MNP1	-	ko:K02935	ko03010, map03010
FUN_004433	Nuclear chaperone required for maturation and nuclear export of pre-60S ribosome subunits	MDN1	-	ko:K14572	ko03008, map03008
FUN_004439	Steryl acetyl hydrolase	-	-	-	-
FUN_004441	Ferritin-like domain	-	-	-	-
FUN_004443	Phenazine biosynthesis-like protein	-	-	-	-
FUN_004444	Lipase (class 3)	-	-	-	-

FUN_004445	Extension to Ser/Thr-type protein kinases	TPK2	2.7.11.11	ko:K04345	ko01522, ko04010, ko04014, ko04020, ko04024, ko04062, ko04113, ko04114, ko04138, ko04140, ko04211, ko04213, ko04261, ko04270, ko04310, ko04340, ko04341, ko04371, ko04530, ko04540, ko04611, ko04713, ko04714, ko04720, ko04723, ko04724, ko04725, ko04726, ko04727, ko04728, ko04740, ko04742, ko04750, ko04910, ko04911, ko04912, ko04913, ko04914, ko04915, ko04916, ko04918, ko04919, ko04921, ko04922, ko04923, ko04924, ko04925, ko04926, ko04927, ko04934, ko04961, ko04962, ko04970, ko04971, ko04976, ko05012, ko05020, ko05030, ko05031, ko05032, ko05110, ko05146, ko05165, ko05166, ko05169, ko05200, ko05203, ko05205, ko05414, map01522, map04010, map04014, map04020, map04024, map04062, map04113, map04114, map04138, map04140, map04211, map04213, map04261, map04270, map04310, map04340, map04341, map04371, map04530, map04540, map04611, map04713, map04714, map04720, map04723, map04724, map04725, map04726, map04727, map04728, map04740, map04742, map04750, map04910, map04911, map04912, map04913, map04914, map04915, map04916, map04918, map04919, map04921, map04922, map04923, map04924, map04925, map04926, map04927, map04934, map04961, map04962, map04970, map04971, map04976, map05012, map05020, map05030, map05031, map05032, map05110, map05146, map05165, map05166, map05169, map05200, map05203, map05205, map05414
FUN_004446	Glycosyltransferase family 8 protein	-	-	-	-
FUN_004447	PRP38 family	-	-	ko:K12849	ko03040, map03040
FUN_004448	Major Facilitator Superfamily	-	-	-	-
FUN_004450	Belongs to the zinc-containing alcohol dehydrogenase family	GEF2	-	ko:K05012	-
FUN_004452	Belongs to the zinc-containing alcohol dehydrogenase family	GEF2	-	ko:K05012	-
FUN_004454	Major Facilitator Superfamily	-	-	ko:K02575	ko00910, map00910
FUN_004456	Clr5 domain	-	-	-	-
FUN_004457	Sugar (and other) transporter	-	-	-	-
FUN_004458	Heterokaryon incompatibility protein (HET)	-	-	-	-
FUN_004459	CoA-transferase family III	-	-	-	-
FUN_004460	integral membrane protein	-	-	-	-
FUN_004463	FAD binding domain	-	-	-	-
FUN_004465	Enoyl-(Acyl carrier protein) reductase	-	-	-	-
FUN_004471	Belongs to the cation transport ATPase (P-type) (TC 3.A.3) family	ENA2	3.6.3.7	ko:K01536	-
FUN_004472	Epoxide hydrolase N terminus	-	-	-	-
FUN_004473	Domain in Tre-2, BUB2p, and Cdc16p. Probable Rab-GAPs.	-	-	ko:K20133	-
FUN_004475	RNA polymerase II transcription factor SIII (Elongin) subunit A	-	-	ko:K15077	-
FUN_004476	Tho complex subunit 7	-	-	ko:K13176	ko03013, map03013
FUN_004477	Belongs to the TBCA family	RBL2	-	ko:K17292	-

FUN_004478	Belongs to the MCM family	CDC54	3.6.4.12	ko:K02212	ko03030, ko04110, ko04111, ko04113, map03030, map04110, map04111, map04113
FUN_004479	Catalyzes the NADPH-dependent reduction of ketopantoate into pantoic acid	-	1.1.1.169	ko:K00077	ko00770, ko01100, ko01110, map00770, map01100, map01110
FUN_004481	Amino acid permease	LYP1	-	ko:K16261	-
FUN_004483	Esterase PHB depolymerase	-	3.1.1.72	ko:K05972	-
FUN_004485	Bestrophin, RFP-TM, chloride channel	-	-	ko:K08994	-
FUN_004486	Tetratricopeptide repeat	-	-	-	-
FUN_004488	)transporter	-	-	-	-
FUN_004489	DNA polymerase alpha/epsilon subunit B	DPB2	2.7.7.7	ko:K02325	ko00230, ko00240, ko01100, ko03030, ko03410, ko03420, ko05166, map00230, map00240, map01100, map03030, map03410, map03420, map05166
FUN_004490	SHNi-TPR	-	-	ko:K11372	-
FUN_004491	NAD(P)H-binding	-	-	-	-
FUN_004493	alpha/beta hydrolase fold	-	-	-	-
FUN_004494	Dihydrodipicolinate synthetase family	-	4.1.2.53	ko:K18339	ko00051, ko01120, map00051, map01120
FUN_004495	Mandelate racemase muconate lactonizing	-	4.2.1.6	ko:K01684	ko00052, ko01100, ko01120, map00052, map01100, map01120
FUN_004496	Fungal specific transcription factor domain	-	-	-	-
FUN_004501	Membrane-associating domain	-	-	-	-
FUN_004504	RNA recognition motif	-	-	-	-
FUN_004505	RNA-binding, Nab2-type zinc finger	-	-	-	-
FUN_004506	Catalyzes the 6-electron oxidation of protoporphyrinogen-IX to form protoporphyrin-IX	HEM14	1.3.3.15, 1.3.3.4	ko:K00231	ko00860, ko01100, ko01110, map00860, map01100, map01110
FUN_004508	integral membrane protein	-	-	-	-
FUN_004509	Alpha/beta hydrolase family	-	-	-	-
FUN_004511	Flavin containing amine oxidoreductase	-	-	-	-
FUN_004512	Major Facilitator Superfamily	-	-	-	-
FUN_004515	Glycoside hydrolase family 131 protein	-	-	-	-
FUN_004516	Catalyzes the NADPH-dependent reduction of ketopantoate into pantoic acid	-	-	-	-
FUN_004517	Belongs to the eukaryotic ribosomal protein eL42 family	-	-	ko:K02929	ko03010, map03010
FUN_004518	Endonuclease that specifically degrades the RNA of RNA- DNA hybrids	-	3.1.26.4	ko:K03469	ko03030, map03030
FUN_004519	HeH/LEM domain	SRC1	-	-	-
FUN_004526	Thioesterase superfamily	-	-	ko:K17362	-
FUN_004527	Sorting nexin C terminal	-	-	-	-
FUN_004528	Chromosome segregation atpase	-	-	-	-
FUN_004529	PCI domain	RPN6	-	ko:K03036	ko03050, ko05169, map03050, map05169
FUN_004531	Vacuolar protein sorting-associated protein 62	-	-	-	-
FUN_004532	DNA-dependent RNA polymerase catalyzes the transcription of DNA into RNA using the four ribonucleoside triphosphates as substrates	RPA190	2.7.7.6	ko:K02999	ko00230, ko00240, ko01100, ko03020, map00230, map00240, map01100, map03020
FUN_004533	pre-mRNA processing factor 3 (PRP3)	prp3	-	ko:K12843	ko03040, map03040
FUN_004536	Belongs to the cytochrome P450 family	-	-	-	-
FUN_004537	Glycoside hydrolase family 20 protein	NAG1	3.2.1.52	ko:K12373	ko00511, ko00513, ko00520, ko00531, ko00603, ko00604, ko01100, ko04142, map00511, map00513, map00520, map00531, map00603, map00604, map01100, map04142

FUN_004538	Glycoside hydrolase family 16 protein	CRH1	3.2.1.73	ko:K01216	-
FUN_004539	Aldehyde dehydrogenase family	PRO2	1.2.1.41	ko:K00147	ko00330, ko00332, ko01100, ko01110, ko01130, ko01230, map00330, map00332, map01100, map01110, map01130, map01230
FUN_004540	Flavin containing amine oxidoreductase	-	-	-	-
FUN_004541	Triose-phosphate Transporter family	-	-	-	-
FUN_004542	Ribosomal protein 60S L18 and 50S L18e	-	-	ko:K02883	ko03010, map03010
FUN_004543	AIR carboxylase	-	4.1.1.21	ko:K11808	ko00230, ko01100, ko01110, map00230, map01100, map01110
FUN_004544	Patatin-like phospholipase	-	-	-	-
FUN_004546	O-methyltransferase	-	-	-	-
FUN_004552	Triose-phosphate Transporter family	-	-	ko:K15280	-
FUN_004553	Mo25-like	HYM1	-	ko:K08272	ko04150, ko04152, map04150, map04152
FUN_004554	Protein tyrosine kinase	CSK1	2.7.11.22	ko:K06667, ko:K07760	ko04111, map04111
FUN_004555	Muniscin C-terminal mu homology domain	SYP1	-	ko:K20042	-
FUN_004556	PAS fold	-	-	-	-
FUN_004557	Peptidylprolyl isomerase	-	-	-	-
FUN_004560	MAP kinase kinase kinase activity	-	-	-	-
FUN_004563	WD40 repeats	UTP18	-	ko:K14553	ko03008, map03008
FUN_004564	salicylate hydroxylase	-	1.14.13.1	ko:K00480	ko00621, ko00624, ko00626, ko01100, ko01120, ko01220, map00621, map00624, map00626, map01100, map01120, map01220
FUN_004566	Calponin homology domain	fim1	-	ko:K17275	-
FUN_004567	Binds the StuA-response elements (StRE) with the consensus sequence 5'-(A T)CGCG(T A)N(A C)-3' at the promoters of target genes (By similarity)	stuA	-	ko:K12763	ko04113, map04113
FUN_004568	WSC domain	-	-	-	-
FUN_004569	Ubiquitin-like modifier-activating enzyme ATG7 N-terminus	ATG7	3.5.4.12	ko:K01493, ko:K08337	ko00240, ko01100, ko04136, ko04138, ko04140, ko04216, map00240, map01100, map04136, map04138, map04140, map04216
FUN_004571	Sir2 family	HST2	-	ko:K11121, ko:K11412	ko00760, ko01100, ko04213, map00760, map01100, map04213
FUN_004572	Belongs to the MIP aquaporin (TC 1.A.8) family	-	-	-	-
FUN_004573	zinc finger	-	-	-	-
FUN_004574	DWNN	MPE1	-	ko:K15541	ko03015, map03015
FUN_004575	Hydantoinase B/oxoprolinase	-	3.5.2.9	ko:K01469	ko00480, map00480
FUN_004576	RNA recognition motif	-	-	ko:K14411	ko03015, map03015
FUN_004577	Domain in Tre-2, BUB2p, and Cdc16p. Probable Rab-GAPs.	GYP1	-	ko:K20360	-
FUN_004578	Putative cyclase	-	-	-	-
FUN_004579	WEE WEE-UNCLASSIFIED protein kinase	SWE1	2.7.11.1	ko:K03114	ko04011, ko04111, ko04113, map04011, map04111, map04113
FUN_004580	Saccharopine dehydrogenase NADP binding domain	-	-	-	-
FUN_004583	Alpha/beta hydrolase of unknown function (DUF1100)	-	-	-	-

FUN_004585	Belongs to the hexokinase family	hxk1	2.7.1.1	ko:K00844	ko00010, ko00051, ko00052, ko00500, ko00520, ko00521, ko00524, ko01100, ko01110, ko01120, ko01130, ko01200, ko04066, ko04910, ko04930, ko04973, ko05230, map00010, map00051, map00052, map00500, map00520, map00521, map00524, map01100, map01110, map01120, map01130, map01200, map04066, map04910, map04930, map04973, map05230
FUN_004586	DnaJ central domain	SCJ1	-	ko:K14002	ko04141, map04141
FUN_004587	Ribosomal protein S17	-	-	ko:K02961	ko03010, map03010
FUN_004588	Protein tyrosine kinase	CDC7	2.7.11.1	ko:K02214	ko04110, ko04111, ko04113, map04110, map04111, map04113
FUN_004589	Regulatory subunit of the condensin complex, a complex required for conversion of interphase chromatin into mitotic-like condense chromosomes. The condensin complex probably introduces positive supercoils into relaxed DNA in the presence of type I topoisomerases and converts nicked DNA into positive knotted forms in the presence of type II topoisomerases	cnd1	-	ko:K06677	ko04111, map04111
FUN_004590	Belongs to the cytochrome P450 family	-	1.14.13.78	ko:K21292	ko00904, ko01100, map00904, map01100
FUN_004591	Belongs to the cytochrome P450 family	-	1.14.13.174	ko:K17645	ko00254, map00254
FUN_004592	Acetyltransferase (GNAT) domain	-	2.3.1.256	ko:K00670	-
FUN_004594	Lecithin:cholesterol acyltransferase	LRO1	2.3.1.158	ko:K00679	ko00561, map00561
FUN_004596	Domain of unknown function (DUF3441)	-	-	-	-
FUN_004597	Clathrin adaptor complex small chain	RET3	-	ko:K20472	-
FUN_004599	Thioredoxin	-	-	ko:K03671	ko04621, ko05418, map04621, map05418
FUN_004600	Regulator of Vps4 activity in the MVB pathway	IST1	-	ko:K19476	ko04144, map04144
FUN_004601	Vacuolar segregation subunit 7	VAC7	-	-	-
FUN_004602	Serine aminopeptidase, S33	-	3.5.1.101	ko:K18457	-
FUN_004603	iron ion binding	-	-	-	-
FUN_004606	Belongs to the glycosyl hydrolase 32 family	INV1	3.2.1.26	ko:K01193	ko00052, ko00500, ko01100, map00052, map00500, map01100
FUN_004608	DDHD	-	-	-	-
FUN_004609	Occurs in almost all aerobically respiring organisms and serves to protect cells from the toxic effects of hydrogen peroxide	CAT1	1.11.1.6	ko:K03781	ko00380, ko00630, ko01110, ko01130, ko01200, ko04011, ko04016, ko04068, ko04146, ko04211, ko04212, ko04213, ko05014, map00380, map00630, map01110, map01130, map01200, map04011, map04016, map04068, map04146, map04211, map04212, map04213, map05014
FUN_004612	Putative esterase	-	-	-	-
FUN_004615	Ring finger	-	-	-	-
FUN_004616	Enoyl-(Acyl carrier protein) reductase	-	-	-	-
FUN_004617	Transcriptional Coactivator p15 (PC4)	-	-	-	-
FUN_004619	detection of bacterial lipoprotein	-	-	-	-
FUN_004620	Pfam:DUF1237	-	-	ko:K09704	-
FUN_004621	Glycoside hydrolase family 125 protein	COG4	-	ko:K20291	-
FUN_004622	Magnesium transporter NIPA	-	-	-	-
FUN_004623	Alpha/beta hydrolase family	-	-	-	-

FUN_004624	Sof1-like domain	sof1	-	ko:K11806	-
FUN_004625	Ribosomal L27e protein family	RPL27B	-	ko:K02901	ko03010, map03010
FUN_004626	Pathogenesis associated protein Cap20	-	-	-	-
FUN_004627	Component of the post-replicative DNA mismatch repair system (MMR)	msh2	-	ko:K08735, ko:K19476	ko01524, ko03430, ko04144, ko05200, ko05210, map01524, map03430, map04144, map05200, map05210
FUN_004628	Major Facilitator Superfamily	-	-	-	-
FUN_004629	Esterase lipase	-	-	-	-
FUN_004630	N-terminal domain of CBF1 interacting co-repressor CIR	-	-	-	-
FUN_004632	PRP1 splicing factor, N-terminal	prp1	-	ko:K12855	ko03040, map03040
FUN_004633	Transcriptional regulator of RNA polII, SAGA, subunit	-	-	ko:K11362	-
FUN_004634	Luciferase-like monooxygenase	-	-	-	-
FUN_004635	Pyridine nucleotide-disulphide oxidoreductase	-	-	-	-
FUN_004636	Ion channel regulatory protein UNC-93	-	-	-	-
FUN_004637	Zinc-binding dehydrogenase	-	-	-	-
FUN_004638	Luciferase-like monooxygenase	-	1.14.14.5	ko:K04091	ko00920, map00920
FUN_004640	Scaffold protein for the de novo synthesis of iron- sulfur (Fe-S) clusters within mitochondria, which is required for maturation of both mitochondrial and cytoplasmic 2Fe-2S and 4Fe-4S proteins	ISU1	-	ko:K22068	-
FUN_004641	Ubiquitinyl hydrolase 1	ubp14	3.4.19.12	ko:K11836	-
FUN_004642	DNA polymerase phi	POL5	2.7.7.7	ko:K02331	-
FUN_004643	Rhodanese Homology Domain	MIH1	3.1.3.48	ko:K02555	ko04011, ko04111, map04011, map04111
FUN_004644	The GINS complex plays an essential role in the initiation of DNA replication	SLD5	-	ko:K10735	-
FUN_004645	Dienelactone hydrolase family	-	-	-	-
FUN_004646	As part of the replication protein A (RPA RP-A), a single-stranded DNA-binding heterotrimeric complex, may play an essential role in DNA replication, recombination and repair. Binds and stabilizes single-stranded DNA intermediates, preventing complementary DNA reannealing and recruiting different proteins involved in DNA	RFA1	-	ko:K07466	ko03030, ko03420, ko03430, ko03440, ko03460, map03030, map03420, map03430, map03440, map03460
FUN_004647	Zinc-binding dehydrogenase	-	1.6.5.5	ko:K00344	-
FUN_004649	Glycoside hydrolase family 81 protein	ACF2	3.2.1.6	ko:K01180	-
FUN_004650	Pyridine nucleotide-disulphide oxidoreductase, dimerisation domain	-	-	-	-
FUN_004651	PAP2 superfamily	AUR1	-	-	-
FUN_004653	Belongs to the mitochondrial carrier (TC 2.A.29) family	-	-	ko:K15109	ko04714, map04714
FUN_004654	RNA recognition motif	GBP2	-	-	-
FUN_004655	Fungal trichothecene efflux pump (TRI12)	NAG4	-	-	-
FUN_004656	Fungal specific transcription factor domain	-	-	-	-
FUN_004657	Belongs to the short-chain dehydrogenases reductases (SDR) family	-	-	-	-
FUN_004659	Hinge domain of cleavage stimulation factor subunit 2	-	-	ko:K14407	ko03015, map03015
FUN_004660	amine oxidase	-	1.4.3.21	ko:K00276	ko00260, ko00350, ko00360, ko00410, ko00950, ko00960, ko01100, ko01110, map00260, map00350, map00360, map00410, map00950, map00960, map01100, map01110
FUN_004661	Glycoside hydrolase family 61 protein	-	-	-	-
FUN_004663	ABC-2 type transporter	-	-	-	-
FUN_004664	Fungal specific transcription factor domain	-	-	-	-

FUN_004665	Cleavage and polyadenylation factor 2 C-terminal	-	-	ko:K14402	ko03015, map03015
FUN_004666	Zinc finger C-x8-C-x5-C-x3-H type (and similar)	-	-	ko:K14404	ko03015, ko05164, map03015, map05164
FUN_004667	Dihydroorotase	URA4	3.5.2.3	ko:K01465	ko00240, ko01100, map00240, map01100
FUN_004668	Belongs to the class I-like SAM-binding methyltransferase superfamily. C5-methyltransferase family	-	2.1.1.37	ko:K00558	ko00270, ko01100, ko05206, map00270, map01100, map05206
FUN_004669	Protein of unknown function (DUF3431)	-	-	-	-
FUN_004671	Acetamidase/Formamidase family	-	3.5.1.49	ko:K01455	ko00460, ko00630, ko00910, ko01200, map00460, map00630, map00910, map01200
FUN_004672	Etoposide-induced protein 2.4 (EI24)	-	-	-	-
FUN_004673	Inheritance of peroxisomes protein 1	-	-	-	-
FUN_004674	Cytochrome domain of cellobiose dehydrogenase	-	-	-	-
FUN_004675	OST3 / OST6 family, transporter family	OST3	-	ko:K12669	ko00510, ko00513, ko01100, ko04141, map00510, map00513, map01100, map04141
FUN_004677	prohibitin homologues	-	-	-	-
FUN_004678	Belongs to the mitochondrial carrier (TC 2.A.29) family	-	-	ko:K14137, ko:K14684	-
FUN_004679	FtsJ-like methyltransferase	MRM2	2.1.1.166, 2.1.1.168	ko:K02427, ko:K15508	-
FUN_004681	Domain of unknwon function (DUF3824)	-	-	-	-
FUN_004682	Acyltransferase family	-	-	-	-
FUN_004683	Adaptins are components of the adaptor complexes which link clathrin to receptors in coated vesicles. Clathrin-associated protein complexes are believed to interact with the cytoplasmic tails of membrane proteins, leading to their selection and concentration	APL2	-	-	-
FUN_004684	VID27 cytoplasmic protein	vid27	2.1.1.221	ko:K15445	-
FUN_004687	Domain present in PSD-95, Dlg, and ZO-1/2.	NMA111	-	-	-
FUN_004688	Fungal specific transcription factor domain	-	-	-	-
FUN_004690	Cytochrome P450	-	-	-	-
FUN_004691	Alpha, alpha-trehalose-phosphate synthase UDP-forming	TPS1	2.4.1.15, 2.4.1.347	ko:K00697	ko00500, ko01100, map00500, map01100
FUN_004692	Transcriptional regulator	-	-	-	-
FUN_004693	malic enzyme	-	1.1.1.40	ko:K00029	ko00620, ko00710, ko01100, ko01120, ko01200, map00620, map00710, map01100, map01120, map01200
FUN_004695	Ppx/GppA phosphatase family	RTG2	-	ko:K11369	-
FUN_004696	NAD( )-dependent glutamate dehydrogenase which degrades glutamate to ammonia and alpha-ketoglutarate	GDH2	1.4.1.2	ko:K15371	ko00220, ko00250, ko00430, ko00910, ko01100, map00220, map00250, map00430, map00910, map01100
FUN_004698	GPN-loop GTPase 3	-	-	ko:K06883	-
FUN_004699	Catalyzes the formation of S-adenosylmethionine from methionine and ATP	SAM2	2.5.1.6	ko:K00789	ko00270, ko01100, ko01110, ko01230, map00270, map01100, map01110, map01230
FUN_004702	Rft protein	RFT1	-	ko:K06316	-
FUN_004703	Patatin-like phospholipase	-	-	-	-
FUN_004704	Domain of unknown function (DUF3554)	GCN1	-	-	-
FUN_004705	Helicase associated domain (HA2) Add an annotation	-	3.6.4.13	ko:K18995	-
FUN_004707	Gnat family	-	-	-	-
FUN_004708	Eukaryotic cytochrome b561	-	-	-	-

FUN_004709	Redoxin	-	1.11.1.15	ko:K11187	ko04146, map04146
FUN_004710	heat shock factor	SKN7	-	ko:K15859	ko02020, map02020
FUN_004711	eIF4-gamma/eIF5/eIF2-epsilon	GCD6	-	ko:K03240	ko03013, map03013
FUN_004712	Ubiquitin-conjugating enzyme E2	-	2.3.2.31	ko:K11968	-
FUN_004713	Conidiation protein 6	-	-	-	-
FUN_004714	Inositol monophosphatase family	-	3.1.3.25	ko:K01092	ko00521, ko00562, ko01100, ko04070, map00521, map00562, map01100, map04070
FUN_004715	Anticodon-binding domain	-	-	-	-
FUN_004716	Catalyzes the oxidative decarboxylation of 6- phosphogluconate to ribulose 5-phosphate and CO(2), with concomitant reduction of NADP to NADPH	GND1	1.1.1.343, 1.1.1.44	ko:K00033	ko00030, ko00480, ko01100, ko01110, ko01120, ko01130, ko01200, map00030, map00480, map01100, map01110, map01120, map01130, map01200
FUN_004717	RNA recognition motif	-	-	-	-
FUN_004718	Fungal domain of unknown function (DUF1750)	-	-	-	-
FUN_004719	Common central domain of tyrosinase	-	1.14.18.1	ko:K00505	ko00350, ko00950, ko00965, ko01100, ko01110, ko04916, map00350, map00950, map00965, map01100, map01110, map04916
FUN_004724	Cupin	-	-	-	-
FUN_004726	The coatomer is a cytosolic protein complex that binds to dilysine motifs and reversibly associates with Golgi non- clathrin-coated vesicles, which further mediate biosynthetic protein transport from the ER, via the Golgi up to the trans Golgi network	-	-	ko:K05236	-
FUN_004728	Tyrosyl-tRNA synthetase	TYS1	6.1.1.1	ko:K01866	ko00970, map00970
FUN_004729	Cytochrome P450	-	-	-	-
FUN_004730	C6 zinc finger domain-containing protein	-	-	-	-
FUN_004731	Glycosyltransferase family 24 protein	KRE5	-	ko:K11718	ko04141, map04141
FUN_004732	Essential subunit of the N-oligosaccharyl transferase (OST) complex which catalyzes the transfer of a high mannose oligosaccharide from a lipid-linked oligosaccharide donor to an asparagine residue within an Asn-X-Ser Thr consensus motif in nascent	OST1	-	ko:K12666	ko00510, ko00513, ko01100, ko04141, map00510, map00513, map01100, map04141
FUN_004733	Indigoidine synthase A like protein	-	2.7.1.83, 4.2.1.70	ko:K16329, ko:K16330	ko00240, map00240
FUN_004734	Indigoidine synthase A like protein	-	2.7.1.83, 4.2.1.70	ko:K16329, ko:K16330	ko00240, map00240
FUN_004736	Competence-damaged protein	-	-	-	-
FUN_004737	VTC domain	VTC4	-	-	-
FUN_004738	Oxidoreductase family, NAD-binding Rossmann fold	-	-	-	-
FUN_004739	Patatin-like phospholipase	-	-	-	-
FUN_004741	MOSC domain	-	-	ko:K07140	-
FUN_004742	Patatin-like phospholipase	-	2.1.2.1	ko:K00600	ko00260, ko00460, ko00630, ko00670, ko00680, ko01100, ko01110, ko01120, ko01130, ko01200, ko01230, ko01523, map00260, map00460, map00630, map00670, map00680, map01100, map01110, map01120, map01130, map01200, map01230, map01523
FUN_004743	Protein of unknown function (DUF3176)	-	-	-	-
FUN_004744	Ribosomal protein s17	-	-	-	-
FUN_004745	Phenazine biosynthesis-like protein	-	-	-	-
FUN_004747	brca1 c terminus	-	-	-	-

FUN_004748	Structure-specific nuclease with 5'-flap endonuclease and 5'-3' exonuclease activities involved in DNA replication and repair. During DNA replication, cleaves the 5'-overhanging flap structure that is generated by displacement synthesis when DNA polymerase encounters the 5'-end of a downstream Okazaki fragment. It enters the flap from the 5'-end and then tracks to cleave the flap base, leaving a nick for ligation. Also involved in the long patch base excision repair (LP-BER) pathway, by cleaving within the apurinic apyrimidinic (AP) site-terminated flap. Acts as a genome stabilization factor that prevents flaps from equilibrating into structurs that lead to duplications and deletions. Also possesses 5'-3' exonuclease activity on nicked or gapped double-stranded DNA, and exhibits RNase H activity. Also involved in replication and repair	FEN1	-	ko:K04799	ko03030, ko03410, ko03450, map03030, map03410, map03450
FUN_004749	glutaryl-CoA dehydrogenase	-	1.3.8.6	ko:K00252	ko00071, ko00310, ko00362, ko00380, ko01100, ko01120, ko01130, map00071, map00310, map00362, map00380, map01100, map01120, map01130
FUN_004750	Belongs to the ubiquitin-conjugating enzyme family	UBC4	2.3.2.23	ko:K06689	ko04013, ko04120, ko04141, ko04624, map04013, map04120, map04141, map04624
FUN_004751	Glycosyltransferase family 20 protein	TPS2	2.4.1.15, 3.1.3.12	ko:K16055	ko00500, ko01100, map00500, map01100
FUN_004752	Allows the formation of correctly charged Gln-tRNA(Gln) through the transamidation of misacylated Glu-tRNA(Gln) in the mitochondria. The reaction takes place in the presence of glutamine and ATP through an activated gamma-phospho-Glu- tRNA(Gln)	PET112	6.3.5.6, 6.3.5.7	ko:K02434	ko00970, ko01100, map00970, map01100
FUN_004754	Belongs to the universal ribosomal protein uL15 family	RPL28	3.6.4.13	ko:K02900, ko:K11273	ko03010, map03010
FUN_004755	Gelsolin repeat	SFB3	-	ko:K14007	ko04141, map04141
FUN_004756	Belongs to the adaptor complexes small subunit family	aps2	-	ko:K11827	ko04144, ko04721, ko04961, ko05016, map04144, map04721, map04961, map05016
FUN_004758	Glycoside hydrolase family 15 protein	SGA1	3.2.1.3	ko:K01178	ko00500, ko01100, map00500, map01100
FUN_004759	Clr5 domain	-	-	-	-
FUN_004760	Hemerythrin HHE cation binding domain	-	-	-	-
FUN_004761	Sister chromatid cohesion protein Dcc1	-	-	ko:K11271	-
FUN_004762	RhoGEF domain	-	-	-	-
FUN_004763	Prolyl-tRNA synthetase, C-terminal	-	6.1.1.15	ko:K01881	ko00970, map00970
FUN_004764	assists the folding of proteins upon ATP hydrolysis	cct1	-	ko:K09493	-
FUN_004765	PI31 proteasome regulator	-	-	-	-
FUN_004768	Protein of unknown function (DUF3112)	-	-	-	-
FUN_004769	WD40 repeats	ATG18	-	ko:K17908	ko04136, ko04138, ko04140, map04136, map04138, map04140
FUN_004770	Leucine carboxyl methyltransferase	PPM2	2.1.1.290, 2.3.1.231	ko:K15451	-
FUN_004771	Scavenger mRNA decapping enzyme (DcpS) N-terminal	-	3.6.1.59	ko:K12584	ko03018, map03018
FUN_004772	DNA polymerase delta, subunit 4	-	-	ko:K03505	ko00230, ko00240, ko01100, ko03030, ko03410, ko03420, ko03430, ko03440, ko05166, map00230, map00240, map01100, map03030, map03410, map03420, map03430, map03440, map05166
FUN_004773	Dual specificity phosphatase, catalytic domain	PPS1	3.1.3.16, 3.1.3.48	ko:K04459	ko04010, map04010
FUN_004774	Protein of unknown function (DUF3533)	-	-	-	-

FUN_004775	ubiquitin carboxyl-terminal hydrolase	YUH1	3.4.19.12	ko:K05609	-
FUN_004776	G protein-coupled glucose receptor regulating Gpa2 C-term	GPR1	-	ko:K12762	ko04113, ko04213, map04113, map04213
FUN_004778	GDP-mannose 4, 6 dehydratase	GAL10	5.1.3.2, 5.1.3.3	ko:K01784, ko:K01785	ko00010, ko00052, ko00520, ko01100, ko01110, ko01120, ko01130, map00010, map00052, map00520, map01100, map01110, map01120, map01130
FUN_004779	Putative tRNA binding domain	ARC1	-	ko:K15437	-
FUN_004781	Catalyzes the hydroxylation of the N(6)-(4-aminobutyl)- L-lysine intermediate to form hypusine, an essential post- translational modification only found in mature eIF-5A	LIA1	1.14.99.29	ko:K06072	-
FUN_004782	DNA-dependent RNA polymerase catalyzes the transcription of DNA into RNA using the four ribonucleoside triphosphates as substrates	RPA12	-	ko:K03000	ko00230, ko00240, ko01100, ko03020, map00230, map00240, map01100, map03020
FUN_004784	SacI homology domain	FIG4	-	-	-
FUN_004785	Major Facilitator Superfamily	-	-	-	-
FUN_004787	Protein of unknown function (DUF1295)	-	-	-	-
FUN_004789	Protein of unknown function (DUF3237)	-	-	ko:K15218	-
FUN_004791	Chromosome segregation protein Spc25	SPC25	-	ko:K11558	-
FUN_004792	Helicase associated domain (HA2) Add an annotation	-	3.6.4.13	ko:K13117	-
FUN_004793	Amino acid permease	-	-	-	-
FUN_004795	Acetyltransferase (GNAT) domain	-	-	-	-
FUN_004797	GAL4-like Zn(II)2Cys6 (or C6 zinc) binuclear cluster DNA-binding domain	-	-	-	-
FUN_004801	K+ potassium transporter	-	-	ko:K03549	-
FUN_004802	Membrane-associating domain	-	-	-	-
FUN_004806	Glycosyltransferase family 31 protein	-	-	-	-
FUN_004809	Hydrolytic ATP binding site of dynein motor region D1	DYN1	-	ko:K10413	ko04145, ko04962, ko05132, map04145, map04962, map05132
FUN_004810	Chloride channel	GEF1	-	ko:K05012	-
FUN_004811	Belongs to the peptidase S10 family	-	3.4.16.5	ko:K13289	ko04142, ko04614, map04142, map04614
FUN_004812	Histone-like transcription factor (CBF/NF-Y) and archaeal histone	-	2.7.7.7	ko:K03004, ko:K03506	ko00230, ko00240, ko01100, ko03020, ko03030, ko03410, ko03420, ko05166, map00230, map00240, map01100, map03020, map03030, map03410, map03420, map05166
FUN_004813	Macro domain	POA1	3.1.3.84	ko:K15529	-
FUN_004814	Component of the ubiquinol-cytochrome c reductase complex (complex III or cytochrome b-c1 complex), which is a respiratory chain that generates an electrochemical potential coupled to ATP synthesis	RIP1	1.10.2.2	ko:K00411	ko00190, ko01100, ko02020, ko04260, ko04714, ko04932, ko05010, ko05012, ko05016, map00190, map01100, map02020, map04260, map04714, map04932, map05010, map05012, map05016
FUN_004815	Involved in nuclear export, actin cytoskeleton organization and vesicular transport	BCP1	-	ko:K15262	-
FUN_004816	GMP synthase	GUA1	6.3.5.2	ko:K01951	ko00230, ko00983, ko01100, map00230, map00983, map01100
FUN_004818	Peptidase family S41	-	-	-	-
FUN_004819	Belongs to the short-chain dehydrogenases reductases (SDR) family	-	1.1.1.381	ko:K16066	ko00240, ko00260, ko01100, map00240, map00260, map01100
FUN_004821	Epoxide hydrolase N terminus	-	-	-	-
FUN_004824	Histidine kinase group protein	-	-	-	-
FUN_004825	emp24/gp25L/p24 family/GOLD	EMP24	-	ko:K20347	-
FUN_004827	Glycoside hydrolase family 13 protein	-	3.2.1.10	ko:K01182	ko00052, ko00500, ko01100, map00052, map00500, map01100
FUN_004832	L-alpha-amino acid transmembrane transport	MUP3	-	-	-

FUN_004833	Alpha/beta hydrolase family	-	-	-	-
FUN_004835	Dipeptidyl peptidase IV (DPP IV) N-terminal region	dpp4	3.4.14.5	ko:K01278, ko:K01282	ko04974, map04974
FUN_004837	Ferric reductase like transmembrane component	-	-	-	-
FUN_004838	Ctr copper transporter family	-	-	-	-
FUN_004839	Catalyzes the methyl transfer from S-adenosyl-methionine to the C-24 of zymosterol to form fecosterol	ERG6	2.1.1.41	ko:K00559	ko00100, ko01100, ko01110, ko01130, map00100, map01100, map01110, map01130
FUN_004842	Belongs to the OSBP family	-	-	ko:K20465	-
FUN_004845	Belongs to the aldehyde dehydrogenase family	-	-	-	-
FUN_004846	Ecdysteroid kinase	-	-	-	-
FUN_004849	Belongs to the peptidase M28 family	LAP2	3.4.11.15	ko:K01264, ko:K01302	-
FUN_004851	HAD-hyrolase-like	-	-	-	-
FUN_004852	Lytic polysaccharide mono-oxygenase, cellulose-degrading	-	-	-	-
FUN_004856	Belongs to the cytochrome b5 family	-	1.1.2.3	ko:K00101	ko00620, ko01100, map00620, map01100
FUN_004857	This magnesium-dependent enzyme catalyzes the hydrolysis of ATP coupled with the transport of calcium	PMC1	3.6.3.8	ko:K01537	-
FUN_004860	Thioesterase-like superfamily	-	-	-	-
FUN_004861	Amino acid permease	-	-	-	-
FUN_004864	Phytanoyl-CoA dioxygenase (PhyH)	-	-	-	-
FUN_004865	Belongs to the GMC oxidoreductase family	-	1.1.99.1	ko:K00108	ko00260, ko01100, map00260, map01100
FUN_004867	Belongs to the ubiquitin-activating E1 family	UBA1	6.2.1.45	ko:K03178	ko04120, ko05012, map04120, map05012
FUN_004868	Component of the cytosolic iron-sulfur (Fe S) protein assembly (CIA) machinery. Required for maturation of extramitochondrial Fe-S proteins. The NBP35-CFD1 heterotetramer forms a Fe-S scaffold complex, mediating the de novo assembly of an Fe-S cluster and its transfer to target apoproteins	NBP35	-	ko:K03593	-
FUN_004872	Uncharacterised protein family (UPF0261)	-	-	-	-
FUN_004873	Phosphoenolpyruvate hydrolase-like	-	-	-	-
FUN_004874	Glutaredoxin	-	-	-	-
FUN_004875	Methyltransferase domain	-	-	-	-
FUN_004876	Dehydrogenase	-	1.1.1.170	ko:K07748	ko00100, ko01100, ko01130, map00100, map01100, map01130
FUN_004878	Spherulation-specific family 4	-	-	-	-
FUN_004879	FAD dependent oxidoreductase	-	-	-	-
FUN_004880	Belongs to the cytochrome P450 family	-	-	-	-
FUN_004881	Major Facilitator Superfamily	-	-	-	-
FUN_004882	C2H2 type zinc finger domain protein	-	-	-	-
FUN_004883	Belongs to the D-isomer specific 2-hydroxyacid dehydrogenase family	-	1.1.1.79, 1.1.1.81	ko:K00049	ko00260, ko00620, ko00630, ko01100, ko01110, ko01120, map00260, map00620, map00630, map01100, map01110, map01120
FUN_004887	Fatty-acid amide hydrolase	AMD2	3.5.1.4	ko:K01426	ko00330, ko00360, ko00380, ko00627, ko00643, ko01120, map00330, map00360, map00380, map00627, map00643, map01120
FUN_004888	Major Facilitator Superfamily	-	-	-	-
FUN_004890	Fungal trichothecene efflux pump (TRI12)	-	-	-	-

FUN_004891	Extracellular dioxygenase	-	-	-	-
FUN_004894	3-demethylubiquinone-9 3-methyltransferase	-	-	-	-
FUN_004895	Zinc-finger of C2H2 type	-	-	-	-
FUN_004897	Domain of unknown function (DUF4202)	-	-	-	-
FUN_004900	Domain present in VPS9	vps901	-	ko:K20131	-
FUN_004901	GAL4-like Zn(II)2Cys6 (or C6 zinc) binuclear cluster DNA-binding domain	-	-	-	-
FUN_004902	Fungal specific transcription factor domain	-	-	-	-
FUN_004906	Sulfatase	-	-	-	-
FUN_004907	Cytochrome P450	-	-	ko:K21293	-
FUN_004909	Alpha-glucuronidase involved in the hydrolysis of xylan, a major structural heterogeneous polysaccharide found in plant biomass representing the second most abundant polysaccharide in the biosphere, after cellulose. Releases 4-O-methylglucuronic acid from xylan	aguA	3.2.1.139	ko:K01235	-
FUN_004913	Prolyl oligopeptidase family	dpp5	-	-	-
FUN_004914	POT family	-	-	ko:K03305	-
FUN_004916	GMC oxidoreductase	-	1.1.99.1	ko:K00108	ko00260, ko01100, map00260, map01100
FUN_004919	Pantoate-beta-alanine ligase	pan6	6.3.2.1	ko:K01918	ko00410, ko00770, ko01100, ko01110, map00410, map00770, map01100, map01110
FUN_004920	Belongs to the DEAD box helicase family	DBP7	3.6.4.13	ko:K14806	-
FUN_004922	Belongs to the cytochrome P450 family	-	1.14.13.177	ko:K17826	ko00404, ko01130, map00404, map01130
FUN_004923	Belongs to the cytochrome b5 family	-	-	-	-
FUN_004924	RNA recognition motif	NOP4	-	ko:K14573	ko03008, map03008
FUN_004926	Heterokaryon incompatibility protein (HET)	-	-	-	-
FUN_004927	Sugar (and other) transporter	-	-	-	-
FUN_004928	Ribosomal protein L6	RPL9B	-	ko:K02940	ko03010, map03010
FUN_004929	Aldo/keto reductase family	-	-	-	-
FUN_004933	Esterase PHB depolymerase	-	3.1.1.72	ko:K05972	-
FUN_004934	Alpha beta hydrolase fold family	-	-	-	-
FUN_004935	Cytochrome P450	-	-	-	-
FUN_004936	Domain that may be involved in binding ubiquitin-conjugating enzymes (UBCs)	CUE5	-	ko:K06653	ko04111, map04111
FUN_004938	Alcohol dehydrogenase GroES-like domain	-	-	-	-
FUN_004939	Serine aminopeptidase, S33	-	3.4.11.5	ko:K01259	ko00330, map00330
FUN_004940	Aminotransferase class-V	hxB	2.8.1.9	ko:K15631	ko00790, map00790
FUN_004943	O-methyltransferase	-	-	-	-
FUN_004945	metallo-protease	-	-	-	-
FUN_004948	Domain of unknown function (DUF1929)	-	1.1.3.9	ko:K04618	ko00052, map00052
FUN_004949	snRNP Sm proteins	-	-	ko:K12627	ko03018, ko03040, map03018, map03040
FUN_004950	PITH domain	-	-	-	-
FUN_004951	Belongs to the SNF7 family	VPS20	-	ko:K12195	ko04144, ko04217, map04144, map04217
FUN_004952	Holliday junction DNA helicase ruvB N-terminus	RIX7	-	ko:K14571	ko03008, map03008
FUN_004959	L-lysine 6-monooxygenase (NADPH-requiring)	-	-	-	-
FUN_004960	Enoyl-(Acyl carrier protein) reductase	-	-	-	-

FUN_004962	Major royal jelly protein	-	-	-	-
FUN_004963	Major Facilitator Superfamily	-	-	-	-
FUN_004965	Enoyl-(Acyl carrier protein) reductase	-	-	-	-
FUN_004966	Uncharacterized protein conserved in bacteria (DUF2334)	-	-	-	-
FUN_004969	Major Facilitator Superfamily	-	-	ko:K03448	-
FUN_004970	Major Facilitator Superfamily	-	-	ko:K03448	-
FUN_004971	Heterokaryon incompatibility protein (HET)	-	-	-	-
FUN_004972	HET domain-containing protein	-	-	-	-
FUN_004973	Leucine Rich repeat	-	-	-	-
FUN_004975	ATPase family associated with various cellular activities (AAA)	-	-	-	-
FUN_004977	Belongs to the short-chain dehydrogenases reductases (SDR) family	-	-	-	-
FUN_004978	GAL4-like Zn(II)2Cys6 (or C6 zinc) binuclear cluster DNA-binding domain	-	-	-	-
FUN_004979	Belongs to the DapA family	-	4.1.3.16	ko:K18123	ko00330, ko00630, ko01100, map00330, map00630, map01100
FUN_004980	Acyclic terpene utilisation family protein AtuA	-	-	-	-
FUN_004981	Secreted protein	-	-	-	-
FUN_004983	Belongs to the major facilitator superfamily. Sugar transporter (TC 2.A.1.1) family	-	-	ko:K08141	-
FUN_004984	Major Facilitator Superfamily	-	-	-	-
FUN_004985	Catalyzes the reversible hydration of cis-homoaconitate to (2R, 3S)-homoisocitrate, a step in the alpha-aminoadipate pathway for lysine biosynthesis	LYS4	4.2.1.36	ko:K01705	ko00300, ko01100, ko01120, ko01130, ko01210, ko01230, map00300, map01100, map01120, map01130, map01210, map01230
FUN_004986	Protein of unknown function (DUF1777)	-	-	ko:K12846	ko03040, map03040
FUN_004987	Glycoside hydrolase family 63 protein	CWH41	3.2.1.106	ko:K01228	ko00510, ko01100, ko04141, map00510, map01100, map04141
FUN_004988	Elongator subunit Iki1	-	-	ko:K11376	-
FUN_004990	Enoyl-(Acyl carrier protein) reductase	-	-	-	-
FUN_004993	RWD	-	2.3.2.31	ko:K11971	-
FUN_004994	Acid phosphatase homologues	-	3.6.1.43	ko:K07252	ko00510, map00510
FUN_004995	Bacterial low temperature requirement A protein (LtrA)	-	-	-	-
FUN_004998	Podospora anserina S mat genomic DNA chromosome	-	-	-	-
FUN_004999	Hus1-like protein	hus1	-	ko:K10903	ko04218, map04218
FUN_005000	MnmE helical domain	MSS1	-	ko:K03650	-
FUN_005001	Belongs to the MAK16 family	mak16	-	ko:K14831	-
FUN_005004	FAD binding domain	-	1.14.13.7	ko:K03380	ko00623, ko00627, ko01120, map00623, map00627, map01120
FUN_005007	peroxidase	-	1.11.1.13	ko:K20205	-
FUN_005009	Luciferase-like monooxygenase	-	-	-	-
FUN_005010	Alpha/beta hydrolase family	-	-	-	-
FUN_005011	Permease for cytosine/purines, uracil, thiamine, allantoin	-	-	ko:K03457	-
FUN_005012	Flavin reductase like domain	-	-	-	-
FUN_005013	Zinc-binding dehydrogenase	-	-	-	-
FUN_005014	DUF1446 domain-containing protein	-	-	-	-
FUN_005015	to Saccharomyces cerevisiae YLR278C	-	-	-	-
FUN_005016	to Saccharomyces cerevisiae YLR278C	-	-	-	-
FUN_005019	KR domain	-	1.1.1.289	ko:K17742	ko00051, map00051

FUN_005020	Hemerythrin HHE cation binding domain	-	-	-	-
FUN_005021	Cutinase	-	3.1.1.72	ko:K05972	-
FUN_005023	Sugar (and other) transporter	-	-	-	-
FUN_005024	Zinc-binding dehydrogenase	-	-	-	-
FUN_005027	Belongs to the type-B carboxylesterase lipase family	-	-	-	-
FUN_005028	Peptidase dimerisation domain	-	-	-	-
FUN_005029	Amidohydrolase family	-	3.5.2.2	ko:K01464	ko00240, ko00410, ko00770, ko00983, ko01100, map00240, map00410, map00770, map00983, map01100
FUN_005031	Aspartokinase	HOM3	2.7.2.4	ko:K00928	ko00260, ko00261, ko00270, ko00300, ko01100, ko01110, ko01120, ko01130, ko01210, ko01230, map00260, map00261, map00270, map00300, map01100, map01110, map01120, map01130, map01210, map01230
FUN_005032	homoserine O-acetyltransferase	MET2	2.3.1.31	ko:K00641	ko00270, ko01100, ko01130, map00270, map01100, map01130
FUN_005033	Carbon-nitrogen hydrolase	NIT1	3.5.5.1, 3.5.5.4, 4.2.1.65	ko:K01501, ko:K13035	ko00380, ko00460, ko00627, ko00643, ko00910, ko01100, ko01110, ko01120, map00380, map00460, map00627, map00643, map00910, map01100, map01110, map01120
FUN_005034	endocytosis	-	2.4.1.25, 3.2.1.33	ko:K01196, ko:K12483	ko00500, ko01100, ko01110, ko04144, map00500, map01100, map01110, map04144
FUN_005035	DegT/DnrJ/EryC1/StrS aminotransferase family	MET15	2.5.1.47, 2.5.1.49	ko:K17069	ko00270, ko00920, ko01100, ko01110, ko01120, ko01130, ko01200, ko01230, map00270, map00920, map01100, map01110, map01120, map01130, map01200, map01230
FUN_005037	Peptidase family S41	-	-	-	-
FUN_005038	Glycoside hydrolase family 85 protein	-	-	-	-
FUN_005039	Nitroreductase family	-	-	-	-
FUN_005040	Plasma membrane ATPase	-	3.6.3.6	ko:K01535	ko00190, map00190
FUN_005045	Catalyzes the NAD( )-dependent oxidation of formate to carbon dioxide. Formate oxidation is the final step in the methanol oxidation pathway in methylotrophic microorganisms. Has a role in the detoxification of exogenous formate in non-	FDH1	1.17.1.9	ko:K00122	ko00630, ko00680, ko01100, ko01120, ko01200, map00630, map00680, map01100, map01120, map01200
FUN_005047	Diaphanous FH3 Domain	-	-	ko:K05740, ko:K11238, ko:K17971	ko04011, ko04510, ko04810, ko04933, ko05131, map04011, map04510, map04810, map04933, map05131
FUN_005049	zinc finger	-	-	-	-
FUN_005050	Belongs to the major facilitator superfamily. Sugar transporter (TC 2.A.1.1) family	-	-	-	-
FUN_005051	Belongs to the aldehyde dehydrogenase family	-	1.2.1.3	ko:K00128	ko00010, ko00053, ko00071, ko00280, ko00310, ko00330, ko00340, ko00380, ko00410, ko00561, ko00620, ko00625, ko00903, ko00981, ko01100, ko01110, ko01120, ko01130, map00010, map00053, map00071, map00280, map00310, map00330, map00340, map00380, map00410, map00561, map00620, map00625, map00903, map00981, map01100, map01110, map01120, map01130
FUN_005054	Cupin	-	-	-	-
FUN_005055	Protein of unknown function (DUF1275)	-	-	-	-
FUN_005057	High-affinity nicotinic acid transporter	-	-	-	-

FUN_005058	Belongs to the type-B carboxylesterase lipase family	-	-	-	-
FUN_005059	integral membrane protein	-	-	-	-
FUN_005060	Transporter	-	-	-	-
FUN_005061	MmgE/PrpD family	-	-	-	-
FUN_005064	Epoxide hydrolase N terminus	-	-	-	-
FUN_005066	resistance protein	-	-	ko:K08158	-
FUN_005067	Amino acid transporter	-	1.14.18.1	ko:K00505	ko00350, ko00950, ko00965, ko01100, ko01110, ko04916, map00350, map00950, map00965, map01100, map01110, map04916
FUN_005068	Extracellular dioxygenase	-	-	-	-
FUN_005069	Alcohol dehydrogenase GroES-like domain	-	-	-	-
FUN_005071	Nitronate monooxygenase	-	1.13.12.16	ko:K00459	ko00910, map00910
FUN_005072	Belongs to the major facilitator superfamily. Sugar transporter (TC 2.A.1.1) family	-	-	-	-
FUN_005073	RNA recognition motif	cwf29	-	ko:K13107	-
FUN_005074	Dynein light intermediate chain (DLIC)	-	-	ko:K10416	ko04145, ko04962, ko05132, map04145, map04962, map05132
FUN_005075	Tyrosine phosphatase family	-	-	-	-
FUN_005076	Belongs to the TRAFAC class TrmE-Era-EngA-EngB-Septin- like GTPase superfamily. Septin GTPase family	CDC11	-	ko:K16945, ko:K16946	-
FUN_005077	WD40 repeats	HSV2	-	-	-
FUN_005078	Catalyzes the epimerization of the S- and R-forms of NAD(P)HX, a damaged form of NAD(P)H that is a result of enzymatic or heat-dependent hydration. This is a prerequisite for the S- specific NAD(P)H-hydrate dehydratase to allow the repair of both epimers of NAD(P)HX	-	5.1.99.6	ko:K17759	-
FUN_005079	Ribosomal protein S16	MRPS16	-	ko:K02959	ko03010, map03010
FUN_005080	Transmembrane amino acid transporter protein	-	-	-	-
FUN_005081	Dynactin subunit p22	-	-	-	-
FUN_005082	Plasma membrane osmosensor that activates the high osmolarity glycerol (HOG) MAPK signaling pathway in response to high osmolarity	SHO1	-	ko:K11246	ko04011, map04011
FUN_005083	Belongs to the BI1 family	-	-	ko:K06890	-
FUN_005084	TRAM1-like protein	lag1	2.3.1.24	ko:K04709	ko00600, ko01100, map00600, map01100
FUN_005085	Glycoside hydrolase family 76 protein	-	6.4.1.4	ko:K01969	ko00280, ko01100, map00280, map01100
FUN_005086	Glycosyl hydrolase family 76	-	3.2.1.101	ko:K08257	-
FUN_005088	Acyl-CoA dehydrogenase, C-terminal domain	-	1.3.8.4	ko:K00253	ko00280, ko01100, map00280, map01100
FUN_005089	Carboxymuconolactone decarboxylase family	-	-	-	-
FUN_005090	Catechol dioxygenase N terminus	-	1.13.11.1	ko:K03381	ko00361, ko00362, ko00364, ko00623, ko01100, ko01120, ko01220, map00361, map00362, map00364, map00623, map01100, map01120, map01220
FUN_005091	Lactonase, 7-bladed beta-propeller	-	5.5.1.5	ko:K14334	ko00362, map00362
FUN_005092	Ndr family	-	-	-	-
FUN_005093	2OG-Fe(II) oxygenase superfamily	-	-	-	-
FUN_005094	AMP-binding enzyme C-terminal domain	-	-	ko:K14818	-
FUN_005095	WD domain, G-beta repeat	SQT1	-	ko:K14818	-
FUN_005101	structural maintenance of chromosomes protein	SMC3	-	ko:K06669	ko04110, ko04111, ko04113, ko04114, map04110, map04111, map04113, map04114

FUN_005102	Glycoside hydrolase family 16 protein	-	-	-	-
FUN_005106	Oxidoreductase family, NAD-binding Rossmann fold	GAL80	-	-	-
FUN_005108	DJ-1/PfpI family	-	-	-	-
FUN_005109	Beta-lactamase	-	-	-	-
FUN_005111	Belongs to the glycosyl hydrolase 43 family	-	-	-	-
FUN_005112	Aminotransferase class-V	-	-	-	-
FUN_005123	Belongs to the major facilitator superfamily. Sugar transporter (TC 2.A.1.1) family	-	-	-	-
FUN_005125	Belongs to the major facilitator superfamily. Sugar transporter (TC 2.A.1.1) family	-	-	-	-
FUN_005128	Taurine catabolism dioxygenase TauD, TfdA family	-	-	-	-
FUN_005135	Beige/BEACH domain	BPH1	-	ko:K22262	-
FUN_005136	Belongs to the peptidase S8 family	PRB1	3.4.21.48	ko:K01336	ko04138, map04138
FUN_005137	GAL4-like Zn(II)2Cys6 (or C6 zinc) binuclear cluster DNA-binding domain	-	-	-	-
FUN_005138	Flavin-containing monooxygenase	-	-	-	-
FUN_005141	Uncharacterized protein conserved in bacteria (DUF2237)	-	-	ko:K09966	-
FUN_005142	PFU (PLAA family ubiquitin binding)	lub1	-	ko:K14018	ko04141, map04141
FUN_005143	Broad-Complex, Tramtrack and Bric a brac	-	-	-	-
FUN_005144	Major Facilitator Superfamily	-	-	-	-
FUN_005145	KR domain	-	-	-	-
FUN_005147	Belongs to the major facilitator superfamily. Sugar transporter (TC 2.A.1.1) family	-	-	-	-
FUN_005148	c6 transcription factor	-	-	-	-
FUN_005150	Belongs to the protein kinase superfamily. RIO-type Ser Thr kinase family	rio1	2.7.11.1	ko:K07178	ko03008, map03008
FUN_005151	Autophagy protein Apg17	atg17	-	ko:K08329	ko04138, map04138
FUN_005152	A Receptor for Ubiquitination Targets	sconB	-	ko:K10259	ko04111, ko04120, map04111, map04120
FUN_005153	Methyltransferase domain	-	-	-	-
FUN_005154	Belongs to the cytochrome P450 family	-	-	-	-
FUN_005155	Belongs to the DHHC palmitoyltransferase family	PFA5	2.3.1.225	ko:K18932	-
FUN_005156	YEATS family	YAF9	-	ko:K11341	-
FUN_005157	NAD(P)H-binding	-	-	-	-
FUN_005158	Partial alpha/beta-hydrolase lipase region	TGL1	3.1.1.13	ko:K01052	ko00100, ko04142, ko04979, map00100, map04142, map04979
FUN_005159	Cytochrome c oxidase assembly protein PET191	-	-	ko:K18178	ko04714, map04714
FUN_005161	AMP-binding enzyme C-terminal domain	-	-	-	-
FUN_005168	PLD-like domain	-	-	-	-
FUN_005171	LamB/YcsF family	-	-	ko:K07160	-
FUN_005172	Allophanate hydrolase subunit 2	-	6.3.4.6	ko:K01941	ko00220, ko00791, ko01100, map00220, map00791, map01100
FUN_005173	Duf1446 domain protein	-	-	-	-
FUN_005174	KR domain	-	-	-	-
FUN_005175	Enoyl-(Acyl carrier protein) reductase	-	-	-	-
FUN_005177	Nitroreductase family	HBN1	-	ko:K07078	-
FUN_005178	Belongs to the enoyl-CoA hydratase isomerase family	-	4.2.1.17	ko:K01715	ko00650, ko01200, map00650, map01200
FUN_005179	Heterokaryon incompatibility protein (HET)	-	-	-	-
FUN_005180	FMN-dependent dehydrogenase	-	1.13.12.4	ko:K00467	ko00620, map00620

FUN_005181	Belongs to the glycosyl hydrolase 47 family	mns1B	3.2.1.113	ko:K01230	ko00510, ko00513, ko01100, ko04141, map00510, map00513, map01100, map04141
FUN_005183	gpi anchored	-	-	-	-
FUN_005184	Repeated motif present between transmembrane helices in cystinosin, yeast ERS1p, mannose-P-dolichol utilization defect 1, and other hypothetical proteins.	-	-	-	-
FUN_005186	3-hydroxyacyl-[acyl-carrier-protein] dehydratase activity	-	-	-	-
FUN_005187	Putative peptidase family	PRA1	-	-	-
FUN_005188	ZIP Zinc transporter	ZRT1	-	ko:K14709	-
FUN_005191	Transcription initiation factor TFIID subunit	-	-	ko:K03135	ko03022, map03022
FUN_005192	cytochrome P450	-	1.14.13.78	ko:K21292	ko00904, ko01100, map00904, map01100
FUN_005193	Belongs to the glycosyl hydrolase 1 family	-	-	-	-
FUN_005195	Domain of unknown function (DUF3328)	-	-	-	-
FUN_005197	Major Facilitator Superfamily	-	-	-	-
FUN_005199	Animal haem peroxidase	-	-	-	-
FUN_005200	Major Facilitator Superfamily	-	-	-	-
FUN_005201	Alpha/beta hydrolase family	-	-	-	-
FUN_005204	NAD(P)H-binding	-	-	-	-
FUN_005205	Belongs to the iron ascorbate-dependent oxidoreductase family	-	-	-	-
FUN_005207	Peptidase family M20/M25/M40	-	-	-	-
FUN_005209	GMC oxidoreductase	-	1.1.3.13	ko:K17066	ko00680, ko01100, ko01120, ko01200, map00680, map01100, map01120, map01200
FUN_005211	non-haem dioxygenase in morphine synthesis N-terminal	-	-	-	-
FUN_005212	Major Facilitator Superfamily	-	-	-	-
FUN_005213	Pleckstrin homology domain	-	-	-	-
FUN_005214	Acetyltransferase (GNAT) domain	ats1	-	-	-
FUN_005215	Belongs to the complex I LYR family	-	-	-	-
FUN_005216	KilA-N	SWI6	-	ko:K06648	ko04011, ko04111, ko04113, map04011, map04111, map04113
FUN_005217	Glycoside hydrolase family 51 protein	-	3.2.1.55	ko:K01209	ko00520, map00520
FUN_005218	Belongs to the major facilitator superfamily. Sugar transporter (TC 2.A.1.1) family	-	-	-	-
FUN_005220	M6 family metalloprotease domain-containing protein	-	-	-	-
FUN_005221	Serine aminopeptidase, S33	-	-	ko:K13535	ko00564, map00564
FUN_005222	Spo7-like protein	SPO7	-	-	-
FUN_005223	Serine aminopeptidase, S33	-	3.1.1.23	ko:K13704	-
FUN_005224	DUF636 domain-containing protein	-	-	-	-
FUN_005227	metallo-beta-lactamase superfamily protein	-	2.5.1.105	ko:K06897	ko00790, map00790
FUN_005229	Cytochrome P450	-	-	-	-
FUN_005230	Belongs to the cytochrome P450 family	-	1.14.14.23	ko:K00489	ko00120, ko00140, ko01100, ko03320, ko04976, ko04979, map00120, map00140, map01100, map03320, map04976, map04979
FUN_005232	Belongs to the thiolase family	-	2.3.1.16	ko:K07513	ko00071, ko00280, ko00592, ko01040, ko01100, ko01110, ko01130, ko01212, ko03320, ko04146, map00071, map00280, map00592, map01040, map01100, map01110, map01130, map01212, map03320, map04146
FUN_005233	Cytochrome P450	-	-	-	-

FUN_005234	Heterokaryon incompatibility protein (HET)	-	-	-	-
FUN_005237	Belongs to the cytochrome P450 family	-	-	-	-
FUN_005238	Glutathione S-transferase, C-terminal domain	-	-	-	-
FUN_005239	phospholipid catabolic process	PLA2G4F	3.1.1.4	ko:K04257, ko:K16342, ko:K19219	ko00564, ko00565, ko00590, ko00591, ko00592, ko01100, ko01110, ko04010, ko04014, ko04072, ko04217, ko04270, ko04370, ko04611, ko04664, ko04724, ko04726, ko04730, ko04740, ko04750, ko04912, ko04913, ko04921, ko05231, map00564, map00565, map00590, map00591, map00592, map01100, map01110, map04010, map04014, map04072, map04217, map04270, map04370, map04611, map04664, map04724, map04726, map04730, map04740, map04750, map04912, map04913, map04921, map05231
FUN_005241	Exo70 exocyst complex subunit	EXO70	-	ko:K07195	ko04910, map04910
FUN_005242	Belongs to the TRAFAC class TrmE-Era-EngA-EngB-Septin- like GTPase superfamily. Septin GTPase family	-	-	ko:K16944	-
FUN_005243	Uncharacterised protein family (UPF0121)	POM33	-	ko:K20724	-
FUN_005245	Phosphorylase is an important allosteric enzyme in carbohydrate metabolism. Enzymes from different sources differ in their regulatory mechanisms and in their natural substrates. However, all known phosphorylases share catalytic and structural properties	GPH1	2.4.1.1	ko:K00688	ko00500, ko01100, ko01110, ko02026, ko04217, ko04910, ko04922, ko04931, map00500, map01100, map01110, map02026, map04217, map04910, map04922, map04931
FUN_005246	Yip1 domain	-	-	-	-
FUN_005247	Heterokaryon incompatibility protein (HET)	-	-	-	-
FUN_005248	Fungal N-terminal domain of STAND proteins	-	-	-	-
FUN_005249	Belongs to the cytochrome P450 family	-	-	-	-
FUN_005251	Holliday junction DNA helicase ruvB N-terminus	ctf18	-	ko:K11269	-
FUN_005252	Belongs to the iron ascorbate-dependent oxidoreductase family	-	-	-	-
FUN_005253	Catalyzes the NADPH-dependent reduction of ketopantoate into pantoic acid	-	1.1.1.169	ko:K00077	ko00770, ko01100, ko01110, map00770, map01100, map01110
FUN_005254	Belongs to the ABC transporter superfamily. ABCG family. PDR (TC 3.A.1.205)	-	-	ko:K08712	ko02010, map02010
FUN_005258	Glutathione S-transferase, C-terminal domain	-	1.8.5.7	ko:K07393	-
FUN_005265	Belongs to the GMC oxidoreductase family	-	1.1.99.1	ko:K00108	ko00260, ko01100, map00260, map01100
FUN_005267	Chitin binding domain	-	-	-	-
FUN_005268	Class II histone deacetylase complex subunits 2 and 3	IRC5	-	ko:K19001	-
FUN_005269	Acetyltransferase (GNAT) domain	-	-	-	-
FUN_005270	Heterokaryon incompatibility protein (HET)	-	-	-	-
FUN_005271	Belongs to the glycosyl hydrolase 5 (cellulase A) family	-	3.2.1.78	ko:K19355	ko00051, map00051
FUN_005276	FAD dependent oxidoreductase	-	1.5.3.1, 1.5.3.7	ko:K00306	ko00260, ko00310, ko01100, ko04146, map00260, map00310, map01100, map04146
FUN_005280	Protein of unknown function (DUF890)	-	2.1.1.181	ko:K06970, ko:K11393	-
FUN_005281	Sirohaem biosynthesis protein C-terminal	MET8	1.3.1.76, 4.99.1.4	ko:K02304	ko00860, ko01100, ko01110, ko01120, map00860, map01100, map01110, map01120
FUN_005282	Rabenosyn Rab binding domain	PEP7	-	ko:K12481	ko04144, map04144
FUN_005284	Exocyst complex component Sec5	SEC5	-	ko:K17637	ko04014, map04014
FUN_005286	Dual specificity phosphatase, catalytic domain	-	-	-	-

FUN_005287	Acetyltransferase (GNAT) domain	-	-	-	-
FUN_005289	Amino acid permease	-	-	-	-
FUN_005290	trichothecene efflux pump	-	-	-	-
FUN_005291	RmlD substrate binding domain	-	-	-	-
FUN_005292	Belongs to the type-B carboxylesterase lipase family	-	-	-	-
FUN_005293	BTB/POZ domain	-	-	-	-
FUN_005296	Belongs to the class-I aminoacyl-tRNA synthetase family	GLN4	6.1.1.18	ko:K01886	ko00970, ko01100, map00970, map01100
FUN_005297	Fungal specific transcription factor domain	-	-	-	-
FUN_005298	Cation-transporting ATPase	SPF1	3.6.3.8	ko:K01537, ko:K14950	-
FUN_005300	Belongs to the mitochondrial carrier (TC 2.A.29) family	-	-	-	-
FUN_005301	Heterokaryon incompatibility protein (HET)	-	-	-	-
FUN_005302	Polycystin cation channel	YVC1	-	-	-
FUN_005303	Eukaryotic translation initiation factor 2 alpha subunit	tif211	-	ko:K03237	ko03013, ko04138, ko04140, ko04141, ko04210, ko04932, ko05160, ko05162, ko05164, ko05168, map03013, map04138, map04140, map04141, map04210, map04932, map05160, map05162, map05164, map05168
FUN_005317	Belongs to the major facilitator superfamily. Sugar transporter (TC 2.A.1.1) family	-	-	-	-
FUN_005318	Alpha-ketoglutarate-dependent 2, 4-dichlorophenoxyacetate dioxygenase	-	-	ko:K06912	ko00361, ko01120, ko01220, map00361, map01120, map01220
FUN_005320	)transporter	-	-	-	-
FUN_005321	Amino acid permease	-	-	ko:K16261	-
FUN_005322	Glutamine amidotransferases class-II	-	-	ko:K18802	-
FUN_005323	Belongs to the two pore domain potassium channel (TC 1.A.1.8) family	TOK1	-	ko:K05389	-
FUN_005327	Amino acid permease	-	-	-	-
FUN_005329	Fungal trichothecene efflux pump (TRI12)	-	-	-	-
FUN_005330	Alpha/beta hydrolase family	-	-	-	-
FUN_005335	Endonuclease that specifically degrades the RNA of RNA- DNA hybrids	-	3.1.26.4	ko:K03469	ko03030, map03030
FUN_005337	Belongs to the TPP enzyme family	-	-	-	-
FUN_005338	SET (Su(var)3-9, Enhancer-of-zeste, Trithorax) domain	SET5	-	ko:K07117, ko:K11426	-
FUN_005341	TAP-like protein	-	-	-	-
FUN_005342	Belongs to the AlaDH PNT family	LYS1	1.5.1.7, 3.4.19.12	ko:K00290, ko:K11874	ko00300, ko00310, ko01100, ko01110, ko01130, ko01230, map00300, map00310, map01100, map01110, map01130, map01230
FUN_005343	STAS domain	-	-	-	-
FUN_005344	Sugar (and other) transporter	JEN1	-	ko:K08178	-
FUN_005345	NAD(P)H-binding	-	-	-	-
FUN_005347	Belongs to the glycosyl hydrolase 5 (cellulase A) family	-	3.2.1.4	ko:K01179	ko00500, ko01100, map00500, map01100
FUN_005349	ADP-ribosylglycohydrolase	-	-	-	-
FUN_005350	Domain of unknown function (DUF3328)	-	-	-	-
FUN_005355	Double-stranded RNA binding motif	mrpl3	-	ko:K17425	-
FUN_005356	pfkB family carbohydrate kinase	-	-	-	-
FUN_005357	Belongs to the CAP family	SRV2	-	ko:K17261	-

FUN_005358	Protein tyrosine kinase	-	-	-	-
FUN_005361	Aminotransferase class-V	-	4.4.1.16, 4.4.1.36	ko:K01763, ko:K20247	ko00340, ko00450, ko01100, map00340, map00450, map01100
FUN_005362	Chromatin modification-related protein EAF7	-	-	ko:K11343, ko:K11399	-
FUN_005363	spindle pole body component	-	-	-	-
FUN_005364	Acyltransferase family	-	-	-	-
FUN_005365	PGAP1-like protein	BST1	-	ko:K05294	ko00563, ko01100, map00563, map01100
FUN_005366	SNARE required for protein transport between the ER and the Golgi complex	BOS1	-	ko:K08496	ko04130, map04130
FUN_005369	Nitrate reductase is a key enzyme involved in the first step of nitrate assimilation in plants, fungi and bacteria	-	1.7.1.1, 1.7.1.2, 1.7.1.3	ko:K10534	ko00910, ko01120, map00910, map01120
FUN_005370	BFD-like [2Fe-2S] binding domain	-	1.7.1.4	ko:K17877	ko00910, ko01120, map00910, map01120
FUN_005371	helicase superfamily c-terminal domain	irc3	-	ko:K17677	-
FUN_005373	Belongs to the peptidase M28 family	-	-	-	-
FUN_005375	Major royal jelly protein	-	-	-	-
FUN_005376	Domain of unknown function (DUF1981)	SEC7	-	ko:K18442	ko04144, map04144
FUN_005377	The pyruvate dehydrogenase complex catalyzes the overall conversion of pyruvate to acetyl-CoA and CO(2)	LAT1	2.3.1.12	ko:K00627	ko00010, ko00020, ko00620, ko01100, ko01110, ko01120, ko01130, ko01200, map00010, map00020, map00620, map01100, map01110, map01120, map01130, map01200
FUN_005378	Belongs to the oxygen-dependent FAD-linked oxidoreductase family	-	-	-	-
FUN_005379	Haem-degrading	-	-	-	-
FUN_005380	intermembrane space import and assembly protein 40	MIA40	-	ko:K17782	-
FUN_005381	Belongs to the two pore domain potassium channel (TC 1.A.1.8) family	TOK1	-	ko:K05389	-
FUN_005382	Electron transfer flavoprotein FAD-binding domain	ETF1	-	ko:K03522	-
FUN_005383	SWIRM-associated region 1	ssr2	-	ko:K11649, ko:K11772	ko04714, ko05225, map04714, map05225
FUN_005384	GYF domain	SMY2	-	ko:K18730	-
FUN_005385	Spc24 subunit of Ndc80	spc24	-	ko:K11557	-
FUN_005386	Has a role in mitochondrial fission	FIS1	-	ko:K17969	ko04137, ko04139, map04137, map04139
FUN_005387	RING-like zinc finger	-	2.3.2.27	ko:K11982	-
FUN_005390	Translation-initiation factor 2	FUN12	-	ko:K03243	ko03013, map03013
FUN_005391	Belongs to the TRAFAC class myosin-kinesin ATPase superfamily. Myosin family	-	2.4.1.16	ko:K00698	ko00520, map00520
FUN_005392	Glycosyltransferase family 2 protein	-	2.4.1.16	ko:K00698	ko00520, map00520
FUN_005393	Actin cytoskeleton-regulatory complex protein END3	END3	-	ko:K20048	-
FUN_005394	Glycosyltransferase family 2 protein	chsD	2.4.1.16	ko:K00698	ko00520, map00520
FUN_005395	Glycoside hydrolase family 16 protein	KRE6	-	ko:K21850	-
FUN_005396	Belongs to the thiamine pyrophosphokinase family	THI80	2.7.6.2	ko:K00949	ko00730, ko01100, map00730, map01100
FUN_005399	Up-frameshift suppressor 2	NMD2	-	ko:K14327	ko03013, ko03015, map03013, map03015
FUN_005400	FAD dependent oxidoreductase	-	-	ko:K20298	-
FUN_005401	Ammonium transporter	-	-	ko:K03320	-
FUN_005402	FES	-	-	ko:K03575	ko03410, map03410

FUN_005403	Belongs to the carnitine choline acetyltransferase family	CAT2	2.3.1.7	ko:K00624	ko04146, map04146
FUN_005404	Malate dehydrogenase	MDH1	1.1.1.37	ko:K00026	ko00020, ko00270, ko00620, ko00630, ko00710, ko01100, ko01110, ko01120, ko01130, ko01200, map00020, map00270, map00620, map00630, map00710, map01100, map01110, map01120, map01130, map01200
FUN_005408	Whi5 like	-	-	-	-
FUN_005410	NADPH adrenodoxin oxidoreductase, mitochondrial	ARH1	1.18.1.6	ko:K18914	-
FUN_005411	ABC transporter	TEF3	-	ko:K03235	-
FUN_005412	Mitochondrial branched-chain alpha-ketoacid dehydrogenase kinase	-	-	-	-
FUN_005413	peptidase inhibitor i9	-	-	-	-
FUN_005414	AARP2CN (NUC121) domain	BMS1	-	ko:K14569	ko03008, map03008
FUN_005415	Acetyl-CoA hydrolase/transferase C-terminal domain	ACH1	3.1.2.1	ko:K01067	ko00620, map00620
FUN_005416	Palmitoyl protein thioesterase	-	3.1.2.22	ko:K01074	ko00062, ko01100, ko01212, ko04142, map00062, map01100, map01212, map04142
FUN_005417	Ribosomal protein L1p/L10e family	CIC1	-	ko:K14775, ko:K14783	-
FUN_005418	transcription factor Tos4	TOS4	-	-	-
FUN_005422	Zinc-finger of RNA-polymerase I-specific TFIIB, Rrn7	-	-	ko:K15219	-
FUN_005423	eukaryotic translation initiation factor 5A	ANB1	-	ko:K03263	-
FUN_005424	Vps52 / Sac2 family	VPS52	-	ko:K20298	-
FUN_005425	fermentation associated protein	CSF1	-	-	-
FUN_005427	regulator medusa	-	-	-	-
FUN_005430	Mitochondrial K+-H+ exchange-related	-	-	-	-
FUN_005431	Saccharomyces cerevisiae YDL133W	-	-	-	-
FUN_005432	Belongs to the cullin family	CDC53	-	ko:K03347	ko04110, ko04111, ko04114, ko04120, ko04141, ko04310, ko04340, ko04341, ko04350, ko04710, ko05168, ko05200, map04110, map04111, map04114, map04120, map04141, map04310, map04340, map04341, map04350, map04710, map05168, map05200
FUN_005434	Aldehyde dehydrogenase family	-	-	-	-
FUN_005435	Heterokaryon incompatibility protein (HET)	-	-	-	-
FUN_005436	Belongs to the cyclin family	CTK2	3.2.1.21	ko:K05349, ko:K15564	ko00460, ko00500, ko00940, ko01100, ko01110, map00460, map00500, map00940, map01100, map01110
FUN_005437	Zinc-binding dehydrogenase	-	-	-	-
FUN_005438	Major Facilitator Superfamily	-	-	-	-
FUN_005439	Major Facilitator Superfamily	-	-	-	-
FUN_005440	Belongs to the glycosyl hydrolase 7 (cellulase C) family	-	3.2.1.4	ko:K19357	ko00500, ko01100, map00500, map01100
FUN_005442	serum paraoxonase arylesterase	-	3.1.1.2, 3.1.8.1	ko:K01045	ko01120, map01120
FUN_005443	Secretory lipase	-	-	-	-
FUN_005445	Tetratricopeptide repeat	-	-	-	-
FUN_005446	Glutamine-serine rich protein MS8	-	-	-	-
FUN_005449	Isocitrate/isopropylmalate dehydrogenase	-	-	-	-

FUN_005450	Glycolipid transfer protein (GLTP)	-	-	-	-
FUN_005452	Belongs to the pirin family	-	-	ko:K06911	-
FUN_005453	Major Facilitator Superfamily	-	-	-	-
FUN_005454	Involved in biosynthesis of the thiamine precursor thiazole. Catalyzes the conversion of NAD and glycine to adenosine diphosphate 5-(2-hydroxyethyl)-4-methylthiazole-2-carboxylic acid (ADT), an adenylated thiazole intermediate. The reaction includes an iron-dependent sulfide transfer from a conserved cysteine residue of the protein to a thiazole intermediate. The enzyme can only undergo a single turnover, which suggests it is a suicide enzyme. May have additional roles in adaptation to various stress conditions and in DNA damage tolerance	THI4	-	ko:K03146	ko00730, ko01100, map00730, map01100
FUN_005456	Rhodanese Homology Domain	-	-	-	-
FUN_005457	K homology RNA-binding domain	-	-	-	-
FUN_005459	Haloacid dehalogenase-like hydrolase	SDT1	-	ko:K07025, ko:K18551	ko00760, map00760
FUN_005460	Peptidase of plants and bacteria	-	-	-	-
FUN_005462	DUF4210	-	-	-	-
FUN_005463	ABC transporter transmembrane region	-	3.6.3.44	ko:K05658	ko02010, ko04976, ko05206, ko05226, map02010, map04976, map05206, map05226
FUN_005465	GTPase-activator protein for Rho-like GTPases	RGA2	-	ko:K16733, ko:K19839	ko04011, map04011
FUN_005466	Isocitrate dehydrogenase NAD subunit, mitochondrial	IDH2	1.1.1.41	ko:K00030	ko00020, ko01100, ko01110, ko01120, ko01130, ko01200, ko01210, ko01230, map00020, map01100, map01110, map01120, map01130, map01200, map01210, map01230
FUN_005467	TatD related DNase	scn1	-	-	-
FUN_005468	KR domain	-	-	-	-
FUN_005470	Guanine nucleotide-binding protein alpha-3 subunit	GPA2	-	ko:K04630, ko:K04640	ko04015, ko04022, ko04024, ko04062, ko04071, ko04261, ko04360, ko04371, ko04540, ko04611, ko04670, ko04713, ko04723, ko04724, ko04725, ko04726, ko04727, ko04728, ko04730, ko04914, ko04915, ko04916, ko04921, ko04923, ko04924, ko04926, ko04934, ko04971, ko05012, ko05030, ko05032, ko05034, ko05133, ko05142, ko05145, ko05200, map04015, map04022, map04024, map04062, map04071, map04261, map04360, map04371, map04540, map04611, map04670, map04713, map04723, map04724, map04725, map04726, map04727, map04728, map04730, map04914, map04915, map04916, map04921, map04923, map04924, map04926, map04934, map04971, map05012, map05030, map05032, map05034, map05133, map05142, map05145, map05200

FUN_005471	Mitogen-activated protein kinase	HOG1	2.7.11.24	ko:K04441	ko01522, ko04010, ko04011, ko04013, ko04015, ko04068, ko04071, ko04139, ko04212, ko04218, ko04261, ko04370, ko04380, ko04550, ko04611, ko04620, ko04621, ko04622, ko04624, ko04657, ko04658, ko04659, ko04660, ko04664, ko04668, ko04670, ko04714, ko04722, ko04723, ko04728, ko04750, ko04912, ko04914, ko04917, ko04926, ko04933, ko05014, ko05120, ko05131, ko05132, ko05133, ko05140, ko05142, ko05145, ko05152, ko05160, ko05164, ko05167, ko05169, ko05205, ko05418, map01522, map04010, map04011, map04013, map04015, map04068, map04071, map04139, map04212, map04218, map04261, map04370, map04380, map04550, map04611, map04620, map04621, map04622, map04624, map04657, map04658, map04659, map04660, map04664, map04668, map04670, map04714, map04722, map04723, map04728, map04750, map04912, map04914, map04917, map04926, map04933, map05014, map05120, map05131, map05132, map05133, map05140, map05142, map05145, map05152, map05160, map05164, map05167, map05169, map05205, map05418
FUN_005472	Transport protein Avl9	-	-	-	-
FUN_005474	Kinetochore complex Sim4 subunit Fta1	-	-	-	-
FUN_005475	Belongs to the heat shock protein 70 family	SSE1	-	ko:K09485, ko:K09489	ko04141, ko04530, ko04612, map04141, map04530, map04612
FUN_005476	Glycosyl hydrolase family 115	-	-	-	-
FUN_005478	Appr-1'-p processing enzyme	-	-	-	-
FUN_005479	Methyltransferase FkbM domain	-	-	-	-
FUN_005480	Sugar-tranasporters, 12 TM	-	-	-	-
FUN_005481	CLASP N terminal	STU1	-	ko:K16578	-
FUN_005482	Family of unknown function (DUF5353)	-	-	-	-
FUN_005485	Carbohydrate esterase family 1 protein	-	-	-	-
FUN_005487	tyrosinase	-	1.14.18.1	ko:K00505	ko00350, ko00950, ko00965, ko01100, ko01110, ko04916, map00350, map00950, map00965, map01100, map01110, map04916
FUN_005488	Ferric reductase like transmembrane component	-	-	-	-
FUN_005490	Belongs to the tannase family	faeB-1	3.1.1.73	ko:K09252	-
FUN_005491	Belongs to the cytochrome P450 family	ERG5	1.14.19.41	ko:K09831	ko00100, ko01100, ko01130, map00100, map01100, map01130
FUN_005494	Elongation factor G, domain IV	EFT2	-	ko:K03234	ko04152, ko04921, map04152, map04921
FUN_005496	Domain in the RNA-binding Lupus La protein; unknown function	-	-	ko:K11090	ko05322, map05322
FUN_005497	S4 RNA-binding domain	-	-	-	-
FUN_005499	MutL C terminal dimerisation domain	PMS1	-	ko:K10858	ko03430, ko03460, map03430, map03460
FUN_005500	Belongs to the MCM family	MCM3	3.6.4.12	ko:K02541	ko03030, ko04110, ko04111, ko04113, map03030, map04110, map04111, map04113
FUN_005501	Belongs to the glycosyl hydrolase 43 family	abnA	3.2.1.99	ko:K06113	-
FUN_005502	Belongs to the short-chain dehydrogenases reductases (SDR) family	-	-	-	-

FUN_005503	phospholipase	-	3.1.4.4	ko:K01115	ko00564, ko00565, ko01100, ko01110, ko04014, ko04024, ko04071, ko04072, ko04144, ko04666, ko04724, ko04912, ko05231, map00564, map00565, map01100, map01110, map04014, map04024, map04071, map04072, map04144, map04666, map04724, map04912, map05231
FUN_005504	Maintenance of telomere capping protein 6	MTC6	-	-	-
FUN_005505	Myb-like DNA-binding domain	-	-	ko:K09425, ko:K21769	ko04218, ko05166, map04218, map05166
FUN_005506	Glycosyltransferase family 41 protein	-	-	-	-
FUN_005507	Translocation protein Sec62	SEC62	-	ko:K12275	ko03060, ko04141, map03060, map04141
FUN_005509	Peptidase family m20 m25 m40 protein	-	-	-	-
FUN_005510	Nucleoside-diphosphate-sugar epimerase	-	-	-	-
FUN_005511	Alpha/beta hydrolase family	-	-	-	-
FUN_005515	Protein of unknown function (DUF3636)	-	-	-	-
FUN_005516	Belongs to the CDS family	CDS1	2.7.7.41	ko:K00981	ko00564, ko01100, ko01110, ko04070, map00564, map01100, map01110, map04070
FUN_005517	PPR repeat	-	-	-	-
FUN_005518	Dual specificity phosphatase, catalytic domain	TEP1	3.1.3.16, 3.1.3.48, 3.1.3.67	ko:K01104, ko:K01110	ko00562, ko01521, ko04068, ko04070, ko04071, ko04115, ko04140, ko04150, ko04151, ko04212, ko04218, ko04510, ko04931, ko05161, ko05165, ko05200, ko05206, ko05213, ko05214, ko05215, ko05218, ko05222, ko05224, ko05225, ko05230, map00562, map01521, map04068, map04070, map04071, map04115, map04140, map04150, map04151, map04212, map04218, map04510, map04931, map05161, map05165, map05200, map05206, map05213, map05214, map05215, map05218, map05222, map05224, map05225, map05230
FUN_005519	ATPase family associated with various cellular activities (AAA)	-	-	-	-
FUN_005520	Fungal specific transcription factor domain	-	-	-	-
FUN_005522	DJ-1/PfpI family	-	-	-	-
FUN_005525	Bacterial low temperature requirement A protein (LtrA)	-	-	-	-
FUN_005526	YEATS family	-	-	-	-
FUN_005527	Fungal specific transcription factor domain	-	-	-	-
FUN_005528	ABC transporter transmembrane region	-	3.6.3.44	ko:K05658	ko02010, ko04976, ko05206, ko05226, map02010, map04976, map05206, map05226

FUN_005530	Basic region leucine zipper	-	-	ko:K04450, ko:K09047	ko04010, ko04013, ko04022, ko04024, ko04151, ko04152, ko04211, ko04261, ko04624, ko04668, ko04714, ko04725, ko04728, ko04911, ko04915, ko04918, ko04922, ko04925, ko04926, ko04927, ko04931, ko04934, ko04962, ko05016, ko05030, ko05031, ko05034, ko05161, ko05164, ko05165, ko05166, ko05169, ko05203, ko05215, map04010, map04013, map04022, map04024, map04151, map04152, map04211, map04261, map04624, map04668, map04714, map04725, map04728, map04911, map04915, map04918, map04922, map04925, map04926, map04927, map04931, map04934, map04962, map05016, map05030, map05031, map05034, map05161, map05164, map05165, map05166, map05169, map05203, map05215
FUN_005531	Podospora anserina S mat genomic DNA chromosome	-	-	-	-
FUN_005532	Major Facilitator Superfamily	-	-	-	-
FUN_005533	Major Facilitator Superfamily	-	-	-	-
FUN_005534	GMC oxidoreductase	-	-	-	-
FUN_005535	short-chain dehydrogenase	RDH1	1.3.1.33	ko:K00218	ko00860, ko01100, ko01110, map00860, map01100, map01110
FUN_005536	Enoyl-(Acyl carrier protein) reductase	-	1.1.1.349	ko:K17644	ko00254, map00254
FUN_005537	Enoyl-(Acyl carrier protein) reductase	-	-	-	-
FUN_005538	Protein of unknown function (DUF3237)	-	-	ko:K15218	-
FUN_005539	Carbohydrate esterase family 15 protein	-	-	-	-
FUN_005541	cytokinin dehydrogenase activity	-	-	-	-
FUN_005546	Belongs to the glycosyl hydrolase 31 family	-	3.2.1.177	ko:K01811	-
FUN_005547	Belongs to the major facilitator superfamily. Sugar transporter (TC 2.A.1.1) family	-	-	-	-
FUN_005548	Glycoside hydrolase family 3 protein	-	3.2.1.21	ko:K05349	ko00460, ko00500, ko00940, ko01100, ko01110, map00460, map00500, map00940, map01100, map01110
FUN_005549	KR domain	-	-	-	-
FUN_005550	DASH complex subunit Ask1	-	-	ko:K11566	-
FUN_005551	Phosphatidylinositol 3- and 4-kinase	lsb6	2.7.1.67	ko:K13711	ko00562, ko01100, ko04070, map00562, map01100, map04070
FUN_005552	SNF2 family N-terminal domain	-	2.7.1.67	ko:K13711	ko00562, ko01100, ko04070, map00562, map01100, map04070
FUN_005553	Major Facilitator Superfamily	-	-	-	-
FUN_005554	Endonuclease/Exonuclease/phosphatase family	-	-	-	-
FUN_005555	Capsular polysaccharide synthesis protein	-	-	-	-
FUN_005557	Cellulose-growth-specific protein	-	-	-	-
FUN_005562	PPR repeat	-	3.6.4.12	ko:K02541, ko:K17669	ko03030, ko04110, ko04111, ko04113, map03030, map04110, map04111, map04113
FUN_005563	Signal-recognition-particle assembly has a crucial role in targeting secretory proteins to the rough endoplasmic reticulum membrane	SRP68	-	ko:K03107	ko03060, map03060
FUN_005564	zinc finger binding to DNA consensus sequence [AT]GATA[AG]	GZF3	-	ko:K09184	-
FUN_005565	alpha/beta hydrolase fold	-	-	-	-

FUN_005566	Belongs to the ATPase C chain family	atp9	1.6.5.3	ko:K02128, ko:K03880	ko00190, ko01100, ko04714, ko04723, ko05010, ko05012, ko05016, map00190, map01100, map04714, map04723, map05010, map05012, map05016
FUN_005568	Belongs to the peptidase C2 family	-	-	-	-
FUN_005569	Acetyltransferase (GNAT) family	-	2.3.1.183	ko:K03823	ko00440, ko01130, map00440, map01130
FUN_005570	Phosphoribosyl synthetase-associated domain	PRS1	2.7.6.1	ko:K00948	ko00030, ko00230, ko01100, ko01110, ko01120, ko01130, ko01200, ko01230, map00030, map00230, map01100, map01110, map01120, map01130, map01200, map01230
FUN_005571	Amidohydrolase	-	-	-	-
FUN_005572	Belongs to the major facilitator superfamily. Sugar transporter (TC 2.A.1.1) family	-	-	-	-
FUN_005573	Fungal specific transcription factor domain	-	-	-	-
FUN_005579	Sir2 family	-	-	-	-
FUN_005580	Serine carboxypeptidase S28	-	3.4.14.2	ko:K01276	-
FUN_005581	Glycoside hydrolase family 128 protein	-	-	-	-
FUN_005582	Glyoxalase family	-	-	-	-
FUN_005585	Domain in Tre-2, BUB2p, and Cdc16p. Probable Rab-GAPs.	GYP8	-	ko:K20372	-
FUN_005586	Mono-functional DNA-alkylating methyl methanesulfonate N-term	-	-	-	-
FUN_005588	Restriction endonuclease	-	-	-	-
FUN_005589	Subunit 21 of Mediator complex	SRB7	-	ko:K15152	-
FUN_005591	Major Facilitator Superfamily	-	-	-	-
FUN_005592	Serine incorporator (Serinc)	TMS1	-	-	-
FUN_005598	Belongs to the heat shock protein 70 family	-	-	-	-
FUN_005599	AIG1 family	-	-	-	-
FUN_005606	FAD binding domain	-	-	-	-
FUN_005607	integral membrane protein	-	-	-	-
FUN_005608	Major Facilitator Superfamily	-	-	-	-
FUN_005609	Belongs to the tannase family	-	-	-	-
FUN_005610	copper radical oxidase	-	1.2.3.15	ko:K20929	-
FUN_005611	Thioredoxin	-	-	-	-
FUN_005612	Belongs to the glycosyl hydrolase 28 family	rgxC	-	-	-
FUN_005617	serine threonine-protein phosphatase	PPH3	3.1.3.16	ko:K15423	ko04922, map04922
FUN_005618	EXOIII	REX4	-	ko:K18327	-
FUN_005619	Belongs to the MYST (SAS MOZ) family	ESA1	2.3.1.48	ko:K11304	ko05166, map05166
FUN_005621	aaa family atpase	-	-	-	-
FUN_005622	Heterokaryon incompatibility protein (HET)	-	-	-	-
FUN_005623	EF hand	-	1.6.99.3	ko:K03885	ko00190, map00190
FUN_005624	chorismate mutase	ARO7	5.4.99.5	ko:K01850	ko00400, ko01100, ko01110, ko01130, ko01230, map00400, map01100, map01110, map01130, map01230
FUN_005625	Adds the first Dol-P-Man derived mannose in an alpha-1, 3 linkage to Man(5)GlcNAc(2)-PP-Dol	ALG3	2.4.1.258	ko:K03845	ko00510, ko00513, ko01100, map00510, map00513, map01100
FUN_005626	Translation initiation factor 1A / IF-1	-	-	ko:K15025	-
FUN_005627	Major Facilitator Superfamily	-	-	-	-
FUN_005628	Aldo/keto reductase family	-	-	-	-

FUN_005630	Heterokaryon incompatibility protein (HET)	-	-	-	-
FUN_005632	Mycolic acid cyclopropane synthetase	-	2.1.1.79	ko:K00574	-
FUN_005633	Ribonuclease H2 non-catalytic subunit (Ylr154p-like)	-	-	ko:K10745	ko03030, map03030
FUN_005634	NUC153 domain	ESF1	-	-	-
FUN_005636	Fungal specific transcription factor domain	-	-	-	-
FUN_005637	Belongs to the ABC transporter superfamily. ABCG family. PDR (TC 3.A.1.205)	-	-	-	-
FUN_005639	Histidine phosphatase superfamily (branch 1)	-	-	ko:K15206	-
FUN_005643	NAD(P)H-binding	-	-	-	-
FUN_005644	Vacuolar membrane-associated protein Iml1	IML1	-	ko:K20404	ko04150, map04150
FUN_005645	Folliculin-interacting protein N-terminus	-	-	-	-
FUN_005646	C6 zinc finger	-	-	-	-
FUN_005647	Major Facilitator Superfamily	-	-	ko:K22134	-
FUN_005648	Protein kinase domain	VPS15	2.7.11.1	ko:K08333	ko04136, ko04138, ko04140, ko04371, map04136, map04138, map04140, map04371
FUN_005649	Glycosyltransferase family 4 protein	ALG2	2.4.1.132, 2.4.1.257	ko:K03843	ko00510, ko00513, ko01100, map00510, map00513, map01100
FUN_005650	Domain of unknown function (DUF4385)	-	-	-	-
FUN_005651	Ras of Complex, Roc, domain of DAPkinase	ras1	-	ko:K07827	ko01521, ko01522, ko04010, ko04012, ko04013, ko04014, ko04015, ko04062, ko04068, ko04071, ko04072, ko04137, ko04138, ko04140, ko04150, ko04151, ko04210, ko04211, ko04213, ko04214, ko04218, ko04320, ko04360, ko04370, ko04371, ko04540, ko04550, ko04650, ko04660, ko04662, ko04664, ko04714, ko04720, ko04722, ko04725, ko04726, ko04730, ko04810, ko04910, ko04912, ko04914, ko04915, ko04916, ko04917, ko04919, ko04921, ko04926, ko04933, ko04960, ko05034, ko05160, ko05161, ko05165, ko05166, ko05167, ko05200, ko05203, ko05205, ko05206, ko05210, ko05211, ko05212, ko05213, ko05214, ko05215, ko05216, ko05218, ko05219, ko05220, ko05221, ko05223, ko05224, ko05225, ko05226, ko05230, ko05231, map01521, map01522, map04010, map04012, map04013, map04014, map04015, map04062, map04068, map04071, map04072, map04137, map04138, map04140, map04150, map04151, map04210, map04211, map04213, map04214, map04218, map04320, map04360, map04370, map04371, map04540, map04550, map04650, map04660, map04662, map04664, map04714, map04720, map04722, map04725, map04726, map04730, map04810, map04910, map04912, map04914, map04915, map04916, map04917, map04919, map04921, map04926, map04933, map04960, map05034, map05160, map05161, map05165, map05166, map05167, map05200, map05203, map05205, map05206, map05210, map05211, map05212, map05213, map05214, map05215, map05216, map05218, map05219, map05220, map05221, map05223, map05224, map05225, map05226, map05230, map05231

FUN_005652	Probable molybdopterin binding domain	-	-	-	-
FUN_005653	Cofilin/tropomyosin-type actin-binding protein	-	-	-	-
FUN_005654	Heterokaryon incompatibility protein (HET)	-	-	-	-
FUN_005655	Heterokaryon incompatibility protein (HET)	-	-	-	-
FUN_005658	Leucine-rich repeat	LEA1	-	ko:K11092	ko03040, map03040
FUN_005659	Fip1 motif	-	-	ko:K14405	ko03015, map03015
FUN_005660	Nucleoporin FG repeat region	NUP49	1.8.5.7	ko:K07393, ko:K14307	ko03013, map03013
FUN_005661	Glutathione S-transferase, N-terminal domain	ECM4	1.8.5.7	ko:K07393	-
FUN_005662	Proteasome/cyclosome repeat	RPN1	-	ko:K03028	ko03050, ko05169, map03050, map05169
FUN_005663	Rio2, N-terminal	rio2	2.7.11.1	ko:K07179	ko03008, map03008
FUN_005664	RNA recognition motif	-	-	-	-
FUN_005665	belongs to the flavoprotein pyridine nucleotide cytochrome reductase family	cprA	1.6.2.4	ko:K00327	-
FUN_005666	Cell morphogenesis N-terminal	TAO3	-	-	-
FUN_005667	F-box-like	-	-	-	-
FUN_005670	Acts as an adapter for the XPO1 CRM1-mediated export of the 60S ribosomal subunit	NMD3	-	ko:K07562	ko03008, ko03013, map03008, map03013
FUN_005671	Ribosomal silencing factor during starvation	ATP25	-	-	-
FUN_005674	Glycoside hydrolase family 79 protein	-	-	-	-
FUN_005677	Ribosome 60S biogenesis N-terminal	-	-	ko:K14861	-
FUN_005678	Protein of unknown function (DUF3533)	-	-	-	-
FUN_005679	AMP-binding enzyme C-terminal domain	-	-	-	-
FUN_005682	to Saccharomyces cerevisiae ASI1 (YMR119W) and ASI3 (YNL008C)	-	-	-	-
FUN_005683	Glutathione-dependent formaldehyde-activating enzyme	-	-	-	-
FUN_005684	Belongs to the alpha-IPM synthase homocitrate synthase family	LYS21	2.3.3.14	ko:K01655	ko00300, ko00620, ko01100, ko01120, ko01130, ko01210, ko01230, map00300, map00620, map01100, map01120, map01130, map01210, map01230
FUN_005685	NUDIX domain	IDI1	5.3.3.2	ko:K01823	ko00900, ko01100, ko01110, ko01130, map00900, map01100, map01110, map01130
FUN_005686	Acetyltransferase (GNAT) domain	-	-	-	-
FUN_005688	Nuf2 family	NUF2	-	ko:K11548	-
FUN_005689	Belongs to the glycosyl hydrolase 18 family	CHT2	3.2.1.14	ko:K01183	ko00520, ko01100, map00520, map01100
FUN_005690	Belongs to the glycosyl hydrolase 18 family	CHT2	3.2.1.14	ko:K01183	ko00520, ko01100, map00520, map01100
FUN_005691	Belongs to the universal ribosomal protein uS12 family	-	-	ko:K02950	ko03010, map03010
FUN_005693	Belongs to the thioredoxin family	TRX1	-	ko:K03671	ko04621, ko05418, map04621, map05418
FUN_005695	zinc finger binding to DNA consensus sequence [AT]GATA[AG]	SFU1	-	-	-
FUN_005696	Belongs to the eIF-2B alpha beta delta subunits family	GCN3	-	ko:K03239	ko03013, map03013
FUN_005697	ankyrin repeat protein	-	-	-	-
FUN_005698	MatE	-	-	ko:K03327	-
FUN_005699	Belongs to the peptidase M28 family	-	-	-	-
FUN_005700	Pex2 / Pex12 amino terminal region	PEX12	-	ko:K06653, ko:K13345	ko04111, ko04146, map04111, map04146
FUN_005701	ammonium transporter	-	-	ko:K03320	-
FUN_005704	Pyruvate formate lyase activating enzyme	-	1.97.1.4	ko:K04070	-
FUN_005706	Alcohol dehydrogenase GroES-like domain	-	-	-	-

FUN_005707	chitin binding protein	-	-	-	-
FUN_005714	Rhomboid family	-	3.4.21.105	ko:K09650	-
FUN_005716	Histidine-specific methyltransferase, SAM-dependent	-	1.14.99.51, 2.1.1.44	ko:K20246	ko00340, map00340
FUN_005718	Catalyzes the first step of the methylation pathway of phosphatidylcholine biosynthesis, the SAM-dependent methylation of phosphatidylethanolamine (PE) to phosphatidylmonomethylethanolamine (PMME)	CHO2	2.1.1.17	ko:K16369	ko00564, ko01100, ko01110, map00564, map01100, map01110
FUN_005719	ATP-dependent serine protease that mediates the selective degradation of misfolded and unassembled polypeptides in the peroxisomal matrix. Necessary for type 2 peroxisome targeting signal (PTS2)-containing protein processing and facilitates peroxisome matrix protein import	-	3.4.21.53	ko:K01338	ko04112, map04112
FUN_005720	to uniprot P13393 Saccharomyces cerevisiae YER148W SPT15 TATA-binding protein general transcription factor that interacts with other factors to form the preinitiation complex at promoters essential for viability	tbp1	-	ko:K03120	ko03022, ko05016, ko05165, ko05166, ko05168, ko05169, ko05203, map03022, map05016, map05165, map05166, map05168, map05169, map05203
FUN_005727	Cytochrome P450	-	1.14.13.63	ko:K10438	ko00643, ko01120, map00643, map01120
FUN_005729	DNA-dependent RNA polymerase catalyzes the transcription of DNA into RNA using the four ribonucleoside triphosphates as substrates	RPO41	2.7.7.6	ko:K10908	-
FUN_005730	dioxygenase	-	-	ko:K19245	-
FUN_005731	mfs nicotinic acid transporter	-	-	-	-
FUN_005732	Belongs to the DapA family	-	4.1.3.16	ko:K18123	ko00330, ko00630, ko01100, map00330, map00630, map01100
FUN_005734	PHD zinc finger	-	-	ko:K11654	-
FUN_005735	Transcription-silencing protein, cryptic loci regulator Clr2	-	-	-	-
FUN_005736	Sugar (and other) transporter	-	-	-	-
FUN_005737	Metallopeptidase family M81	-	-	-	-
FUN_005738	Major Facilitator Superfamily	-	-	-	-
FUN_005739	Belongs to the cytochrome P450 family	-	-	-	-
FUN_005742	Terpene synthase	-	-	-	-
FUN_005743	Cytochrome P450	-	-	-	-
FUN_005744	Major Facilitator Superfamily	-	-	-	-
FUN_005745	integral membrane protein	-	-	-	-
FUN_005747	Belongs to the oxygen-dependent FAD-linked oxidoreductase family	-	-	-	-
FUN_005748	Belongs to the major facilitator superfamily. Sugar transporter (TC 2.A.1.1) family	-	3.5.4.3	ko:K01487	ko00230, ko01100, map00230, map01100
FUN_005749	spindle pole body component	-	-	ko:K14849, ko:K16571	-
FUN_005750	Biotin and Thiamin Synthesis associated domain	BIO2	2.8.1.6	ko:K01012	ko00780, ko01100, map00780, map01100
FUN_005751	Cys/Met metabolism PLP-dependent enzyme	-	2.3.1.47	ko:K00652	ko00780, ko01100, map00780, map01100
FUN_005752	AAA domain	bioDA	2.6.1.62, 6.3.3.3	ko:K00833, ko:K19562	ko00780, ko01100, map00780, map01100
FUN_005753	tRNA intron endonuclease, catalytic C-terminal domain	SEN34	3.1.27.9	ko:K15323	-
FUN_005754	PAXNEB protein	ELP4	-	ko:K11375	-
FUN_005756	RNA polymerase Rpb8	RPB8	-	ko:K03016	ko00230, ko00240, ko01100, ko03020, ko04623, ko05016, ko05169, map00230, map00240, map01100, map03020, map04623, map05016, map05169
FUN_005757	Domain of unknown function (DUF4743)	-	2.7.6.2	ko:K00949	ko00730, ko01100, map00730, map01100

FUN_005758	S-adenosyl-L-methionine-dependent transferase that acts as a component of the wybutosine biosynthesis pathway. Wybutosine is a hyper modified guanosine with a tricyclic base found at the 3'-position adjacent to the anticodon of eukaryotic phenylalanine tRNA. Catalyzes the transfer of the alpha-amino-alpha- carboxypropyl (acp) group from S-adenosyl-L-methionine to the C-7 position of 4-demethylwyosine (imG-14) to produce wybutosine-86	TRM12	2.5.1.114	ko:K07055	-
FUN_005760	Nuclear pore protein 84 / 107	NUP84	-	ko:K14301	ko03013, map03013
FUN_005761	Ubiquitin homologues	SMT3	-	ko:K12160	ko03013, ko05418, map03013, map05418
FUN_005763	CoA binding domain	-	6.2.1.4, 6.2.1.5	ko:K01899	ko00020, ko00640, ko01100, ko01110, ko01120, ko01130, ko01200, map00020, map00640, map01100, map01110, map01120, map01130, map01200
FUN_005764	KilA-N	-	-	-	-
FUN_005765	RHO protein GDP dissociation inhibitor	RDI1	-	ko:K12462	ko04722, ko04962, map04722, map04962
FUN_005767	non-SMC mitotic condensation complex subunit 1	APL6	-	ko:K12397	ko04142, map04142
FUN_005769	serine threonine-protein phosphatase	PPG1	3.1.3.16	ko:K17615	-
FUN_005770	Belongs to the uridine kinase family	URK1	2.7.1.48	ko:K00876	ko00240, ko00983, ko01100, map00240, map00983, map01100
FUN_005771	CT11-RanBPM	-	-	-	-
FUN_005772	Belongs to the MCM family	MCM6	3.6.4.12	ko:K02542	ko03030, ko04110, ko04111, ko04113, map03030, map04110, map04111, map04113
FUN_005773	serine threonine-protein phosphatase	-	3.1.3.16	ko:K04382	ko03015, ko04013, ko04071, ko04111, ko04113, ko04114, ko04136, ko04138, ko04140, ko04151, ko04152, ko04261, ko04350, ko04390, ko04391, ko04530, ko04728, ko04730, ko05142, ko05160, ko05165, map03015, map04013, map04071, map04111, map04113, map04114, map04136, map04138, map04140, map04151, map04152, map04261, map04350, map04390, map04391, map04530, map04728, map04730, map05142, map05160, map05165
FUN_005774	Ulp1 protease family, C-terminal catalytic domain	-	-	-	-
FUN_005775	Zinc-binding dehydrogenase	-	-	-	-
FUN_005777	Belongs to the GDA1 CD39 NTPase family	YND1	3.6.1.5	ko:K14642	ko00230, ko00240, map00230, map00240
FUN_005778	Glutamine amidotransferase class-I	-	3.4.19.16, 6.3.5.2	ko:K01951, ko:K22314	ko00230, ko00983, ko01100, map00230, map00983, map01100
FUN_005779	Fungal trichothecene efflux pump (TRI12)	-	-	-	-
FUN_005780	This magnesium-dependent enzyme catalyzes the hydrolysis of ATP coupled with the transport of calcium	PMC1	3.6.3.8	ko:K01537	-
FUN_005781	DASH complex subunit Dad1	DAD1	-	ko:K11553	-
FUN_005782	Belongs to the small GTPase superfamily. Rho family	rho4	-	ko:K07975	-
FUN_005783	Acid phosphatase homologues	LCB3	-	ko:K04716, ko:K04717	ko00600, ko04071, map00600, map04071
FUN_005784	Secretory pathway protein Ssp120	-	-	-	-
FUN_005785	Phosphate acyltransferases	-	-	-	-
FUN_005786	GAL4-like Zn(II)2Cys6 (or C6 zinc) binuclear cluster DNA-binding domain	-	-	-	-
FUN_005787	WD40 repeats	asa1	-	-	-
FUN_005788	ABC transporter	-	-	-	-

FUN_005789	Wyosine base formation	YPO7	4.1.3.44	ko:K15449	-
FUN_005790	Phosphoadenosine phosphosulfate reductase family	FAD1	2.7.7.2	ko:K00953	ko00740, ko01100, ko01110, map00740, map01100, map01110
FUN_005791	Pyruvate flavodoxin/ferredoxin oxidoreductase, thiamine diP-bdg	MET5	1.8.1.2	ko:K00381	ko00920, ko01100, ko01120, map00920, map01100, map01120
FUN_005792	Belongs to the Ca(2) cation antiporter (CaCA) (TC 2.A.19) family	-	-	ko:K07300	-
FUN_005793	SNF2 family N-terminal domain	-	-	ko:K15173	ko04918, map04918
FUN_005794	Fungal specific transcription factor domain	-	-	-	-
FUN_005796	Putative DNA-binding (bihelical) motif predicted to be involved in chromosomal organisation	RAD18	2.3.2.27	ko:K10627	-
FUN_005797	Regulatory subunit of the dimeric UBA3-ULA1 E1 enzyme	-	-	ko:K04532	ko05010, map05010
FUN_005798	Ribose 5-phosphate isomerase A (phosphoriboisomerase A)	RKI1	5.3.1.6	ko:K01807	ko00030, ko00710, ko01100, ko01110, ko01120, ko01130, ko01200, ko01230, map00030, map00710, map01100, map01110, map01120, map01130, map01200, map01230
FUN_005800	Lytic polysaccharide monooxygenase	-	-	-	-
FUN_005802	ER-Golgi trafficking TRAPP I complex 85 kDa subunit	-	-	ko:K20305	-
FUN_005803	Belongs to the tRNA nucleotidyltransferase poly(A) polymerase family	CCA1	2.7.7.72	ko:K00974	ko03013, map03013
FUN_005804	FAD dependent oxidoreductase	DAO1	1.4.3.1, 1.4.3.3	ko:K00272, ko:K00273	ko00250, ko00260, ko00311, ko00330, ko00472, ko01100, ko01130, ko04146, map00250, map00260, map00311, map00330, map00472, map01100, map01130, map04146
FUN_005805	OTU-like cysteine protease	-	3.4.19.12	ko:K12655, ko:K13717	ko04622, map04622
FUN_005806	C-8 sterol isomerase	ERG2	-	ko:K09829	ko00100, ko01100, ko01130, map00100, map01100, map01130
FUN_005807	Fungal Zn(2)-Cys(6) binuclear cluster domain	-	-	-	-
FUN_005812	Mitochondrial F1-F0 ATP synthase subunit F of fungi	ATP17	-	ko:K02139	ko00190, ko01100, map00190, map01100
FUN_005813	ETC complex I subunit conserved region	NdufS4	-	ko:K03937	ko00190, ko01100, ko04714, ko04723, ko04932, ko05010, ko05012, ko05016, map00190, map01100, map04714, map04723, map04932, map05010, map05012, map05016
FUN_005814	CBD_II	-	-	-	-
FUN_005817	Pyridoxamine 5'-phosphate oxidase	-	1.4.3.5	ko:K00275	ko00750, ko01100, ko01120, map00750, map01100, map01120
FUN_005818	DNA polymerase subunit Cdc27	-	-	ko:K03504	ko00230, ko00240, ko01100, ko03030, ko03410, ko03420, ko03430, ko03440, ko05166, map00230, map00240, map01100, map03030, map03410, map03420, map03430, map03440, map05166
FUN_005819	Tubulin folding cofactor D C terminal	-	-	ko:K21767	-
FUN_005821	Belongs to the STXBP unc-18 SEC1 family	sec1	-	ko:K15292	ko04721, map04721
FUN_005822	Mitochondrial degradasome RNA helicase subunit C terminal	SUV3	3.6.4.13	ko:K17675	-
FUN_005823	Inositolphosphorylceramide synthase subunit Kei1	-	-	-	-
FUN_005824	Enoyl-(Acyl carrier protein) reductase	-	-	-	-
FUN_005825	Uncharacterized conserved protein (DUF2293)	-	-	-	-
FUN_005826	Domain of unknown function (DUF814)	-	-	-	-
FUN_005827	Acetyltransferase (GNAT) family	-	-	-	-
FUN_005828	Protein tyrosine kinase	NPR1	-	-	-

FUN_005829	Svf1-like C-terminal lipocalin-like domain	SVF1	-	-	-
FUN_005830	DNA mismatch repair protein Mlh1 C-terminus	mlh1	-	ko:K08734	ko01524, ko03430, ko03460, ko05200, ko05210, ko05213, ko05226, map01524, map03430, map03460, map05200, map05210, map05213, map05226
FUN_005831	Hamartin protein	-	-	-	-
FUN_005832	GDSL-like Lipase/Acylhydrolase family	-	-	-	-
FUN_005833	TAP42-like family	TAP42	-	ko:K17606	ko04136, ko04138, ko04140, map04136, map04138, map04140
FUN_005834	Belongs to the helicase family. Dicer subfamily	dcl1	-	ko:K11592	ko05206, map05206
FUN_005835	Major Facilitator Superfamily	-	-	-	-
FUN_005836	RNA recognition motif. (a.k.a. RRM, RBD, or RNP domain)	-	-	ko:K11094	ko03040, map03040
FUN_005837	ATP synthase subunit D	VMA8	-	ko:K02149	ko00190, ko01100, ko04145, ko04150, ko04721, ko04966, ko05110, ko05120, ko05323, map00190, map01100, map04145, map04150, map04721, map04966, map05110, map05120, map05323
FUN_005838	HAT (Half-A-TPR) repeats	SYF1	-	ko:K12867	ko03040, map03040
FUN_005840	aaa family atpase	-	-	-	-
FUN_005843	TFIIA is a component of the transcription machinery of RNA polymerase II and plays an important role in transcriptional activation	toa2	-	ko:K03123	ko03022, ko05203, map03022, map05203
FUN_005844	Belongs to the V-ATPase proteolipid subunit family	vma16	-	ko:K03661	ko00190, ko01100, ko04142, ko04145, ko04721, ko05110, ko05120, ko05152, ko05323, map00190, map01100, map04142, map04145, map04721, map05110, map05120, map05152, map05323
FUN_005845	Belongs to the V-ATPase proteolipid subunit family	-	2.10.1.1, 2.7.7.75	ko:K03750, ko:K15376	ko00790, ko01100, ko04727, map00790, map01100, map04727
FUN_005848	CPSF A subunit region	-	-	ko:K10610	ko03420, ko04120, ko05161, ko05203, map03420, map04120, map05161, map05203
FUN_005849	Sin-like protein conserved region	-	-	ko:K14721	ko00230, ko00240, ko03020, ko04623, ko05169, map00230, map00240, map03020, map04623, map05169
FUN_005850	Belongs to the 3-hydroxyisobutyrate dehydrogenase family	-	1.1.1.31	ko:K00020	ko00280, ko01100, map00280, map01100
FUN_005851	Proteolipid membrane potential modulator	-	-	-	-
FUN_005852	Protein of unknown function (DUF1688)	-	-	-	-
FUN_005853	Phosphoenolpyruvate phosphomutase	-	-	-	-
FUN_005854	TAP-like protein	-	-	-	-
FUN_005855	Zinc-binding dehydrogenase	ZTA1	1.6.5.5	ko:K00344	-
FUN_005856	Splits internally a 1, 3-beta-glucan molecule and transfers the newly generated reducing end (the donor) to the non- reducing end of another 1, 3-beta-glucan molecule (the acceptor) forming a 1, 3-beta linkage, resulting in the elongation of 1, 3- beta-glucan chains in the cell wall	GAS4	-	-	-
FUN_005857	Sec7 domain	GEA2	-	ko:K18443	ko04144, map04144
FUN_005858	Glycosyltransferase family 62 protein	ANP1	-	ko:K05533	ko00513, ko01100, map00513, map01100
FUN_005859	3' exoribonuclease family, domain 1	MTR3	-	ko:K12587	ko03018, map03018
FUN_005861	Exocyst component 84 C-terminal	EXO84	-	ko:K19986	-
FUN_005862	MAS20 protein import receptor	TOM20	-	ko:K17770	-
FUN_005863	Signal recognition particle receptor beta subunit	ryh1	-	ko:K07893	-

FUN_005864	Nop53 (60S ribosomal biogenesis)	-	-	ko:K14840, ko:K18327	ko05168, map05168
FUN_005865	Required for endonucleolytic cleavage during polyadenylation-dependent pre-mRNA 3'-end formation	CLP1	2.7.1.78	ko:K14399	ko03015, map03015
FUN_005866	Belongs to the inositol phosphokinase (IPK) family	-	2.7.1.140, 2.7.1.151, 2.7.1.78	ko:K00915, ko:K14399	ko00562, ko01100, ko03015, ko04070, ko04138, map00562, map01100, map03015, map04070, map04138
FUN_005867	Argonaute siRNA chaperone (ARC) complex subunit Arb1	-	-	-	-
FUN_005868	Protein of unknown function (DUF775)	-	-	-	-
FUN_005870	Pyridoxine 5'-phosphate oxidase C-terminal dimerisation region	PDX3	1.4.3.5	ko:K00275	ko00750, ko01100, ko01120, map00750, map01100, map01120
FUN_005872	Domain of unknown function UPF0086	-	3.1.26.5	ko:K03538	ko03008, ko03013, map03008, map03013
FUN_005873	Glycoside hydrolase family 125 protein	-	-	ko:K09704	-
FUN_005874	HD domain	-	-	-	-
FUN_005875	Belongs to the asparaginase 1 family	-	3.5.1.1	ko:K01424	ko00250, ko00460, ko01100, ko01110, map00250, map00460, map01100, map01110
FUN_005876	Oxysterol-binding protein	-	-	ko:K20465	-
FUN_005877	integral membrane protein	-	-	-	-
FUN_005879	tpr domain protein	-	-	-	-
FUN_005881	Glycosyl hydrolase family 61	-	-	-	-
FUN_005883	Taurine catabolism dioxygenase TauD, TfdA family	-	1.14.11.17	ko:K03119, ko:K19245	ko00430, ko00920, map00430, map00920
FUN_005884	Glycosyltransferase family 15 protein	KRE2	-	ko:K10967	ko00514, map00514
FUN_005885	Topoisomerase II-associated protein PAT1	PAT1	-	ko:K12617	ko03018, map03018
FUN_005886	Belongs to the peptidase A1 family	-	3.4.23.18	ko:K06004	-
FUN_005887	Helicase associated domain (HA2) Add an annotation	PRP22	3.6.4.13	ko:K12818	ko03040, map03040
FUN_005888	Alcohol dehydrogenase GroES-like domain	ADH1	1.1.1.1	ko:K13953	ko00010, ko00071, ko00350, ko00625, ko00626, ko00830, ko00980, ko00982, ko01100, ko01110, ko01120, ko01130, ko01220, map00010, map00071, map00350, map00625, map00626, map00830, map00980, map00982, map01100, map01110, map01120, map01130, map01220
FUN_005890	Belongs to the hexokinase family	-	-	-	-
FUN_005891	Gamma interferon inducible lysosomal thiol reductase (GILT)	-	-	-	-
FUN_005892	Protein of unknown function (DUF1681)	-	-	ko:K20069	-
FUN_005894	haloacid dehalogenase-like hydrolase	-	-	-	-
FUN_005895	Required for the maintenance of the structure of the mitochondrial inner membrane. Involved in mitochondrial morphology	SHE9	-	ko:K17983	-
FUN_005896	Fungal specific transcription factor domain	-	-	ko:K21451	-
FUN_005897	Belongs to the aldehyde dehydrogenase family	-	-	-	-
FUN_005900	Belongs to the glycosyl hydrolase 7 (cellulase C) family	-	3.2.1.4, 3.2.1.91	ko:K01225, ko:K19357	ko00500, ko01100, map00500, map01100
FUN_005901	Belongs to the SNF7 family	-	-	ko:K15053	ko04144, ko04217, map04144, map04217
FUN_005902	Conserved hypothetical ATP binding protein	-	-	ko:K06883	-
FUN_005903	WD domain, G-beta repeat	BUB3	-	ko:K02180	ko04110, ko04111, ko05166, map04110, map04111, map05166

FUN_005905	Component of ribonuclease P, a protein complex that generates mature tRNA molecules by cleaving their 5'-ends. Also a component of RNase MRP	POP5	3.1.26.5	ko:K03537	ko03008, ko03013, map03008, map03013
FUN_005906	Belongs to the synaptobrevin family	-	-	ko:K08515	ko04130, map04130
FUN_005907	Carbamoyl-phosphate synthase small chain, CPSase domain	URA2	2.1.3.2, 6.3.5.5	ko:K01954, ko:K11541	ko00240, ko00250, ko01100, map00240, map00250, map01100
FUN_005908	Ion transport protein	-	-	-	-
FUN_005909	RTA1 like protein	-	-	-	-
FUN_005910	Alpha/beta hydrolase family	-	-	-	-
FUN_005911	Belongs to the sterol desaturase family	-	-	-	-
FUN_005912	Belongs to the sterol desaturase family	-	-	-	-
FUN_005913	Belongs to the eukaryotic ribosomal protein eL6 family	RPL6	-	ko:K02934	ko03010, map03010
FUN_005914	Proteasome complex subunit Rpn13 ubiquitin receptor	-	-	ko:K06691	ko03050, ko05169, map03050, map05169
FUN_005915	Belongs to the oxygen-dependent FAD-linked oxidoreductase family	-	-	-	-
FUN_005916	Belongs to the glycosyl hydrolase 11 (cellulase G) family	-	3.2.1.8	ko:K01181	-
FUN_005917	epoxide hydrolase	-	3.1.3.76, 3.3.2.10	ko:K08726	ko00590, ko00625, ko01100, ko01120, ko04146, map00590, map00625, map01100, map01120, map04146
FUN_005918	ammonium transporter	MEP1	-	ko:K03320	-
FUN_005920	Glycoside hydrolase family 128 protein	-	-	-	-
FUN_005921	ML-like domain	-	-	-	-
FUN_005923	Vacuolar protein sorting-associated protein 62	VPS62	-	-	-
FUN_005925	Major Facilitator Superfamily	-	-	-	-
FUN_005927	Isocitrate lyase family	-	-	-	-
FUN_005929	Fungal specific transcription factor domain	-	-	-	-
FUN_005930	Belongs to the major facilitator superfamily. Sugar transporter (TC 2.A.1.1) family	-	-	-	-
FUN_005931	Short chain dehydrogenase	-	1.1.1.289	ko:K17742	ko00051, map00051
FUN_005932	Alcohol dehydrogenase GroES-like domain	-	1.1.1.9	ko:K05351	ko00040, ko01100, map00040, map01100
FUN_005933	Phosphoribulokinase uridine kinase	-	-	-	-
FUN_005934	KR domain	-	1.1.1.289	ko:K17742	ko00051, map00051
FUN_005935	FANCL C-terminal domain	-	1.14.19.1, 2.3.2.27	ko:K00507, ko:K22377	ko01040, ko01212, ko03320, ko04152, ko04212, map01040, map01212, map03320, map04152, map04212
FUN_005936	Helical and beta-bridge domain	RAD3	3.6.4.12	ko:K10844	ko03022, ko03420, map03022, map03420
FUN_005937	Ornithine decarboxylase antizyme	-	-	-	-
FUN_005938	Peptidylprolyl isomerase	FPR3	5.2.1.8	ko:K14826	-
FUN_005939	Deoxycytidyl transferase involved in DNA repair. Transfers a dCMP residue from dCTP to the 3'-end of a DNA primer in a template-dependent reaction. May assist in the first step in the bypass of abasic lesions by the insertion of a nucleotide opposite the lesion. Required for normal induction of mutations by physical and chemical agents	REV1	-	ko:K03515	ko03460, map03460
FUN_005940	Belongs to the yippee family	-	-	-	-
FUN_005941	present in yeast cell wall integrity and stress response component proteins	-	-	-	-
FUN_005942	Serine/Threonine protein kinases, catalytic domain	-	-	ko:K00924	-
FUN_005944	Adenylyl- / guanylyl cyclase, catalytic domain	-	4.6.1.1	ko:K01768	ko00230, ko02025, ko04113, ko04213, map00230, map02025, map04113, map04213
FUN_005945	Gar1/Naf1 RNA binding region	-	-	ko:K14763	-

FUN_005946	Protein of unknown function (DUF3445)	-	2.4.1.259, 2.4.1.261	ko:K03846	ko00510, ko00513, ko01100, map00510, map00513, map01100
FUN_005947	Etoposide-induced protein 2.4 (EI24)	-	-	-	-
FUN_005948	Belongs to the peptidase A1 family	45748	3.4.23.25	ko:K01381	ko04138, map04138
FUN_005949	Involved in the biosynthesis of sterol glucoside	ATG26	2.4.1.173	ko:K05841	-
FUN_005950	catalytic domain of ctd-like phosphatases	fcp1	3.1.3.16	ko:K15732	-
FUN_005952	GAL4-like Zn(II)2Cys6 (or C6 zinc) binuclear cluster DNA-binding domain	-	-	-	-
FUN_005953	Belongs to the universal ribosomal protein uL11 family	MRPL19	-	ko:K02867	ko03010, map03010
FUN_005954	DNA replication and checkpoint protein	RPN12	-	ko:K03031	ko03050, ko05169, map03050, map05169
FUN_005955	DNA replication and checkpoint protein	RPN12	-	ko:K03031	ko03050, ko05169, map03050, map05169
FUN_005956	Dihydrolipoyl dehydrogenase	LPD1	1.8.1.4	ko:K00382, ko:K17583	ko00010, ko00020, ko00260, ko00280, ko00620, ko00630, ko00640, ko01100, ko01110, ko01120, ko01130, ko01200, map00010, map00020, map00260, map00280, map00620, map00630, map00640, map01100, map01110, map01120, map01130, map01200
FUN_005957	Domain in DAP-5, eIF4G, MA-3 and other proteins.	-	-	ko:K17583	-
FUN_005958	Signal recognition particle 14kD protein	-	-	ko:K03104	ko03060, map03060
FUN_005959	Dopa 4, 5-dioxygenase family	-	-	ko:K10253	-
FUN_005960	OB-fold nucleic acid binding domain	-	6.1.1.22	ko:K01893	ko00970, map00970
FUN_005961	Metallopeptidase family M24	-	-	-	-
FUN_005962	Metallopeptidase family M24	-	-	-	-
FUN_005963	Endonuclease/Exonuclease/phosphatase family	-	-	-	-
FUN_005964	SNF5 / SMARCB1 / INI1	SNF5	-	ko:K11648	ko04714, ko05225, map04714, map05225
FUN_005965	Golgi phosphoprotein 3 (GPP34)	VPS74	-	ko:K15620	-
FUN_005966	Carrier of the growing fatty acid chain in fatty acid biosynthesis	ACP2	-	ko:K03955	ko00190, ko01100, ko04714, ko04723, ko04932, ko05010, ko05012, ko05016, map00190, map01100, map04714, map04723, map04932, map05010, map05012, map05016
FUN_005967	60S ribosome biogenesis protein Rrp14	-	-	-	-
FUN_005969	Protein of unknown function (DUF3445)	-	2.4.1.259, 2.4.1.261	ko:K03846	ko00510, ko00513, ko01100, map00510, map00513, map01100
FUN_005970	Guanine nucleotide exchange factor for Ras-like GTPases; N-terminal motif	LTE1	-	ko:K06681	ko04111, map04111
FUN_005972	CorA-like Mg2+ transporter protein	-	-	-	-
FUN_005973	Transcription initiation factor TFIID subunit A	-	-	ko:K03126	ko03022, map03022
FUN_005974	Key enzyme for ketone body catabolism. Transfers the CoA moiety from succinate to acetoacetate. Formation of the enzyme-CoA intermediate proceeds via an unstable anhydride species formed between the carboxylate groups of the enzyme and substrate	-	2.8.3.5	ko:K01027	ko00072, ko00280, ko00650, map00072, map00280, map00650
FUN_005976	MIT (microtubule interacting and transport) domain	-	-	-	-
FUN_005977	PCI domain	-	-	ko:K12177	-
FUN_005978	Mad3/BUB1 hoMad3/BUB1 homology region 1	BUB1	2.7.11.1	ko:K02178, ko:K06680	ko04110, ko04111, ko04113, ko04114, ko04914, map04110, map04111, map04113, map04114, map04914
FUN_005979	Lectin family glycoprotein receptor	-	-	ko:K10080	ko04141, map04141
FUN_005980	SPFH domain / Band 7 family	PHB2	-	ko:K17081	-

FUN_005981	Belongs to the eukaryotic-type primase small subunit family	PRI1	-	ko:K02684	ko00230, ko00240, ko01100, ko03030, map00230, map00240, map01100, map03030
FUN_005982	Domain of unknown function (DUF1917)	-	-	-	-
FUN_005983	Amidohydrolase family	DAL1	3.5.2.5	ko:K01466	ko00230, ko01100, ko01120, map00230, map01100, map01120
FUN_005984	Zinc finger, C2H2 type	ACE2	-	ko:K09202, ko:K09238	ko04111, map04111
FUN_005986	Belongs to the cytochrome P450 family	-	-	-	-
FUN_005987	C-5 cytosine-specific DNA methylase	-	-	-	-
FUN_005989	Cell division protein 48 (CDC48), domain 2	SEC18	3.6.4.6	ko:K06027	ko04138, ko04721, ko04727, ko04962, map04138, map04721, map04727, map04962
FUN_005990	Uncharacterized conserved protein	-	-	ko:K22243	-
FUN_005991	Belongs to the eukaryotic ribosomal protein eL19 family	-	-	-	-
FUN_005992	Belongs to the eukaryotic ribosomal protein eL19 family	-	-	-	-
FUN_005993	Belongs to the eukaryotic ribosomal protein eL19 family	RPL19B	-	ko:K02885	ko03010, map03010
FUN_005994	Transcription initiation factor IID, 18kD subunit	SPT3	-	ko:K11313, ko:K19476	ko04144, ko05202, map04144, map05202
FUN_005995	Tetrapyrrole (Corrin/Porphyrin) Methylases	DPH5	2.1.1.314	ko:K00586	-
FUN_005998	CutC family	-	-	ko:K06201	-
FUN_005999	Occurs in almost all aerobically respiring organisms and serves to protect cells from the toxic effects of hydrogen peroxide	-	1.11.1.6	ko:K03781	ko00380, ko00630, ko01110, ko01130, ko01200, ko04011, ko04016, ko04068, ko04146, ko04211, ko04212, ko04213, ko05014, map00380, map00630, map01110, map01130, map01200, map04011, map04016, map04068, map04146, map04211, map04212, map04213, map05014
FUN_006000	Eisosome component PIL1	-	-	-	-
FUN_006001	Protein transporter sec24	SEC24	-	ko:K14007	ko04141, map04141
FUN_006002	zinc finger	USV1	-	ko:K21544	-
FUN_006003	Belongs to the TRAFAC class myosin-kinesin ATPase superfamily. Kinesin family	-	-	ko:K10403	ko04914, map04914
FUN_006004	60S ribosomal protein L30	RPL30	-	ko:K02908	ko03010, map03010
FUN_006005	Involved in autophagy and cytoplasm to vacuole transport (Cvt) vesicle formation. Plays a key role in the organization of the preautophagosomal structure phagophore assembly site (PAS), the nucleating site for formation of the sequestering vesicle	ATG9	-	ko:K17907	ko04136, ko04137, ko04138, ko04140, map04136, map04137, map04138, map04140
FUN_006006	Conserved oligomeric complex COG6	COG6	-	ko:K20293	-
FUN_006007	NIF3 (NGG1p interacting factor 3)	-	-	-	-
FUN_006008	Zinc-binding dehydrogenase	-	-	-	-
FUN_006009	Multicopper oxidase	-	-	-	-
FUN_006010	MRNA splicing factor RNA helicase (Cdc28)	-	3.6.4.13	ko:K12813, ko:K12814	ko03040, map03040
FUN_006011	Transcription factor IIIC subunit delta N-term	-	-	-	-
FUN_006012	Glycosyltransferase family 33 protein	ALG1	2.4.1.142	ko:K03842	ko00510, ko00513, ko01100, map00510, map00513, map01100
FUN_006013	Cation efflux family	MSC2	-	ko:K14692	-
FUN_006014	SET (Su(var)3-9, Enhancer-of-zeste, Trithorax) domain	-	-	-	-
FUN_006016	KR domain	-	-	-	-
FUN_006018	acyl-CoA dehydrogenase	-	1.7.3.1	ko:K19823	ko00910, map00910

FUN_006019	DASH complex subunit Spc34	-	-	-	-
FUN_006020	Proteasome regulatory subunit C-terminal	RPN3	-	ko:K03033, ko:K03126	ko03022, ko03050, ko05169, map03022, map03050, map05169
FUN_006021	Telomere recombination	-	2.7.7.87	ko:K07566	-
FUN_006022	Conserved region of Rad21 / Rec8 like protein	MCD1	-	ko:K06670	ko04110, ko04111, map04110, map04111
FUN_006023	Belongs to the cation transport ATPase (P-type) (TC 3.A.3) family	PMR1	3.6.3.8	ko:K01537, ko:K20291	-
FUN_006025	response to estrogen	-	-	-	-
FUN_006026	Interconversion of serine and glycine	-	2.1.2.1	ko:K00600	ko00260, ko00460, ko00630, ko00670, ko00680, ko01100, ko01110, ko01120, ko01130, ko01200, ko01230, ko01523, map00260, map00460, map00630, map00670, map00680, map01100, map01110, map01120, map01130, map01200, map01230, map01523
FUN_006028	tpr repeat protein	CNS1	-	ko:K02993	ko03010, map03010
FUN_006029	Meiotic cell cortex C-terminal pleckstrin homology	-	-	-	-
FUN_006031	Leucine carboxyl methyltransferase	PPM1	2.1.1.233	ko:K18203	-
FUN_006032	RNA polymerase Rpb5, C-terminal domain	RPB5	-	ko:K03013	ko00230, ko00240, ko01100, ko03020, ko04623, ko05016, ko05169, map00230, map00240, map01100, map03020, map04623, map05016, map05169
FUN_006033	3-hydroxyacyl-CoA dehydrogenase, C-terminal domain	-	-	-	-
FUN_006034	AMP-binding enzyme C-terminal domain	-	-	-	-
FUN_006035	Mitotic checkpoint protein	MAD1	2.1.1.233	ko:K06679, ko:K18203	ko04111, ko04113, ko04114, ko05166, map04111, map04113, map04114, map05166
FUN_006036	c6 finger domain protein	-	-	-	-
FUN_006037	Sas10/Utp3/C1D family	-	-	ko:K14765	-
FUN_006038	Belongs to the DEAD box helicase family	DBP3	3.6.4.13	ko:K14811	-

FUN_006039	Mitogen-activated protein kinase	tmk1	2.7.11.24	ko:K04371	ko01521, ko01522, ko01524, ko04010, ko04011, ko04012, ko04013, ko04014, ko04015, ko04022, ko04024, ko04062, ko04066, ko04068, ko04071, ko04072, ko04114, ko04140, ko04150, ko04151, ko04210, ko04214, ko04218, ko04261, ko04270, ko04320, ko04350, ko04360, ko04370, ko04371, ko04380, ko04510, ko04520, ko04540, ko04550, ko04611, ko04620, ko04621, ko04650, ko04657, ko04658, ko04659, ko04660, ko04662, ko04664, ko04666, ko04668, ko04713, ko04720, ko04722, ko04723, ko04724, ko04725, ko04726, ko04730, ko04810, ko04910, ko04912, ko04914, ko04915, ko04916, ko04917, ko04919, ko04921, ko04926, ko04930, ko04933, ko04934, ko04960, ko05010, ko05020, ko05034, ko05131, ko05132, ko05133, ko05140, ko05142, ko05145, ko05152, ko05160, ko05161, ko05164, ko05165, ko05167, ko05200, ko05203, ko05205, ko05210, ko05211, ko05212, ko05213, ko05214, ko05215, ko05216, ko05218, ko05219, ko05220, ko05221, ko05223, ko05224, ko05225, ko05226, ko05230, ko05231, map01521, map01522, map01524, map04010, map04011, map04012, map04013, map04014, map04015, map04022, map04024, map04062, map04066, map04068, map04071, map04072, map04114, map04140, map04150, map04151, map04210, map04214, map04218, map04261, map04270, map04320, map04350, map04360, map04370, map04371, map04380, map04510, map04520, map04540, map04550, map04611, map04620, map04621, map04650, map04657, map04658, map04659, map04660, map04662, map04664, map04666, map04668, map04713, map04720, map04722, map04723, map04724, map04725, map04726, map04730, map04810, map04910, map04912, map04914, map04915, map04916, map04917, map04919, map04921, map04926, map04930, map04933, map04934, map04960, map05010, map05020, map05034, map05131, map05132, map05133, map05140, map05142, map05145, map05152, map05160, map05161, map05164, map05165, map05167, map05200, map05203, map05205, map05210,
FUN_006041	M protein, serotype 2.1	-	-	-	-
FUN_006042	GAF domain	CHK1	2.7.13.3	ko:K19690	ko02020, map02020
FUN_006043	DIL	-	-	-	-
FUN_006044	Allantoicase repeat	DAL2	3.5.3.4, 4.3.2.3	ko:K01477, ko:K01483	ko00230, ko01100, ko01120, map00230, map01100, map01120
FUN_006045	Belongs to the cytochrome b5 family	-	-	-	-
FUN_006047	Ndr family	-	-	-	-
FUN_006048	D-xylulose 5-phosphate/D-fructose 6-phosphate phosphoketolase	-	4.1.2.22, 4.1.2.9	ko:K01621	ko00030, ko00710, ko01100, ko01120, map00030, map00710, map01100, map01120
FUN_006049	Methyltransferase domain	-	-	-	-
FUN_006050	duf1630-domain-containing protein	-	-	-	-
FUN_006051	Vacuolar 14 Fab1-binding region	-	-	ko:K10875, ko:K15305	ko03440, ko05166, ko05203, map03440, map05166, map05203
FUN_006052	vacuolar H -ATPase assembly protein	-	-	-	-

FUN_006053	Belongs to the FPP GGPP synthase family	COQ1	2.5.1.82, 2.5.1.83	ko:K05355	ko00900, ko01110, map00900, map01110
FUN_006054	WD domain, G-beta repeat	SWD3	-	ko:K14963	ko04934, map04934
FUN_006055	Endonuclease/Exonuclease/phosphatase family	ISC1	3.1.4.12	ko:K12351	ko00600, ko01100, ko04071, map00600, map01100, map04071
FUN_006056	Rad54 N terminal	RAD54	-	ko:K10875	ko03440, map03440
FUN_006057	Beta-glucanases participate in the metabolism of beta- glucan, the main structural component of the cell wall. Acts on lutean, pustulan and 1, 6-oligo-beta-D-glucosides	exgB	3.2.1.75	ko:K22277	-
FUN_006059	Belongs to the phosphohexose mutase family	-	5.4.2.2, 5.4.2.7	ko:K01835, ko:K15779	ko00010, ko00030, ko00052, ko00230, ko00500, ko00520, ko00521, ko01100, ko01110, ko01120, ko01130, map00010, map00030, map00052, map00230, map00500, map00520, map00521, map01100, map01110, map01120, map01130
FUN_006060	Fatty acid desaturase	SLD	1.14.19.18	ko:K13076	-
FUN_006061	Putative zinc finger in N-recognin (UBR box)	-	2.3.2.27	ko:K11979, ko:K16261	-
FUN_006062	Belongs to the MCM family	MCM2	3.6.4.12	ko:K02540	ko03030, ko04110, ko04111, ko04113, map03030, map04110, map04111, map04113
FUN_006063	Gti1/Pac2 family	PTH2	-	ko:K21631	-
FUN_006064	Major Facilitator Superfamily	DAL5	-	ko:K08192	-
FUN_006065	Chitin synthesis regulation, resistance to Congo red	-	-	-	-
FUN_006066	Belongs to the small GTPase superfamily. Arf family	-	-	ko:K07955	-
FUN_006067	DNA-dependent RNA polymerase catalyzes the transcription of DNA into RNA using the four ribonucleoside triphosphates as substrates	RPA135	2.7.7.6	ko:K03002	ko00230, ko00240, ko01100, ko03020, map00230, map00240, map01100, map03020
FUN_006068	Pyridoxamine 5'-phosphate oxidase like	-	-	-	-
FUN_006069	Nuclear segregation protein	BFR1	-	-	-
FUN_006070	Uroporphyrinogen-III synthase HemD	-	4.2.1.75	ko:K01719	ko00860, ko01100, ko01110, ko01120, map00860, map01100, map01110, map01120
FUN_006071	Kinetochore protein Mis14 like	-	-	ko:K11561	-
FUN_006072	RasGAP C-terminus	gap1	-	ko:K05767	ko04810, map04810
FUN_006074	Pyridine nucleotide-disulphide oxidoreductase	-	1.6.99.3	ko:K03885	ko00190, map00190
FUN_006075	Belongs to the arginase family	CAR1	3.5.3.1	ko:K01476	ko00220, ko00330, ko01100, ko01110, ko01130, ko01230, ko05146, map00220, map00330, map01100, map01110, map01130, map01230, map05146
FUN_006077	Frequency clock protein	-	-	-	-
FUN_006078	COQ9	COQ9	-	ko:K18587	-
FUN_006079	Coiled-coil domain-containing protein 94 homolog	-	-	-	-
FUN_006081	Putative zinc finger in N-recognin, a recognition component of the N-end rule pathway	UBR1	2.3.2.27	ko:K10625	-
FUN_006082	Mago binding	-	-	ko:K14294	ko03013, ko03015, map03013, map03015
FUN_006084	SPFH domain / Band 7 family	-	-	ko:K07192	ko04910, map04910
FUN_006085	A domain family that is part of the cupin metalloenzyme superfamily.	JHD1	1.14.11.27	ko:K10276	-
FUN_006086	RNA recognition motif	-	-	ko:K12833	ko03040, map03040
FUN_006087	RED-like protein N-terminal region	-	-	-	-
FUN_006088	Proteasome subunit beta type	PRE2	3.4.25.1	ko:K02737	ko03050, map03050
FUN_006089	Exonuclease V - a 5' deoxyribonuclease	-	-	ko:K17815	-
FUN_006090	Histone H3	HHT1	-	ko:K11253	ko05034, ko05202, ko05322, map05034, map05202, map05322

FUN_006091	Core component of nucleosome. Nucleosomes wrap and compact DNA into chromatin, limiting DNA accessibility to the cellular machineries which require DNA as a template. Histones thereby play a central role in transcription regulation, DNA repair, DNA replication and chromosomal stability. DNA accessibility is regulated via a complex set of post-translational modifications of histones, also called histone code, and nucleosome remodeling	HHF1	-	ko:K11254	ko05034, ko05203, ko05322, map05034, map05203, map05322
FUN_006092	Haloacid dehalogenase, type II	-	3.8.1.2	ko:K01560	ko00361, ko00625, ko01100, ko01120, map00361, map00625, map01100, map01120
FUN_006093	cytidine deoxycytidylate deaminase family protein	-	-	-	-
FUN_006094	Ras family, other	ras2	-	ko:K07827	ko01521, ko01522, ko04010, ko04012, ko04013, ko04014, ko04015, ko04062, ko04068, ko04071, ko04072, ko04137, ko04138, ko04140, ko04150, ko04151, ko04210, ko04211, ko04213, ko04214, ko04218, ko04320, ko04360, ko04370, ko04371, ko04540, ko04550, ko04650, ko04660, ko04662, ko04664, ko04714, ko04720, ko04722, ko04725, ko04726, ko04730, ko04810, ko04910, ko04912, ko04914, ko04915, ko04916, ko04917, ko04919, ko04921, ko04926, ko04933, ko04960, ko05034, ko05160, ko05161, ko05165, ko05166, ko05167, ko05200, ko05203, ko05205, ko05206, ko05210, ko05211, ko05212, ko05213, ko05214, ko05215, ko05216, ko05218, ko05219, ko05220, ko05221, ko05223, ko05224, ko05225, ko05226, ko05230, ko05231, map01521, map01522, map04010, map04012, map04013, map04014, map04015, map04062, map04068, map04071, map04072, map04137, map04138, map04140, map04150, map04151, map04210, map04211, map04213, map04214, map04218, map04320, map04360, map04370, map04371, map04540, map04550, map04650, map04660, map04662, map04664, map04714, map04720, map04722, map04725, map04726, map04730, map04810, map04910, map04912, map04914, map04915, map04916, map04917, map04919, map04921, map04926, map04933, map04960, map05034, map05160, map05161, map05165, map05166, map05167, map05200, map05203, map05205, map05206, map05210, map05211, map05212, map05213, map05214, map05215, map05216, map05218, map05219, map05220, map05221, map05223, map05224, map05225, map05226, map05230, map05231
FUN_006095	Acetyl-coenzyme A synthetase N-terminus	-	6.2.1.17	ko:K01908	ko00640, ko01100, map00640, map01100
FUN_006096	PKS_DH	-	-	-	-
FUN_006097	Steryl acetyl hydrolase	-	-	-	-
FUN_006099	Altered inheritance of mitochondria protein 21	-	-	-	-
FUN_006100	MafB19-like deaminase	TAD2	-	ko:K15441	-
FUN_006101	Heterokaryon incompatibility protein (HET)	-	-	-	-
FUN_006104	Per1-like family	-	-	-	-
FUN_006105	Serine/Threonine protein kinases, catalytic domain	NPKA	2.7.11.22	ko:K08818	-

FUN_006106	Arginine methyltransferase involved in the assembly or stability of mitochondrial NADH ubiquinone oxidoreductase complex (complex I)	-	1.3.3.15, 1.3.3.4	ko:K00231, ko:K18164	ko00860, ko01100, ko01110, ko04714, map00860, map01100, map01110, map04714
FUN_006107	Binds to actin and affects the structure of the cytoskeleton. At high concentrations, profilin prevents the polymerization of actin, whereas it enhances it at low concentrations	PFY1	-	ko:K05759	ko04013, ko04015, ko04810, ko05131, ko05132, map04013, map04015, map04810, map05131, map05132
FUN_006108	Prion-inhibition and propagation	-	-	-	-
FUN_006109	Calcineurin-like phosphoesterase	-	-	-	-
FUN_006110	CBM18-CE4-CBM18-CBM18. carbohydrate-binding module family 18	-	3.5.1.41	ko:K01452	ko00520, ko01100, map00520, map01100
FUN_006111	Phosphoric diester hydrolase activity. It is involved in the biological process described with lipid metabolic process	-	-	-	-
FUN_006112	WD40 repeats	NAN1	-	ko:K14552	ko03008, map03008
FUN_006113	Mitochondrial import receptor subunit Tom6, fungal	-	-	-	-
FUN_006114	PB1 domain	-	-	-	-
FUN_006115	Copper/zinc superoxide dismutase (SODC)	CCS1	-	ko:K04569, ko:K07213	ko04978, ko05014, map04978, map05014
FUN_006116	Ribosomal protein L13	-	-	ko:K02871	ko03010, map03010
FUN_006117	Catalyzes the oxidation of uric acid to 5- hydroxyisourate, which is further processed to form (S)-allantoin	-	1.7.3.3	ko:K00365	ko00230, ko00232, ko01100, ko01120, map00230, map00232, map01100, map01120
FUN_006118	Rapamycin-insensitive companion of mTOR, domain 5	-	-	ko:K08267	ko04150, map04150
FUN_006119	ATP-dependent protease La (LON) substrate-binding domain	-	-	-	-
FUN_006120	Major Facilitator Superfamily	-	-	-	-
FUN_006121	GDP-mannose 4, 6 dehydratase	GRP2	1.1.1.283	ko:K17741	ko00620, ko00640, ko04011, map00620, map00640, map04011
FUN_006122	Nuclear transport factor 2 (NTF2) domain	NTF2	-	-	-
FUN_006123	Belongs to the thiolase family	-	2.3.1.16	ko:K07513	ko00071, ko00280, ko00592, ko01040, ko01100, ko01110, ko01130, ko01212, ko03320, ko04146, map00071, map00280, map00592, map01040, map01100, map01110, map01130, map01212, map03320, map04146
FUN_006124	Sugar (and other) transporter	-	-	-	-
FUN_006125	Belongs to the AAA ATPase family	-	-	ko:K03066	ko03050, ko05169, map03050, map05169
FUN_006126	Belongs to the Ca(2) cation antiporter (CaCA) (TC 2.A.19) family	-	-	ko:K07300	-
FUN_006127	RING-like zinc finger	-	-	-	-
FUN_006128	short chain dehydrogenase	-	-	-	-
FUN_006131	Protein of unknown function (DUF3292)	-	-	-	-
FUN_006132	Rab subfamily of small GTPases	SEC4	-	ko:K07901, ko:K18158	ko04144, ko04152, ko04530, ko04972, map04144, map04152, map04530, map04972
FUN_006133	ATP synthase regulation protein NCA2	-	-	ko:K18158	-
FUN_006134	CFEM domain	-	-	-	-
FUN_006135	Cobalamin-independent synthase, Catalytic domain	-	-	-	-
FUN_006136	Nup93/Nic96	NIC96	-	ko:K14309	ko03013, map03013
FUN_006137	Mediator complex subunit 27	-	-	-	-
FUN_006138	MaoC like domain	-	-	-	-
FUN_006139	Protein kinase C terminal domain	SCH9	2.7.11.1	ko:K08286, ko:K19800	ko04138, ko04213, map04138, map04213

FUN_006140	Histone-like transcription factor (CBF/NF-Y) and archaeal histone	NCB2	-	ko:K21751	-
FUN_006141	Belongs to the SNF7 family	-	-	ko:K12197, ko:K16230	ko04144, ko04217, map04144, map04217
FUN_006142	Belongs to the peptidase M28 family	-	2.3.2.5	ko:K00683	-
FUN_006144	This enzyme condenses acetyl-CoA with acetoacetyl-CoA to form HMG-CoA, which is the substrate for HMG-CoA reductase	ERG13	2.3.3.10	ko:K01641	ko00072, ko00280, ko00650, ko00900, ko01100, ko01110, ko01130, map00072, map00280, map00650, map00900, map01100, map01110, map01130
FUN_006145	Cytochrome c oxidase assembly protein COX16	COX16	-	ko:K18182	ko04714, map04714
FUN_006146	Domain in UVSB PI-3 kinase, MEI-41 and ESR-1	MEC1	2.7.11.1	ko:K02543, ko:K06640	ko03460, ko04110, ko04111, ko04113, ko04115, ko04214, ko04218, ko05165, ko05166, map03460, map04110, map04111, map04113, map04115, map04214, map04218, map05165, map05166
FUN_006148	Cytochrome P450	-	-	-	-
FUN_006149	Putative cyclase	-	-	-	-
FUN_006150	Allows the formation of correctly charged Gln-tRNA(Gln) through the transamidation of misacylated Glu-tRNA(Gln) in the mitochondria. The reaction takes place in the presence of glutamine and ATP through an activated gamma-phospho-Glu- tRNA(Gln)	HER2	6.3.5.6, 6.3.5.7	ko:K02433	ko00970, ko01100, map00970, map01100
FUN_006151	Integral peroxisomal membrane peroxin	-	-	-	-
FUN_006152	Pam16	-	-	ko:K17805	-
FUN_006153	Cid1 family poly A polymerase	-	2.7.7.19	ko:K03514	ko03018, map03018
FUN_006154	Calcium-activated chloride channel	-	-	ko:K19327	-
FUN_006155	alpha/beta hydrolase fold	-	-	-	-
FUN_006156	Belongs to the actin family	ARP6	-	ko:K11662	-
FUN_006157	Ribosomal subunit 39S	-	-	-	-
FUN_006158	Carboxylesterase family	-	-	-	-
FUN_006160	Pre-mRNA-splicing factor of RES complex	CWC26	-	ko:K13106	-
FUN_006163	AAA domain (dynein-related subfamily)	AFG2	-	ko:K14575	ko03008, map03008
FUN_006165	Possible membrane-associated motif in LPS-induced tumor necrosis factor alpha factor (LITAF), also known as PIG7, and other animal proteins.	-	-	ko:K19363	ko04142, map04142
FUN_006166	AAR2 protein	-	-	ko:K13205	-
FUN_006167	Component of the endosomal sorting complex required for transport II (ESCRT-II), which is required for multivesicular body (MVB) formation and sorting of endosomal cargo proteins into MVBs	dot2	-	ko:K12188	ko04144, map04144
FUN_006168	Acid phosphatase homologues	-	3.1.3.4, 3.1.3.81	ko:K18693	ko00561, ko00564, ko01110, map00561, map00564, map01110
FUN_006169	Component of the eukaryotic translation initiation factor 3 (eIF-3) complex, which is involved in protein synthesis of a specialized repertoire of mRNAs and, together with other initiation factors, stimulates binding of mRNA and methionyl-tRNAi to the 40S ribosome. The eIF-3 complex specifically targets and initiates translation of a subset of mRNAs involved in cell proliferation	NIP1	-	ko:K03252	ko03013, map03013
FUN_006170	Serine/Threonine protein kinases, catalytic domain	YAK1	2.7.12.1	ko:K18670	-
FUN_006171	L-lysine 6-monooxygenase (NADPH-requiring)	-	1.14.13.195, 1.14.13.196	ko:K10531	-

FUN_006172	Condensation domain	-	-	ko:K22148	-
FUN_006173	Fungal trichothecene efflux pump (TRI12)	-	-	-	-
FUN_006175	Glucosidase II beta subunit-like protein	-	-	ko:K08288	ko04141, map04141
FUN_006177	Grap2 and cyclin-D-interacting	-	-	-	-
FUN_006180	Esterase-like activity of phytase	-	-	-	-
FUN_006183	Reversible hydration of carbon dioxide	-	-	-	-
FUN_006184	Domain of unknown function (DUF3328)	-	-	-	-
FUN_006185	Belongs to the cytochrome P450 family	-	-	-	-
FUN_006188	Aldo/keto reductase family	-	-	-	-
FUN_006190	PrpF protein	-	-	-	-
FUN_006191	Fungal specific transcription factor domain	-	-	-	-
FUN_006192	Belongs to the mitochondrial carrier (TC 2.A.29) family	CTP1	-	ko:K15100	-
FUN_006194	Uncharacterised MFS-type transporter YbfB	-	-	-	-
FUN_006195	Di-sulfide bridge nucleocytoplasmic transport domain	-	-	-	-
FUN_006196	Domain of unknown function (DUF1729)	FAS1	2.3.1.86	ko:K00668	ko00061, ko01100, ko01212, map00061, map01100, map01212
FUN_006197	Belongs to the beta-ketoacyl-ACP synthases family	FAS1	2.3.1.86	ko:K00667, ko:K00668	ko00061, ko01100, ko01212, map00061, map01100, map01212
FUN_006198	Amidohydrolase family	-	3.5.2.2	ko:K01464	ko00240, ko00410, ko00770, ko00983, ko01100, map00240, map00410, map00770, map00983, map01100
FUN_006203	Paf1	-	-	ko:K15174	ko04011, map04011
FUN_006204	Probably involved in transcription regulation via its interaction with the INO80 complex, a chromatin remodeling complex	ARP8	-	ko:K11673	-
FUN_006205	Fungal specific transcription factor domain	DAL81	-	-	-
FUN_006206	Permease for cytosine/purines, uracil, thiamine, allantoin	-	-	ko:K03457	-
FUN_006207	Isochorismatase family	-	-	-	-
FUN_006208	OPT oligopeptide transporter protein	-	-	-	-
FUN_006209	Belongs to the AAA ATPase family	BCS1	-	ko:K08900	-
FUN_006210	RNA recognition motif containing protein	-	-	-	-
FUN_006211	EthD domain	-	-	-	-
FUN_006212	Carbon-nitrogen hydrolase	-	-	-	-
FUN_006213	EthD domain	-	-	-	-
FUN_006214	component of the eukaryotic translation initiation factor 3 (eIF-3) complex, which is involved in protein synthesis of a specialized repertoire of mRNAs and, together with other initiation factors, stimulates binding of mRNA and methionyl-tRNAi to the 40S ribosome. The eIF-3 complex specifically targets and initiates translation of a subset of mRNAs involved in cell proliferation	-	-	ko:K15029	-
FUN_006217	Cytochrome oxidase c assembly	-	-	-	-
FUN_006218	Protein of unknown function (DUF2458)	-	-	-	-
FUN_006221	Protein of unknown function (DUF3176)	-	-	-	-
FUN_006222	Tripeptidyl-peptidase	-	3.4.14.9	ko:K01279	ko04142, map04142
FUN_006223	Belongs to the oxygen-dependent FAD-linked oxidoreductase family	easE	-	-	-
FUN_006224	ATP adenylyltransferase	-	2.7.7.53	ko:K00988	ko00230, map00230

FUN_006225	Kelch motif	-	-	-	-
FUN_006226	ABC1 family	-	-	ko:K08869	-
FUN_006227	Pyridine nucleotide-disulphide oxidoreductase	-	-	-	-
FUN_006230	Succinyl-CoA synthetase functions in the citric acid cycle (TCA), coupling the hydrolysis of succinyl-CoA to the synthesis of ATP and thus represents the only step of substrate- level phosphorylation in the TCA. The beta subunit provides nucleotide specificity of the enzyme and binds the substrate succinate, while the binding sites for coenzyme A and phosphate are found in the alpha subunit	LSC2	6.2.1.4, 6.2.1.5	ko:K01900	ko00020, ko00640, ko01100, ko01110, ko01120, ko01130, ko01200, map00020, map00640, map01100, map01110, map01120, map01130, map01200
FUN_006231	Protein of unknown function (DUF3245)	-	-	-	-
FUN_006232	Calcineurin-like phosphoesterase	-	-	-	-
FUN_006233	Regulator of chromosome condensation (RCC1) repeat	-	-	ko:K11493	-
FUN_006235	Chromatin remodelling complex Rsc7/Swp82 subunit	NPL6	-	ko:K11761	-
FUN_006237	FAD dependent oxidoreductase	-	-	-	-
FUN_006238	basic region leucin zipper	HAC1	-	ko:K16230	-
FUN_006239	RimK-like ATP-grasp domain	VIP1	-	-	-
FUN_006240	Utp21 specific WD40 associated putative domain	UTP21	-	ko:K14554	ko03008, map03008
FUN_006244	Belongs to the TRAFAC class myosin-kinesin ATPase superfamily. Kinesin family	-	2.8.3.13	ko:K10402, ko:K17387, ko:K18703	ko05206, map05206
FUN_006245	Belongs to the class I-like SAM-binding methyltransferase superfamily. Protein arginine N- methyltransferase family	HMT1	2.1.1.319	ko:K11434	ko04068, ko04922, map04068, map04922
FUN_006247	Lumazine binding domain	RIB5	2.5.1.9	ko:K00793	ko00740, ko01100, ko01110, map00740, map01100, map01110
FUN_006248	Belongs to the TRAFAC class myosin-kinesin ATPase superfamily. Kinesin family	-	2.8.3.13	ko:K10402, ko:K17387, ko:K18703	ko05206, map05206
FUN_006249	PHD-finger	-	-	-	-
FUN_006250	Brf1-like TBP-binding domain	BRF1	-	ko:K15196	-
FUN_006252	peroxidase	-	1.11.1.13	ko:K20205	-
FUN_006253	Belongs to the cyclin family	-	-	ko:K15563	-
FUN_006254	alpha/beta hydrolase fold	-	-	-	-
FUN_006255	Putative adipose-regulatory protein (Seipin)	-	-	ko:K19365	-
FUN_006258	Poly(ADP-ribose) polymerase and DNA-Ligase Zn-finger region	-	-	-	-
FUN_006261	zinc finger	-	-	-	-
FUN_006264	NAD P-binding protein	-	-	-	-
FUN_006266	Plays a role in vesicular protein sorting	vps35	-	ko:K18468	ko04144, map04144
FUN_006268	Acyl CoA binding protein	-	-	-	-
FUN_006269	Four repeated domains in the Fasciclin I family of proteins, present in many other contexts.	-	-	-	-
FUN_006270	Shikimate dehydrogenase substrate binding domain	-	-	-	-
FUN_006271	Xylose isomerase-like TIM barrel	-	1.13.11.27	ko:K00457	ko00130, ko00350, ko00360, ko01100, map00130, map00350, map00360, map01100
FUN_006272	Belongs to the major facilitator superfamily. Sugar transporter (TC 2.A.1.1) family	qutD	-	-	-
FUN_006273	Shikimate dehydrogenase substrate binding domain	-	1.1.1.24	ko:K09484	ko00400, map00400

FUN_006274	Inositol-1-monophosphatase	-	3.1.3.25	ko:K01092	ko00521, ko00562, ko01100, ko04070, map00521, map00562, map01100, map04070
FUN_006276	Ubiquitin fusion degradation protein UFD1	-	-	-	-
FUN_006278	Links covalently the heme group to the apoprotein of cytochrome c	CYC3	4.4.1.17	ko:K01764	ko00860, map00860
FUN_006279	Fumble	-	2.7.1.33	ko:K09680	ko00770, ko01100, map00770, map01100
FUN_006280	Myb DNA-binding domain protein	-	-	-	-
FUN_006281	Uncharacterized protein family UPF0016	GDT1	-	-	-
FUN_006282	Methyltransferase domain	-	-	-	-
FUN_006283	Cytochrome P450	-	-	-	-
FUN_006284	transcription, DNA-templated	RBF1	-	-	-
FUN_006285	Catalyzes the phosphorylation of D-fructose 6-phosphate to fructose 1, 6-bisphosphate by ATP, the first committing step of glycolysis	PFK1	2.7.1.11	ko:K00850	ko00010, ko00030, ko00051, ko00052, ko00680, ko01100, ko01110, ko01120, ko01130, ko01200, ko01230, ko03018, ko04152, ko05230, map00010, map00030, map00051, map00052, map00680, map01100, map01110, map01120, map01130, map01200, map01230, map03018, map04152, map05230
FUN_006286	Zinc-binding dehydrogenase	ETR1	1.3.1.38	ko:K07512	ko00062, ko01100, ko01212, map00062, map01100, map01212
FUN_006287	Major royal jelly protein	-	-	-	-
FUN_006288	Subunit 17 of Mediator complex	SRB4	-	ko:K15134	-
FUN_006289	IMP-specific 5'-nucleotidase involved in IMP (inositol monophosphate) degradation	ISN1	3.1.3.99	ko:K18550	ko00230, ko00760, ko01100, map00230, map00760, map01100
FUN_006291	HEC/Ndc80p family	NDC80	-	ko:K11547	-
FUN_006292	Extension to Ser/Thr-type protein kinases	-	2.7.11.1	ko:K22008	-
FUN_006294	Heterokaryon incompatibility protein (HET)	-	-	-	-
FUN_006295	Glycoside hydrolase family 3 protein	-	3.2.1.37	ko:K15920	ko00520, ko01100, map00520, map01100
FUN_006296	Carbohydrate esterase family 1 protein	-	-	-	-
FUN_006297	DNA-directed RNA polymerase III subunit Rpc31	RPC31	-	ko:K03024	ko00230, ko00240, ko01100, ko03020, ko04623, ko05169, map00230, map00240, map01100, map03020, map04623, map05169
FUN_006299	Thioesterase-like superfamily	-	-	-	-
FUN_006301	Belongs to the sterol desaturase family	ERG25	1.14.18.9	ko:K07750	ko00100, ko01100, ko01130, map00100, map01100, map01130
FUN_006302	bZIP transcription factor	kapC	-	ko:K09043	-
FUN_006303	Voltage-dependent anion channel	-	-	-	-

FUN_006304	Rapamycin binding domain	TOR1	2.7.11.1	ko:K07203	ko01521, ko01522, ko04012, ko04066, ko04072, ko04136, ko04138, ko04139, ko04140, ko04150, ko04151, ko04152, ko04211, ko04212, ko04213, ko04218, ko04371, ko04630, ko04659, ko04714, ko04910, ko04919, ko04920, ko04930, ko04931, ko05165, ko05167, ko05200, ko05205, ko05206, ko05210, ko05212, ko05214, ko05215, ko05221, ko05224, ko05225, ko05226, ko05230, ko05231, map01521, map01522, map04012, map04066, map04072, map04136, map04138, map04139, map04140, map04150, map04151, map04152, map04211, map04212, map04213, map04218, map04371, map04630, map04659, map04714, map04910, map04919, map04920, map04930, map04931, map05165, map05167, map05200, map05205, map05206, map05210, map05212, map05214, map05215, map05221, map05224, map05225, map05226, map05230, map05231
FUN_006306	FAD-binding domain	-	-	-	-
FUN_006308	Eukaryotic translation initiation factor 4G1	-	-	ko:K03260	ko03013, ko05416, map03013, map05416
FUN_006309	THO complex subunit 1 transcription elongation factor	-	-	ko:K12878	ko03013, ko03040, map03013, map03040
FUN_006310	Replication Fork Protection Component Swi3	CSM3	-	ko:K10998	-
FUN_006311	DSHCT	SKI2	-	ko:K12599	ko03018, map03018
FUN_006312	Cse1	NMD5	-	ko:K20223	ko04013, map04013
FUN_006313	Enoyl-CoA hydratase/isomerase	-	4.2.1.17	ko:K07511	ko00062, ko00071, ko00280, ko00310, ko00380, ko00410, ko00627, ko00640, ko00650, ko00930, ko01100, ko01110, ko01120, ko01130, ko01200, ko01212, map00062, map00071, map00280, map00310, map00380, map00410, map00627, map00640, map00650, map00930, map01100, map01110, map01120, map01130, map01200, map01212
FUN_006314	Involved in the retrieval of endoplasmic reticulum membrane proteins from the early Golgi compartment	-	-	-	-
FUN_006315	Taurine catabolism dioxygenase TauD, TfdA family	-	1.14.11.8	ko:K00474	ko00310, map00310
FUN_006317	Belongs to the AAA ATPase family	RPT5	-	ko:K03065	ko03050, ko05169, map03050, map05169
FUN_006318	Protein of unknown function (DUF3419)	-	-	ko:K13621	ko00564, map00564
FUN_006319	Intron-binding protein aquarius N-terminus	-	-	ko:K12874	ko03040, map03040
FUN_006320	Component of the core-TFIIH basal transcription factor involved in nucleotide excision repair (NER) of DNA	TFB2	-	ko:K03144	ko03022, ko03420, ko05203, map03022, map03420, map05203
FUN_006321	Thioesterase superfamily	-	-	ko:K17362	-
FUN_006323	Timeless protein C terminal region	TOF1	-	ko:K10997	-
FUN_006324	3-hydroxyacyl-CoA dehydrogenase, C-terminal domain	-	1.1.1.157	ko:K00074	ko00360, ko00362, ko00650, ko01100, ko01120, map00360, map00362, map00650, map01100, map01120
FUN_006329	Cyclin domain-containing protein	-	-	-	-
FUN_006332	Cation efflux family	zrg17	-	-	-
FUN_006333	IBR domain, a half RING-finger domain	-	-	-	-
FUN_006336	Putative stress-responsive nuclear envelope protein	-	-	-	-
FUN_006338	Heterokaryon incompatibility protein (HET)	-	-	-	-
FUN_006341	Domain of unknown function (DUF4291)	-	-	-	-

FUN_006342	Protein of unknown function (DUF3433)	-	-	-	-
FUN_006343	Belongs to the small GTPase superfamily. Arf family	ARL1	-	ko:K07942	-
FUN_006345	O-methyltransferase	-	2.1.1.109	ko:K17649	ko00254, map00254
FUN_006347	Belongs to the oxygen-dependent FAD-linked oxidoreductase family	-	-	-	-
FUN_006350	Major Facilitator Superfamily	-	-	-	-
FUN_006351	Pyridine nucleotide-disulphide oxidoreductase, dimerisation domain	-	-	-	-
FUN_006352	Exonuclease	rex2	-	ko:K13288	ko03008, map03008
FUN_006353	DNA polymerase	POL1	2.7.7.7	ko:K02320	ko00230, ko00240, ko01100, ko03030, map00230, map00240, map01100, map03030
FUN_006354	Catalyzes the cleavage of L-kynurenine (L-Kyn) and L-3- hydroxykynurenine (L-3OHKyn) into anthranilic acid (AA) and 3- hydroxyanthranilic acid (3-OHAA),	BNA5	3.7.1.3	ko:K01556	ko00380, ko01100, map00380, map01100
FUN_006355	Iron-sulphur cluster biosynthesis	ISA2	-	ko:K22072	-
FUN_006356	3-beta hydroxysteroid dehydrogenase/isomerase family	-	-	-	-
FUN_006359	Putative serine esterase (DUF676)	-	-	-	-
FUN_006361	Has a role in a nucleosome assembly pathway that is required for the integrity of heterochromatin and proper chromosome segregation	HIR3	-	-	-
FUN_006362	ChAPs (Chs5p-Arf1p-binding proteins)	BUD7	-	ko:K19881	-
FUN_006363	Belongs to the universal ribosomal protein uS9 family	RPS16	-	ko:K02960	ko03010, map03010
FUN_006364	Regulator of chromosome condensation (RCC1) repeat	ATS1	-	ko:K15462	-
FUN_006365	Rad9	-	3.1.11.2	ko:K10994	ko04218, map04218
FUN_006367	Thiamin biosynthesis protein (Thi-4)	THI20	2.7.1.49, 2.7.4.7, 3.5.99.2	ko:K00877	ko00730, ko01100, map00730, map01100
FUN_006368	Belongs to the DEAD box helicase family	DBP2	3.6.4.13	ko:K12823	ko03040, ko05202, ko05205, map03040, map05202, map05205
FUN_006369	zinc finger	-	-	ko:K21455	-
FUN_006370	PPR repeat family	-	-	-	-
FUN_006371	Alcohol dehydrogenase GroES-like domain	-	-	-	-
FUN_006373	Carbohydrate esterase family 12 protein	-	3.1.1.86	ko:K15530	-
FUN_006374	Rtf2 RING-finger	rtf2	-	-	-
FUN_006375	Protein of unknown function (DUF1765)	-	-	-	-
FUN_006376	ATP synthase complex subunit h	ATP14	-	ko:K02141	ko00190, ko01100, map00190, map01100
FUN_006377	TFIIB zinc-binding	SUA7	-	ko:K03124	ko03022, ko05169, ko05203, map03022, map05169, map05203
FUN_006381	Aldo/keto reductase family	-	-	-	-
FUN_006382	Haloacid dehalogenase-like hydrolase	-	-	-	-
FUN_006383	CUE domain	DEF1	-	-	-
FUN_006385	Serine/Threonine protein kinases, catalytic domain	PRP4	2.7.11.1	ko:K08827	-
FUN_006386	Domain of unknown function (DUF3425)	-	-	-	-
FUN_006387	Conserved proline-rich protein	-	-	-	-
FUN_006388	Domain of unknown function (DUF384)	HGH1	-	-	-
FUN_006389	Major Facilitator Superfamily	-	-	-	-
FUN_006390	transcription initiation factor IIF subunit alpha	TFG1	-	ko:K03138	ko03022, map03022
FUN_006391	Cid1 family poly A polymerase	-	2.7.7.52	ko:K13291	-

FUN_006392	Belongs to the major facilitator superfamily. Sugar transporter (TC 2.A.1.1) family	HGT1	-	-	-
FUN_006393	Belongs to the ubiquitin-conjugating enzyme family	UBC6	2.3.2.23	ko:K04554	ko04120, ko04141, ko05012, map04120, map04141, map05012
FUN_006394	Surface antigen	-	-	ko:K07277	-
FUN_006396	Belongs to the cytochrome b5 family	DAP1	-	ko:K17278	-
FUN_006398	Catechol dioxygenase N terminus	-	-	-	-
FUN_006399	Protein of unknown function (DUF3445)	-	-	-	-
FUN_006402	Late exocytosis, associated with Golgi transport	-	-	ko:K21989	-
FUN_006403	Catalyzes the conversion of GlcNAc-6-P into GlcNAc-1-P during the synthesis of uridine diphosphate UDP-GlcNAc, which is a biosynthetic precursor of chitin and also supplies the amino sugars for N-linked oligosaccharides of glycoproteins	PCM1	5.4.2.3	ko:K01836	ko00520, ko01100, ko01130, map00520, map01100, map01130
FUN_006404	RING finger domain protein	-	-	ko:K17421	-
FUN_006405	Belongs to the small GTPase superfamily. Rho family	RHO2	-	ko:K07975	-
FUN_006406	May act as a negative regulator of salt tolerance	NST1	-	ko:K14798	-
FUN_006407	ATPase family associated with various cellular activities (AAA)	-	-	-	-
FUN_006408	Mitochondrial genome maintenance MGM101	MGM101	-	-	-
FUN_006409	Belongs to the cytochrome P450 family	-	-	-	-
FUN_006410	Biotin-protein ligase, N terminal	BPL1	6.3.4.10, 6.3.4.11, 6.3.4.15, 6.3.4.9	ko:K01942	ko00780, ko01100, map00780, map01100
FUN_006411	Belongs to the OSBP family	kes1	-	ko:K20465	-
FUN_006412	ABC transporter	-	1.14.13.1	ko:K00480	ko00621, ko00624, ko00626, ko01100, ko01120, ko01220, map00621, map00624, map00626, map01100, map01120, map01220
FUN_006413	Belongs to the phosphohexose mutase family	PGM2	5.4.2.2	ko:K01835	ko00010, ko00030, ko00052, ko00230, ko00500, ko00520, ko00521, ko01100, ko01110, ko01120, ko01130, map00010, map00030, map00052, map00230, map00500, map00520, map00521, map01100, map01110, map01120, map01130
FUN_006416	Diacylglycerol kinase catalytic domain	-	-	-	-
FUN_006417	Domain of unknown function (DUF4078)	-	-	-	-
FUN_006418	homoserine dehydrogenase	HOM6	1.1.1.3	ko:K00003	ko00260, ko00270, ko00300, ko01100, ko01110, ko01120, ko01130, ko01230, map00260, map00270, map00300, map01100, map01110, map01120, map01130, map01230
FUN_006419	Carbohydrate esterase family 8 protein	-	3.1.1.11	ko:K01051	ko00040, ko01100, map00040, map01100
FUN_006421	Polysaccharide biosynthesis protein	-	-	-	-
FUN_006423	Autophagy-related protein 11	ATG11	-	ko:K08330	ko04136, ko04138, ko04139, map04136, map04138, map04139
FUN_006424	Centromere protein H (CENP-H)	-	-	-	-
FUN_006425	HMG-box domain	NHP6	-	-	-
FUN_006426	Catalytic component of the signal peptidase complex (SPC), which catalyzes the cleavage of N-terminal signal sequences of proteins targeted to the endoplasmic reticulum. Signal peptide cleavage occurs during the translocation (cotranslationally or post-translationally) through the translocon pore into the endoplasmic reticulum	SEC11	3.4.21.89	ko:K13280	ko03060, map03060

FUN_006428	FAD binding	-	1.14.13.1	ko:K00480	ko00621, ko00624, ko00626, ko01100, ko01120, ko01220, map00621, map00624, map00626, map01100, map01120, map01220
FUN_006430	Glycoside hydrolase family 78 protein	-	-	-	-
FUN_006432	Domain in Tre-2, BUB2p, and Cdc16p. Probable Rab-GAPs.	-	-	ko:K19953	-
FUN_006433	Belongs to the ubiquitin-conjugating enzyme family	-	3.4.21.89	ko:K13280	ko03060, map03060
FUN_006434	BTB/POZ domain	-	-	-	-
FUN_006436	Phosphoribosyl transferase domain	-	-	ko:K07101	-
FUN_006437	Inositol-1-monophosphatase	-	3.1.3.25	ko:K01092	ko00521, ko00562, ko01100, ko04070, map00521, map00562, map01100, map04070
FUN_006438	Thioesterase superfamily	-	-	-	-
FUN_006439	Glycoside hydrolase family 55 protein	-	3.2.1.58	ko:K01210	ko00500, map00500
FUN_006441	SNF2 family N-terminal domain	rhp26	-	ko:K10841	ko03420, map03420
FUN_006442	AMP-binding enzyme	-	-	-	-
FUN_006443	DJ-1 PfpI family protein	-	-	-	-
FUN_006445	Peroxisomal membrane anchor protein (Pex14p) conserved region	PEX14	-	ko:K13343	ko04146, map04146
FUN_006448	Abscisic acid G-protein coupled receptor	-	-	ko:K22193	-
FUN_006449	Universal stress protein family	-	-	-	-
FUN_006450	Transferase family	-	-	-	-
FUN_006451	Ribosome-associated complex head domain	zuo1	4.1.3.4	ko:K01640, ko:K09522	ko00072, ko00280, ko00281, ko00650, ko01100, ko04146, map00072, map00280, map00281, map00650, map01100, map04146
FUN_006452	GRIP domain	IMH1	-	-	-
FUN_006453	Protein tyrosine kinase	SKY1	2.7.11.1	ko:K08832	-
FUN_006454	ankyrin repeat protein	-	-	-	-
FUN_006455	Major Facilitator Superfamily	-	-	-	-
FUN_006456	Alcohol acetyltransferase	ATF1	-	-	-
FUN_006458	Aminotransferase class I and II	-	2.3.1.47	ko:K00652	ko00780, ko01100, map00780, map01100
FUN_006459	polyketide synthase	-	-	-	-
FUN_006460	Mechanosensitive ion channel	-	-	ko:K22048	-
FUN_006461	Pyridoxal 5'-phosphate (PLP)-binding protein, which may be involved in intracellular homeostatic regulation of pyridoxal 5'-phosphate (PLP), the active form of vitamin B6	-	-	ko:K06997	-
FUN_006462	Aldose 1-epimerase	-	5.1.3.3	ko:K01785	ko00010, ko00052, ko01100, ko01110, ko01120, ko01130, map00010, map00052, map01100, map01110, map01120, map01130
FUN_006463	c2h2 zinc finger protein	SIP5	-	-	-
FUN_006465	Altered inheritance of mitochondria protein 6	AIM6	-	-	-
FUN_006466	Pyridoxal 5'-phosphate (PLP)-binding protein, which may be involved in intracellular homeostatic regulation of pyridoxal 5'-phosphate (PLP), the active form of vitamin B6	-	-	ko:K06997	-
FUN_006468	Belongs to the GMC oxidoreductase family	-	-	-	-
FUN_006469	amino acid	-	-	ko:K16261	-
FUN_006471	Oxidoreductase-like protein, N-terminal	-	-	-	-
FUN_006474	Adaptins are components of the adaptor complexes which link clathrin to receptors in coated vesicles. Clathrin-associated protein complexes are believed to interact with the cytoplasmic tails of membrane proteins, leading to their selection and concentration	-	-	ko:K11824	ko04144, ko04721, ko04961, ko05016, map04144, map04721, map04961, map05016
FUN_006478	Domain of unknown function (DUF1768)	-	-	ko:K09935	-

FUN_006479	Fungal specific transcription factor domain	-	-	-	-
FUN_006480	Belongs to the protein kinase superfamily	SKS1	-	-	-
FUN_006481	integral membrane protein	-	-	-	-
FUN_006483	Fes/CIP4 homology domain	HOF1	-	ko:K20121	-
FUN_006484	Domain of unknown function (DUF3819)	CDC39	-	ko:K12604	ko03018, map03018
FUN_006486	Oxidoreductase family, NAD-binding Rossmann fold	-	-	-	-
FUN_006489	ATPase family associated with various cellular activities (AAA)	-	-	-	-
FUN_006491	Belongs to the peptidase C19 family	-	3.4.19.12	ko:K11874	-
FUN_006492	Ribosomal protein L9, N-terminal domain	-	-	-	-
FUN_006493	Fructose-bisphosphate aldolase class-II	FBA1	3.2.1.25, 4.1.2.13	ko:K01192, ko:K01624	ko00010, ko00030, ko00051, ko00511, ko00680, ko00710, ko01100, ko01110, ko01120, ko01130, ko01200, ko01230, ko04142, map00010, map00030, map00051, map00511, map00680, map00710, map01100, map01110, map01120, map01130, map01200, map01230, map04142
FUN_006495	Amidohydrolase family	-	3.5.4.3	ko:K01487	ko00230, ko01100, map00230, map01100
FUN_006496	Belongs to the actin family	ARP9	3.1.1.73	ko:K09252, ko:K11768	-
FUN_006497	Intracellular phospholipase B that catalyzes the double deacylation of phosphatidylcholine (PC) to glycerophosphocholine (GroPCho). Plays an important role in membrane lipid homeostasis	NTE1	3.1.1.5, 3.1.3.99	ko:K14676, ko:K18550	ko00230, ko00564, ko00760, ko01100, map00230, map00564, map00760, map01100
FUN_006498	GH65-CBM32. glycoside hydrolase family 65	ATH1	3.2.1.28	ko:K01194	ko00500, ko01100, map00500, map01100
FUN_006499	The glycine cleavage system catalyzes the degradation of glycine	GCV2	1.4.4.2	ko:K00281	ko00260, ko00630, ko01100, ko01110, ko01130, ko01200, map00260, map00630, map01100, map01110, map01130, map01200
FUN_006501	Electron transfer flavoprotein-ubiquinone oxidoreductase, 4Fe-4S	-	1.5.5.1	ko:K00311	-
FUN_006502	RNA recognition motif	-	-	ko:K12893, ko:K14651	ko03022, ko03040, ko05168, ko05202, map03022, map03040, map05168, map05202
FUN_006503	Beta-lactamase	-	-	-	-
FUN_006504	Transferrin receptor-like dimerisation domain	-	3.4.17.21	ko:K01301	-
FUN_006505	Belongs to the short-chain dehydrogenases reductases (SDR) family	-	1.1.1.100	ko:K00059	ko00061, ko00333, ko00780, ko01040, ko01100, ko01130, ko01212, map00061, map00333, map00780, map01040, map01100, map01130, map01212
FUN_006508	Ferritin-like domain	-	-	-	-
FUN_006509	Belongs to the class I-like SAM-binding methyltransferase superfamily. Trm1 family	TRM1	2.1.1.215, 2.1.1.216	ko:K00555	-
FUN_006512	RING-variant domain	-	-	-	-
FUN_006513	Transport and Golgi organisation 2	-	-	-	-
FUN_006516	GPI anchored protein	-	-	-	-
FUN_006517	Protein of unknown function (DUF3112)	-	-	-	-
FUN_006520	Transcription factor tfiiic complex subunit	-	-	ko:K15205	-
FUN_006521	Cytochrome b2	-	1.1.2.3	ko:K00101	ko00620, ko01100, map00620, map01100
FUN_006522	Belongs to the major facilitator superfamily. Sugar transporter (TC 2.A.1.1) family	-	-	-	-
FUN_006524	Lactonase, 7-bladed beta-propeller	-	-	-	-
FUN_006525	Lactonase, 7-bladed beta-propeller	-	-	-	-
FUN_006526	Belongs to the oxygen-dependent FAD-linked oxidoreductase family	-	-	-	-

FUN_006529	SET (Su(var)3-9, Enhancer-of-zeste, Trithorax) domain	-	-	-	-
FUN_006530	Leucine rich repeat	-	-	-	-
FUN_006532	Belongs to the eukaryotic ribosomal protein eL13 family	rpl13	-	ko:K02873	ko03010, map03010
FUN_006533	Protein HRI1	-	-	-	-
FUN_006535	Predicted integral membrane zinc-ribbon metal-binding protein	-	-	-	-
FUN_006536	RNA recognition motif	SLT11	-	ko:K12872	ko03040, map03040
FUN_006537	Catalyzes the methyl transfer from S-adenosyl-methionine to the C-24 of zymosterol to form fecosterol	ERG6	2.1.1.41	ko:K00559	ko00100, ko01100, ko01110, ko01130, map00100, map01100, map01110, map01130
FUN_006538	Transketolase, pyrimidine binding domain	PDB1	1.2.4.1	ko:K00162	ko00010, ko00020, ko00620, ko01100, ko01110, ko01120, ko01130, ko01200, ko04066, ko04922, ko05230, map00010, map00020, map00620, map01100, map01110, map01120, map01130, map01200, map04066, map04922, map05230
FUN_006540	reductase	CSH1	1.1.1.353	ko:K17647	ko00254, map00254
FUN_006541	Major Facilitator Superfamily	-	-	-	-
FUN_006542	Belongs to the DEAD box helicase family	RRP3	3.6.4.13	ko:K14777	-
FUN_006543	Brix	SSF1	-	ko:K14859	-
FUN_006545	Haemolysin-III related	-	-	ko:K07297	ko04152, ko04211, ko04920, ko04932, map04152, map04211, map04920, map04932
FUN_006547	Major Facilitator Superfamily	-	-	-	-
FUN_006548	Belongs to the short-chain dehydrogenases reductases (SDR) family	-	-	-	-
FUN_006549	Short chain dehydrogenase reductase	-	-	-	-
FUN_006550	Fungal specific transcription factor domain	-	-	-	-
FUN_006551	Aldo/keto reductase family	-	1.1.1.116	ko:K00062	-
FUN_006552	Belongs to the aldehyde dehydrogenase family	-	1.2.1.8	ko:K00130	ko00260, ko01100, map00260, map01100
FUN_006553	Lycopene cyclase protein	-	-	-	-
FUN_006554	Heterokaryon incompatibility protein (HET)	-	-	-	-
FUN_006555	Transcription factor TFIID (or TATA-binding protein, TBP)	tbp1	-	ko:K03120	ko03022, ko05016, ko05165, ko05166, ko05168, ko05169, ko05203, map03022, map05016, map05165, map05166, map05168, map05169, map05203
FUN_006556	FAD dependent oxidoreductase	-	1.5.3.1, 1.5.3.7	ko:K00306	ko00260, ko00310, ko01100, ko04146, map00260, map00310, map01100, map04146
FUN_006557	Methyltransferase domain	-	-	ko:K17803	-
FUN_006559	Uncharacterized alpha/beta hydrolase domain (DUF2235)	-	-	-	-
FUN_006561	Acetolactate synthase	ILV2	2.2.1.6	ko:K01652	ko00290, ko00650, ko00660, ko00770, ko01100, ko01110, ko01130, ko01210, ko01230, map00290, map00650, map00660, map00770, map01100, map01110, map01130, map01210, map01230
FUN_006562	Tetratricopeptide repeat	-	-	ko:K20309	-
FUN_006563	DJ-1/PfpI family	-	-	-	-
FUN_006567	Cytochrome P450	-	-	-	-
FUN_006568	Glycosyltransferase family 2 protein	DPM1	2.4.1.83	ko:K00721	ko00510, ko01100, map00510, map01100
FUN_006569	Saccharomyces cerevisiae YMR130W	-	-	ko:K07025	-
FUN_006570	ABC transporter transmembrane region	-	-	-	-
FUN_006572	Galactokinase galactose-binding signature	GAL1	2.7.1.6	ko:K00849	ko00052, ko00520, ko01100, map00052, map00520, map01100
FUN_006573	DASH complex subunit Duo1	-	-	-	-

FUN_006575	Tubulin-tyrosine ligase family	-	-	-	-
FUN_006576	Primase zinc finger	-	-	ko:K10736	-
FUN_006577	Ydr279p protein family (RNase H2 complex component)	-	-	-	-
FUN_006579	Protein of unknown function (DUF1691)	-	-	-	-
FUN_006581	Belongs to the major facilitator superfamily. Sugar transporter (TC 2.A.1.1) family	-	-	ko:K08141	-
FUN_006582	Alpha-amylase domain	-	3.2.1.10	ko:K01182	ko00052, ko00500, ko01100, map00052, map00500, map01100
FUN_006583	Belongs to the major facilitator superfamily. Sugar transporter (TC 2.A.1.1) family	-	-	ko:K08141	-
FUN_006586	Major Facilitator Superfamily	-	-	-	-
FUN_006588	NAD dependent epimerase/dehydratase family	-	1.1.1.283	ko:K17741	ko00620, ko00640, ko04011, map00620, map00640, map04011
FUN_006590	Alpha/beta hydrolase family	-	-	-	-
FUN_006597	NAD dependent epimerase dehydratase	-	-	-	-
FUN_006598	Belongs to the type-B carboxylesterase lipase family	-	5.4.3.8	ko:K01845	ko00860, ko01100, ko01110, ko01120, map00860, map01100, map01110, map01120
FUN_006602	Pyridine nucleotide-disulphide oxidoreductase	-	-	-	-
FUN_006603	Belongs to the major facilitator superfamily. Sugar transporter (TC 2.A.1.1) family	-	-	-	-
FUN_006604	fungal specific transcription factor	-	-	-	-
FUN_006605	SET (Su(var)3-9, Enhancer-of-zeste, Trithorax) domain	-	-	-	-
FUN_006606	alpha/beta hydrolase fold	-	-	-	-
FUN_006607	EthD domain	-	-	-	-
FUN_006608	ThiJ/PfpI family-like	-	-	-	-
FUN_006609	Cytochrome P450	-	1.14.14.54	ko:K10437, ko:K21295	ko00360, ko00643, ko01100, ko01120, map00360, map00643, map01100, map01120
FUN_006610	polyketide synthase	-	-	-	-
FUN_006613	Heterokaryon incompatibility protein	-	-	-	-
FUN_006615	Reticulon-like protein	-	-	-	-
FUN_006616	integral membrane protein	-	-	-	-
FUN_006617	Fungal specific transcription factor domain	-	-	-	-
FUN_006618	Sugar (and other) transporter	-	-	-	-
FUN_006619	Belongs to the ubiquitin-conjugating enzyme family	-	-	-	-
FUN_006620	NAD dependent epimerase dehydratase family protein	-	-	-	-
FUN_006622	E1-E2 ATPase	-	3.6.3.4	ko:K01533	-
FUN_006623	Rad4 beta-hairpin domain 1	-	-	ko:K10838	ko03420, map03420
FUN_006624	Heterokaryon incompatibility protein (HET)	-	-	-	-
FUN_006625	Belongs to the glutamine synthetase family	GLN1	6.3.1.2	ko:K01915	ko00220, ko00250, ko00630, ko00910, ko01100, ko01120, ko01230, ko02020, ko04217, ko04724, ko04727, map00220, map00250, map00630, map00910, map01100, map01120, map01230, map02020, map04217, map04724, map04727
FUN_006626	Component of the Mediator complex, a coactivator involved in the regulated transcription of nearly all RNA polymerase II-dependent genes. Mediator functions as a bridge to convey information from gene-specific regulatory proteins to the basal RNA polymerase II transcription machinery. Mediator is recruited to promoters by direct interactions with regulatory proteins and serves as a scaffold for the assembly of a functional preinitiation complex with RNA polymerase II and the general transcription	-	-	-	-

FUN_006628	Glutathione-dependent formaldehyde-activating enzyme	-	-	-	-
FUN_006629	Transcription factor TFIIH complex subunit Tfb5	-	-	ko:K10845	ko03022, ko03420, map03022, map03420
FUN_006631	Belongs to the type-B carboxylesterase lipase family	-	-	-	-
FUN_006632	cytokinin dehydrogenase activity	-	1.1.2.4	ko:K00102	ko00620, map00620
FUN_006633	Amidase	-	-	-	-
FUN_006634	SLIDE	ISW2	-	ko:K11654	-
FUN_006635	FAD binding domain	-	-	-	-
FUN_006637	Class II Aldolase and Adducin N-terminal domain	-	-	-	-
FUN_006638	Lipase 3 N-terminal region	-	-	-	-
FUN_006639	ATPase required for the post-translational delivery of tail-anchored (TA) proteins to the endoplasmic reticulum. Recognizes and selectively binds the transmembrane domain of TA proteins in the cytosol. This complex then targets to the endoplasmic reticulum by membrane-bound receptors, where the tail- anchored protein is released for insertion. This process is regulated by ATP binding and hydrolysis. ATP binding drives the homodimer towards the closed dimer state, facilitating recognition of newly synthesized TA membrane proteins. ATP hydrolysis is required for insertion. Subsequently, the homodimer reverts towards the open dimer state, lowering its	GET3	3.6.3.16	ko:K01551	-
FUN_006641	KR domain	-	-	-	-
FUN_006642	Src homology 3 domains	PIN3	-	ko:K20522	-
FUN_006646	fad binding domain protein	-	1.14.13.1	ko:K00480	ko00621, ko00624, ko00626, ko01100, ko01120, ko01220, map00621, map00624, map00626, map01100, map01120, map01220
FUN_006647	alcohol dehydrogenase	-	-	-	-
FUN_006648	Domain in Tre-2, BUB2p, and Cdc16p. Probable Rab-GAPs.	-	-	-	-
FUN_006649	Major Facilitator Superfamily	-	-	-	-
FUN_006650	Zinc-binding dehydrogenase	-	-	-	-
FUN_006651	Multicopper oxidase	-	-	-	-
FUN_006653	Alpha/beta hydrolase family	-	-	-	-
FUN_006654	Uso1 / p115 like vesicle tethering protein, head region	USO1	2.1.1.319	ko:K11436, ko:K20361	-
FUN_006655	XRN 5'-3' exonuclease N-terminus	RAT1	-	ko:K12619	ko03008, ko03018, map03008, map03018
FUN_006656	Major Facilitator Superfamily	-	-	-	-
FUN_006658	integral membrane protein	-	-	-	-
FUN_006660	Cytochrome domain of cellobiose dehydrogenase	-	-	-	-
FUN_006662	Predicted membrane protein (DUF2231)	-	-	-	-
FUN_006663	cytokinin dehydrogenase activity	-	1.1.2.4	ko:K00102	ko00620, map00620
FUN_006666	Fungal trichothecene efflux pump (TRI12)	-	-	-	-
FUN_006667	PCRF	MRF1	-	ko:K02835	-
FUN_006668	Gamma-glutamyltranspeptidase	-	2.3.2.2, 3.4.19.13	ko:K00681	ko00430, ko00460, ko00480, ko01100, map00430, map00460, map00480, map01100
FUN_006670	Tpr domain-containing protein	-	-	-	-
FUN_006671	SOS response associated peptidase (SRAP)	-	-	-	-
FUN_006672	2OG-Fe(II) oxygenase superfamily	-	-	-	-

FUN_006673	Belongs to the glycosyl hydrolase 43 family	-	-	-	-
FUN_006676	WD domain, G-beta repeat	cpc2	-	ko:K14753	ko05162, map05162
FUN_006677	NmrA-like family	-	-	-	-
FUN_006678	Component of the spliceosomal U1 snRNP, which is essential for recognition of the pre-mRNA 5' splice-site and the subsequent assembly of the spliceosome. U1-C is directly involved in initial 5' splice-site recognition for both constitutive and regulated alternative splicing. The interaction with the 5' splice-site seems to precede base-pairing between the pre-mRNA and the U1 snRNA. Stimulates commitment or early (E) complex formation by stabilizing the base pairing of the 5' end of the U1 snRNA and	YHC1	-	ko:K11095	ko03040, map03040
FUN_006679	Calponin homology domain	SCP1	-	ko:K20526	-
FUN_006680	Accessory subunit of the mitochondrial membrane respiratory chain NADH dehydrogenase (Complex I), that is believed not to be involved in catalysis. Complex I functions in the transfer of electrons from NADH to the respiratory chain. The immediate electron acceptor for the enzyme is believed to be ubiquinone	-	-	ko:K18160	ko04714, map04714
FUN_006682	Belongs to the peptidase S10 family	-	-	-	-
FUN_006684	RTA1 like protein	-	-	-	-
FUN_006687	Glyoxalase-like domain	-	-	-	-
FUN_006688	Aminopeptidase P, N-terminal domain	ICP55	3.4.11.26	ko:K18573	-
FUN_006689	Sir2 family	-	-	-	-
FUN_006690	Fringe-like	-	-	-	-
FUN_006691	Transcription factor PAP1	YAP1	-	ko:K09043	-
FUN_006693	Catalytic subunit of the poly(A)-nuclease (PAN) deadenylation complex, one of two cytoplasmic mRNA deadenylases involved in mRNA turnover. PAN specifically shortens poly(A) tails of RNA and the activity is stimulated by poly(A)-binding protein PAB1. PAN deadenylation is followed by rapid degradation of the shortened mRNA tails by the CCR4-NOT complex. Deadenylated mRNAs are then degraded by two alternative mechanisms, namely exosome- mediated 3'-5' exonucleolytic degradation, or deadenlyation- dependent mRNA decaping and subsequent 5'-3' exonucleolytic degradation by XRN1. May also be involved in post-transcriptional maturation of	PAN2	3.1.13.4	ko:K12571	ko03018, map03018
FUN_006695	NADH:flavin oxidoreductase / NADH oxidase family	-	1.6.99.1	ko:K00354	-
FUN_006696	Methyltransferase small domain	-	-	-	-
FUN_006697	Glycosyltransferase family 2 protein	CHS3	2.4.1.16	ko:K00698	ko00520, map00520
FUN_006701	Belongs to the ketol-acid reductoisomerase family	ILV5	1.1.1.86	ko:K00053	ko00290, ko00770, ko01100, ko01110, ko01130, ko01210, ko01230, map00290, map00770, map01100, map01110, map01130, map01210, map01230
FUN_006702	Belongs to the ubiquitin-conjugating enzyme family	UBC12	-	ko:K10579	ko04120, map04120
FUN_006703	RibD C-terminal domain	RIB7	1.1.1.302	ko:K14654	ko00740, ko01100, map00740, map01100
FUN_006704	emp24/gp25L/p24 family/GOLD	ERV25	-	ko:K20352	-
FUN_006705	Enoyl-(Acyl carrier protein) reductase	-	-	-	-
FUN_006706	RAVE protein 1 C terminal	RAV1	-	-	-
FUN_006707	Belongs to the class I-like SAM-binding methyltransferase superfamily. Protein arginine N- methyltransferase family	rmt3	2.1.1.319	ko:K11436	-
FUN_006710	Enoyl-(Acyl carrier protein) reductase	-	-	-	-
FUN_006711	Heterokaryon incompatibility protein (HET)	-	-	-	-
FUN_006715	CASC3/Barentsz eIF4AIII binding	-	-	-	-

FUN_006716	C-x8-c-x5-c-x3-h type zinc finger protein	-	-	-	-
FUN_006718	Belongs to the STXBP unc-18 SEC1 family	SLY1	-	ko:K19998	-
FUN_006719	Heterokaryon incompatibility protein (HET)	-	-	-	-
FUN_006720	Universal stress protein family	-	-	-	-
FUN_006721	Ribosomal protein S7p/S5e	-	-	ko:K02992	ko03010, map03010
FUN_006722	Heterokaryon incompatibility protein (HET)	-	-	-	-
FUN_006723	Belongs to the argonaute family	-	-	ko:K11593	-
FUN_006725	Exocyst complex component SEC3 N-terminal PIP2 binding PH	-	-	ko:K19983	-
FUN_006726	SRP40, C-terminal domain	-	-	-	-
FUN_006727	Essential component of the vacuolar proton pump (V- ATPase), a multimeric enzyme that catalyzes the translocation of protons across the membranes. Required for assembly and activity of the V-ATPase	VPH1	-	ko:K02154	ko00190, ko01100, ko04142, ko04145, ko04721, ko04966, ko05110, ko05120, ko05152, ko05165, ko05323, map00190, map01100, map04142, map04145, map04721, map04966, map05110, map05120, map05152, map05165, map05323
FUN_006728	Aminotransferase class I and II	-	-	-	-
FUN_006729	Protein of unknown function (DUF1295)	-	-	-	-
FUN_006730	50S ribosome-binding GTPase	-	-	ko:K03978	-
FUN_006732	Sec63 Brl domain	HFM1	3.6.4.12	ko:K15271	-
FUN_006733	Nucleotide-sugar transporter	gms1	-	ko:K15272	-
FUN_006735	Recruits TFIIH to the initiation complex and stimulates the RNA polymerase II C-terminal domain kinase and DNA-dependent ATPase activities of TFIIH. Both TFIIH and TFIIE are required for promoter clearance by RNA polymerase	-	-	ko:K03137	ko03022, ko05169, ko05203, map03022, map05169, map05203
FUN_006736	O-methyltransferase that catalyzes the 2 O-methylation steps in the ubiquinone biosynthetic pathway	COQ3	2.1.1.114, 2.1.1.222, 2.1.1.64	ko:K00568, ko:K00591	ko00130, ko01100, ko01110, map00130, map01100, map01110
FUN_006737	Mitochondrial ribosomal protein L27	MRPL27	-	ko:K17422	-
FUN_006738	Utp14 protein	-	-	ko:K14567	ko03008, map03008
FUN_006739	Glycoside hydrolase family 81 protein	ACF2	3.2.1.6	ko:K01180	-
FUN_006740	Belongs to the Ca(2) cation antiporter (CaCA) (TC 2.A.19) family	VCX1	-	ko:K07300	-
FUN_006744	TAP-like protein	-	-	-	-
FUN_006746	Metal dependent phosphohydrolase	-	-	-	-
FUN_006747	Carbonic anhydrase	-	-	-	-
FUN_006748	NACHT domain	-	-	-	-
FUN_006750	Domain of unknown function (DUF1996)	-	-	-	-
FUN_006751	Telomere regulation protein Stn1	-	-	-	-
FUN_006753	DnaJ molecular chaperone homology domain	-	-	ko:K09529	-
FUN_006756	Transporter	-	-	-	-
FUN_006759	Belongs to the short-chain dehydrogenases reductases (SDR) family	-	-	-	-
FUN_006761	Enoyl-(Acyl carrier protein) reductase	-	-	-	-
FUN_006762	Calcium-activated chloride channel	-	-	ko:K19327	-
FUN_006763	integral membrane protein	-	-	-	-
FUN_006764	Survival protein SurE	-	3.1.3.5	ko:K03787	ko00230, ko00240, ko00760, ko01100, ko01110, map00230, map00240, map00760, map01100, map01110
FUN_006765	Isochorismatase family	-	-	-	-

FUN_006766	Aminotransferase class I and II	HIS5	2.6.1.9	ko:K00817	ko00340, ko00350, ko00360, ko00400, ko00401, ko00960, ko01100, ko01110, ko01130, ko01230, map00340, map00350, map00360, map00400, map00401, map00960, map01100, map01110, map01130, map01230
FUN_006767	GTP1/OBG	MTG2	1.14.11.27	ko:K03979, ko:K10277	-
FUN_006768	Belongs to the short-chain dehydrogenases reductases (SDR) family	-	-	-	-
FUN_006771	Belongs to the TRAFAC class myosin-kinesin ATPase superfamily. Kinesin family	-	-	ko:K10396	ko04144, ko04728, map04144, map04728
FUN_006772	Belongs to the ubiquitin-conjugating enzyme family	UBC2	2.3.2.23	ko:K10573, ko:K10574	ko04120, map04120
FUN_006773	Sulfatase	-	3.1.6.6	ko:K01133	-
FUN_006774	Belongs to the DEAD box helicase family	MSS116	3.6.4.13	ko:K17679	-
FUN_006776	Glycosyl transferase family 8	GolS4	2.4.1.123	ko:K18819	ko00052, map00052
FUN_006777	60s ribosomal protein l7	RPL7	-	ko:K02937	ko03010, map03010
FUN_006778	Belongs to the class I-like SAM-binding methyltransferase superfamily. RsmB NOP	NCL1	2.1.1.202	ko:K15334	-
FUN_006779	Methyltransferase required for the conversion of 2- polyprenyl-6-methoxy-1, 4-benzoquinol (DDMQH2) to 2-polyprenyl-3- methyl-6-methoxy-1, 4-benzoquinol	COQ5	2.1.1.201	ko:K06127	ko00130, ko01100, ko01110, map00130, map01100, map01110
FUN_006780	Dehydrogenase E1 component	-	1.2.4.4	ko:K00166	ko00280, ko00640, ko01100, ko01110, ko01130, map00280, map00640, map01100, map01110, map01130
FUN_006782	Belongs to the ABC transporter superfamily. ABCG family. PDR (TC 3.A.1.205)	SNQ2	-	ko:K08712	ko02010, map02010
FUN_006783	Phosphoenolpyruvate carboxykinase	PCK1	4.1.1.49	ko:K01610	ko00010, ko00020, ko00620, ko00710, ko01100, ko01110, ko01120, ko01130, ko01200, map00010, map00020, map00620, map00710, map01100, map01110, map01120, map01130, map01200
FUN_006784	Eukaryotic membrane protein family	-	-	-	-
FUN_006785	Protein tyrosine kinase	PKC1	2.7.11.1	ko:K08793	-
FUN_006786	Belongs to the ubiquitin-conjugating enzyme family	ubc8	2.3.2.23	ko:K10576	ko04120, map04120
FUN_006787	Catalyzes the reversible reaction in which hydroxymethyl group from 5, 10-methylenetetrahydrofolate is transferred onto alpha-ketoisovalerate to form ketopantoate	ECM31	2.1.2.11	ko:K00606	ko00770, ko01100, ko01110, map00770, map01100, map01110
FUN_006788	Ubiquitin-like protein involved in cytoplasm to vacuole transport (Cvt)	ATG12	-	ko:K08336	ko04068, ko04136, ko04138, ko04140, ko04621, ko04622, map04068, map04136, map04138, map04140, map04621, map04622
FUN_006789	Zinc finger in DBF-like proteins	DBF4	-	ko:K02309	ko04111, ko04113, map04111, map04113
FUN_006791	Ring finger domain	-	-	-	-
FUN_006792	C6 zinc finger	-	-	-	-
FUN_006794	Guanine nucleotide exchange factor synembryn	-	-	-	-
FUN_006795	Rap1-interacting factor 1 N terminal	-	-	-	-
FUN_006796	Catalyzes the interconversion between the alpha and beta anomers from at least three hexose 6-phosphate sugars (Glc6P, Gal6P, and Man6P)	YMR099C	5.1.3.15	ko:K01792	ko00010, ko01100, ko01110, ko01120, ko01130, map00010, map01100, map01110, map01120, map01130
FUN_006797	Protein tyrosine kinase	IKS1	-	ko:K08286	-
FUN_006798	Centrosome microtubule-binding domain of Cep57	-	-	-	-
FUN_006799	phosphoric diester hydrolase activity	-	-	-	-
FUN_006800	Belongs to the cytochrome P450 family	-	-	-	-
FUN_006801	Rsm1-like	-	-	-	-

FUN_006802	Inositol monophosphatase family	MET22	3.1.3.7	ko:K01082	ko00920, ko01100, ko01120, ko01130, map00920, map01100, map01120, map01130
FUN_006804	Belongs to the universal ribosomal protein uL6 family	MRPL6	-	ko:K02933	ko03010, map03010
FUN_006805	IEC3 subunit of the Ino80 complex, chromatin re-modelling	-	-	-	-
FUN_006806	ORMDL family	ORM1	-	-	-
FUN_006807	Sodium/hydrogen exchanger family	-	-	-	-
FUN_006808	Mitochondrial ribosomal subunit protein	RSM24	-	ko:K17413	-
FUN_006809	Involved in protein N-glycosylation. Essential for the second step of the dolichol-linked oligosaccharide pathway	ALG13	2.4.1.141	ko:K07432	ko00510, ko00513, ko01100, map00510, map00513, map01100
FUN_006810	Cupin	-	-	-	-
FUN_006812	Belongs to the glycosyl hydrolase 5 (cellulase A) family	EXG2	3.2.1.58	ko:K01210	ko00500, map00500
FUN_006813	Belongs to the peptidase S8 family	-	-	-	-
FUN_006814	Belongs to the peptidase S8 family	-	-	-	-
FUN_006815	Belongs to the multicopper oxidase family	-	-	-	-
FUN_006816	GDP-fucose transporter	-	-	-	-
FUN_006817	Domain of unknown function (DUF3425)	-	-	-	-
FUN_006818	SNF2 family N-terminal domain	-	-	-	-
FUN_006822	acyl-CoA dehydrogenase	-	1.3.8.7	ko:K00249	ko00071, ko00280, ko00410, ko00640, ko01100, ko01110, ko01130, ko01200, ko01212, ko03320, map00071, map00280, map00410, map00640, map01100, map01110, map01130, map01200, map01212, map03320
FUN_006823	Inner centromere protein, ARK binding region	-	-	-	-
FUN_006824	Membrane-associating domain	NCE102	-	-	-
FUN_006825	PSP1 C-terminal conserved region	-	-	-	-
FUN_006827	Belongs to the eukaryotic ribosomal protein eL15 family	rpl15	-	ko:K02877	ko03010, map03010
FUN_006828	DnaJ molecular chaperone homology domain	-	-	-	-
FUN_006831	PHD/FYVE-zinc-finger like domain	-	-	-	-
FUN_006833	P-loop containing Nucleoside Triphosphate Hydrolase	-	3.6.4.13	ko:K13983, ko:K18422	-
FUN_006834	Belongs to the glycosyl hydrolase 43 family	-	-	-	-
FUN_006835	Heterokaryon incompatibility protein (HET)	-	-	-	-
FUN_006836	Nudix family	-	3.6.1.21	ko:K18447	ko00051, ko00230, ko00500, ko01100, ko01110, map00051, map00230, map00500, map01100, map01110
FUN_006837	TRAM, LAG1 and CLN8 homology domains.	-	-	-	-
FUN_006838	Heterokaryon incompatibility protein (HET)	-	-	-	-
FUN_006839	Seryl-tRNA synthetase N-terminal domain	DIA4	6.1.1.11	ko:K01875	ko00970, map00970
FUN_006841	Molybdate transporter of MFS superfamily	-	-	-	-
FUN_006842	Belongs to the type-B carboxylesterase lipase family	-	-	-	-
FUN_006843	Belongs to the ATG13 family. Fungi subfamily	ATG13	-	ko:K08331	ko04136, ko04138, ko04140, ko04211, map04136, map04138, map04140, map04211
FUN_006844	Regulator of chromosome condensation (RCC1) repeat	-	-	-	-
FUN_006845	KR domain	-	-	-	-
FUN_006846	Carbohydrate esterase family 16 protein	-	-	-	-
FUN_006847	Lactonase, 7-bladed beta-propeller	-	-	-	-

FUN_006848	Lactonase, 7-bladed beta-propeller	-	-	-	-
FUN_006849	Copper-fist	HAA1	-	-	-
FUN_006850	Belongs to the globin family	-	1.14.12.17	ko:K05916	ko05132, map05132
FUN_006852	ARID/BRIGHT DNA binding domain	RSC9	-	ko:K11763	-
FUN_006853	PHD zinc finger	-	2.5.1.87	ko:K11778, ko:K19197	ko00900, ko01110, map00900, map01110
FUN_006855	Up-regulated During Septation	-	-	-	-
FUN_006856	N-acetyltransferase	-	-	-	-
FUN_006857	NADH-ubiquinone oxidoreductase MWFE subunit	-	-	-	-
FUN_006858	Belongs to the MYST (SAS MOZ) family	SAS3	2.3.1.48	ko:K11306, ko:K11378	-
FUN_006859	Belongs to the cytochrome P450 family	-	-	-	-
FUN_006860	Phosphate acyltransferases	SCT1	2.3.1.15, 2.3.1.42	ko:K13507	ko00561, ko00564, ko01100, ko01110, map00561, map00564, map01100, map01110
FUN_006861	Glycoside hydrolase family 76 protein	-	3.2.1.101	ko:K08257	-
FUN_006862	cell division	CCDC124	-	ko:K02357	-
FUN_006863	Hydrolyzes a variety of simple alpha-D-galactoside as well as more complex molecules such as oligosaccharides and polysaccharides	aglA	3.2.1.22	ko:K07407	ko00052, ko00561, ko00600, ko00603, map00052, map00561, map00600, map00603
FUN_006864	CDC45-like protein	CDC45	-	ko:K06628	ko04110, ko04111, ko04113, map04110, map04111, map04113
FUN_006865	Belongs to the MIP aquaporin (TC 1.A.8) family	FPS1	-	ko:K03441	-
FUN_006866	Destroys radicals which are normally produced within the cells and which are toxic to biological systems	SOD2	1.15.1.1	ko:K04564	ko04013, ko04068, ko04146, ko04211, ko04212, ko04213, ko05016, map04013, map04068, map04146, map04211, map04212, map04213, map05016
FUN_006867	Siderophore biosynthesis protein domain	-	-	-	-
FUN_006868	Peptidase C13 family	GPI8	-	ko:K05290	ko00563, ko01100, map00563, map01100
FUN_006869	OPT oligopeptide transporter protein	OPT8	-	-	-
FUN_006873	G-patch domain-containing protein	-	-	ko:K13103	-
FUN_006875	Major Facilitator Superfamily	-	-	-	-
FUN_006876	Ubiquinol-cytochrome C reductase hinge protein	QCR6	-	ko:K00416	ko00190, ko01100, ko04260, ko04714, ko04932, ko05010, ko05012, ko05016, map00190, map01100, map04260, map04714, map04932, map05010, map05012, map05016
FUN_006877	Aldolase/RraA	-	-	-	-
FUN_006878	Peptidase family M41	YME1	-	ko:K08955	ko04139, map04139
FUN_006879	Serine/Threonine protein kinases, catalytic domain	PKH1	2.7.11.1	ko:K06276	ko01524, ko03320, ko04011, ko04068, ko04071, ko04140, ko04150, ko04151, ko04152, ko04210, ko04510, ko04660, ko04664, ko04722, ko04910, ko04919, ko04931, ko04960, ko05145, ko05160, ko05205, ko05213, ko05215, ko05223, ko05231, map01524, map03320, map04011, map04068, map04071, map04140, map04150, map04151, map04152, map04210, map04510, map04660, map04664, map04722, map04910, map04919, map04931, map04960, map05145, map05160, map05205, map05213, map05215, map05223, map05231

FUN_006880	SWIB/MDM2 domain	ssr3	2.7.11.1	ko:K06276, ko:K11650, ko:K11760, ko:K11775	ko01524, ko03320, ko04011, ko04068, ko04071, ko04140, ko04150, ko04151, ko04152, ko04210, ko04510, ko04660, ko04664, ko04714, ko04722, ko04910, ko04919, ko04931, ko04960, ko05145, ko05160, ko05205, ko05213, ko05215, ko05223, ko05225, ko05231, map01524, map03320, map04011, map04068, map04071, map04140, map04150, map04151, map04152, map04210, map04510, map04660, map04664, map04714, map04722, map04910, map04919, map04931, map04960, map05145, map05160, map05205, map05213, map05215, map05223, map05225, map05231
FUN_006881	S-adenosylhomocysteine deaminase activity	-	-	-	-
FUN_006883	Aldose 1-epimerase	-	5.1.3.3	ko:K01785	ko00010, ko00052, ko01100, ko01110, ko01120, ko01130, map00010, map00052, map01100, map01110, map01120, map01130
FUN_006885	DNA binding domain with preference for A/T rich regions	-	-	-	-
FUN_006886	Xeroderma pigmentosum G N-region	-	-	ko:K15339	-
FUN_006887	Belongs to the ubiquitin-conjugating enzyme family	-	2.3.2.23	ko:K10582	ko04120, map04120
FUN_006889	Baculoviral inhibition of apoptosis protein repeat	-	-	-	-
FUN_006890	Mitochondrial matrix Mmp37	TAM41	-	ko:K17807	-
FUN_006891	MobA-like NTP transferase domain	-	2.7.7.13	ko:K00966	ko00051, ko00520, ko01100, ko01110, map00051, map00520, map01100, map01110
FUN_006892	Belongs to the iron ascorbate-dependent oxidoreductase family	-	-	-	-
FUN_006893	Protein of unknown function (DUF1349)	-	-	ko:K09702	-
FUN_006894	Belongs to the complex I 30 kDa subunit family	ALI1	1.6.5.3, 1.6.99.3	ko:K03936	ko00190, ko01100, ko04714, ko04723, ko04932, ko05010, ko05012, ko05016, map00190, map01100, map04714, map04723, map04932, map05010, map05012, map05016
FUN_006895	RING/Ubox like zinc-binding domain	NOT4	2.3.2.27	ko:K10643	ko03018, map03018
FUN_006897	zinc finger	-	-	-	-
FUN_006898	MOSC N-terminal beta barrel domain	-	-	ko:K07140	-
FUN_006899	zinc finger	-	-	-	-
FUN_006900	MRC1-like domain	-	-	ko:K11272	ko04111, map04111
FUN_006901	Plus-3 domain	RTF1	-	ko:K15178	-
FUN_006902	Catalyzes the hydroxylation of 2-polyprenyl-3-methyl-6- methoxy-1, 4-benzoquinol (DMQH2) during ubiquinone biosynthesis. Has also a structural role in the COQ enzyme complex, stabilizing other COQ polypeptides	COQ7	-	ko:K06134	ko00130, ko01100, ko01110, map00130, map01100, map01110
FUN_006904	Set domain-containing protein	-	-	-	-
FUN_006905	Belongs to the peptidase S10 family	-	3.4.16.6	ko:K01288	-
FUN_006907	Glycosyltransferase family 25 (LPS biosynthesis protein)	-	-	-	-
FUN_006908	Belongs to the peptidase S10 family	-	-	-	-
FUN_006909	Adds multiple copies of isopentenyl pyrophosphate (IPP) to farnesyl pyrophosphate (FPP) to produce dehydrodolichyl diphosphate (Dedol-PP), a precursor of dolichol which is utilized as a sugar carrier in protein glycosylation in the endoplasmic reticulum	RER2	2.5.1.87	ko:K11778	ko00900, ko01110, map00900, map01110
FUN_006911	GINS complex protein	PSF3	-	ko:K10734	-
FUN_006912	Domain of unknown function (DUF3437)	BLM3	-	ko:K06699	ko03050, map03050
FUN_006913	Ornithine cyclodeaminase/mu-crystallin family	-	-	-	-

FUN_006914	Ribosomal protein L7Ae/L30e/S12e/Gadd45 family	rpl8	-	ko:K02936	ko03010, map03010
FUN_006915	Mannosyltransferase involved in glycosylphosphatidylinositol-anchor biosynthesis	GPI18	-	ko:K07542	ko00563, ko01100, map00563, map01100
FUN_006916	Aldo/keto reductase family	-	-	ko:K15303	ko00980, map00980
FUN_006918	FAD linked oxidase-like protein	-	-	-	-
FUN_006919	cytochrome P-450	-	-	-	-
FUN_006920	cytochrome P450	-	1.14.13.78	ko:K21292	ko00904, ko01100, map00904, map01100
FUN_006921	Diaphanous GTPase-binding Domain	-	-	-	-
FUN_006922	BRCA1-associated protein 2	-	2.3.2.27	ko:K00413, ko:K10632	ko00190, ko01100, ko02020, ko04014, ko04260, ko04714, ko04932, ko05010, ko05012, ko05016, map00190, map01100, map02020, map04014, map04260, map04714, map04932, map05010, map05012, map05016
FUN_006923	A Receptor for Ubiquitination Targets	CDC4	-	ko:K03361, ko:K22071	ko04111, ko04120, map04111, map04120
FUN_006927	Tat pathway signal sequence	-	-	-	-
FUN_006930	Transmembrane amino acid transporter protein	-	-	ko:K14209	ko04138, map04138
FUN_006931	Dehydrogenase reductase	-	-	-	-
FUN_006932	Nucleoporin autopeptidase	NUP145	-	ko:K14297, ko:K18720	ko03013, ko05164, map03013, map05164
FUN_006933	EF-hand domain	CDC31	-	ko:K16466	-
FUN_006934	Core subunit of the mitochondrial membrane respiratory chain NADH dehydrogenase (Complex I) that is believed to belong to the minimal assembly required for catalysis. Complex I functions in the transfer of electrons from NADH to the respiratory chain	NDH51	1.6.5.3, 1.6.99.3	ko:K03942	ko00190, ko01100, ko04714, ko04723, ko04932, ko05010, ko05012, ko05016, map00190, map01100, map04714, map04723, map04932, map05010, map05012, map05016
FUN_006936	Animal haem peroxidase	-	-	-	-
FUN_006937	Ribosome recycling factor	RRF1	-	ko:K02838	-
FUN_006938	RTA1 domain-containing protein	-	-	-	-
FUN_006939	Positively regulates the activity of the minus-end directed microtubule motor protein dynein. May enhance dynein- mediated microtubule sliding by targeting dynein to the microtubule plus end. Required for nuclear migration during vegetative growth as well as development. Required for retrograde early endosome (EE) transport from the hyphal tip. Required for localization of dynein to the mitotic spindle poles. Recruits additional proteins to the dynein complex at SPBs	LIS1	-	ko:K16794	ko00565, ko01100, map00565, map01100
FUN_006940	TOG	-	-	ko:K16803	-
FUN_006942	Belongs to the multicopper oxidase family	lcc1	-	-	-
FUN_006943	Catalyzes the conversion of D-ribulose 5-phosphate to formate and 3, 4-dihydroxy-2-butanone 4-phosphate	RIB3	4.1.99.12	ko:K02858	ko00740, ko01100, ko01110, map00740, map01100, map01110
FUN_006944	Permease family	-	-	ko:K06901	-
FUN_006947	Belongs to the FBPase class 1 family	FBP1	3.1.3.11	ko:K03841	ko00010, ko00030, ko00051, ko00680, ko00710, ko01100, ko01110, ko01120, ko01130, ko01200, ko04152, ko04910, map00010, map00030, map00051, map00680, map00710, map01100, map01110, map01120, map01130, map01200, map04152, map04910
FUN_006949	Mitochondrial calcium uniporter	-	-	ko:K20858	ko04020, ko04218, ko04621, map04020, map04218, map04621

FUN_006952	Cyclin	PCL1	-	ko:K06656, ko:K06657	ko04111, map04111
FUN_006953	May be involved in recycling of membrane proteins between an endocytic compartment and the TGN	VPS13	-	ko:K19525	-
FUN_006954	Protein of unknown function (DUF3128)	-	-	-	-
FUN_006955	Belongs to the heat shock protein 70 family	SSA2	-	ko:K03283	ko03040, ko04010, ko04141, ko04144, ko04213, ko04612, ko04915, ko05134, ko05145, ko05162, ko05164, ko05169, map03040, map04010, map04141, map04144, map04213, map04612, map04915, map05134, map05145, map05162, map05164, map05169
FUN_006956	Protein of unknown function (DUF3429)	-	-	-	-
FUN_006957	Belongs to the FGGY kinase family	GUT1	2.7.1.30	ko:K00864	ko00561, ko01100, ko03320, ko04626, map00561, map01100, map03320, map04626
FUN_006958	3-keto-steroid reductase	ERG27	1.1.1.270	ko:K09827	ko00100, ko01100, ko01130, map00100, map01100, map01130
FUN_006960	Uba domain-containing protein ucp14	-	-	-	-
FUN_006961	Belongs to the glycosyl hydrolase 47 family	-	3.2.1.113	ko:K01230	ko00510, ko00513, ko01100, ko04141, map00510, map00513, map01100, map04141
FUN_006962	Heterokaryon incompatibility protein (HET)	-	-	-	-
FUN_006963	Peptidase family S41	-	-	-	-
FUN_006964	Pfam:WWbp	-	-	-	-
FUN_006965	Belongs to the thiolase family	POT1	2.3.1.16	ko:K07513	ko00071, ko00280, ko00592, ko01040, ko01100, ko01110, ko01130, ko01212, ko03320, ko04146, map00071, map00280, map00592, map01040, map01100, map01110, map01130, map01212, map03320, map04146
FUN_006966	Sucrase ferredoxin domain-containing protein	-	-	-	-
FUN_006971	NAD(P)-binding Rossmann-like domain	-	-	-	-
FUN_006972	Belongs to the FMO family	-	1.14.13.8	ko:K00485	ko00982, ko01120, map00982, map01120
FUN_006973	Major Facilitator Superfamily	-	-	-	-
FUN_006975	Isochorismatase family	-	-	-	-
FUN_006976	Sodium:solute symporter family	DUR4	-	ko:K20989	-
FUN_006979	NAD(P)H-binding	-	-	-	-
FUN_006981	Glycosyl hydrolase family 115	-	-	-	-
FUN_006984	Voltage-dependent anion channel	-	-	-	-
FUN_006986	Epsin N-terminal homology (ENTH) domain	-	-	ko:K12471	ko04144, map04144
FUN_006987	Zinc finger, C2H2 type	-	-	-	-
FUN_006988	Belongs to the globin family	-	1.14.12.17	ko:K05916	ko05132, map05132
FUN_006989	Sodium-phosphate symporter which plays a fundamental housekeeping role in phosphate transport	-	-	ko:K14640	-
FUN_006993	Enoyl-(Acyl carrier protein) reductase	-	-	-	-
FUN_006994	F-box domain protein	-	-	-	-

FUN_006995	G protein alpha subunit	-	-	ko:K04630	ko04015, ko04022, ko04024, ko04062, ko04071, ko04261, ko04360, ko04371, ko04540, ko04611, ko04670, ko04713, ko04723, ko04724, ko04725, ko04726, ko04727, ko04728, ko04730, ko04914, ko04915, ko04916, ko04921, ko04923, ko04924, ko04926, ko04934, ko04971, ko05012, ko05030, ko05032, ko05034, ko05133, ko05142, ko05145, ko05200, map04015, map04022, map04024, map04062, map04071, map04261, map04360, map04371, map04540, map04611, map04670, map04713, map04723, map04724, map04725, map04726, map04727, map04728, map04730, map04914, map04915, map04916, map04921, map04923, map04924, map04926, map04934, map04971, map05012, map05030, map05032, map05034, map05133, map05142, map05145, map05200
FUN_006996	Acetyltransferase (GNAT) domain	-	-	-	-
FUN_006997	Natural resistance-associated macrophage protein	SMF3	-	ko:K12346	-
FUN_006998	CDC24 Calponin	CDC24	-	ko:K11236	ko04011, map04011
FUN_006999	A Receptor for Ubiquitination Targets	-	-	ko:K03362	ko04114, ko04120, ko04218, ko04310, ko04340, ko04341, ko04390, ko04624, ko04710, ko05131, map04114, map04120, map04218, map04310, map04340, map04341, map04390, map04624, map04710, map05131
FUN_007000	Belongs to the ABC transporter superfamily. ABCG family. PDR (TC 3.A.1.205) subfamily	CDR1	-	ko:K08711, ko:K08712	ko02010, map02010
FUN_007001	Reversible hydration of carbon dioxide	NCE103	4.2.1.1	ko:K01673	ko00910, map00910
FUN_007004	Aldo/keto reductase family	CSH1	-	-	-
FUN_007005	GAL4-like Zn(II)2Cys6 (or C6 zinc) binuclear cluster DNA-binding domain	-	-	-	-
FUN_007006	Major Facilitator Superfamily	-	-	-	-
FUN_007007	Cupin domain	-	-	-	-
FUN_007008	Prenyltransferase and squalene oxidase repeat	RAM1	2.5.1.58	ko:K05954	ko00900, ko01130, map00900, map01130
FUN_007009	Stress responsive A/B Barrel Domain	-	-	-	-
FUN_007011	MobA-like NTP transferase domain	MPG1	2.7.7.13	ko:K00966	ko00051, ko00520, ko01100, ko01110, map00051, map00520, map01100, map01110
FUN_007012	MobA-like NTP transferase domain	MPG1	2.7.7.13	ko:K00966	ko00051, ko00520, ko01100, ko01110, map00051, map00520, map01100, map01110
FUN_007013	Major Facilitator Superfamily	-	-	-	-
FUN_007016	RNA helicase	SPB4	3.6.4.13	ko:K14809	-
FUN_007017	Phosphatidate cytidylyltransferase	DGK1	2.7.1.174	ko:K16368	ko00564, ko01100, ko01110, map00564, map01100, map01110
FUN_007018	Kelch motif	-	-	-	-
FUN_007019	Belongs to the ubiquitin-conjugating enzyme family	ubc15	2.3.2.23	ko:K10575	ko04120, ko04141, ko05012, map04120, map04141, map05012
FUN_007021	Peptidase C1-like family	LAP3	3.4.22.40	ko:K01372	-
FUN_007023	Belongs to the 5'-nucleotidase family	-	-	-	-
FUN_007024	Class II Aldolase and Adducin N-terminal domain	-	-	-	-
FUN_007025	Phosphoglycerate mutase family	-	-	-	-
FUN_007026	Heterokaryon incompatibility protein (HET)	-	-	-	-
FUN_007027	APSES transcription factor Xbp1	-	-	-	-

FUN_007028	Glutaredoxin	-	2.5.1.18	ko:K00799, ko:K03448	ko00480, ko00980, ko00982, ko00983, ko01524, ko05200, ko05204, ko05225, ko05418, map00480, map00980, map00982, map00983, map01524, map05200, map05204, map05225, map05418
FUN_007029	Zinc-finger double-stranded RNA-binding	SNU23	-	ko:K12848	ko03040, map03040
FUN_007032	Homeobox KN domain	-	-	-	-
FUN_007034	Belongs to the thiolase family	-	2.3.1.9	ko:K00626	ko00071, ko00072, ko00280, ko00310, ko00362, ko00380, ko00620, ko00630, ko00640, ko00650, ko00720, ko00900, ko01100, ko01110, ko01120, ko01130, ko01200, ko01212, ko02020, map00071, map00072, map00280, map00310, map00362, map00380, map00620, map00630, map00640, map00650, map00720, map00900, map01100, map01110, map01120, map01130, map01200, map01212, map02020
FUN_007036	Heterokaryon incompatibility protein (HET)	-	-	-	-
FUN_007038	Zinc-binding dehydrogenase	-	-	-	-
FUN_007039	Control of topological states of DNA by transient breakage and subsequent rejoining of DNA strands. Topoisomerase II makes double-strand breaks	TOP2	5.99.1.3	ko:K03164	ko01524, map01524
FUN_007040	Sds3-like	-	-	-	-
FUN_007042	Belongs to the short-chain dehydrogenases reductases (SDR) family	-	-	-	-
FUN_007043	Ribosomal protein L6e	RPL6	-	ko:K02934	ko03010, map03010
FUN_007048	Domain of unknown function (DUF1992)	-	-	-	-
FUN_007049	TFIIH p62 subunit, N-terminal domain	TFB1	-	ko:K03141	ko03022, ko03420, ko05203, map03022, map03420, map05203
FUN_007050	2-oxoglutarate dehydrogenase N-terminus	KGD1	1.2.4.2	ko:K00164	ko00020, ko00310, ko00380, ko01100, ko01110, ko01120, ko01130, ko01200, map00020, map00310, map00380, map01100, map01110, map01120, map01130, map01200
FUN_007052	Alcohol dehydrogenase GroES-like domain	-	-	-	-
FUN_007053	Domain of unknown function (DUF3425)	-	-	-	-
FUN_007054	calmodulin-like	-	-	ko:K13448	ko04626, map04626
FUN_007055	Glycoside hydrolase family 11 protein	-	3.2.1.8	ko:K01181	-
FUN_007058	IBR domain, a half RING-finger domain	-	-	-	-
FUN_007059	GMC oxidoreductase	-	1.1.99.1	ko:K00108	ko00260, ko01100, map00260, map01100
FUN_007060	Belongs to the cytochrome P450 family	-	-	-	-
FUN_007063	AKAP7 2'5' RNA ligase-like domain	-	-	ko:K18666	-
FUN_007064	Histidine Phosphotransfer domain	YPD1	-	ko:K11232	ko02020, ko04011, map02020, map04011
FUN_007065	Aspartate aminotransferase	AAT1	2.6.1.1	ko:K14455	ko00220, ko00250, ko00270, ko00330, ko00350, ko00360, ko00400, ko00710, ko00950, ko00960, ko01100, ko01110, ko01120, ko01130, ko01200, ko01210, ko01230, ko04975, map00220, map00250, map00270, map00330, map00350, map00360, map00400, map00710, map00950, map00960, map01100, map01110, map01120, map01130, map01200, map01210, map01230, map04975
FUN_007066	Genetic interactor of prohibitins 3	GEP3	-	ko:K19829	-
FUN_007067	Glycosyltransferase family 34 protein	MNN11	-	ko:K05532	ko00513, ko01100, map00513, map01100

FUN_007068	Phosphomannose isomerase type I	-	5.3.1.8	ko:K01809	ko00051, ko00520, ko01100, ko01110, ko01130, map00051, map00520, map01100, map01110, map01130
FUN_007070	Enoyl-(Acyl carrier protein) reductase	-	-	-	-
FUN_007073	GCN5-like protein 1 (GCN5L1)	-	-	ko:K20185	-
FUN_007074	Domain of Unknown Function (DUF913)	TOM1	2.3.2.26	ko:K10592	ko04120, map04120
FUN_007076	Belongs to the cytochrome P450 family	-	1.14.14.1, 1.6.2.4	ko:K14338	ko00071, ko00380, ko00627, ko01120, map00071, map00380, map00627, map01120
FUN_007077	Belongs to the short-chain dehydrogenases reductases (SDR) family	-	1.1.1.184, 1.1.1.189, 1.1.1.197	ko:K00079	ko00590, ko00790, ko00980, ko01100, ko05204, map00590, map00790, map00980, map01100, map05204
FUN_007078	Methyltransferase domain	-	-	-	-
FUN_007079	Belongs to the short-chain dehydrogenases reductases (SDR) family	AYR1	1.1.1.101	ko:K06123	ko00564, ko00565, ko01100, map00564, map00565, map01100
FUN_007080	lysine 2, 3-aminomutase activity	-	5.4.3.2	ko:K01843	ko00310, map00310
FUN_007081	GET complex subunit GET2	-	-	ko:K22386	-
FUN_007082	Mitochondrial ribosomal protein L51 / S25 / CI-B8 domain	-	-	ko:K17440	-
FUN_007084	Protein of unknown function (DUF4448)	-	-	-	-
FUN_007086	Peptidase dimerisation domain	-	4.3.1.15	ko:K01751	-
FUN_007087	DSBA-like thioredoxin domain	-	-	-	-
FUN_007089	DUF1741	-	-	-	-
FUN_007090	AAA domain	NRK1	2.7.1.173, 2.7.1.22	ko:K10524	ko00760, ko01100, map00760, map01100
FUN_007091	MRG	EAF3	-	ko:K11339	-
FUN_007093	helix loop helix domain	-	-	-	-
FUN_007095	Belongs to the helicase family. Dicer subfamily	dcl2	-	-	-
FUN_007096	Vacuolar sorting protein 39 domain 1	VAM6	-	ko:K20183	ko04138, map04138
FUN_007097	HbrB-like	-	-	-	-
FUN_007098	Glycoside hydrolase family 5 protein	-	3.2.1.78	ko:K19355	ko00051, map00051
FUN_007099	Frataxin-like domain	YFH1	1.16.3.1	ko:K19054	ko00860, map00860
FUN_007100	CSL zinc finger	DPH3	-	ko:K15455	-
FUN_007101	Exocyst complex component Sec6	SEC6	-	ko:K06110	-
FUN_007103	Common central domain of tyrosinase	-	1.14.18.1	ko:K00505	ko00350, ko00950, ko00965, ko01100, ko01110, ko04916, map00350, map00950, map00965, map01100, map01110, map04916
FUN_007105	trafficking pga2	-	-	-	-
FUN_007106	CENP-S protein	MHF1	-	-	-
FUN_007108	Glucose dehydrogenase C-terminus	-	1.1.1.303, 1.1.1.4	ko:K00004	ko00650, map00650
FUN_007109	Alcohol dehydrogenase GroES-like domain	-	1.1.1.2	ko:K00002	ko00010, ko00040, ko00561, ko00930, ko01100, ko01110, ko01120, ko01130, ko01220, map00010, map00040, map00561, map00930, map01100, map01110, map01120, map01130, map01220
FUN_007110	Ctf8	-	-	ko:K11270	-
FUN_007111	Carbohydrate-binding module family 1 protein	-	-	-	-

FUN_007112	AAA C-terminal domain	MGS1	-	ko:K07478	-
FUN_007113	DegT/DnrJ/EryC1/StrS aminotransferase family	BNA3	2.6.1.7	ko:K14264	ko00380, ko01100, map00380, map01100
FUN_007114	Tetratricopeptide repeat	SSN6	-	ko:K06665	ko04011, ko04111, map04011, map04111
FUN_007116	Beta-glucan synthesis-associated protein (SKN1)	KRE6	-	ko:K21850	-
FUN_007117	Steryl acetyl hydrolase	-	-	-	-
FUN_007118	Steroid receptor RNA activator (SRA1)	SEC31	-	ko:K14005	ko04141, map04141
FUN_007119	Peptidylprolyl isomerase	-	5.2.1.8	ko:K01802, ko:K09564	ko03040, map03040
FUN_007121	This protein is one of the nuclear-coded polypeptide chains of cytochrome c oxidase, the terminal oxidase in mitochondrial electron transport	COX12	-	ko:K02267	ko00190, ko01100, ko04260, ko04714, ko04932, ko05010, ko05012, ko05016, map00190, map01100, map04260, map04714, map04932, map05010, map05012, map05016
FUN_007122	Guanine nucleotide exchange factor for Ras-like small GTPases	-	-	-	-
FUN_007125	impB/mucB/samB family C-terminal domain	-	2.7.7.7	ko:K03510	ko03460, map03460
FUN_007126	Pyridoxal-dependent decarboxylase conserved domain	DPL1	4.1.2.27	ko:K01634	ko00600, ko01100, ko04071, map00600, map01100, map04071
FUN_007127	Belongs to the universal ribosomal protein uL16 family	mrpl16	-	-	-
FUN_007128	Replication factor C subunit 1	rfc1	-	ko:K10754	ko03030, ko03420, ko03430, map03030, map03420, map03430
FUN_007132	Ribosomal protein L30p/L7e	MRPL33	-	ko:K02907	ko03010, map03010
FUN_007134	Essential component of the PAM complex, a complex required for the translocation of transit peptide-containing proteins from the inner membrane into the mitochondrial matrix in an ATP-dependent manner	mge1	-	ko:K03687	-
FUN_007135	RNA polymerases D	rpb3	-	ko:K03011	ko00230, ko00240, ko01100, ko03020, ko05016, ko05169, map00230, map00240, map01100, map03020, map05016, map05169
FUN_007136	Calcipressin	-	-	-	-
FUN_007137	Belongs to the eukaryotic ribosomal protein eS6 family	RPS6	-	ko:K02991	ko01521, ko03010, ko04066, ko04150, ko04151, ko04371, ko04714, ko04910, ko05205, map01521, map03010, map04066, map04150, map04151, map04371, map04714, map04910, map05205
FUN_007138	Phosphoribosyl synthetase-associated domain	PRS4	2.7.6.1	ko:K00948	ko00030, ko00230, ko01100, ko01110, ko01120, ko01130, ko01200, ko01230, map00030, map00230, map01100, map01110, map01120, map01130, map01200, map01230
FUN_007139	Up-regulated During Septation	-	-	-	-
FUN_007140	Ferredoxin-fold anticodon binding domain	MSF1	6.1.1.20	ko:K01889	ko00970, map00970
FUN_007141	Belongs to the mitochondrial carrier (TC 2.A.29) family	DIC1	-	ko:K13577	ko04964, map04964
FUN_007142	WD40 repeats	-	-	ko:K12782	ko04113, map04113
FUN_007143	Protein prenyltransferase alpha subunit repeat	BET4	2.5.1.60	ko:K14050	-
FUN_007144	Mus7/MMS22 family	-	-	-	-
FUN_007147	ATPase family associated with various cellular activities (AAA)	-	-	-	-
FUN_007148	WD domain, G-beta repeat	-	-	-	-
FUN_007149	glycoside hydrolase family 63	-	-	-	-
FUN_007150	Mob1/phocein family	MOB1	-	ko:K06685	ko04111, ko04390, ko04391, ko04392, map04111, map04390, map04391, map04392
FUN_007151	Belongs to the glycosyl hydrolase 5 (cellulase A) family	-	3.2.1.4	ko:K01179	ko00500, ko01100, map00500, map01100
FUN_007152	Homologues of snake disintegrins	ADM-B	-	-	-

FUN_007153	Acetyl-coenzyme A synthetase	ACS2	6.2.1.1	ko:K01895	ko00010, ko00620, ko00640, ko00680, ko00720, ko01100, ko01110, ko01120, ko01130, ko01200, map00010, map00620, map00640, map00680, map00720, map01100, map01110, map01120, map01130, map01200
FUN_007154	Methyltransferase domain	-	-	-	-
FUN_007155	Scaffold protein Nfu/NifU N terminal	-	-	ko:K22074	-
FUN_007160	HMGL-like	-	4.1.3.4	ko:K01640	ko00072, ko00280, ko00281, ko00650, ko01100, ko04146, map00072, map00280, map00281, map00650, map01100, map04146
FUN_007162	GDP dissociation inhibitor	MRS6	-	-	-
FUN_007163	Pyridoxamine 5'-phosphate oxidase	-	-	-	-
FUN_007164	Belongs to the eukaryotic ribosomal protein eS30 family	rps30a	-	ko:K02983	ko03010, map03010
FUN_007165	Protein of unknown function (DUF1687)	-	-	-	-
FUN_007167	CorA-like Mg2+ transporter protein	-	-	-	-
FUN_007168	Belongs to the TRAFAC class dynamin-like GTPase superfamily. Dynamin Fzo YdjA family	-	-	-	-
FUN_007169	Putative zinc finger motif, C2HC5-type	-	-	-	-
FUN_007170	PPIases accelerate the folding of proteins. It catalyzes the cis-trans isomerization of proline imidic peptide bonds in oligopeptides	cyp1	5.2.1.8	ko:K12733	ko03040, map03040
FUN_007171	Alcohol dehydrogenase GroES-like domain	-	-	-	-
FUN_007174	LysM domain	-	-	-	-
FUN_007178	Protein of unknown function (DUF2470)	-	-	-	-
FUN_007179	Belongs to the class-III pyridoxal-phosphate-dependent aminotransferase family	ARG8	2.6.1.11	ko:K00818	ko00220, ko01100, ko01110, ko01130, ko01210, ko01230, map00220, map01100, map01110, map01130, map01210, map01230
FUN_007180	SNF2 family N-terminal domain	-	-	-	-
FUN_007181	HMG-box domain	RFG1	-	ko:K09274, ko:K22496	-
FUN_007182	HMG-box domain	RFG1	-	ko:K09274, ko:K22496	-
FUN_007183	alpha/beta hydrolase fold	-	-	ko:K12389	ko04142, map04142
FUN_007185	Methyltransferase domain	-	-	-	-
FUN_007186	iron ion binding	-	-	-	-
FUN_007187	Alpha/beta hydrolase family	-	-	-	-
FUN_007190	Male sterility protein	LYS2	1.2.1.95	ko:K00143	ko00300, ko01100, ko01110, ko01130, ko01230, map00300, map01100, map01110, map01130, map01230
FUN_007191	Uncharacterized conserved protein (DUF2183)	-	-	-	-
FUN_007192	KR domain	-	-	-	-
FUN_007193	snRNP Sm proteins	-	-	ko:K12620	ko03018, map03018
FUN_007197	Domain of unknown function (DUF1996)	-	-	-	-
FUN_007198	Amidase	-	-	-	-
FUN_007199	Amidase	-	-	-	-
FUN_007201	KR domain	-	-	-	-

FUN_007202	Belongs to the glutamine synthetase family	-	6.3.1.2	ko:K01915	ko00220, ko00250, ko00630, ko00910, ko01100, ko01120, ko01230, ko02020, ko04217, ko04724, ko04727, map00220, map00250, map00630, map00910, map01100, map01120, map01230, map02020, map04217, map04724, map04727
FUN_007203	Belongs to the aldehyde dehydrogenase family	MSC7	-	-	-
FUN_007204	Major Facilitator Superfamily	-	-	-	-
FUN_007205	Epoxide hydrolase N terminus	-	3.3.2.9	ko:K01253	ko00980, ko04976, ko05204, map00980, map04976, map05204
FUN_007207	to NAD-dependent epimerase dehydratase	-	-	-	-
FUN_007208	Belongs to the cytochrome P450 family	-	-	-	-
FUN_007210	Belongs to the malate synthase family	-	2.3.3.9	ko:K01638	ko00620, ko00630, ko01100, ko01110, ko01120, ko01200, map00620, map00630, map01100, map01110, map01120, map01200
FUN_007211	Belongs to the short-chain dehydrogenases reductases (SDR) family	-	-	-	-
FUN_007214	Alpha galactosidase A	-	3.2.1.22	ko:K07407	ko00052, ko00561, ko00600, ko00603, map00052, map00561, map00600, map00603
FUN_007215	Zinc-binding dehydrogenase	-	-	-	-
FUN_007216	Ethanolamine utilisation protein EutQ	-	-	-	-
FUN_007217	ATP synthase (E/31 kDa) subunit	VMA4	-	ko:K02150	ko00190, ko01100, ko04145, ko04150, ko04721, ko04966, ko05110, ko05120, ko05323, map00190, map01100, map04145, map04150, map04721, map04966, map05110, map05120, map05323
FUN_007218	Magnesium transporter NIPA	-	-	-	-
FUN_007219	Heterokaryon incompatibility protein (HET)	-	-	-	-
FUN_007220	Fibronectin type 3 domain	-	-	ko:K19877	-
FUN_007221	Serine aminopeptidase, S33	-	-	-	-
FUN_007222	Protein of unknown function (DUF3632)	-	-	-	-
FUN_007223	Glycosyl transferase family group 2	-	-	-	-
FUN_007224	Glycoside hydrolase family 16 protein	-	-	-	-
FUN_007225	Belongs to the peroxisomal membrane protein PXMP2 4 family	-	-	-	-
FUN_007226	Putative serine dehydratase domain	-	-	-	-
FUN_007227	Glycosyl hydrolase	-	-	-	-
FUN_007228	Dienelactone hydrolase family	-	-	-	-
FUN_007229	FAD binding domain	-	-	-	-
FUN_007230	Acetyltransferase (GNAT) domain	-	-	-	-
FUN_007232	Domain of unknown function (DUF1996)	-	-	-	-
FUN_007233	Belongs to the cytochrome P450 family	-	-	-	-
FUN_007237	Taurine catabolism dioxygenase TauD, TfdA family	-	-	-	-
FUN_007238	Domain of unknown function (DUF1965)	-	1.4.3.21	ko:K00276	ko00260, ko00350, ko00360, ko00410, ko00950, ko00960, ko01100, ko01110, map00260, map00350, map00360, map00410, map00950, map00960, map01100, map01110
FUN_007239	Short-chain dehydrogenase reductase sdr	-	-	-	-
FUN_007241	Alpha galactosidase A	aglD	3.2.1.22	ko:K07407	ko00052, ko00561, ko00600, ko00603, map00052, map00561, map00600, map00603
FUN_007242	short-chain dehydrogenase reductase	-	-	-	-
FUN_007243	Kelch motif	-	-	-	-

FUN_007245	Ferric reductase NAD binding domain	FRP1	-	-	-
FUN_007246	Ctr copper transporter family	-	-	-	-
FUN_007248	Nascent polypeptide-associated complex subunit beta	EGD1	-	ko:K01527	ko04214, map04214
FUN_007249	Pyridine nucleotide-disulphide oxidoreductase	-	-	-	-
FUN_007250	Belongs to the FMO family	FMO1	-	-	-
FUN_007251	Major Facilitator Superfamily	-	-	ko:K03448	-
FUN_007252	Belongs to the metallo-dependent hydrolases superfamily. Peptidase M19 family	-	-	-	-
FUN_007253	3' exoribonuclease family, domain 1	-	-	ko:K12586	ko03018, map03018
FUN_007254	Serine/Threonine protein kinases, catalytic domain	KIN3	2.7.11.1	ko:K20872	-
FUN_007255	Broad-Complex, Tramtrack and Bric a brac	-	-	ko:K06867	-
FUN_007256	Dienelactone hydrolase family	-	-	-	-
FUN_007257	Fungal specific transcription factor domain	-	-	-	-
FUN_007258	Phospholipase/Carboxylesterase	-	-	-	-
FUN_007259	helicase superfamily c-terminal domain	SUB2	3.6.4.13	ko:K12812	ko03013, ko03015, ko03040, ko05164, map03013, map03015, map03040, map05164
FUN_007260	Ketopantoate reductase PanE/ApbA C terminal	-	-	-	-
FUN_007261	Belongs to the hexokinase family	GLK1	2.7.1.1	ko:K00844	ko00010, ko00051, ko00052, ko00500, ko00520, ko00521, ko00524, ko01100, ko01110, ko01120, ko01130, ko01200, ko04066, ko04910, ko04930, ko04973, ko05230, map00010, map00051, map00052, map00500, map00520, map00521, map00524, map01100, map01110, map01120, map01130, map01200, map04066, map04910, map04930, map04973, map05230
FUN_007263	zinc finger	-	-	-	-
FUN_007264	Phosphoglycerate mutase family	SHB17	3.1.3.37	ko:K22315	-
FUN_007266	Acetyltransferase (GNAT) family	-	-	-	-
FUN_007267	Carbohydrate-binding module family 1 protein	-	-	-	-
FUN_007268	Acetyltransferase (GNAT) family	-	2.3.1.258	ko:K20793	-
FUN_007269	Protein of Unknown function (DUF1690)	-	-	ko:K17788	-
FUN_007270	Belongs to the class I-like SAM-binding methyltransferase superfamily. C5-methyltransferase family	-	2.1.1.37	ko:K00558	ko00270, ko01100, ko05206, map00270, map01100, map05206
FUN_007273	Signal recognition particle, alpha subunit, N-terminal	SRP101	-	ko:K13431, ko:K21989	ko03060, map03060
FUN_007274	YIF1	hrf1	-	ko:K20362	-
FUN_007275	Cyclin	PCL5	-	ko:K21197	ko04138, map04138
FUN_007277	Phosphatidylinositol-4-phosphate 5-Kinase	-	-	-	-
FUN_007279	CorA-like Mg2+ transporter protein	MRS2	-	ko:K16075	-
FUN_007280	belongs to the acetylglutamate kinase family	6	1.2.1.38, 2.7.2.8	ko:K12659	ko00220, ko01100, ko01110, ko01130, ko01210, ko01230, map00220, map01100, map01110, map01130, map01210, map01230
FUN_007281	Synaptobrevin	SNC2	-	ko:K08513	ko04130, map04130
FUN_007282	Glycoside hydrolase family 76 protein	-	3.2.1.101	ko:K08257	-
FUN_007283	Peptidase dimerisation domain	-	3.5.1.16	ko:K01438	ko00220, ko01100, ko01110, ko01130, ko01210, ko01230, map00220, map01100, map01110, map01130, map01210, map01230

FUN_007284	belongs to the purine pyrimidine phosphoribosyltransferase family	ADE4	2.4.2.14	ko:K00764	ko00230, ko00250, ko01100, ko01110, ko01130, map00230, map00250, map01100, map01110, map01130
FUN_007288	Rgp1	RGP1	-	ko:K20477	-
FUN_007291	FAD-binding domain	-	-	-	-
FUN_007293	WH1 domain	-	-	ko:K05747	ko04062, ko04144, ko04520, ko04530, ko04666, ko04810, ko05100, ko05130, ko05131, ko05132, ko05231, map04062, map04144, map04520, map04530, map04666, map04810, map05100, map05130, map05131, map05132, map05231
FUN_007296	AMP-binding enzyme	-	-	-	-
FUN_007298	MmgE/PrpD family	PDH1	4.2.1.79	ko:K01720	ko00640, map00640
FUN_007299	Functions as actin-binding component of the Arp2 3 complex which is involved in regulation of actin polymerization and together with an activating nucleation-promoting factor (NPF) mediates the formation of branched actin networks. Seems to contact the mother actin filament	ARC19	-	ko:K05755	ko04144, ko04666, ko04810, ko05100, ko05130, ko05131, ko05132, map04144, map04666, map04810, map05100, map05130, map05131, map05132
FUN_007300	Belongs to the peptidase C19 family	UBP6	3.4.19.12	ko:K11843	-
FUN_007301	N terminus of Rad21 / Rec8 like protein	rec8	-	ko:K12780, ko:K13054	ko04113, ko04114, map04113, map04114
FUN_007302	Fungal protein of unknown function (DUF1752)	GAT1	-	ko:K09184	-
FUN_007303	DJ-1/PfpI family	-	3.5.1.124	ko:K05687	ko05012, map05012
FUN_007304	Fungal trichothecene efflux pump (TRI12)	-	-	-	-
FUN_007306	GDSL-like Lipase/Acylhydrolase family	-	-	-	-
FUN_007307	Sugar (and other) transporter	-	-	-	-
FUN_007309	Bacterial low temperature requirement A protein (LtrA)	-	-	-	-
FUN_007310	guanyl-nucleotide exchange factor activity	-	-	-	-
FUN_007311	Domain in Tre-2, BUB2p, and Cdc16p. Probable Rab-GAPs.	-	-	-	-
FUN_007312	K homology RNA-binding domain	-	-	ko:K13210	-
FUN_007313	Aldehyde oxidase and xanthine dehydrogenase, a/b hammerhead domain	-	1.17.1.4, 1.17.3.2	ko:K00106	ko00230, ko00232, ko00983, ko01100, ko01110, ko01120, ko04146, map00230, map00232, map00983, map01100, map01110, map01120, map04146
FUN_007314	Phospholipase/Carboxylesterase	-	3.1.1.5	ko:K06128, ko:K06130, ko:K06999	ko00564, ko05231, map00564, map05231
FUN_007315	endonuclease III	-	4.2.99.18	ko:K01741	-
FUN_007317	Belongs to the oxygen-dependent FAD-linked oxidoreductase family	-	-	-	-
FUN_007318	cfem domain-containing protein	-	-	-	-
FUN_007320	S-adenosyl-L-methionine-dependent protein-arginine N- methyltransferase that methylates the delta-nitrogen atom of arginine residues to form N5-methylarginine (type IV) in target proteins. Monomethylates ribosomal protein L12	RMT2	2.1.1.322	ko:K18477	-
FUN_007322	breast cancer carboxy-terminal domain	-	-	-	-
FUN_007324	DnaJ molecular chaperone homology domain	-	-	ko:K05516	-
FUN_007326	NAD(P)H-binding	-	-	-	-
FUN_007330	phosphatidylglycerol acyl-chain remodeling	-	-	-	-
FUN_007331	Transferrin receptor-like dimerisation domain	-	3.4.17.21	ko:K01301	-
FUN_007332	Growth-Arrest-Specific Protein 2 Domain	-	-	-	-

FUN_007333	Belongs to the major facilitator superfamily. Sugar transporter (TC 2.A.1.1) family	-	-	-	-
FUN_007334	Chromatin organization modifier domain	NEW1	-	ko:K03235	-
FUN_007335	Glycoside hydrolase family 3 protein	bglE	3.2.1.21	ko:K05349	ko00460, ko00500, ko00940, ko01100, ko01110, map00460, map00500, map00940, map01100, map01110
FUN_007336	Belongs to the citrate synthase family	CIT3	2.3.3.1	ko:K01647	ko00020, ko00630, ko01100, ko01110, ko01120, ko01130, ko01200, ko01210, ko01230, map00020, map00630, map01100, map01110, map01120, map01130, map01200, map01210, map01230
FUN_007337	Belongs to the isocitrate lyase PEP mutase superfamily. Isocitrate lyase family	ICL2	4.1.3.1	ko:K01637	ko00630, ko01100, ko01110, ko01120, ko01200, map00630, map01100, map01110, map01120, map01200
FUN_007338	Microsomal epoxide hydrolase	-	-	-	-
FUN_007339	AAA domain	SMC5	-	-	-
FUN_007340	Fumarase C C-terminus	FUM1	4.2.1.2	ko:K01679	ko00020, ko00620, ko00720, ko01100, ko01110, ko01120, ko01130, ko01200, ko04934, ko05200, ko05211, map00020, map00620, map00720, map01100, map01110, map01120, map01130, map01200, map04934, map05200, map05211
FUN_007341	Anaphase-promoting complex subunit 11 RING-H2 finger	SAN1	-	-	-
FUN_007342	Extension to Ser/Thr-type protein kinases	DBF2	-	ko:K06684, ko:K06686	ko04111, ko04392, map04111, map04392
FUN_007345	GAL4-like Zn(II)2Cys6 (or C6 zinc) binuclear cluster DNA-binding domain	-	-	-	-
FUN_007346	AMP-binding enzyme C-terminal domain	-	-	-	-
FUN_007348	WW domain binding protein 11	-	-	-	-
FUN_007349	Heat shock protein 9/12	-	-	-	-
FUN_007350	Glycoside hydrolase family 76 protein	-	-	-	-
FUN_007351	Four repeated domains in the Fasciclin I family of proteins, present in many other contexts.	-	-	ko:K05747	ko04062, ko04144, ko04520, ko04530, ko04666, ko04810, ko05100, ko05130, ko05131, ko05132, ko05231, map04062, map04144, map04520, map04530, map04666, map04810, map05100, map05130, map05131, map05132, map05231
FUN_007352	Belongs to the group II decarboxylase family	GAD1	4.1.1.15	ko:K01580	ko00250, ko00410, ko00430, ko00650, ko01100, ko01110, ko01120, ko02024, ko04727, ko04940, map00250, map00410, map00430, map00650, map01100, map01110, map01120, map02024, map04727, map04940
FUN_007353	FAD binding domain of DNA photolyase	-	4.1.99.3	ko:K01669	-
FUN_007355	NUDIX domain	-	3.6.1.13	ko:K01515	ko00230, map00230
FUN_007357	Redoxin	-	1.11.1.15	ko:K14171	ko04122, map04122
FUN_007358	Protein of unknown function (DUF1348)	-	-	ko:K09958	-
FUN_007359	Roadblock/LC7 domain	-	-	ko:K10419	-
FUN_007360	Protein of unknown function DUF262	-	-	-	-
FUN_007361	Belongs to the short-chain dehydrogenases reductases (SDR) family	-	1.1.1.300	ko:K11153	ko00830, ko01100, map00830, map01100
FUN_007362	Belongs to the histidine acid phosphatase family	-	3.1.3.2	ko:K01078	ko00730, ko00740, ko01100, map00730, map00740, map01100
FUN_007363	FMN-dependent dehydrogenase	-	1.13.12.4	ko:K00467	ko00620, map00620
FUN_007364	His Kinase A (phosphoacceptor) domain	-	-	-	-
FUN_007365	Domain of unknown function (DUF1989)	-	-	-	-

FUN_007366	Belongs to the short-chain dehydrogenases reductases (SDR) family	-	-	-	-
FUN_007369	ribosomal protein	RPL24	-	ko:K02896	ko03010, map03010
FUN_007373	Interferon-induced 6-16 family	-	-	-	-
FUN_007374	Mediates the uptake of pyruvate into mitochondria	-	-	ko:K22139	-
FUN_007376	Fungal kinase associated-1 domain	GIN4	2.7.11.1	ko:K02515, ko:K06668, ko:K18679, ko:K18680	ko04011, ko04111, map04011, map04111
FUN_007378	Glutathione-dependent formaldehyde-activating	-	-	-	-
FUN_007380	Transient receptor potential (TRP) ion channel	-	-	-	-
FUN_007381	Argininosuccinate lyase C-terminal	ARG4	4.3.2.1	ko:K01755	ko00220, ko00250, ko01100, ko01110, ko01130, ko01230, map00220, map00250, map01100, map01110, map01130, map01230
FUN_007383	Belongs to the ubiquitin-conjugating enzyme family	-	2.3.2.23	ko:K10580	ko04120, ko04624, map04120, map04624
FUN_007384	Zinc-ribbon, C4HC2 type	-	-	ko:K20409	ko04150, map04150
FUN_007385	Amino acid permease	HNM1	-	ko:K19564	-
FUN_007386	SRF-type transcription factor (DNA-binding and dimerisation domain)	MCM1	-	ko:K12412	ko04011, ko04111, map04011, map04111
FUN_007387	tetraspanin	-	-	-	-
FUN_007389	Belongs to the cation transport ATPase (P-type) (TC 3.A.3) family. Type IV subfamily	DNF1	3.6.1.3, 3.6.3.1, 3.6.3.8	ko:K01509, ko:K01530, ko:K01537	ko00230, ko04742, map00230, map04742
FUN_007391	Predicted AdoMet-dependent methyltransferase	TRM44	2.1.1.211	ko:K15447	-
FUN_007392	Predicted AdoMet-dependent methyltransferase	TRM44	2.1.1.211	ko:K15447	-
FUN_007395	Peptidase family M20/M25/M40	-	-	-	-
FUN_007397	Ras-binding domain of Byr2	STE11	2.7.11.25	ko:K11228	ko04011, map04011
FUN_007398	Alcohol dehydrogenase GroES-like domain	-	1.1.1.1	ko:K13953	ko00010, ko00071, ko00350, ko00625, ko00626, ko00830, ko00980, ko00982, ko01100, ko01110, ko01120, ko01130, ko01220, map00010, map00071, map00350, map00625, map00626, map00830, map00980, map00982, map01100, map01110, map01120, map01130, map01220
FUN_007399	DNA polymerase III, delta subunit	RFC5	-	ko:K10756	ko03030, ko03420, ko03430, map03030, map03420, map03430
FUN_007401	Telomere stability and silencing	-	-	-	-
FUN_007402	Plectin/S10 domain	-	-	ko:K02947	ko03010, map03010
FUN_007403	Belongs to the peroxidase family	-	-	-	-
FUN_007404	Leucine-rich repeats, outliers	RAD7	-	ko:K15082	-
FUN_007405	Stc1 domain	-	-	-	-
FUN_007406	AARP2CN (NUC121) domain	TSR1	-	ko:K14799	-
FUN_007407	BING4CT (NUC141) domain	utp7	-	ko:K14768	-
FUN_007408	YeeE YedE family integral membrane protein	-	-	ko:K07112	-
FUN_007409	Methyltransferase domain	-	-	-	-
FUN_007410	Methyltransferase TYW3	-	2.1.1.282	ko:K15450	-
FUN_007411	Conserved hypothetical protein (DUF2461)	-	-	-	-
FUN_007412	GAL4-like Zn(II)2Cys6 (or C6 zinc) binuclear cluster DNA-binding domain	-	-	-	-

FUN_007415	Seems to be required for maximal rate of protein biosynthesis. Enhances ribosome dissociation into subunits and stabilizes the binding of the initiator Met-tRNA(I) to 40 S ribosomal subunits	TIF11	-	ko:K03236	ko03013, map03013
FUN_007416	ERCC4 domain	rad16	-	ko:K10848	ko03420, ko03460, map03420, map03460
FUN_007417	CBP4	cbp4	-	-	-
FUN_007418	Sel1-like repeats.	-	-	-	-
FUN_007421	Presenilin, signal peptide peptidase, family	-	-	ko:K09595	-
FUN_007422	Lactoylglutathione lyase	-	-	ko:K07032	-
FUN_007423	Belongs to the peptidase A1 family	-	-	-	-
FUN_007426	SNARE associated Golgi protein	-	-	-	-
FUN_007427	cytochrome P450	-	-	-	-
FUN_007429	Fungal specific transcription factor domain	-	-	-	-
FUN_007431	Catalyzes the hydrolysis of cutin, a polyester that forms the structure of plant cuticle	-	3.1.1.74	ko:K08095	-
FUN_007432	Protein-L-isoaspartate(D-aspartate) O-methyltransferase (PCMT)	-	-	-	-
FUN_007433	HET domain-containing protein	-	-	-	-
FUN_007434	Putative diphthamide synthesis protein	DPH1	2.5.1.108	ko:K07561	-
FUN_007436	Belongs to the eukaryotic ribosomal protein eS8 family	RPS8A	-	ko:K02995	ko03010, map03010
FUN_007438	ATP synthase delta (OSCP) subunit	ATP5	-	ko:K02137	ko00190, ko01100, ko04714, ko05010, ko05012, ko05016, map00190, map01100, map04714, map05010, map05012, map05016
FUN_007439	CLN3 protein	BTN1	-	ko:K12389	ko04142, map04142
FUN_007440	CLN3 protein	BTN1	-	ko:K12389	ko04142, map04142
FUN_007441	Histone deacetylation protein Rxt3	-	-	-	-
FUN_007442	6-phosphofructo-2-kinase	FBP26	2.7.1.105, 3.1.3.46	ko:K19029	ko00051, ko04152, ko04919, map00051, map04152, map04919
FUN_007445	Mitochondrial branched-chain alpha-ketoacid dehydrogenase kinase	PDK2	2.7.11.2	ko:K00898	-
FUN_007446	Electron carrier protein. The oxidized form of the cytochrome c heme group can accept an electron from the heme group of the cytochrome c1 subunit of cytochrome reductase. Cytochrome c then transfers this electron to the cytochrome oxidase complex, the final protein carrier in the mitochondrial electron-transport chain	CYC1	-	ko:K08738	ko00920, ko01100, ko01120, ko01524, ko02020, ko04115, ko04210, ko04214, ko04215, ko04932, ko05010, ko05012, ko05014, ko05016, ko05134, ko05145, ko05152, ko05161, ko05164, ko05167, ko05168, ko05200, ko05210, ko05222, ko05416, map00920, map01100, map01120, map01524, map02020, map04115, map04210, map04214, map04215, map04932, map05010, map05012, map05014, map05016, map05134, map05145, map05152, map05161, map05164, map05167, map05168, map05200, map05210, map05222, map05416
FUN_007447	short chain dehydrogenase	-	-	-	-
FUN_007448	Uncharacterized protein conserved in bacteria (DUF2236)	-	-	-	-
FUN_007449	Required for respiratory activity and maintenance and expression of the mitochondrial genome	RRG9	-	-	-
FUN_007450	Heterokaryon incompatibility protein (HET)	-	-	-	-
FUN_007452	DUF2407 C-terminal domain	-	-	-	-
FUN_007453	Iron-sulphur cluster biosynthesis	ISA1	-	ko:K22063	-
FUN_007454	Belongs to the arginase family	-	3.5.3.11	ko:K01480	ko00330, ko01100, map00330, map01100
FUN_007455	MatE	ERC1	-	ko:K03327	-

FUN_007457	n-acetylglucosamine-6-phosphate deacetylase	-	3.5.1.25	ko:K01443	ko00520, ko01130, map00520, map01130
FUN_007458	Protein kinase C conserved region 2 (CalB)	-	-	-	-
FUN_007459	Fungal specific transcription factor domain	-	-	-	-
FUN_007460	UV-endonuclease UvdE	-	-	ko:K13281	-
FUN_007461	Heterokaryon incompatibility protein (HET)	-	-	-	-
FUN_007463	Flavin containing amine oxidoreductase	-	-	-	-
FUN_007464	Ankyrin repeat	-	-	-	-
FUN_007465	Vesicle transport through interaction with t-SNAREs 1	vti1	-	ko:K08493	ko04130, ko04138, map04130, map04138
FUN_007466	integral membrane protein	-	-	-	-
FUN_007467	endonuclease exonuclease phosphatase	-	-	-	-
FUN_007468	PAPA-1-like conserved region	-	-	ko:K11676	-
FUN_007469	Protein of unknown function (DUF726)	-	-	-	-
FUN_007470	Protein tyrosine kinase	-	-	-	-
FUN_007471	structural constituent of cell wall	-	-	-	-
FUN_007472	Heterokaryon incompatibility protein (HET)	-	-	-	-
FUN_007473	Protein of unknown function (DUF4050)	-	-	-	-
FUN_007478	Pyridine nucleotide-disulphide oxidoreductase	-	-	-	-
FUN_007479	Glu-tRNAGln amidotransferase C subunit	-	-	-	-
FUN_007480	Soluble NSF attachment protein, SNAP	SEC17	-	ko:K15296	ko04138, ko04721, map04138, map04721
FUN_007481	Protein of unknown function (DUF726)	-	-	-	-
FUN_007483	Mitochondrial membrane ATP synthase (F(1)F(0) ATP synthase or Complex V) produces ATP from ADP in the presence of a proton gradient across the membrane which is generated by electron transport complexes of the respiratory chain. F-type ATPases consist of two structural domains, F(1) - containing the extramembraneous catalytic core, and F(0) - containing the membrane proton channel, linked together by a central stalk and a peripheral stalk. During catalysis, ATP synthesis in the catalytic domain of F(1) is coupled via a rotary mechanism of the central stalk subunits to proton translocation. Part of the complex F(0) domain and the peripheric stalk, which acts as a stator to hold the catalytic alpha(3)beta(3) subcomplex and subunit a ATP6 static	ATP7	-	ko:K02138	ko00190, ko01100, ko04714, ko05010, ko05012, ko05016, map00190, map01100, map04714, map05010, map05012, map05016
FUN_007484	NAC domain	EGD2	-	ko:K03626	-
FUN_007485	Ribosomal protein L14	-	-	ko:K02875	ko03010, map03010
FUN_007486	Ribosomal protein l14	-	-	ko:K02875	ko03010, map03010
FUN_007488	Belongs to the small GTPase superfamily. SAR1 family	SAR1	-	ko:K07953	ko04141, ko05134, map04141, map05134
FUN_007494	Sodium-phosphate symporter which plays a fundamental housekeeping role in phosphate transport	-	-	ko:K14640	-
FUN_007497	Major Facilitator Superfamily	-	-	-	-
FUN_007499	NmrA-like family	-	-	-	-
FUN_007502	Sugar (and other) transporter	-	-	-	-
FUN_007503	polyketide synthase	-	-	-	-
FUN_007504	Ubiquitin 3 binding protein But2 C-terminal domain	-	-	-	-
FUN_007506	NAD(P)H-binding	-	-	-	-
FUN_007512	Transporter	-	-	-	-
FUN_007513	Luciferase-like monooxygenase	-	-	-	-

FUN_007514	Fungal specific transcription factor domain	-	-	-	-
FUN_007515	Permease for cytosine/purines, uracil, thiamine, allantoin	-	-	ko:K03457	-
FUN_007516	5-amino-1-ribofuranosylimidazole-4-carboxamide transmembrane transporter activity	-	-	ko:K03457	-
FUN_007517	Luciferase-like monooxygenase	-	-	-	-
FUN_007518	Luciferase-like monooxygenase	-	-	-	-
FUN_007519	Luciferase-like monooxygenase	-	-	-	-
FUN_007520	Alcohol dehydrogenase GroES-like domain	-	-	-	-
FUN_007522	Protein of unknown function (DUF1214)	-	-	-	-
FUN_007523	acyl-coa dehydrogenase	-	-	-	-
FUN_007524	Amidohydrolase family	-	-	-	-
FUN_007525	short chain dehydrogenase	-	-	-	-
FUN_007527	Domain in Tre-2, BUB2p, and Cdc16p. Probable Rab-GAPs.	GYP7	-	ko:K20168	ko04137, map04137
FUN_007528	Sec1-binding region of Mso1	-	-	-	-
FUN_007529	CAS/CSE protein, C-terminus	CSE1	-	ko:K18423	-
FUN_007530	Domain of unknown function (DUF3384)	TSC2	-	-	-
FUN_007532	Threonyl and Alanyl tRNA synthetase second additional domain	-	-	-	-
FUN_007534	Fungal protein of unknown function (DUF2011)	-	-	-	-
FUN_007535	Helicase associated domain (HA2) Add an annotation	ECM16	3.6.4.13	ko:K14780	-
FUN_007536	Proteasome assembly chaperone 3	-	-	ko:K11877	-
FUN_007537	Hsp70 nucleotide exchange factor FES1	FES1	-	-	-
FUN_007538	Belongs to the actin family	ACT1	-	ko:K05692	ko04015, ko04145, ko04210, ko04390, ko04391, ko04510, ko04520, ko04530, ko04611, ko04670, ko04714, ko04745, ko04810, ko04919, ko04921, ko05100, ko05110, ko05130, ko05131, ko05132, ko05164, ko05205, ko05225, ko05410, ko05412, ko05414, ko05416, ko05418, map04015, map04145, map04210, map04390, map04391, map04510, map04520, map04530, map04611, map04670, map04714, map04745, map04810, map04919, map04921, map05100, map05110, map05130, map05131, map05132, map05164, map05205, map05225, map05410, map05412, map05414, map05416, map05418
FUN_007539	Major Facilitator Superfamily	-	-	-	-
FUN_007540	Sugar (and other) transporter	-	-	-	-
FUN_007542	Belongs to the glycosyl hydrolase 5 (cellulase A) family	-	3.2.1.4	ko:K01179	ko00500, ko01100, map00500, map01100
FUN_007543	hemK methyltransferase family member 1	-	2.1.1.297	ko:K02493	-
FUN_007544	Heterokaryon incompatibility protein (HET)	-	-	-	-
FUN_007545	NACHT domain	-	-	-	-
FUN_007546	NACHT domain	-	-	-	-
FUN_007549	Belongs to the class-I aminoacyl-tRNA synthetase family	-	6.1.1.4	ko:K01869	ko00970, map00970
FUN_007550	Belongs to the class-I aminoacyl-tRNA synthetase family	-	6.1.1.4	ko:K01869	ko00970, map00970
FUN_007551	Amino acid permease	-	-	-	-
FUN_007554	Aminotransferase class-V	SER1	2.6.1.52	ko:K00831	ko00260, ko00680, ko00750, ko01100, ko01120, ko01130, ko01200, ko01230, map00260, map00680, map00750, map01100, map01120, map01130, map01200, map01230

FUN_007555	Catalyzes the reversible hydration of cis-homoaconitate to (2R, 3S)-homoisocitrate, a step in the alpha-aminoadipate pathway for lysine biosynthesis	LYS4	4.2.1.36	ko:K01705	ko00300, ko01100, ko01120, ko01130, ko01210, ko01230, map00300, map01100, map01120, map01130, map01210, map01230
FUN_007557	glycoside hydrolase family 3 protein	-	3.2.1.37	ko:K15920	ko00520, ko01100, map00520, map01100
FUN_007558	Beta-lactamase	-	-	-	-
FUN_007560	Belongs to the cytochrome P450 family	-	-	-	-
FUN_007561	Amino acid permease	-	-	-	-
FUN_007563	Phytanoyl-CoA dioxygenase (PhyH)	-	-	-	-
FUN_007564	X-Pro dipeptidyl-peptidase C-terminal non-catalytic domain	-	-	ko:K06978	-
FUN_007565	Major facilitator superfamily transporter	-	-	-	-
FUN_007567	Protein of unknown function (DUF1640)	-	-	-	-
FUN_007568	Enoyl-CoA hydratase/isomerase	ECI1	5.3.3.8	ko:K13239	ko00071, ko04146, map00071, map04146
FUN_007569	Transcriptional regulator	-	-	-	-
FUN_007570	HEAT repeat protein	-	-	-	-
FUN_007571	FAD binding domain-containing protein	-	-	-	-
FUN_007573	Sedoheptulose-1, 7-bisphosphatase	-	3.1.3.37	ko:K01100	ko00710, ko01100, ko01120, ko01200, map00710, map01100, map01120, map01200
FUN_007575	preribosome binding	-	-	ko:K14571	ko03008, map03008
FUN_007576	This magnesium-dependent enzyme catalyzes the hydrolysis of ATP coupled with the transport of calcium	PMC1	3.6.3.8	ko:K01537	-
FUN_007577	Spindle pole body component	alp4	-	ko:K16569	-
FUN_007579	KR domain	-	-	-	-
FUN_007581	Proteasome subunit alpha type	PUP2	3.4.25.1	ko:K02729	ko03050, map03050
FUN_007582	Ubiquinol-cytochrome-c reductase complex subunit (QCR10)	-	-	ko:K00420	ko00190, ko01100, ko04260, ko04714, ko04932, ko05010, ko05012, ko05016, map00190, map01100, map04260, map04714, map04932, map05010, map05012, map05016
FUN_007583	Belongs to the cytochrome P450 family	-	1.14.13.174	ko:K17645	ko00254, map00254
FUN_007584	Major Facilitator Superfamily	-	-	-	-
FUN_007585	Kelch motif	-	-	-	-
FUN_007586	Belongs to the glycosyl hydrolase 17 family	-	3.2.1.39	ko:K01199	ko00500, map00500
FUN_007588	plasma membrane ATPase	PMA1	3.6.3.6	ko:K01535	ko00190, map00190
FUN_007589	Aldo/keto reductase family	-	1.1.1.358	ko:K19654	-
FUN_007590	proline-rich domain in spliceosome associated proteins	sap145	-	ko:K12829	ko03040, map03040
FUN_007591	Serine arginine repetitive matrix protein 1	-	-	-	-
FUN_007592	NmrA-like family	-	-	-	-
FUN_007593	DNA-dependent RNA polymerase catalyzes the transcription of DNA into RNA using the four ribonucleoside triphosphates as substrates	rpc2	2.7.7.6	ko:K03021	ko00230, ko00240, ko01100, ko03020, ko04623, ko05169, map00230, map00240, map01100, map03020, map04623, map05169
FUN_007595	GPI-Mannosyltransferase II co-activator	-	-	-	-
FUN_007596	Tubulin is the major constituent of microtubules. It binds two moles of GTP, one at an exchangeable site on the beta chain and one at a non-exchangeable site on the alpha chain	TUB1	-	ko:K07374	ko04145, ko04210, ko04530, ko04540, ko05130, map04145, map04210, map04530, map04540, map05130
FUN_007597	Minichromosome loss protein, Mcl1, middle region	mcl1	-	ko:K11274	-
FUN_007598	Histone chaperone domain CHZ	chz1	-	-	-

FUN_007599	GXGXG motif	GLT1	1.4.1.13, 1.4.1.14	ko:K00264	ko00250, ko00910, ko01100, ko01110, ko01120, ko01130, ko01230, map00250, map00910, map01100, map01110, map01120, map01130, map01230
FUN_007600	RNA12 protein	YME2	-	-	-
FUN_007601	SNARE complex subunit	BET1	-	ko:K08504	ko04130, map04130
FUN_007602	Catalyzes the reversible transfer of the terminal phosphate group between ATP and AMP. Plays an important role in cellular energy homeostasis and in adenine nucleotide metabolism. Adenylate kinase activity is critical for regulation of the phosphate utilization and the AMP de novo biosynthesis pathways	ADK1	2.7.4.3	ko:K00939	ko00230, ko00730, ko01100, ko01110, ko01130, map00230, map00730, map01100, map01110, map01130
FUN_007603	RPR	PCF11	-	ko:K07478, ko:K14400	ko03015, map03015
FUN_007604	TFIIS helical bundle-like domain	IWS1	-	ko:K17498	-
FUN_007605	Glycoside hydrolase family 55 protein	-	-	-	-
FUN_007606	Nucleoporin complex subunit 54	NUP57	-	ko:K14308	ko03013, map03013
FUN_007607	Accessory subunit of the mitochondrial membrane respiratory chain NADH dehydrogenase (Complex I), that is believed not to be involved in catalysis. Complex I functions in the transfer of electrons from NADH to the respiratory chain. The immediate electron acceptor for the enzyme is believed to be ubiquinone	NdufA8	-	ko:K03952	ko00190, ko01100, ko04714, ko04723, ko04932, ko05010, ko05012, ko05016, map00190, map01100, map04714, map04723, map04932, map05010, map05012, map05016
FUN_007609	Apc15p protein	-	-	-	-
FUN_007610	Cytochrome c oxidase subunit IV	cox5	-	ko:K02263	ko00190, ko01100, ko04260, ko04714, ko04932, ko05010, ko05012, ko05016, map00190, map01100, map04260, map04714, map04932, map05010, map05012, map05016
FUN_007611	Glyceraldehyde-3-phosphate dehydrogenase	GPD	1.2.1.12	ko:K00134	ko00010, ko00710, ko01100, ko01110, ko01120, ko01130, ko01200, ko01230, ko04066, ko05010, map00010, map00710, map01100, map01110, map01120, map01130, map01200, map01230, map04066, map05010
FUN_007612	histone deacetylase	HDA1	3.5.1.98	ko:K11407	ko05034, ko05165, ko05203, map05034, map05165, map05203
FUN_007613	Cyclin	PHO80	-	ko:K06654	ko04111, map04111
FUN_007615	Armadillo/beta-catenin-like repeats	-	-	-	-
FUN_007616	Produces ATP from ADP in the presence of a proton gradient across the membrane	ATP2	2.4.1.18, 3.6.3.14	ko:K00700, ko:K02133	ko00190, ko00500, ko01100, ko01110, ko04714, ko05010, ko05012, ko05016, map00190, map00500, map01100, map01110, map04714, map05010, map05012, map05016
FUN_007617	Glycoside hydrolase family 13 protein	GLC3	2.4.1.18	ko:K00700	ko00500, ko01100, ko01110, map00500, map01100, map01110
FUN_007618	C-terminal, D2-small domain, of ClpB protein	MCX1	-	ko:K03544	ko04112, map04112

Table S3 Predicted pathogen-host interaction genes in E00680

Gene ID	Hit Protein	Similarity	Hit length	Function	Pathogen species	Host group	Host species
FUN_000200	G4N6P9	92.6%	310	CMGC/CK2 protein kinase catalytic subunit	*Magnaporthe oryzae*	monocots	*Oryza sativa*
FUN_000887	G4MU71	91.7%	303	vacuolar protein sorting-associated protein 26B-B	*Magnaporthe oryzae*	monocots	*Oryza sativa*
FUN_000887	G4MU71	91.7%	303	vacuolar protein sorting-associated protein 26B-B	*Magnaporthe oryzae*	monocots	*Hordeum vulgare*
FUN_000887	I1RC52	92.9%	296	components of retromer	*Fusarium graminearum*	monocots	*Triticum aestivum*
FUN_000887	G1XAL6	91.6%	296	arrestin	*Orbilia oligospora*	nematodes	*Caenorhabditis elegans*
FUN_000980	P41388	98.0%	447	beta-tubulin	*Venturia inaequalis*		
FUN_000980	P53376	96.2%	447	beta-tubulin	*Rhynchosporium commune*	monocots	*Hordeum vulgare*
FUN_000980	Q7LLP2	97.7%	440	Beta2-tubulin housekeeping gene	*Blumeria graminis*	monocots	*Hordeum vulgare*
FUN_000980	Q7LLP2	97.7%	440	Beta2-tubulin housekeeping gene	*Blumeria graminis*	monocots	*Triticum aestivum*
FUN_000980	Q7Z7W3	97.7%	440	Beta2-tubulin housekeeping gene	*Blumeria graminis*	monocots	*Hordeum vulgare*
FUN_000980	Q7Z7W3	97.7%	440	Beta2-tubulin housekeeping gene	*Blumeria graminis*	monocots	*Triticum aestivum*
FUN_000980	Q7Z7T9	98.7%	398	beta-tubulin	*Monilinia fructicola*		
FUN_001426	T0LLS6	90.8%	195	Rho GTPase protein	*Colletotrichum gloeosporioides*	eudicots	*Populus x beijingensis*
FUN_001615	I1S1V9	92.4%	145	transcription factor	*Fusarium graminearum*	monocots	*Triticum*
FUN_001923	A0A0D2XC66	90.2%	174	calcineurin subunit B	*Fusarium oxysporum*	eudicots	*Solanum lycopersicum*
FUN_001923	G4MNE6	91.4%	174		*Magnaporthe oryzae*	monocots	*Hordeum vulgare*
FUN_001923	G4MNE6	91.4%	174		*Magnaporthe oryzae*	monocots	*Triticum*
FUN_001923	G4MNE6	91.4%	174		*Magnaporthe oryzae*	monocots	*Triticum*
FUN_003562	D3J127	91.4%	417	mitogen-activated protein (MAP) kinase	*Alternaria alternata*	eudicots	*Citrus x tangelo*
FUN_003562	A3QYW5	93.5%	400	MAP kinases	*Bipolaris maydis*	monocots	*Zea mays*
FUN_003562	A3QYW5	93.5%	400	MAP kinases	*Bipolaris maydis*	monocots	*Zea mays*
FUN_004271	Q9UWF0	100.0%	149		*Magnaporthe oryzae*	monocots	*Hordeum vulgare*
FUN_004271	Q9UWF0	100.0%	149		*Magnaporthe oryzae*	monocots	*Triticum*
FUN_004271	Q9UWF0	100.0%	149		*Magnaporthe oryzae*	monocots	*Triticum*
FUN_004756	A0A0D2XX76	93.8%	144	palmitoylation of FonAP-2 complex subunits	*Fusarium oxysporum*	eudicots	*Citrullus lanatus*
FUN_004756	Q4ICG5	93.1%	144	AP-2 complex subunit sigma	*Fusarium graminearum*	monocots	*Triticum aestivum*
FUN_005471	D3J126	96.1%	355	high-osmolarity glycerol response kinase	*Alternaria alternata*	eudicots	*Pyrus communis*
FUN_005471	Q0U4L8	96.1%	355	mitogen-activated protein kinase	*Parastagonospora nodorum*	monocots	*Triticum aestivum*
FUN_005471	Q4W6D3	95.5%	355	MAP kinases	*Bipolaris maydis*	monocots	*Zea mays*
FUN_005471	Q4W6D3	95.5%	355	MAP kinases	*Bipolaris maydis*	monocots	*Zea mays*
FUN_005471	M2SJ60	95.2%	355	mitogen-activated protein kinases	*Bipolaris sorokiniana*	monocots	*Hordeum vulgare*
FUN_005471	M2SJ60	95.2%	355	mitogen-activated protein kinases	*Bipolaris sorokiniana*	monocots	*Hordeum vulgare*
FUN_005471	Q1KTF2	93.6%	357	MAP kinase	*Zymoseptoria tritici*	monocots	*Triticum*
FUN_005471	B0XR80	94.8%	344	MAP kinase SakA	*Aspergillus fumigatus*	rodents	*Mus musculus*
FUN_005471	Q4WSF6	94.8%	344	MAPkinase	*Aspergillus fumigatus*	rodents	*Mus musculus*
FUN_005471	Q4WSF6	94.8%	344	MAPkinase	*Aspergillus fumigatus*	rodents	*Mus musculus*
FUN_005471	Q875L0	94.8%	345	MAP kinase	*Cryphonectria parasitica*	eudicots	*Castanea dentata*
FUN_005471	G2XG03	93.6%	346	encoding a homolog of a high-osmolarity glycerol (HOG) response mitogen-activated protein kinase	*Verticillium dahliae*	eudicots	*Cotinus obovatus*
FUN_005471	E9EYM7	94.2%	345	MAPK	*Metarhizium robertsii*	moths	*Galleria mellonella*
FUN_005471	Q9UV51	93.6%	345	MAP kinase	*Magnaporthe oryzae*	monocots	*Oryza sativa*
FUN_005471	P0C431	93.6%	345	MAPK	*Fusarium graminearum*	monocots	*Triticum*

Gene ID	Hit Protein	Similarity	Hit length	Function	Pathogen species	Host group	Host species
FUN_005471	P0C431	93.6%	345	MAPK	*Fusarium graminearum*	eudicots	*Solanum lycopersicum*
FUN_005471	A0A0D2XQS0	93.3%	345	MAP kinase	*Fusarium oxysporum*	eudicots	*Solanum lycopersicum*
FUN_005471	A0A0D2XQS0	93.3%	345	MAP kinase	*Fusarium oxysporum*	eudicots	*Solanum lycopersicum*
FUN_005471	A0A0D2XQS0	93.3%	345	MAP kinase	*Fusarium oxysporum*	eudicots	*Solanum lycopersicum*
FUN_005471	A0A0D2XQS0	93.3%	345	MAP kinase	*Fusarium oxysporum*	moths	*Galleria mellonella*
FUN_005471	A0A0D2XQS0	93.3%	345	MAP kinase	*Fusarium oxysporum*	moths	*Galleria mellonella*
FUN_005471	A0A0D2XQS0	93.3%	345	MAP kinase	*Fusarium oxysporum*	moths	*Galleria mellonella*
FUN_005471	Q6PWX2	92.2%	347	mitogen-activated protein kinase	*Beauveria bassiana*	moths	*Galleria mellonella*
FUN_005471	Q6PWX2	92.2%	347	mitogen-activated protein kinase	*Beauveria bassiana*	moths	*Galleria mellonella*
FUN_005471	K9FTS6	92.4%	344	mitogen-activated protein kinase	*Penicillium digitatum*	eudicots	*Citrus sinensis*
FUN_005471	K9FTS6	92.4%	344	mitogen-activated protein kinase	*Penicillium digitatum*	eudicots	*Citrus sinensis*
FUN_005471	A1IVT7	91.4%	347	stress activated MAPK	*Botrytis cinerea*	eudicots	*Phaseolus vulgaris*
FUN_005769	I1RMT4	93.7%	300	serine/threonine-protein phosphatase (EC 3.1.3.16)	*Fusarium graminearum*	monocots	*Triticum aestivum*
FUN_006039	F5HEA9	99.1%	350	MAP kinase	*Parastagonospora nodorum*	monocots	*Triticum*
FUN_006039	Q2TLM7	99.1%	350	mitogen activated protein kinase (MAPK)	*Parastagonospora nodorum*	monocots	*Triticum*
FUN_006039	Q6R3Q3	99.1%	350	Fus3/Kss1 MAP kinase homologue	*Alternaria brassicicola*	eudicots	*Brassica oleracea*
FUN_006039	Q6R3Q3	99.1%	350	Fus3/Kss1 MAP kinase homologue	*Alternaria brassicicola*	eudicots	*Brassica napus*
FUN_006039	Q6R3Q3	99.1%	350	Fus3/Kss1 MAP kinase homologue	*Alternaria brassicicola*	eudicots	*Brassica carinata*
FUN_006039	Q6R3Q3	99.1%	350	Fus3/Kss1 MAP kinase homologue	*Alternaria brassicicola*	eudicots	*Brassica juncea*
FUN_006039	Q96X31	99.1%	350	MAP kinase	*Pyrenophora teres*	monocots	*Hordeum vulgare*
FUN_006039	Q9UVN7	98.6%	350	MAP kinase	*Bipolaris maydis*	monocots	*Zea mays*
FUN_006039	D2IV71	95.7%	350		*Alternaria alternata*	eudicots	*Citrus x tangelo*
FUN_006039	Q9HG08	94.9%	352	MAP kinase	*Botrytis cinerea*	eudicots	*Dianthus caryophyllus*
FUN_006039	Q9HG08	94.9%	352	MAP kinase	*Botrytis cinerea*	eudicots	*Solanum lycopersicum*
FUN_006039	Q9HG08	94.9%	352	MAP kinase	*Botrytis cinerea*	eudicots	*Rosa*
FUN_006039	H1VA10	93.8%	355	mitogen-activated protein kinase	*Colletotrichum higginsianum*	eudicots	*Arabidopsis thaliana*
FUN_006039	L2GIQ8	93.8%	355	MAPK gene	*Colletotrichum fructicola*	eudicots	*Pyrus communis*
FUN_006039	Q9UW09	94.1%	355	MAP kinase	*Colletotrichum lagenaria*	eudicots	*Cucumis sativus*
FUN_006039	G4N0Z0	93.3%	356		*Magnaporthe oryzae*	monocots	*Oryza sativa*
FUN_006039	G4N0Z0	93.3%	356		*Magnaporthe oryzae*	monocots	*Hordeum vulgare*
FUN_006039	A0A194W6H1	93.5%	355	mitogen-activated protein kinase	*Valsa mali*	eudicots	*Malus domestica*
FUN_006039	A0A063C6H4	93.0%	355	CMGC kinase	*Ustilaginoidea virens*	monocots	*Oryza sativa*
FUN_006039	Q92246	93.0%	356	MAP kinase	*Magnaporthe oryzae*	monocots	*Oryza sativa*
FUN_006039	A0A0C4DHU3	93.0%	355	mitogen-activated protein kinase	*Fusarium oxysporum*	eudicots	*Solanum lycopersicum*
FUN_006039	Q9HGU2	93.0%	355	MAP kinase	*Fusarium oxysporum*	eudicots	*Solanum lycopersicum*
FUN_006039	Q9HGU2	93.0%	355	MAP kinase	*Fusarium oxysporum*	rodents	*Mus musculus*
FUN_006039	I1RQN9	93.0%	355	MAP kinase	*Fusarium graminearum*	monocots	*Triticum*
FUN_006039	I1RQN9	93.0%	355	MAP kinase	*Fusarium graminearum*	eudicots	*Solanum lycopersicum*
FUN_006039	I1RQN9	93.0%	355	MAP kinase	*Fusarium graminearum*	eudicots	*Solanum lycopersicum*
FUN_006039	I1RQN9	93.0%	355	MAP kinase	*Fusarium graminearum*	monocots	*Triticum*
FUN_006039	G2XH29	93.5%	353	putative MAPK gene	*Verticillium dahliae*	eudicots	*Gossypium hirsutum*
FUN_006039	Q7ZA49	92.4%	356		*Lecanicillium fungicola*	basidiomycetes	*Agaricus bisporus*
FUN_006039	E9EWV4	92.4%	355	MAPK	*Metarhizium robertsii*	moths	*Galleria mellonella*

Gene ID	Hit Protein	Similarity	Hit length	Function	Pathogen species	Host group	Host species
FUN_006039	E9DS25	92.4%	355	Fus3/Kss1-type mitogen-activated protein kinase (MAPK) gene	*Metarhizium acridum*	grasshoppers	*Locusta migratoria*
FUN_006039	Q96VL1	92.4%	355	MAP kinase	*Claviceps purpurea*	monocots	*Secale cereale*
FUN_006039	Q5EKM7	92.6%	353	MAP kinase	*Verticillium dahliae*	eudicots	*Capsicum frutescens*
FUN_006039	Q5EKM7	92.6%	353	MAP kinase	*Verticillium dahliae*	eudicots	*Lactuca sativa*
FUN_006039	Q5EKM7	92.6%	353	MAP kinase	*Verticillium dahliae*	eudicots	*Gossypium hirsutum*
FUN_006039	Q5EKM7	92.6%	353	MAP kinase	*Verticillium dahliae*	eudicots	*Citrullus lanatus*
FUN_006039	Q5EKM7	92.6%	353	MAP kinase	*Verticillium dahliae*	eudicots	*Cynara cardunculus*
FUN_006039	Q5EKM7	92.6%	353	MAP kinase	*Verticillium dahliae*	eudicots	*Solanum lycopersicum*
FUN_006039	Q5EKM7	92.6%	353	MAP kinase	*Verticillium dahliae*	eudicots	*Solanum melongena*
FUN_006039	K9FDB7	92.5%	346	mitogen-activated protein kinase	*Penicillium digitatum*	eudicots	*Citrus reticulata*
FUN_006039	Q7Z8D0	91.0%	356	mitogen-activated protein kinase	*Beauveria bassiana*	butterflies	*Pieris brassicae*
FUN_006676	B0Y4S0	91.1%	316	governs growth and development	*Aspergillus fumigatus*	rodents	*Mus musculus*
FUN_006676	B0Y4S0	91.1%	316	governs growth and development	*Aspergillus fumigatus*	rodents	*Mus musculus*
FUN_006676	Q4WQK8	91.1%	316	involved in antifungal drug susceptibility and virulence	*Aspergillus fumigatus*	rodents	*Mus musculus*
FUN_006772	G4MVC5	91.1%	135	ubiquitin ligase	*Magnaporthe oryzae*	monocots	*Oryza sativa*
FUN_006772	G4MVC5	91.1%	135	ubiquitin ligase	*Magnaporthe oryzae*	monocots	*Hordeum vulgare*
FUN_007210	Q5J4D6	92.1%	543	malate synthase	*Parastagonospora nodorum*	monocots	*Triticum*
FUN_007538	A0A384K6F2	99.5%	375	conserved actin protein	*Botrytis cinerea*	eudicots	*Solanum lycopersicum*
FUN_007538	A0A384K6F2	99.5%	375	conserved actin protein	*Botrytis cinerea*	eudicots	*Malus domestica*
FUN_007596	Q4WKG5	93.1%	450	tubulin alpha-1 chain	*Aspergillus fumigatus*		
FUN_007596	Q4WKG5	93.1%	450	tubulin alpha-1 chain	*Aspergillus fumigatus*	rodents	*Mus musculus*
FUN_007825	A0A177DD11	99.2%	118	ubiquitin E3 ligase	*Alternaria alternata*	eudicots	*Citrus x microcarpa*
FUN_007825	N4X184	99.2%	118	ubiquitin-like autophagy protein	*Bipolaris maydis*	monocots	*Zea mays*
FUN_007825	A6RPU4	97.5%	118	autophagy-related protein	*Botrytis cinerea*	eudicots	*Solanum lycopersicum*
FUN_007825	A6RPU4	97.5%	118	autophagy-related protein	*Botrytis cinerea*	eudicots	*Cucumis sativus*
FUN_007825	A6RPU4	97.5%	118	autophagy-related protein	*Botrytis cinerea*	eudicots	*Malus domestica*
FUN_007825	A6RPU4	97.5%	118	autophagy-related protein	*Botrytis cinerea*	eudicots	*Vitis vinifera*
FUN_007825	A0A0D2Y8P9	97.5%	118	ubiquitin-like protein conjugated to PE involved in phagophore expansion	*Fusarium oxysporum*	eudicots	*Solanum lycopersicum*
FUN_007825	A0A0D2Y8P9	97.5%	118	ubiquitin-like protein conjugated to PE involved in phagophore expansion	*Fusarium oxysporum*	moths	*Galleria mellonella*
FUN_007825	W7MMQ7	97.5%	118	autophagy-related protein	*Fusarium verticillioides*	monocots	*Zea mays*
FUN_007825	W7MMQ7	97.5%	118	autophagy-related protein	*Fusarium verticillioides*	monocots	*Zea mays*
FUN_007825	H9B3V9	98.3%	117	autophagy	*Verticillium dahliae*	eudicots	*Arabidopsis thaliana*
FUN_007825	A0A4T0VYD8	99.1%	116	autophagy	*Colletotrichum higginsianum*	eudicots	*Arabidopsis thaliana*
FUN_007825	A7E8H4	96.6%	118	autophagy-related protein 8	*Sclerotinia sclerotiorum*	eudicots	*Solanum lycopersicum*
FUN_007825	A0A063BP02	98.3%	116	microtubule-associated protein	*Ustilaginoidea virens*	monocots	*Oryza sativa*
FUN_007825	J4UTT5	98.3%	116	autophagy-related gene 8	*Beauveria bassiana*	moths	*Galleria mellonella*
FUN_007825	Q51MW4	94.9%	117		*Magnaporthe oryzae*	monocots	*Oryza sativa*
FUN_007825	B6VCT7	94.0%	117	autophagy	*Magnaporthe oryzae*	monocots	*Oryza sativa*
FUN_008964	U3Q4A0	94.6%	552	calcineurin phosphatase	*Alternaria alternata*	eudicots	*Citrus x microcarpa*
FUN_009213	G2YY48	90.8%	185	Rac-like GTPase	*Botrytis cinerea*	eudicots	*Phaseolus vulgaris*
FUN_009457	Q6XSF5	96.7%	275	G beta protein subunit	*Bipolaris maydis*	monocots	*Zea mays*

Gene ID	Hit Protein	Similarity	Hit length	Function	Pathogen species	Host group	Host species
FUN_009503	D2X3B5	98.9%	353	class I Ga subunit of the GTP-binding protein	*Alternaria alternata*	eudicots	*Citrus x tangelo*
FUN_009503	O74227	98.9%	353	G protein alpha subunit	*Bipolaris maydis*	monocots	*Zea mays*
FUN_009503	I1RNF9	94.9%	353	G protein alpha subunit	*Fusarium graminearum*	monocots	*Hordeum vulgare*
FUN_009503	Q96VA7	94.9%	353	G alpha protein subunit	*Fusarium oxysporum*	eudicots	*Solanum lycopersicum*
FUN_009503	Q00580	94.6%	353	G alpha protein subunit	*Cryphonectria parasitica*	eudicots	*Castanea dentata*
FUN_009503	O42784	94.3%	353	G alpha protein subunit	*Colletotrichum trifolii*	eudicots	*Medicago sativa*
FUN_009503	Q875J4	94.3%	353	G alpha protein subunit	*Trichoderma virens*	basidiomycetes	*Agroathelia rolfsii*
FUN_009503	B2Z3E2	94.3%	353	encodes a Ga sub-unit that regulates sexual and asexual development and secondary metabolism	*Colletotrichum graminicola*	monocots	*Zea mays*
FUN_009503	O13315	94.1%	353	G alpha protein subunit	*Magnaporthe oryzae*	monocots	*Oryza sativa*
FUN_009503	Q9HEP9	94.6%	353	G alpha protein subunit	*Botrytis cinerea*	eudicots	*Solanum lycopersicum*
FUN_009503	Q9HEP9	94.6%	353	G alpha protein subunit	*Botrytis cinerea*	eudicots	*Phaseolus vulgaris*
FUN_009503	Q6EH42	96.6%	349	G alpha protein subunit	*Parastagonospora nodorum*	monocots	*Triticum*
FUN_009613	Q6S9D7	92.1%	240	adhesin	*Paracoccidioides brasiliensis*	moths	*Galleria mellonella*
FUN_009762	G4MYQ8	90.6%	138	ER-derived vesicles protein ERV14	*Magnaporthe oryzae*	monocots	*Oryza sativa*
FUN_009762	G4MYQ8	90.6%	138	ER-derived vesicles protein ERV14	*Magnaporthe oryzae*	monocots	*Hordeum vulgare*
FUN_009762	G4MYQ8	90.6%	138	ER-derived vesicles protein ERV14	*Magnaporthe oryzae*	monocots	*Oryza sativa*
FUN_009762	G4MYQ8	90.6%	138	ER-derived vesicles protein ERV14	*Magnaporthe oryzae*	monocots	*Hordeum vulgare*
FUN_009762	A0A2H3GEQ4	91.2%	137	endoplasmic reticulum cargo receptor	*Fusarium graminearum*	monocots	*Triticum aestivum*
FUN_009919	Q4WJS6	96.2%	316	serine/threonine-protein phosphatase	*Aspergillus fumigatus*	rodents	*Mus musculus*
FUN_010083	I1RC95	90.3%	390	transcription factor	*Fusarium graminearum*	monocots	*Triticum*
FUN_011024	Q4HTT1	91.7%	132	transcription factor	*Fusarium graminearum*	monocots	*Triticum*
FUN_011931	A9Z1V6	90.4%	405	calcium/calmodulin-dependent kinase	*Parastagonospora nodorum*	monocots	*Triticum*

Table S4 Predicted secondary metabolite biosynthetic gene clusters in E00680

Scaffold	Region	From	To	Type	Most similar known cluster	Similarity
2	1	247,369	291,244	NRPS-like	choline	100%
2	2	992,161	1,037,645	T1PKS	neosartorin	21%
18	1	142,794	164,422	terpene	squalestatin S1	40%
23	1	257,174	303,777	T1PKS	1,3,5,8-tetrahydroxynapthalene/1-(alpha-l-(2-O-methyl)-6-deoxymannopyranosyloxy)-3,6,8-trimethoxynaphthalene	100%
26	1	157,424	204,673	T1PKS	4-epi-15-epi-brefeldin A	20%
37	1	206,485	249,295	NRPS-like	aspulvinone H/aspulvinone B1	100%
44	4	3,048	48,871	NRPS	metachelin C/metachelin A/metachelin A-CE/metachelin B/dimerumic acid 11-mannoside/dimerumic acid	50%

Table S5 Predicted virulence-associated genes in E00680

Gene ID	Similarity	Uniprot index	Organism	Pfam ID	Description	Host	Disease
FUN_000616	90.8%	C5GVY6_AJEDR	*Ajellomyces dermatitidis*	PF00091	Tubulin	vertebrata	cutaneous Infection
FUN_000980	98.7%	Q7Z7T9_MONFR	*Monilinia fructicola*	PF00091	Tubulin	xyloid	Brown Fruit Rot
FUN_005471	94.8%	HOG1_CRYPA	*Cryphonectria parasitica*	PF00069	Pkinase	xyloid	Canker
FUN_006039	99.1%	Q6R3Q3_ALTBR	*Alternaria brassicicola*	PF00069	Pkinase	herb	Leaf Spot
FUN_007210	92.1%	Q5J4D6_PHAND	*Phaeosphaeria nodorum*	PF01274	Malate_synthase	herb	Glume Blotch
FUN_007596	93.1%	Q4WKG5_ASPFU	*Neosartorya fumigata*	PF00091	Tubulin	vertebrata	Infection
FUN_009457	96.7%	Q6XSF5_COCHE	*Cochliobolus heterostrophus*	PF00400	WD40	herb	Leaf Blight
FUN_009503	96.6%	Q6EH42_PHAND	*Phaeosphaeria nodorum*	PF00503	G-alpha	herb	Glume Blotch
FUN_011931	90.4%	A9Z1V6_PHAND	*Phaeosphaeria nodorum*	PF00069	Pkinase	herb	Glume Blotch

Table S6 Up-regulated metabolites in wheat plants after E00680 inoculation under salt stress

Group	Metabolite	Fold change
Organoheterocyclic compounds	DL-Tryptophan	2.38
	Skatole	2.3
	Indole-3-acrylic acid	2.29
	Dl-Indole-3-lactic acid	2.29
	Indole	2.27
	trans-3-Indoleacrylic acid	1.83
	5-Hydroxytryptophan	1.59
	2-{(3S)-1-[4-(Methylsulfonyl)benzyl]-3-pyrrolidinyl}-1,3-benzothiazole	1.33
	1-Methylguanine	1.28
	Adenine	1.28
	3-Hydroxypicolinic acid	1.26
	Indole-3-pyruvate	1.24
	L-5-Hydroxytryptophan	1.23
	Indole-3-pyruvic acid	1.23
	Xanthurenic acid	1.21
Lipids and lipid-like molecules	Geranyl monophosphate	1.62
	LPI 18:3	1.53
	8-Epiloganic acid	1.42
	Auraptene	1.4
	LPI 18:2	1.33
	Yuheinoside	1.3
	All trans retinal	1.28
	11-Deoxy prostaglandin F1β	1.27
	(R)-3-Hydroxy myristic acid	1.26
	Methyl jasmonate	1.25
	Methyl linolenate	1.25
	2-Methylpentanedioic acid	1.24
	trans-beta-Ionone	1.21
Benzenoids	N4-Acetylsulfamethoxazole	3.48
	Efetaal	1.44
	Shikonin	1.44
	4-Methoxybenzaldehyde	1.44
	4-Methylphenol	1.31
	Tyramine	1.27
	5-Methoxysalicylic acid	1.25
Nucleosides, nucleotides, and analogues	NAD+	2.7
	UDP-N-acetylglucosamine	2.55
	Uridine 5'-monophosphate	1.52
	Xanthosine	1.29
Organic acids and derivatives	L-Mimosine	1.77
	Nicotianamine	1.37
	N-Acetyl-DL-glutamic acid	1.37
	L-Histidine	1.33
Phenylpropanoids and polyketides	Isomucronulatol-7-O-glucoside	2.91
	Coumarin	1.47
	Neosaxitoxin	1.43
	Rhamnetin	1.3
Organic oxygen compounds	DIMBOA glucoside	1.32
	(2R)-2,3-Dihydroxypropanoic acid	1.29
	Zeatin-7-N-glucoside	1.21

Table S7 Up-regulated metabolites in rhizosphere soil after E00680 inoculation under salt stress

Group	Metabolite	Fold change
Lipids and lipid-like molecules	Lactitol	13.22
	Dihydroroseoside	6.08
	LPE 17:2	4.75
	Prostaglandin A2	3.61
	LPE 18:2	2.64
	Tetrahydrocorticosterone	2.44
	LPG 17:1	2.4
	LPI 18:3	2.31
	15(S)-HpETE	2.25
	LPG 18:3	2.15
	Corchorifatty acid F	2.01
	Prostaglandin E3	1.87
	LPG 15:1	1.85
	4-Hydroxyretinoic Acid	1.8
	Diethyl succinate	1.79
	Methyl dihydrojasmonate	1.69
	LPI 15:1	1.69
	3-Methoxy prostaglandin F1α	1.66
	2-Hydroxy-3-methylbutanoic acid	1.66
	Gibberellic acid	1.65
	19-Nortestosterone	1.62
	Prostaglandin F1α	1.55
	LPI 14:0	1.5
	13,14-Dihydro-15-keto Prostaglandin E2	1.49
	Prostaglandin D1	1.49
	Prostaglandin E2	1.48
	LPI 13:0	1.39
	Undecanedioic acid	1.35
	2-Methylbutyroylcarnitine	1.34
	6-Ketoprostaglandin F1α	1.33
	Tretinoin	1.26
Organic acids and derivatives	Terephthalic Acid	6.07
	gamma-Glutamylmethionine	3.74
	N-Acetyl-DL-tryptophan	2.3
	Cyclohexylsulfamate	1.88
	Dl-Lanthionine	1.79
	N-Acetylvaline	1.7
	Diethyloxalpropionate	1.48
	Oxaceprol	1.46
	D-2-Aminoadipic acid	1.35
Organoheterocyclic compounds	Riboflavin	2.63
	6-Hydroxymelatonin	1.86
	DL-Tryptophan	1.66
	5-Methoxyindole-3-Carbaldehyde	1.66
	gamma-Nonanolactone	1.58
	2-Amino-6-methylmercaptopurine	1.49
	Corey Lactone Diol	1.39
	Neopterin	1.36
Benzenoids	Monobutyl phthalate	22.64
	2-Naphthol	2.19
	2-Anisic acid	1.8
	2,4-Dinitrophenol	1.73
	5-Aminosalicylic Acid	1.56
	4-Butylresorcinol	1.46
	Asaraldehyde	1.43
	4-Hydroxy-3-methylbenzoic acid	1.26

Group	Metabolite	Fold change
Organic oxygen compounds	D-Raffinose	3.43
	Ribulose-5-phosphate	3.26
	L-Kynurenine	2.69
	D-(+)-Maltose	2.01
	Pantothenic acid	1.81
	Sucrose	1.75
Phenylpropanoids and polyketides	Hesperetin	1.88
	DL-4-Hydroxyphenyllactic acid	1.84
	Ferulic acid	1.64
	alpha-Benzylsuccinic acid	1.39
	2-Phenylpropionic acid	1.27

Table S8 Key genes related to E00680-mediated salt stress tolerance

Gene ID	Scaffold	Start	Stop	Strand	Symbol	Annotation
FUN_002026	contig_2	1793959	1794597	+	AQY	Aquaporin
FUN_002071	contig_2	1939316	1939890	+	AQY	Aquaporin
FUN_009085	contig_16	133744	134794	+	AQY	Aquaporin
FUN_000816	contig_1	2554150	2560663	+	CCH1	calcium channel protein
FUN_004857	contig_6	1315025	1318898	-	PMC	plasma membrane calcium
FUN_005780	contig_8	578203	582471	+	PMC	plasma membrane calcium
FUN_007576	contig_12	225581	229360	+	PMC	plasma membrane calcium
FUN_008865	contig_15	554422	557047	+	TRK1	low affinity potassium transporter
FUN_003269	contig_4	454080	456120	+	KCH1	Potassium transporter
FUN_005323	contig_7	768151	770641	-	TOK1	Potassium channel
FUN_005381	contig_7	917474	919833	-	TOK1	Potassium channel
FUN_001752	contig_2	1048428	1050353	+	CNH1	Na+/H+ antiporter
FUN_002385	contig_2	2841757	2845098	-	CNH1	Na+/H+ antiporter
FUN_013109	contig_46	77951	82930	+	CTA3	potassium/sodium efflux ATPase
FUN_006740	contig_10	365913	367357	-	VCX1	Vacuolar calcium ion transporter
FUN_008358	contig_14	134585	136797	+	NHX1	monovalent cation:H+ antiporter
FUN_010821	contig_22	542253	542884	+	GPX	Glutathione peroxidase 2
FUN_001943	contig_2	1587666	1588931	-	GTT1	bifunctional glutathione transferase/peroxidase
FUN_003514	contig_4	1191553	1192620	+	GST2	Glutathione S-transferase 2
FUN_008019	contig_13	318590	319443	+	GST2	Glutathione S-transferase 2
FUN_001528	contig_2	365527	367017	-	GLR	Glutathione reductase
FUN_009702	contig_17	906837	909095	+	GLR	Glutathione reductase
FUN_008451	contig_14	453948	455524	-	GSH2	Glutathione synthetase
FUN_009940	contig_18	653108	653732	+	SOD	Superoxide dismutase [Mn], mitochondrial
FUN_001312	contig_1	4130892	4131742	+	SOD	Superoxide dismutase [Cu-Zn]
FUN_006866	contig_10	758298	758951	-	SOD	Superoxide dismutase [Mn], mitochondrial
FUN_000970	contig_1	3019954	3020850	-	SOD	Cell surface superoxide dismutase [Cu-Zn]
FUN_012397	contig_36	89221	90093	-	SOD	Cell surface superoxide dismutase [Cu-Zn]
FUN_002351	contig_2	2736413	2738743	-	CAT	catalase
FUN_004609	contig_6	577295	578906	-	CAT	catalase
FUN_000245	contig_1	830922	832625	-	ARO	Aromatic/aminoadipate aminotransferase
FUN_004136	contig_5	1034397	1036032	+	TDC	aromatic-L-amino-acid/L-tryptophan decarboxylase
FUN_003657	contig_4	1615958	1617590	+	ALDH	aldehyde dehydrogenase
FUN_000781	contig_1	2435575	2437267	-	ALDH	aldehyde dehydrogenase
FUN_005051	contig_6	1845683	1847222	-	ALDH	aldehyde dehydrogenase
FUN_002182	contig_2	2265254	2266650	-	NIT	Nitrilase
FUN_005033	contig_6	1790334	1791547	+	NIT	Nitrilase
FUN_003876	contig_5	298562	300725	-	AMD	amidase
FUN_000902	contig_1	2835429	2836009	+	AMD	amidase
FUN_000418	contig_1	1285353	1287256	-	AMD	amidase
FUN_003196	contig_4	208067	209232	+	AMD	amidase
FUN_003344	contig_4	689211	691092	-	AMD	amidase
FUN_004887	contig_6	1401599	1403284	-	AMD	amidase
FUN_000901	contig_1	2834247	2834853	+	AMD	amidase
FUN_002172	contig_2	2233166	2235065	+	AMD	amidase
FUN_003144	contig_4	46260	47718	+	AMD	amidase
FUN_003957	contig_5	550770	552856	+	AMD	amidase
FUN_007076	contig_11	61961	65534	-	CYPA1	cytochrome P450 family 1 subfamily A1
FUN_004334	contig_5	1611513	1613053	+	CYPA1	cytochrome P450 family 1 subfamily A1
FUN_001497	contig_2	271982	274843	-	CYPA1	cytochrome P450 family 1 subfamily A1

Table S9 BLAST-based comparison of key genes associated with salt tolerance in E00680 and *Serendipita indica*

Query ID	Subjec ID	Identity	Alignment	E-value	BitScore	Descriptions in *Serendipita indica* DSM 11827
FUN_002351-T1	CAG7854074.1	58.57	729	0.00E+00	858	Catalase-1
FUN_008358-T1	CAG7852884.1	57.96	440	4.34E-162	479	Uncharacterized Na(+)/H(+) antiporter
FUN_006740-T1	CAG7849510.1	53.68	367	1.70E-122	361	Vacuolar calcium ion transporter
FUN_000781-T1	CAG7846154.1	53.66	505	0.00E+00	523	Aldehyde dehydrogenase
FUN_001312-T1	CAG7852205.1	52.63	114	3.11E-32	114	Superoxide dismutase [Cu-Zn]
FUN_007576-T1	CAG7849261.1	50.77	1111	0.00E+00	1025	Calcium-transporting ATPase 2
FUN_009702-T1	CAG7846696.1	50.20	490	4.66E-142	426	Glutathione reductase
FUN_010821-T1	CAG7849533.1	49.72	181	3.04E-57	176	Glutathione peroxidase 2
FUN_005780-T1	CAG7849261.1	48.93	1122	0.00E+00	993	Calcium-transporting ATPase 2
FUN_006866-T1	CAG7851187.1	48.86	176	2.36E-53	167	Superoxide dismutase [Mn], mitochondrial
FUN_001528-T1	CAG7846696.1	48.70	460	2.00E-139	420	Glutathione reductase
FUN_009940-T1	CAG7851187.1	47.73	176	9.81E-49	156	Superoxide dismutase [Mn], mitochondrial
FUN_004857-T1	CAG7849261.1	47.69	1059	0.00E+00	907	Calcium-transporting ATPase 2
FUN_003514-T1	CAG7852492.1	47.01	217	1.70E-63	198	Disulfide-bond oxidoreductase YfcG
FUN_008019-T1	CAG7852492.1	45.81	203	3.90E-56	178	Disulfide-bond oxidoreductase YfcG
FUN_002385-T1	CAG7846549.1	45.06	466	2.29E-119	385	Na(+)/H(+) antiporter
FUN_005051-T1	CAG7846154.1	40.62	485	2.17E-113	341	Aldehyde dehydrogenase
FUN_002071-T1	CAG7850958.1	40.57	106	1.39E-18	78	Related to aquaporin
FUN_001497-T1	CAG7851386.1	40.26	914	0.00E+00	669	Bifunctional cytochrome P450/NADPH--P450 reductase 2
FUN_008451-T1	CAG7852736.1	39.82	540	8.61E-103	317	Glutathione synthetase
FUN_003657-T1	CAG7851991.1	39.56	407	8.85E-99	302	Aldehyde dehydrogenase family 3 member B1
FUN_009085-T1	CAG7850958.1	39.13	230	2.88E-46	155	Related to aquaporin
FUN_004609-T1	CAG7854074.1	39.11	427	4.30E-93	296	Catalase-1
FUN_013109-T1	CAG7849245.1	38.02	1123	0.00E+00	719	Sodium transport ATPase 1
FUN_003344-T1	CAG7847927.1	37.95	585	7.73E-118	363	Acetamidase
FUN_000418-T1	CAG7853805.1	37.77	503	2.53E-104	323	Acetamidase
FUN_007076-T1	CAG7851386.1	36.52	1076	0.00E+00	630	Bifunctional cytochrome P450/NADPH--P450 reductase 2
FUN_004887-T1	CAG7847927.1	36.24	574	9.54E-104	327	Acetamidase
FUN_004136-T1	CAG7846363.1	35.58	520	9.53E-110	334	Tyrosine decarboxylase 1
FUN_002026-T1	CAG7850958.1	35.21	71	8.97E-06	41	Related to aquaporin
FUN_001943-T1	CAG7847067.1	34.87	238	3.72E-36	126	Related to glutathione S-transferase
FUN_000816-T1	CAG7853715.1	33.98	1898	0.00E+00	992	Calcium-channel protein cch1
FUN_001752-T1	CAG7846549.1	33.49	421	6.26E-70	240	Na(+)/H(+) antiporter
FUN_000245-T1	CAG7848863.1	32.17	516	2.59E-84	276	Aromatic/aminoadipate aminotransferase 1
FUN_003957-T1	CAG7846928.1	32.14	168	1.85E-11	65	Glutamyl-tRNA(Gln) amidotransferase subunit A, mitochondrial
FUN_008865-T1	CAG7854666.1	31.64	828	3.96E-110	357	Potassium transport protein 2
FUN_000901-T1	CAG7847927.1	31.46	89	3.93E-06	44	Acetamidase
FUN_003144-T1	CAG7846928.1	30.74	270	2.43E-18	86	Glutamyl-tRNA(Gln) amidotransferase subunit A, mitochondrial
FUN_003269-T1	CAG7848647.1	28.36	275	8.12E-23	100	Uncharacterized protein
FUN_003196-T1	CAG7849342.1	28.02	339	1.90E-19	87	Cytochrome c1, heme protein, mitochondrial
FUN_003876-T1	CAG7854551.1	27.03	296	1.66E-16	81	Putative amidase PB8B6.03
FUN_005323-T1	CAG7847745.1	27.02	396	5.54E-29	121	Related to TOK1-Voltage-gated, outward-rectifying K+ channel protein of the plasma
FUN_002172-T1	CAG7854551.1	26.32	247	2.64E-15	77	Putative amidase PB8B6.03
FUN_004334-T1	CAG7853420.1	26.05	238	4.51E-12	66	Ras-related protein Rab-8A Flags: Precursor
FUN_005381-T1	CAG7847745.1	25.61	289	6.04E-16	80	Related to TOK1-Voltage-gated, outward-rectifying K+ channel protein of the plasma

Table S10 Primer sequences used for PCR and qPCR validation

Gene	Forward	Reverse
TraesCS2B02G355900	CGGCAATATTCGAGCGTGTG	GACTCCAGCTCAGCCATCTC
TraesCS2D02G381000	AAACATCGTTCCAACACTTCCC	TTAAGTCCACGACACCCTCC
TraesCS7A02G531800	GAACCTGTGAACAGCCTCCTG	ATCTGATGGACCACGTGAGAAT
TraesCS7B02G321300	AGAAGAAGAAGGCCCACACG	AGGGAAAGCACCAAGCTCTC
TraesCS7B02G448000	CCTCCATGGGCAGCTACTTC	CTCTTGCTCCTGGAGGTGAC
TraesCS6A02G216500	CTTCTCTCCATCCGGTACGC	CGTTGATGATCTTGGCGTCG
TraesCS4A02G245100	CTGGCTTTACAGCGACGAGA	AAAGAGCAGCAAACATGGCG
TraesCS7D02G155900	CCCGGAATCGATCGAGGAAG	GCAACATCTGCTTTGGCTCC
TraesCS1A02G291200	TGGCCTCGAAAGGATCAAATC	CCAGGCCATGGCAACCATAA
TraesCS6A02G175800	ATTTGGGCGATCTGGAGGTG	CCTCACTTGGCCCTCTTCTG
TraesCS7A02G530500	AGGTGAGCATTCCATCGTCC	CATTGAAGGACCAACACCGC
TraesCS5B02G332500	ACCGTCATCGGCTACAAGAG	GACGAGGACGAAGATGGTGG
TraesCS7D02G089800	CGGGTGGAGATTGTGTCGAT	CAATTACACGACGCCACGAC
E00680-*tub2*	AATTGGTGCTGCCTTCTGGT	CACTCCGGATCCATCGAGAC
Wheat *β-tubulin*	AGTTCACGGCCATGTTCA	ACGAGGTCGTTCATGTTGCT

Table S11 GenBank accession numbers for phylogenetic analyses

**Fungal strain**	***LSU***	***SSU***	***ITS***	***tub2***
Amniculicola parva CBS 123092 (outer group)	NG_056970.1	NG_016504.1	MH863272.1	NA
Polyplosphaeria fusca KT 1616	AB524604.1	AB524463.1	AB524789.1	AB524851.1
Quadricrura bicornis CBS 125427	AB524613.1	AB524472.1	AB524797.1	AB524859.1
Quadricrura meridionalis CBS 125684	AB524614.1	AB524473.1	AB524798.1	AB524860.1
Quadricrura septentrionalis CBS 125429	AB524615.1	AB524474.1	AB524799.1	AB524861.1
Ernakulamia cochinensis MFLUCC 18 1237	MN913716.1	MT864326.1	MT627670.1	NA
Ernakulamia krabiensis MFLUCC 18 0237	MK347990.1	MK347880.1	MK347773.1	NA
Ernakulamia tanakae NFCCI 4615	MN937211.1	NA	MN937229.1	MN938312.1
Triplosphaeria acuta KT 1170	AB524633.1	AB524492.1	AB524809.1	AB524871.1
Triplosphaeria cylindrica KT 1800	AB524635.1	AB524494.1	AB524810.1	AB524872.1
Triplosphaeria maxima KT 870	AB524637.1	AB524496.1	AB524812.1	AB524874.1
Triplosphaeria yezoensis KT 1715	AB524638.1	AB524497.1	AB524813.1	AB524875.1
Shrungabeeja aquatica MFLUCC 18 0664	MT627663.1	NA	MT627722.1	NA
Shrungabeeja longiappendiculata BCC 76463	KT376472.1	KT376471.1	KT376474.1	NA
Shrungabeeja vadirajensis MFLUCC 17 2362	MN913685.1	NA	MT627681.1	NA
Pseudotetraploa bambusicola CGMCC 3.20939	ON332933.1	ON332923.1	ON332915.1	NA
Pseudotetraploa curviappendiculata HC 4930	AB524608.1	AB524467.1	AB524792.1	AB524854.1
Pseudotetraploa javanica HC 4934	AB524611.1	AB524470.1	AB524795.1	AB524857.1
Pseudotetraploa longissima HC 4933	AB524612.1	AB524471.1	AB524796.1	AB524858.1
Tetraploa aquatica MFLU 19 0996	MT530453.1	MT530454.1	MT530449.1	NA
Tetraploa aristata CBS 996.70	AB524627.1	AB524486.1	AB524805.1	AB524867.1
Tetraploa bambusae KUMCC 21 0844	ON077067.1	ON077073.1	ON077078.1	ON075065.1
Tetraploa dwibahubeeja NFCCI 4621	MN937207.1	NA	MN937225.1	MN938308.1
Tetraploa endophytica CBS 147114	MW659165.1	NA	KT270279.1	NA
Tetraploa juncicola CBS 149046	ON603800.1	NA	ON603780.1	NA
Tetraploa nagasakiensis KT 1682	AB524630.1	AB524489.1	AB524806.1	AB524868.1
Tetraploa pseudoaristata NFCCI 4624	MN937214.1	NA	MN937232.1	MN938315.1
Tetraploa sasicola KT 563	AB524631.1	AB524490.1	AB524807.1	AB524869.1
Tetraploa thailandica MFLUCC 21 0030	MZ412530.1	MZ413274.1	MZ412518.1	NA
Tetraploa thrayabahubeeja NFCCI 4627	MN937217.1	NA	MN937235.1	MN938318.1
Tetraploa yunnanensis MFLUCC 19 0319	MN913735.1	MT864341.1	MT627743.1	NA
Tetraploa yakushimensis KT 1906	AB524632.1	AB524491.1	AB524808.1	AB524870.1
Tetraploa cylindrica KUMCC 20 0205	MT893204.1	MT893203.1	MT893205.1	MT899417.1
Tetraploa dashaoensis KUMCC 21 0010	OL473555.1	OL473556.1	OL473549.1	OL505601.1
Tetraploa obpyriformis KUMCC 21 0011	OL473554.1	OL473557.1	OL473558.1	OL505600.1
Tetraploa wurfbainiae MHZU 23 0231	OR626041.1	OR626044.1	OR626040.1	OR653395.1
